# Dehydrogenative Photocyclization of 3-Styryl Indoles to Fused Indole Systems

**DOI:** 10.1021/acs.joc.4c02103

**Published:** 2024-11-07

**Authors:** Yunus Taskesenligil, Nurullah Saracoglu

**Affiliations:** † Department of Chemistry, Faculty of Sciences, Atatürk University, 25240 Erzurum, Türkiye; ‡ Biotechnology Institute, Ankara University, 06135 Ankara, Türkiye

## Abstract

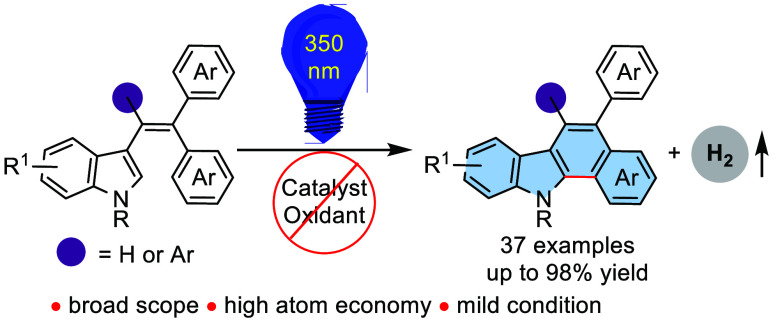

The synthesis of 5-aryl- or 5,6-diaryl-11*H*-benzo[*a*]carbazoles has been achieved from 3-styryl indoles through catalyst-free photoinitiated dehydrogenative cyclization transformation, providing a range of structurally diverse products in excellent yields under mild conditions. The protocol is also applicable to furan and thiophene samples. This Mallory-type reaction takes place in an argon atmosphere without external oxidants. Finally, detailed mechanistic studies were performed, and kinetic isotope effect experiments indicate that dehydrogenative annulation reaction involves photoinduced 6π-electrocyclic ring-closing and hydrogen evolution cascade processes.

## Introduction

**Figure 1:**
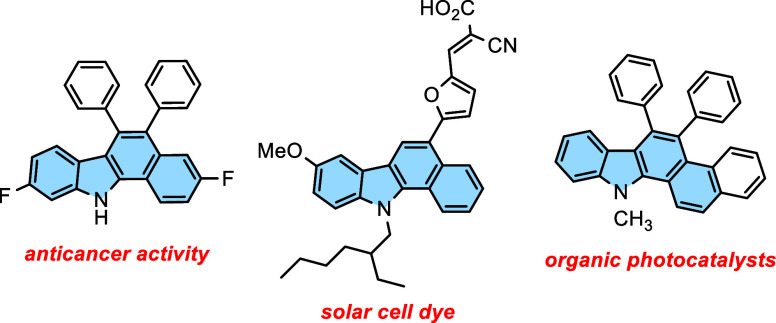
Representative benzo[*a*]carbazoles.

Carbazole is an important heterocyclic moiety that is commonly found in natural products, clinical medicine, drug discovery, and materials science.^1,2^ Particularly, benzo-annulated carbazole ring systems and large π-conjugated systems are key components in a large number of compounds employed in applications ranging from organic materials to biomedicine. For example, this structural motif has diverse biological activities such as anticancer,^3^ antifungal,^4^ and antiestrogenic properties,^5^ among others (Figure 1). However, the functionalized benzo[*a*]carbazoles are attracted a great deal as the key building blocks for the design and the construction of organic electronic and optoelectronic materials such as light-emitting diodes, organic photovoltaic cells (OPV), organic field-effect transistors, and dye-sensitized solar cells (Figure 1).^6^ Recently, Maji et al. prepared a series of new naphthocarbazole derivatives as organo-photocatalysts and demonstrated their applicability as catalysts in reductive dehalogenative borylation, phosphorylation, and dehydrohalide intramolecular C–C coupling reactions, as well as the dimerization of carbonyls and imines (Figure 1).^7^

Owing to their importance, the development of new and efficient processes has always been a vibrant field for the synthesis of fused carbazole derivatives. Besides the classic Fischer indolization,^8^ transition-metal-catalyzed cross-coupling reactions of prefunctionalized *o*-ethynylanilines,^9^ alkynyl arylazides,^10^ indole derivatives,^11^ alkyne annulation,^3b^ or metal-free visible-light-induced direct benzylic C(sp^3^)–H activation from tetrahydronaphthalene and arylhydrazine hydrochlorides using molecular oxygen as a green oxidant^12^ have been also utilized for accessing benzo[*a*]carbazoles.

**Scheme 1:**
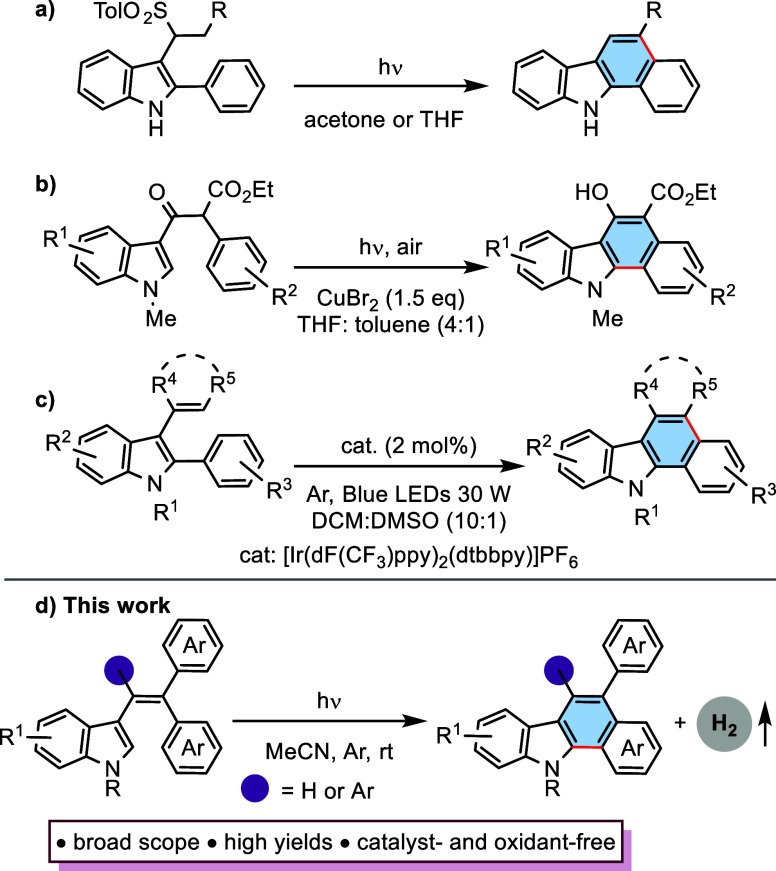
Synthesis of Benzo[*a*]carbazoles via Photochemical Cyclization

Also, the C–H amination strategy using 2-nitrobiaryls^13^ and α-azidobiaryls^14^ as the substrate has been developed. Ultimately, most of these reactions require harsh reaction conditions or complex substrates. Therefore, the exploration of very mild and clean conditions for the synthesis of benzo[*a*]carbazoles is still highly desirable. In this context, several successful photoinduced intramolecular cyclizations leading to access benzo[*a*]carbazole manifolds have recently been disclosed. For example, 2-aryl-3-(1-tosylalky)indoles photocyclized to benzo[*a*]carbazoles with irradiated phosphor-coated lamps (Scheme 1a).^15^ Additionally, oxidative photochemical cyclization of ethyl 3-(indol-3-yl)-3-oxo-2-phenylpropanoate derivatives provided 5-hydroxy-benzo[*a*]carbazoles (Scheme 1b).^16^ Recently, aryl indole-based substrates provided access to various benzo[*a*]carbazole ring systems with diverse functional groups via a dehydrogenative 6π-photocyclization protocol under visible-light-mediated conditions (Scheme 1c).^17^ However, given the current limitations and the importance of benzo[*a*]carbazoles, developing environmentally friendly and efficient cyclization reactions to construct such molecular skeletons remains an urgent need. Herein, we report a photochemical cyclization (by optimization of the Mallory cyclization) of 3-styryl indoles in the absence of external oxidants, furnishing numerous 5-aryl- and 5,6-diaryl-11*H*-benzo[*a*]carbazoles (Scheme 1d). Diaryl-ethylene-like molecules have emerged to construct complex polycyclic aromatic compounds from simple molecules and their heterocyclic analogues via the UV-light-mediated dehydrogenative photocyclization reaction.^18^ This powerful tool is termed the Mallory reaction and initiates with the 6π-electronic photocyclization of the substrate and continues with oxidation (formally −H_2_) or elimination. Lately, Zhang et al. have used photoinduced cyclization under irradiation with an Ar atmosphere to construct various complicated fused polyheterocyclics.^19^

## Results and Discussion

For our first attempt, we selected 3-(2,2-diphenylvinyl)-1*H*-indole (**1a**, 0.2 mmol) as the model substrate to investigate the ideal reaction conditions for the formation of 5-phenyl-11*H*-benzo[*a*]carbazole (**2a**) under irradiation (254–420 nm) using a Rayonet photoreactor (Table 1, entries 1–4). To our delight, the corresponding cyclization product **2a** was isolated in 97% yield upon irradiation with light of 350 nm (UV lamp of 30 W) under an Ar atmosphere in MeCN (2 mL) (Table 1, entry 3). Lower yields were observed when the reaction time was shortened (Table 1, entries 5 and 6). Furthermore, the yield of **2a** remained nearly the same with an increasing reaction time (Table 1, entry 7). Replacing MeCN with other solvents, such as DCM, toluene, and THF, led to a decrease in yields (Table 1, entries 8–10). When the MeCN–H_2_O (4:1, v/v) mixture was used for the reaction, a complex mixture formed, which could not be separated (Table 1, entry 11). Using air or oxygen instead of argon produced **2a** with 92% and 60% yields for identical parameters, respectively (Table 1, entries 12 and 13).

**Table 1:**
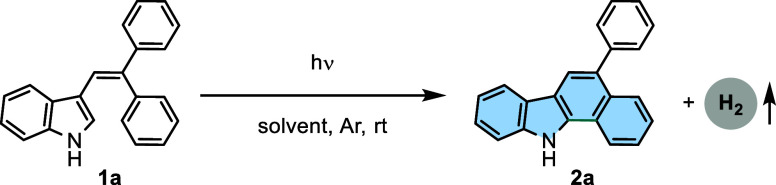
Optimization of the Reaction Conditions^a^

entry	solvent	light (nm)^b^	time (h)	yield of **2a** (%)^c^
1	MeCN	254	6	trace
2	MeCN	300	6	20
3	MeCN	350	6	97
4	MeCN	420	6	trace
5	MeCN	350	2	35
6	MeCN	350	4	80
7	MeCN	350	10	95
8	THF	350	6	94
9	DCM	350	6	82
10	toluene	350	6	85
11	MeCN/H_2_O^d^	350	6	
12	O_2_ instead of Ar, 350 nm, MeCN, 6 h			92
13	air instead of Ar, 350 nm, MeCN, 6 h			60

a Reaction conditions: **1a** (0.2 mmol), solvent (2 mL).

b Photochemistry was carried out at room temperature in solvent (0.1 M) in a Rayonet photoreactor under an argon (Ar) atmosphere.

c Isolated yield.

d MeCN/H_2_O (4:1, v/v), unseparated complex mixture.

**Scheme 2:**
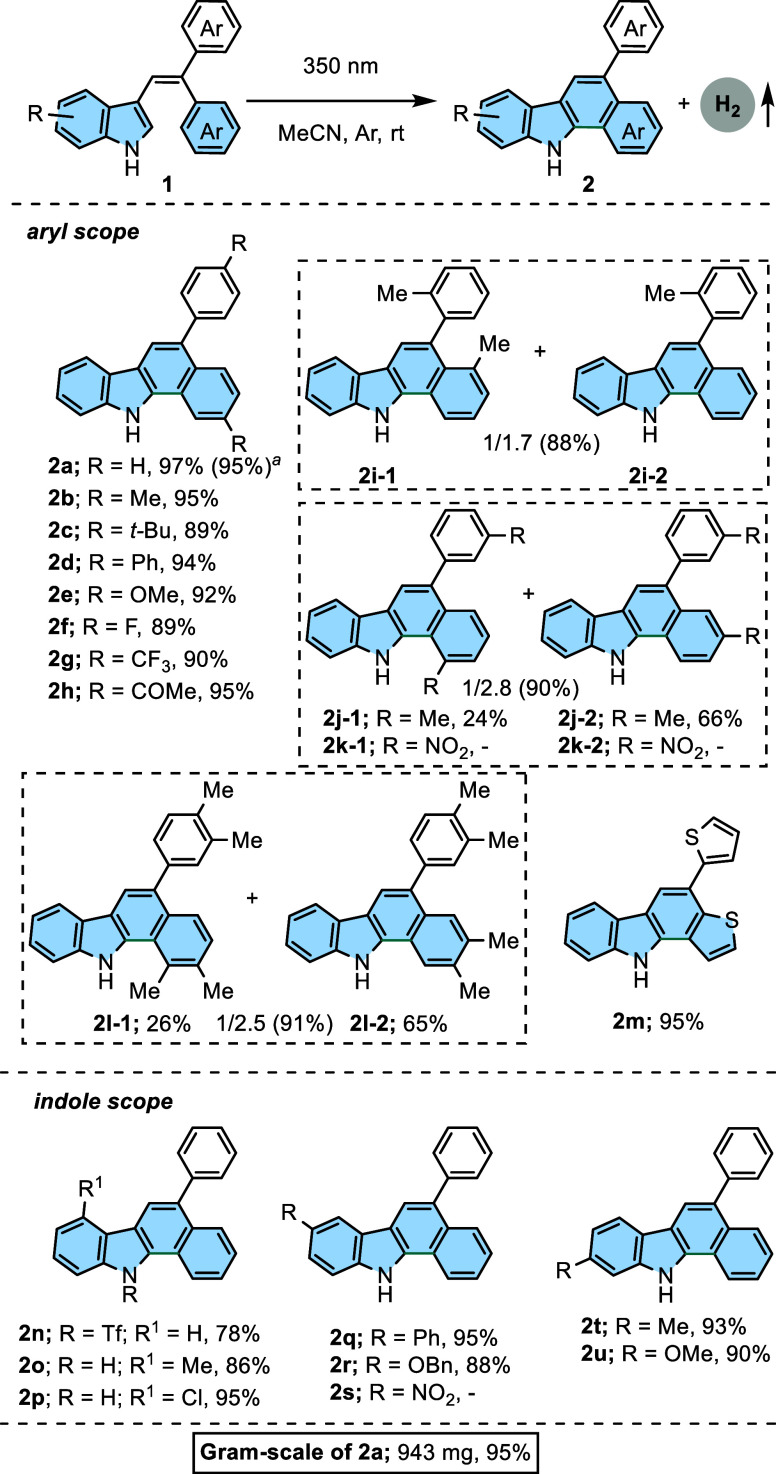
Synthesis of 5-Aryl-11*H*-benzo[*a*]carbazoles

With the optimized conditions, the scope of aryl/indolyl groups in the vinyl unit was examined and is shown in Scheme 2. Irradiation of 3-(2,2-diarylvinyl)-1*H*-indoles **1a–m** with a 350 nm UV lamp gave the corresponding benzo[*a*]carbazoles **2a–m** in 88–97% yields. A diverse array of diaryls, including alkyl, aryl, OMe, COMe, F, and CF_3_ groups, was found to be suitable dehydrogenative intramolecular substrates. Para-substituted phenyl groups bearing electron-donating groups ranging from a strong alkoxy group to weak alkyl and phenyl groups could be smoothly converted into the functionalized benzo[*a*]carbazole molecules in excellent yields (**2b–2e**, 89–95%). Gratifyingly, both a strong electron-withdrawing group such as COMe and inductive electron-withdrawing groups as the F and CF_3_ were well-tolerated in the reaction, giving rise to the desired products (**2f–2h**, 89–95%) in excellent yields. When methyl substituents were attached to the ortho or meta positions of the phenyl ring in ethylene, the reactions also exhibited good performance to construct **2i-1/2** and **2j-1/2** in 88–90% yields. It is important to note that removal of methane gas allows the construction of **2i-2**.^20^ Nevertheless, the photocyclization reaction of substrate **1k** including a strong electron-withdrawing group as a *meta* nitro group failed to deliver the desired products **2k-1** and/or **2k-2**. Substrate **1l** containing methyl groups in meta and para positions was tolerated to construct the product mixture **2l-1** and **2l-2** under the reaction conditions. Noteworthily, the isomeric mixtures of **2j** and **2l** were successfully separated using column chromatography, whereas the separation of a mixture of **2i** failed to deliver the products **2i-1** and **2i-2**. Substrate **1m** including geminal dithiophene was also compatible with this transformation under standard conditions, obtaining the corresponding product **2m** in excellent yields (95%). Subsequently, we extended the scope of application of this method to substituted indoles. For this aim, 1-, 4-, 5-, and 6-substituted indoles (**2n–2s**) were subjected to photocyclization under the optimized reaction conditions. Product **2n** was obtained in a good yield (78%) when the (trifluoromethyl)sulfonyl substitution as a strong electron-withdrawing substituent existed at the 1 position. Besides, 4-Me- and 4-Cl-substituted indoles **1o–1p** furnished target products **2o** and **2p** in excellent yields (86 and 95%). Furthermore, 5- and 6-substituted indole substrates **1q-1r** and **1t-1u** bearing electron-donating (5-Ph, 5-OBn, 6-Me, and 6-OMe) functional groups still proceeded efficiently under this protocol (**2q-2r** and **2t-2u**, 88–95%). However, the 5-nitro-substituted indole substrate **1s** was not tolerated.

**Scheme 3:**
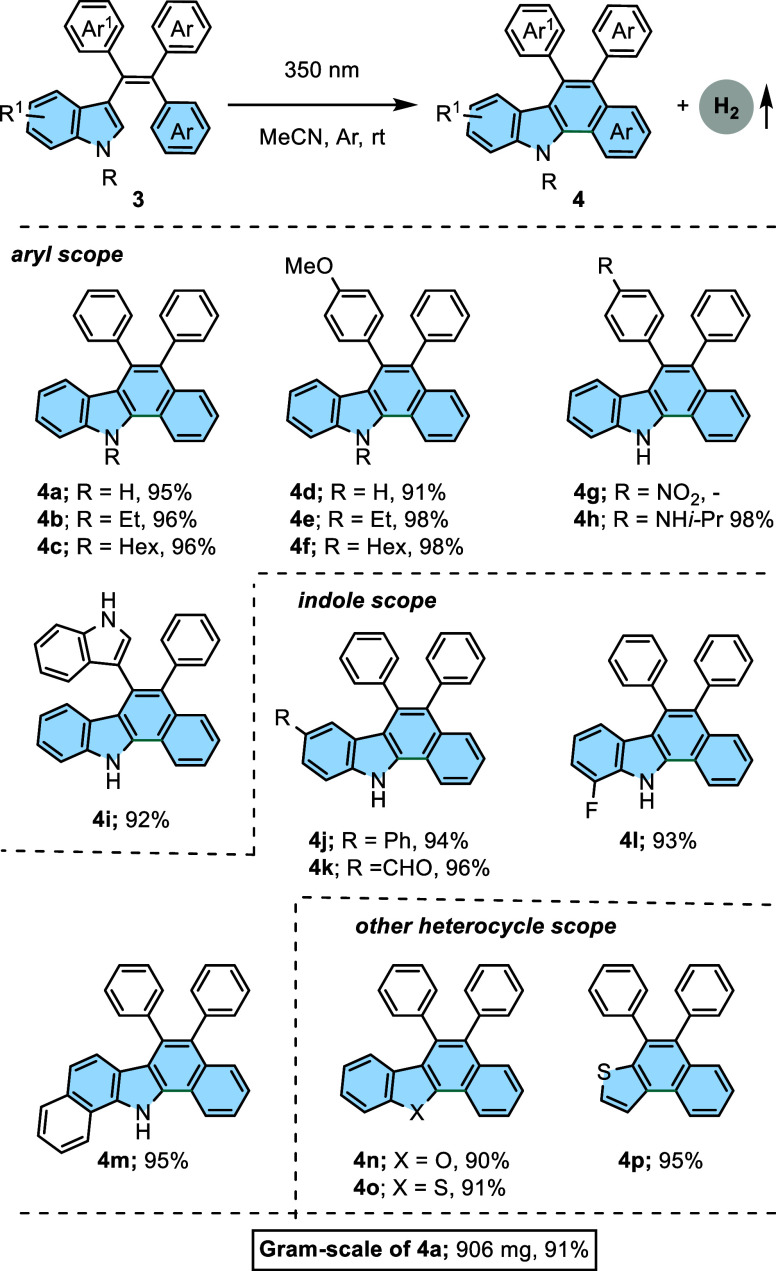
Synthesis of 5,6-Diaryl-11*H*-benzo[*a*]carbazoles

Next, we surveyed the reaction scope concerning the applicability of this strategy to the construction of 5,6-diaryl-11*H*-benzo[*a*]carbazoles **4**. Various 3-(1,2,2-triarylvinyl)-1*H*-indoles **3** were irradiated, providing the corresponding 5,6-diaryl-11*H*-benzo[*a*]carbazoles in 91–98% yields (Scheme 3). We turned our attention to the phenyl and indole moieties in the vinyl part. These rings were modified with electron-donating groups (OMe and NH*i*-Pr) for phenyl and *N*-alkyl, C5–Ph, C5–CHO, and C7–F for indole. This led to the formation of the corresponding products **4a–4i**, achieving excellent yields. However, substrate **3g** did not react at 350 nm. It is noteworthy that substrates **1k**, **1s**, and **3g** with nitro substituents were not unsuitable for the photocyclization process because the nitro group enhances the intersystem crossing pathway from the singlet state to the triplet manifold due to spin–orbital coupling.^21^ Using an indol-3-yl ring instead of a phenyl ring, the reaction could still proceed, yielding the target product **4i** which was obtained with a yield of 92%. According to the ^13^C nuclear magnetic resonance (NMR) spectrum of **4i**, it shows an atropisomer due to the restriction of rotation of phenyl and indolyl rings on the main skeleton. Furthermore, even when the indole ring was replaced by a 1*H*-benzo[*g*]indole ring, product **4m** was obtained with an impressive yield of 95%. We also explored the other heterocycle scopes such as benzofuran, benzothiophene, and thiophene. As a result, the desired products **4n–4p** were obtained with yields from 90% to 95%.

**Scheme 4:**
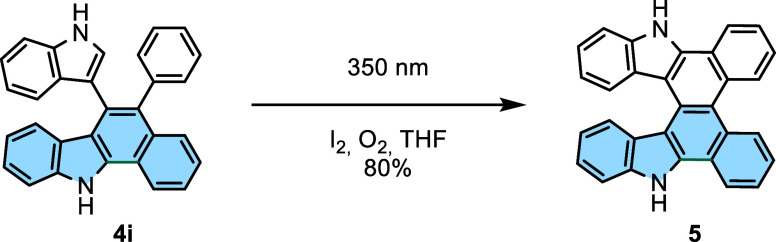
Synthesis of 9,18-Dihydrobenzo[*a*]benzo[1,2]carbazolo[3,4-*c*]carbazole (**5**)

Also, irradiation of **4i** under optimized reaction conditions failed to produce product **5**. Alternatively, photocyclization of **4i** in the presence of oxygen with I_2_ catalysis gave **5** in high yield (Scheme 4). This result suggests that the possible photocyclization intermediate of atropisomer **4i** is presumably reversible under oxidant-free or inert conditions due to its very short lifetime. This experiment revealed that the second dehydrogenative ring-closing reaction efficiently occurred under oxidative conditions and was suppressed under an inert atmosphere, where an oxidant is required to promote the reaction.

**Scheme 5:**
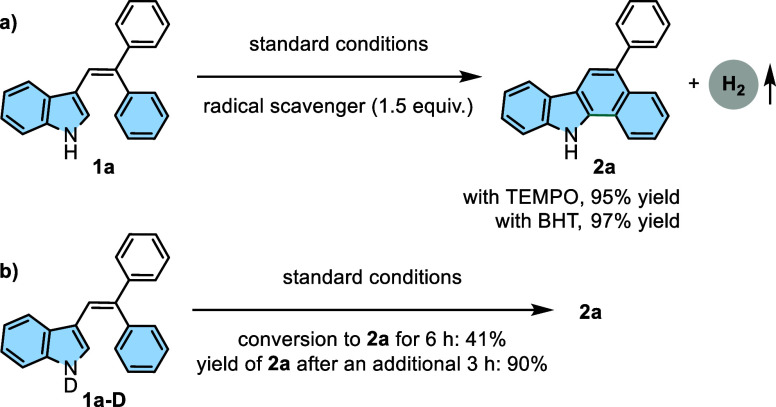
Control Experiments

To further demonstrate the scalability of the process, the gram-scale syntheses of **2a** and **4a** demonstrated excellent results (**2a**, 95%, 943 mg; **4a**, 91%, 906 mg) (Schemes 2 and 3). Free-radical inhibition experiments were conducted to probe the mechanism, as shown in Scheme 5a–e. At the outset, control experiments in the presence of 2,2,6,6-tetramethylpiperidnyl-1-oxide (1.5 equiv) and 2,6-di-*tert*-butyl-4-methylphenol (1.5 equiv) yielded **4a** in 95% and 97% under optimized reaction conditions, respectively (Scheme 5a), indicating that this cyclization process might not proceed via a radical pathway. Moreover, N-deuterated indole **1a-D** was synthesized and subjected to reaction under standard conditions (Scheme 5b). Conversion to **2a** was exclusively obtained in a 41% conversion ratio. The *k*_H_/*k*_D_ value was found to be ∼2.4. This observation can be attributed to the involvement of the N–H bond of the indole ring in the rate-limiting step of the reaction, i.e., hydrogen evolution via tautomerization. Furthermore, the first resulting mixture was exposed for another 3 h to standard conditions. The conversion was completed, and **2a** was isolated in 90% yield.

**Scheme 6:**
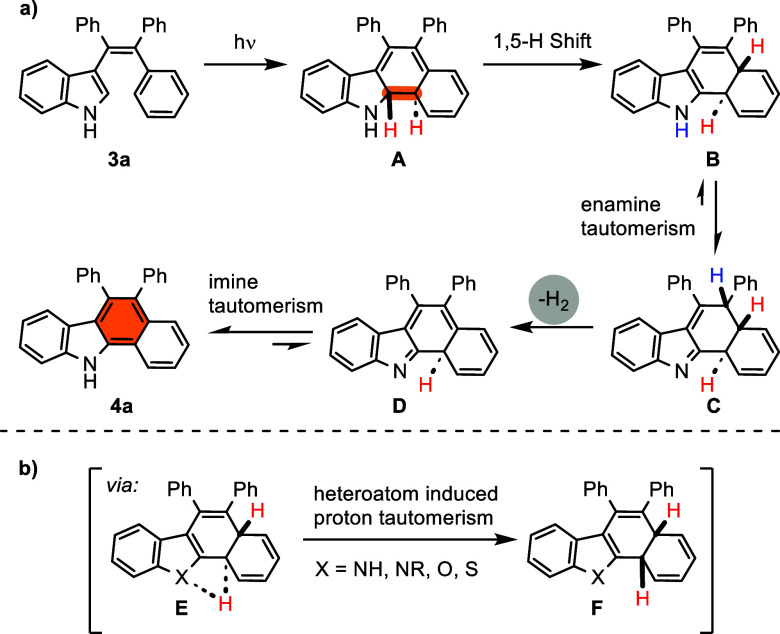
Plausible Reaction Mechanism

Based on controlled experiments and previous mechanistic reports,^18^ a plausible mechanism is proposed in Scheme 6. Irradiation of **3a** in MeCN gives trans-cyclization intermediate **A** via an intramolecular 6π-electrocyclic reaction, followed by a thermal suprafacial [1,5]-*H* shift to form intermediate **B**. Subsequently, enamine tautomerism of **B** leads to the formation of intermediate **C**. Intermediate **C** undergoes the concerted syn-elimination of a hydrogen molecule (H_2_) and gives product **D**. Finally, the aromaticity of the main skeleton is achieved through imine tautomerization (Scheme 6a). Furthermore, the critical step of the mechanism, the shuttle of the hydrogen atom to the opposite surface (from **E** to **F**), can be attributed to the possible proton tautomerism that the heteroatom in the ring may cause (Scheme 6b).^19^

## Conclusions

In summary, an efficient dehydrogenative photocyclization of 3-styryl indoles is disclosed for the first time. This transformation employs irradiation with UV lamps (350 nm) in MeCN at room temperature under an argon atmosphere, affording access to 5-aryl- or 5,6-diaryl-11*H*-benzo[*a*]carbazoles via hydrogen evolution. Prior to this study, to the best of our knowledge, catalyst- and oxidant-free photocyclization to generate 5-aryl- or 5,6-diaryl-11*H*-benzo[*a*]carbazoles has not been documented to date. The developed method possesses a broad substrate scope and a wide functional group tolerance. The gram-scale experiments verified the practicality of the developed methodology. Control experiments guided a concerted mechanism via an intramolecular 6π-electrocyclic reaction, followed by the syn-elimination of a hydrogen molecule. The Mallory-type protocol enables a straightforward method for 11*H*-benzo[*a*]carbazole skeletons, which could have potential biological activity and organic functional material properties.

## Data Availability

The data underlying this study are available in the published article and its Supporting Information.

## Supporting Information Available

Experimental procedures and characterization data for all compounds and NMR and HRMS spectra (PDF)

## References

[ref1] Schmidt A. W.; Reddy K. R.; Knölker H. J. Occurrence, biogenesis, and synthesis of biologically active carbazole alkaloids. Chem. Rev. 2000, 112, 3193–3328. 10.1021/cr200447s. 22480243

[ref2] aRadhakrishna K.; Manjunath S. B.; Devadiga D.; Chetri R.; Nagaraja A. T. Review on carbazole-based hole transporting materials for perovskite solar cell. ACS Appl. Energy Mater. 2023, 6, 3635–3664. 10.1021/acsaem.2c03025.

[ref3] aWang Y. Q.; Li X. H.; He Q.; Chen Y.; Xie Y. Y.; Ding J.; Miao Z.-H.; Yang C. H. Design, synthesis and biological evaluation of substituted 11H-benzo[*a*]carbazole-5-carboxamides as novel antitumor agents. Eur. J. Med. Chem. 2011, 46, 5878–5884. 10.1016/j.ejmech.2011.09.050. 22000922

[ref4] Segall A. I.; Vitale M. F.; Perez V. L.; Pizzorno M. T. HPLC analysis of 5*H*-benzo[*a*]carbazole with antifungal activity. J. Pharm. Biomed. Anal. 2003, 31, 1021–1026. 10.1016/S0731-7085(02)00704-5. 12684115

[ref5] Von Angerer E.; Prekajac J. Benzo[*a*]carbazole derivatives. Synthesis, Estrogen Receptor Binding Affinities, and Mammary Tumor Inhibiting Activity. J. Med. Chem. 1986, 29, 380–386. 10.1021/jm00153a013. 3950918

[ref6] aIvaniuk K.; Cherpak V.; Stakhira P.; Hotra Z.; Minaev B.; Baryshnikov G.; Stromylo E.; Volyniuk D.; Grazulevicius J. V.; Lazauskas A.; et al. Highly Luminous Sky-Blue Organic Light-Emitting Diodes Based on the Bis[(1,2)(5,6)]indoloanthracene Emissive Layer. J. Phys. Chem. C 2016, 120, 6206–6217. 10.1021/acs.jpcc.6b00696.

[ref7] Das S.; Kundu S.; Metya A.; Maji M. S. A toolbox approach to revealing a series of naphthocarbazoles to showcase photocatalytic reductive syntheses. Chem. Sci. 2024, 15, 13466–13474. 10.1039/D4SC03438D. 39183925 PMC11339970

[ref8] Xiao F.; Liao Y.; Wu M.; Deng G.-J. One-pot synthesis of carbazoles from cyclohexanones and arylhydrazine hydrochlorides under metal-free conditions. Green Chem. 2012, 14, 3277–3280. 10.1039/c2gc36473e.

[ref9] Chen C.-C.; Chin L.-Y.; Yang S.-C.; Wu M.-J. Synthetic development and mechanistic study on Pd(II)-catalyzed cyclization of enediynes to benzo[*a*]carbazoles. Org. Lett. 2010, 12, 5652–5655. 10.1021/ol1024458. 21087056

[ref10] Li N.; Lian X. L.; Li Y. H.; Wang T. Y.; Han Z. Y.; Zhang L.; Gong L. Z. Gold-catalyzed direct assembly of aryl-annulated carbazoles from 2-alkynyl arylazides and alkynes. Org. Lett. 2016, 18, 4178–4181. 10.1021/acs.orglett.6b01627. 27529360

[ref11] Tsuchimoto T.; Matsubayashi H.; Kaneko M.; Nagase Y.; Miyamura T.; Shirakawa E. Indium-catalyzed annulation of 2-aryl-and 2-heteroarylindoles with propargyl ethers: Concise synthesis and photophysical properties of diverse aryl-and heteroaryl-annulated[a]carbazoles. J. Am. Chem. Soc. 2008, 130, 15823–15835. 10.1021/ja803954e. 18980318

[ref12] Shen J.; Li N.; Yu Y.; Ma C. Visible-light-induced oxidation/[3 + 2] cycloaddition/oxidative aromatization to construct benzo[*a*]carbazoles from 1,2,3,4-tetrahydronaphthalene and arylhydrazine hydrochlorides. Org. Lett. 2019, 21 (17), 7179–7183. 10.1021/acs.orglett.9b02939. 31456407

[ref13] Gao H.; Xu Q. L.; Yousufuddin M.; Ess D. H.; Kürti L. Rapid Synthesis of Fused *N*-Heterocycles by Transition-Metal-Free Electrophilic Amination of Arene C-H Bonds. Angew. Chem., Int. Ed. 2014, 53, 2701–2705. 10.1002/anie.201309973. 24481643

[ref14] Alt I. T.; Plietker B. Iron-Catalyzed Intramolecular C (sp^2^)– H Amination. Angew. Chem., Int. Ed. 2016, 55, 1519–1522. 10.1002/anie.201510045. 26663257

[ref15] Protti S.; Palmieri A.; Petrini M.; Fagnoni M.; Ballini R.; Albini A. A Photochemical Route to Benzo[*a*]carbazoles *via* Domino Elimination/Electrocyclization of 2-Aryl-3-(1-tosylalkyl) indoles. Adv. Synth. Catal. 2013, 355, 643–646. 10.1002/adsc.201201051.

[ref16] Li Y.; Pang Z.; Zhang T.; Yang J.; Yu W. Oxidative photochemical cyclization of ethyl 3-(indol-3-yl)-3-oxo-2-phenylpropanoate derivatives: synthesis of benzo[*a*]carbazoles. Tetrahedron 2015, 71, 3351–3358. 10.1016/j.tet.2015.03.107.

[ref17] He Y.; Zhang Y.; Zhao H.; Wang X.; He G.; Huang R.; Wei S.; Zhang Z.; Fu Q. Photocatalytic Dehydrogenative 6π-Photocyclisation of Indole Derivatives *via* Successive Energy Transfer. Adv. Synth. Catal. 2024, 366, 508–517. 10.1002/adsc.202301174.

[ref18] aMallory F. B.; Mallory C. W. Photocyclization of stilbenes and related molecules. Org. React. 1984, 30, 1–456. 10.1002/0471264180.or030.01.

[ref19] aKang Y.; He Y.; Sui J.; Wang T.; Liang Y.; Zhang Z. Synthesis of dibenzo[*e,g*]isoindol-1-ones *via* photoinduced intramolecular annulation of 3,4-diphenyl-1*H*-pyrrol-2(5*H*)-ones. Tetrahedron 2021, 84, 131981 10.1016/j.tet.2021.131981.

[ref20] aLvov A. G.; Shirinian V. Z.; Zakharov A. V.; Krayushkin M. M.; Kachala V. V.; Zavarzin I. V. General Photoinduced sequential electrocyclization/[1,9]-sigmatropic rearrangement/ring-opening reaction of diarylethenes. J. Org. Chem. 2015, 80, 11491–11500. 10.1021/acs.joc.5b02237. 26524463

[ref21] aRomero I. E.; Lantaño B.; Postigo A.; Bonesi S. M. Photoinduced [6π]-Electrocyclic Reaction of Mono-Di-and Trisubstituted Triphenylamines in Acetonitrile. A Steady-State Investigation. J. Org. Chem. 2022, 87, 13439–13454. 10.1021/acs.joc.2c00756. 35675160

