# Supplementary material for: Dehydrogenative Photocyclization of 3-Styryl Indoles to Fused Indole Systems
Source: J Org Chem. 2024 Nov 7;89(23):17447–52. doi: 10.1021/acs.joc.4c02103 (PMC11629392; doi:10.1021/acs.joc.4c02103)

Supporting Information

Dehydrogenative-photocyclization of 3-styryl indoles to fused indole systems

Yunus Taskesenligil,[a] and Nurullah Saracoglu[a,b]*

[a]Department of Chemistry, Faculty of Sciences, Atatürk University, 25240, Erzurum, Türkiye

[b]Biotechnology Institute, Ankara University, 06135, Ankara, Türkiye

E-mail: saracoglu@ankara.edu.tr

Contents

**1. General Information**	**S3**
**2. Experimental Section**	**S3-S29**
**3. References**	**S-30-S30**
**4. NMR Spectrum**	**S31-S145**
**5. Mass Spectrum**	**S146-S179**

1. General Information

The solvents and reagents were sourced from commercial suppliers. Tetrahydrofuran was used by distillation over sodium metal in the presence of benzophenone. Proton (^{1}H) and proton-decoupled carbon (^{13}C{^{1}H}) NMR spectra were recorded using a Bruker Avance II 400MHz instrument with tetramethylsilane (TMS) as an internal standard in $CDCl_3$ or DMSO-d_6 or Acetone-d_6 at 400 and 101 MHz, respectively. Peak patterns were described using the following abbreviations: bs = broad singlet, s = singlet, d = doublet, t = triplet, q = quartet, hept = heptet, and m = multiplet when appropriate. High-resolution mass spectra (HRMS) were recorded on an Agilent QTOF (Quadrupole time-of-flight) spectrometry device. Thin layer chromatography (TLC) was performed on alumina plates precoated with silica gel (Merck silica gel, 60 F254). Melting points were determined on a Buchi 539 capillary melting apparatus (uncorrected). Column chromatography was performed using silica gel pore size 60 Å, 70-230 mesh (sigma). All photochemical reactions were carried out using a Rayonet RPR-200 photochemical reactor equipped with 16 fluorescent lamps RPR-3500 Å under an atmosphere of argon (Ar) using anhydrous solvents.

2. Experimental Section

General Procedure A: Preparation of *N*-Protected Indole-3-carboxyaldehydes (S9–S16)[1]

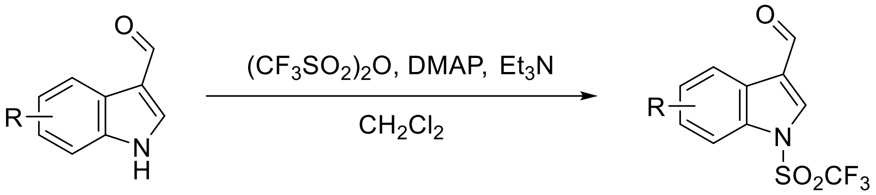

S1–S8 **S9–S16**

Indole-3-carboxyaldehyde derivative (**S1–S18**) (4 mmol, 1eq), Et_3N (2.2 mL, 8 mmol), and DMAP (488 mg, 4 mmol) were dissolved in 40 mL dichloromethane and the solution was cooled to 0 °C. To this mixture was added trifluoromethanesulfonic anhydride (2 mL, 12 mmol) dropwise. The reaction mixture was stirred for 2 hours at the same temperature. After the reaction was quenched with ice water, the mixture was extracted with dichloromethane (2×10 mL). The organic phase was dried over Na_2SO_4 and the solvent was removed under reduced pressure. The residue was purified by column chromatography on a silica gel column (eluent: ethyl acetate/hexane, v/v) to afford the 1-((trifluoromethyl)sulfonyl)-1*H*-indole-3-carbaldehydes (**S9–S16**).

1-((Trifluoromethyl)sulfonyl)-1*H*-indole-3-carbaldehyde (S9)[1]

White solid (720 mg, 65%, m.p: 104–105 °C). Ethyl acetate/hexane (5:95, v/v) as the eluent. **^{1}H NMR (400 MHz, $CDCl_3$) δ** 10.16 (s, CHO, 1H), 8.40 – 8.28 (m, 1H), 8.03 (s, 1H), 7.95 – 7.87 (m, 1H), 7.62 – 7.42 (m, 2H). **^{13}C{^{1}H} NMR (101 MHz, $CDCl_3$) δ** 185.1, 136.2, 136.0, 127.7, 126.6, 126.4, 124.8, 123.2, 119.5 (q, *J* = 323.7 Hz), 113.7.

4-Methyl-1-((trifluoromethyl)sulfonyl)-1*H*-indole-3-carbaldehyde (S10)

White solid (721 mg, 62%, m.p: 58–59 °C). Ethyl acetate/hexane (5:95, v/v) as the eluent. **^{1}H NMR (400 MHz, $CDCl_3$) δ** 10.14 (s, CHO 1H), 7.99 (s, 1H), 7.69 (d, *J* = 7.9 Hz, 1H), 7.42 – 7.25 (t, *J* = 7.9 Hz, 1H), 7.19 (d, *J* = 7.9 Hz, 1H), 2.73 (s, 3H). **^{13}C{^{1}H} NMR (101 MHz, $CDCl_3$) δ** 184.8, 136.8, 135.9, 133.8, 128.2, 127.3, 126.8, 125.8, 119.5 (q, *J* = 323.7 Hz), 11.4, 22.6. **HRMS (ESI-TOF) *m/z*:** [M + H]$^+$ calcd for $C_{11}H_9F_3NO_3S$, 292.0250; found, 292.0242.

4-Chloro-1-((trifluoromethyl)sulfonyl)-1*H*-indole-3-carbaldehyde (S11)

White solid (710 mg, 57%, m.p: 84–85 °C). Ethyl acetate/hexane (5:95, v/v) as the eluent. **^{1}H NMR (400 MHz, $CDCl_3$) δ** 10.79 (s, CHO, 1H), 8.15 (s, 1H), 7.89 (d, *J* = 8.1 Hz, 1H), 7.51 (d, *J* = 8.1 Hz, 1H), 7.43 (t, *J* = 8.1 Hz, 1H). **^{13}C{^{1}H} NMR (101 MHz, $CDCl_3$) δ** 185.8, 137.2, 131.7, 127.6, 127.4, 127.1, 125.6, 124.5, 119.4 (q, *J* = 323.7 Hz). 112.8. **HRMS (ESI-TOF) *m/z*:** $[M + H]^+$ calcd for $C_{10}H_5ClF_3NO_3S$, 311.9704; found, 311.9709.

5-Bromo-1-((trifluoromethyl)sulfonyl)-1*H*-indole-3-carbaldehyde (S12)

White solid (897 mg, 63%, m.p: 123–124 °C). Ethyl acetate/hexane (5:95, v/v) as the eluent. **^{1}H NMR (400 MHz, $CDCl_3$) δ** 10.12 (s, CHO, 1H), 8.51 (d, *J* = 2.0 Hz, 1H), 8.03 (s, 1H), 7.77 (d, *J* = 8.9 Hz, 1H), 7.61 (dd, *J* = 8.9, 2.0 Hz, 1H). **^{13}C{^{1}H} NMR (101 MHz, $CDCl_3$) δ** 184.6, 136.6, 134.8, 130.8, 128.0, 126.1, 123.9, 120.5, 119.4 (q, *J* = 323.7 Hz), 115.0. **HRMS (ESI-TOF) *m/z*:** $[M + H]^+$ calcd for $C_{10}H_6BrF_3NO_3S$, 355.9198; found, 355.9197.

5-(Benzyloxy)-1-((trifluoromethyl)sulfonyl)-1*H*-indole-3-carbaldehyde (S13)

White solid (918 mg, 60%, m.p: 154–155 °C). Ethyl acetate/hexane (5:95, v/v) as the eluent. **^{1}H NMR (400 MHz, $CDCl_3$) δ** 10.12 (s, CHO, 1H), 7.98 (s, 1H), 7.92 (d, *J* = 2.6 Hz, 1H), 7.79 (d, *J* = 9.2 Hz, 1H), 7.49 (d, *J* = 7.2 Hz, 2H), 7.44 – 7.39 (m, 2H), 7.38 – 7.33 (m, 1H), 7.18 (dd, *J* = 9.2, 2.6 Hz, 1H), 5.15 (s, 2H). **^{13}C{^{1}H} NMR (101 MHz, $CDCl_3$) δ** 185.2, 157.8, 136.5, 136.5, 130.5, 128.8, 128.3, 127.8, 127.5, 124.7, 119.4 (q, *J* = 323.8 Hz), 117.7, 114.5, 106.1, 70.7. **HRMS (ESI-TOF) *m/z*:** $[M + H]^+$ calcd for $C_{17}H_{13}F_3NO_4S$, 384.0512; found, 384.0514.

5-Nitro-1-((trifluoromethyl)sulfonyl)-1*H*-indole-3-carbaldehyde (S14)

Yellow solid (888 mg, 69%, m.p: 133–134 °C). Ethyl acetate/hexane (10:90, v/v) as the eluent. **^{1}H NMR (400 MHz, $CDCl_3$) δ** 10.20 (s, CHO 1H), 9.22 (d, *J* = 2.3 Hz, 1H), 8.40 (dd, *J* = 9.3, 2.3 Hz, 1H), 8.23 (s, 1H), 8.05 (d, *J* = 9.3 Hz, 1H). **^{13}C{^{1}H} NMR (101 MHz, $CDCl_3$) δ** 184.2, 146.6, 138.8, 137.9, 126.9, 124.5, 122.9, 119.5, 119.4 (q, *J* = 323.6 Hz), 114.4. **HRMS (ESI-TOF) *m/z*:** $[M + H]^+$ calcd for $C_{10}H_6F_3N_2O_5S$, 322.9944; found, 322.9943.

6-Methyl-1-((trifluoromethyl)sulfonyl)-1*H*-indole-3-carbaldehyde (S15)

White solid (675 mg, 58%, m.p: 117–118 °C). Ethyl acetate/hexane (5:95, v/v) as the eluent. **^{1}H NMR (400 MHz, $CDCl_3$) δ** 10.12 (s, CHO, 1H), 8.20 (d, *J* = 8.1 Hz, 1H), 7.95 (s, 1H), 7.70 (s, 1H), 7.31 (d, *J* = 8.1 Hz, 1H), 2.52 (s, 3H). **^{13}C{^{1}H} NMR (101 MHz, $CDCl_3$) δ** 185.2, 138.4, 136.6, 135.5, 128.0, 124.9, 124.0, 122.7, 119.5 (q, *J* = 323.7 Hz), 113.7, 22.1. **HRMS (ESI-TOF) *m/z*:** $[M + H]^+$ calcd for $C_{11}H_9F_3NO_3S$, 292.0250; found, 292.0247.

6-Methoxy-1-((trifluoromethyl)sulfonyl)-1*H*-indole-3-carbaldehyde (S16)

White solid (749 mg, 61%, m.p: 124–125 °C). Ethyl acetate/hexane (5:95, v/v) as the eluent. **^{1}H NMR (400 MHz, $CDCl_3$) δ** 10.09 (s, 1H), 8.19 (d, *J* = 8.8 Hz, 1H), 7.91 (s, 1H), 7.38 (d, *J* = 2.2 Hz, 1H), 7.08 (dd, *J* = 8.8, 2.3 Hz, 1H), 3.88 (s, 3H). **^{13}C{^{1}H} NMR (101 MHz, $CDCl_3$) δ** 185.1, 160.0, 137.4, 134.8, 124.9, 123.7, 119.8, 119.5 (q, *J* = 323.8 Hz), 115.4, 98.0, 56.0. **HRMS (ESI-TOF) *m/z*:** $[M + H]^+$ calcd for $C_{11}H_8F_3NO_4S$, 308.0199; found, 308.0195.

General Procedure B: Preparation of Corey-Fuchs Products (S17–S24)

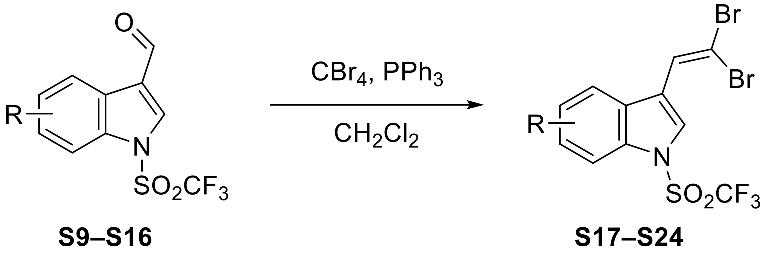

To a solution of indole-3-carbaldehyde derivative (**S9–S16**) (2 mmol) and CBr_4 (994 mg, 3 mmol) in dichloromethane (20 mL) at 0 °C was added dropwise a solution of PPh_3 (1.57 g, 6 mmol) in CH_2Cl_2 (5 mL). The mixture was stirred for 6 h at room temperature. The reaction progress was monitored by thin-layer chromatography (TLC). Then, the organic phase was washed with water (2×10 mL), dried over Na_2SO_4, and the solvent was removed under reduced pressure. The crude product was purified on silica gel column chromatography (eluent: ethyl acetate/hexane, v/v) to afford Corey-Fuchs products (**S17–S24**).

3-(2,2-Dibromovinyl)-1-((trifluoromethyl)sulfonyl)-1*H*-indole (S17)

Light brown solid (813 mg, 94%, m.p: 61–62 °C). Ethyl acetate/hexane (10:90, v/v) as the eluent. **^{1}H NMR (400 MHz, $CDCl_3$) δ** 8.05 (s, 1H), 7.92 (dd, *J* = 7.6, 1.4 Hz, 1H), 7.69 – 7.59 (m, 1H), 7.57 (s, 1H), 7.53 – 7.39 (m, 2H). **^{13}C{^{1}H} NMR (101 MHz, $CDCl_3$) δ** 134.8, 129.6, 126.8, 126.0, 125.3, 124.6, 120.0, 119.6 (q, *J* = 324.2 Hz), 119.56, 114.0, 93.2. **HRMS (APCI-TOF) *m/z*:** $[M + H]^+$ calcd for $C_{11}H_7Br_2F_3NO_2S$, 431.8511; found, 431.8509.

3-(2,2-Dibromovinyl)-4-methyl-1-((trifluoromethyl)sulfonyl)-1*H*-indole (S18)

Yellow oil (795 mg, 89%). Ethyl acetate/hexane (10:90, v/v) as the eluent. **^{1}H NMR (400 MHz, $CDCl_3$) δ** 7.78 (d, *J* = 7.7 Hz, 1H), 7.71 (s, 1H), 7.67 (d, *J* = 1.3 Hz, 1H), 7.31 (t, *J* = 7.7 Hz, 1H), 7.15 (d, *J* = 7.7 Hz, 1H), 2.64 (s, 3H). **^{13}C{^{1}H} NMR (101 MHz, $CDCl_3$) δ** 135.4, 132.1, 129.2, 127.9, 127.2, 126.4, 125.1, 121.3, 119.6 (q, *J* = 324.3 Hz). 111.8, 93.6, 20.3. **HRMS (APCI-TOF) *m/z*:** $[M + H]^+$ calcd for $C_{12}H_9Br_2F_3NO_2S$, 445.8667; found, 445.8658.

4-Chloro-3-(2,2-dibromovinyl)-1-((trifluoromethyl)sulfonyl)-1*H*-indole (S19)

Yellow solid (859 mg, 92%, m.p: 83–84 °C). Ethyl acetate/hexane (10:90, v/v) as the eluent. **^{1}H NMR (400 MHz, $CDCl_3$) δ** 8.04 (d, *J* = 1.3 Hz, 1H), 7.91 (s, 1H), 7.86 (dd, *J* = 7.8, 1.3 Hz, 1H), 7.39 – 7.32 (m, 2H). **^{13}C{^{1}H} NMR (101 MHz, $CDCl_3$) δ** 136.3, 127.9, 127.5, 127.2, 126.8, 126.5, 126.3, 120.2, 119.6 (q, *J* = 324.1 Hz), 112.7, 93.1. **HRMS (APCI-TOF) *m/z*:** $[M]^+$ calcd for $C_{11}H_6Br_2ClF_3NO_2S$, 464.8048; found, 464.8049.

5-Bromo-3-(2,2-dibromovinyl)-1-((trifluoromethyl)sulfonyl)-1*H*-indole (S20)

Light brown solid (972 mg, 95%, m.p: 109–110 °C). Ethyl acetate/hexane (10:90, v/v) as the eluent. **^{1}H NMR (400 MHz, $CDCl_3$) δ** 8.04 (s, 1H), 7.81 – 7.76 (m, 2H), 7.56 (dd, *J* = 8.8, 1.9 Hz, 1H), 7.49 (d, *J* = 0.8 Hz, 1H). **^{13}C{^{1}H} NMR (101 MHz, $CDCl_3$) δ** 133.6, 131.3, 129.9, 125.7, 125.4, 122.6, 119.6 (q, *J* = 324.2 Hz), 119.4, 119.1, 115.5, 94.1. **HRMS (APCI-TOF) *m/z*:** $[M]^+$ calcd for $C_{11}H_5{}^{80}Br_3F_3NO_2S$, 512.7502; found, 512.7483.

5-(Benzyloxy)-3-(2,2-dibromovinyl)-1-((trifluoromethyl)sulfonyl)-1*H*-indole (S21)

White solid (921 mg, 90%, m.p: 137–138 °C). Ethyl acetate/hexane (10:90, v/v) as the eluent. **^{1}H NMR (400 MHz, $CDCl_3$) δ** 8.01 (s, 1H), 7.84 – 7.78 (m, 1H), 7.51 – 7.34 (m, 6H), 7.16 – 7.10 (m, 2H), 5.14 (s, 2H). **^{13}C{^{1}H} NMR (101 MHz, $CDCl_3$) δ** 157.1, 136.7, 130.8, 129.3, 128.9, 128.4, 127.8, 126.1,

125.4, 120.0, 119.7 (q, J = 324.3 Hz), 116.2, 115.0, 103.5, 93.2, 70.9. **HRMS (APCI-TOF) *m/z*:** [M + H]$^+$ calcd for $C_{18}H_{13}Br_2F_3NO_3S$, 537.8930; found, 537.8932.

3-(2,2-Dibromovinyl)-5-nitro-1-((trifluoromethyl)sulfonyl)-1*H*-indole (S22)

Yellow solid (917 mg, 96%, m.p: 118–119 °C). Ethyl acetate/hexane (10:90, v/v) as the eluent. **^{1}H NMR (400 MHz, CDCl$_3$) δ** 8.58 (d, J = 2.2 Hz, 1H), 8.36 (dd, J = 9.2, 2.2 Hz, 1H), 8.21 (s, 1H), 8.06 (d, J = 9.2 Hz, 1H), 7.60 (s, 1H). **^{13}C{^{1}H} NMR (101 MHz, CDCl$_3$) δ** 145.6, 137.7, 129.9, 127.1, 124.7, 121.9, 120.4, 119.4 (d, J = 323.9 Hz), 116.1, 114.6, 95.4. **HRMS (APCI-TOF) *m/z*:** [M]$^+$ calcd for $C_{11}H_5Br_2F_3N_2O_4S$, 475.8289; found, 475.8298.

3-(2,2-Dibromovinyl)-6-methyl-1-((trifluoromethyl)sulfonyl)-1*H*-indole (S23)

Light brown solid (759 mg, 85%, m.p: 84–85 °C). Ethyl acetate/hexane (10:90, v/v) as the eluent. **^{1}H NMR (400 MHz, CDCl$_3$) δ** 7.98 (s, 1H), 7.72 (s, 1H), 7.53 (s, 1H), 7.48 (d, J = 8.1 Hz, 1H), 7.24 (d, J = 8.1 Hz, 1H), 3.03 (s, 3H). **^{13}C{^{1}H} NMR (101 MHz, CDCl$_3$) δ** 137.3, 135.1, 127.3, 126.7, 126.2, 123.9, 119.9, 119.6 (q, J = 324.2 Hz). 119.1, 114.0, 92.9, 22.0. **HRMS (APCI-TOF) *m/z*:** [M + H]$^+$ calcd for $C_{12}H_9Br_2F_3NO_2S$, 445.8667; found, 445.8664.

3-(2,2-Dibromovinyl)-6-methoxy-1-((trifluoromethyl)sulfonyl)-1*H*-indole (S24)

Light brown solid (768 mg, 83%, m.p: 69–70 °C). Ethyl acetate/hexane (10:90, v/v) as the eluent. **^{1}H NMR (400 MHz, CDCl$_3$) δ** 7.92 (s, 1H), 7.51 (s, 1H), 7.47 (d, J = 8.8 Hz, 1H), 7.41 (d, J = 2.2 Hz, 1H), 7.02 (dd, J = 8.8, 2.2 Hz, 1H), 3.88 (s, 3H). **^{13}C{^{1}H} NMR (101 MHz, CDCl$_3$) δ** 159.5, 135.9, 126.2, 123.3, 123.1, 120.1, 120.0, 119.7 (q, J = 324.3 Hz), 114.5, 98.2, 93.0, 56.0. **HRMS (APCI-TOF) *m/z*:** [M]$^+$ calcd for $C_{12}H_8{}^{80}Br_2F_3NO_3S$, 462.8523; found, 462.8497.

General Procedure C: Preparation of Suzuki Coupling Products (1a–1m)

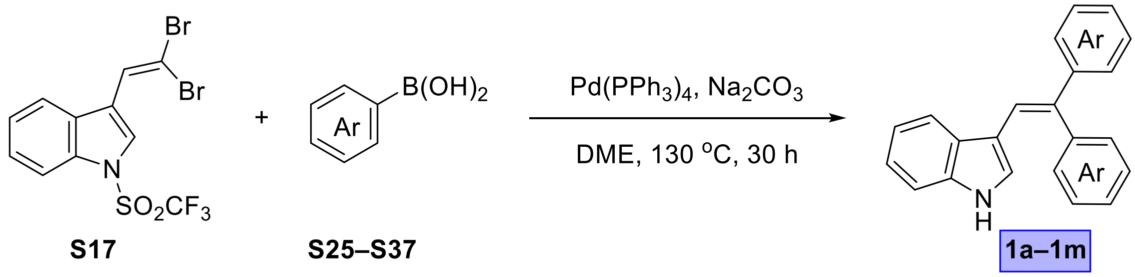

To a solution of 3-(2,2-dibromovinyl)-1-((trifluoromethyl)sulfonyl)-1*H*-indole (**S17**) (217 mg, 0.5 mmol, 1 equiv.) and arylboronic acid (**S25–38**) (1.25 mmol, 2.5 equiv.) in a mixture of dimethoxyethane (DME) (10 mL) and water (10 mL) was added Na_2CO_3 (530 mg, 5 mmol, 10 equiv.). After dissolved gases were removed by bubbling N_2 gas through the solution for about 10 minutes, $Pd(PPh_3)_4$ (23 mg, 0.02 mmol, 0.04 equiv.) was added to the mixture, and the reaction mixture was stirred in an oil bath at 130 °C for 30 hours. To the reaction mixture, cooled to room temperature, aqueous sodium bicarbonate solution was then added. The mixture was then extracted with dichloromethane (3×30 mL). The combined organic layers were dried over anhydrous Na_2SO_4, filtered, and concentrated in vacuo. The residue was purified by column chromatography on silica gel (eluent: ethyl acetate/hexane, v/v) to afford the desired coupling products (**1a–1m**).

3-(2,2-Diphenylvinyl)-1*H*-indole (1a)[13]

Phenylboronic acid (**S25**) (152 mg, 1.25 mmol) was used. White solid (100 mg, 68%, m.p: 148–149 °C). Ethyl acetate/hexane (5:95, v/v) as the eluent. **^{1}H NMR (400 MHz, CDCl$_3$) δ** 7.88 (bs, 1H), 7.78 (dd, J = 7.2, 1.3 Hz, 1H). 7.50 – 7.15 (m, 14H), 6.20 (d, J = 2.6 Hz, 1H). **^{13}C{^{1}H} NMR (101 MHz, CDCl$_3$) δ** 143.0, 142.1, 138.4, 135.2, 130.2, 129.3, 128.4, 127.8, 127.4, 126.9, 126.8, 123.3, 122.6, 120.2, 118.7, 118.6, 114.1, 111.2. **HRMS (ESI-TOF) *m/z*:** [M + H]$^+$ calcd for $C_{22}H_{18}N$, 296.1434; found, 296.1431.

3-(2,2-Diphenylvinyl)-1*H*-indole-1-d (1a–D)

3-(2,2-Diphenylvinyl)-1*H*-indole-1-d (**1a–D**) was prepared by following reported procedure.[14] 3-(2,2-diphenylvinyl)-1*H*-indole (**1a**) (50.3 mg, 0.17 mmol) was dissolved in CD_3OD (1 mL, 24.7 mmol) in a sealed tube stirred in rt for 48 h, the solvent was removed under vacuum to afford quantitative yield of the product **1a–D** with 95% D. **^{1}H NMR (400 MHz, Acetone-*d*$_6$)** δ 7.79 (d, *J* = 7.4 Hz, 1H), 7.52 – 7.47 (m, 2H), 7.46 – 7.39 (m, 4H), 7.38 – 7.28 (m, 5H), 7.26 – 7.18 (m, 1H), 7.16 – 7.04 (m, 2H), 6.23 (s, 1H).

3-(2,2-Di-*p*-tolylvinyl)-1*H*-indole (1b)

p-Tolylboronic acid (**S26**) (170 mg, 1.25 mmol) was used. White solid (170 mg, 70%, m.p: 171–172 ^{o}C). Ethyl acetate/hexane (5:95, v/v) as the eluent. **^{1}H NMR (400 MHz, $CDCl_3$) δ** 7.84 (bs, 1H), 7.78 (dd, *J* = 7.0, 1.4 Hz, 1H), 7.35 – 7.32 (m, AA' part of AA'BB' system, 2H), 7.30 (s, 1H), 7.29 – 7.14 (m, 9H), 6.24 (d, *J* = 2.4 Hz, 1H), 2.46 (s, 3H), 2.39 (s, 3H). **^{13}C{^{1}H} NMR (101 MHz, $CDCl_3$) δ** 140.5, 139.2, 138.3, 136.9, 136.6, 135.2, 130.0, 129.9, 129.1, 127.8, 126.8, 123.1, 122.4, 120.1, 118.7, 117.6, 114.2, 111.1, 21.5, 21.2. **HRMS (ESI-TOF) *m/z*:** $[M + H]^+$ calcd for $C_{24}H_{22}N$, 324.1747; found, 324.1749.

3-(2,2-Bis(4-(*tert*-butyl)phenyl)vinyl)-1*H*-indole (1c)

(4-(*tert*-Butyl)phenyl)boronic acid (**S27**) (222 mg, 1.25 mmol) was used. White solid (128 mg, 63%, m.p: 150–151 ^{o}C). Ethyl acetate/hexane (5:95, v/v) as the eluent. **^{1}H NMR (400 MHz, $CDCl_3$) δ** 7.74 (bs, 1H), 7.62 (d, *J* = 7.7 Hz, 1H), 7.38 – 7.31 (m, AA' part of AA'BB' system, 2H), 7.29 – 7.24 (m, 4H), 7.21 (s, 1H), 7.18 – 7.12 (m, 3H), 7.11 – 7.02 (m, 2H), 6.09 (d, *J* = 2.2 Hz, 1H), 1.29 (s, 9H), 1.24 (s, 9H). **^{13}C{^{1}H} NMR (101 MHz, $CDCl_3$) δ** 150.2, 149.8, 140.4, 139.0, 138.1, 135.3, 129.6, 127.8, 126.4, 126.0, 125.2, 123.1, 122.4, 120.0, 118.8, 117.8, 114.3, 111.1, 34.8, 34.6, 31.7, 31.5. **HRMS (ESI-TOF) *m/z*:** $[M + H]^+$ calcd for $C_{30}H_{34}N$, 408.2686; found, 408.2685.

3-(2,2-Di([1,1'-biphenyl]-4-yl)vinyl)-1*H*-indole (1d)

[1,1'-Biphenyl]-4-ylboronic acid (**S28**) (152 mg, 1.25 mmol) was used. White solid (145 mg, 65%, m.p: 110–111 ^{o}C). Ethyl acetate/hexane (5:95, v/v) as the eluent. **^{1}H NMR (400 MHz, DMSO-*d*$_6$) δ** 11.11 (bs, 1H), 7.84 (d, *J* = 8.1 Hz, 2H), 7.81 – 7.76 (m, 3H), 7.69 – 7.62 (m, 4H), 7.54 – 7.31 (m, 12H), 7.12 (t, *J* = 7.3 Hz, 1H), 7.05 (t, *J* = 7.3 Hz, 1H), 6.25 (d, *J* = 2.4 Hz, 1H). **^{13}C{^{1}H} NMR (101 MHz, DMSO-*d*$_6$) δ** 141.3, 140.8, 139.8, 139.7, 139.0, 138.2, 135.3, 135.2, 130.3, 129.1, 129.0, 127.7, 127.6, 127.4, 127.3, 126.7, 126.6 (2C), 126.5, 124.0, 121.8, 119.4, 119.1, 118.5, 112.4, 111.6. **HRMS (ESI-TOF) *m/z*:** $[M + H]^+$ calcd for $C_{34}H_{26}N$, 448.2060; found, 448.2061.

3-(2,2-Bis(4-methoxyphenyl)vinyl)-1*H*-indole (1e)

(4-Methoxyphenyl)boronic acid (**S29**) (190 mg, 1.25 mmol) was used. White solid (108 mg, 61%, m.p: 168–169 ^{o}C). Ethyl acetate/hexane (5:95, v/v) as the eluent. **^{1}H NMR (400 MHz, DMSO-*d*$_6$) δ** 11.00 (bs, 1H), 7.73 (d, *J* = 7.9 Hz, 1H), 7.34 – 7.27 (m, 3H), 7.25 (s, 1H), 7.17 – 7.12 (m, AA' part of AA'BB' system, 2H), 7.12 – 7.07 (m, 1H), 7.07 – 7.00 (m, 3H), 6.92 – 6.85 (m, BB' part of AA'BB' system, 2H), 6.15 (d, *J* = 2.4 Hz, 1H), 3.81 (s, 3H), 3.74 (s, 3H). **^{13}C{^{1}H} NMR (101 MHz, DMSO-*d*$_6$) δ** 158.5, 158.2, 135.7, 135.3, 135.2, 133.9, 130.7, 127.4, 127.3, 123.3, 121.6, 119.2, 118.4, 116.9, 114.8, 113.7, 112.7, 111.5, 55.1 (2C). **HRMS (ESI-TOF) *m/z*:** $[M + H]^+$ calcd for $C_{24}H_{22}NO_2$, 356.1645; found, 356.1647.

3-(2,2-Bis(4-fluorophenyl)vinyl)-1*H*-indole (1f)

(4-Fluorophenyl)boronic acid boronic acid (**S30**) (237 mg, 1.25 mmol) was used. White solid (133 mg, 62%, m.p: 100–101 ^{o}C). Ethyl acetate/hexane (5:95, v/v) as the eluent. **^{1}H NMR (400 MHz, $CDCl_3$) δ** 7.92 (bs, 1H), 7.73 (d, *J* = 7.8 Hz, 1H), 7.39 – 7.27 (m, 5H), 7.26 (s, 1H), 7.24 – 7.11 (m, 4H), 7.08 –

7.00 (m, 2H), 6.28 (d, *J* = 2.4 Hz, 1H). **$^{13}C\{^1H\}$ NMR (101 MHz, $CDCl_3$) δ** 162.3 (d, *J* = 246.3 Hz), 162.2 (d, *J* = 246.5 Hz), 139.2 (d, *J* = 2.8 Hz), 137.7 (d, *J* = 3.4 Hz), 136.4, 135.3, 131.9 (d, *J* = 7.8 Hz), 128.4 (d, *J* = 7.8 Hz), 127.6, 123.2, 122.7, 120.3, 119.0, 118.8, 116.3 (d, *J* = 21.2 Hz), 115.2 (d, *J* = 21.4 Hz), 113.9, 111.2. **HRMS (ESI-TOF) *m/z*:** $[M + H]^+$ calcd for $C_{22}H_{16}F_2N$, 332.1245; found, 332.1242.

3-(2,2-Bis(4-(trifluoromethyl)phenyl)vinyl)-1*H*-indole (1g)

(4-(Trifluoromethyl)phenyl)boronic acid (**S31**) (175 mg, 1.25 mmol) was used. White solid (99 mg, 60%, m.p: 164–165 °C). Ethyl acetate/hexane (5:95, v/v) as the eluent. **^1H NMR (400 MHz, $CDCl_3$) δ** 7.95 (bs, 1H), 7.70 – 7.63 (m, 3H), 7.56 – 7.51 (m, AA' part of AA'BB' system, 2H), 7.43 – 7.36 (m, 5H), 7.27 (d, *J* = 7.4 Hz, 1H), 7.23 – 7.12 (m, 2H), 6.21 (d, *J* = 2.6 Hz, 1H). **$^{13}C\{^1H\}$ NMR (101 MHz, $CDCl_3$) δ** 145.9, 145.3, 135.5, 135.3, 130.8, 129.9 (q, *J* = 32.3 Hz), 128.9 (q, *J* = 32.4 Hz), 127.5, 127.0, 126.4 (q, *J* = 3.1 Hz), 125.5 (q, *J* = 3.2 Hz), 124.44 (q, *J* = 271.6 Hz), 124.37 (q, *J* = 271.5 Hz), 123.9, 123.0, 121.7, 120.7, 118.7, 113.3, 111.4. **HRMS (ESI-TOF) *m/z*:** $[M + H]^+$ calcd for $C_{24}H_{16}F_6N$, 432.1181; found, 432.1180.

1,1'-((2-(1*H*-Indol-3-yl)ethene-1,1-diyl)bis(4,1-phenylene))bis(ethan-1-one) (1h)

(4(4-Acetylphenyl)boronic acid (**S32**) (205 mg, 1.25 mmol) was used. Yellow solid (131 mg, 69%, m.p: 216–217 °C). Ethyl acetate/hexane (5:95, v/v) as the eluent. **^1H NMR (400 MHz, $CDCl_3$) δ** 8.29 (bs, 1H), 8.08 – 7.99 (m, AA' part of AA'BB' system, 2H), 7.99 – 7.85 (m, AA' part of AA'BB' system, 2H), 7.73 (d, *J* = 7.5 Hz, 1H), 7.49 (s, 1H), 7.47 – 7.40 (m, 4H), 7.37 – 7.27 (m, 1H), 7.25 – 7.16 (m, 2H), 6.28 (d, *J* = 2.6 Hz, 1H), 2.67 (s, 3H), 2.60 (s, 3H). **$^{13}C\{^1H\}$ NMR (101 MHz, $CDCl_3$) δ** 198.2, 197.8, 147.0, 146.9, 136.4, 135.9, 135.39, 135.36, 130.7, 129.5, 128.7, 127.6, 126.7, 124.2, 122.9, 121.8, 120.6, 118.6, 113.4, 111.4, 26.8, 26.7. **HRMS (ESI-TOF) *m/z*:** $[M + H]^+$ calcd for $C_{26}H_{22}NO_2$, 380.1645; found, 380.1647.

3-(2,2-di-*o*-Tolylvinyl)-1*H*-indole (1i)

o-Tolylboronic acid (**S33**) (170 mg, 1.25 mmol) was used. Yellow oil (94 mg, 58%). Ethyl acetate/hexane (5:95, v/v) as the eluent. **^1H NMR (400 MHz, Acetone-*d*$_6$) δ** 10.19 (bs, 1H), 7.71 (d, *J* = 7.5 Hz, 1H), 7.39 – 7.36 (m, 1H), 7.29 – 7.19 (m, 6H), 7.17 – 7.10 (m, 3H), 7.07 (dd, *J* = 7.9, 1.1 Hz, 1H), 7.04 (s, 1H), 6.32 (d, *J* = 2.6 Hz, 1H), 2.42 (s, 3H), 2.14 (s, 3H). **$^{13}C\{^1H\}$ NMR (101 MHz, Acetone-*d*$_6$) δ** 144.1, 143.1, 136.9, 136.8, 136.6, 136.3, 131.8, 131.5, 131.1, 130.5, 128.6, 128.1, 127.3, 127.2, 126.3, 123.94, 123.93, 122.6, 120.3, 119.0, 113.8, 112.2, 21.5, 20.0. **HRMS (ESI-TOF) *m/z*:** $[M + H]^+$ calcd for $C_{24}H_{22}N$, 324.1747; found, 324.1747.

3-(2,2-Di-*m*-tolylvinyl)-1*H*-indole (1j)

m-Tolylboronic acid (**S34**) (170 mg, 1.25 mmol) was used. Light yellow oil (99 mg, 61%). Ethyl acetate/hexane (5:95, v/v) as the eluent. **^1H NMR (400 MHz, Acetone-*d*$_6$) δ** 10.15 (bs, 1H), 7.80 (dd, *J* = 7.7, 0.7 Hz, 1H), 7.43 (s, 1H), 7.40 – 7.34 (m, 2H), 7.29 (s, 1H), 7.24 (dd, *J* = 7.7, 0.7 Hz, 1H), 7.22 – 7.16 (m, 2H), 7.14 – 7.03 (m, 5H), 6.23 (d, *J* = 2.6 Hz, 1H), 2.35 (s, 3H), 2.31 (s, 3H). **$^{13}C\{^1H\}$ NMR (101 MHz, Acetone-*d*$_6$) δ** 143.8, 143.1, 139.6, 138.3, 138.1, 136.5, 131.1, 130.0, 128.9, 128.8, 128.7, 128.0, 127.8, 127.7, 124.6 (2C), 122.6, 120.3, 119.2, 119.1, 114.0, 112.2, 21.6, 21.5. **HRMS (ESI-TOF) *m/z*:** $[M + H]^+$ calcd for $C_{24}H_{22}N$, 324.1747; found, 324.1747.

3-(2,2-Bis(3-nitrophenyl)vinyl)-1*H*-indole (1k)

(3-Nitrophenyl)boronic acid (**S35**) (109 mg, 1.25 mmol) was used. Orange solid (114 mg, 59%, m.p: 215–216 °C). Ethyl acetate/hexane (5:95, v/v) as the eluent. **^1H NMR (400 MHz, Acetone-*d*$_6$) δ** 10.38 (bs, 1H), 8.38 – 8.32 (m, 1H), 8.24 (d, *J* = 0.9 Hz, 1H), 8.18 (t, *J* = 2.0 Hz, 1H), 8.13 (dd, *J* = 8.0, 1.8

Hz, 1H), 7.87 (d, *J* = 8.0 Hz, 1H), 7.85 – 7.82 (m, 2H), 7.76 (d, *J* = 8.0 Hz, 1H), 7.73 (s, 1H), 7.64 (t, *J* = 8.0 Hz, 1H), 7.39 (d, *J* = 8.0 Hz, 1H), 7.18 – 7.13 (m, 1H), 7.12 – 7.07 (m, 1H), 6.51 (d, *J* = 2.6 Hz, 1H). **$^{13}C\{^1H\}$ NMR (101 MHz, Acetone-*d*$_6$) δ** 150.3, 149.6, 144.9, 143.6, 137.9, 136.8, 133.4, 133.3, 132.0, 130.6, 128.4, 126.1, 126.0, 124.4, 123.6, 123.2, 122.2, 121.7, 120.8, 119.4, 113.1, 112.5. **HRMS (ESI-TOF) *m/z*:** $[M + H]^+$ calcd for $C_{22}H_{16}N_3O_4$, 386.1135; found, 386.1129.

3-(2,2-Bis(3,4-dimethylphenyl)vinyl)-1*H*-indole (1l)

(3,4-Dimethylphenyl)boronic acid (**S36**) (188 mg, 1.25 mmol) was used. Light yellow solid (105 mg, 60%, m.p: 135–136 °C). Ethyl acetate/hexane (5:95, v/v) as the eluent. **^1H NMR (400 MHz, Acetone-*d*$_6$) δ** 10.09 (bs, 1H), 7.78 (d, *J* = 7.7 Hz, 1H), 7.38 – 7.31 (m, 2H), 7.26 – 7.21 (m, 2H), 7.14 – 7.03 (m, 5H), 7.00 (dd, *J* = 7.7, 1.6 Hz, 1H), 6.27 (d, *J* = 2.5 Hz, 1H), 2.33 (s, 3H), 2.26 (s, 3H), 2.23 (s, 3H), 2.23 (s, 3H). **$^{13}C\{^1H\}$ NMR (101 MHz, Acetone-*d*$_6$) δ** 141.8, 140.7, 138.2, 138.0, 136.7, 136.5, 136.0, 135.4, 131.6, 131.1, 130.2, 128.8, 128.4, 128.0, 125.0, 124.3, 122.5, 120.6, 119.1, 118.2, 114.1, 112.1, 20.0, 19.8, 19.7, 19.4. **HRMS (ESI-TOF) *m/z*:** $[M + H]^+$ calcd for $C_{26}H_{26}N$, 352.2060; found, 352.2060.

3-(2,2-Di(thiophen-2-yl)vinyl)-1*H*-indole (1m)

Thiophene-2-ylboronic acid (**S37**) (160 mg, 1.25 mmol) was used. Yellow solid (115 mg, 75%, m.p: 157–158 °C). Ethyl acetate/hexane (5:95, v/v) as the eluent. **^1H NMR (400 MHz, $CDCl_3$) δ** 8.04 (bs, 1H), 7.86 – 7.64 (m, 1H), 7.50 – 7.39 (m, 2H), 7.31 (dd, *J* = 6.7, 2.0 Hz, 1H), 7.25 – 7.14 (m, 4H), 7.08 (dd, *J* = 3.5, 0.9 Hz, 1H), 6.98 (dd, *J* = 5.1, 3.7 Hz, 1H), 6.95 – 6.76 (m, 1H), 6.36 (d, *J* = 2.6 Hz, 1H). **$^{13}C\{^1H\}$ NMR (101 MHz, $CDCl_3$) δ** 148.1, 141.4, 135.3, 127.7, 127.6, 127.5, 127.4, 126.5, 124.6, 124.4, 123.9, 123.7, 122.7, 120.7, 120.4, 118.6, 113.2, 111.2. **HRMS (ESI-TOF) *m/z*:** $[M + H]^+$ calcd for $C_{18}H_{14}NS_2$, 308.0562; found, 308.0563.

3-(2,2-Diphenylvinyl)-1-((trifluoromethyl)sulfonyl)-1*H*-indole (1n)

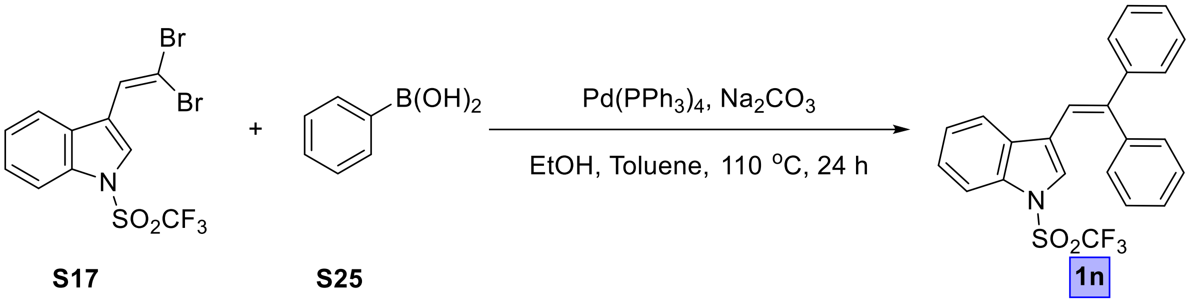

S17 **S25** **1n**

To a solution of 3-(2,2-dibromovinyl)-1-((trifluoromethyl)sulfonyl)-1*H*-indole (**S17**) (0.6 mmol, 1 equiv.) and phenylboronic acid (**S25**) (183 mg, 1.5 mmol, 2.5 equiv.) in a mixture of ethanol (6 mL) and water (6 mL) was added Na_2CO_3 (636 mg, 6 mmol, 10 equiv.). After dissolved gases were removed by bubbling N_2 gas through the solution for about 10 minutes, $Pd(PPh_3)_4$ (23 mg, 0.02 mmol, 0.04 equiv.) was added to the mixture, and the reaction mixture was stirred in an oil bath at 130 °C for 30 hours. To the reaction mixture, cooled to room temperature, aqueous sodium bicarbonate solution was then added. The mixture was then extracted with dichloromethane (3×30 mL). The combined organic layers were dried over anhydrous Na_2SO_4, filtered, and concentrated in vacuo. The residue was purified by column chromatography on silica gel (eluent: ethyl acetate/hexane, 1:99, v/v) to afford the coupling product **1n**. White solid (192 mg, 75%, m.p: 93–94 °C). **^1H NMR (400 MHz, $CDCl_3$)** δ 7.88 – 7.78 (m, =CH, 1H), 7.74 – 7.64 (m, =CH, 1H), 7.54 – 7.28 (m, =CH, 10H), 7.27 – 7.20 (m, =CH, 2H), 7.07 (s, =CH, 1H), 6.27 (s, 1H). **$^{13}C\{^1H\}$ NMR (101 MHz, $CDCl_3$) δ** 145.8, 141.8, 140.3, 135.0, 131.1, 129.4, 129.4, 128.6, 128.3, 128.2, 127.4, 126.1, 124.9, 123.7, 121.6, 119.9, 119.6 (q, *J* = 324.4 Hz), 115.2, 113.8. **HRMS (APCI-TOF) *m/z*:** $[M + H]^+$ calcd for $C_{23}H_{17}F_3NO_2S$, 428.0927; found, 428.0949.

General Procedure D: Preparation of Suzuki Coupling Products (1o–1u)

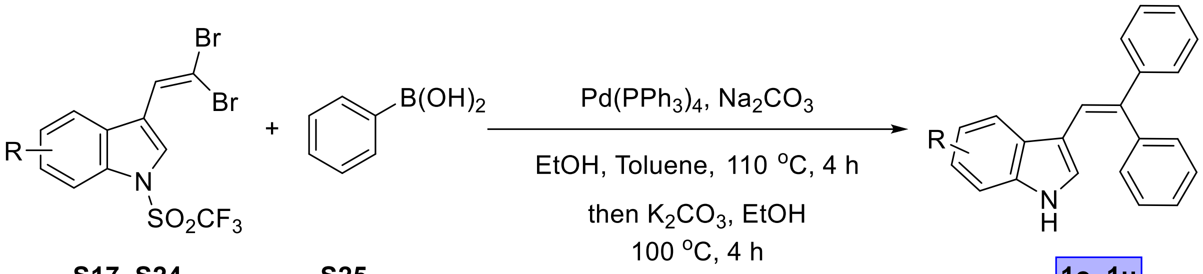

S17–S24 **S25** **1o–1u**

To a solution of 3-(2,2-dibromovinyl)-1-((trifluoromethyl)sulfonyl)-1*H*-indole derivative (**S17–S24**) (0.6 mmol, 1 equiv.) and phenylboronic acid (**S25**) (183 mg, 1.5 mmol, 2.5 equiv.) in a mixture of ethanol (6 mL) and water (6 mL) was added Na_2CO_3 (636 mg, 6 mmol, 10 equiv.). After dissolved gases were removed by bubbling N_2 gas through the solution for about 10 minutes, $Pd(PPh_3)_4$ (23 mg, 0.02 mmol, 0.04 equiv.) was added to the mixture, and the reaction mixture was stirred in an oil bath at 130 °C for 30 hours. To the reaction mixture, cooled to room temperature, aqueous sodium bicarbonate solution was then added. The mixture was then extracted with dichloromethane (3×30 mL). The combined organic layers were dried over anhydrous Na_2SO_4, filtered, and concentrated in vacuo. Then, the residue was dissolved in MeOH (20 mL), and K_2CO_3 (414 mg, 3 mmol, 5 eV) was added to this solution and then the mixture was boiled at 90 °C for 5 hours. The solvent was removed under reduced pressure, the mixture was extracted with ethyl acetate (3×15 mL), and the organic layer was dried over Na_2SO_4, and concentrated under reduced pressure. The residue was purified by column chromatography on silica gel (eluent: ethyl acetate/hexane, v/v) to afford the desired coupling products (**1o–1u**).

3-(2,2-Diphenylvinyl)-4-methyl-1*H*-indole (1o)

Off-white solid (89 mg, 48%, m.p: 128–129 °C). Ethyl acetate/hexane (5:95, v/v) as the eluent**.** **^{1}H NMR (400 MHz, Acetone-*d*$_6$) δ** 10.10 (bs, 1H), 7.69 (s, 1H), 7.46 – 7.16 (m, 11H), 6.98 (t, *J* = 7.6 Hz, 1H), 6.83 (d, *J* = 7.1 Hz, 1H), 6.34 (d, *J* = 2.4 Hz, 1H), 2.80 (s, 3H). **^{13}C{^{1}H} NMR (101 MHz, Acetone-*d*$_6$) δ** 144.3, 142.9, 138.4, 137.0, 131.2, 129.9 (2C), 129.2, 127.9, 127.4 (2C), 125.4, 122.8, 122.6, 122.5, 115.4, 110.5, 110.4, 21.7. **HRMS (ESI-TOF) *m/z*:** $[M + H]^+$ calcd for $C_{23}H_{20}N$, 310.1590; found, 310.1594.

4-Chloro-3-(2,2-diphenylvinyl)-1*H*-indole (1p)

Off-white solid (110 mg, 56%, m.p: 125–126 °C). Ethyl acetate/hexane (5:95, v/v) as the eluent. **^{1}H NMR (400 MHz, Acetone-*d*$_6$) δ** 10.43 (bs, 1H), 8.09 (d, *J* = 0.8 Hz, 1H), 7.50 – 7.43 (m, 2H), 7.42 – 7.26 (m, 8H), 7.26 – 7.20 (m, 1H), 7.12 – 7.05 (m, 2H), 6.38 (dd, *J* = 2.8, 0.8 Hz, 1H). **^{13}C{^{1}H} NMR (101 MHz, Acetone-*d*$_6$) δ** 144.1, 142.8, 138.6, 138.3, 131.0, 130.1, 129.2, 128.1, 127.6, 127.4, 126.7, 126.6, 124.9, 123.2, 121.9, 121.2, 114.4, 111.7. **HRMS (ESI-TOF) *m/z*:** $[M + H]^+$ calcd for $C_{22}H_{17}ClN$, 330.1044; found, 330.1043.

3-(2,2-Diphenylvinyl)-5-phenyl-1*H*-indole (1q)

Off-white solid (122 mg, 55%, m.p: 151–152 °C). Ethyl acetate/hexane (5:95, v/v) as the eluent. **^{1}H NMR (400 MHz, Acetone-*d*$_6$) δ** 10.25 (bs, 1H), 8.05 (d, *J* = 0.6 Hz, 1H), 7.73 – 7.67 (m, 2H), 7.57 (s,

1H), 7.53 – 7.40 (m, 9H), 7.35 – 7.27 (m, 5H), 7.26 – 7.20 (m, 1H), 6.34 (dd, *J* = 2.1, 0.6 Hz, 1H). **$^{13}C\{^1H\}$ NMR (101 MHz, Acetone-d_6) δ** 143.9, 143.4, 143.2, 138.2, 136.3, 133.8, 130.9, 130.2, 129.5, 129.2, 129.1, 128.2, 128.0, 127.4, 127.4, 127.1, 125.7, 122.3, 119.8, 117.9, 114.6, 112.7. **HRMS (ESI-TOF) *m/z*:** $[M + H]^+$ calcd for $C_{28}H_{22}N$, 372.1747; found, 372.1733.

5-(Benzyloxy)-3-(2,2-diphenylvinyl)-1*H*-indole (1r)

White solid (130 mg, 50%, m.p: 172–173 °C). Ethyl acetate/hexane (5:95, v/v) as the eluent. **^1H NMR (400 MHz, Acetone-d_6) δ** 10.08 (bs, 1H), 7.56 – 7.45 (m, 4H), 7.44 – 7.18 (m, 14H), 6.87 (dd, *J* = 8.7, 2.4 Hz, 1H), 6.30 (d, *J* = 2.6 Hz, 1H), 5.11 (s, 2H). **$^{13}C\{^1H\}$ NMR (101 MHz, Acetone) δ** 154.5, 144.1, 143.3, 139.2, 137.6, 132.0, 130.9, 130.1, 129.2, 129.1, 128.5, 128.4, 128.1, 127.4, 127.3, 125.8, 125.6, 120.2, 114.1, 113.7, 113.0, 102.9, 71.2, 114.6, 112.7. **HRMS (ESI-TOF) *m/z*:** $[M + H]^+$ calcd for $C_{29}H_{24}NO$, 402.1852; found, 402.1831.

3-(2,2-Diphenylvinyl)-5-nitro-1*H*-indole (1s)

Orange solid (102 mg, 50%, m.p: 195–196 °C). Ethyl acetate/hexane (5:95, v/v) as the eluent. **^1H NMR (400 MHz, Acetone-d_6) δ** 10.79 (bs, 1H), 8.71 (s, 1H), 8.03 (d, *J* = 8.9 Hz, 1H), 7.55 – 7.40 (m, 7H), 7.37 – 7.24 (m, 5H), 6.48 (s, 1H). **$^{13}C\{^1H\}$ NMR (101 MHz, Acetone-d_6) δ** 143.3, 142.7, 142.6, 140.5, 139.6, 130.6, 130.2, 129.2, 128.4, 128.1, 127.9, 127.6, 118.4, 118.0, 116.9 (2C), 116.6, 112.7. **HRMS (ESI-TOF) *m/z*:** $[M + H]^+$ calcd for $C_{22}H_{17}N_2O_2$, 341.1285; found, 341.1278.

3-(2,2-Diphenylvinyl)-6-methyl-1*H*-indole (1t)

Brown oil (81 mg, 50%). Ethyl acetate/hexane (5:95, v/v) as the eluent. **^1H NMR (400 MHz, Acetone-d_6)** δ 9.99 (bs, 1H), 7.65 (d, *J* = 8.1 Hz, 1H), 7.51 – 7.40 (m, 6H), 7.35 – 7.27 (m, 4H), 7.24 – 7.20 (m, 1H), 7.16 (dd, *J* = 1.5, 0.7 Hz, 1H), 6.96 – 6.91 (m, 1H), 6.19 (d, *J* = 2.1, 0.5 Hz, 1H), 2.40 (s, 3H). **$^{13}C\{^1H\}$ NMR (101 MHz, Acetone-d_6) δ** 143.8, 143.1, 137.7, 137.0, 132.1, 130.7, 130.1, 129.0, 128.9, 128.0, 127.22, 127.20, 124.1, 122.1, 119.9, 118.9, 113.9, 112.1, 21.7. **HRMS (ESI-TOF) *m/z*:** $[M + H]^+$ calcd for $C_{23}H_{20}N$, 310.1590; found, 310.1590.

3-(2,2-Diphenylvinyl)-6-methoxy-1*H*-indole (1u)

Off-white solid (82 mg, 42%, m.p: 126–127 °C). Ethyl acetate/hexane (5:95, v/v) as the eluent. **^1H NMR (400 MHz, Acetone-d_6) δ** 9.95 (bs, 1H), 7.62 (d, *J* = 8.7 Hz, 1H), 7.51 – 7.45 (m, 2H), 7.44 – 7.38 (m, 4H), 7.36 – 7.26 (m, 4H), 7.22 (t, *J* = 7.2 Hz, 1H), 6.89 (d, *J* = 2.2 Hz, 1H), 6.75 (dd, *J* = 8.7, 2.3 Hz, 1H), 6.15 (d, *J* = 2.6 Hz, 1H), 3.77 (s, 3H). **$^{13}C\{^1H\}$ NMR (101 MHz, Acetone-d_6) δ** 157.6, 143.9, 143.2, 137.9, 137.4, 130.8, 130.1, 129.1, 128.1, 127.29, 127.28, 123.6, 123.0, 119.93, 119.90, 114.0, 110.6, 95.3, 55.7. **HRMS (ESI-TOF) *m/z*:** $[M + H]^+$ calcd for $C_{23}H_{20}NO$, 326.1539; found, 326.1530.

General Procedure E: Preparation of *N*-Tosylindole Derivatives (S43–S47)[2]

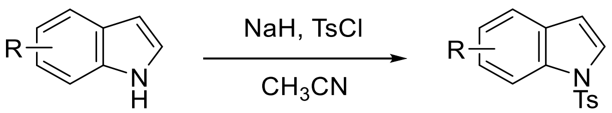

S38–S42 **S43–S47**

A solution of indole derivative (**S39–S43**) (1.17 g, 10 mmol) in 20 mL of CH_3CN was cooled to 0 °C, and NaH (60%; 479 mg, 12 mmol) was added. After stirring at the same temperature for 30 min, *p*-toluenesulfonyl chloride (1.92 g, 10.1 mmol) was slowly added to the mixture. Then, the reaction mixture was allowed to stir at room temperature for 12 h. Upon reaction completion, the reaction was quenched with saturated NH_4Cl solution. The mixture was extracted with dichloromethane (3×40 mL). The organic phase was dried over Na_2SO_4 and the solvent was removed under reduced pressure. The residue was purified by column chromatography on a silica gel column (eluent: ethyl acetate/hexane, v/v) to afford the *N*-tosylindole derivatives (**S44–S48**).

1-Tosyl-1*H*-indole (S43)[2]

White solid (2.68 g, 99%, m.p: 82–83 °C). Ethyl acetate/hexane (10:90, v/v) as the eluent. **^{1}H NMR (400 MHz, $CDCl_3$) δ** 7.99 (d, *J* = 8.3 Hz, 1H), 7.80 – 7.72 (m, AA' part of AA'BB' system, 2H), 7.56 (d, *J* = 3.6 Hz, 1H), 7.52 (d, *J* = 7.9 Hz, 1H), 7.34 – 7.27 (m, 1H), 7.25 – 7.17 (m, 3H), 6.65 (d, *J* = 3.6 Hz, 1H), 2.33 (s, 3H). **^{13}C{^{1}H} NMR (101 MHz, $CDCl_3$) δ** 145.0, 135.4, 134.9, 130.9, 130.0, 126.9, 126.4, 124.6, 123.4, 121.5, 113.6, 109.1, 21.6.

5-Phenyl-1-tosyl-1H-indole (S44)[3]

White solid (3.40 g, 98%, m.p: 158–159 °C). Ethyl acetate/hexane (10:90, v/v) as the eluent. **^{1}H NMR (400 MHz, $CDCl_3$) δ** 8.06 (d, *J* = 8.6 Hz, 1H), 7.84 – 7.78 (m, AA' part of AA'BB' system, 2H), 7.73 (d, *J* = 1.5 Hz, 1H), 7.64 – 7.57 (m, 3H), 7.56 (dd, *J* = 8.6, 1.5 Hz, B part of AB system, 1H), 7.44 (t, *J* = 7.4 Hz, 2H), 7.34 (t, *J* = 7.4 Hz, 1H), 7.25 – 7.19 (m, AA' part of AA'BB' system, 2H), 6.71 (d, *J* = 3.6 Hz, 1H), 2.34 (s, 3H). **^{13}C{^{1}H} NMR (101 MHz, $CDCl_3$) δ** 145.1, 141.4, 136.9, 135.5, 134.4, 131.4, 130.0, 128.9, 127.5, 127.2, 127.1, 127.0, 124.3, 119.9, 113.9, 109.4, 21.7.

1-Tosyl-1*H*-indole-5-carbaldehyde (S45)[4]

Light brown solid (2.93 g, 98%, m.p: 123–124 °C). Ethyl acetate/hexane (10:90, v/v) as the eluent. **^{1}H NMR (400 MHz, $CDCl_3$) δ** 10.02 (s, CHO, 1H), 8.11 (d, *J* = 8.6 Hz, A part of AB system, 1H), 8.06 (d, *J* = 1.3 Hz, 1H), 7.85 (dd, *J* = 8.6, 1.3 Hz, B part of AB system, 1H), 7.82 – 7.75 (m, AA' part of AA'BB' system, 2H), 7.67 (d, *J* = 3.6 Hz, 1H), 7.45 – 6.95 (m, AA' part of AA'BB' system, 2H), 6.77 (d, *J* = 3.6 Hz, 1H), 2.35 (s, 3H). **^{13}C{^{1}H} NMR (101 MHz, $CDCl_3$) δ** 191.9, 145.7, 138.2, 135.1, 132.4, 131.0, 130.2, 128.2, 127.0, 125.4, 124.9, 114.1, 109.5, 21.7.

7-Fluoro-1-tosyl-1*H*-indole (S46)[3]

White solid (2.60 g, 90%, m.p: 116–117 °C). Ethyl acetate/hexane (10:90, v/v) as the eluent. **^{1}H NMR (400 MHz, $CDCl_3$) δ** 7.82 – 7.76 (m, AA' part of AA'BB' system, 2H), 7.73 (d, *J* = 3.6 Hz, 1H), 7.27 (d, *J* = 8.0 Hz, 1H), 7.24 – 7.20 (m, BB' part of AA'BB' system, 2H), 7.07 (td, *J* = 8.0, 4.2 Hz, 1H), 6.90 (dd, *J* = 12.0, 8.0 Hz, 1H), 6.63 (dd, *J* = 3.6, 2.3 Hz, 1H), 2.33 (s, 3H). **^{13}C{^{1}H} NMR (101 MHz, $CDCl_3$) δ** 149.8 (d, *J* = 250.3 Hz), 145.1, 135.7, 135.2 (d, *J* = 3.6 Hz), 129.9, 128.9, 127.8 (d, *J* = 2.4 Hz), 124.1 (d, *J* = 6.8 Hz), 122.0 (d, *J* = 10.7 Hz), 117.3 (d, *J* = 3.8 Hz), 111.2 (d, *J* = 19.9 Hz), 107.8 (d, *J* = 1.5 Hz), 21.7.

1-Tosyl-1*H*-benzo[*g*]indole (S47)[5]

Ethyl acetate/hexane (10:90, v/v) as the eluent. White solid (2.39 g, 92%, m.p: 92–93 °C). **^{1}H NMR (400 MHz, $CDCl_3$) δ** 9.09 (d, *J* = 8.6 Hz, 1H), 7.93 (d, *J* = 3.7 Hz, 1H), 7.86 (d, *J* = 8.6 Hz, 1H), 7.65 (d, *J* = 8.6 Hz, 1H), 7.60 – 7.54 (m, 3H), 7.54 – 7.49 (m, 1H), 7.45 – 7.40 (m, 1H), 7.13 – 7.07 (m, BB' part of AA'BB' system, 2H), 6.82 (d, *J* = 3.7 Hz, 1H), 2.25 (s, 3H). **^{13}C{^{1}H} NMR (101 MHz, $CDCl_3$) δ** 145.0, 135.6, 132.4, 130.7, 130.0, 129.9, 129.7, 129.1, 126.9, 126.4, 126.0, 124.8, 123.9, 123.3, 120.2, 109.6, 21.6.

General Procedure F: Preparation of 3-Bromoindole Derivatives[6]

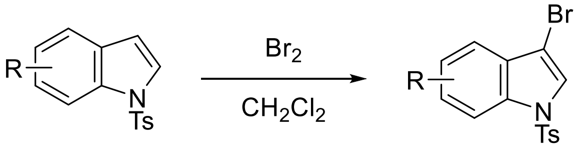

S43–S47 **S48–S52**

To a solution of 1-tosyl-1*H*-indole derivative (**S43–S47**) (10 mmol) in CH_2Cl_2 (15 mL) was added dropwise Br_2 (0.56 mL, 11 mmol) solution in 15 mL dichloromethane at 0 °C. After stirring the mixture

for 3 hours at room temperature, the solvent was evaporated under reduced pressure. The residue was purified by column chromatography on a silica gel column (eluent: ethyl acetate/hexane, v/v) to afford the 1-((trifluoromethyl)sulfonyl)-1*H*-indole-3-carbaldehydes (**S48–S52**).

3-Bromo-1-tosyl-1*H*-indole (S48)[6]

White solid (1.77 g, 98%, m.p: 125–126 ºC). Ethyl acetate/hexane (10:90, v/v) as the eluent. **^{1}H NMR (400 MHz, $CDCl_3$) δ** 7.99 (d, *J* = 8.2 Hz, =CH 1H), 7.79 – 7.74 (m, AA' part of AA'BB' system, =CH, 2H), 7.62 (s, =CH, 1H), 7.49 (d, *J* = 7.7 Hz, =CH, 1H), 7.43 – 7.34 (m, =CH, 1H), 7.34 – 7.27 (m, =CH, 1H), 7.20 – 7.25 (m, BB' part of AA'BB' system, =CH, 2H), 2.34 (s, 3H). **^{13}C{^{1}H} NMR (101 MHz, $CDCl_3$) δ** 145.5, 135.0, 134.4, 130.2, 129.9, 127.1, 125.9, 124.9, 124.0, 120.2, 113.8, 99.7, 21.7.

3-Bromo-5-phenyl-1-tosyl-1*H*-indole (S49)

White solid (2.49 g, 97%, m.p: 129–130 ºC). Ethyl acetate/hexane (10:90, v/v) as the eluent. **^{1}H NMR (400 MHz, $CDCl_3$) δ** 8.05 (d, *J* = 8.6 Hz, A part of AB system, 1H), 7.83 – 7.78 (m, AA' part of AA'BB' system, 2H), 7.67 (d, *J* = 1.6 Hz, 1H), 7.65 (s, 1H), 7.63 – 7.58 (m, 3H), 7.45 (t, *J* = 7.6 Hz, 2H), 7.39 – 7.32 (m, 1H), 7.29 – 7.23 (m, BB' part of AA'BB' system, 2H), 2.36 (s, 3H). **^{13}C{^{1}H} NMR (101 MHz, $CDCl_3$) δ** 145.6, 140.9, 137.7, 135.0, 133.8, 130.4, 130.2, 128.0, 127.52, 127.48, 127.1, 125.5, 118.5, 114.0, 100.0, 21.8. (One carbon signal overlapped). **HRMS (ESI-TOF) *m/z*:** $[M + H]^+$ calcd for $C_{21}H_{17}BrNO_2S$, 426.0158; found 426.0155.

3-Bromo-1-tosyl-1*H*-indole-5-carbaldehyde (S50)

Brown solid (1.98 g, 95%, m.p: 159–160 ºC). Ethyl acetate/hexane (10:90, v/v) as the eluent. **^{1}H NMR (400 MHz, $CDCl_3$) δ** 10.07 (s, CHO, 1H), 8.12 (d, *J* = 8.7 Hz, A part of AB system, 1H), 8.03 (d, *J* = 1.4 Hz, 1H), 7.92 (dd, *J* = 8.7, 1.4 Hz, B part of AB system, 1H), 7.87 – 7.77 (m, AA' part of AA'BB' system, 2H), 7.72 (s, 1H), 7.30 – 7.26 (m, BB' part of AA'BB' system, 2H), 2.36 (s, 3H). **^{13}C{^{1}H} NMR (101 MHz, $CDCl_3$) δ** 191.5, 146.2, 137.6, 134.6, 132.7, 130.4, 130.2, 127.2, 126.6, 126.2, 123.8, 114.3, 99.9, 21.8. **HRMS (ESI-TOF) *m/z*:** $[M + H]^+$ calcd for $C_{16}H_{13}BrNO_3S$, 377.9794; found 377.9795.

3-Bromo-7-fluoro-1-tosyl-1*H*-indole (S51)

Off-white solid (1.69 g, 85%, m.p: 113–114 ºC). Ethyl acetate/hexane (10:90, v/v) as the eluent. **^{1}H NMR (400 MHz, $CDCl_3$) δ** 7.88 – 7.79 (m, 3H), 7.33 – 7.26 (m, 3H), 7.25 – 7.18 (m, 1H), 7.07 – 6.98 (m, 1H), 2.39 (s, 3H). **^{13}C{^{1}H} NMR (101 MHz, $CDCl_3$) δ** 149.4 (d, *J* = 252.0 Hz), 145.6, 135.1, 133.9 (d, *J* = 3.6 Hz), 130.1, 127.9 (d, *J* = 2.5 Hz), 127.3, 124.6 (d, *J* = 6.9 Hz), 121.6, 116.0 (d, *J* = 3.8 Hz), 112.3 (d, *J* = 19.9 Hz), 98.0 (d, *J* = 2.9 Hz), 21.8. **HRMS (ESI-TOF) *m/z*:** $[M + H]^+$ calcd for $C_{15}H_{12}BrFNO_2S$, 367.9751; found 367.9750.

3-Bromo-1-tosyl-1*H*-benzo[*g*]indole (S52)

Off-white solid (2.19 g, 95%, m.p: 123–124 ºC). Ethyl acetate/hexane (10:90, v/v) as the eluent. **^{1}H NMR (400 MHz, $CDCl_3$) δ** 9.07 (d, *J* = 8.5 Hz, 1H), 7.99 (s, 1H), 7.88 (d, *J* = 8.1 Hz, 1H), 7.72 (d, *J* = 8.5 Hz, 1H), 7.61 – 7.52 (m, 4H), 7.47 (t, *J* = 7.5 Hz, 1H), 7.15 – 7.08 (m, BB' part of AA'BB' system, 2H), 2.27 (s, 3H). **^{13}C{^{1}H} NMR (101 MHz, $CDCl_3$) δ** 145.4, 134.9, 132.8, 130.1, 129.3, 128.7, 127.9, 127.6, 127.0, 126.8, 126.7, 125.5, 124.0, 122.9, 118.3, 100.2, 21.7. **HRMS (ESI-TOF) *m/z*:** $[M + H]^+$ calcd for $C_{19}H_{15}BrNO_2S$, 400.0001; found 400.0006.

3-(4,4,5,5-Tetramethyl-1,3,2-dioxaborolan-2-yl)-1-tosyl-1*H*-indole (S53)

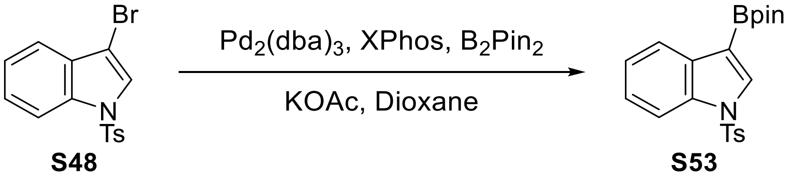

S48 **S53**

A mixture of 3-bromo-1-tosyl-1*H*-indole (**S48**) (700 mg, 2 mmol), KOAc (589 g, 6 mmol), and B_2pin_2 (609 mg, 5.61 mmol) in dioxane (4 mL) were prepared. After dissolved gases were removed by bubbling N_2 gas through the mixture for about 10 minutes, $Pd_2(dba)_3$ (36.6 mg, 0.39 mmol) and Xphos (38.1 mg, 0.79 mmol) were added to the mixture, and the reaction mixture was stirred in an oil bath at 110 °C for 20 hours. After cooling to room temperature, the mixture was filtered over celite (with 40 mL of EtOAc). The ethyl acetate layer was washed with (3×40 mL) and dried over anhydrous Na_2SO_4. The solvent was removed under vacuum. The crude product was purified by column chromatography on silica gel (eluent: ethyl acetate/hexane, 2:98, v/v) to afford the compound **S53** (604 mg, 76%, m.p: 126–127 °C). **^{1}H NMR (400 MHz, CDCl$_3$) δ** 8.01 (s, 1H), 7.97 – 7.92 (m, 2H), 7.84 – 7.77 (m, AA' part of AA'BB' system, 2H), 7.33 – 7.24 (m, 2H), 7.24 – 7.19 (m, BB' part of AA'BB' system, 2H), 2.33 (s, 3H), 1.36 (s, 12H). **^{13}C{^{1}H} NMR (101 MHz, CDCl$_3$) δ** 145.2 (2C), 135.5(2C), 135.3, 133.8, 130.0, 127.1, 124.6, 123.5, 123.2, 113.2, 83.7, 25.0, 21.7. **HRMS (APCI-TOF) *m/z*:** $[M + H]^+$ calcd for $C_{21}H_{25}BNO_4S$, 397.1628; found 397.1623.

General Procedure G: Preparation of Suzuki Coupling Products (S57–S59, S62, S63–S66)

To a solution of boronic ester (**S53** or **S62**) (1 mmol, 1 equiv.), vinylbromide (**S54–S56,**[7] **S60**) or arylbromide (**S48–S52**) (1.1 mmol, 1.1 eq) in a mixture of dioxane (20 mL) and H_2O (10 mL) was added Cs_2CO_3 (530 mg, 1.5 mmol, 1.5 equiv.). After dissolved gases were removed by bubbling N_2 gas through the solution for about 10 minutes, $Pd(PPh_3)_4$ (0.04 mmol, 0.04 equiv.) was added to the mixture, and the reaction mixture was stirred in an oil bath at 110 °C for 16 hours. After the reaction mixture was cooled to room temperature, the mixture was extracted with dichloromethane (3×30 mL). The combined organic layers were dried over anhydrous Na_2SO_4, filtered, and the solvent was removed under vacuum. The residue was purified by column chromatography on silica gel (eluent: ethyl acetate/hexane, v/v) to afford the desired coupling products (**S57–S59, S61, S63–S66**).

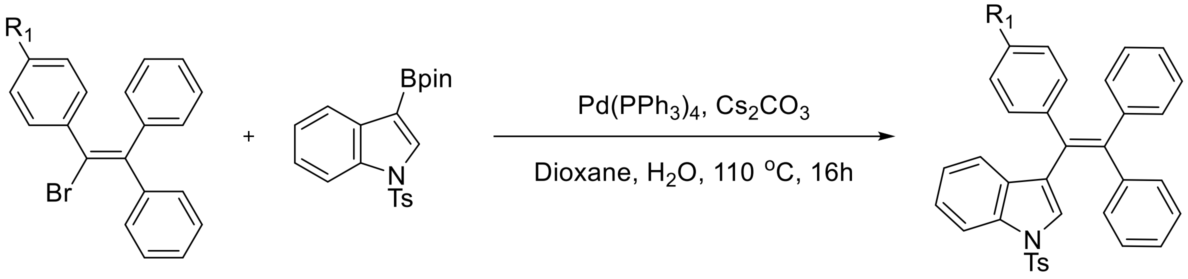

S54: R_1= H
S55: R_1= OMe
S56: R_1= NO_2

S53

S57: R_1= H
S58: R_1= OMe
S59: R_1= NO_2

1-Tosyl-3-(1,2,2-triphenylvinyl)-1*H*-indole (S57)

White solid (394 mg, 75%, m.p: 179–180 °C). Ethyl acetate/hexane (10:90, v/v) as the eluent. **^{1}H NMR (400 MHz, CDCl$_3$) δ** 7.83 (d, *J* = 8.3 Hz, =CH, 1H), 7.48 – 7.45 (m, AA' part of AA'BB' system, 2H), 7.26 (s, 1H), 7.23 – 6.89 (m, =CH, 19H), 6.83 (d, *J* = 7.9 Hz, 1H), 2.39 (s, 3H). **^{13}C{^{1}H} NMR (101 MHz, CDCl$_3$) δ** 144.7, 144.2, 143.1, 143.0, 141.9, 135.4, 134.9, 131.8, 131.5, 130.9, 130.8, 130.4, 129.9, 128.1, 127.9, 127.9, 127.3, 127.03, 126.97, 126.9, 126.8, 125.0, 124.4, 123.1, 121.4, 113.4, 21.8. **HRMS (APCI-TOF) *m/z*:** $[M + H]^+$ calcd for $C_{35}H_{28}NO_2S$, 526.1835; found 526.1832.

3-(1-(4-Methoxyphenyl)-2,2-diphenylvinyl)-1-tosyl-1*H*-indole (S58)

White solid (440 mg, 79%, m.p: 205–206 °C). Ethyl acetate/hexane (5:95, v/v) as the eluent. **^{1}H NMR (400 MHz, CDCl$_3$) δ** 7.83 (d, *J* = 8.3 Hz, 1H), 7.54 – 7.42 (m, AA' part of AA'BB' system, 2H), 7.26 (s, 1H), 7.24 – 6.93 (m, 16H), 6.85 (d, *J* = 8.0 Hz, 1H), 6.69 – 6.53 (m, BB' part of AA'BB' system, 2H), 3.73 (s, 3H), 2.40 (s, 3H). **^{13}C{^{1}H} NMR (101 MHz, CDCl$_3$) δ** 158.5, 144.7, 144.5, 143.3, 142.1, 135.4, 135.0, 134.1, 132.1, 131.5, 131.3, 130.9, 130.4, 129.9, 128.03, 127.97, 127.3, 127.0, 126.7, 126.6,

125.1, 124.3, 123.1, 121.5, 113.4, 113.3, 55.2, 21.7. **HRMS (APCI-TOF) *m/z*:** $[M + H]^+$ calcd for $C_{36}H_{30}NO_3S$, 556.1941; found 556.1942.

3-(1-(4-Nitrophenyl)-2,2-diphenylvinyl)-1-tosyl-1*H*-indole (S59)

Yellow solid (468 mg, 82%, m.p: 178–179 °C). Ethyl acetate/hexane (5:95, v/v) as the eluent. **^{1}H NMR (400 MHz, $CDCl_3$) δ** 7.99 – 7.90 (m, AA' part of AA'BB' system, 2H), 7.87 (d, J = 8.3 Hz, 1H), 7.53 – 7.43 (m, AA' part of AA'BB' system, 2H), 7.25 – 7.12 (m, 10H), 7.09 – 7.01 (m, 6H), 6.99 (t, J = 7.4 Hz, 1H), 6.78 (d, J = 7.9 Hz, 1H), 2.42 (s, 3H). **$^{13}C\{^1H\}$ NMR (101 MHz, $CDCl_3$) δ** 149.2, 146.4, 146.3, 145.0, 143.3, 141.9, 135.2, 134.9, 131.6, 131.4, 130.3, 130.1, 130.0, 129.7, 128.4, 128.2, 127.8, 127.6, 127.5, 127.0, 124.8, 123.6, 123.4, 123.3, 120.8, 113.6, 21.8. **HRMS (ESI-TOF) *m/z*:** $[M + H]^+$ calcd for $C_{35}H_{27}N_2O_4S$, 571.1686; found 571.1691

3,3'-(2,2-Diphenylethene-1,1-diyl)bis(1-tosyl-1*H*-indole) (S61)

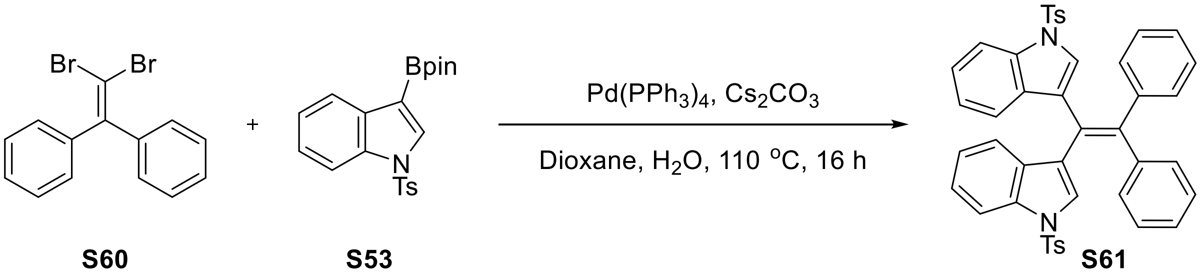

Boronic ester (**S53**) used 2.5 eqv. Off-white solid (503 mg, 70%, m.p: 210–211 °C). Ethyl acetate/hexane (10:90, v/v) as the eluent. **^{1}H NMR (400 MHz, $CDCl_3$) δ** 7.86 (d, J = 8.4 Hz, 2H), 7.46 – 7.37 (m, AA' part of AA'BB' system, 4H), 7.20 (s, 2H), 7.18 – 7.00 (m, 16H), 6.85 – 6.75 (m, 4H), 2.38 (s, 6H). **$^{13}C\{^1H\}$ NMR (101 MHz, $CDCl_3$) δ** 145.0, 144.8, 143.1, 135.1, 135.0, 130.5, 130.1, 129.9, 128.1, 127.2, 127.1, 126.8, 124.4, 124.4, 123.2, 122.2, 120.9, 113.6, 21.7. **HRMS (ESI-TOF) *m/z*:** $[M + H]^+$ calcd for $C_{44}H_{35}N_2O_4S_2$, 719.203; found 719.2032.

4,4,5,5-Tetramethyl-2-(1,2,2-triphenylvinyl)-1,3,2-dioxaborolane (S62)[8]

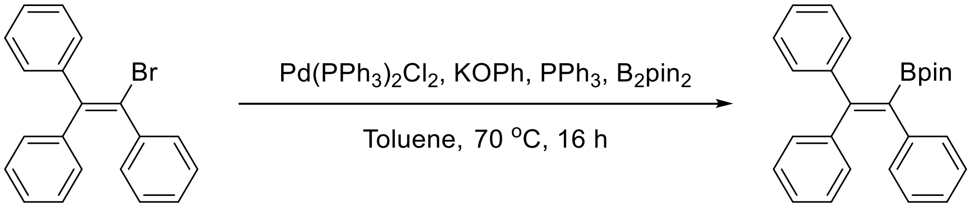

To the solution of (2-bromoethene-1,1,2-triyl)tribenzene (**S54**) (670 mg, 2.0 mmol) in 12 mL of toluene, $PdCl_2(PPh_3)_2$ (41 mg, 0.06 mmol), PPh_3 (32 mg, 0.12 mmol), bis(pinacolato)diboron (558 mg, 1.1 mmol) and KOPh (powder, 396 mg, 3.0 mmol) were added. The mixture was stirred at 70 °C in a sealed tube for 15 hours, then cooled to room temperature and extracted with dichloromethane (3×30 mL). The organic phase was dried over Na_2SO_4 and the solvent was evaporated in reduced pressure. The crude product was purified from a silica gel column with an EtOAc/hexane (4:96) mixture to give **S63** as a white solid (481 mg, 63%, m.p 78–79 °C). **^{1}H NMR (400 MHz, $CDCl_3$) δ** 7.40 – 7.27 (m, 5H), 7.18 – 7.04 (m, 8H), 7.00 – 6.95 (m, 2H), 1.14 (s, 12H). **$^{13}C\{^1H\}$ NMR (101 MHz, $CDCl_3$) δ** 151.5, 144.8, 141.9, 141.8, 131.0, 129.8, 129.5, 128.1, 128.1, 127.7, 127.6, 126.9, 126.0, 83.8, 24.6. (One carbon signals overlapped).

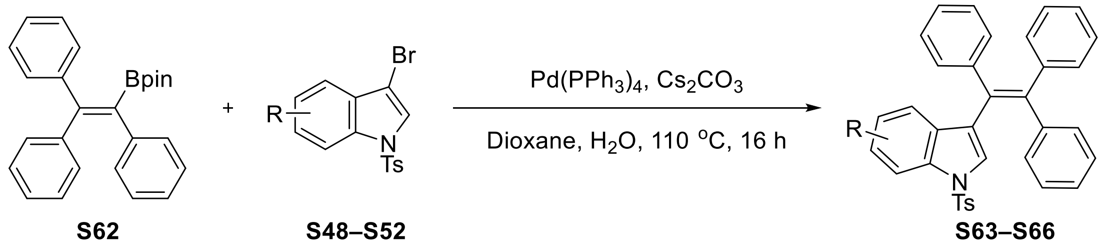

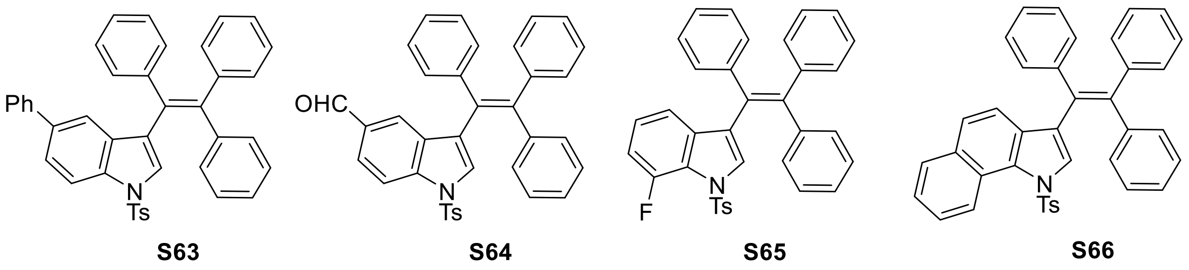

5-Phenyl-1-tosyl-3-(1,2,2-triphenylvinyl)-1*H*-indole (S63)

White solid (493 mg, 82%, m.p: 207–208 °C). Ethyl acetate/hexane (5:95, v/v) as the eluent. **^{1}H NMR (400 MHz, $CDCl_3$) δ** 7.91 (d, *J* = 8.6 Hz, 1H), 7.60 – 7.50 (m, AA' part of AA'BB' system, 2H), 7.41 (dd, *J* = 8.6, 1.7 Hz, 1H), 7.35 – 7.30 (m, 2H), 7.29 – 7.21 (m, 6H), 7.16 – 7.02 (m, 16H), 2.42 (s, 3H). **^{13}C{^{1}H} NMR (101 MHz, $CDCl_3$) δ** 144.8, 144.1, 143.0, 142.9, 142.2, 141.3, 136.4, 135.3, 134.4, 131.7, 131.5, 131.1, 131.0, 130.5, 130.0, 128.7, 128.1, 128.0, 127.98, 127.89, 127.3, 127.04, 127.00, 126.96, 126.94, 126.87, 125.6, 123.9, 120.0, 113.6, 21.8. **HRMS (ESI-TOF) *m/z*:** [M + H]$^+$ calcd for $C_{41}H_{32}NO_2S$, 602.2148; found 602.2156.

1-Tosyl-3-(1,2,2-triphenylvinyl)-1*H*-indole-5-carbaldehyde (S64)

Off-white solid (420 mg, 76%, m.p: 165–166 °C). Ethyl acetate/hexane (5:95, v/v) as the eluent. **^{1}H NMR (400 MHz, $CDCl_3$) δ** 9.72 (s, CHO, 1H), 7.96 (d, *J* = 8.6 Hz, 1H), 7.73 (dd, *J* = 8.6, 1.5 Hz, 1H), 7.57 – 7.46 (m, AA' part of AA'BB' system, 2H), 7.32 (d, *J* = 1.3 Hz, 1H), 7.30 (s, 1H), 7.24 (d, *J* = 8.2 Hz, 2H), 7.17 – 6.98 (m, 15H), 2.41 (s, 3H). **^{13}C{^{1}H} NMR (101 MHz, $CDCl_3$) δ** 191.9, 145.4, 143.9, 143.8, 142.6 (2C), 141.7, 138.1, 134.9, 132.0, 131.4, 130.7, 130.8, 130.4, 130.1, 128.8, 128.1 (2C), 128.0, 127.3, 127.1, 127.0 (2C), 125.7, 125.6, 124.4, 113.9, 21.8. **HRMS (ESI-TOF) *m/z*:** [M + H]$^+$ calcd for $C_{36}H_{28}NO_3S$, 554.1784; found 554.1787.

7-Fluoro-1-tosyl-3-(1,2,2-triphenylvinyl)-1*H*-indole (S65)

Off-White solid (424 mg, 78%, m.p: 184–185 °C). Ethyl acetate/hexane (5:95, v/v) as the eluent. **^{1}H NMR (400 MHz, $CDCl_3$) δ** 7.52 – 7.42 (m, AA' part of AA'BB' system, 2H), 7.36 (s, 1H), 7.25 – 7.21 (m, BB' part of AA'BB' system, 2H), 7.21 – 7.04 (m, 15H), 6.91 – 6.78 (m, 2H), 6.64 (d, *J* = 7.6 Hz, 1H), 2.41 (s, 3H). **^{13}C{^{1}H} NMR (101 MHz, $CDCl_3$) δ** 149.5 (d, *J* = 250.4 Hz), 144.9, 144.4, 143.7, 142.7, 141.4, 135.5, 135.0 (d, *J* = 3.1 Hz), 131.4, 130.8, 130.4, 129.8, 129.7, 128.2, 128.0, 127.9, 127.8 (d, *J* = 1.5 Hz), 127.1, 127.0, 126.8, 123.7 (d, *J* = 6.6 Hz), 123.4 (d, *J* = 1.1 Hz), 121.9 (d, *J* = 11.1 Hz), 117.0 (d, *J* = 3.7 Hz), 110.9 (d, *J* = 19.8 Hz), 21.8 (s), (1 carbon signal overlappedtır). **HRMS (ESI-TOF) *m/z*:** [M + H]$^+$ calcd for $C_{35}H_{27}FNO_2S$, 554.1741; found 554.1747.

1-Tosyl-3-(1,2,2-triphenylvinyl)-1*H*-benzo[*g*]indole (S66)

Off-white solid (460 mg, 80%, m.p: 241–242 °C). Ethyl acetate/hexane (5:95, v/v) as the eluent. **^{1}H NMR (400 MHz, $CDCl_3$) δ** 9.00 (d, *J* = 8.7 Hz, 1H), 7.77 (d, *J* = 7.9 Hz, 1H), 7.58 (s, 1H), 7.47 (t, *J* = 7.5 Hz, 1H), 7.42 (d, *J* = 8.6 Hz, 1H), 7.38 (t, *J* = 7.5 Hz, 1H), 7.33 – 7.28 (m, AA' part of AA'BB' system, 2H), 7.20 – 7.04 (m, 18H), 2.33 (s, 3H). **^{13}C{^{1}H} NMR (101 MHz, $CDCl_3$) δ** 144.8, 144.4,

143.7, 142.8, 141.8, 135.4, 132.2, 131.7, 131.5, 130.8, 130.7, 130.5, 130.01, 129.97, 129.8, 129.0, 128.04, 127.97 (2C), 127.2, 127.00, 126.97, 126.7, 126.2, 125.6, 125.3, 124.7, 123.8, 123.1, 119.6, 21.7. **HRMS (ESI-TOF) *m/z*:** [M + H]$^+$ calcd for $C_{39}H_{30}NO_2S$, 576.1992; found 576.1994.

General Procedure H: Removal of Tosyl Group Attached to Nitrogen Atom (Preparation of 3a-3m)

To a solution of *N*-tosyl indole derivative (**S57–S59, S61, S63–S66**) (0.5 mmol l) in 1,4-dioxane (10 mL) was added NaOH (10 M, 10 mL), and the resultant mixture was then stirred at 100 °C for 16 hours. The resultant mixture was extracted with dichloromethane (3×30 mL), and the organic layer was washed with H_2O, dried (Na_2SO_4), filtered, and concentrated under reduced pressure. The crude product was purified by column chromatography on silica gel (eluent: ethyl acetate/hexane, v/v) to afford the *N*-free indole derivatives (**3a, 3d, 3g-3m**).

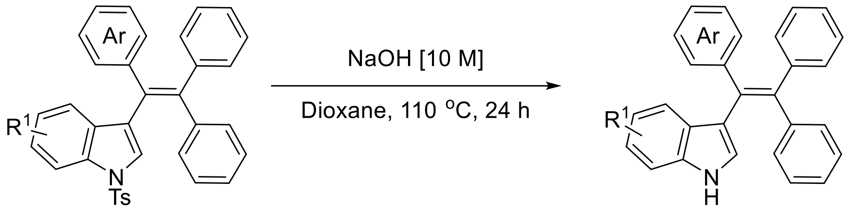

S57–S59, S61, S63–S66 **3a, 3d, 3g–3m**

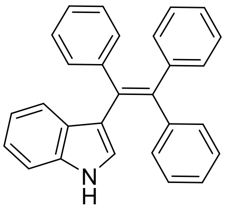

3a

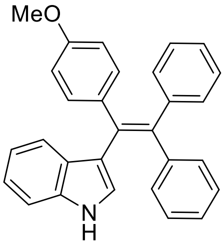

3d

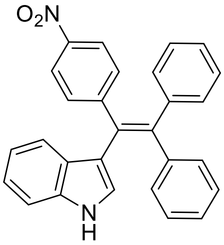

3g

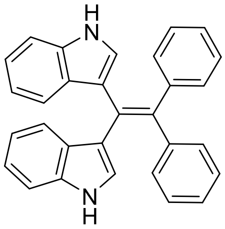

3i

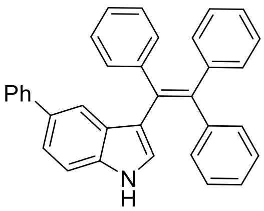

3j

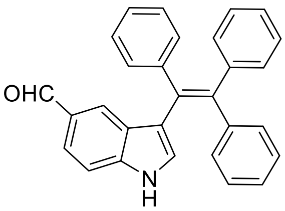

3k

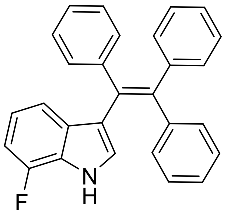

3l

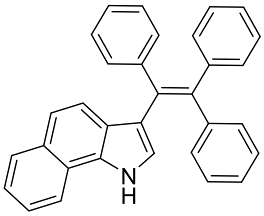

3m

3-(1,2,2-Triphenylvinyl)-1*H*-indole (3a)

White solid (150 mg, 81%, m.p: 233–234 °C). Ethyl acetate/hexane (5:95, v/v) as the eluent. **^{1}H NMR (400 MHz, DMSO-*d*$_6$) δ** 11.00 (bs, 1H), 7.26 (d, *J* = 8.1 Hz, 1H), 7.18 – 7.02 (m, 13H), 7.00 – 6.89 (m, 3H), 6.75 (d, *J* = 2.4 Hz, 1H), 6.70 (t, *J* = 7.4 Hz, 1H), 6.64 (d, *J* = 7.9 Hz, 1H). **^{13}C{^{1}H} NMR (101 MHz, DMSO-*d*$_6$) δ** 144.8, 143.6, 143.3, 138.7, 135.9, 134.4, 131.0, 130.6, 130.1, 127.8, 127.7, 127.6, 127.0, 126.8, 126.4, 126.11, 126.05, 120.8, 119.6, 118.7, 116.9, 111.4. **HRMS (APCI-TOF) *m/z*:** [M + H]$^+$ calcd for $C_{28}H_{22}N$, 372.1747; found 372.1748.

3-(1-(4-Methoxyphenyl)-2,2-diphenylvinyl)-1*H*-indole (3d)

Light-yellow solid (140 mg, 70%, m.p: 201–202 °C). Ethyl acetate/hexane (5:95, v/v) as the eluent. **^{1}H NMR (400 MHz, Aseton-*d*$_6$) δ** 10.13 (bs, 1H), 7.31 (d, *J* = 8.2 Hz, 1H), 7.17 – 7.02 (m, 12H), 7.02 – 6.96 (m, 1H), 6.85 – 6.78 (m, 2H), 6.75 (t, *J* = 7.5 Hz, 1H), 6.69 – 6.63 (m, BB' part of AA'BB' system, 2H), 3.72 (s, 3H). **^{13}C{^{1}H} NMR (101 MHz, DMSO-*d*$_6$) δ** 159.2, 146.4, 145.4, 139.5, 137.4, 136.8,

135.2, 133.0, 132.3, 131.4, 128.5, 128.5, 127.8, 126.7, 122.0, 121.2, 119.8, 119.1, 114.1, 113.8 (2C), 112.1, 55.3. **HRMS (APCI-TOF) *m/z*:** $[M + H]^+$ calcd for $C_{29}H_{24}NO$, 402.1852; found 402.1859.

3-(1-(4-Nitrophenyl)-2,2-diphenylvinyl)-1*H*-indole (3g)

Red solid (177 mg, 85%, m.p: 112–113 °C). Ethyl acetate/hexane (10:90, v/v) as the eluent. **^{1}H NMR (400 MHz, DMSO-*d$_6$*) δ** 11.14 (bs, 1H), 8.04 – 7.92 (m, AA' part of AA'BB' system, 2H), 7.36 – 7.26 (m, 3H), 7.21 – 7.05 (m, 8H), 7.04 – 6.95 (m, 3H), 6.86 (s, 1H), 6.75 (t, *J* = 7.4 Hz, 1H), 6.69 (d, *J* = 8.0 Hz, 1H). **^{13}C{^{1}H} NMR (101 MHz, DMSO-*d$_6$*) δ** 151.1, 145.4, 144.0, 142.7, 141.6, 136.0, 132.6, 131.8, 131.0, 130.0, 128.1, 127.9, 127.3, 126.9, 126.7, 126.3, 122.9, 121.1, 119.2, 119.0, 116.1, 111.7. **HRMS (ESI-TOF) *m/z*:** $[M + H]^+$ calcd for $C_{28}H_{21}N_2O_2$, 417.1598; found 417.1598.

3,3'-(2,2-Diphenylethene-1,1-diyl)bis(1*H*-indole) (3i)

Off-white solid (160 mg, 78%, m.p: 259–260 °C). Ethyl acetate/hexane (10:90, v/v) as the eluent. **^{1}H NMR (400 MHz, $CDCl_3$) δ** 7.90 (bs, 2H), 7.25 (d, *J* = 8.2 Hz, 2H), 7.18 (dd, *J* = 7.8, 1.5 Hz, 4H), 7.10 – 7.01 (m, 8H), 6.96 (d, *J* = 8.0 Hz, 2H), 6.81 (d, *J* = 2.5 Hz, 2H), 6.78 (t, *J* = 7.5 Hz, 2H). **^{13}C{^{1}H} NMR (101 MHz, $CDCl_3$) δ** 145.3, 138.8, 135.9, 131.1, 127.7, 127.4, 126.6, 126.2, 125.9, 121.7, 121.0, 119.6, 119.5, 110.8. **HRMS (ESI-TOF) *m/z*:** $[M + H]^+$ calcd for $C_{30}H_{23}N_2$, 411.1856; found 411.1857.

3j: Since **3j** could not be purified, it was used directly in the dehydrogenative-photocyclization step.

3-(1,2,2-Triphenylvinyl)-1*H*-indole-5-carbaldehyde (3k)

Off-white solid (160 mg, 80%, m.p: 258–259 °C). Ethyl acetate/hexane (10:90, v/v) as the eluent. **^{1}H NMR (400 MHz, DMSO-*d$_6$*) δ** 11.54 (bs, 1H), 9.64 (s, CHO, 1H), 7.51 (dd, *J* = 8.5, 1.2 Hz, 1H), 7.42 (d, *J* = 8.5 Hz, 1H), 7.31 (s, 1H), 7.17 – 6.98 (m, 15H), 6.95 (d, *J* = 2.4 Hz, 1H**). ^{13}C{^{1}H} NMR (101 MHz, DMSO-*d$_6$*) δ** 192.1, 144.3, 143.3, 143.2, 140.0, 139.3, 133.3, 130.9, 130.5, 130.0, 128.9, 128.5, 127.85, 127.82, 127.77, 126.6, 126.42, 126.35, 126.30, 125.0, 121.0, 119.3, 112.2. **HRMS (ESI-TOF) *m/z*:** $[M + H]^+$ calcd for $C_{29}H_{22}NO$, 400.1696; found 400.1696.

7-Fluoro-3-(1,2,2-triphenylvinyl)-1*H*-indole (3l)

Off-white solid (146 mg, 75%, m.p: 215–216 °C). Ethyl acetate/hexane (10:90, v/v) as the eluent. **^{1}H NMR (400 MHz, $CDCl_3$) δ** 8.07 (bs, 1H), 7.24 – 7.06 (m, 15H), 6.86 – 6.70 (m, 3H), 6.60 (d, *J* = 7.7 Hz, 1H). **^{13}C{^{1}H} NMR (101 MHz, $CDCl_3$) δ** 149.4 (d, *J* = 243.5 Hz), 145.0, 143.8, 143.2 (2C), 140.6, 133.6, 131.6 (2C), 131.2, 130.8, 127.9, 127.8 (2C), 127.0, 126.6, 126.4 (d, *J* = 4.1 Hz), 124.3 (d, *J* = 13.1 Hz), 120.0, 119.8 (d, *J* = 6.1 Hz), 116.7 (d, *J* = 3.3 Hz), 106.7 (d, *J* = 15.7 Hz). **HRMS (ESI-TOF) *m/z*:** $[M + H]^+$ calcd for $C_{28}H_{21}FN$, 390.1653; found 390.1652.

3-(1,2,2-Trifenilvinil)-1*H*-benzo[*g*]indole (3m)

Off-white solid (169 mg, 80%, m.p: 267–268 °C). Ethyl acetate/hexane (10:90, v/v) as the eluent. **^{1}H NMR (400 MHz, DMSO-*d$_6$*) δ** 11.94 (bs, 1H), 8.24 (d, *J* = 8.0 Hz, 1H), 7.79 (d, *J* = 7.8 Hz, 1H), 7.49 (t, *J* = 7.4 Hz, 1H), 7.35 (t, *J* = 7.4 Hz, 1H), 7.22 – 6.96 (m, 16H), 6.91 – 6.77 (m, 2H). **^{13}C{^{1}H} NMR (101 MHz, DMSO-*d$_6$*) δ** 144.7, 143.6, 143.5, 139.2, 134.3, 131.0, 130.6, 130.3, 130.2, 129.4, 128.2, 127.8 (3C), 127.7, 126.5, 126.2, 125.3, 124.8, 123.6, 122.7, 121.9, 120.4, 119.9, 119.3, 119.1. **HRMS (ESI-TOF) *m/z*:** $[M + H]^+$ calcd for $C_{32}H_{24}N$, 422.1903; found 422.1903.

4-(1-(1*H*-Indol-3-yl)-2,2-diphenylvinyl)-*N*-isopropylaniline (3h)

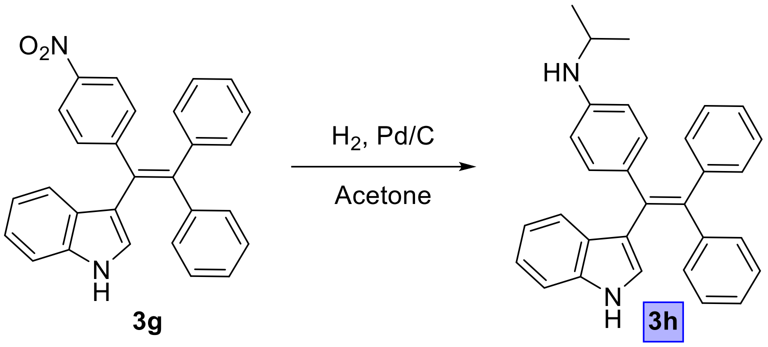

A solution of 3-(1-(4-nitrophenyl)-2,2-diphenylvinyl)-1*H*-indole (**3g**; 208 mg, 0.5 mmol) in acetone (15 mL) was cooled in an ice bath and Pd/C (21 mg, 10%) was quickly added to this solution. The dissolved gases in the resulting suspension were removed using a vacuum pump, and the environment was then saturated with hydrogen gas. After this process was repeated 3 times, the resulting suspension was saturated with H_2 gas in a balloon and stirred at room temperature for 12 hours. Finally, the reaction mixture was filtered through a blue band filter paper, and the solvent was removed under reduced pressure to yield 4-(1-(1*H*-indol-3-yl)-2,2-diphenylvinyl)-*N*-isopropylaniline (**3h**) as a yellow solid (204 mg, 95%, m.p: 139–140 °C). **^{1}H NMR (400 MHz, Acetone-*d*$_6$) δ** 10.08 (bs, 1H), 7.30 (dd, *J* = 8.1, 0.8 Hz, 1H), 7.16 – 6.95 (m, 11H), 6.92 (d, *J* = 8.0 Hz, 1H), 6.90 – 6.84 (m, AA' part of AA'BB' system, 2H), 6.82 – 6.73 (m, 2H), 6.40 – 6.29 (m, BB' part of AA'BB' system, 2H), 4.64 (bs, 1H), 3.55 (hept, *J* = 6.3 Hz, 1H), 1.14 (d, *J* = 6.3 Hz, 6H). **^{13}C{^{1}H} NMR (101 MHz, Acetone-*d*$_6$) δ** 147.6, 146.9, 145.9, 137.6, 137.2, 135.8, 132.8, 132.2, 131.8, 131.4, 128.5, 128.4 (2C), 128.3, 127.7, 126.3, 121.8, 121.4, 119.5, 119.4, 112.6, 112.0, 44.4, 22.9. **HRMS (ESI-TOF) *m/z*:** $[M + H]^+$ calcd for $C_{31}H_{29}N_2$, 429.2325; found 429.2315.

General Procedure I: Preparation of *N*-Alkylated-indole Derivatives (3b, 3c, 3e, 3f)

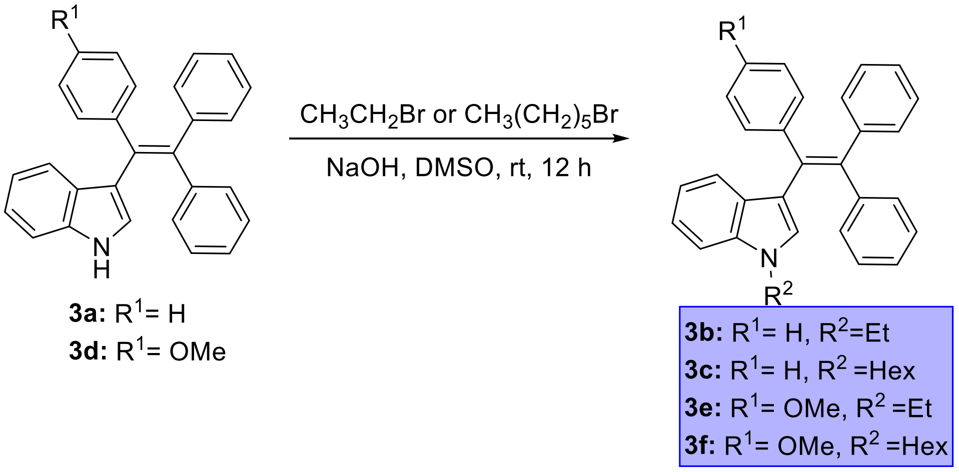

To the solution of indole derivative (**3a** or **3d**) (0.5 mmol) in DMSO (10 mL) was added NaOH (0.6 mmol). The mixture was stirred at room temperature for 30 minutes, and ethyl bromide (or hexyl bromide) (0.6 mmol) was added dropwise. After the reaction mixture was stirred at room temperature for 12 hours, H_2O (10 mL) was added to this mixture. The resultant mixture was extracted with EtOAc (2×30 mL), and the organic layer was washed with H_2O, dried (Na_2SO_4), filtered, and concentrated under reduced pressure. The crude product was purified by column chromatography on silica gel (eluent: ethyl acetate/hexane, v/v) to afford the N-alkylated-indole derivatives (**3b**, **3c**, **3e**, **3f**).

1-Ethyl-3-(1,2,2-triphenylvinyl)-1*H*-indole (3b)

Light-yellow solid (180 mg, 90%, m.p: 146–147 °C). Ethyl acetate/hexane (5:95, v/v) as the eluent. **^{1}H NMR (400 MHz, $CDCl_3$) δ** 7.42 – 6.89 (m, 17H), 6.84 (t, *J* = 7.0 Hz, 1H), 6.75 (d, *J* = 8.0 Hz, 1H), 6.64 (d, *J* = 1.7 Hz, 1H), 4.00 (q, *J* = 7.0 Hz, 2H), 1.27 (t, *J* = 7.0 Hz, 3H). **^{13}C{^{1}H} NMR (101 MHz, $CDCl_3$) δ** 145.5, 144.1, 143.5, 139.2, 135.8, 134.6, 131.7, 131.4, 131.0, 130.0, 128.1, 127.8, 127.7 (2C), 126.5, 126.1, 126.1, 121.3, 121.1, 119.1, 117.4, 109.1, 40.8, 15.5. **HRMS (APCI-TOF) *m/z*:** $[M + H]^+$ calcd for $C_{30}H_{26}N$, 400.2060; found 400.2060.

1-Hexyl-3-(1,2,2-triphenylvinyl)-1*H*-indole (3c)

Light-yellow solid (210 mg, 92%, m.p: 116–117 °C). Ethyl acetate/hexane (5:95, v/v) as the eluent. **^{1}H NMR (400 MHz, $CDCl_3$) δ** 7.23 (d, *J* = 8.2 Hz, 1H), 7.20 – 7.06 (m, 16H), 6.82 (t, *J* = 7.5 Hz, 1H), 6.75 (d, *J* = 8.0 Hz, 1H), 6.62 (s, 1H), 3.92 (t, *J* = 6.9 Hz, 2H), 1.71 – 1.37 (m, 2H), 1.35 – 1.19 (m, 4H), 1.19 – 1.07 (m, 2H), 0.89 (t, *J* = 6.9 Hz, 3H). **^{13}C{^{1}H} NMR (101 MHz, $CDCl_3$) δ** 145.5, 144.2, 143.6, 139.3, 136.1, 134.6, 131.7, 131.4, 130.9, 130.6, 128.1, 127.8, 127.7, 127.7, 126.5, 126.1, 126.1, 121.2, 121.1, 119.1, 117.3, 109.2, 46.3, 31.5, 30.1, 26.5, 22.6, 14.2. **HRMS (APCI-TOF) *m/z*:** $[M + H]^+$ calcd for $C_{34}H_{34}N$, 456.2686; found 456.2688.

1-Ethyl-3-(1-(4-methoxyphenyl)-2,2-diphenylvinyl)-1*H*-indole (3e)

Light-yellow solid (196 mg, 91%, m.p: 153–154 °C). Ethyl acetate/hexane (5:95, v/v) as the eluent. **^{1}H NMR (400 MHz, $CDCl_3$) δ** 7.23 (d, *J* = 8.1 Hz, 1H), 7.15 – 7.03 (m, 13H), 6.83 (t, *J* = 7.4 Hz, 1H), 6.74 (d, *J* = 7.9 Hz, 1H), 6.67 – 6.61 (m, BB' part of AA'BB' system, 2H), 6.59 (s, 1H), 3.96 (q, *J* = 7.2 Hz, 2H), 3.75 (s, 3H), 1.22 (t, *J* = 7.2 Hz, 3H). **^{13}C{^{1}H} NMR (101 MHz, $CDCl_3$) δ** 158.2, 145.8, 145.8, 144.4, 138.3, 135.8, 134.1, 132.5, 131.7, 131.0, 130.1, 128.3, 127.79, 127.77, 126.0, 125.9, 121.4, 121.1, 119.1, 117.51, 113.1, 109.1, 55.2, 40.8, 15.5. **HRMS (APCI-TOF) *m/z*:** $[M + H]^+$ calcd for $C_{31}H_{28}NO$, 430.2165; found 430.2166.

1-Hexyl-3-(1-(4-methoxyphenyl)-2,2-diphenylvinyl)-1*H*-indole (3f)

Light-yellow solid (214 mg, 88%, m.p: 170–171 °C). Ethyl acetate/hexane (5:95, v/v) as the eluent. **^{1}H NMR (400 MHz, $CDCl_3$) δ** 7.23 (d, *J* = 8.2 Hz, 1H), 7.16 – 7.03 (m, 13H), 6.83 (t, *J* = 7.4 Hz, 1H), 6.78 (d, *J* = 7.9 Hz, 1H), 6.67 – 6.63 (m, BB' part of AA'BB' system, 2H), 6.60 (s, 1H), 3.91 (t, *J* = 6.9 Hz, 2H), 3.75 (s, 3H), 1.66 – 1.55 (m, 2H), 1.33 – 1.17 (m, 4H), 1.15 – 1.05 (m, 2H), 0.89 (t, *J* = 6.8 Hz, 3H). **^{13}C{^{1}H} NMR (101 MHz, $CDCl_3$) δ** 158.2, 145.8, 144.5, 138.4, 136.1, 135.8, 134.0, 132.5, 131.8, 131.0, 130.7, 128.2, 127.81, 127.77, 126.0, 125.9, 121.4, 121.1, 119.0, 117.5, 113.1, 109.2, 55.2, 46.2, 31.5, 30.1, 26.5, 22.6, 14.2. HRMS (APCI-TOF) *m/z*: $[M + H]^+$ calcd for $C_{35}H_{36}NO$, 486.2791; found 486.2792.

3-Bromobenzofuran (S69)[9]

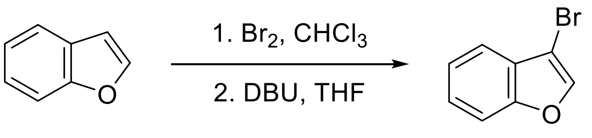

S67 **S68**

To a solution of benzofuran (**S67**) (500 mg, 4.23 mmol, 1 equiv.) in dichloromethane (15 mL), cooled to 0 °C, was added a solution of bromine (5.67 mL, 110 mmol) in dichloromethane (30 mL), and the mixture was stirred for 1 more hour at the same temperature. After adding 20 mL of water and 10 mL of $Na_2S_2O_3$ solution, the mixture was extracted with dichloromethane (3×30 mL). After the combined organic layer was dried over Na_2SO_4 and the solvent was evaporated under reduced pressure, the crude product was dissolved in anhydrous THF (10 mL). After the solution was cooled to 0 °C, 1,8-diazobicyclo[5.4.0]undec-7-ene (**DBU**; 1.26 mL, 8.46 mmol, 2 equiv.) was added dropwise. After 12

hours, the reaction was quenched with saturated NH_4Cl solution. The mixture was extracted with dichloromethane (3×40 mL). The organic phase was dried over Na_2SO_4 and the solvent was removed under reduced pressure. The residue was purified by column chromatography on a silica gel column (eluent: ethyl acetate/hexane, 5:95, v/v) to afford 3-bromobenzofuran (**S68**) as yellow oil (767 mg, 92%). **^{1}H NMR (400 MHz, $CDCl_3$) δ** 7.66 (s, 1H), 7.59 – 7.54 (m, 1H), 7.50 (d, *J* = 7.7 Hz, 1H), 7.41 – 7.30 (m, 2H). **^{13}C{^{1}H} NMR (101 MHz, $CDCl_3$) δ** 154.5, 142.8, 127.2, 125.6, 123.6, 119.9, 111.9, 98.1.

3-(1,2,2-Triphenylvinyl)benzofuran (3n)

Compound **3n** was prepared according to general procedure G. White solid (268 mg, 72%, m.p: 118–119 °C). Ethyl acetate/hexane (2:98, v/v) as the eluent. **^{1}H NMR (400 MHz, $CDCl_3$) δ** 7.40 (d, *J* = 8.2 Hz, 1H), 7.24 – 7.04 (m, 17H), 6.98 (t, *J* = 7.5 Hz, 1H), 6.79 (d, *J* = 7.8 Hz, 1H). **^{13}C{^{1}H} NMR (101 MHz, $CDCl_3$) δ** 155.2, 145.5, 144.1, 143.1, 142.6, 142.1, 131.5, 131.0, 130.54, 130.46, 128.1, 128.0, 127.85, 127.76, 127.0, 126.9, 126.8, 124.0, 123.1, 122.5, 121.4, 111.3. **HRMS (ESI-TOF) *m/z*:** $[M + H]^+$ calcd for $C_{28}H_{21}O$, 373.1587; found 373.1583.

3-(1,2,2-Triphenylvinyl)benzo[*b*]thiophene (3o)[10]

Compound **3o** was prepared according to general procedure G. Off-white solid (287 mg, 74%, m.p: 80–81 °C). Ethyl acetate/hexane (5:95, v/v) as the eluent. **^{1}H NMR (400 MHz, $CDCl_3$) δ** 7.79 (dd, *J* = 8.0, 0.5 Hz, 1H), 7.41 (d, *J* = 8.0 Hz, 1H), 7.26 (t, *J* = 7.5 Hz, 1H), 7.23 – 7.00 (m, 17H). **^{13}C{^{1}H} NMR (101 MHz, $CDCl_3$) δ** 144.0, 143.6, 143.1, 142.03, 139.98, 139.0, 138.8, 134.6, 131.6, 130.7, 130.2, 127.92, 127.90, 127.7, 127.2, 126.9, 126.8, 126.7, 124.00, 123.97, 123.6, 122.6.

2-(1,2,2-Triphenylvinyl)thiophene (3p)[10]

Compound **3p** was prepared according to general procedure G. Off-white solid (267 mg, 79%, m.p: 198–199 °C). Ethyl acetate/hexane (5:95, v/v) as the eluent. **^{1}H NMR (400 MHz, $CDCl_3$) δ** 7.21 – 7.13 (m, 5H), 7.11 – 7.05 (m, 5H), 7.03 (dd, *J* = 5.2, 1.1 Hz, 1H), 7.00 – 6.94 (m, 3H), 6.93 – 6.88 (m, 2H), 6.68 (dd, *J* = 5.2, 3.7 Hz, 1H), 6.49 (dd, *J* = 3.7, 1.1 Hz, 1H). **^{13}C{^{1}H} NMR (101 MHz, $CDCl_3$) δ** 146.5, 143.7, 143.28, 143.27, 141.3, 134.0, 131.4, 131.1, 131.0, 129.7, 128.4, 127.8, 127.7, 127.2, 127.1, 126.5, 126.3, 126.2. **HRMS (ESI-TOF) *m/z*:** $[M + H]^+$ calcd for $C_{24}H_{19}S$, 339.1208; found 339.1209.

General Procedure J: Dehydrogenative-Photocyclization

A) Preparation of 2a–2u

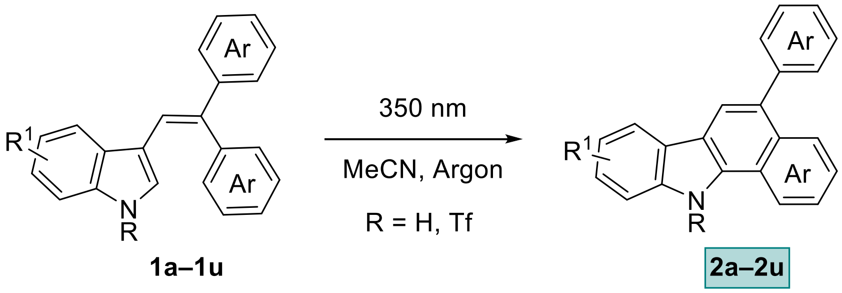

1a–1u **2a–2u**

In a round-bottom flask equipped with a magnetic stirring bar and condenser, triaryl vinyl or tetraaryl vinyl derivatives (**1a–1t** or **3a–3p**) (0.2 mmol) were dissolved in CH_3CN (2 mL). The solution was purged with the stream of Ar for 20 min and then irradiated in a Rayonet reactor with 16 lamps (350 nm, 1 lamp 14 W) under an argon atmosphere for 6 hours. The solution was evaporated on a rotary evaporator. Dehydrogenative-photocyclization products (**2a–2u** or **4a–4p**) were purified via silica gel column chromatography using EtOAc/petroleum ether as the eluent.

5-Phenyl-11*H*-benzo[*a*]carbazole (2a)[11]

Off-white solid (56 mg, 97%, m.p: 101–102 °C). Ethyl acetate/hexane (5:95, v/v) as the eluent. **^{1}H NMR (400 MHz, Acetone-d_6) δ** 11.35 (bs, 1H), 8.57 (ddd, *J* = 8.3, 1.1, 0.6 Hz, 1H), 8.22 (d, *J* = 7.5 Hz, 1H), 8.15 (s, 1H), 8.00 (d, *J* = 8.3 Hz, 1H), 7.69 – 7.40 (m, 9H), 7.26 (ddd, *J* = 8.0, 7.2, 1.1 Hz, 1H). **^{13}C{^{1}H} NMR (101 MHz, Acetone-d_6) δ** 142.7, 140.3, 136.1, 133.0, 131.6, 131.4, 129.2 (2C), 127.7, 126.4, 126.1, 125.7, 124.9, 122.7, 121.2, 120.7, 120.4, 118.5, 112.24, 112.19. **HRMS (ESI-TOF) *m/z*:** [M + H]$^+$ calcd for $C_{22}H_{16}N$, 294.1277; found, 294.1277.

Gram-scale synthesis of 2a. Compound **2a** (948 mg, 95%) was prepared by following general procedure J using **1a** (1g, 3.4 mmol) in CH_3CN (34 mL).

2-Methyl-5-(*p*-tolyl)-11*H*-benzo[*a*]carbazole (2b)

Off-white solid (61 mg, 95%, m.p: 145–146 °C). Ethyl acetate/hexane (5:95, v/v) as the eluent. **^{1}H NMR (400 MHz, Acetone-d_6) δ** 11.24 (bs, 1H), 8.33 (d, *J* = 0.7 Hz, 1H), 8.18 (dd, *J* = 7.8, 0.7 Hz, 1H), 8.04 (s, 1H), 7.91 (d, *J* = 8.6 Hz, 1H), 7.66 (d, *J* = 8.1 Hz, 1H), 7.48 – 7.37 (m, 3H), 7.36 – 7.29 (m, 3H), 7.24 (ddd, *J* = 8.0, 7.2, 1.0 Hz, 1H), 2.57 (s, 3H), 2.44 (s, 3H). **^{13}C{^{1}H} NMR (101 MHz, Acetone-d_6) δ** 140.2, 139.8, 137.0, 135.8, 132.8, 131.1, 129.8, 129.7, 127.9, 127.7, 125.5, 124.9, 122.8, 121.9, 120.5, 120.2, 120.0, 118.5, 112.1, 112.1, 21.8, 21.2. **HRMS (ESI-TOF) *m/z*:** [M + H]$^+$ calcd for $C_{24}H_{20}N$, 322.1590; found, 322.1592.

2-(*tert*-Butyl)-5-(4-(*tert*-butyl)phenyl)-11*H*-benzo[*a*]carbazole (2c)

White solid (72 mg, 89%, m.p: >300 °C). Ethyl acetate/hexane (5:95, v/v) as the eluent. **^{1}H NMR (400 MHz, DMSO-d_6) δ** 12.25 (bs, 1H), 8.58 (d, *J* = 1.5 Hz, 1H), 8.16 (d, *J* = 7.7 Hz, 1H), 8.06 (s, 1H), 7.87 (d, *J* = 8.9 Hz, 1H), 7.66 (d, *J* = 7.7 Hz, 1H), 7.58 (dd, *J* = 8.9, 1.5 Hz, 1H), 7.55 – 7.50 (m, AA' part of AA'BB' system, 2H), 7.49 – 7.43 (m, BB' part of AA'BB' system, 2H), 7.41 (t, *J* = 7.7 Hz, 1H), 7.20 (t, *J* = 7.7 Hz, 1H), 1.45 (s, 9H), 1.37 (s, 9H). **^{13}C{^{1}H} NMR (101 MHz, DMSO-d_6) δ** 149.1, 147.9, 138.9, 138.4, 135.1, 130.7, 129.9, 128.2, 126.3, 125.1, 124.6, 123.8, 123.4, 121.3, 119.9, 119.6, 119.1, 117.7, 116.9, 111.3, 34.9, 34.3, 31.3 (2C). **HRMS (ESI-TOF) *m/z*:** $C_{30}H_{32}N$ [M + H]$^+$ calcd for 406.2529; found, 406.2530.

5-([1,1'-Biphenyl]-4-yl)-2-phenyl-11*H*-benzo[*a*]carbazole (2d)

White solid (84 mg, 94%, m.p: 135–136 °C). Ethyl acetate/hexane (5:95, v/v) as the eluent. **^{1}H NMR (400 MHz, DMSO-d_6) δ** 12.41 (bs, 1H), 9.04 (d, *J* = 1.7 Hz, 1H), 8.24 (d, *J* = 8.2 Hz, 1H), 8.22 (s, 1H), 8.05 (d, *J* = 8.8 Hz, 1 H), 7.92 (dd, *J* = 7.2, 1.2 Hz, 2H), 7.86 (dd, *J* = 8.9, 1.9 Hz, 1H), 7.82 (d, *J* = 8.3 Hz, 2H), 7.76 (dd, *J* = 7.2, 1.2 Hz, 2H), 7.71 (d, *J* = 8.1 Hz, 1H), 7.64 (d, *J* = 8.2 Hz, 2H), 7.56 (t, *J* = 7.7 Hz, 2H), 7.50 (t, *J* = 7.7 Hz, 2H), 7.48 – 7.37 (m, 3H), 7.29 – 7.21 (m, 1H). **^{13}C{^{1}H} NMR (101 MHz, DMSO-d_6) δ** 130.6, 130.2, 130.1, 129.3, 129.0, 127.2, 125.7, 121.1, 120.7, 119.4, 119.3 (2C), 118.0, 117.8, 117.4, 117.2, 117.0 (2C), 115.2, 114.5, 113.6, 112.1, 110.9, 110.4, 110.3, 109.6, 107.6, 101.8. **HRMS (ESI-TOF) *m/z*:** [M + H]$^+$ calcd for $C_{34}H_{24}N$, 446.1903; found, 446.1906.

2-Methoxy-5-(4-methoxyphenyl)-11*H*-benzo[*a*]carbazole (2e)

White solid (65 mg, 92%, m.p: 197–198 °C). Ethyl acetate/hexane (5:95, v/v) as the eluent. **^{1}H NMR (400 MHz, DMSO-d_6) δ** 12.12 (bs, 1H), 8.17 (d, *J* = 7.7 Hz, 1H), 8.05 (d, *J* = 2.5 Hz, 1H), 7.94 (s, 1H), 7.82 (d, *J* = 9.2 Hz, 1H), 7.65 (d, *J* = 7.7 Hz, 1H), 7.47 – 7.38 (m, 3H), 7.21 (t, *J* = 7.7 Hz, 1H), 7.16 (dd, *J* = 9.2, 2.5 Hz, 1H), 7.13 – 7.05 (m, BB' part of AA'BB' system, 2H), 3.99 (s, 3H), 3.84 (s, 3H). **^{13}C{^{1}H} NMR (101 MHz, DMSO-d_6) δ** 158.3, 157.2, 138.9, 134.5, 133.6, 131.3, 130.9, 128.2, 125.2, 124.7, 123.4, 122.5, 120.0, 119.1, 117.8, 117.3, 116.8, 113.8, 111.3, 102.0, 55.5, 55.2. **HRMS (ESI-TOF) *m/z*:** [M + H]$^+$ calcd for $C_{24}H_{20}NO_2$, 354.1489; found, 354.1488.

2-Fluoro-5-(4-fluorophenyl)-11*H*-benzo[*a*]carbazole (2f)

White solid (58 mg, 89%, m.p: 198–199 ºC). Ethyl acetate/hexane (5:95, v/v) as the eluent. **^{1}H NMR (400 MHz, Acetone-*d*$_6$) δ** 11.32 (bs, 1H), 8.24 (dd, *J* = 10.2, 2.7 Hz, 1H), 8.20 (d, *J* = 7.9 Hz, 1H), 8.09 (s, 1H), 7.95 (dd, *J* = 9.3, 5.7 Hz, 1H), 7.66 (dd, *J* = 8.2, 0.8 Hz, 1H), 7.59 – 7.52 (m, 2H), 7.44 (ddd, *J* = 8.2, 7.1, 1.2 Hz, 1H), 7.35 – 7.23 (m, 4H). **^{13}C{^{1}H} NMR (101 MHz, Acetone-*d*$_6$) δ** 163.4 (d, *J* = 160.6 Hz), 160.9 (d, *J* = 160.7 Hz), 140.3, 138.5 (d, *J* = 3.2 Hz), 135.6 (d, *J* = 4.7 Hz), 133.0 (d, *J* = 7.9 Hz), 131.8, 130.4 (d, *J* = 9.1 Hz), 128.5, 126.1, 124.6, 123.4 (d, *J* = 9.2 Hz), 120.9, 120.6 (d, *J* = 1.7 Hz), 120.5, 119.2, 115.9 (d, *J* = 21.4 Hz), 115.4 (d, *J* = 24.5 Hz), 112.4, 106.6 (d, *J* = 22.1 Hz). **^{19}F NMR (376 MHz, CDCl$_3$) δ** -114.49, -115.70. **HRMS (ESI-TOF) *m/z*:** [M + H]$^+$ calcd for $C_{22}H_{14}F_2N$, 330.1089; found, 330.1085.

2-(Trifluoromethyl)-5-(4-(trifluoromethyl)phenyl)-11*H*-benzo[*a*]carbazole (2g)

Light-yellow solid (77 mg, 90%, m.p: 201–202 ºC). Ethyl acetate/hexane (5:95, v/v) as the eluent. **^{1}H NMR (400 MHz, Acetone-*d*$_6$) δ** 11.66 (bs, 1H), 8.99 (s, 1H), 8.34 (s, 1H), 8.22 (d, *J* = 7.7 Hz, 1H), 8.11 (d, *J* = 8.8 Hz, 1H), 7.92 – 7.85 (m, AA' part of AA'BB' system, 2H), 7.81 – 7.76 (m, BB' part of AA'BB' system, 2H), 7.74 (dd, *J* = 8.9, 1.7 Hz, 1H), 7.70 (d, *J* = 8.2 Hz, 1H), 7.47 (ddd, *J* = 8.2, 7.2, 1.1 Hz, 1H), 7.32 – 7.22 (m, 1H). **^{13}C{^{1}H} NMR (101 MHz, Acetone-*d*$_6$) δ** 145.4, 139.8, 135.9, 131.6, 130.3, 128.9 (q, *J* = 32.1 Hz), 127.9, 126.8 (q, *J* = 32.3 Hz), 125.8, 125.5 (q, *J* = 3.8 Hz), 125.0 (q, *J* = 274.0 Hz), 124.9 (q, *J* = 271.2 Hz), 123.7, 123.4, 120.9 (q, *J* = 3.1 Hz), 120.8, 120.3, 120.3, 120.1 (q, *J* = 4.1 Hz), 118.9, 111.8, 111.7. **^{19}F NMR (376 MHz, CDCl$_3$) δ** -61.98, -62.33. **HRMS (ESI-TOF) *m/z*:** [M + H]$^+$ calcd for $C_{24}H_{14}F_6N$, 430.1025; found, 430.1002.

1-(4-(2-Acetyl-11*H*-benzo[*a*]carbazol-5-yl)phenyl)ethan-1-one (2h)

White solid (65 mg, 95%, m.p: >300 ºC). Ethyl acetate/hexane (10:90, v/v) as the eluent. **^{1}H NMR (400 MHz, DMSO-*d*$_6$) δ** 12.64 (bs, 1H), 9.38 (bs, 1H), 8.37 (s, 1H), 8.26 (d, *J* = 7.7 Hz, 1H), 8.16 – 8.09 (m, AA' part of AA'BB' system, 2H), 8.03 – 7.94 (m, 2H), 7.78 – 7.65 (m, 3H), 7.48 (t, *J* = 7.5 Hz, 1H), 7.27 (t, *J* = 7.5 Hz, 1H), 2.78 (s, 3H), 2.67 (s, 3H). **^{13}C{^{1}H} NMR (101 MHz, DMSO-*d*$_6$) δ** 197.7 (2C), 145.6, 139.1, 136.2, 135.4, 133.4, 131.5, 130.6, 129.6, 128.5, 126.4, 125.4, 124.7, 123.5, 123.5, 123.1, 120.5, 120.3, 119.7, 117.7, 111.7, 26.8 (2C). **HRMS (ESI-TOF) *m/z*:** [M + H]$^+$ calcd for $C_{26}H_{20}NO_2$, 378.1489; found, 378.1487.

A mixture of 4-methyl-5-(*o*-tolyl)-11*H*-benzo[*a*]carbazole and 5-(*o*-tolyl)-11*H*-benzo[*a*]carbazole (2i-1+2i-2) (1:1.7)

Compounds **2i-1** and **2i-2** did not separate by chromatography and crystallization. Off-white solid (54 mg, 88%; 1:1.7). Ethyl acetate/hexane (5:95, v/v) as the eluent. **^{1}H NMR (400 MHz, CDCl$_3$) δ** 8.77 (bs, 1.7H), 8.73 (bs, 1H), 8.19 – 8.11 (m, 3.7H), 8.11 – 8.04 (m, 3.7H), 7.95 (s, 1H), 7.67 – 7.57 (m, 6.4H), 7.52 – 7.44 (m, 6.4H), 7.44 – 7.37 (m, 8.5H), 7.36 – 7.30 (m, 4.4H), 2.12 (s, 5.1H), 2.08 (s, 3H), 2.07 (s, 3H). **^{13}C{^{1}H} NMR (101 MHz, CDCl$_3$) δ** 145.6, 141.2, 138.9, 138.7, 137.6, 137.2, 137.0, 135.4, 134.6, 132.2, 131.2, 130.6, 129.9, 129.4, 129.3, 127.6, 127.2, 125.8, 125.5, 125.4, 125.29, 125.27, 125.14, 125.10, 124.3, 124.1, 122.1, 121.1, 120.8, 120.2, 120.12, 120.08, 120.0, 119.6, 118.0, 117.4, 111.24, 111.17, 24.6, 20.6, 20.4 (Six carbon signals overlapped). **HRMS (APCI-TOF) *m/z*:** [M + H]$^+$ calcd for $C_{24}H_{20}N$, 322.1590; found, 322.1577. **HRMS (APCI-TOF) *m/z*:** $C_{23}H_{18}N$ [M + H]$^+$ calcd for 308.1434; found, 308.1412.

1-Methyl-5-(*m*-tolyl)-11*H*-benzo[*a*]carbazole (2j–1)

2j–1 was eluted as the first fraction from the silica gel column (eluent: ethyl acetate/hexane; 1:99, v/v). Light-yellow oil (15 mg, 24%). **^{1}H NMR (400 MHz, Acetone-*d*$_6$) δ** 10.78 (bs, 1H), 8.23 (d, *J* = 7.9 Hz, 1H), 8.14 (s, 1H), 7.85 (d, *J* = 8.2 Hz, 1H), 7.78 (d, *J* = 8.2 Hz, 1H), 7.45 – 7.39 (m, 3H), 7.39 – 7.31 (m, 3H), 7.30 – 7.24 (m, 2H), 3.22 (s, 3H), 2.44 (s, 3H). **^{13}C{^{1}H} NMR (101 MHz, Acetone-*d*$_6$) δ** 143.2, 140.2, 138.5, 135.7, 133.8, 133.6, 132.9, 131.9, 128.9, 128.4, 128.4, 128.3, 126.1, 125.4, 125.3, 123.8,

122.9, 120.9, 120.4, 120.2, 119.4, 112.7, 24.2, 21.5. **HRMS (ESI-TOF) *m/z*:** $[M + H]^+$ calcd for $C_{24}H_{20}N$, 322.1590; found, 322.1591.

3-Methyl-5-(*m*-tolyl)-11*H*-benzo[*a*]carbazole (2j–2)

2j–2 was eluted as the second fraction from the silica gel column (eluent: ethyl acetate/hexane; 1:99, v/v). Light-yellow oil (42 mg, 66%). **^{1}H NMR (400 MHz, Acetone-d_6) δ** 11.29 (bs, 1H), 8.47 (d, *J* = 8.4 Hz, 1H), 8.18 (d, *J* = 7.8 Hz, 1H), 8.10 (s, 1H), 7.83 (s, 1H), 7.66 (d, *J* = 8.1 Hz, 1H), 7.49 – 7.35 (m, 5H), 7.30 – 7.18 (m, 2H), 2.44 (s, 6H). **^{13}C{^{1}H} NMR (101 MHz, Acetone-d_6) δ** 142.8, 140.1, 138.5, 136.1, 135.4, 132.6, 131.9, 131.8, 128.9, 128.4, 128.2, 128.1, 127.0, 125.3, 124.9, 122.6, 121.1, 120.7, 120.4, 120.2, 117.8, 112.0, 22.0, 21.6. **HRMS (ESI-TOF) *m/z*:** $[M + H]^+$ calcd for $C_{24}H_{20}N$, 322.1590; found, 322.1591.

5-(3,4-Dimethylphenyl)-1,2-dimethyl-11*H*-benzo[*a*]carbazole (2l–1)

2l–1 was eluted as the first fraction from the silica gel column (eluent: ethyl acetate/hexane; 1:99, v/v). White solid (18 mg, 26%, m.p: 184–185 °C). **^{1}H NMR (400 MHz, Acetone-d_6) δ** 10.78 (bs, 1H), 8.20 (d, *J* = 7.5 Hz, 1H), 8.05 (s, 1H), 7.77 (d, *J* = 5.5 Hz, 1H), 7.75 (d, *J* = 5.5 Hz, 1H), 7.44 – 7.37 (m, 1H), 7.33 – 7.21 (m, 5H), 3.12 (s, 3H), 2.53 (s, 3H), 2.36 (s, 3H), 2.35 (s, 3H). **^{13}C{^{1}H} NMR (101 MHz, Acetone-d_6) δ** 140.8, 140.0, 136.9, 135.6, 135.5, 133.9, 133.8, 132.4, 131.9, 130.9, 130.2, 128.7, 128.6, 125.6, 125.3, 123.9, 123.7, 120.2, 120.1, 119.9, 119.7, 112.6, 21.1, 19.9, 19.5, 18.8. **HRMS (ESI-TOF) *m/z*:** $[M + H]^+$ calcd for $C_{26}H_{24}N$, 350.1903; found, 350.1904.

5-(3,4-Dimethylphenyl)-2,3-dimethyl-11*H*-benzo[*a*]carbazole (2l–2)

2l-1 was eluted as the first fraction from the silica gel column (eluent: ethyl acetate/hexane; 1:99, v/v). White solid (45 mg, 65%, m.p: 231–232 °C). **^{1}H NMR (400 MHz, Acetone-d_6) δ** 11.16 (bs, 1H), 8.29 (s, 1H), 8.15 (d, *J* = 8.0 Hz, 1H), 8.00 (s, 1H), 7.80 (s, 1H), 7.64 (d, *J* = 8.0 Hz, 1H), 7.41 – 7.36 (m, 1H), 7.35 (s, 1H), 7.29 – 7.26 (m, 2H), 7.25 – 7.20 (m, 1H), 2.49 (s, 3H), 2.36 (s, 9H). **^{13}C{^{1}H} NMR (101 MHz, Acetone-d_6) δ** 140.5, 140.1, 137.0, 135.7, 135.6, 135.5, 135.2, 132.5, 132.4, 130.6, 130.2, 128.7, 127.7, 125.2, 125.1, 122.5, 121.4, 120.4, 120.1, 120.0, 117.9, 112.0, 20.5, 20.3, 19.9, 19.5. **HRMS (ESI-TOF) *m/z*:** $[M + H]^+$ calcd for $C_{26}H_{24}N$, 350.1903; found, 350.1905.

4-(Thiophen-2-yl)-10*H*-thieno[3,2-*a*]carbazole (2m)

Light-yellow solid (58 mg, 95%, m.p: 146–147 °C). Ethyl acetate/hexane (5:95, v/v) as the eluent. **^{1}H NMR (400 MHz, $CDCl_3$) δ** 8.28 (bs, 1H), 8.21 (s, 1H), 8.13 (d, *J* = 7.8 Hz, 1H), 7.64 (dd, *J* = 3.5, 0.8 Hz, 1H), 7.51 – 7.38 (m, 5H), 7.33 (ddd, *J* = 8.0, 5.8, 2.3 Hz, 1H), 7.24 (dd, *J* = 5.1, 3.6 Hz, 1H). **^{13}C{^{1}H} NMR (101 MHz, $CDCl_3$) δ** 143.7, 138.8, 137.4, 133.9, 127.8, 126.1, 125.4, 125.0, 124.8, 124.5, 123.9, 121.7, 120.2 (2C), 119.7, 119.6, 117.2, 111.2. **HRMS (ESI-TOF) *m/z*:** $[M + H]^+$ calcd for $C_{18}H_{12}NS_2$, 306.0406; found, 306.0409.

5-Phenyl-11-((trifluoromethyl)sulfonyl)-11*H*-benzo[*a*]carbazole (2n)

Yellow oil (66 mg, 78%). Ethyl acetate/hexane (5:95, v/v) as the eluent. **^{1}H NMR (400 MHz, $CDCl_3$) δ** 8.00 (d, *J* = 8.4 Hz, 1H), 7.97 (d, *J* = 7.6 Hz, 1H), 7.86 (s, 1H), 7.71 (d, *J* = 8.0 Hz, 1H), 7.63 – 7.58 (m, 1H), 7.58 – 7.51 (m, 4H), 7.50 – 7.45 (m, 2H), 7.45 – 7.42 (m, 1H), 7.40 – 7.34 (m, 1H), 6.89 (s, 1H). **^{13}C{^{1}H} NMR (101 MHz, $CDCl_3$) δ** 142.07, 139.89, 134.72, 133.94, 133.73, 131.93, 131.36, 131.14, 130.09, 129.20, 128.61, 128.57, 127.97, 127.83, 127.71, 127.21, 127.05, 126.78, 126.30, 119.66 (q, *J* = 322.6 Hz). 121.74. **^{19}F NMR (376 MHz, $CDCl_3$) δ** -76.17. **HRMS (APCI-TOF) *m/z*:** $[M]^+$ calcd for $C_{23}H_{14}F_3NO_2S$, 425.0692; found, 425.0688.

7-Methyl-5-phenyl-11*H*-benzo[*a*]carbazole (2o)

Light-yellow solid (53 mg, 86%, m.p: 151–152 °C). Ethyl acetate/hexane (5:95, v/v) as the eluent. **^{1}H NMR (400 MHz, Acetone-d_6) δ** 11.40 (bs, 1H), 8.58 (d, *J* = 8.2 Hz, 1H), 8.23 (s, 1H), 8.00 (d, *J* = 8.5

Hz, 1H), 7.66 – 7.43 (m, 8H), 7.31 (t, J = 7.3 Hz, 1H), 7.03 (d, J = 7.2 Hz, 1H), 2.91 (s, 3H). **$^{13}C\{^{1}H\}$ NMR (101 MHz, Acetone-d_6) δ** 143.0, 140.3, 136.1, 133.1, 132.8, 131.4, 131.0, 129.2 (2C), 127.7, 127.6, 126.3, 126.1, 125.6, 123.3, 122.7, 122.4, 121.9, 119.1, 110.0, 21.0. **HRMS (ESI-TOF) *m/z*:** [M + H]$^+$ calcd for $C_{23}H_{18}N$, 308.1434; found, 308.1434.

7-Chloro-5-phenyl-11*H*-benzo[*a*]carbazole (2p)

Brown solid (62 mg, 95%, m.p: 184–185 °C). Ethyl acetate/hexane (5:95, v/v) as the eluent. **^{1}H NMR (400 MHz, Acetone-d_6) δ** 11.67 (bs, 1H), 8.59 – 8.56 (m, 1H), 8.55 (s, 1H), 7.99 (d, J = 8.4 Hz, 1H), 7.69 – 7.62 (m, 2H), 7.61 – 7.52 (m, 5H), 7.50 – 7.44 (m, 1H), 7.39 (t, J = 7.4 Hz, 1H), 7.26 (dd, J = 7.7, 0.7 Hz, 1H). **$^{13}C\{^{1}H\}$ NMR (101 MHz, Acetone-d_6) δ** 142.6, 141.3, 136.5, 133.3, 131.6, 131.2, 129.2, 128.1, 127.8, 127.7, 126.7, 126.6, 126.2, 122.7, 122.6, 122.2, 121.0, 117.4, 111.1 (One carbon signal overlapped). **HRMS (ESI-TOF) *m/z*:** [M + H]$^+$ calcd for $C_{22}H_{15}ClN$, 328.0888; found, 328.0887.

5,8-Diphenyl-11*H*-benzo[*a*]carbazole (2q)

Off-white solid (70 mg, 95%, m.p: 101–102 °C). Ethyl acetate/hexane (5:95, v/v) as the eluent. **^{1}H NMR (400 MHz, Acetone-d_6) δ** 11.41 (bs, 1H), 8.58 (d, J = 8.9 Hz, 1H), 8.56 (s, 1H), 8.27 (s, 1H), 8.02 (d, J = 8.5 Hz, 1H), 7.81 (dd, J = 8.3, 1.1 Hz, 2H), 7.74 (d, J = 1.1 Hz, 2H), 7.67 – 7.59 (m, 3H), 7.57 – 7.50 (m, 3H), 7.50 – 7.44 (m, 3H), 7.32 (t, J = 7.9 Hz, 1H). **$^{13}C\{^{1}H\}$ NMR (101 MHz, Acetone-d_6) δ** 143.0, 142.7, 139.8, 136.7, 133.7, 133.1, 131.7, 131.3, 129.6, 129.2, 127.9 (2C), 127.8, 127.2, 126.4, 126.2, 125.6, 125.1, 122.73, 122.69, 121.4, 119.0, 118.8, 112.6. **HRMS (ESI-TOF) *m/z*:** [M + H]$^+$ calcd for $C_{28}H_{20}N$, 370.1590; found, 370.1593.

8-(Benzyloxy)-5-phenyl-11*H*-benzo[*a*]carbazole (2r)

Light-yellow oil (70 mg, 88%). Ethyl acetate/hexane (5:95, v/v) as the eluent. **^{1}H NMR (400 MHz, Acetone-d_6) δ** 11.22 (bs, 1H), 8.54 (d, J = 8.1 Hz, 1H), 8.12 (s, 1H), 7.99 (d, J = 8.4 Hz, 1H), 7.90 (d, J = 2.3 Hz, 1H), 7.65 – 7.42 (m, 10H), 7.38 (t, J = 7.4 Hz, 2H), 7.31 (d, J = 7.0 Hz, 1H), 7.16 (dd, J = 8.7, 2.3 Hz, 1H), 5.23 (s, 2H). **$^{13}C\{^{1}H\}$ NMR (101 MHz, Acetone-d_6) δ** 154.2, 142.7, 138.9, 136.7, 135.2, 132.4, 131.5, 131.2, 130.1, 129.2, 129.1, 128.4, 128.3, 127.6, 126.2, 126.0, 125.2, 122.7, 122.6, 121.2, 118.4, 116.0, 112.9, 104.4, 71.2. **HRMS (ESI-TOF) *m/z*:** [M + H]$^+$ calcd for $C_{29}H_{22}NO$, 400.1696; found, 400.1698.

9-Methyl-5-phenyl-11*H*-benzo[*a*]carbazole (2t)

White solid (57 mg, 93%, m.p: 188–189 °C). Ethyl acetate/hexane (5:95, v/v) as the eluent. **^{1}H NMR (400 MHz, Acetone-d_6) δ** 11.20 (bs, 1H), 8.52 (d, J = 8.2 Hz, 1H), 8.10 (s, 1H), 8.07 (d, J = 8.0 Hz, 1H), 7.98 (d, J = 8.5 Hz, 1H), 7.64 – 7.51 (m, 5H), 7.50 – 7.43 (m, 3H), 7.09 (d, J = 8.0 Hz, 1H), 2.52 (s, 3H). **$^{13}C\{^{1}H\}$ NMR (101 MHz, Acetone-d_6) δ** 142.8, 140.8, 135.9, 135.5, 132.8, 131.33, 131.29, 130.1, 129.2, 127.7, 126.2, 125.8, 122.73, 122.68, 122.56, 122.1, 121.1, 120.4, 118.6, 112.2, 22.1. **HRMS (ESI-TOF) *m/z*:** [M + H]$^+$ calcd for $C_{23}H_{18}N$, 308.1434; found, 308.1434.

9-Methoxy-5-phenyl-11*H*-benzo[*a*]carbazole (2u)

Off-white solid (58 mg, 90%, m.p: 201–202 °C). Ethyl acetate/hexane (5:95, v/v) as the eluent. **^{1}H NMR (400 MHz, Acetone-d_6) δ** 11.18 (bs, 1H), 8.49 (d, J = 7.9 Hz, 1H), 8.07 (s, 1H), 8.07 (d, J = 8.6 Hz, 1H), 7.97 (d, J = 8.5 Hz, 1H), 7.63 – 7.51 (m, 5H), 7.48 – 7.42 (m, 2H), 7.17 (d, J = 2.3 Hz, 1H), 6.90 (dd, J = 8.6, 2.3 Hz, 1H), 3.89 (s, 3H). **$^{13}C\{^{1}H\}$ NMR (101 MHz, Acetone-d_6) δ** 159.7, 142.9, 141.6, 135.7, 133.0, 131.4, 130.8, 129.2 (2C), 127.7, 126.3, 125.6, 122.6, 122.4, 121.4, 120.9, 118.9, 118.8, 109.9, 95.8, 55.8. **HRMS (ESI-TOF) *m/z*:** [M + H]$^+$ calcd for $C_{23}H_{18}NO$, 324.1383; found, 324.1389.

B) Preparation of 4a–4p

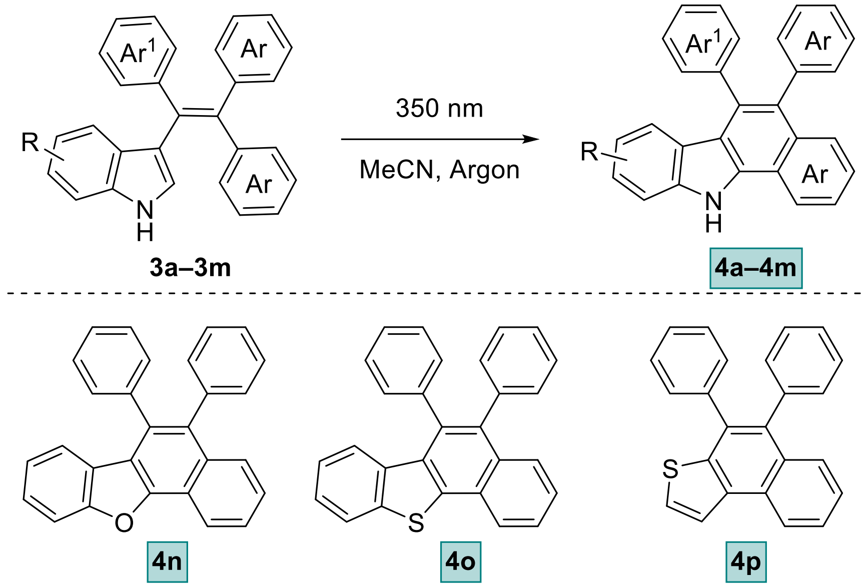

5,6-Diphenyl-11*H*-benzo[*a*]carbazole (4a)[12]

Off-white solid (70 mg, 95%, m.p: 242–243 °C). Ethyl acetate/hexane (5:95, v/v) as the eluent. **^{1}H NMR (400 MHz, CDCl$_3$) δ** 8.86 (bs, 1H), 8.18 (d, *J* = 8.1 Hz, 1H), 7.68 (d, *J* = 8.4 Hz, 1H), 7.61 – 7.56 (m, 1H), 7.54 (d, *J* = 8.1 Hz, 1H), 7.47 – 7.40 (m, 1H), 7.38 – 7.09 (m, 11H) 6.95 (t, *J* = 7.5 Hz, 1H), 6.75 (d, *J* = 8.0 Hz, 1H). **^{13}C{^{1}H} NMR (101 MHz, CDCl$_3$) δ** 140.2, 139.4, 138.9, 135.0, 134.5, 132.0, 131.9, 131.1, 130.4, 128.3, 128.0, 127.6, 126.9, 126.4, 125.6, 125.4, 124.8, 124.5, 122.1, 120.5, 119.8, 117.5, 110.9. (1 carbon signal overlapped). **HRMS (APCI-TOF) *m/z*:** [M + H]$^+$ calcd for $C_{28}H_{20}N$, 370.1590; found 370.1592.

Gram-scale reaction for 4a. Compound **4a** (906 mg, 91%) was prepared by following general procedure J using **3a** (1g, 2.7 mmol) in CH_3CN (14 mL).

11-Ethyl-5,6-diphenyl-11*H*-benzo[*a*]carbazole (4b)

Yellow solid (76 mg, 96%, m.p: 248–249 °C). Ethyl acetate/hexane (5:95, v/v) as the eluent. **^{1}H NMR (400 MHz, CDCl$_3$) δ** 8.64 (d, *J* = 8.5 Hz, 1H), 7.72 (d, *J* = 8.5 Hz, 1H), 7.62 (t, *J* = 7.6 Hz, 1H), 7.55 (d, *J* = 8.3 Hz, 1H), 7.42 (t, *J* = 7.6 Hz, 1H), 7.38 (t, *J* = 7.6 Hz, 1H), 7.32 – 7.16 (m, 10H), 6.93 (t, *J* = 7.6 Hz, 1H), 6.64 (d, *J* = 8.0 Hz, 1H), 4.93 (q, *J* = 7.1 Hz, 2H), 1.75 (t, *J* = 7.1 Hz, 3H). **^{13}C{^{1}H} NMR (101 MHz, CDCl$_3$) δ** 140.6, 140.5, 139.8, 135.2, 134.2, 132.9, 132.0, 131.2, 130.4, 128.7, 128.1, 127.6, 126.9, 126.4, 125.2, 124.9, 124.6, 123.4, 122.3, 122.0, 121.6, 119.5, 118.1, 108.7, 41.0, 15.3. **HRMS (ESI-TOF) *m/z*:** [M + H]$^+$ calcd for $C_{30}H_{24}N$, 398.1903; found 398.1903.

11-Hexyl-5,6-diphenyl-11*H*-benzo[*a*]carbazole (4c)

Yellow solid (87 mg, 96%, m.p: 160–161 °C). Ethyl acetate/hexane (5:95, v/v) as the eluent. **^{1}H NMR (400 MHz, CDCl$_3$) δ** 8.61 (d, *J* = 8.5 Hz, 1H), 7.72 (d, *J* = 8.5 Hz, 1H), 7.66 – 7.58 (m, 1H), 7.54 (d, *J* = 8.3 Hz, 1H), 7.47 – 7.40 (m, 1H), 7.40 – 7.34 (m, 1H), 7.34 – 7.09 (m, 10H), 6.92 (t, *J* = 7.5 Hz, 1H), 6.63 (d, *J* = 8.0 Hz, 1H), 4.83 (t, *J* = 7.5 Hz, 2H), 2.16 (p, *J* = 7.5 Hz, 2H), 1.62 (p, *J* = 7.5 Hz, 2H), 1.52 – 1.30 (m, 4H), 0.94 (t, *J* = 7.5 Hz, 3H). **^{13}C{^{1}H} NMR (101 MHz, CDCl$_3$) δ** 141.0, 140.5, 139.8, 135.2, 134.2, 132.9, 132.0, 131.2, 130.4, 128.7, 128.1, 127.6, 126.9, 126.4, 125.1, 124.9, 124.6, 123.3, 122.2, 122.0, 121.7, 119.4, 118.1, 109.0, 46.4, 31.8, 30.1, 27.0, 22.8, 14.2. **HRMS (ESI-TOF) *m/z*:** [M + H]$^+$ calcd for $C_{34}H_{32}N$, 454.2529; found 454.2529.

6-(4-Methoxyphenyl)-5-phenyl-11*H*-benzo[*a*]carbazole (4d)

Off-white solid (72 mg, 91%, m.p: 275–276 °C). Ethyl acetate/hexane (5:95, v/v) as the eluent. **^{1}H NMR (400 MHz, DMSO-*d*$_6$) δ** 12.38 (bs, 1H), 8.64 (d, *J* = 7.9 Hz, 1H), 7.70 – 7.58 (m, BB' part of AA'BB' system, 2H), 7.54 – 7.41 (m, 2H), 7.37 – 7.20 (m, =CH, 5H), 7.21 – 7.09 (m, =CH, 4H), 6.94 – 6.78 (m, 2H), 6.63 (d, *J* = 7.7 Hz, 1H), 3.75 (s, 3H). **^{13}C{^{1}H} NMR (101 MHz, DMSO-*d*$_6$) δ** 157.8, 139.3, 139.1, 134.6, 134.1, 132.1, 131.5, 131.1, 130.9, 129.7, 127.5, 126.9, 126.3, 125.5, 125.1, 124.2, 123.4, 121.8, 120.9, 120.4, 118.7, 116.4, 113.2, 111.2, 54.9. **HRMS (ESI-TOF) *m/z*:** [M + H]$^+$ calcd for $C_{29}H_{22}NO$, 400.1696; found 400.1702.

11-Ethyl-6-(4-methoxyphenyl)-5-phenyl-11*H*-benzo[a]carbazole (4e)

Light-yellow solid (83 mg, 98%, m.p: 295–296 °C). Ethyl acetate/hexane (5:95, v/v) as the eluent. **^{1}H NMR (400 MHz, CDCl$_3$) δ** 8.64 (d, *J* = 8.5 Hz, 1H), 7.70 (d, *J* = 8.4 Hz, 1H), 7.67 – 7.58 (m, 1H), 7.55 (d, *J* = 8.3 Hz, 1H), 7.46 – 7.35 (m, 2H), 7.30 – 7.11 (m, 7H), 6.96 (t, *J* = 7.5 Hz, 1H), 6.83 (d, *J* = 8.6 Hz, 2H), 6.75 (d, *J* = 8.0 Hz, 1H), 4.93 (q, *J* = 7.2 Hz, 2H), 3.82 (s, 3H), 1.75 (t, *J* = 7.2 Hz, 3H). **^{13}C{^{1}H} NMR (101 MHz, CDCl$_3$) δ** 158.4, 140.6, 140.0, 134.91, 134.89, 134.2, 133.0, 132.8, 132.0, 131.4, 128.7, 127.7, 126.3, 125.1, 124.9, 124.6, 123.6, 122.4, 122.0, 121.6, 119.5, 118.5, 113.5, 108.7, 55.3, 41.0, 29.9, 15.3. **HRMS (ESI-TOF) *m/z*:** [M + H]$^+$ calcd for $C_{31}H_{26}NO$, 428.2009; found 428.2010.

11-Hexyl-6-(4-methoxyphenyl)-5-phenyl-11*H*-benzo[*a*]carbazole (4f)

Yellow solid (94 mg, 98%, m.p: 181–182 °C). Ethyl acetate/hexane (5:95, v/v) as the eluent. **^{1}H NMR (400 MHz, CDCl$_3$) δ** 8.60 (d, *J* = 8.4 Hz, 1H), 7.70 (dd, *J* = 8.4, 0.6 Hz, 1H), 7.64 – 7.57 (m, 1H), 7.54 (d, *J* = 8.4 Hz, 1H), 7.46 – 7.35 (m, 2H), 7.29 – 7.23 (m, 2H), 7.23 – 7.17 (m, 3H), 7.17 – 7.12 (m, AA' part of AA'BB' system, 2H), 6.95 (t, *J* = 7.4 Hz, 1H), 6.88 – 6.80 (m, BB' part of AA'BB' system, 2H), 6.74 (d, *J* = 8.0 Hz, 1H), 4.89 – 4.77 (m, 2H), 3.82 (s, 3H), 2.21 – 2.11 (m, 2H), 1.66 – 1.57 (m, 2H), 1.50 – 1.35 (m, 4H), 0.94 (t, J = 7.1 Hz, 3H). **^{13}C{^{1}H} NMR (101 MHz, CDCl$_3$) δ** 158.4, 141.0, 140.0, 134.87, 134.2, 133.0, 132.8, 132.0, 131.6, 131.4, 128.7, 127.7, 126.3, 125.0, 124.8, 124.5, 123.4, 122.3, 121.9, 121.6, 119.4, 118.5, 113.5, 109.0, 55.3, 46.4, 31.8, 30.1, 27.0, 22.8, 14.2. **HRMS (ESI-TOF) *m/z*:** [M + H]$^+$ calcd for $C_{35}H_{34}NO$, 484.2635; found 484.2640.

***N*-Isopropyl-4-(5-phenyl-11*H*-benzo[*a*]carbazole-6-yl)aniline (4h)**

Yellow solid (83 mg, 98%, m.p: 247–248 °C). Ethyl acetate/hexane (5:95, v/v) as the eluent. **^{1}H NMR (400 MHz, Acetone-*d*$_6$) δ** 11.41 (bs, 1H), 8.56 (d, *J* = 7.8 Hz, 1H), 7.64 – 7.56 (m, 2H), 7.55 (d, *J* = 8.1 Hz, 1H), 7.42 (ddd, *J* = 8.1, 7.0, 1.1 Hz, 1H), 7.33 – 7.24 (m, 3H), 7.24 – 7.17 (m, 3H), 7.08 – 6.95 (m, 3H), 6.92 (ddd, *J* = 8.1, 7.0, 1.1 Hz, 1H), 6.58 – 6.42 (m, BB' part of AA'BB' system, 2H), 4.60 (bs, 1H), 3.61 (hep, *J* = 6.3 Hz, 1H), 1.19 (d, *J* = 6.3 Hz, 6H). **^{13}C{^{1}H} NMR (101 MHz, Acetone-*d*$_6$) δ** 147.7, 141.0, 140.3, 136.4, 135.7, 132.7, 132.7, 131.6, 131.4, 128.6, 128.3, 128.2, 126.8, 126.0, 125.6, 125.2, 125.0, 122.8, 122.1, 121.5, 119.6, 118.6, 113.1, 111.8, 44.5, 22.9. **HRMS (ESI-TOF) *m/z*:** [M + H]$^+$ calcd for $C_{31}H_{27}N_2$, 427.2169; found 427.2161.

6-(1*H*-Indol-3-yl)-5-phenyl-11*H*-benzo[*a*]carbazole (4i)

Off-white solid (75 mg, 92%, m.p: 218–219 °C). Ethyl acetate/hexane (5:95, v/v) as the eluent. **^{1}H NMR (400 MHz, CDCl$_3$) δ** 8.94 (bs, 1H), 8.19 (d, *J* = 8.1 Hz, CH, 1H), 7.94 (bs, 1H), 7.75 (d, *J* = 8.4 Hz, 1H), 7.60 (t, *J* = 7.4 Hz, 1H), 7.55 – 7.48 (m, 1H), 7.48 – 7.38 (m, 2H), 7.37 – 7.26 (m, 4H), 7.23 – 7.17 (m, 2H), 7.15 – 7.06 (m, 2H), 7.00 (t, *J* = 7.4 Hz, 1H), 6.90 – 6.76 (m, 3H). **^{13}C{^{1}H} NMR (101 MHz, CDCl$_3$) δ** 140.5, 138.9, 135.6, 134.6, 133.4, 132.1, 132.0, 130.9, 128.5, 128.4, 127.9, 127.4, 127.1, 126.3, 125.4, 125.3, 124.6, 124.5, 123.6, 122.5, 122.0, 120.6, 120.5, 119.9, 119.7, 118.7, 115.6, 111.0, 110.7. (1 carbon signal overlapped). **HRMS (ESI-TOF) *m/z*:** [M + H]$^+$ calcd for $C_{30}H_{21}N_2$, 409.1699; found 409.1696.

5,6,8-Triphenyl-11*H*-benzo[*a*]carbazole (4j)

White solid (84 mg, 94%, m.p: >300 °C). Ethyl acetate/hexane (5:95, v/v) as the eluent. **^{1}H NMR (400 MHz, Acetone-*d*$_6$) δ** 11.53 (bs, 1H), 8.60 (ddd, *J* = 8.4, 1.2, 0.7 Hz, 1H), 7.74 – 7.65 (m, 1H), 7.65 – 7.61 (m, 2H), 7.59 (ddd, *J* = 8.4, 1.2, 0.7 Hz, 1H), 7.51 – 7.43 (m, 1H), 7.40 – 7.28 (m, 9H), 7.28 – 7.17 (m, 6H), 6.95 – 6.69 (m, 1H). **^{13}C{^{1}H} NMR (101 MHz, Acetone-*d*$_6$) δ** 142.8, 141.4, 140.4, 139.9, 136.3, 135.7, 132.8, 132.6, 131.1, 129.4, 128.8, 128.42, 128.36, 127.6, 127.4, 127.2, 127.0, 126.3, 126.1, 125.4, 124.3, 122.3, 121.8, 120.7, 118.1, 112.3. (2 carbon signal overlapped). **HRMS (ESI-TOF) *m/z*:** [M + H]$^+$ calcd for $C_{34}H_{24}N$, 446.1903; found 446.1905.

5,6-Diphenyl-11*H*-benzo[*a*]carbazole-8-carbaldehyde (4k)

White solid (76 mg, 96%, m.p: >300 °C). Ethyl acetate/hexane (5:95, v/v) as the eluent. **^{1}H NMR (400 MHz, DMSO-*d*$_6$) δ** 12.96 (bs, 1H), 9.62 (s, CHO, 1H), 8.67 (d, *J* = 8.2 Hz, 1H), 7.85 (d, *J* = 8.5 Hz, A part of AB system, 1H), 7.79 (d, *J* = 8.5 Hz, B part of AB system, 1H), 7.72 (t, *J* = 7.5 Hz, 1H), 7.53 (t, *J* = 7.5 Hz, 1H), 7.49 (t, *J* = 8.5 Hz, 1H), 7.38 – 7.32 (m, 3H), 7.30 – 7.21 (m, 5H), 7.19 (d, *J* = 7.7 Hz, 2H), 7.03 (s, 1H). **^{13}C{^{1}H} NMR (101 MHz, DMSO-*d*$_6$) δ** 191.4, 142.8, 139.3, 138.7, 135.8, 134.2, 131.5, 131.4, 130.7, 129.7, 128.2, 128.1, 127.6, 127.2, 127.1, 126.5, 126.2, 125.7, 125.4, 124.5, 123.3, 121.9, 120.5, 116.6, 111.9. **HRMS (ESI-TOF) *m/z*:** [M + H]$^+$ calcd for $C_{29}H_{20}NO$, 398.1539; found 398.1544.

10-Fluoro-5,6-diphenyl-11*H*-benzo[*a*]carbazole (4l)

White solid (72 mg, 93%, m.p: 218–219 °C). Ethyl acetate/hexane (5:95, v/v) as the eluent. **^{1}H NMR (400 MHz, CDCl$_3$) δ** 8.93 (bs, 1H), 8.12 (d, *J* = 8.0 Hz, 1H), 7.59 (d, *J* = 8.5 Hz, 1H), 7.57 – 7.47 (m, 1H), 7.42 – 7.31 (m, 1H), 7.25 – 7.07 (m, 10H), 6.96 (dd, *J* = 10.8, 8.0 Hz, 1H), 6.75 (td, *J* = 8.0, 4.9 Hz, 1H), 6.39 (d, *J* = 8.0 Hz, 1H). **^{13}C{^{1}H} NMR (101 MHz, CDCl$_3$) δ** 149.3 (d, *J* = 242.9 Hz), 139.9, 139.2, 134.8 (d, *J* = 6.1 Hz), 132.2, 131.9, 131.7, 130.3, 128.3, 128.0, 127.9 (d, *J* = 4.8 Hz), 127.6, 127.1, 127.03, 126.98, 126.5, 126.0, 125.7, 120.6, 120.5 (d, *J* = 5.9 Hz), 119.9 (d, *J* = 5.9 Hz), 117.8 (d, *J* = 3.5 Hz), 117.7 (d, *J* = 2.5 Hz), 109.7 (d, *J* = 15.7 Hz). **^{19}F NMR (376 MHz, CDCl$_3$) δ** -135.30. **HRMS (ESI-TOF) *m/z*:** [M + H]$^+$ calcd for $C_{28}H_{19}FN$, 388.1496; found 388.1495.

5,6-Diphenyl-13*H*-dibenzo[*a,i*]carbazole (4m)

Off-white solid (79 mg, 95%, m.p: 242–243 °C). Ethyl acetate/hexane (5:95, v/v) as the eluent. **^{1}H NMR (400 MHz, Acetone-*d*$_6$) δ** 12.13 (bs, 1H), 8.79 (d, *J* = 8.2 Hz, 1H), 8.70 (d, *J* = 8.3 Hz, 1H), 7.93 (d, *J* = 8.0 Hz, 1H), 7.70 – 7.59 (m, 2H), 7.57 (d, *J* = 8.4 Hz, 1H), 7.53 – 7.41 (m, 2H), 7.37 – 7.28 (m, 6H), 7.28 – 7.17 (m, 5H), 6.73 (d, *J* = 8.8 Hz, 1H). **^{13}C{^{1}H} NMR (101 MHz, Acetone-*d*$_6$) δ** 141.4, 140.5, 135.6, 135.3, 134.6, 132.6, 132.5, 132.2, 131.8, 131.2, 129.3, 128.7, 128.4, 128.3, 127.6, 127.1, 126.3, 126.2, 125.9, 125.8, 122.7, 122.22, 122.19, 121.5, 120.5, 120.3, 119.0. (1 carbon signal overlapped). **HRMS (ESI-TOF) *m/z*:** [M + H]$^+$ calcd for $C_{32}H_{22}N$, 420.1747; found 420.1746.

5,6-Diphenylnaphtho[1,2-*b*]benzofuran (4n)

White solid (66 mg, 90%, m.p: 228–229 °C). Ethyl acetate/hexane (5:95, v/v) as the eluent. **^{1}H NMR (400 MHz, CDCl$_3$) δ** 8.57 (d, *J* = 8.2 Hz, 1H), 7.78 – 7.59 (m, 3H), 7.53 – 7.46 (m, 1H), 7.43 – 7.36 (m, 1H), 7.36 – 7.18 (m, 10H), 7.12 – 7.06 (m, 1H), 6.82 (d, *J* = 7.4 Hz, 1H). **^{13}C{^{1}H} NMR (101 MHz, CDCl$_3$) δ** 156.3, 151.4, 139.1, 138.7, 134.0, 134.0, 132.6, 131.8, 130.3, 128.1, 127.8, 127.7, 127.2, 126.8, 126.5, 126.2, 126.1, 125.3, 122.7, 122.1, 121.0, 120.8, 118.4, 111.7. **HRMS (ESI-TOF) *m/z*:** [M + H]$^+$ calcd for $C_{28}H_{19}O$, 371.1430; found 371.1432.

5,6-Diphenylbenzo[*b*]naphtho[2,1-*d*]thiophene (4o)

Off-white solid (70 mg, 91%, m.p: 213–214 °C). Ethyl acetate/hexane (5:95, v/v) as the eluent. **^{1}H NMR (400 MHz, CDCl$_3$) δ** 8.27 (dd, *J* = 8.2, 0.6 Hz, 1H), 7.94 (dd, *J* = 8.0, 0.6 Hz, 1H), 7.67 – 7.55 (m, 2H),

7.54 – 7.43 (m, 1H), 7.40 – 7.15 (m, 11H), 7.11 – 6.98 (m, 1H), 6.61 (dd, *J* = 8.4, 0.5 Hz, 1H). **^{13}C{^{1}H} NMR (101 MHz, CDCl$_3$) δ** 140.0, 139.4, 138.9, 137.6, 137.3, 136.9, 135.9, 131.5, 131.4, 131.1, 130.4, 128.3, 128.2, 128.1, 127.6, 127.2, 126.7, 126.6, 126.5, 125.6, 125.0, 124.6, 124.1, 122.8. **HRMS (ESI-TOF) *m/z*:** [M + H]$^+$ calcd for $C_{28}H_{19}S$, 387.1202; found 387.1201.

4,5-Diphenylnaphtho[1,2-b]thiophene (4p)

Off-white solid (64 mg, 95%, m.p: 193–194 °C). Ethyl acetate/hexane (5:95, v/v) as the eluent. **^{1}H NMR (400 MHz, CDCl$_3$) δ** 8.34 (d, *J* = 8.2 Hz, 1H), 8.00 (d, *J* = 5.5 Hz, 1H), 7.60 (d, *J* = 8.4 Hz, 1H), 7.53 (t, *J* = 7.4 Hz, 1H), 7.49 (d, *J* = 5.5 Hz, 1H), 7.41 – 7.27 (m, 1H), 7.24 – 7.10 (m, 10H). **^{13}C{^{1}H} NMR (101 MHz, CDCl$_3$) δ** 140.03, 139.96, 139.0, 135.5, 135.0, 133.9, 131.7, 131.4, 130.2, 128.9, 128.1, 128.0, 127.8, 127.4, 126.84, 126.76, 126.3, 125.6, 123.7, 122.4. **HRMS (ESI-TOF) *m/z*:** [M + H]$^+$ calcd for $C_{24}H_{17}S$, 337.1045; found 337.1045.

9,18-Dihydrobenzo[*a*]benzo[1,2]carbazolo[3,4-*c*]carbazole (5)

To a solution of 6-(1*H*-indol-3-yl)-5-phenyl-11H-benzo[*a*]carbazole (**4i**) (61 mg, 0.15 mmol) in THF (4 mL) was added I_2 (38 mg, 0.15 mmol). The mixture was stirred under UV light (350 nm) in a rayonette apparatus under an oxygen atmosphere for 12 hours. At the end of this period, 10 mL of $Na_2S_2O_3$ solution was added to the reaction mixture and then extracted with dichloromethane (3×30 mL). The organic layer was dried over Na_2SO_4 and the solvent was evaporated in reduced pressure. The crude product was purified by column chromatography on a silica gel column (eluent: ethyl acetate/hexane, 5:95, v/v) to afford 9,18-dihydrobenzo[*a*]benzo[1,2]carbazolo[3,4-*c*]carbazole (**5**) as a light-yellow solid (48 mg, 80%, m.p: 210–211 °C). **^{1}H NMR (400 MHz, DMSO-*d$_6$*) δ** 12.65 (bs, 2H), 8.86 (dd, *J* = 5.9, 3.4 Hz, 2H), 8.73 (dd, *J* = 5.9, 3.4 Hz, 2H), 7.77 (d, *J* = 8.1 Hz, 2H), 7.74 – 7.68 (m, 4H), 7.66 (d, *J* = 8.1 Hz, 2H), 7.41 (t, *J* = 7.5 Hz, 2H), 7.09 (t, *J* = 7.6 Hz, 2H). **^{13}C{^{1}H} NMR (101 MHz, DMSO-*d$_6$*) δ** 139.0, 135.2, 129.4, 128.3, 126.0, 124.6, 124.2, 124.0, 123.6, 123.5, 122.6, 120.9, 118.2, 117.0, 112.3, 111.5. **HRMS** (ESI-TOF) *m/z*: [M + H]$^+$ calcd for $C_{30}H_{19}N_2$, 407.1543; found 407.1544.

Gram-Scale experiments

(a) Compound **2a** (943 mg, 95%) was synthesized by following general procedure **J** using 3-(2,2-diphenylvinyl)-1*H*-indole (**1a**) (1.0 g, 3.39 mmol) in MeCN (34 mL).

(b) Compound **4a** (906 mg, 91%) was synthesized by following general procedure **J** using 3-(1,2,2-triphenylvinyl)-1*H*-indole (**3a**) (1.0 g, 2.69 mmol) in MeCN (27 mL).

Control experiments

(a) Free-radical inhibition experiments: The experiments were performed according to general procedure **J** with 3-(2,2-diphenylvinyl)-1*H*-indole (**1a**) (59 mg, 0.2 mmol) and TEMPO (47 mg, 0.3 mmol) or BHT (66 mg, 0.3 mmol). After purification, **2a** was obtained with 95% and 97% yields, respectively.

(b) Deuterium labeling experiment: The experiment was performed according to general procedure **J** using 3-(2,2-diphenylvinyl)-1*H*-indole-1-d (**1a–D**) 59 mg, 0.2 mmol) for 6 h under standard conditions. First, the conversion to **2a** was exclusively obtained in a 41% ratio. Then, this mixture was again exposed for 3 h to standard conditions. The conversion was complete, and after purification, **2a** was isolated in a 90% yield (52 mg).

3. References

1. Chen, X.; Fan, H.; Zhang, S.; Yu, C.; Wang, W. Facile Installation of 2-Reverse Prenyl Functionality into Indoles by a Tandem *N*-Alkylation–Aza-Cope Rearrangement Reaction and Its Application in Synthesis. *Chem. Eur. J.* **2016**, 22, 716-723.

2. Guo, J.; Hao, Y.; Ji, X.; Wang, Z.; Liu, Y.; Ma, D.; Li, Y.; Pang, H.; Ni, J.; Wang, Q. Optimization, structure–activity relationship, and mode of action of nortopsentin analogues containing thiazole and oxazole moieties. *J. Agric. Food. Chem.* **2019**, 67, 10018−10031.

3. Jayaraman, A.; Castro, L. C. M.; Desrosiers, V.; Fontaine, F. G. Metal-free borylative dearomatization of indoles: exploring the divergent reactivity of aminoborane C–H borylation catalysts. *Chem. Sci.* **2018**, 9, 5057−5063.

4. Sakata, Y.; Yasui, E.; Takatori, K.; Suzuki, Y.; Mizukami, M.; Nagumo, S. Syntheses of polycyclic tetrahydrofurans by cascade reactions consisting of five-membered ring selective Prins cyclization and Friedel–Crafts cyclization. *J. Org. Chem.* **2018**, 83, 9103−9118.

5. Tayu, M.; Watanabe, R.; Isogi, S.; Saito, N. A Catalytic Construction of Indoles via Formation of Ruthenium Vinylidene Species from *N*-Arylynamides. *Adv. Synth. Catal.* **2021**, 363, 1147−1151.

6. Yao, C. H.; Song, J. S.; Chen, C. T.; Yeh, T. K.; Hsieh, T. C.; Wu, S. H.; Huang, C.-Y.; Huang, Y. L.; Wang, M. H.; Liu, Y. W.; Tsai, C. H.; Kumar, C. R.; Lee, J. C. Synthesis and biological evaluation of novel C-indolylxylosides as sodium-dependent glucose co-transporter 2 inhibitors. *Eur. J. Med. Chem.* **2012**, 55, 32−38.

7. Lafzi, F.; Taskesenligil, Y.; Canımkurbey, B.; Pıravadılı, S.; Kilic, H.; Saracoglu, N. Four-winged propeller-shaped indole-modified and indole-substituted tetraphenylethylenes: greenish-blue emitters with aggregation-induced emission features for conventional organic light-emitting diodes. *ACS Omega* **2022**, 7, 44322−44337.

8. Jana, D.; Ghorai, B. K. Pyridine-cored V-shaped π-conjugated oligomers: synthesis and optical properties. *Tetrahedron* **2012**, 68, 7309−7316.

9. Saxena, P.; Maida, N.; Kapur, M. Dioxazolones as masked ester surrogates in the Pd(II)-catalyzed direct C–H arylation of 6,5-fused heterocycles. *Chem. Commun.* **2019**, 55, 11187−11190.

10. Zhou, S.; Guo, S.; Liu, W.; Yang, Q.; Sun, H.; Ding, R.; Qian, Z.; Feng, H. Rational design of reversibly photochromic molecules with aggregation-induced emission by introducing photoactive thienyl and benzothienyl groups. *J. Mater. Chem. C* **2020**, 8, 13197−13204.

11. Li, N.; Lian, X. L.; Li, Y. H.; Wang, T. Y.; Han, Z. Y.; Zhang, L.; Gong, L. Z. Gold-catalyzed direct assembly of aryl-annulated carbazoles from 2-alkynyl arylazides and alkynes. *Org. Lett.* **2016**, 18, 4178−4181.

12. Huang, J. R.; Qin, L.; Zhu, Y. Q.; Song, Q.; Dong, L. Multi-site cyclization via initial C–H activation using a rhodium(III) catalyst: rapid assembly of frameworks containing indoles and indolines. *Chem. Commun.* **2015**, 51, 2844−2847.

13. Cabrero-Antonino J. R.; Adam, R.; Junge, K.; Beller, M. Cobalt-catalysed reductive C–H alkylation of indoles using carboxylic acids and molecular hydrogen. *Chem. Sci.* **2017**, 8, 6439−6450.

14. Zhang, H.; Wang, H.-Y.; Luo, Y.; Chen, C.; Cao, Y.; Chen, P.; Guo, Y.-L.; Lan, Y.; Liu, G. Regioselective palladium-catalyzed CH Bond trifluoroethylation of indoles: exploration and mechanistic insight. *ACS Catal.* **2018**, 8, 2173– 2180

4. NMR Spectrum

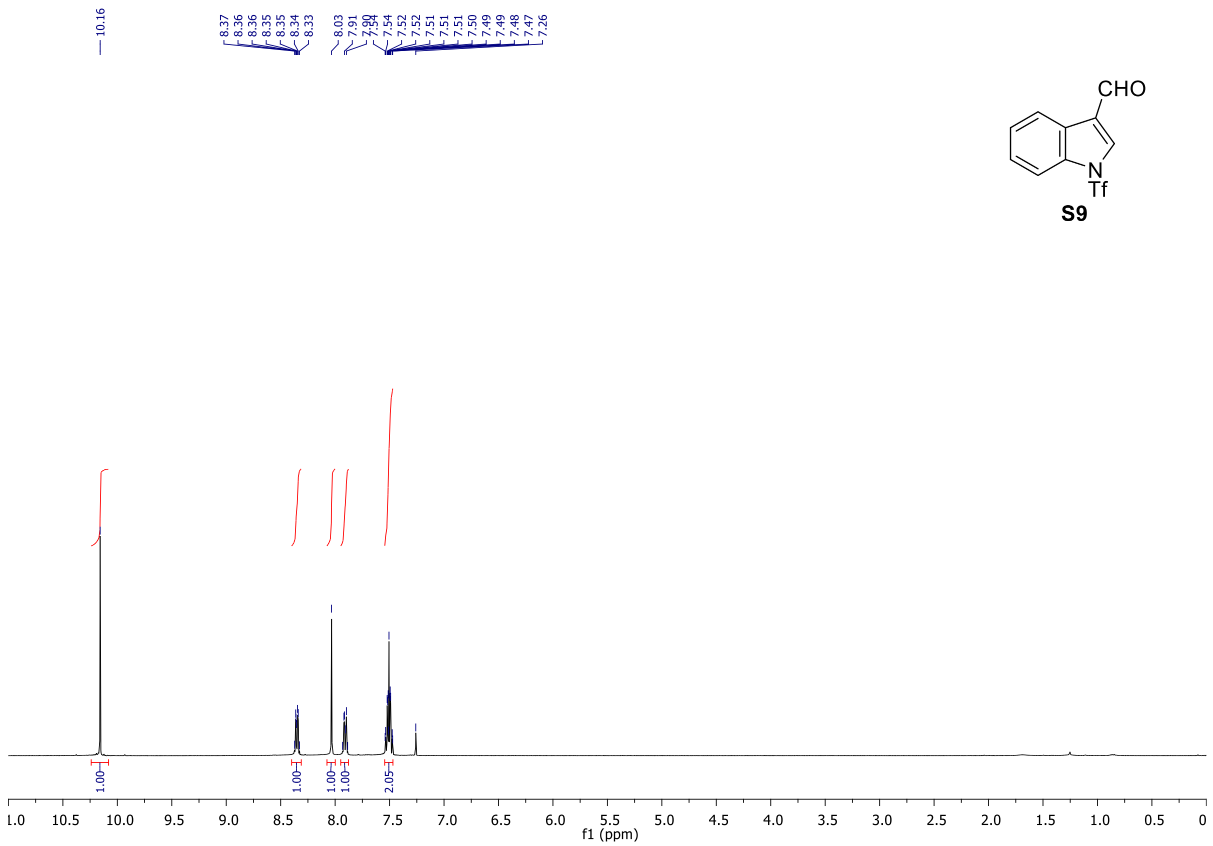

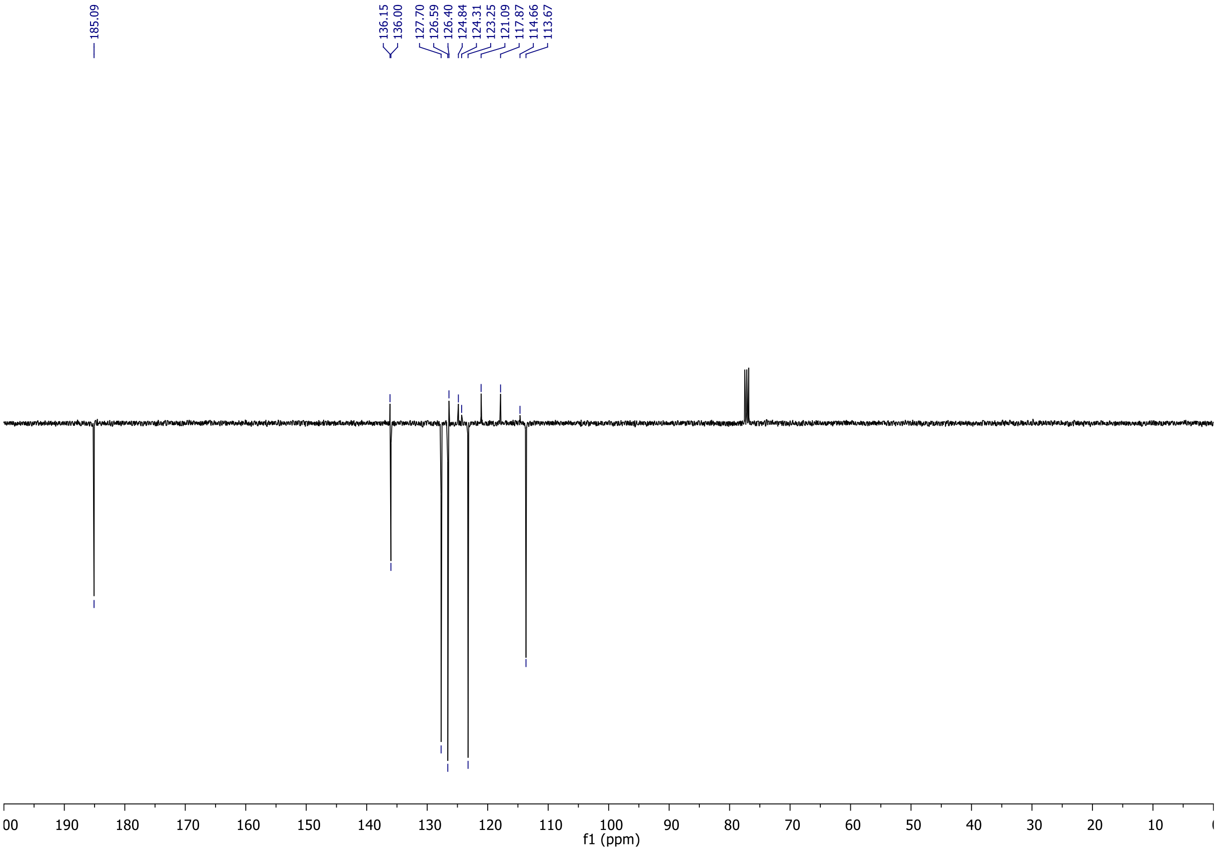

^{1}H NMR (400 MHz) and ^{13}C{^{1}H} (APT) NMR (100 MHz) spectra of **S9** ($CDCl_3$)

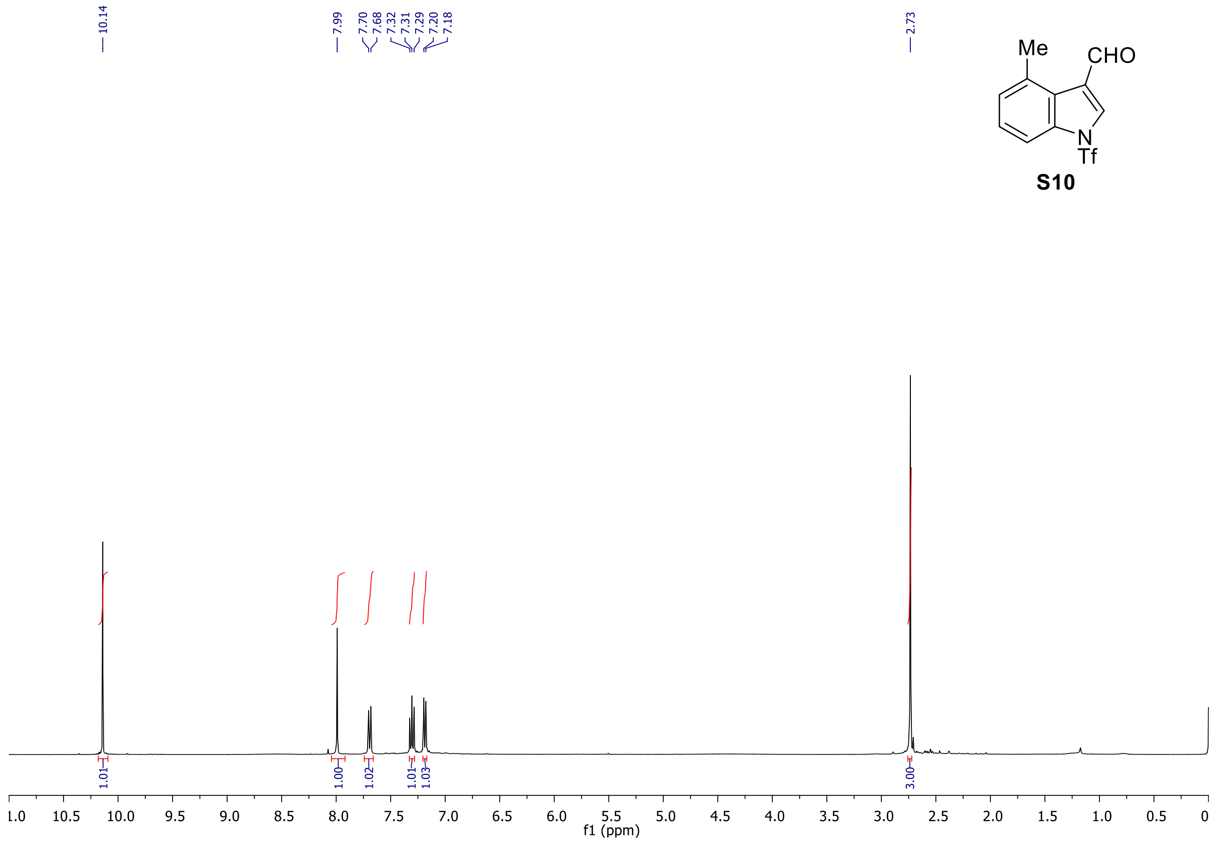

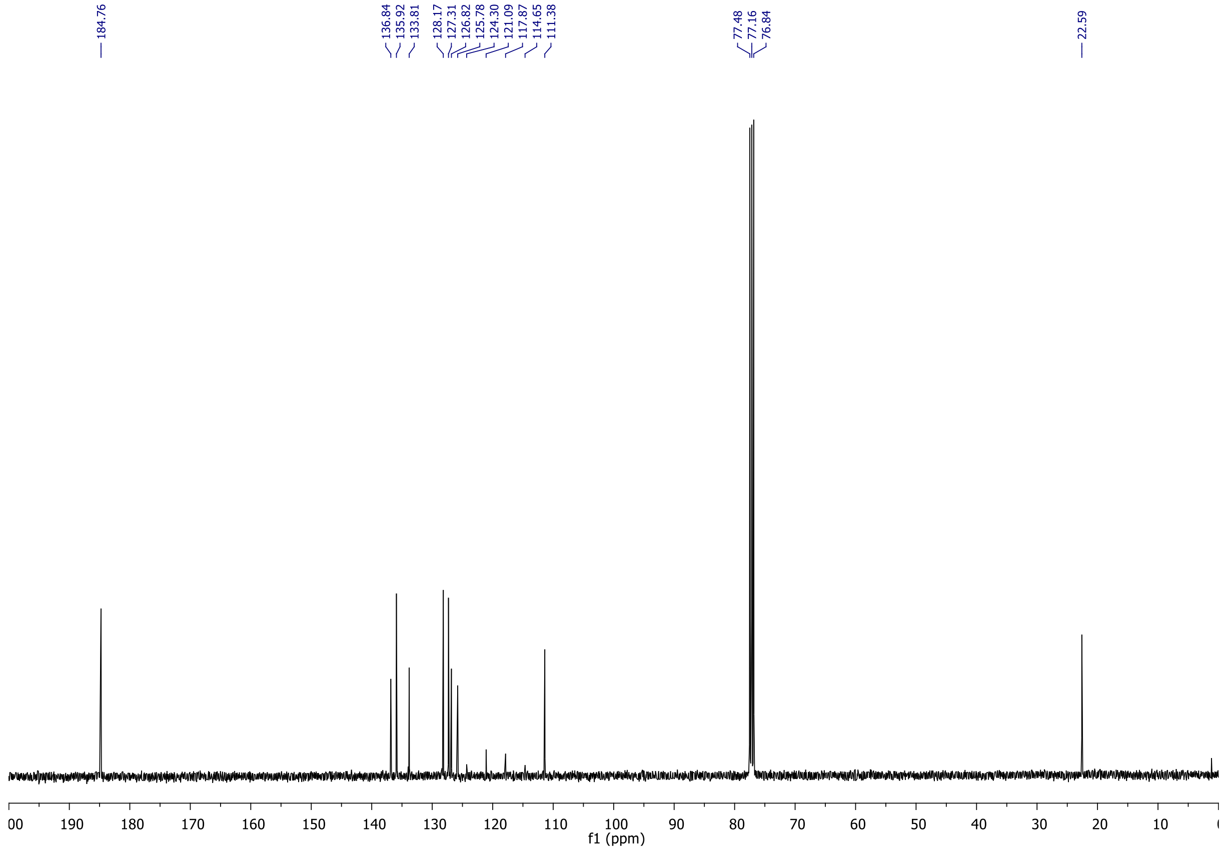

^{1}H NMR (400 MHz) and $^{13}C\{^{1}H\}$ NMR (100 MHz) spectra of **S10** ($CDCl_3$)

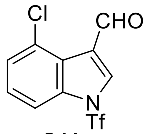

S11

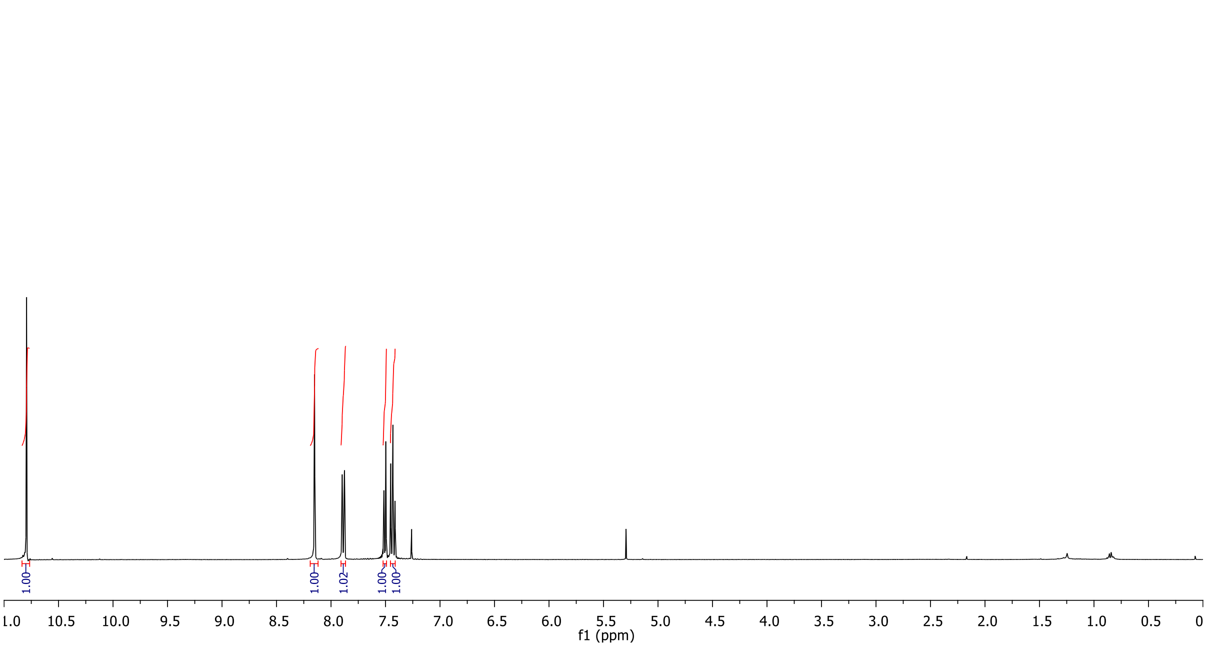

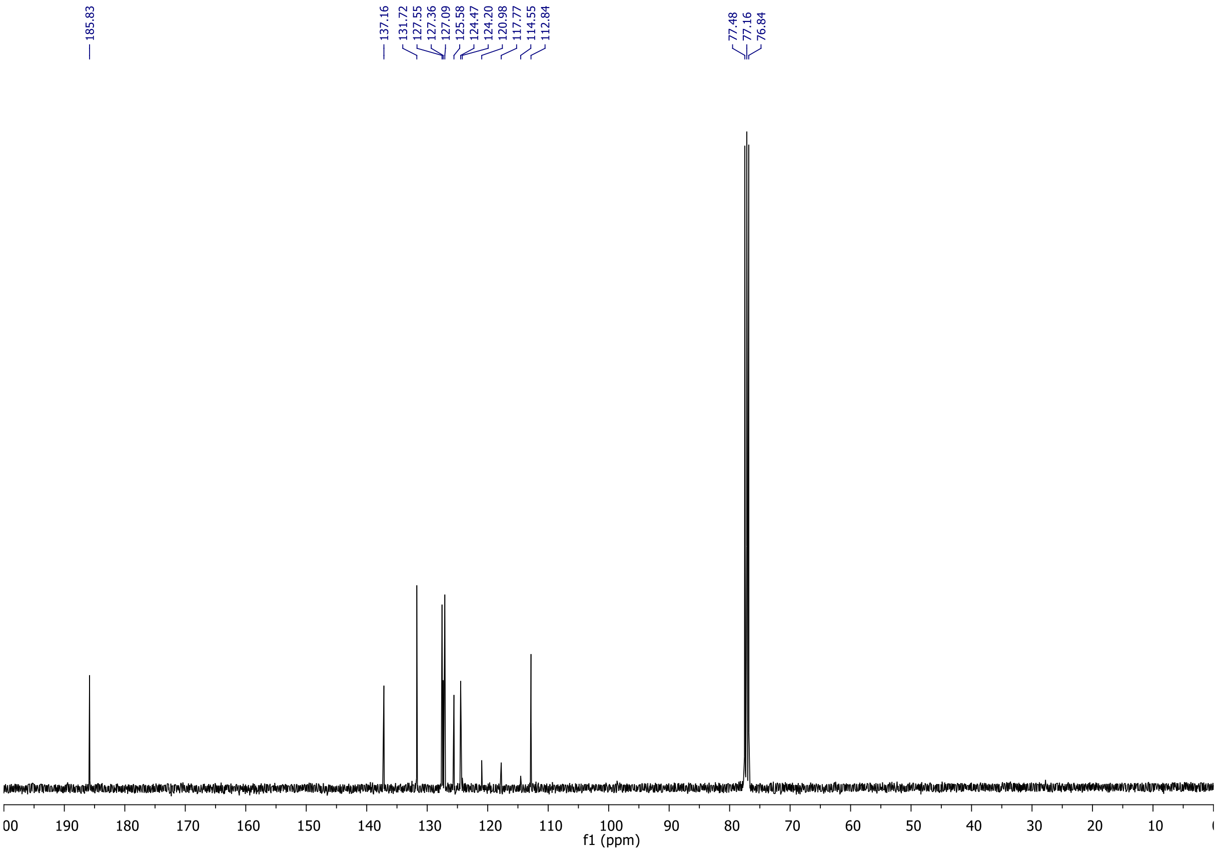

^{1}H NMR (400 MHz) and ^{13}C{^{1}H} NMR (100 MHz) spectra of **S11** ($CDCl_3$)

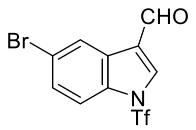

S12

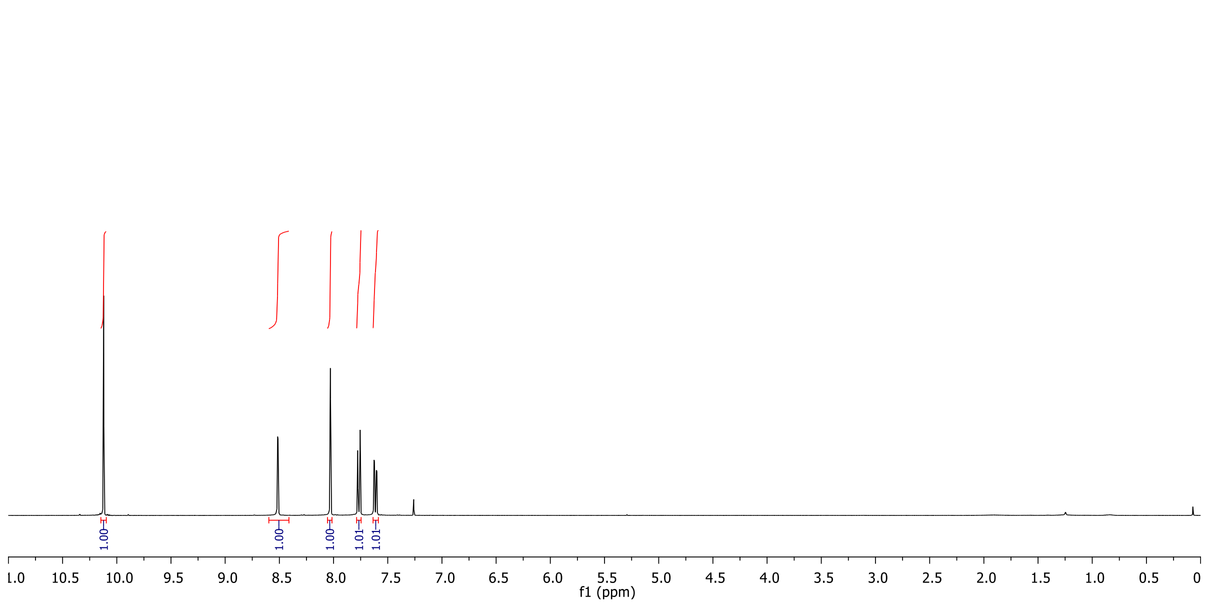

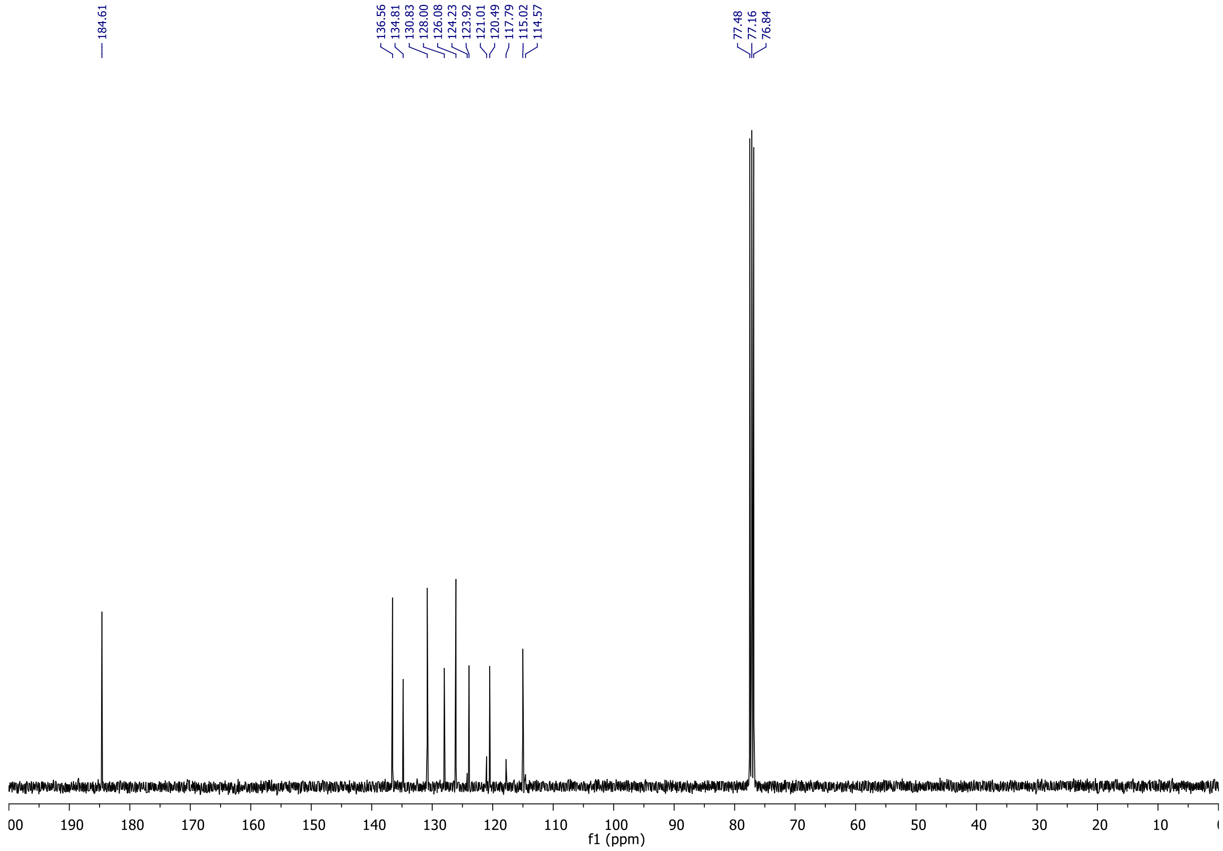

^{1}H NMR (400 MHz) and ^{13}C{^{1}H} NMR (100 MHz) spectra of **S12** ($CDCl_3$)

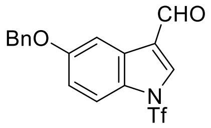

S13

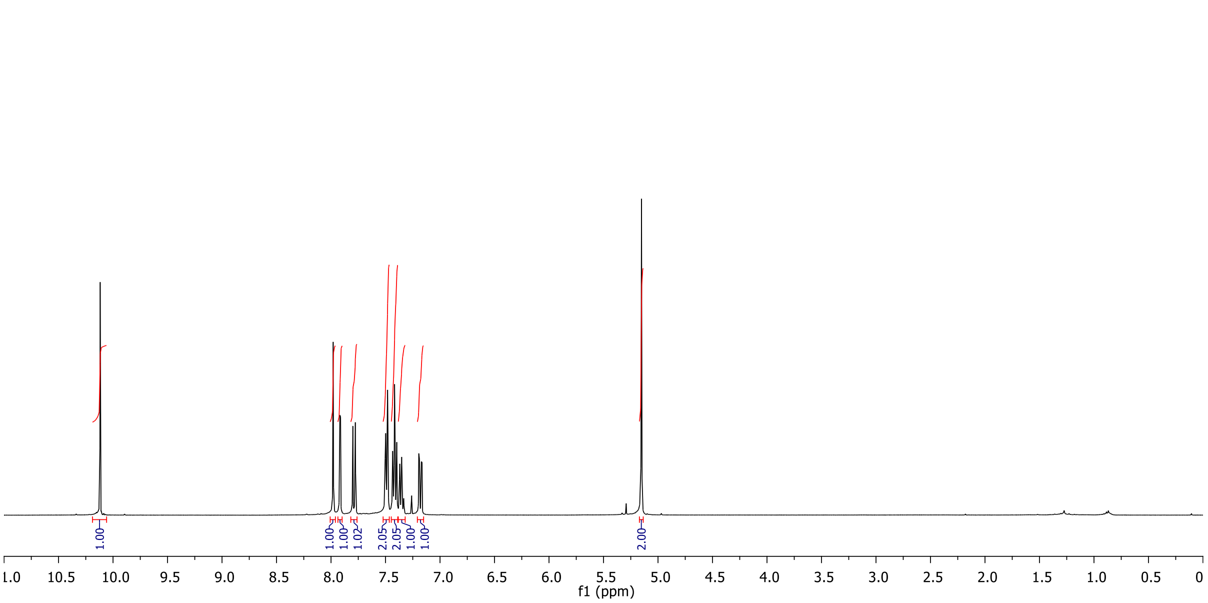

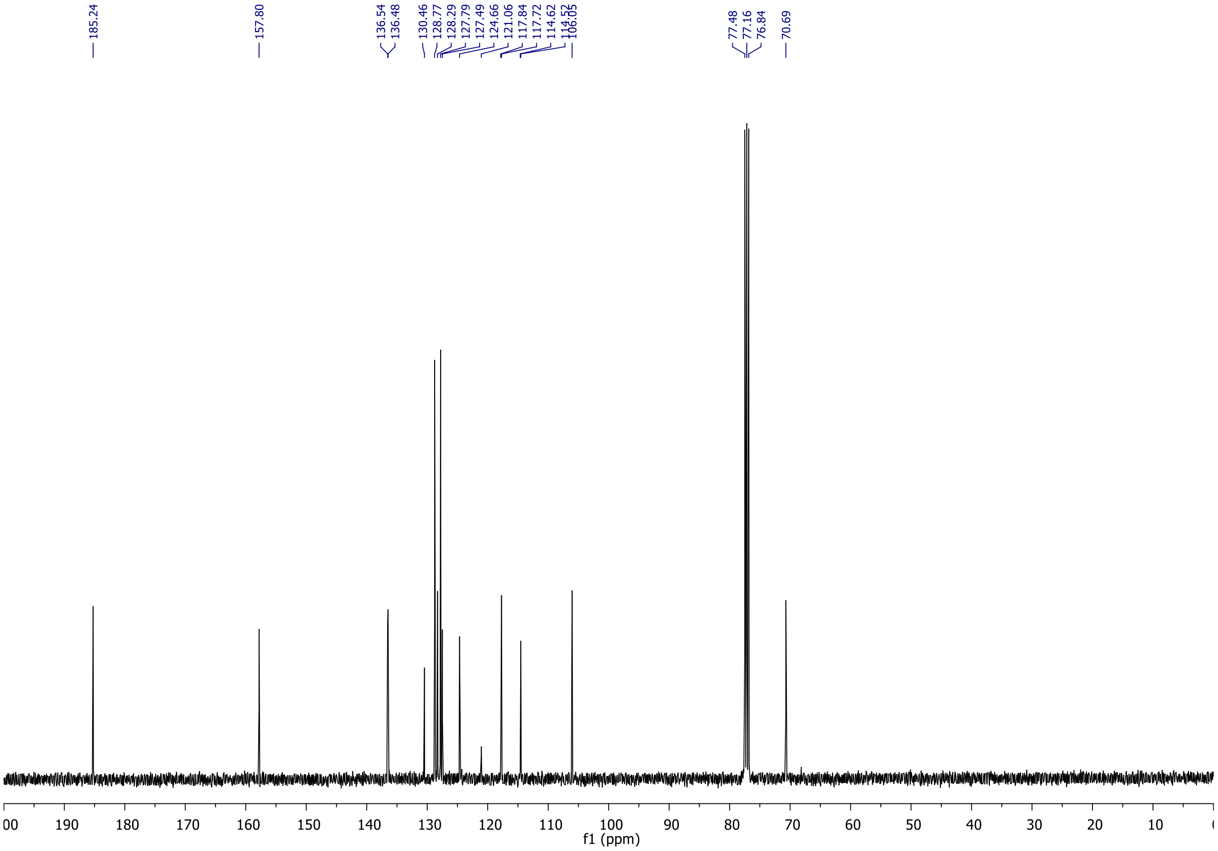

^{1}H NMR (400 MHz) and ^{13}C{^{1}H} NMR (100 MHz) spectra of **S13** ($CDCl_3$)

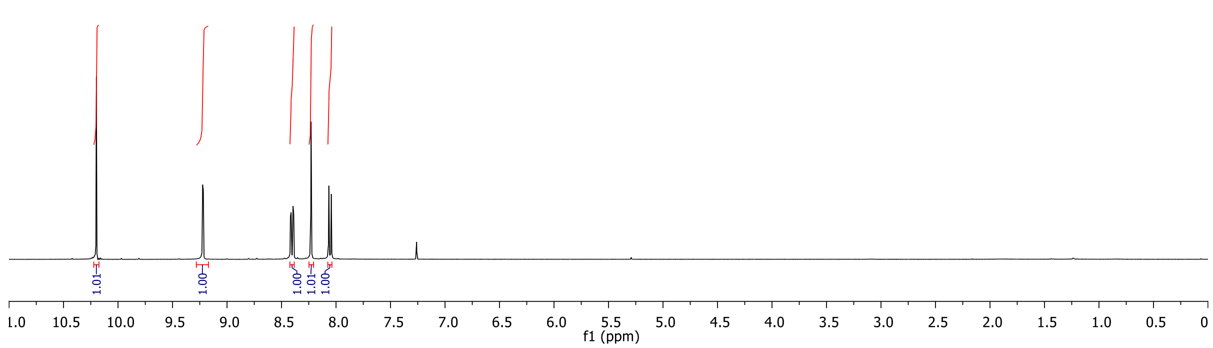

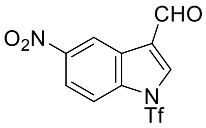

S14

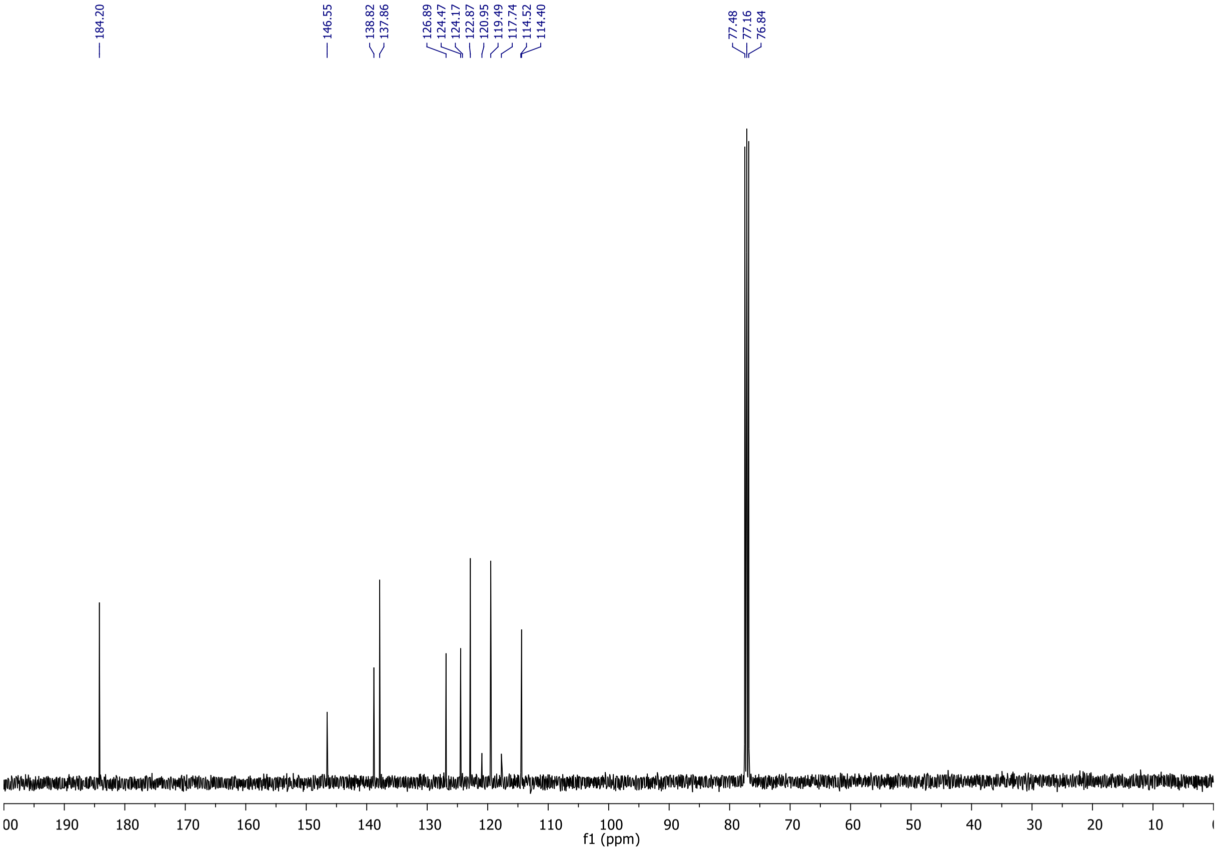

^{1}H NMR (400 MHz) and ^{13}C{^{1}H} NMR (100 MHz) spectra of **S14** ($CDCl_3$)

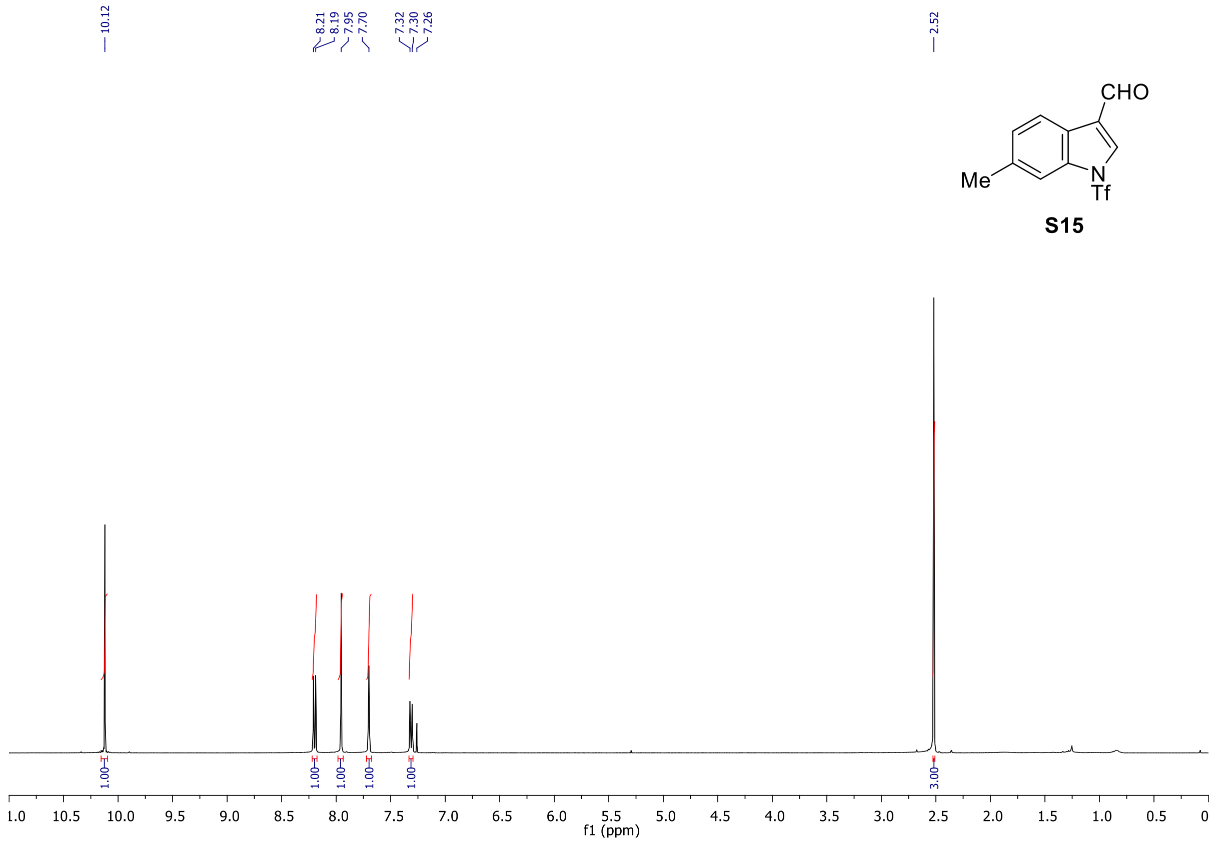

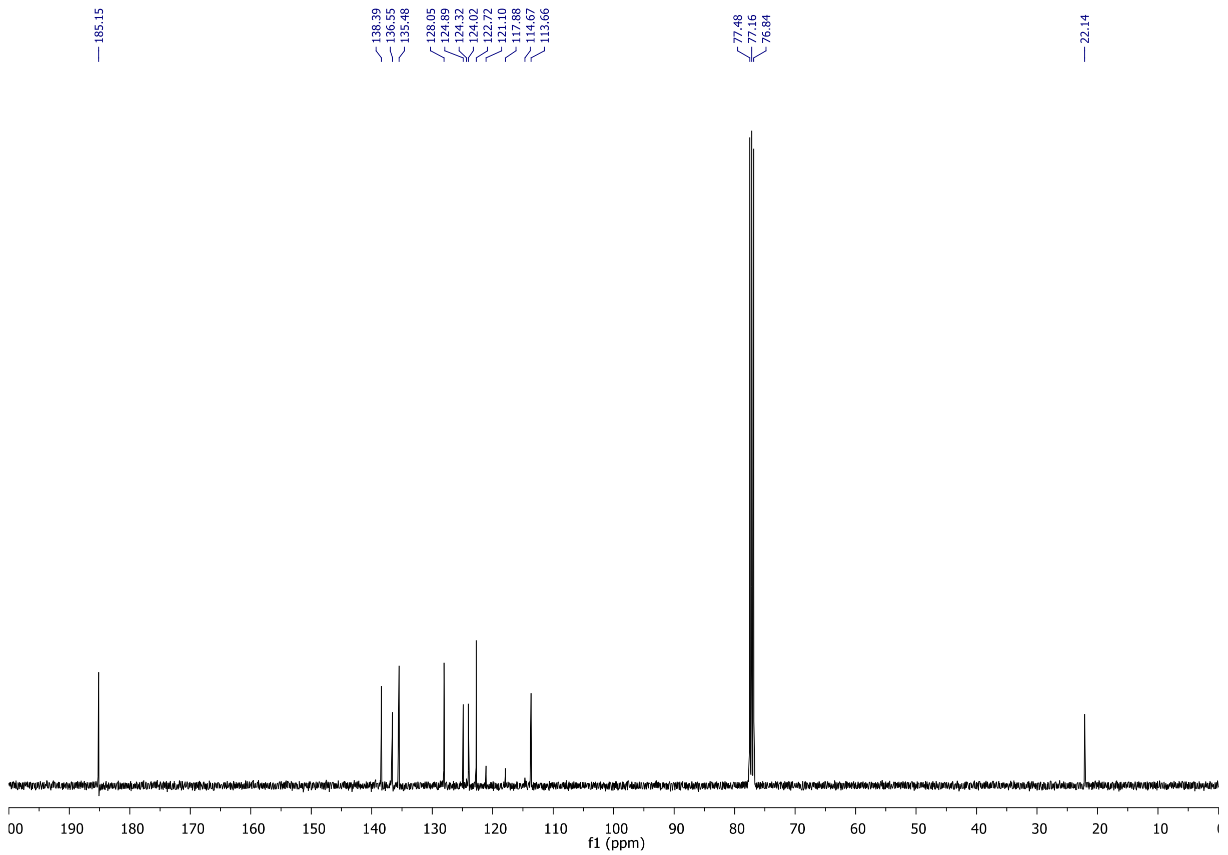

^{1}H NMR (400 MHz) and $^{13}C\{^{1}H\}$ NMR (100 MHz) spectra of **S15** ($CDCl_3$)

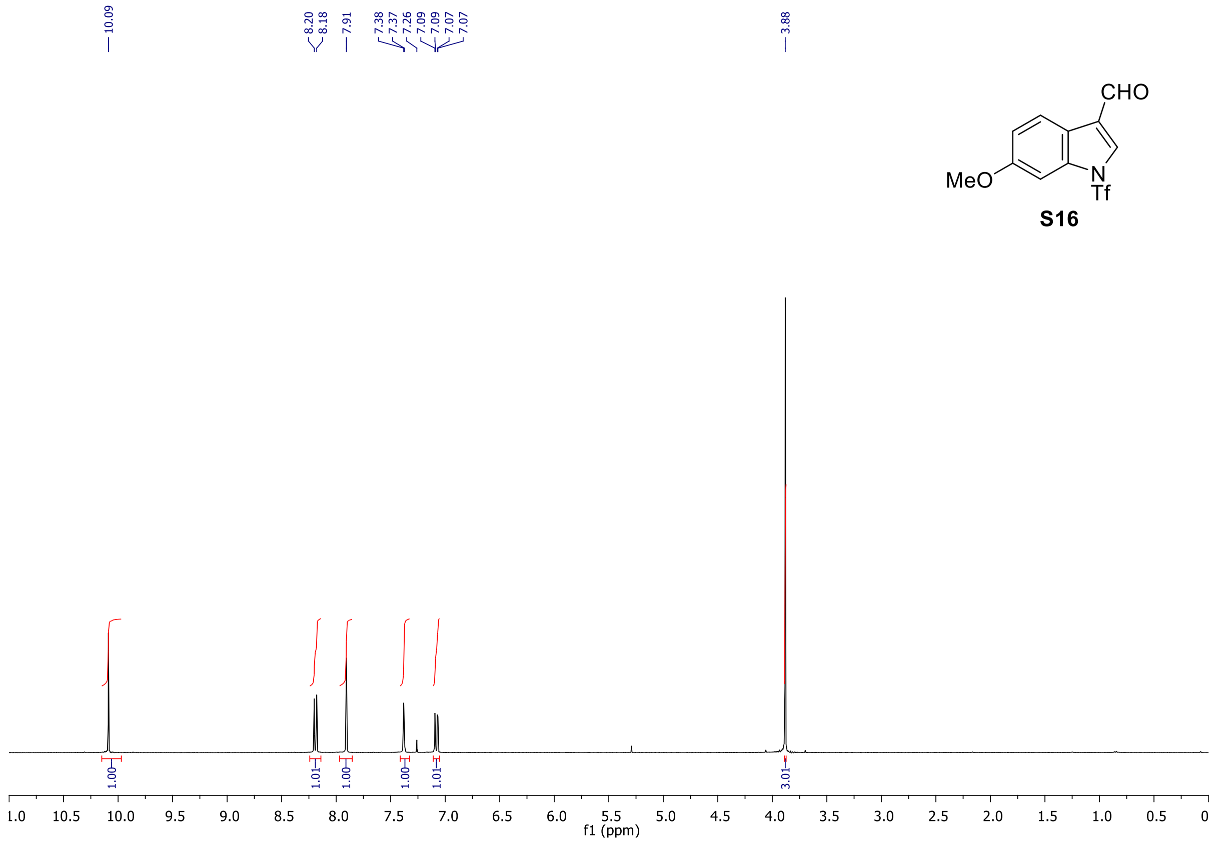

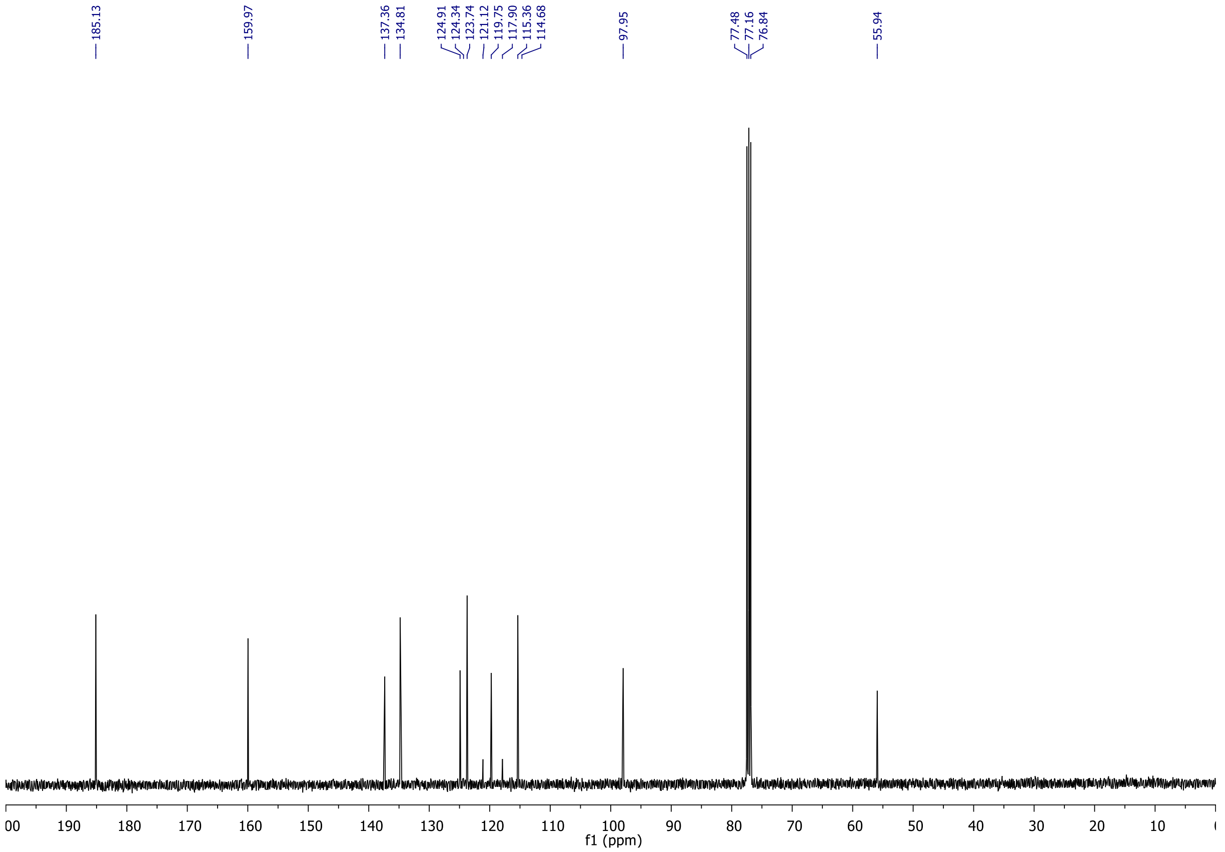

^{1}H NMR (400 MHz) and ^{13}C{^{1}H} NMR (100 MHz) spectra of **S16** ($CDCl_3$)

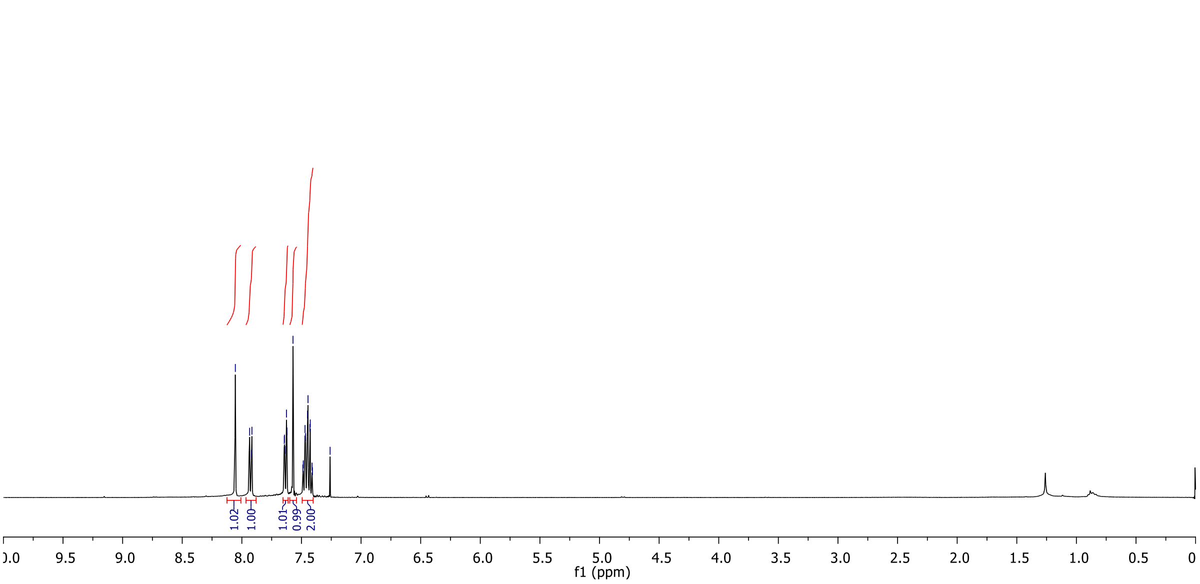

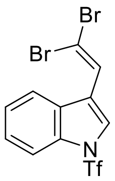

S17

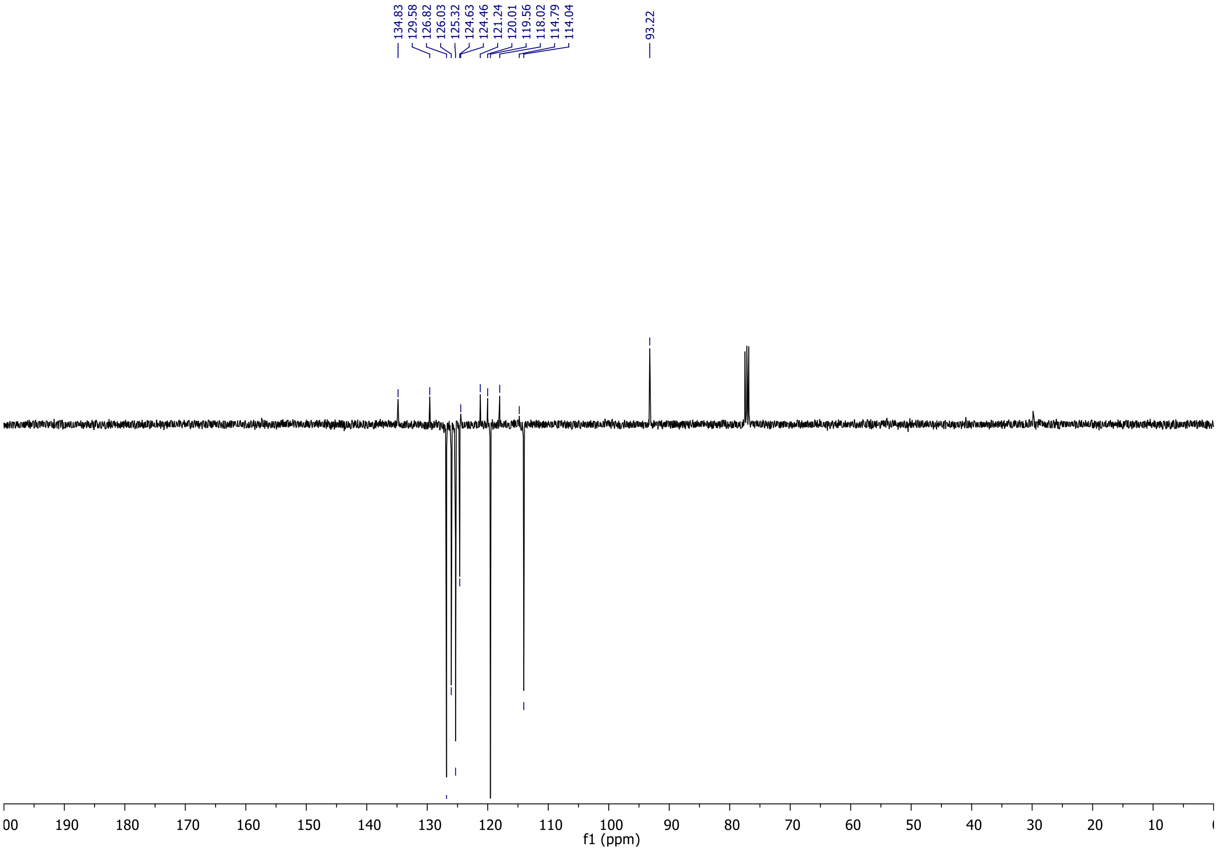

^{1}H NMR (400 MHz) and ^{13}C{^{1}H} (APT) NMR (100 MHz) spectra of **S17** ($CDCl_3$)

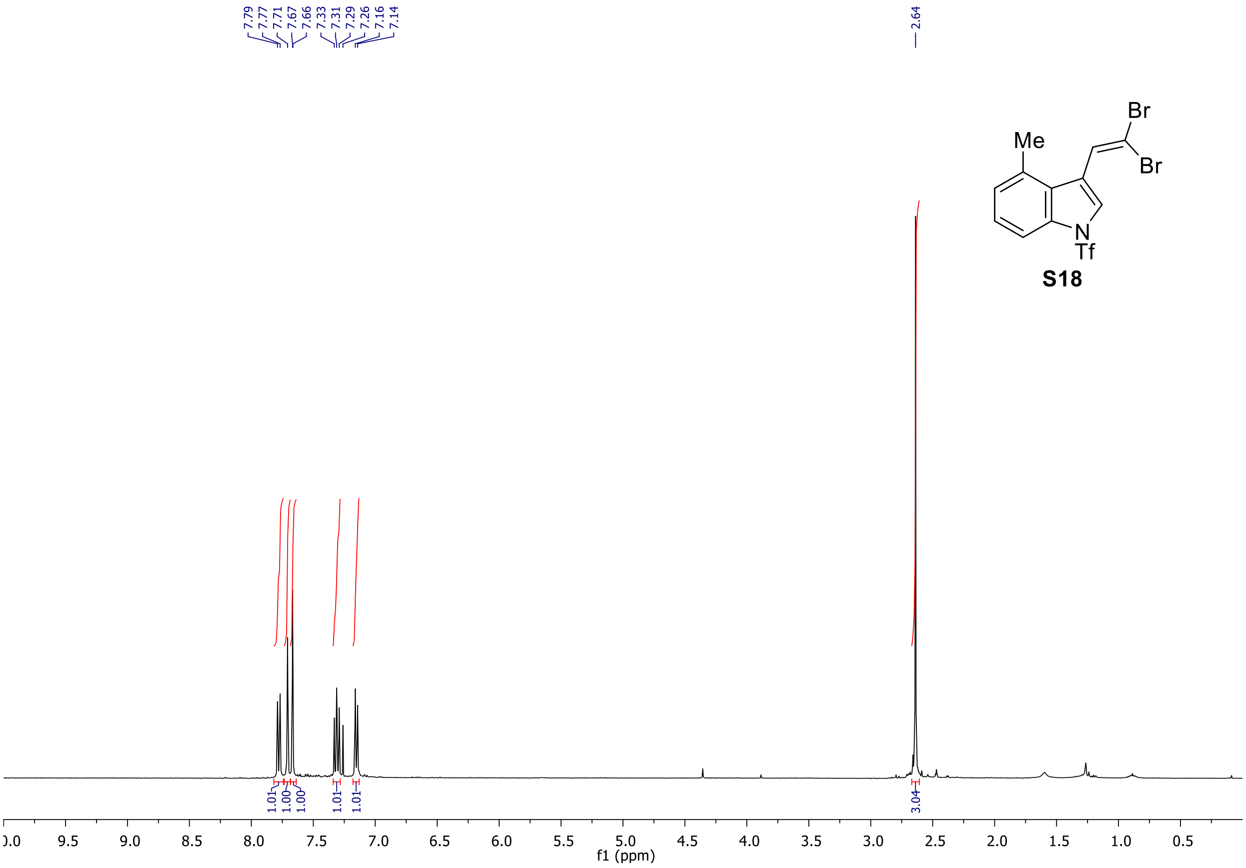

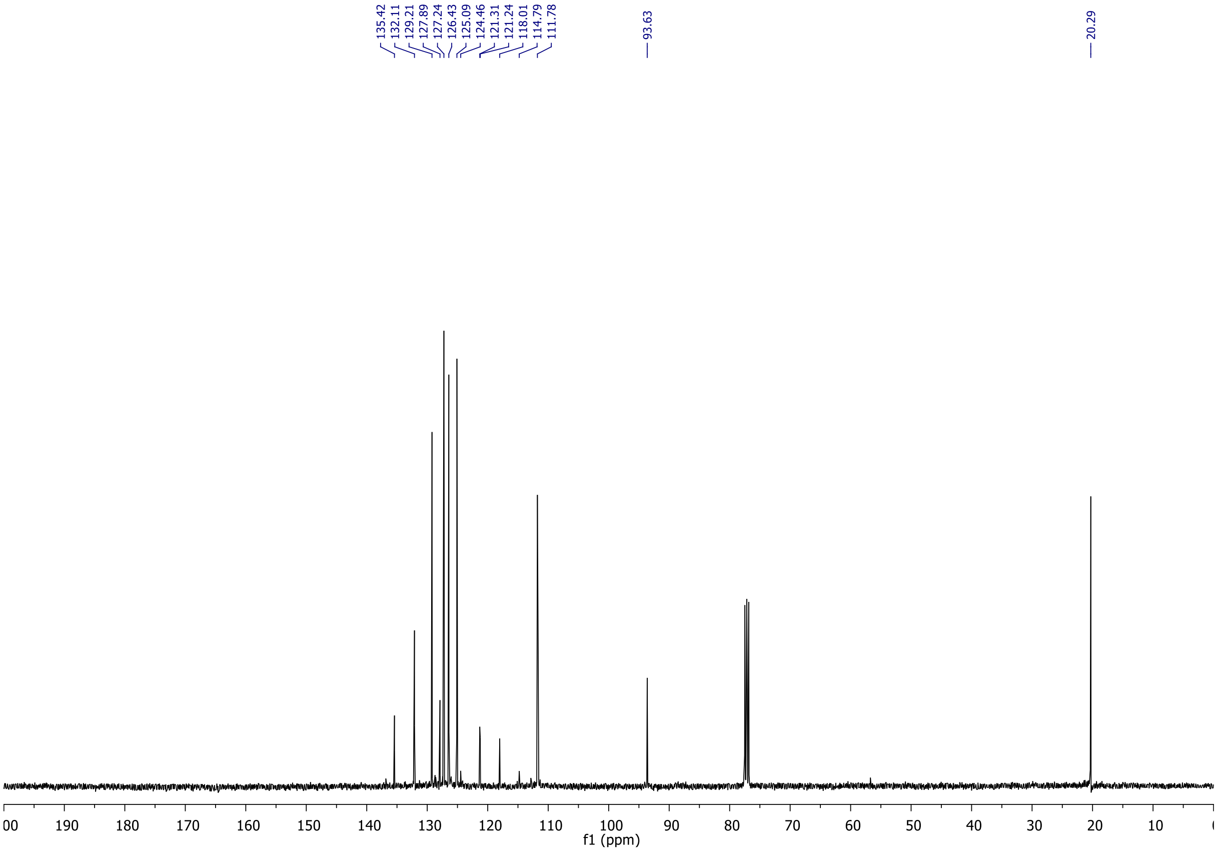

^{1}H NMR (400 MHz) and ^{13}C{^{1}H} NMR (100 MHz) spectra of **S18** ($CDCl_3$)

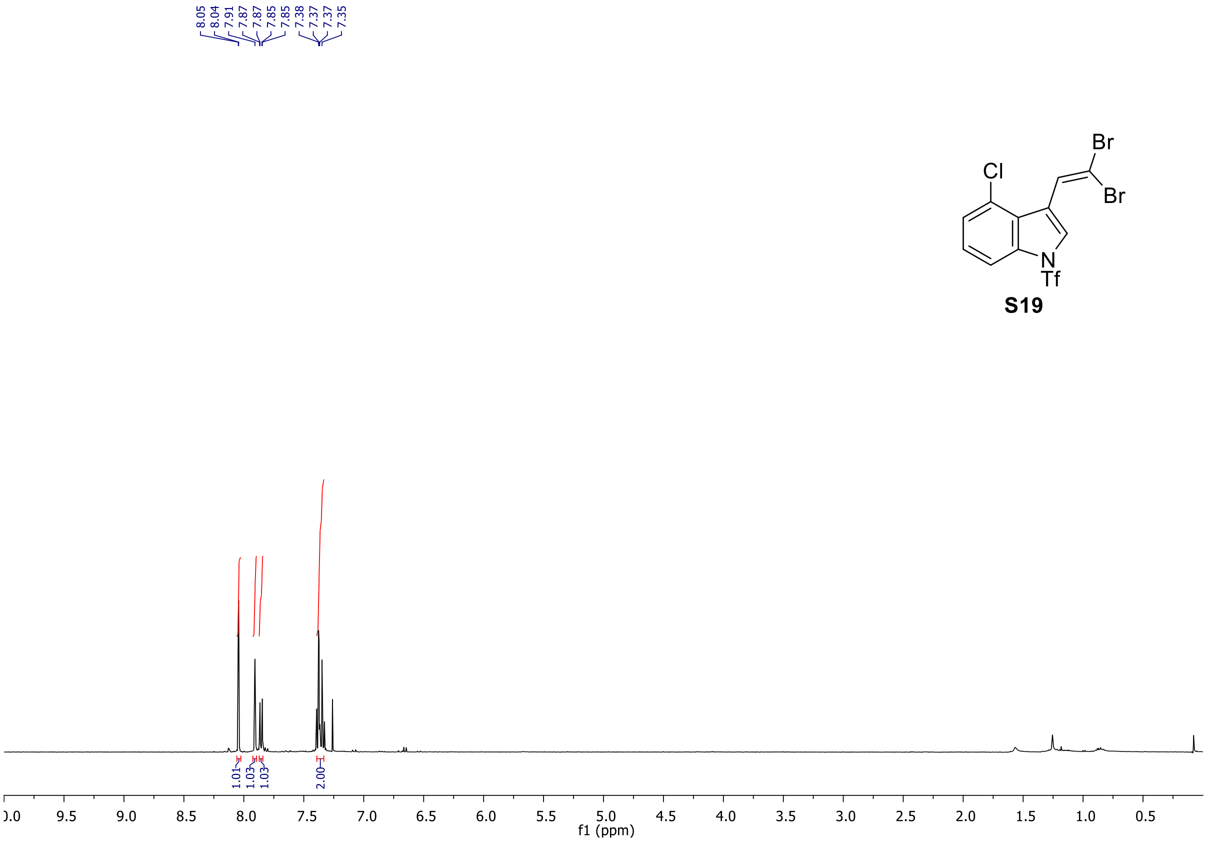

S19

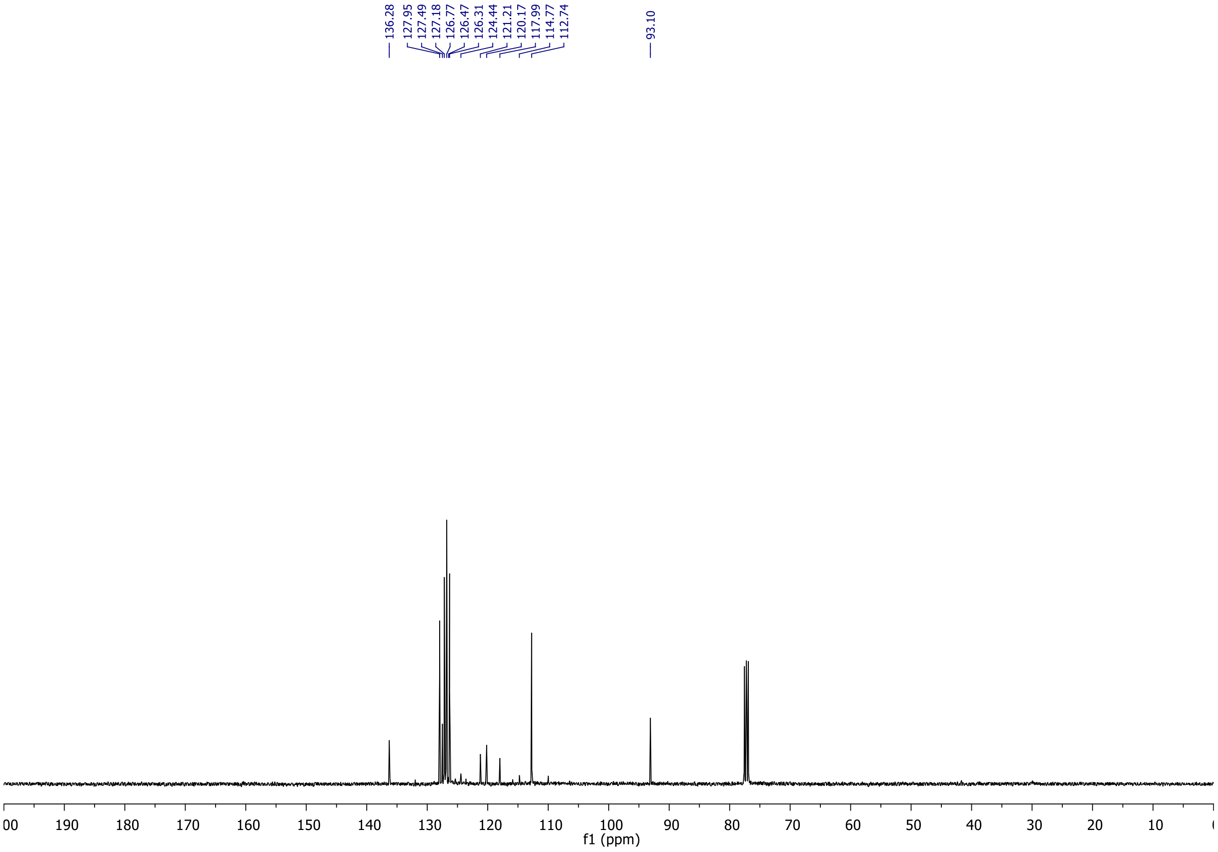

^{1}H NMR (400 MHz) and ^{13}C{^{1}H} NMR (100 MHz) spectra of **S19** ($CDCl_3$)

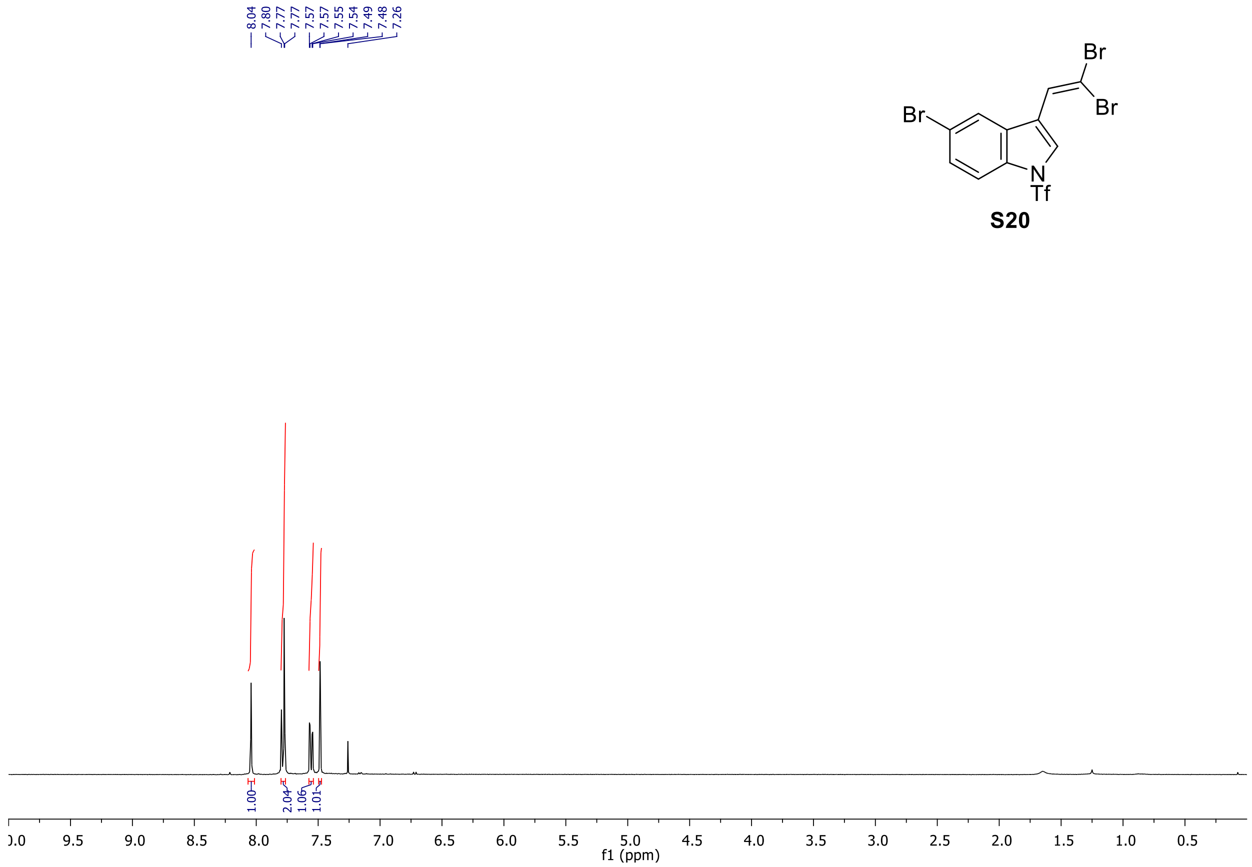

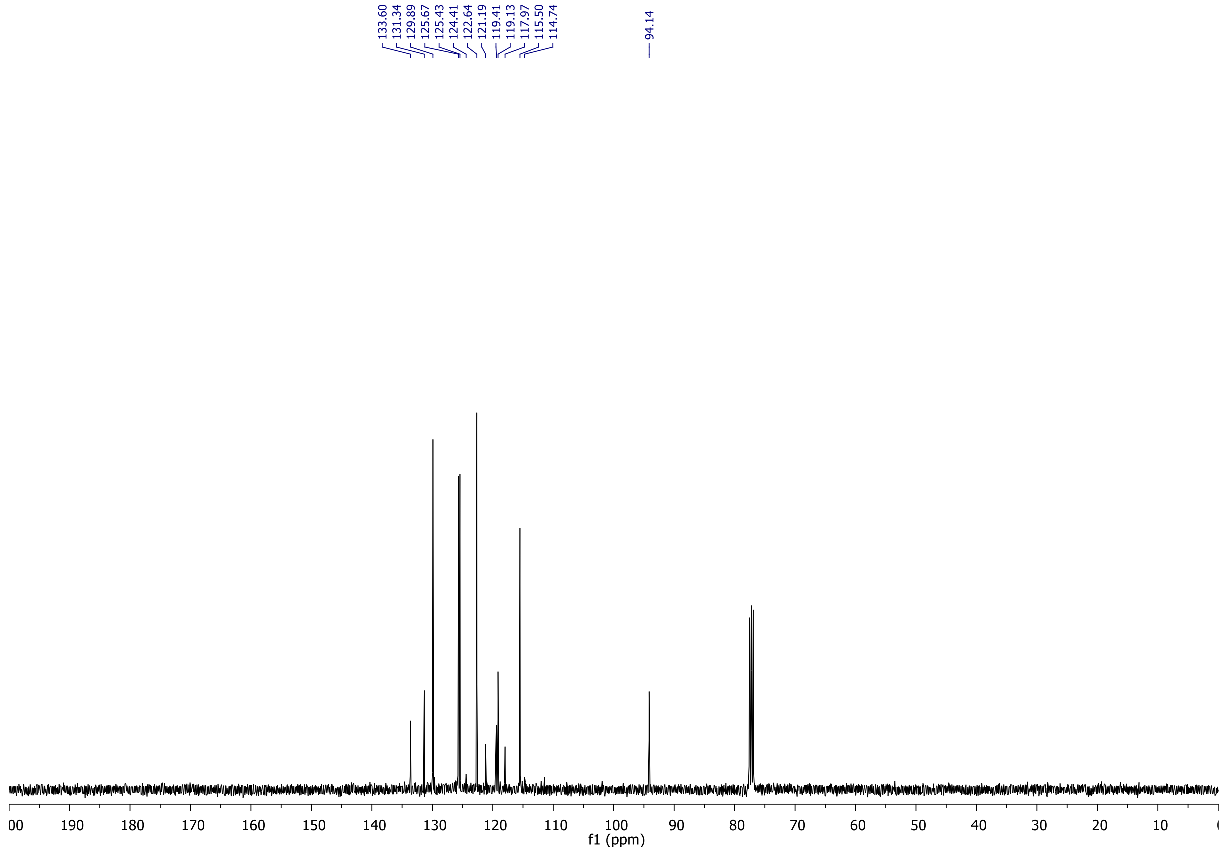

^{1}H NMR (400 MHz) and ^{13}C{^{1}H} NMR (100 MHz) spectra of **S20** ($CDCl_3$)

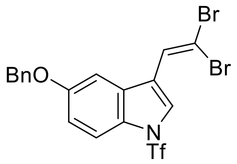

S21

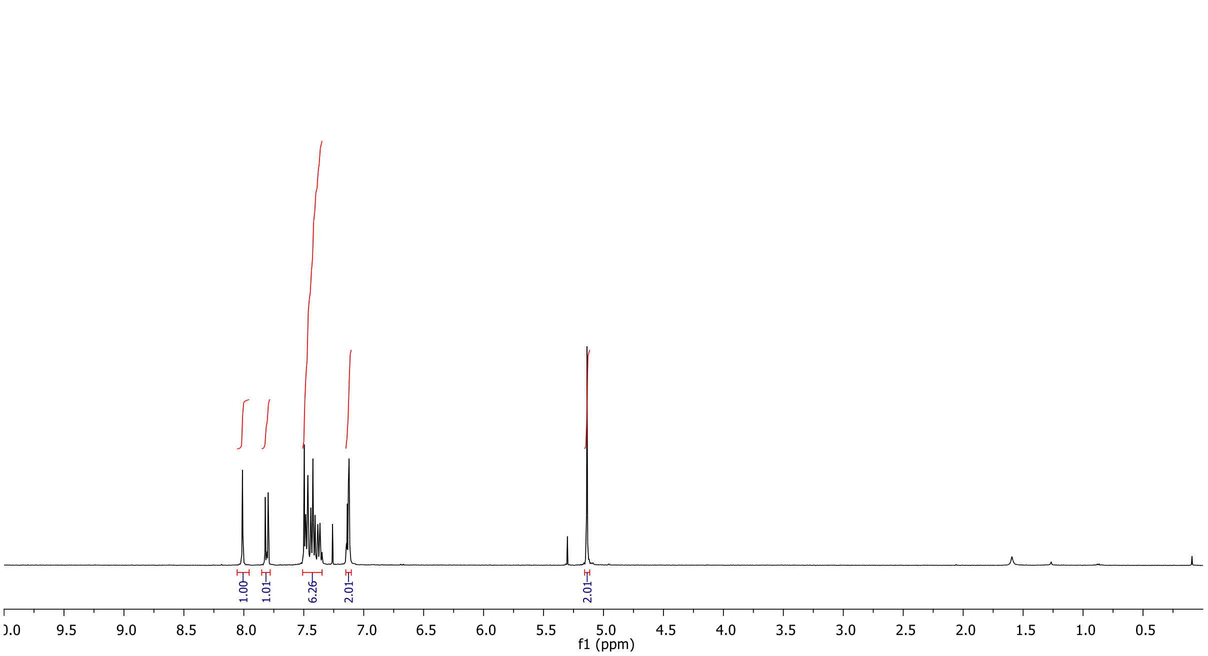

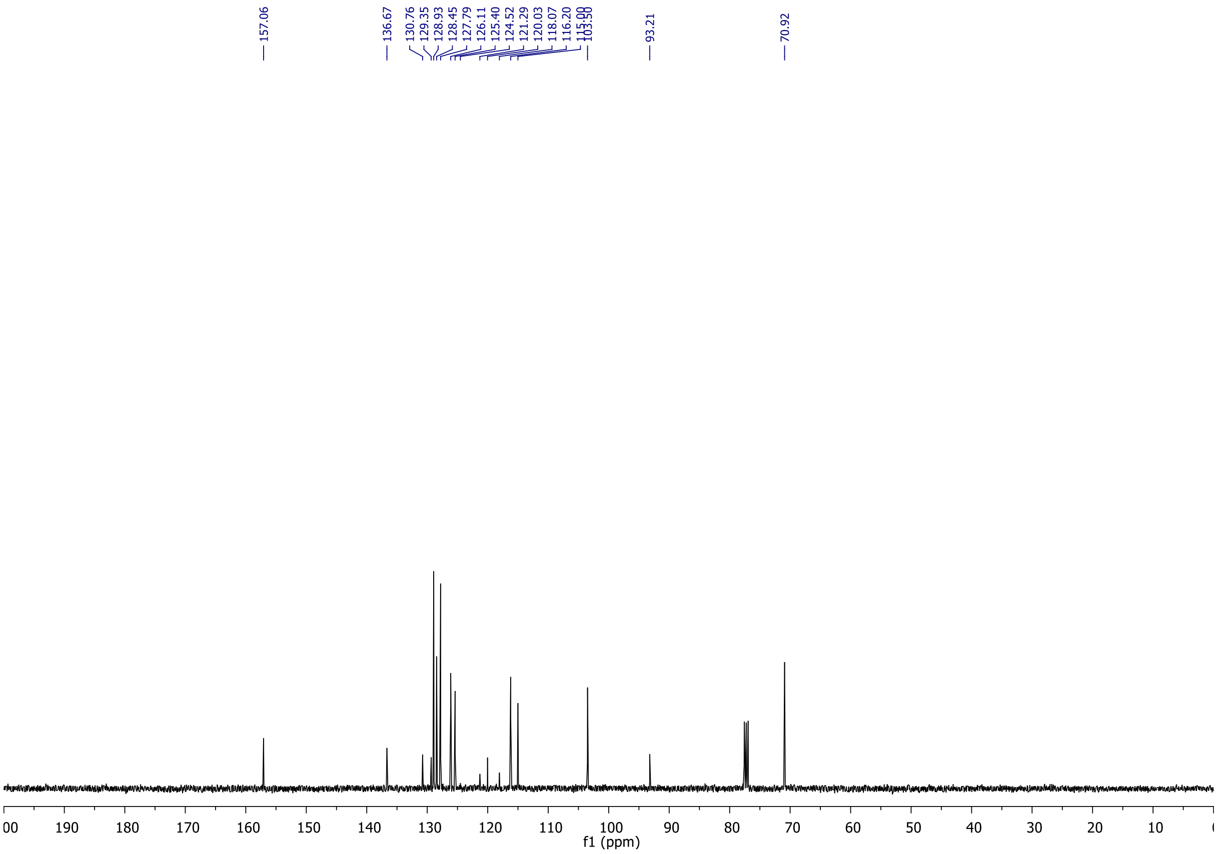

^{1}H NMR (400 MHz) and ^{13}C{^{1}H} NMR (100 MHz) spectra of **S21** ($CDCl_3$)

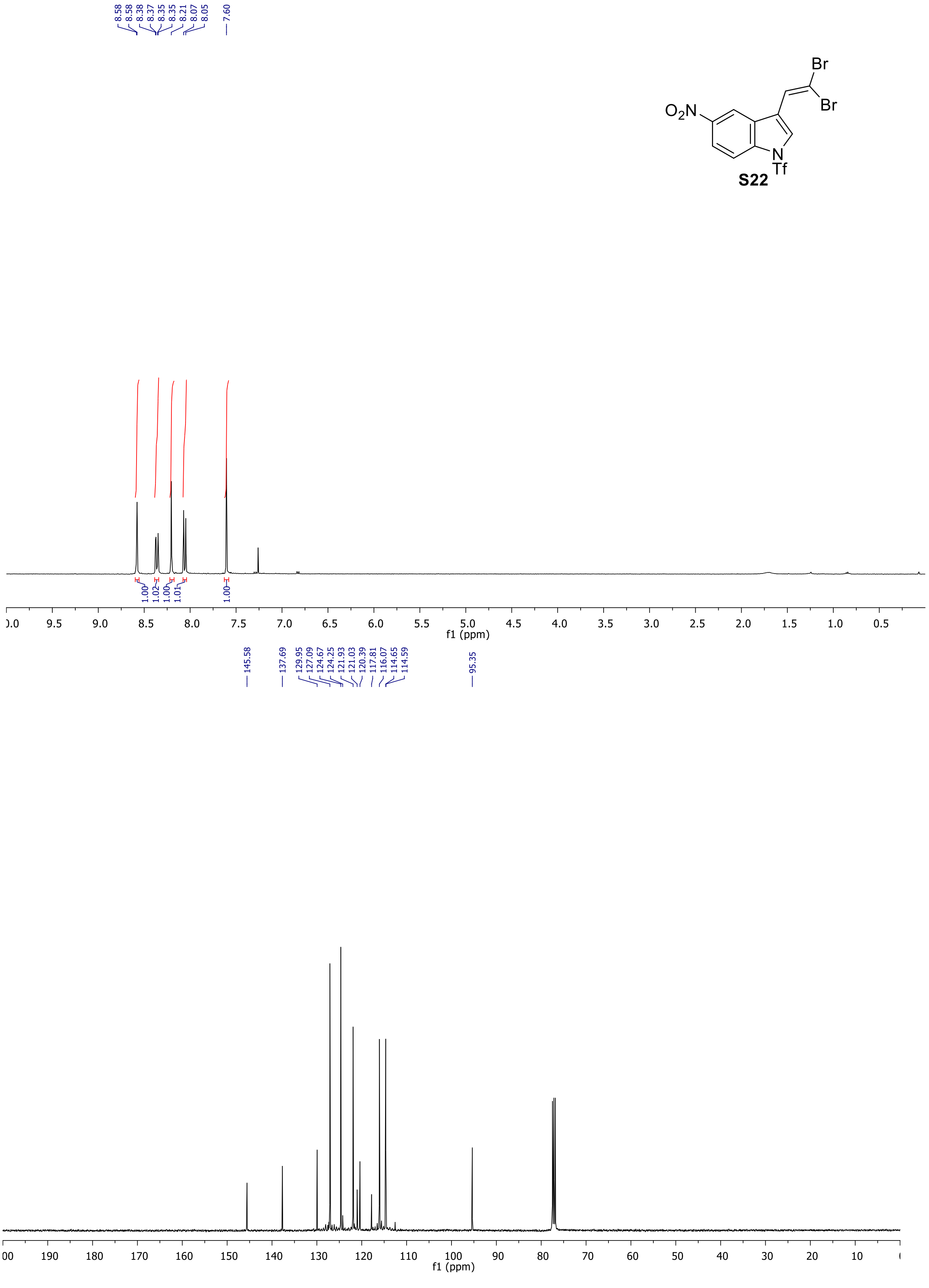

^{1}H NMR (400 MHz) and ^{13}C{^{1}H} NMR (100 MHz) spectra of **S22** ($CDCl_3$)

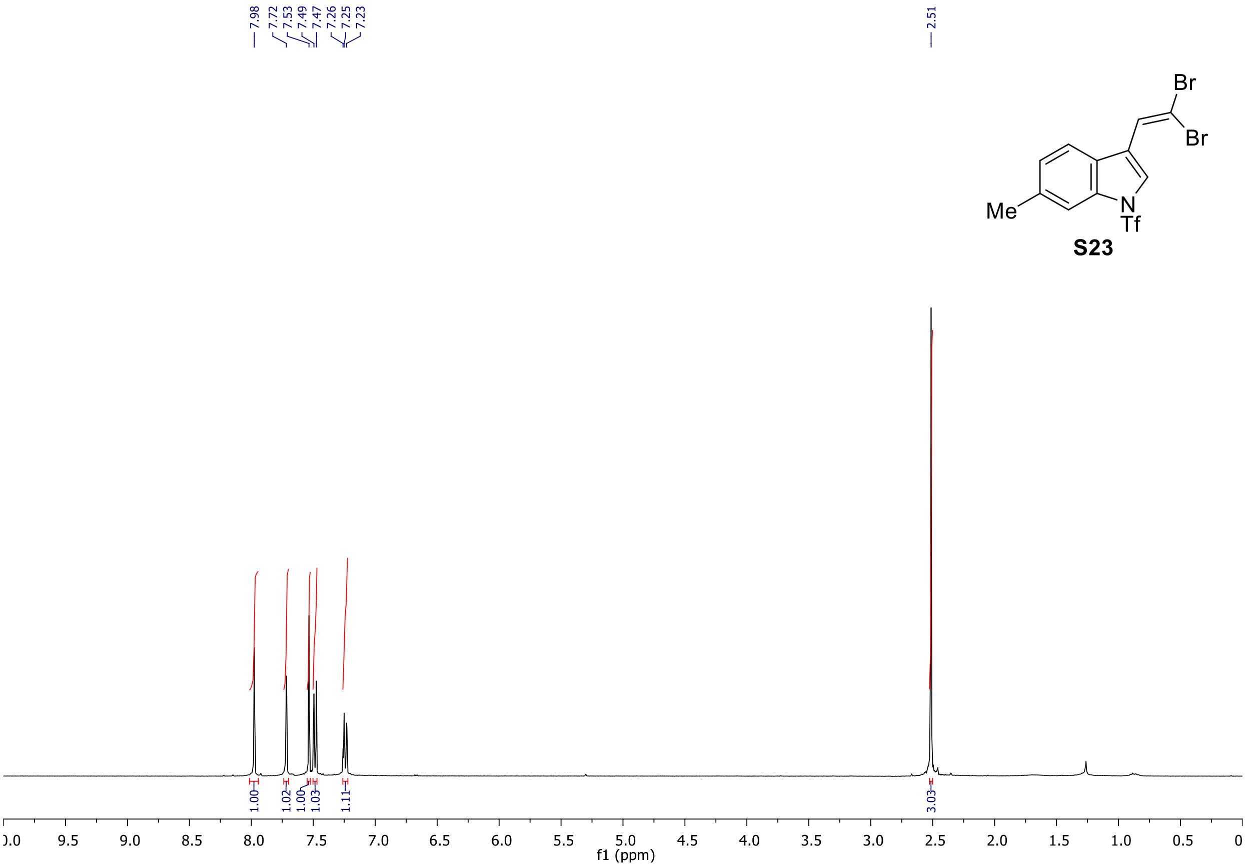

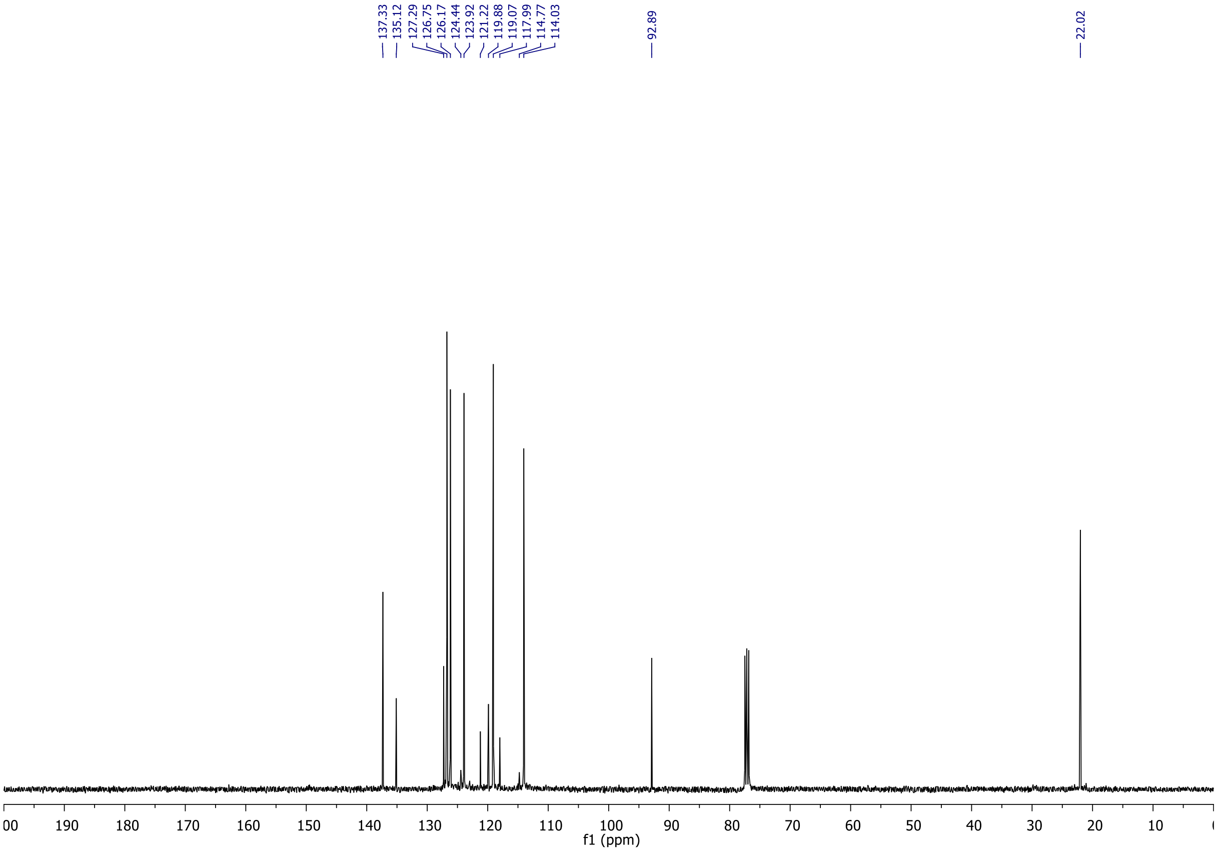

^{1}H NMR (400 MHz) and $^{13}C\{^{1}H\}$ NMR (100 MHz) spectra of **S23** ($CDCl_3$)

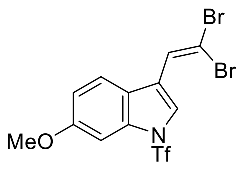

S24

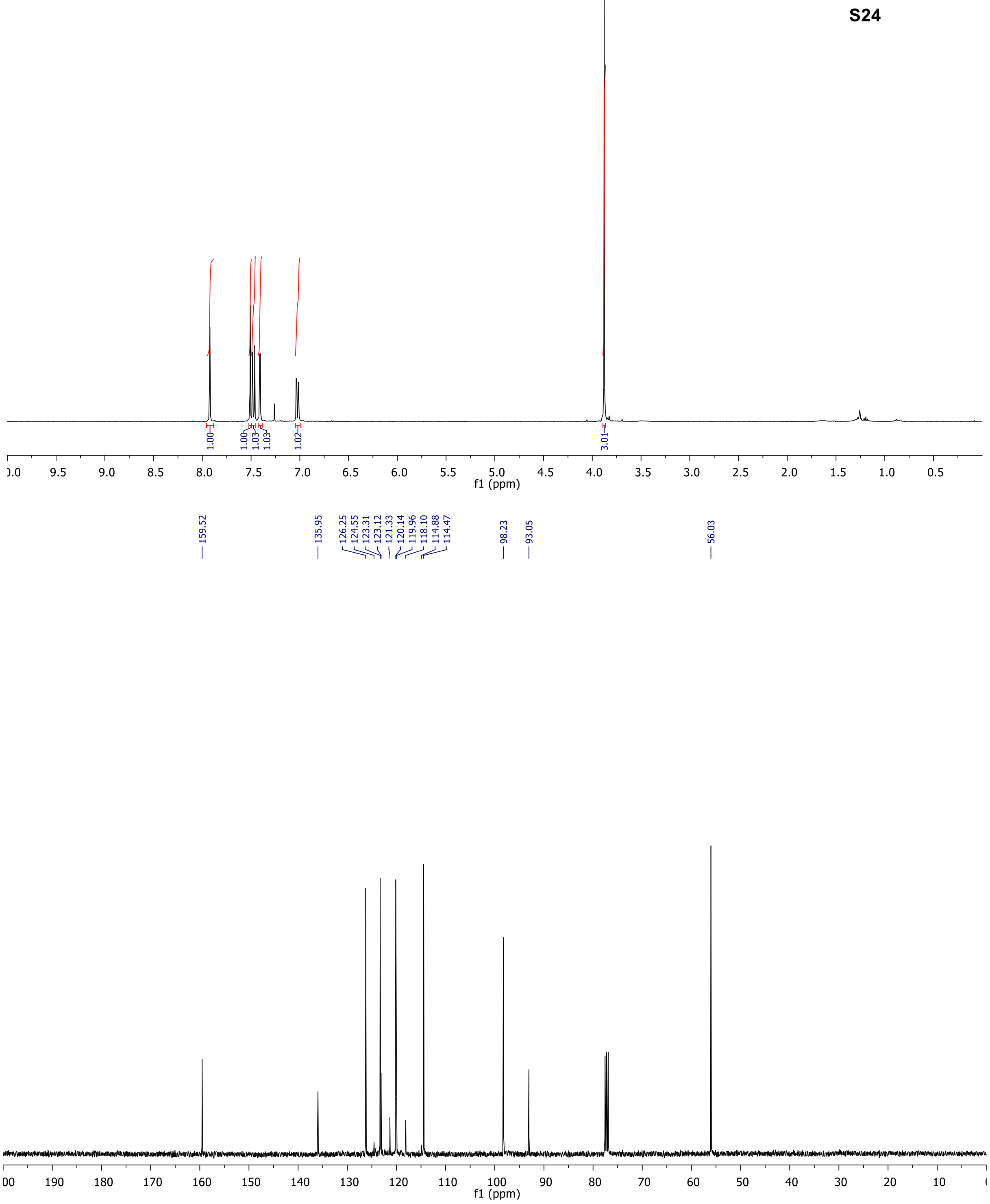

^{1}H NMR (400 MHz) and ^{13}C{^{1}H} NMR (100 MHz) spectra of **S24** ($CDCl_3$)

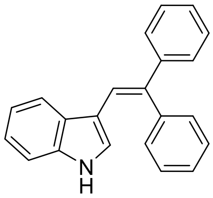

1a

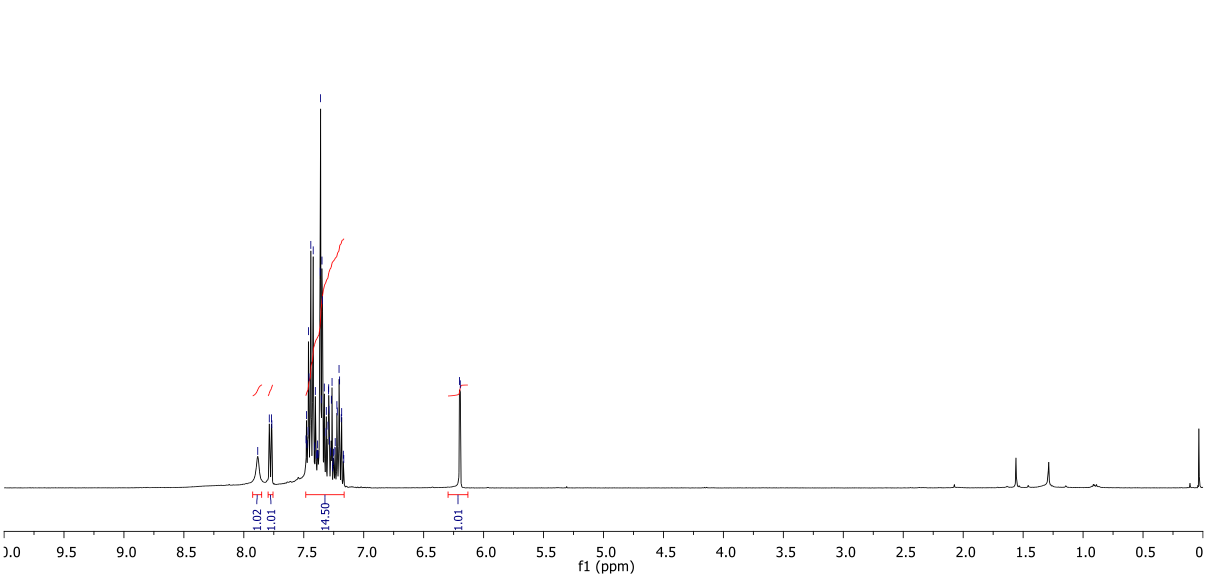

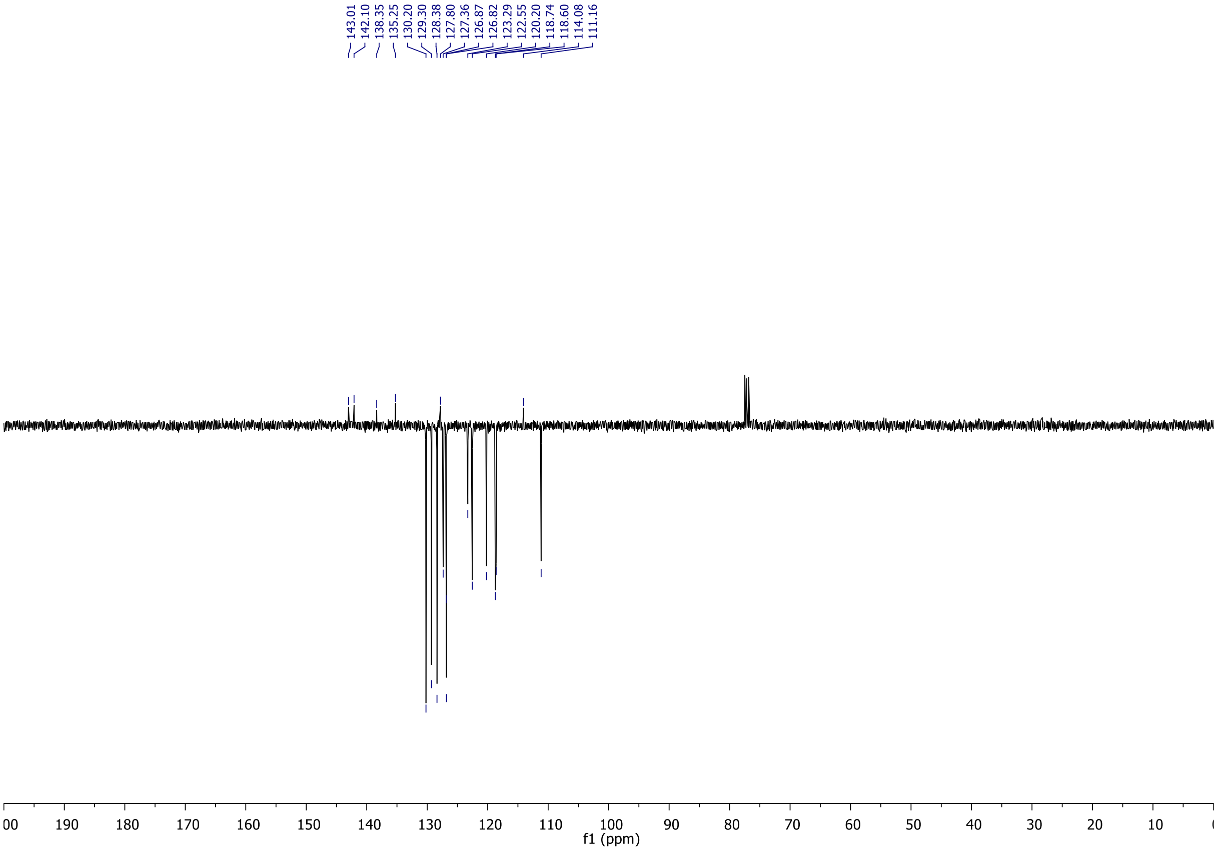

^{1}H NMR (400 MHz) and ^{13}C{^{1}H} (APT) NMR (100 MHz) spectra of **1a** ($CDCl_3$)

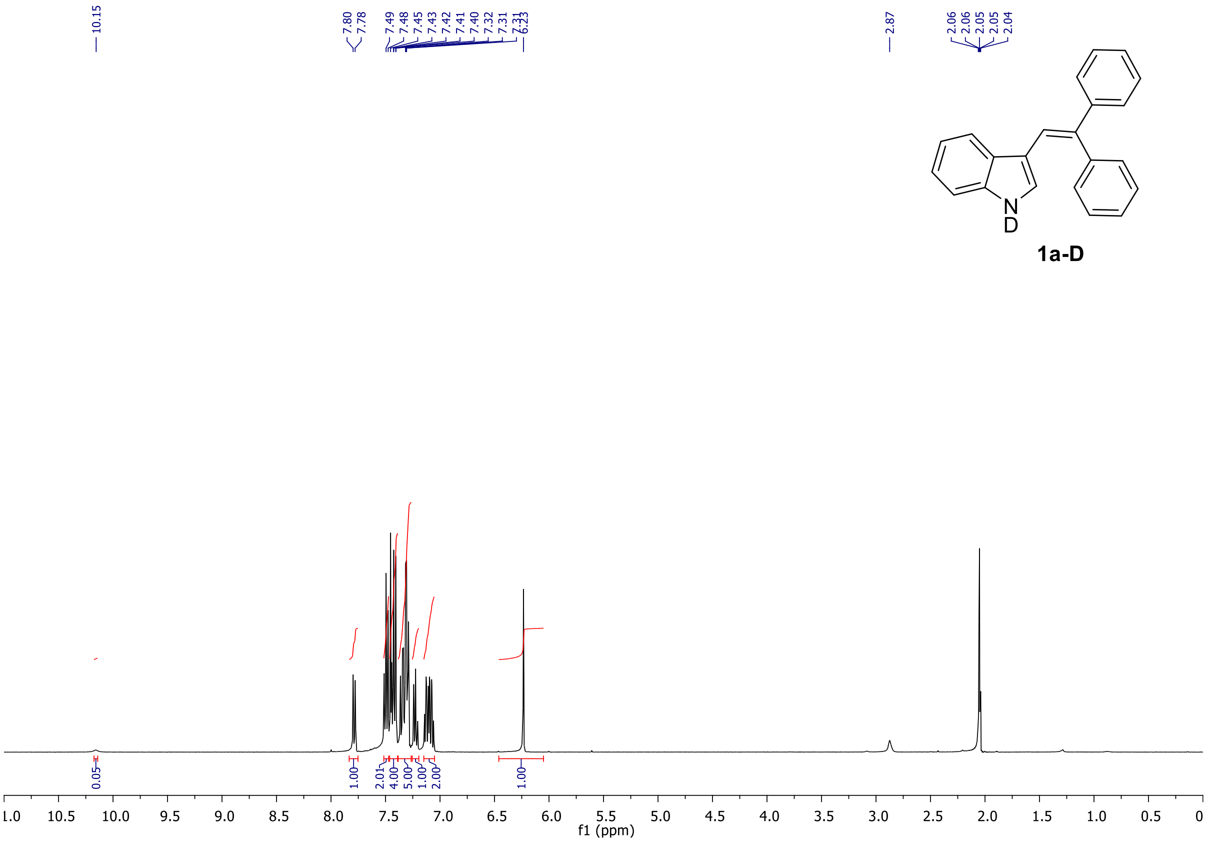

^{1}H NMR (400 MHz) spectra of **1a-D** (Acetone-d_6)

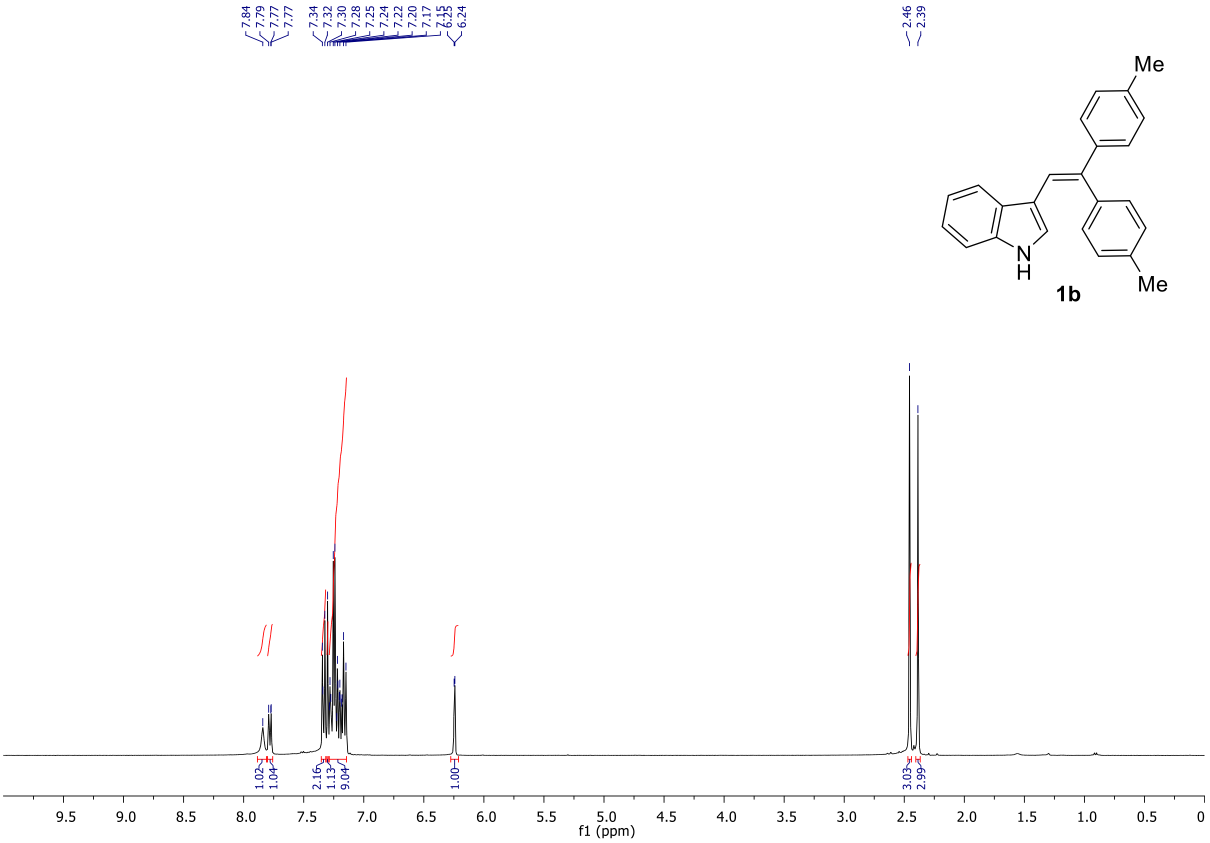

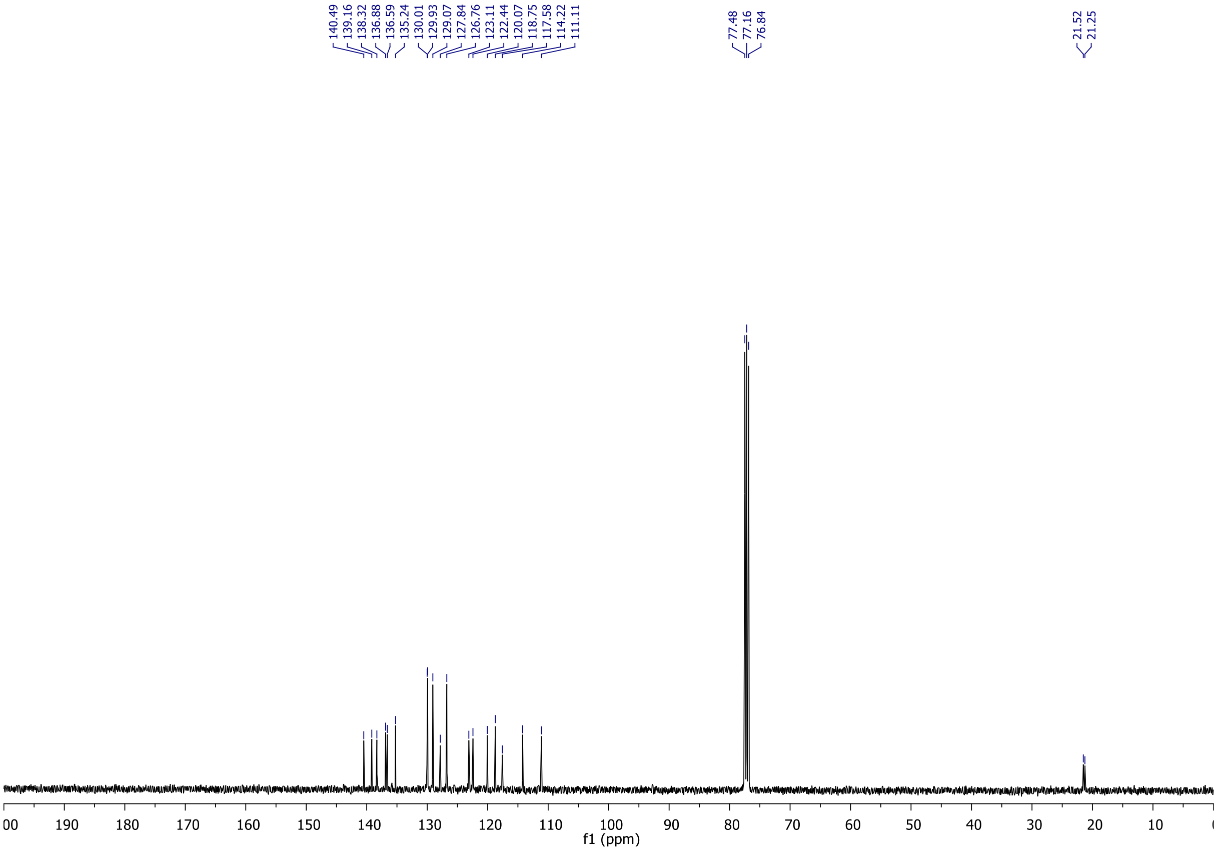

^{1}H NMR (400 MHz) and $^{13}C\{^{1}H\}$ NMR (100 MHz) spectra of **1b** ($CDCl_3$)

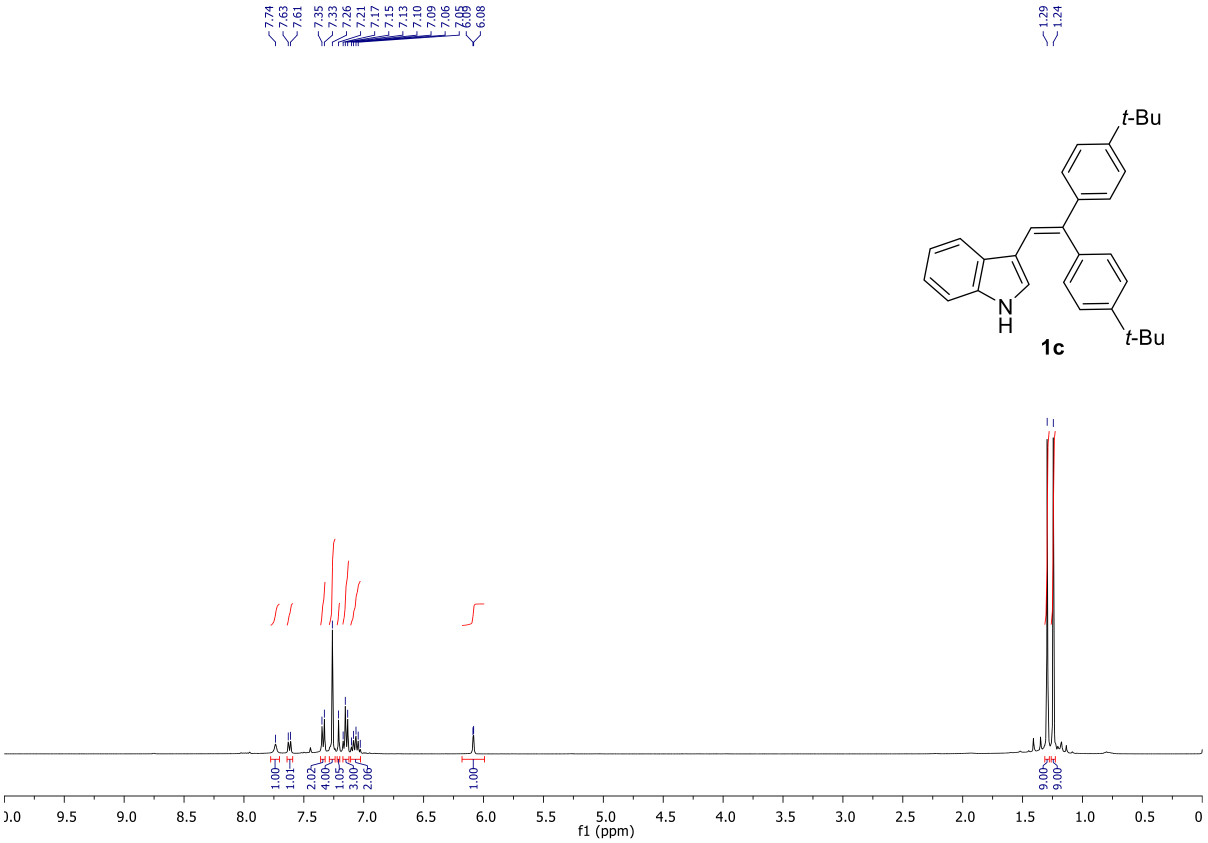

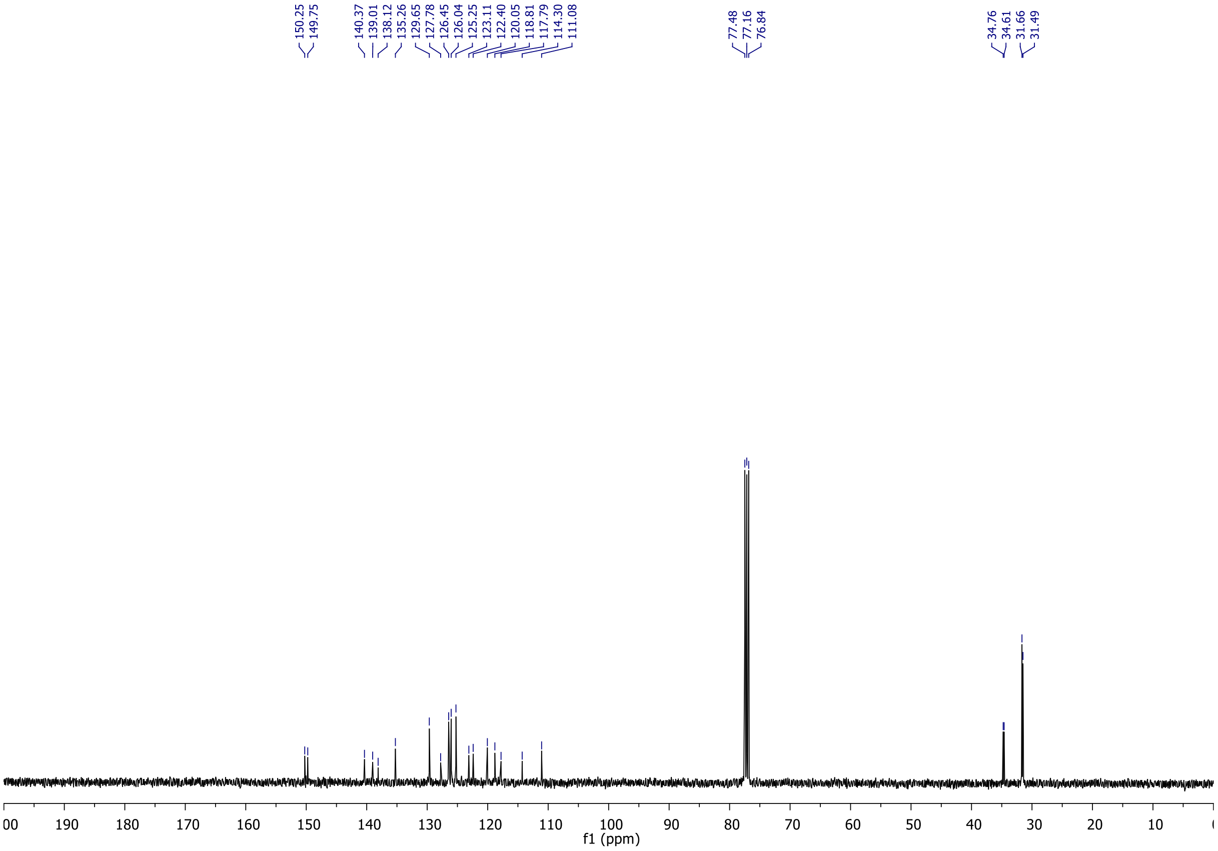

^{1}H NMR (400 MHz) and $^{13}C\{^{1}H\}$ NMR (100 MHz) spectra of **1c** ($CDCl_3$)

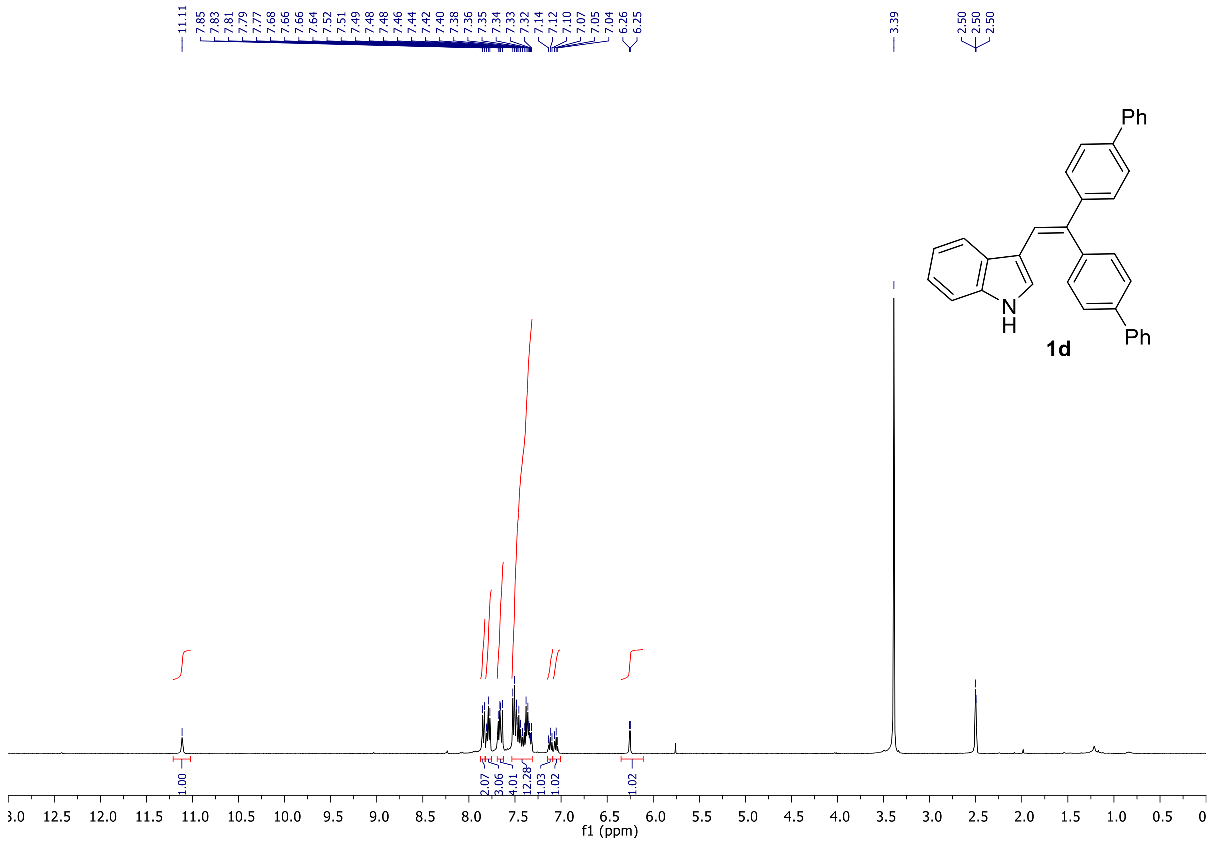

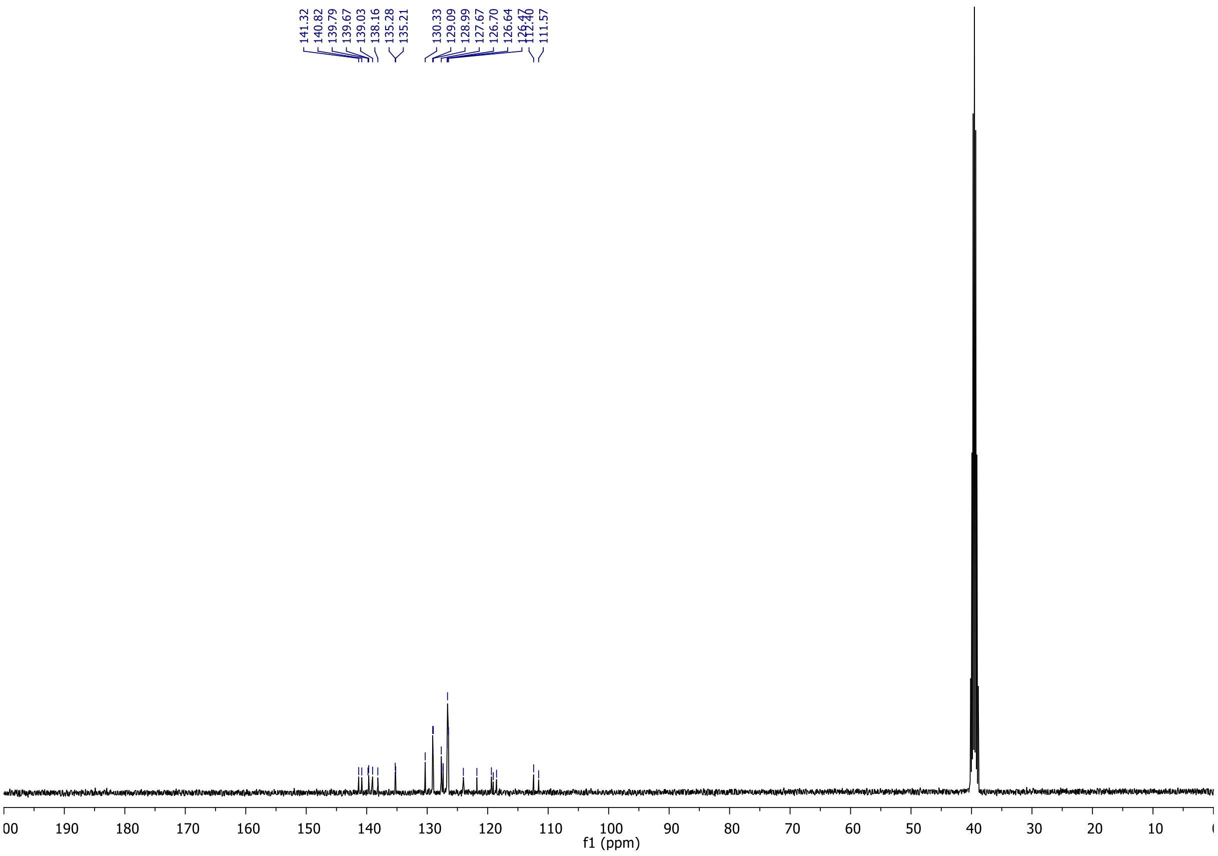

^{1}H NMR (400 MHz) and $^{13}C\{^{1}H\}$ NMR (100 MHz) spectra of **1d** (DMSO-d_6)

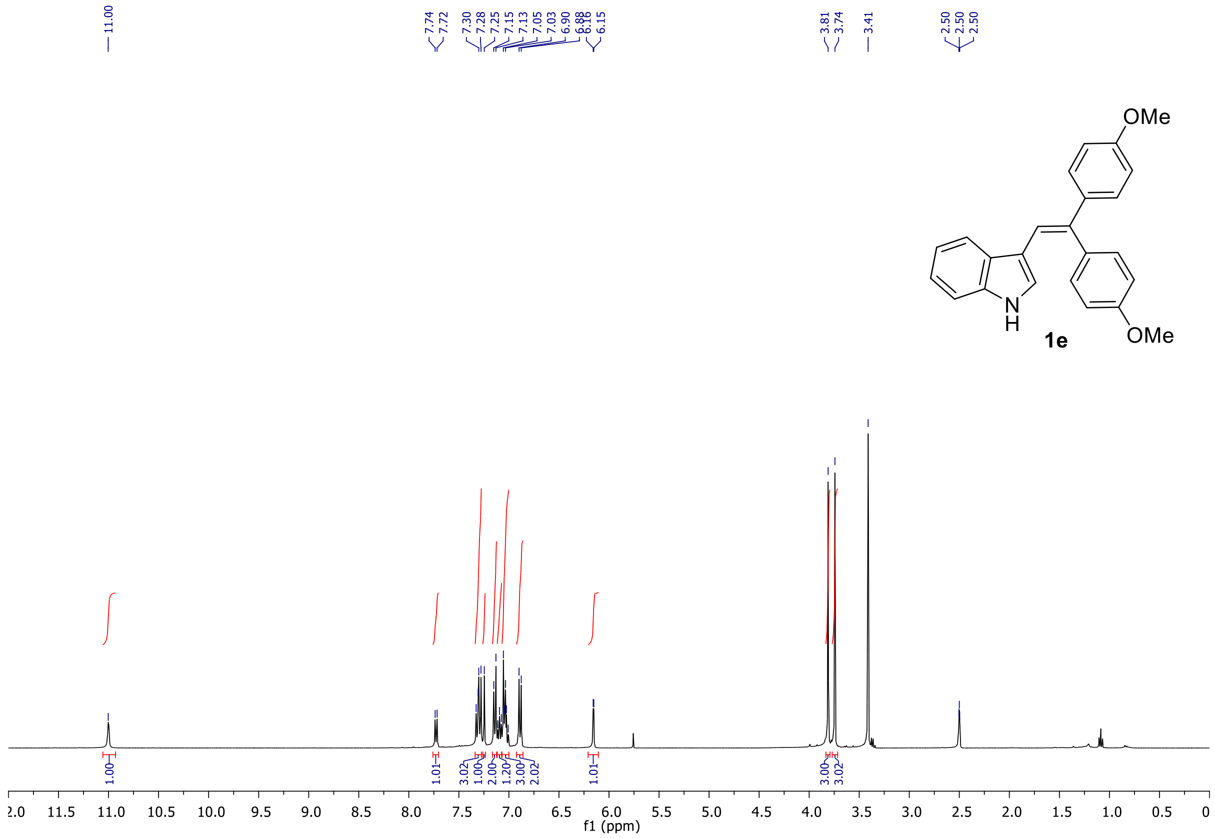

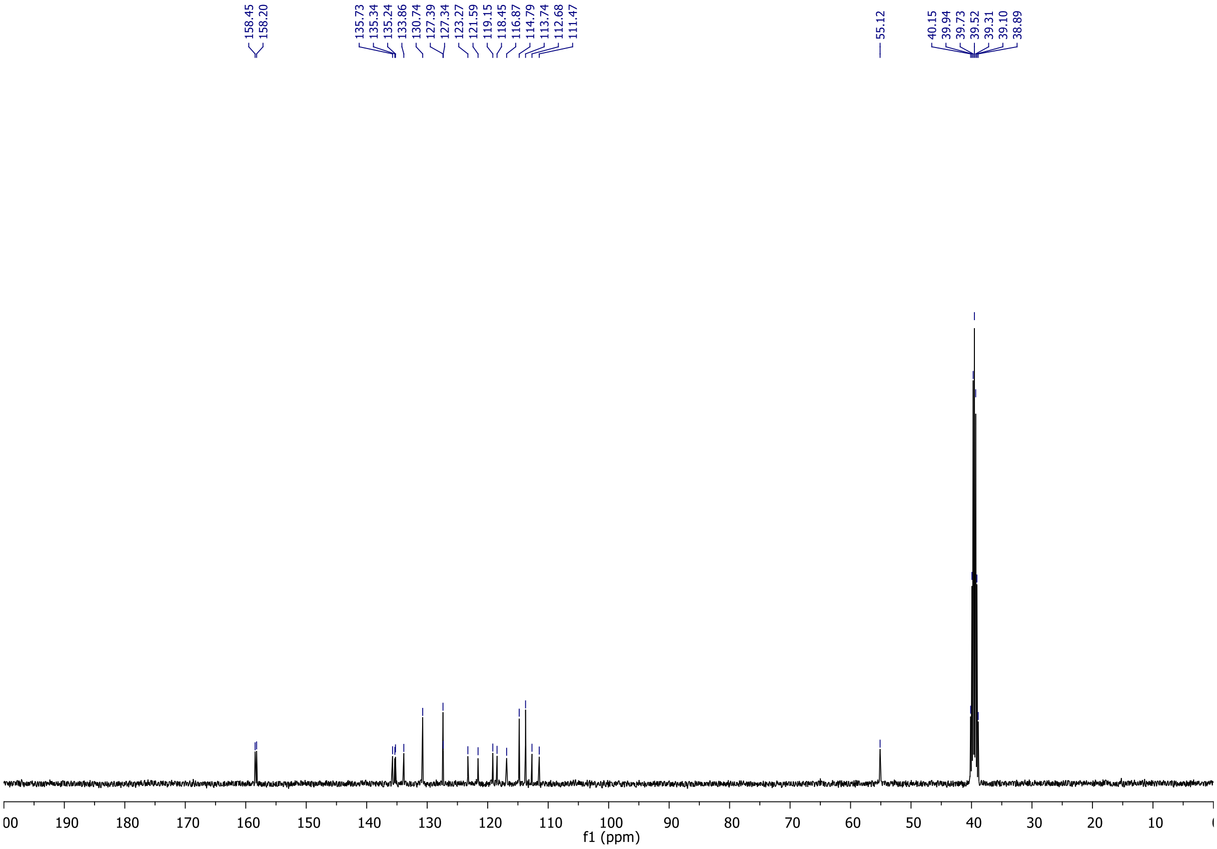

^{1}H NMR (400 MHz) and ^{13}C{^{1}H} NMR (100 MHz) spectra of **1e** (DMSO-d_6)

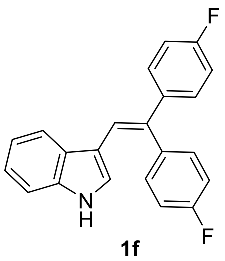

1f

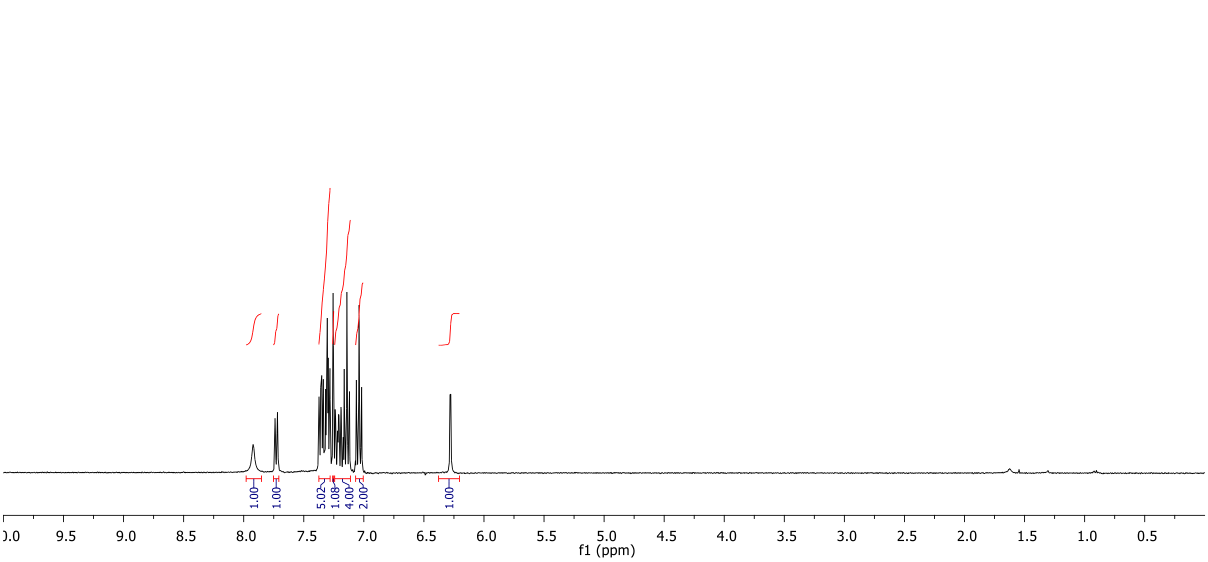

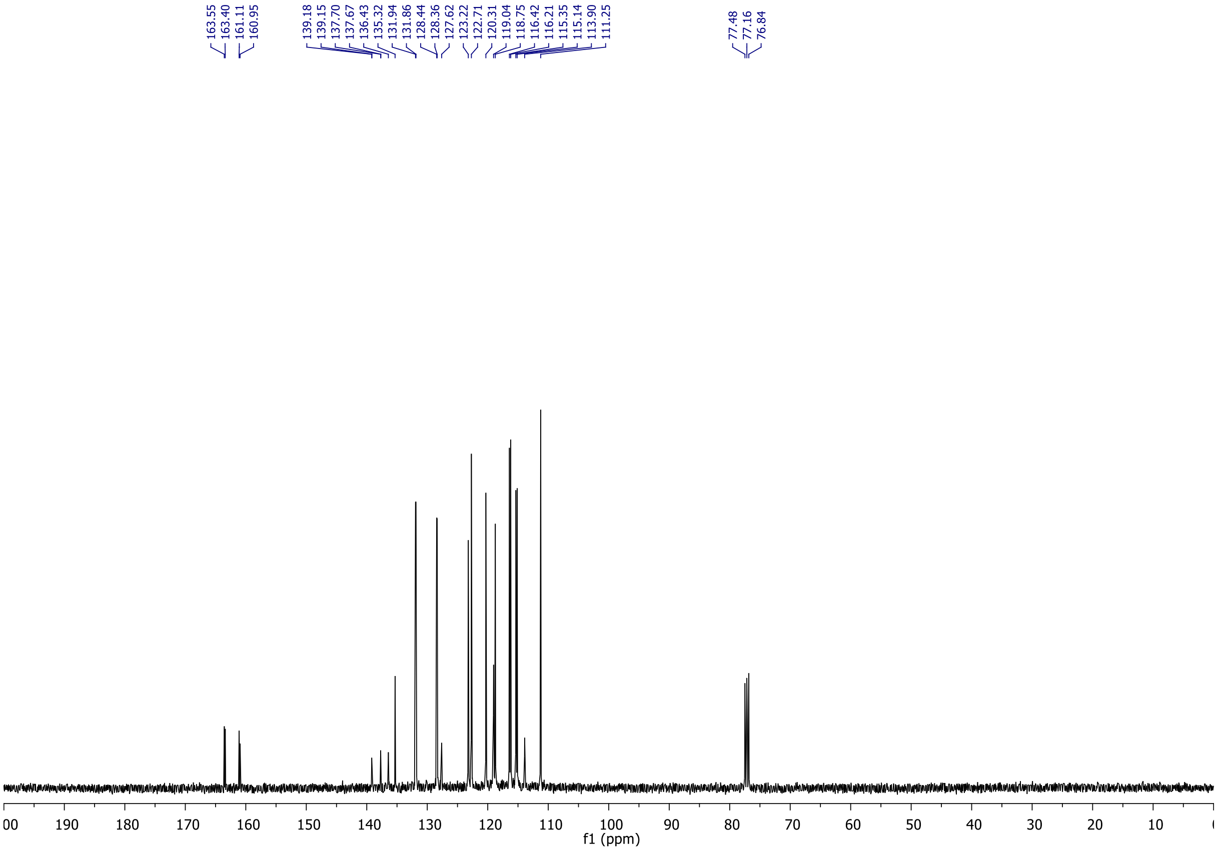

^{1}H NMR (400 MHz) and ^{13}C{^{1}H} NMR (100 MHz) spectra of **1f** ($CDCl_3$)

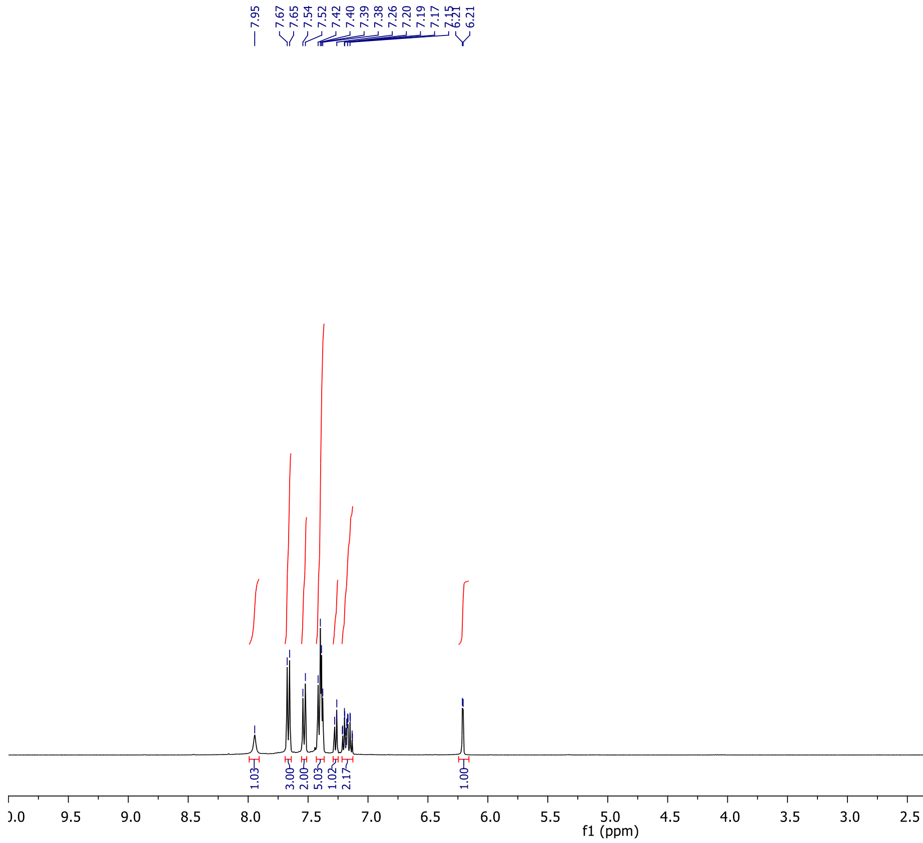

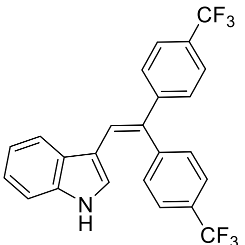

1g

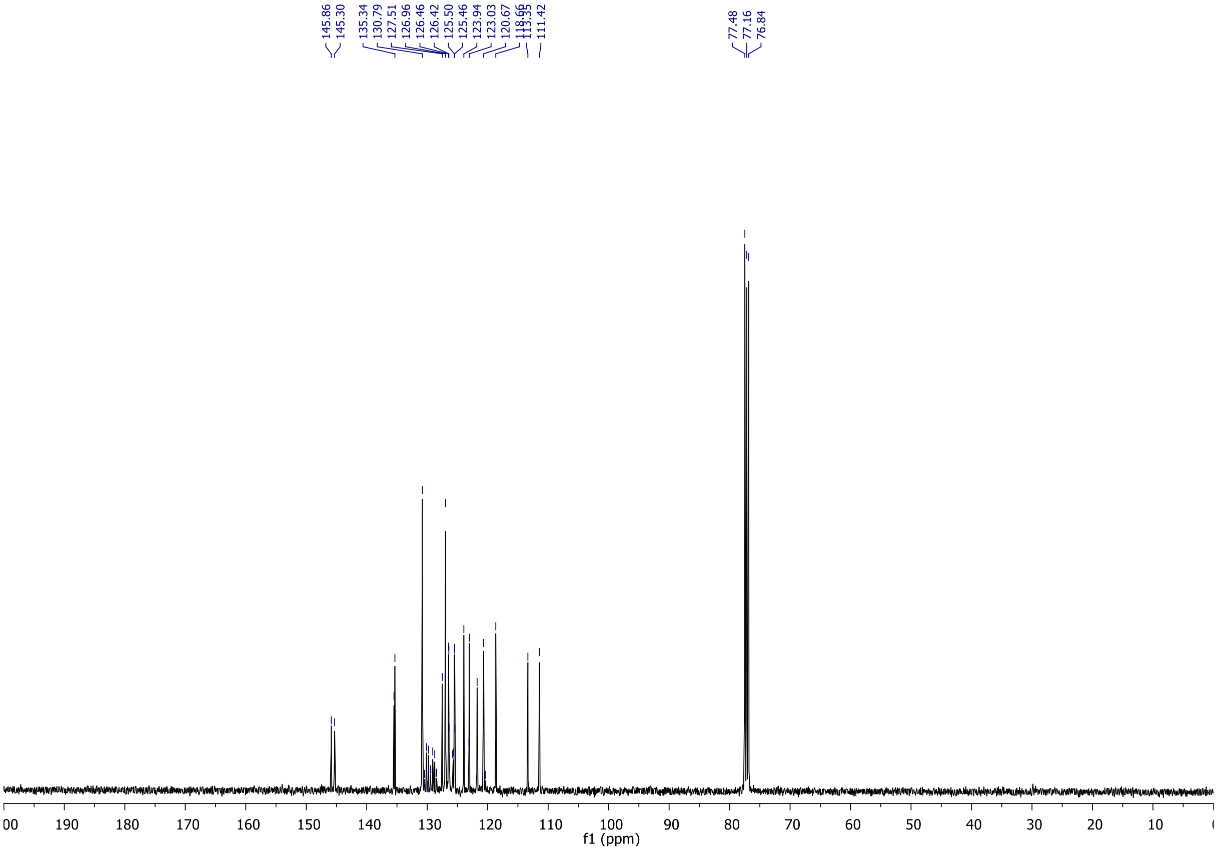

^{1}H NMR (400 MHz) and $^{13}C\{^{1}H\}$ NMR (100 MHz) spectra of **1g** ($CDCl_3$)

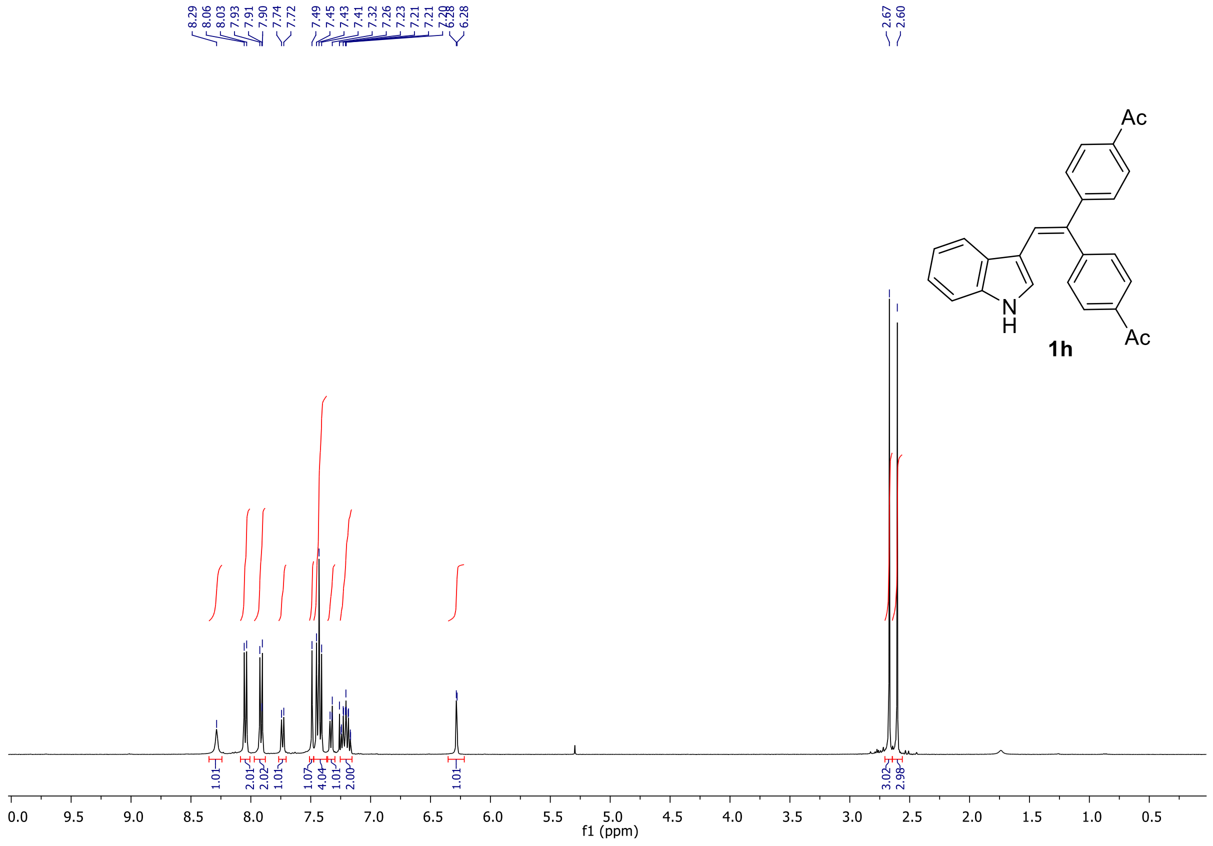

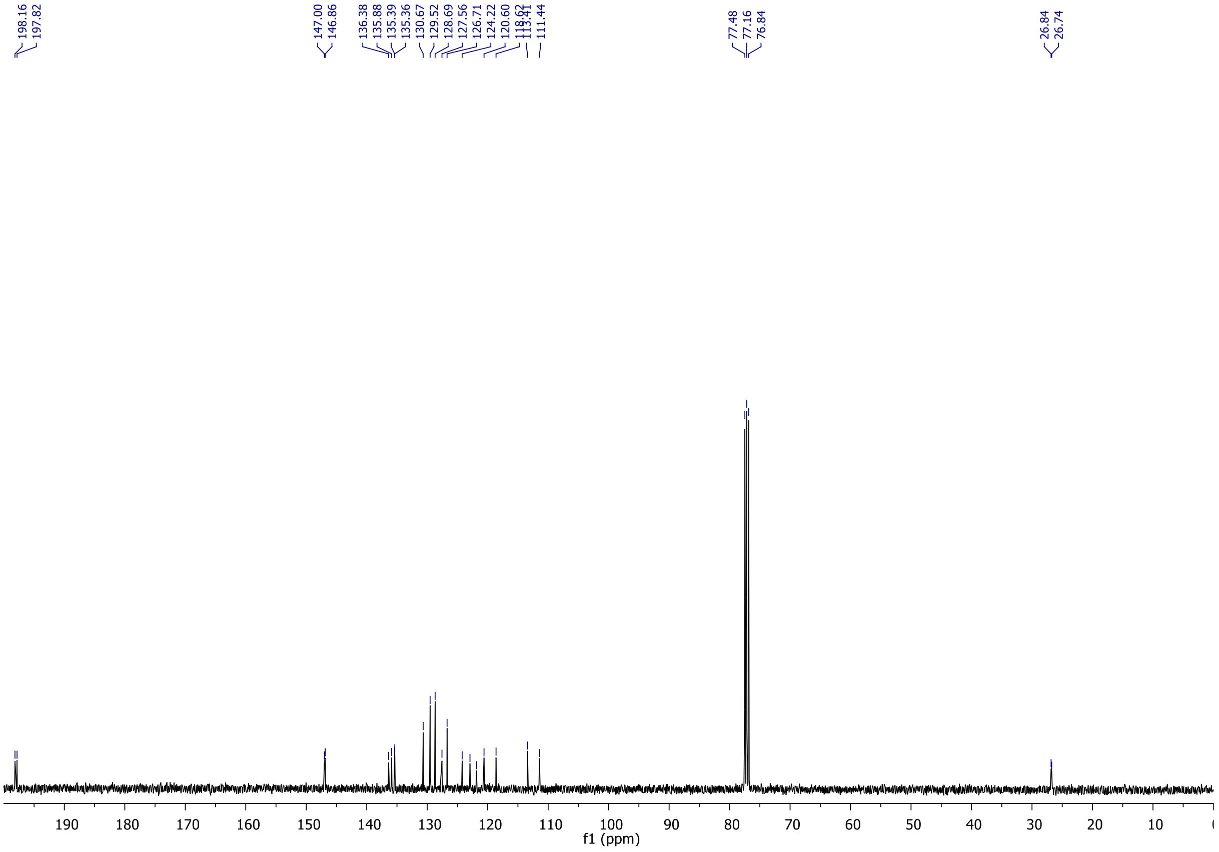

^{1}H NMR (400 MHz) and $^{13}C\{^{1}H\}$ NMR (100 MHz) spectra of **1h** ($CDCl_3$)

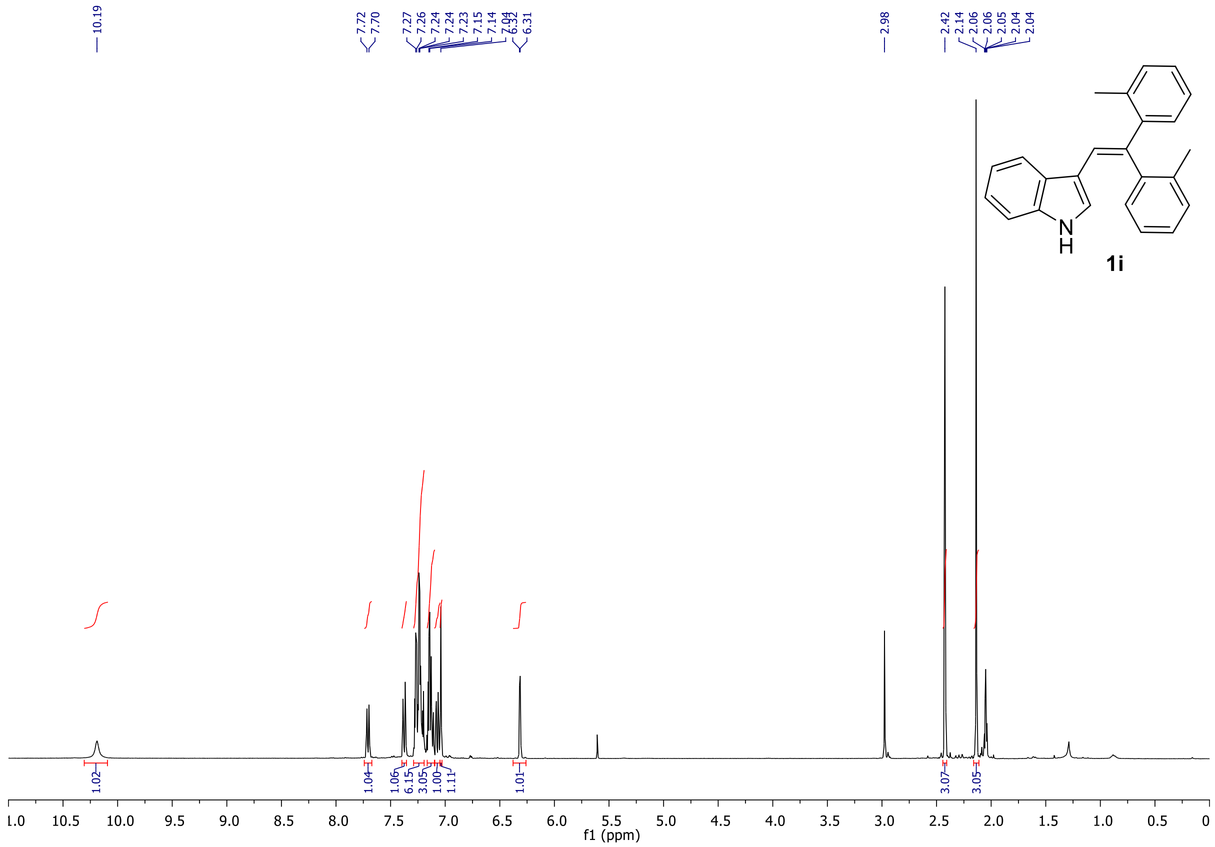

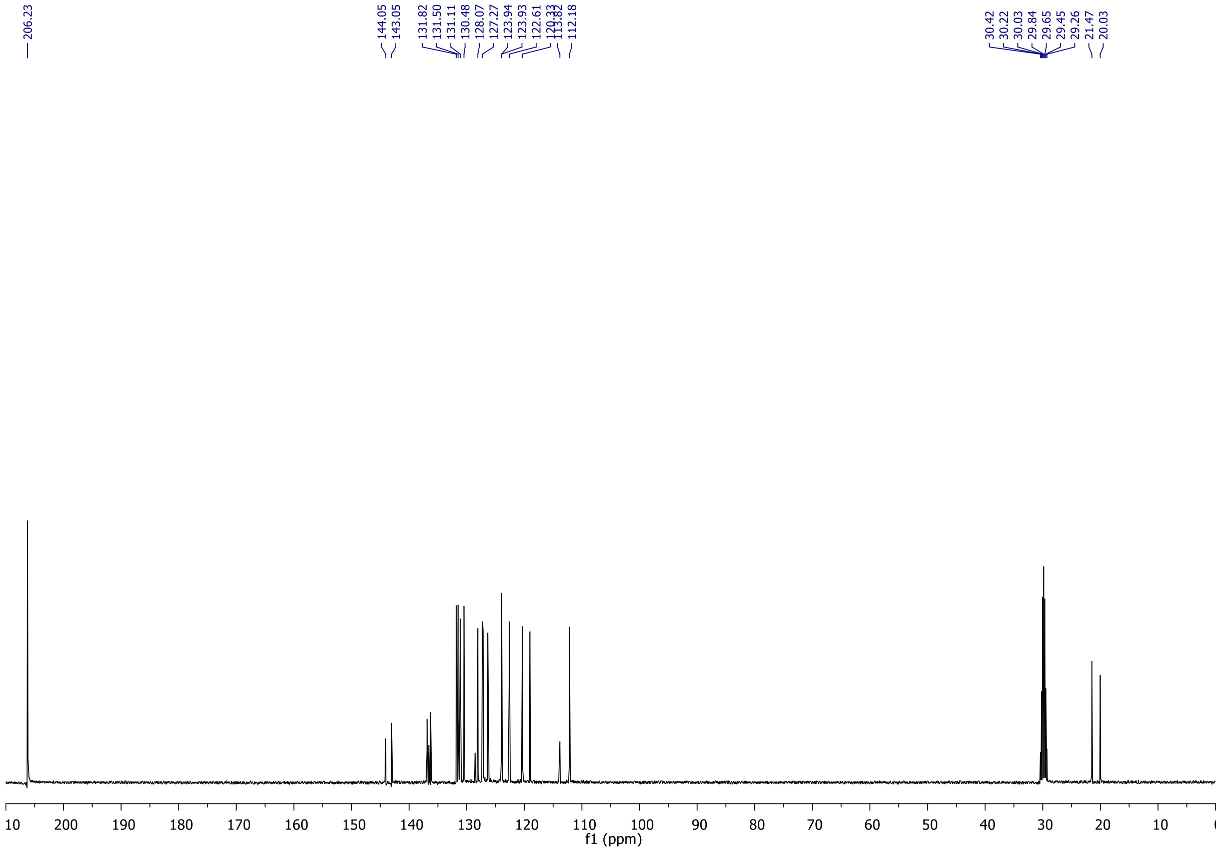

^{1}H NMR (400 MHz) and ^{13}C{^{1}H} NMR (100 MHz) spectra of **1i** (Acetone-d_6)

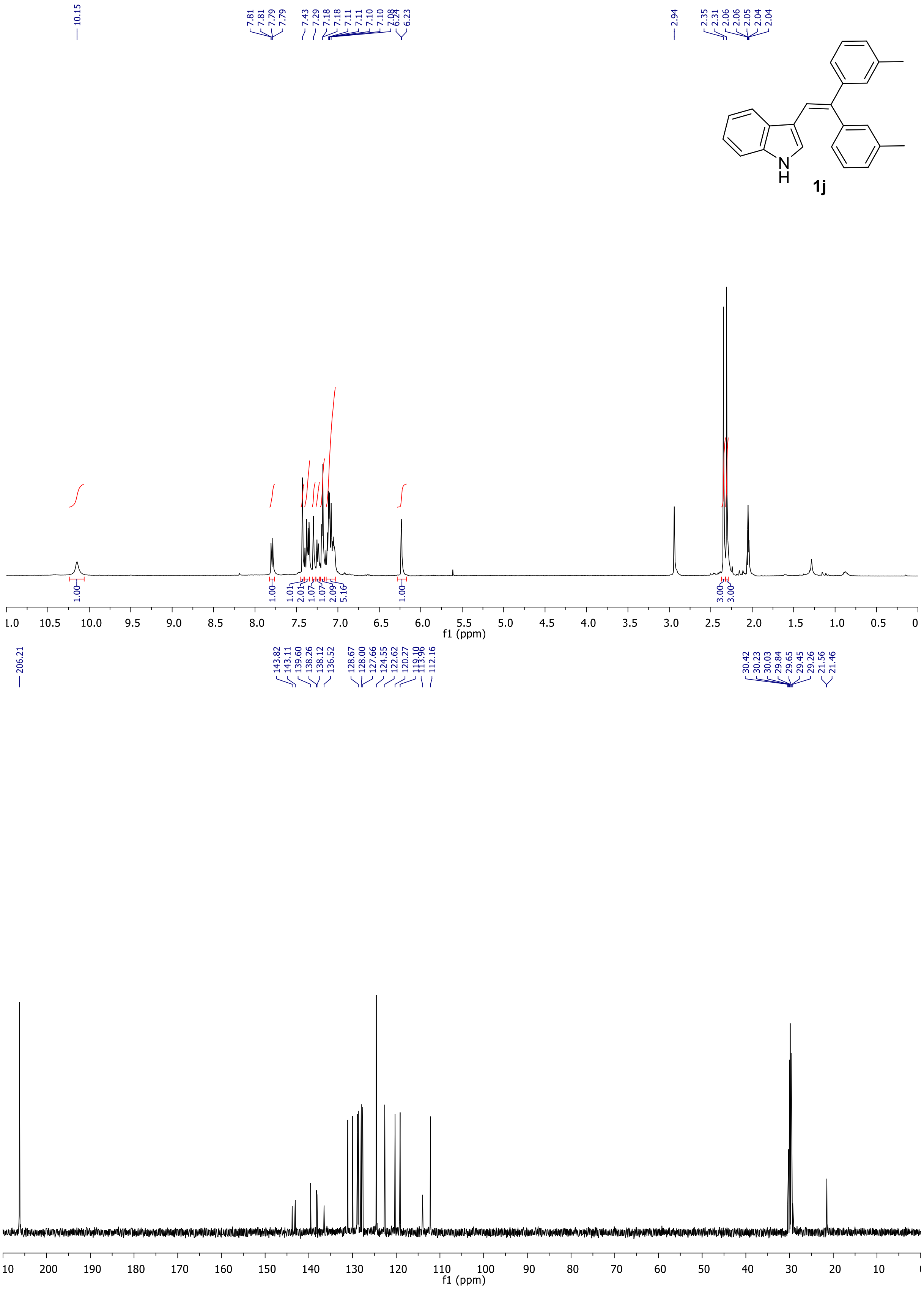

^{1}H NMR (400 MHz) and ^{13}C{^{1}H} NMR (100 MHz) spectra of **1j** (Acetone-d_6)

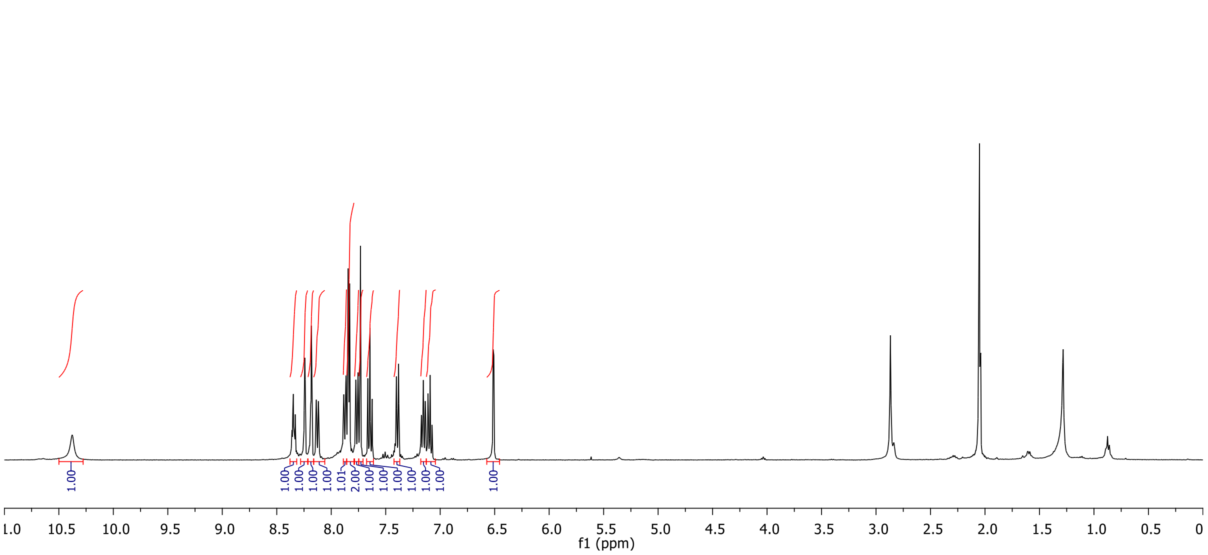

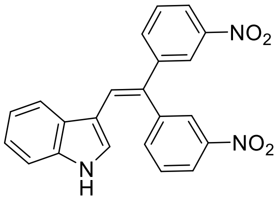

1k

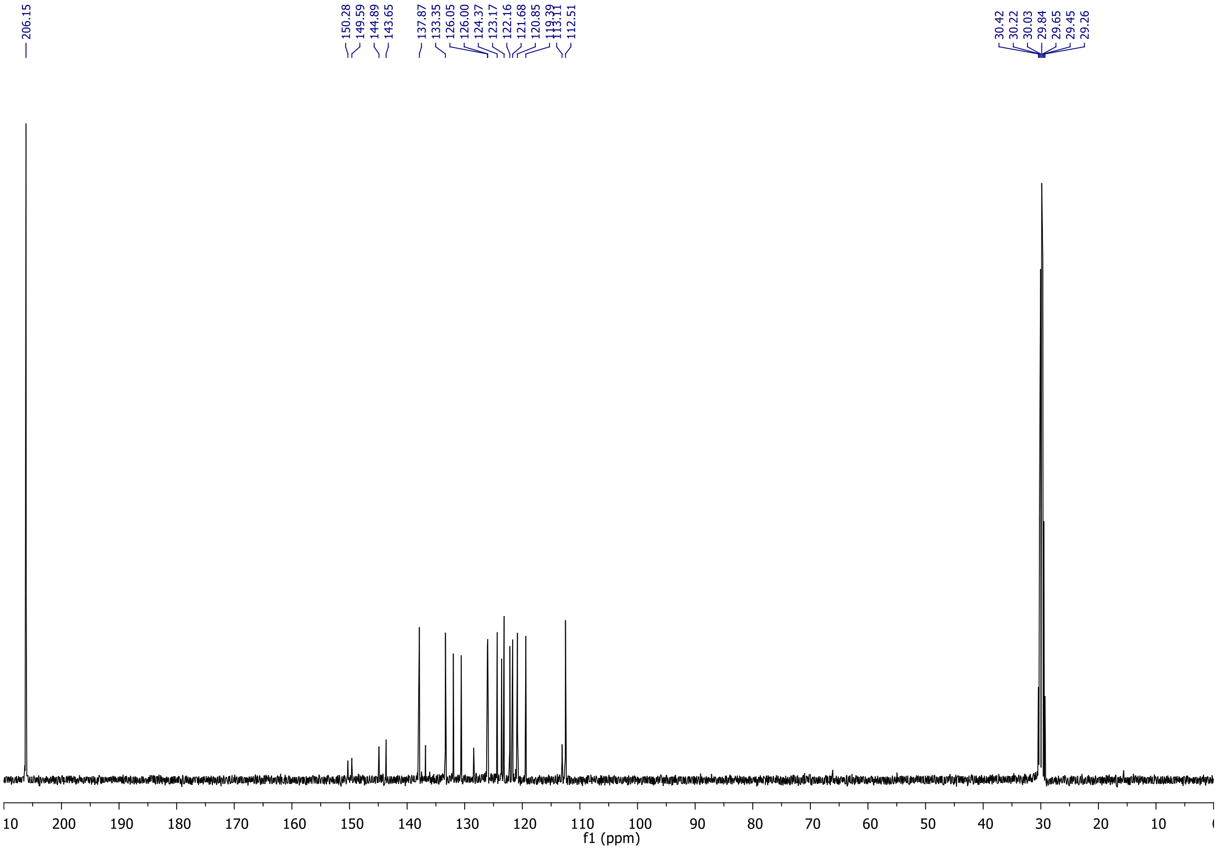

^{1}H NMR (400 MHz) and $^{13}C\{^{1}H\}$ NMR (100 MHz) spectra of **1k** (Acetone-d_6)

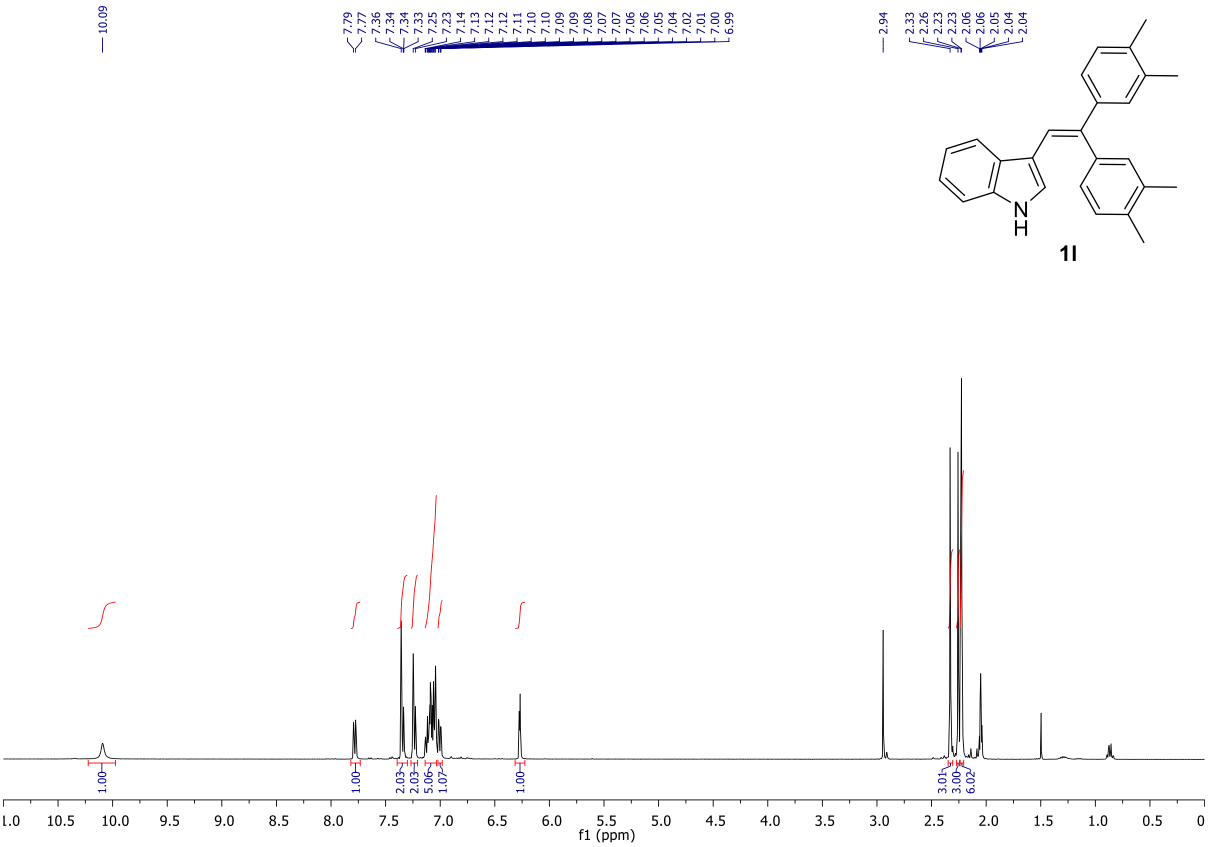

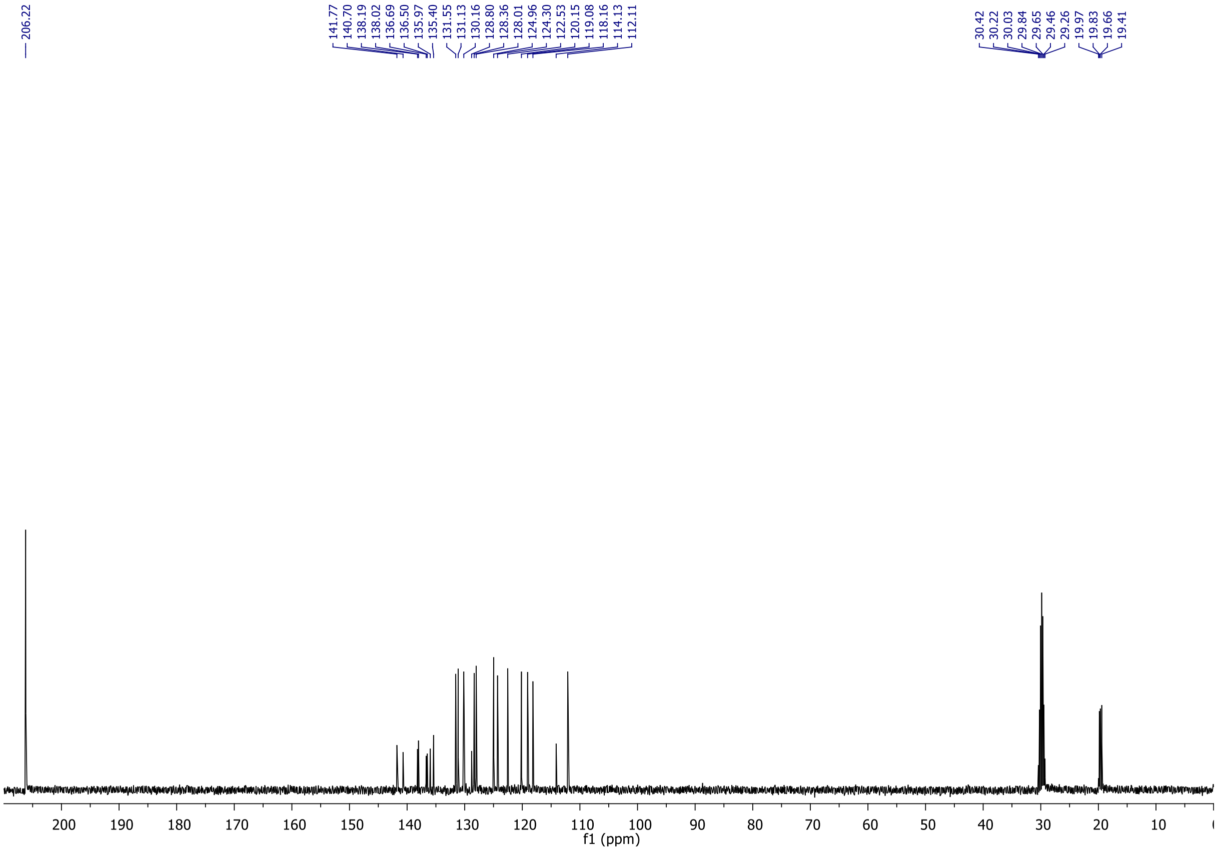

^{1}H NMR (400 MHz) and $^{13}C\{^{1}H\}$ NMR (100 MHz) spectra of **1l** (Acetone-d_6)

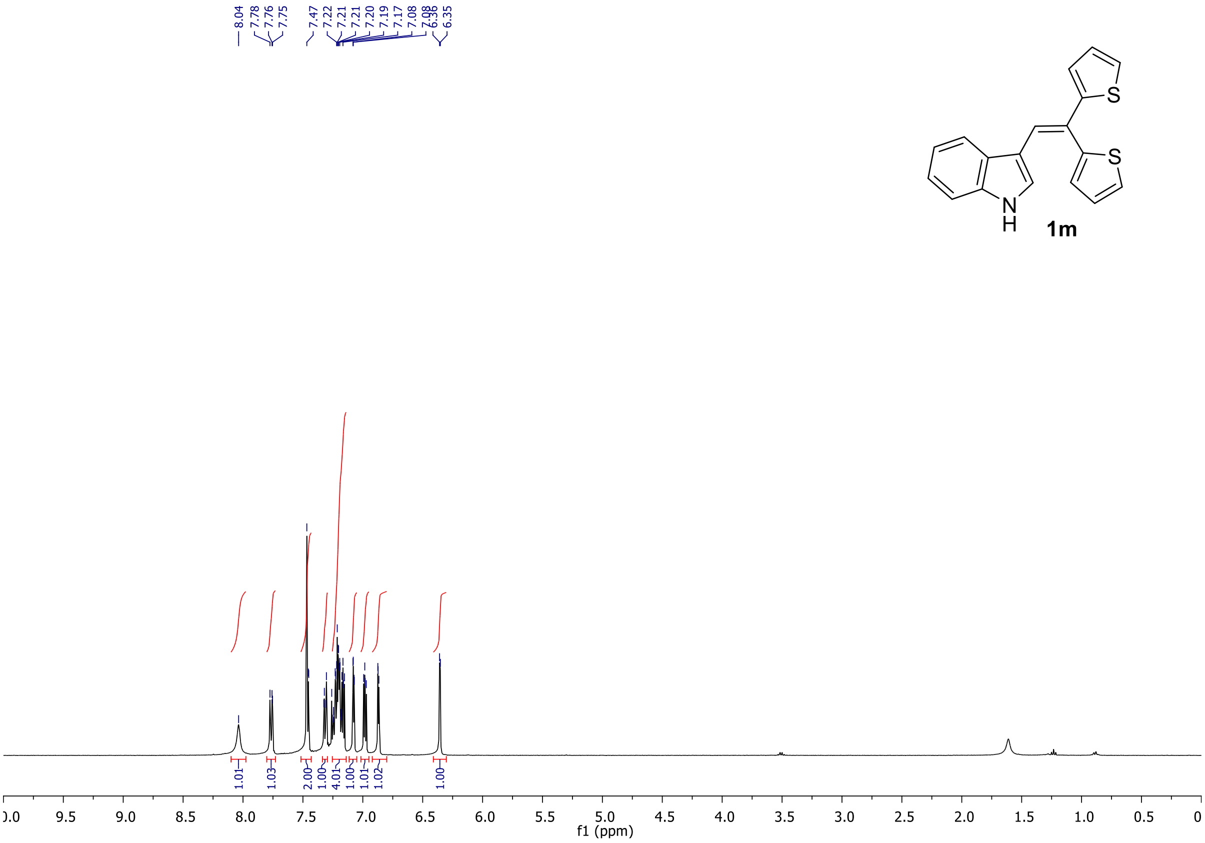

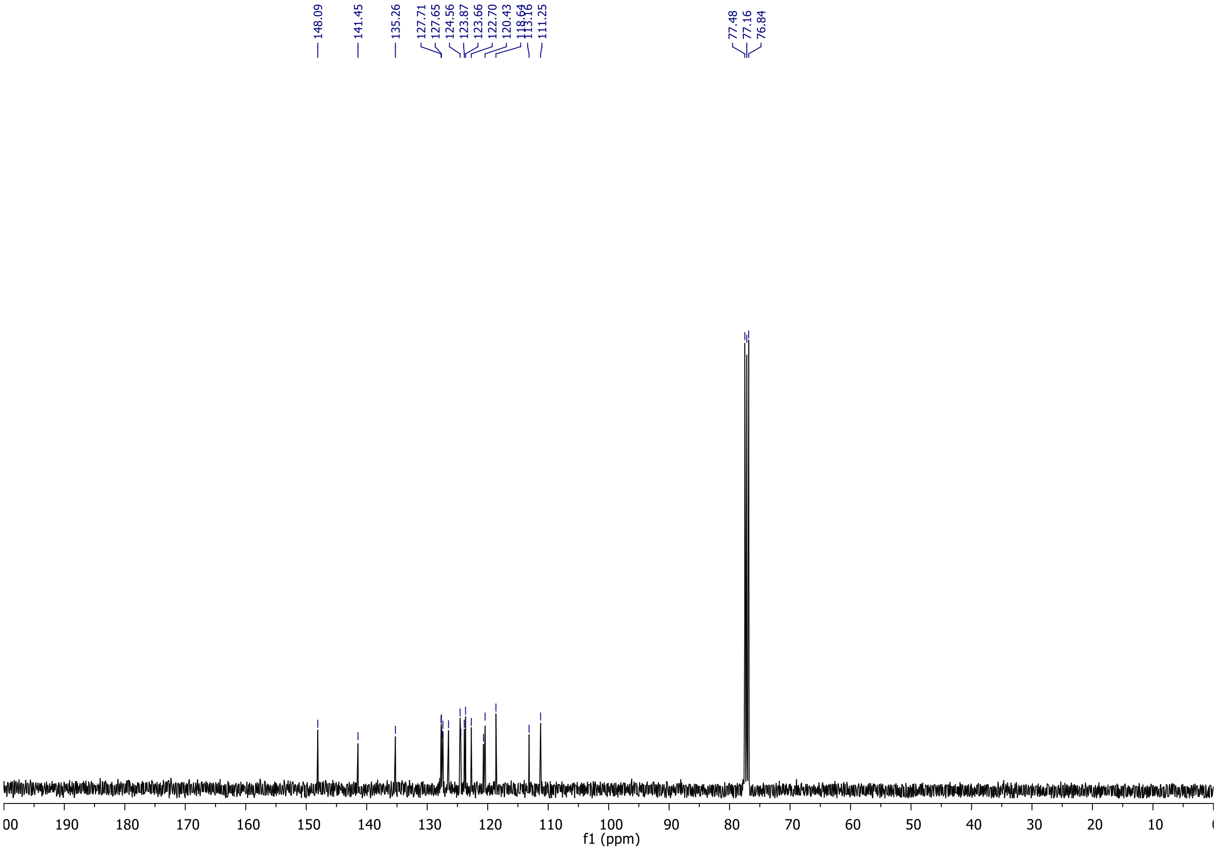

^{1}H NMR (400 MHz) and $^{13}C\{^{1}H\}$ NMR (100 MHz) spectra of **1m** ($CDCl_3$)

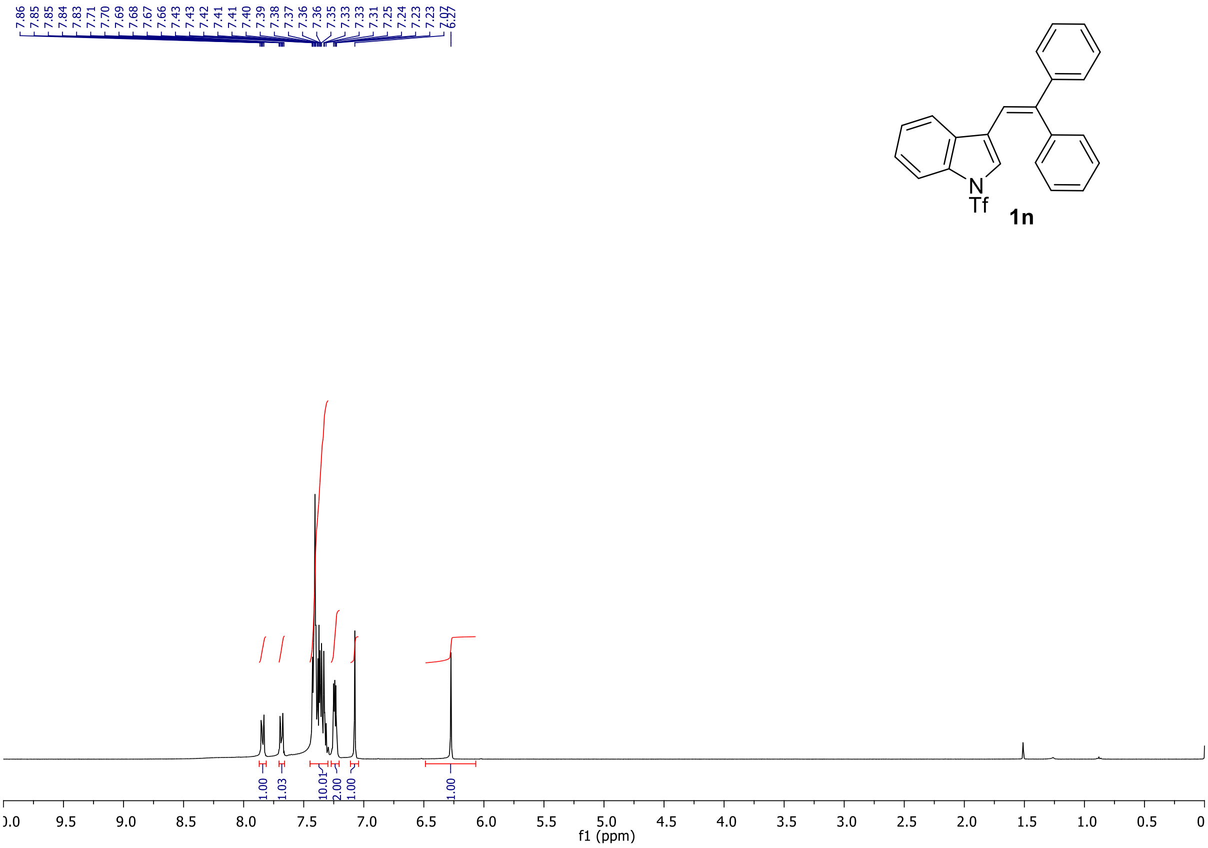

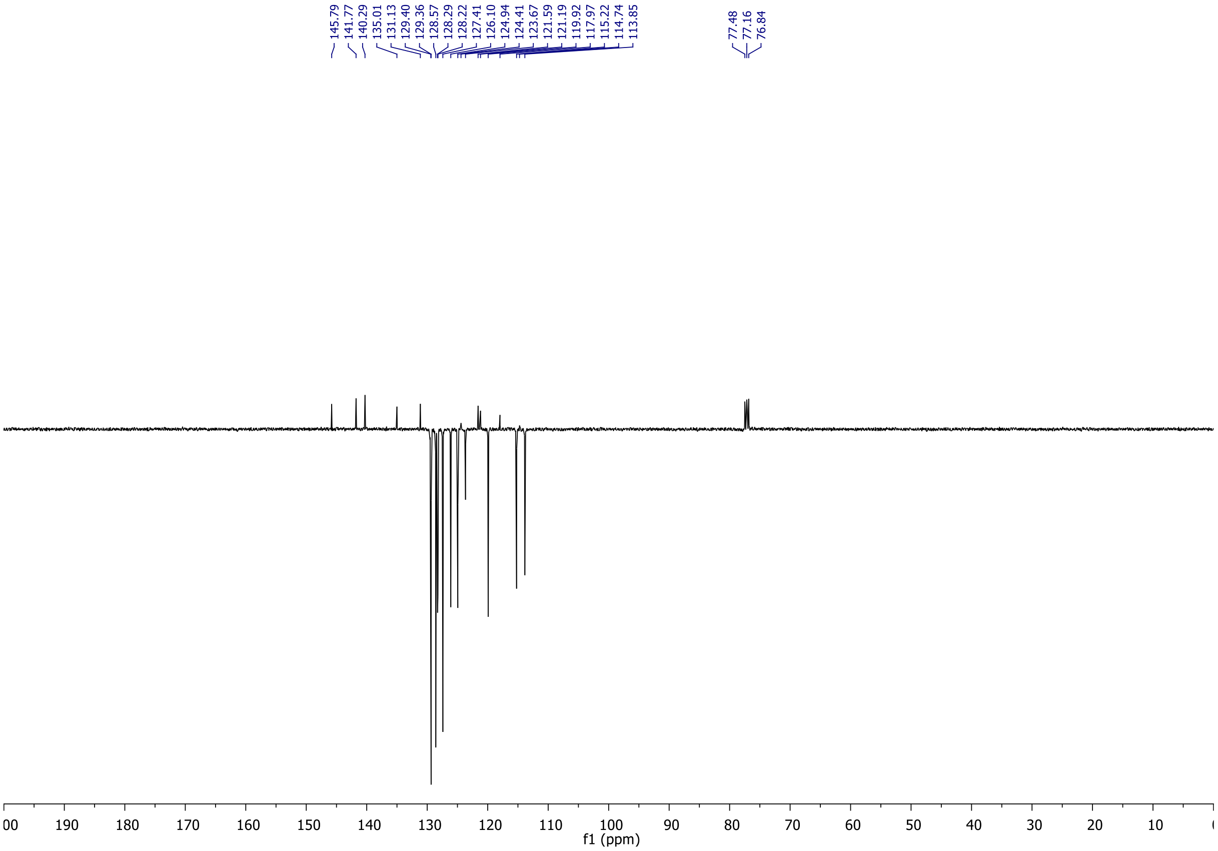

^{1}H NMR (400 MHz) and $^{13}C\{^{1}H\}$ (APT) NMR (100 MHz) spectra of **1n** ($CDCl_3$)

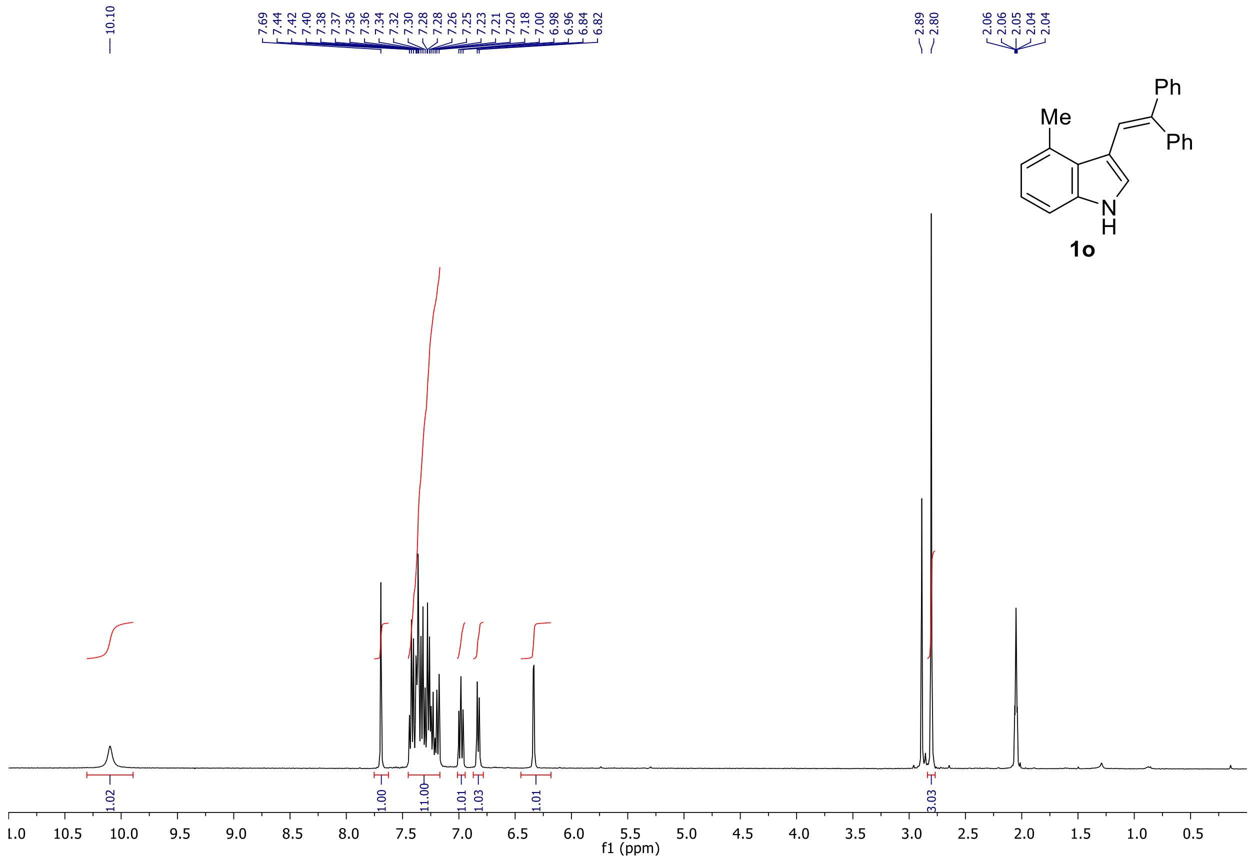

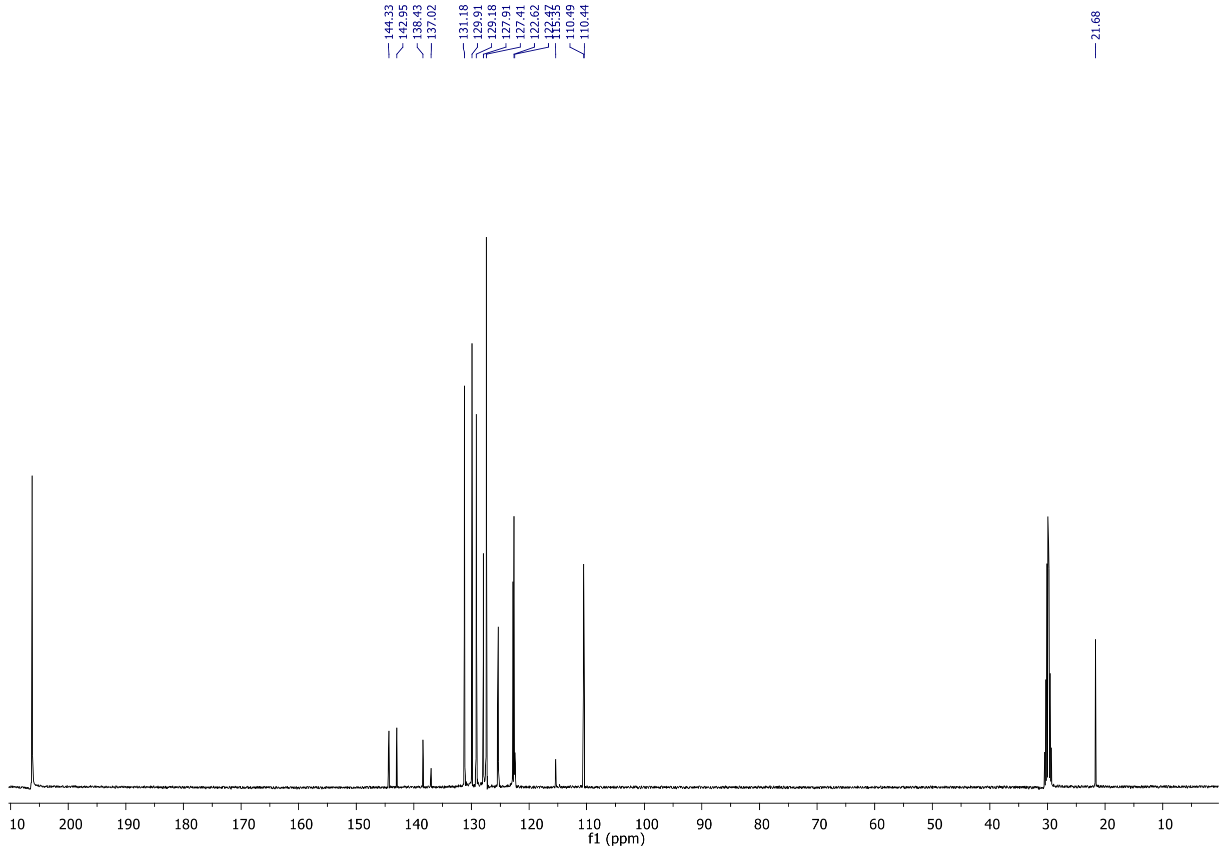

^{1}H NMR (400 MHz) and $^{13}C\{^{1}H\}$ NMR (100 MHz) spectra of **1o** (Acetone-d_6)

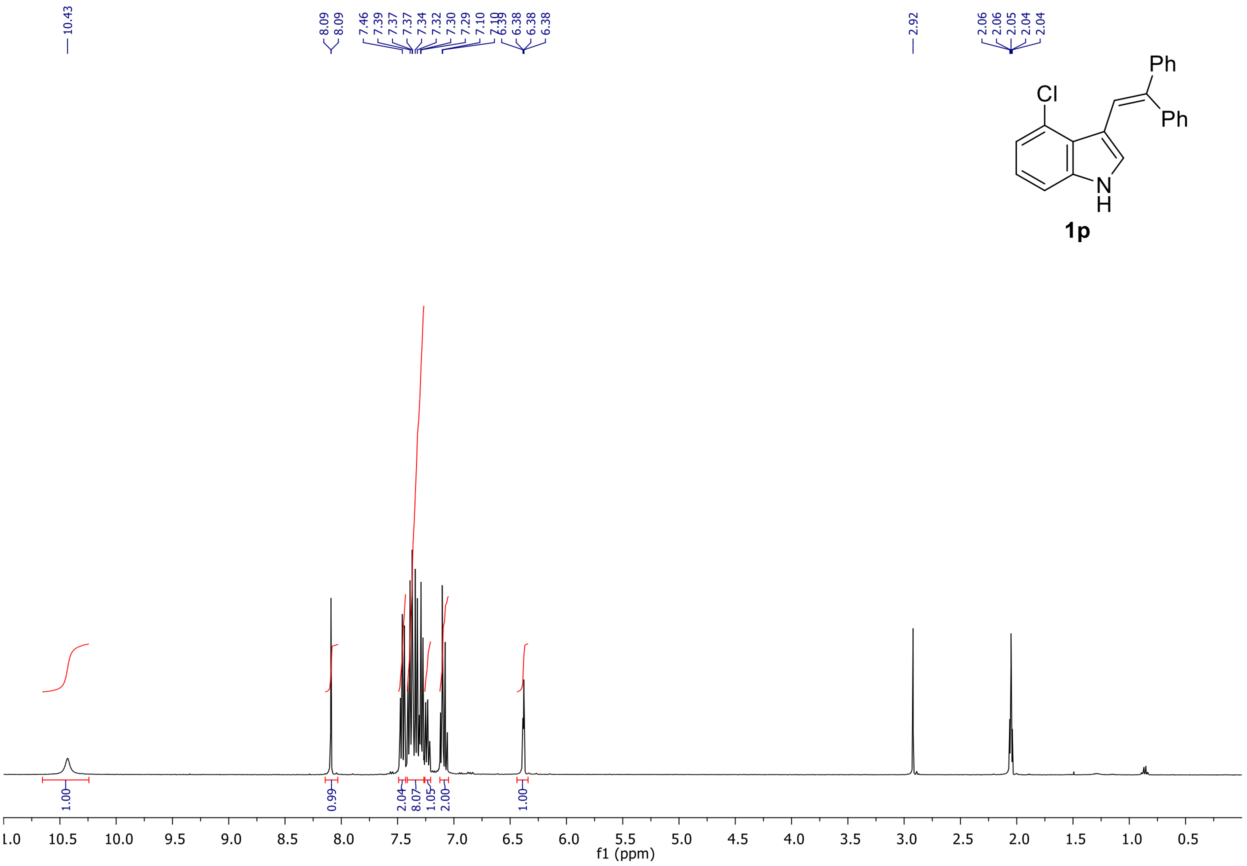

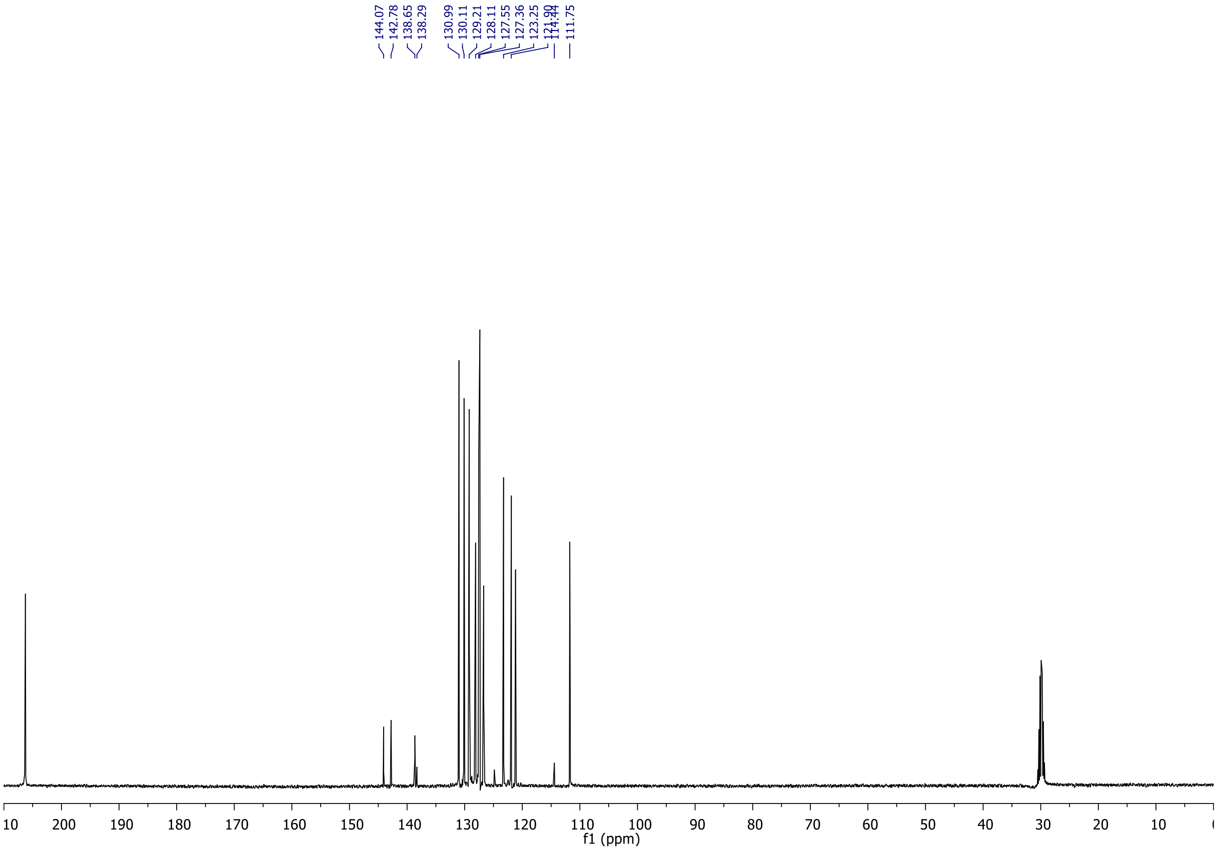

^{1}H NMR (400 MHz) and $^{13}C\{^{1}H\}$ NMR (100 MHz) spectra of **1p** (Acetone-d_6)

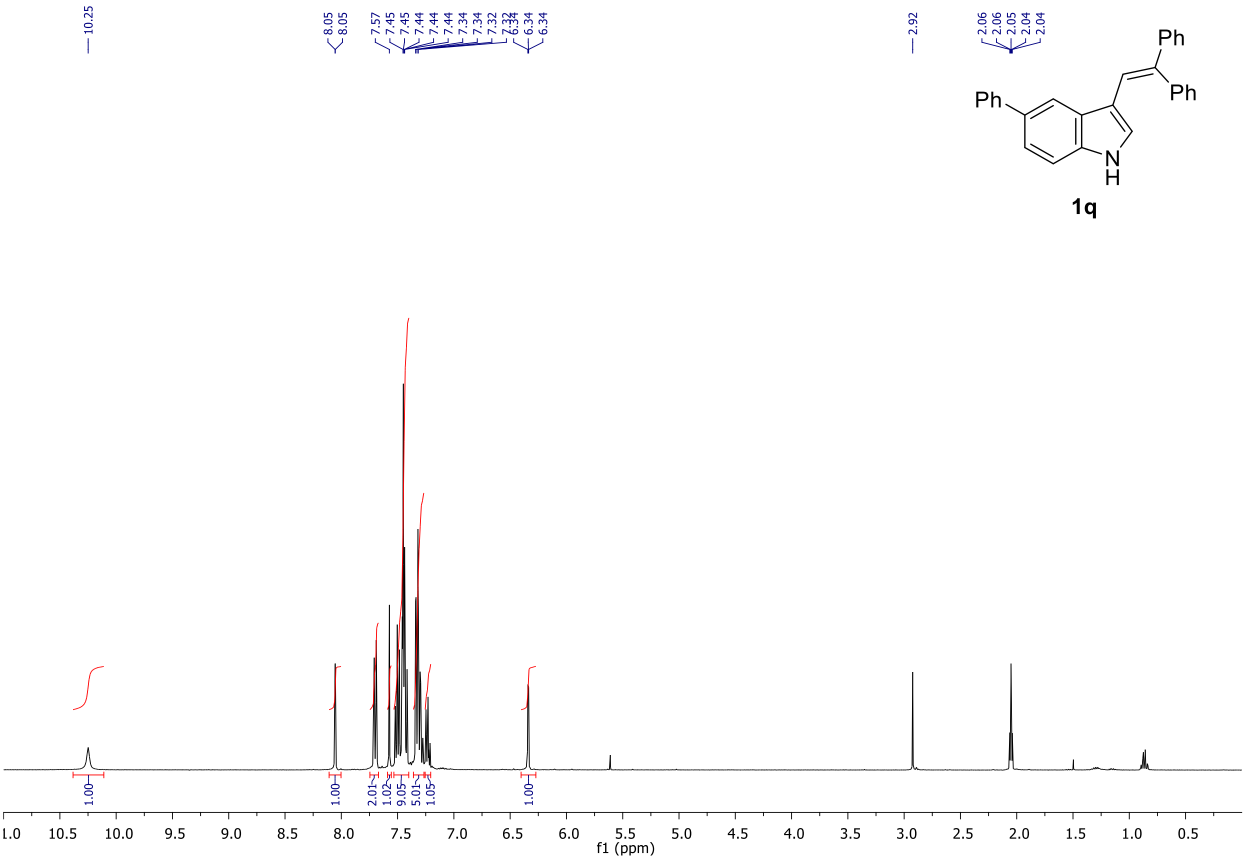

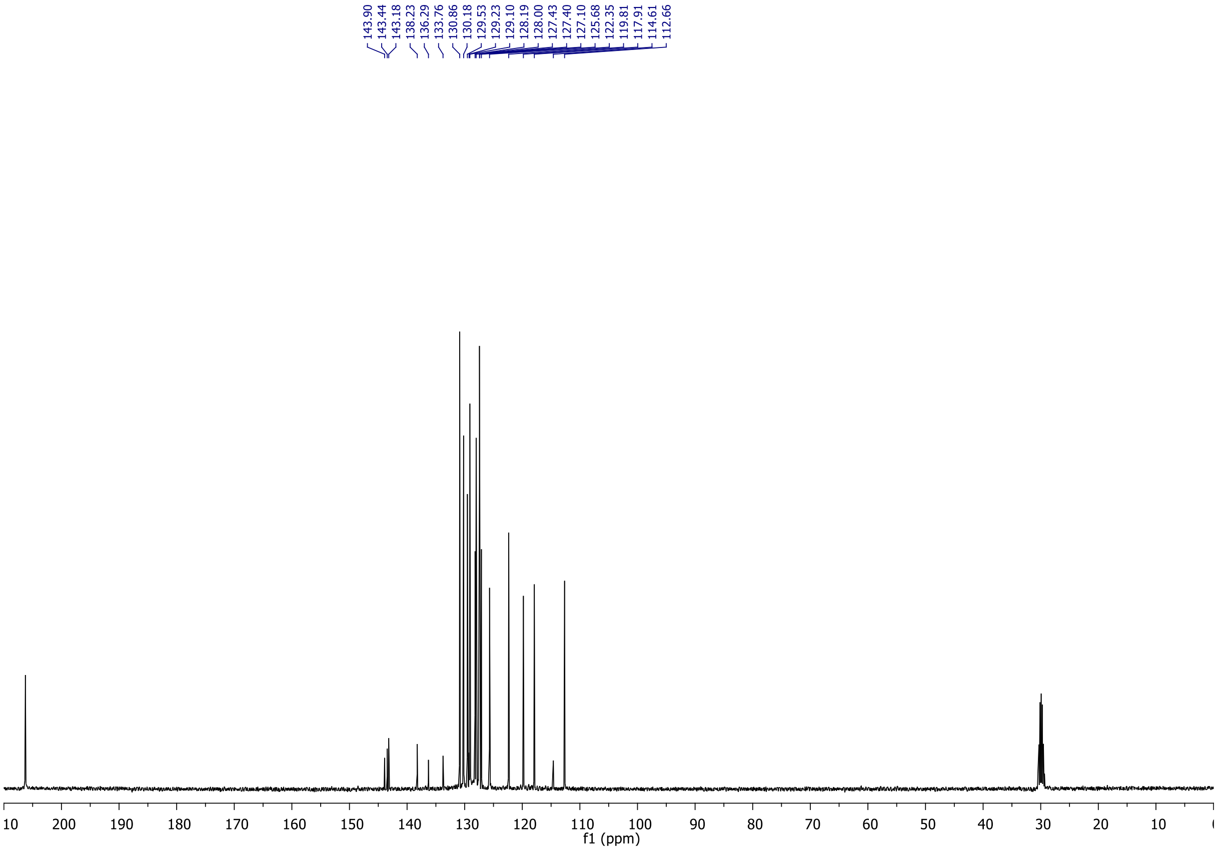

^{1}H NMR (400 MHz) and $^{13}C\{^{1}H\}$ NMR (100 MHz) spectra of **1q** (Acetone-d_6)

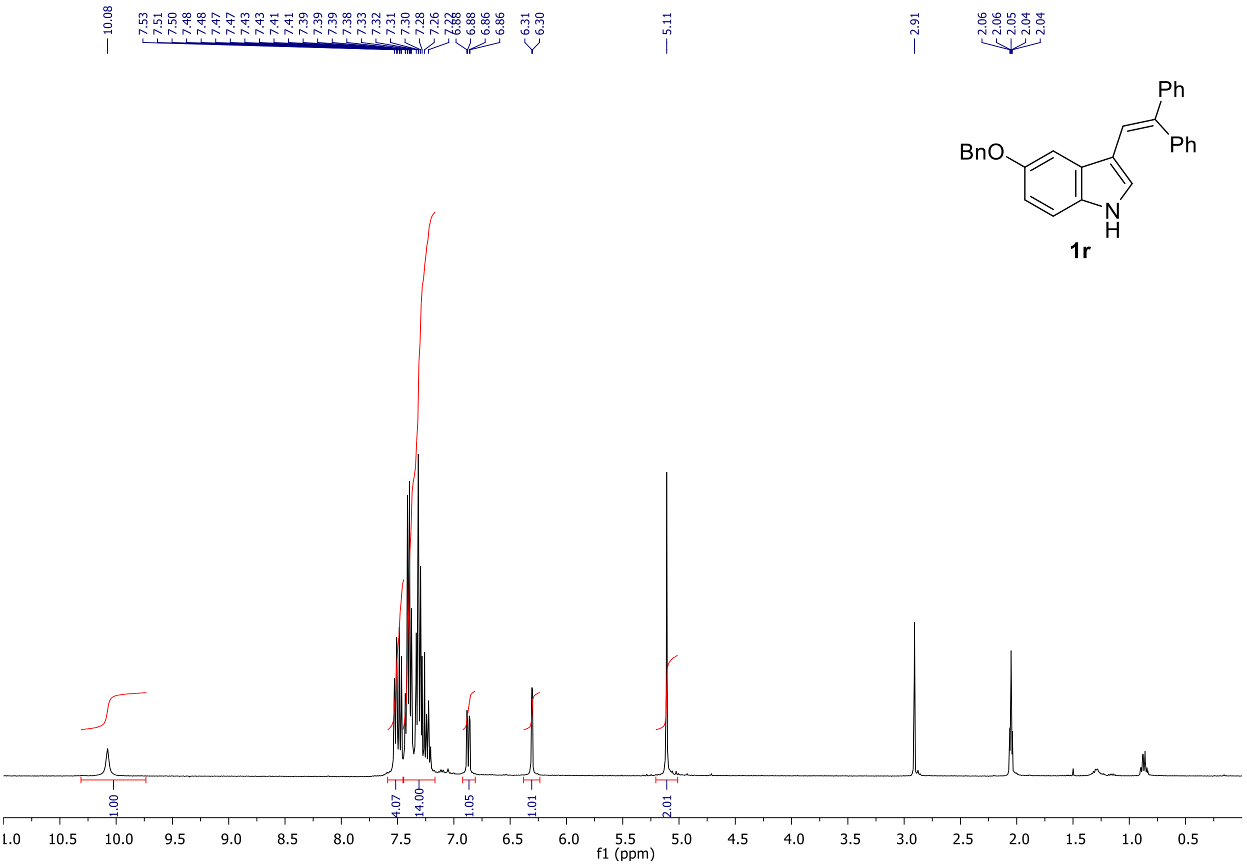

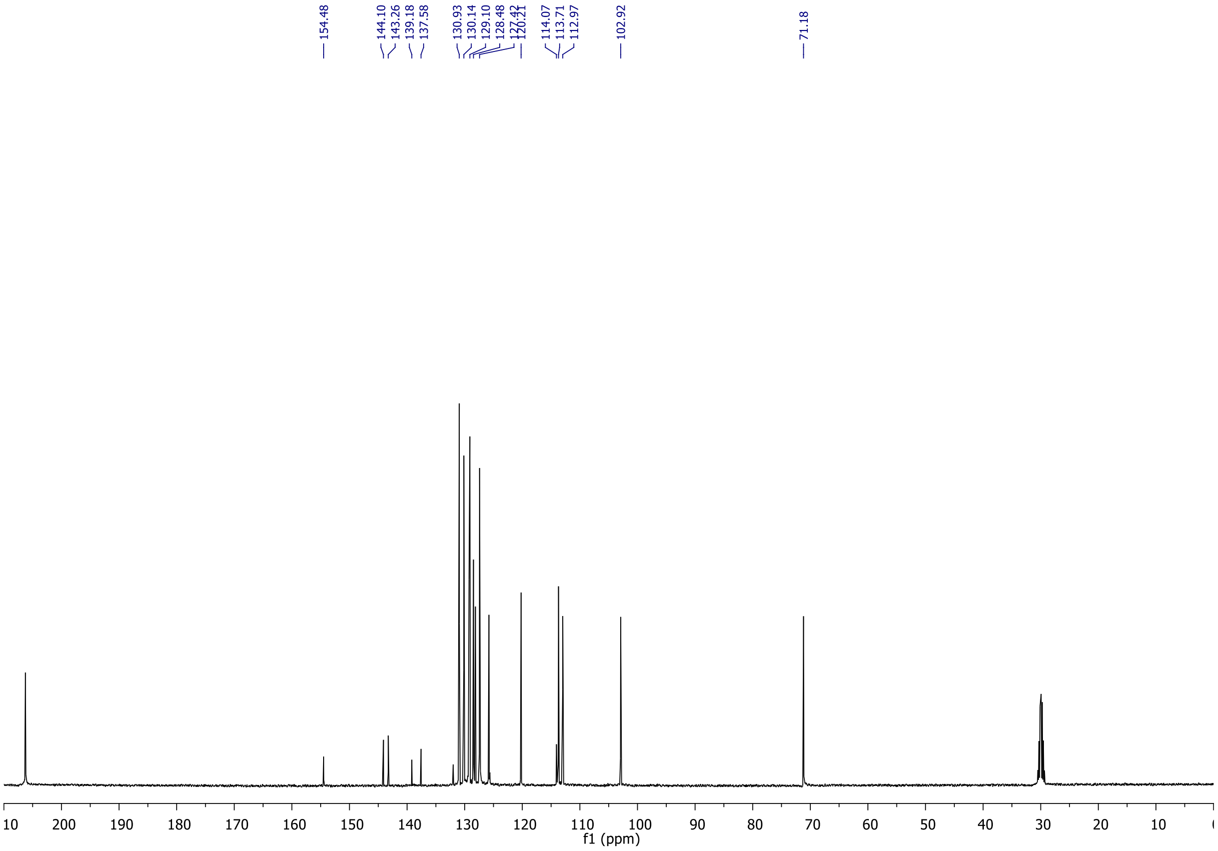

^{1}H NMR (400 MHz) and $^{13}C\{^{1}H\}$ NMR (100 MHz) spectra of **1r** (Acetone-d_6)

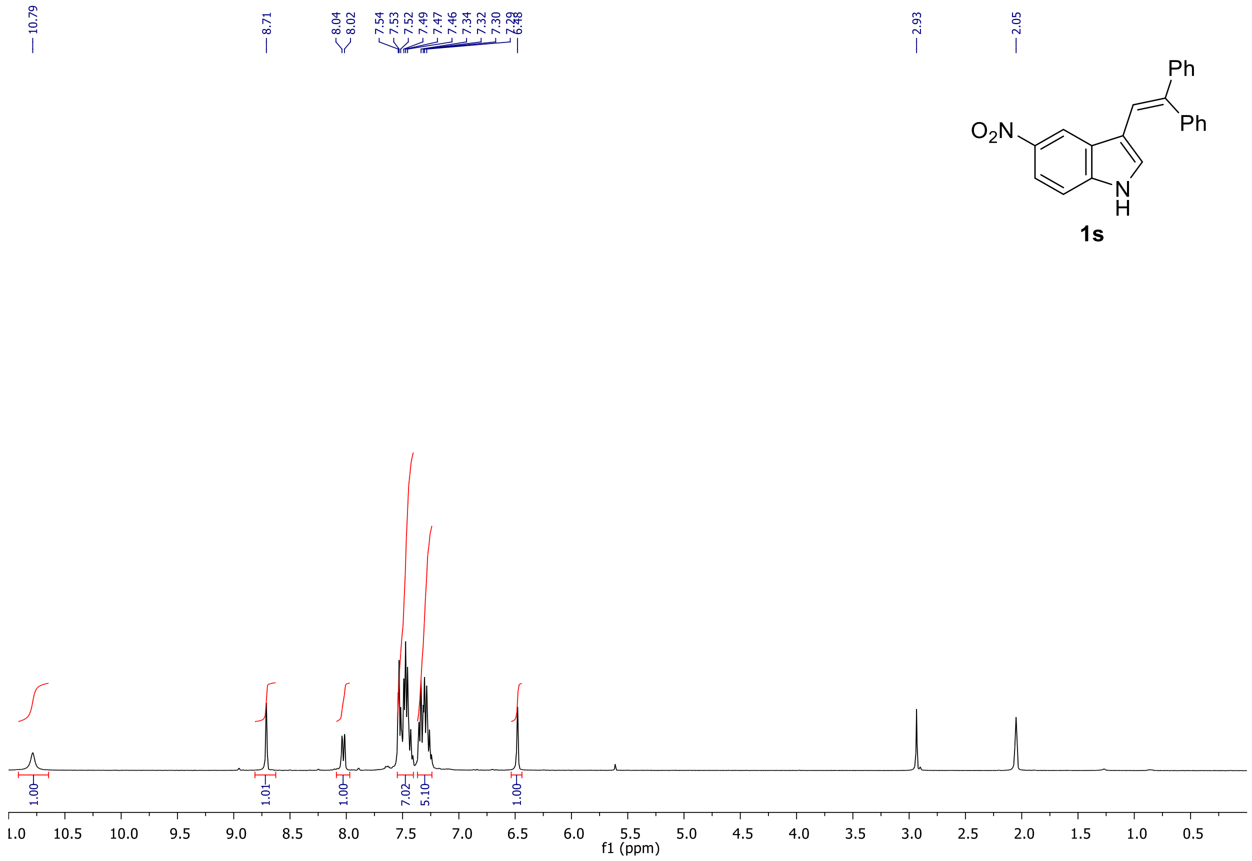

1s

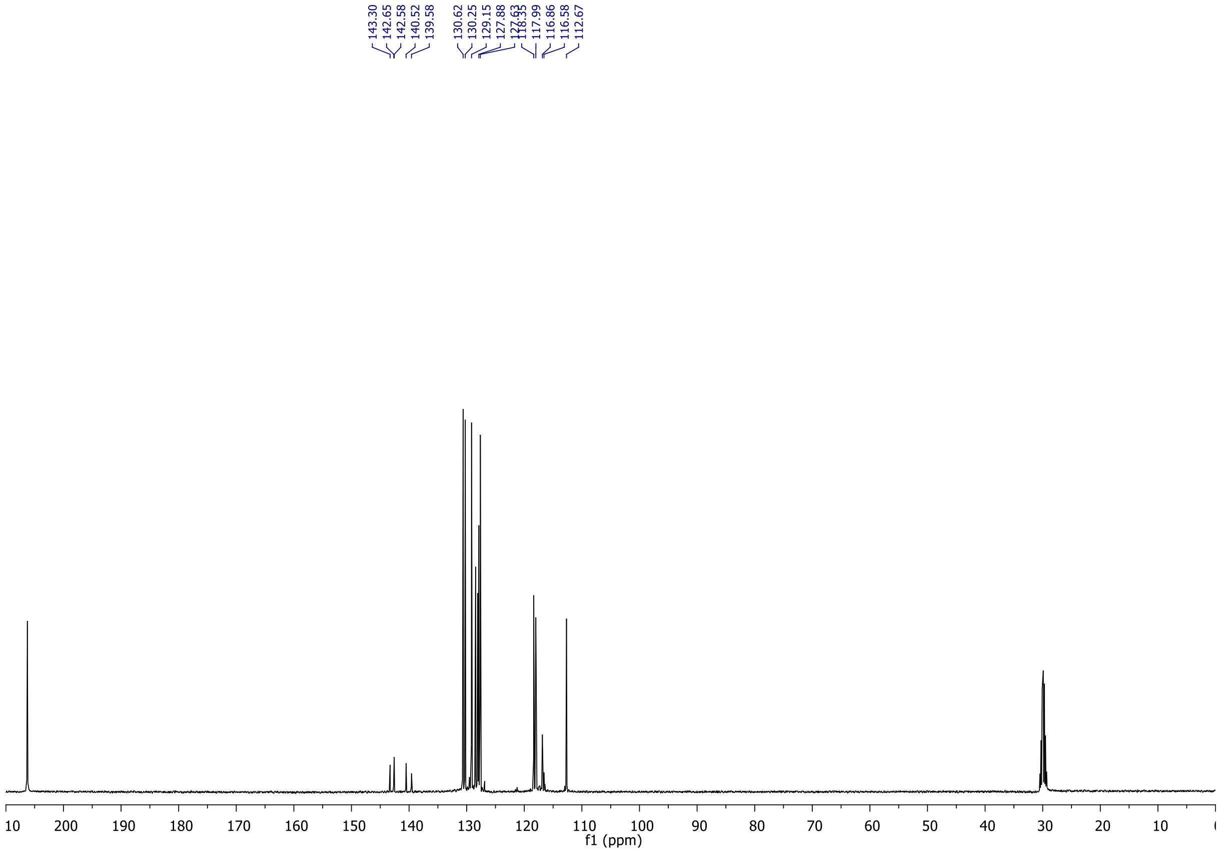

^{1}H NMR (400 MHz) and $^{13}C\{^{1}H\}$ NMR (100 MHz) spectra of **1s** (Acetone-d_6)

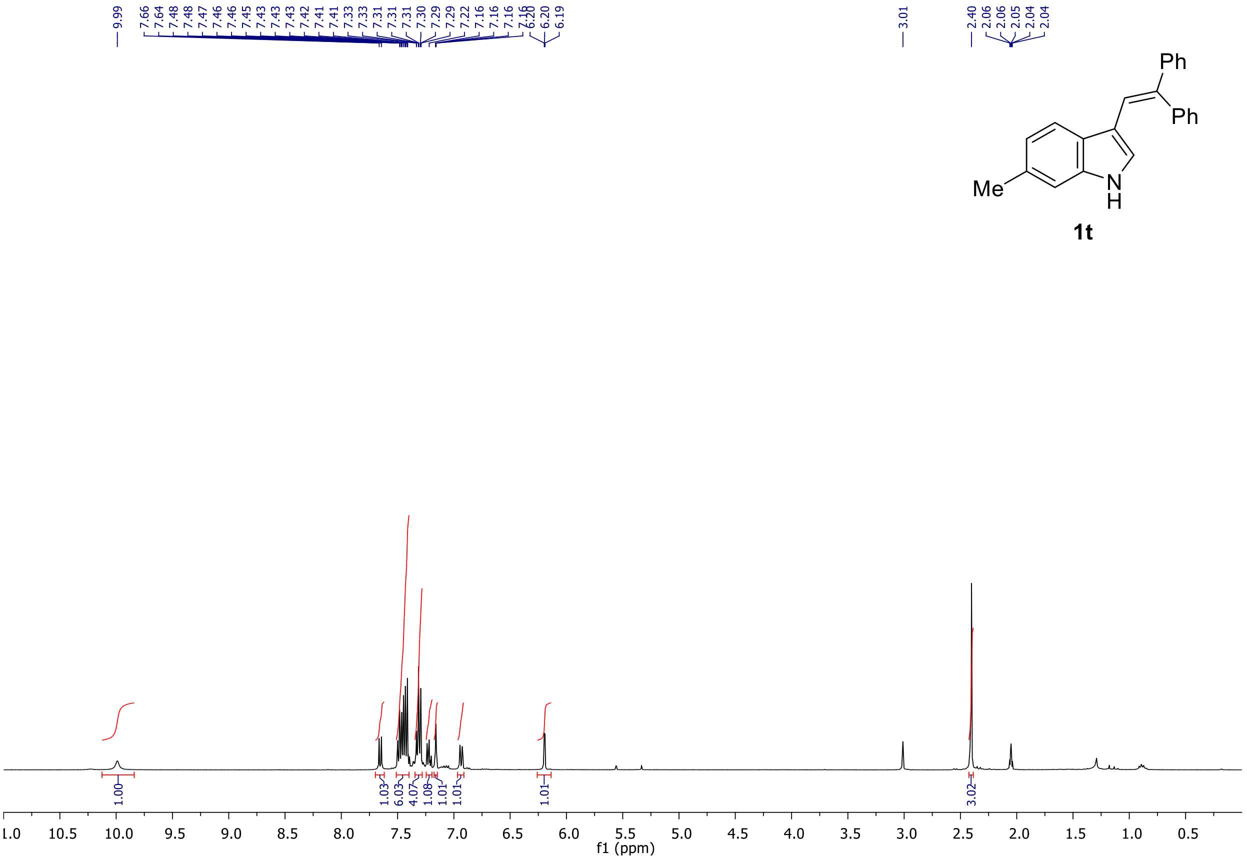

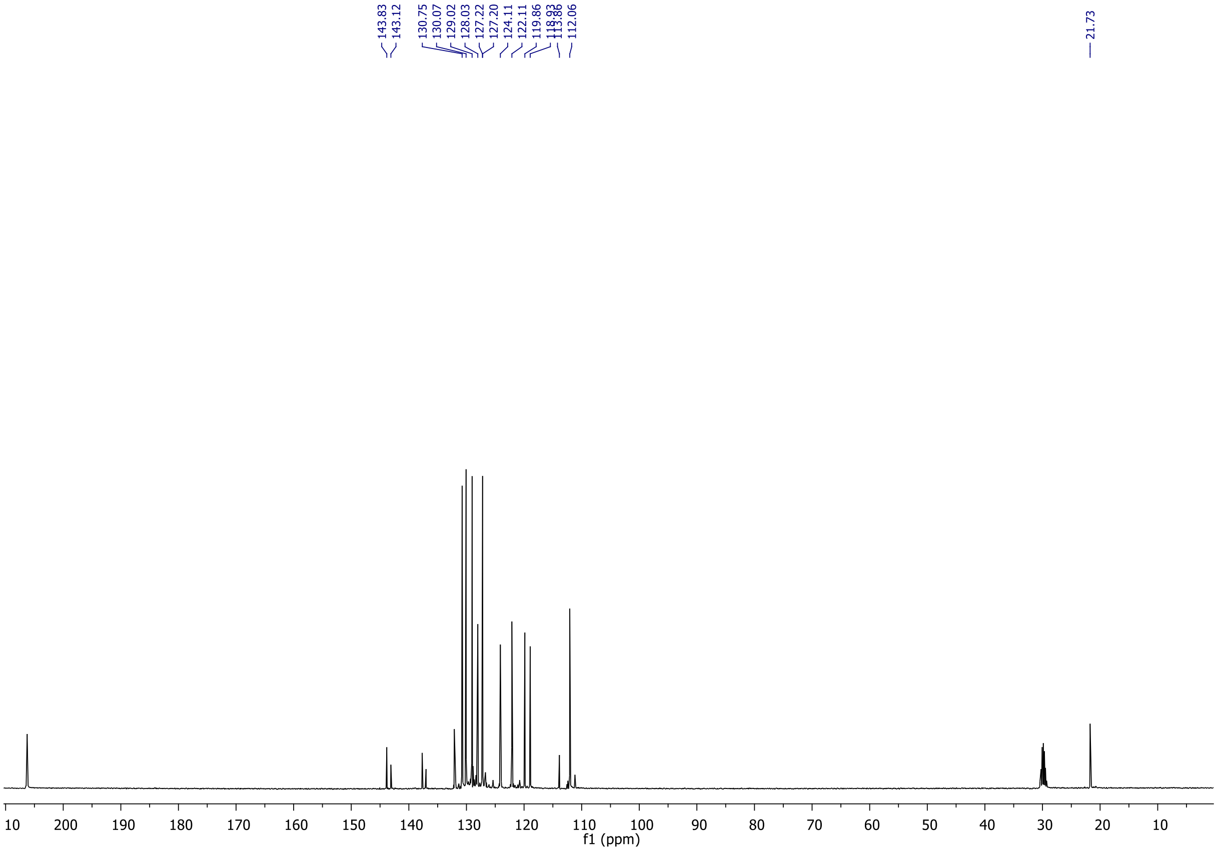

^{1}H NMR (400 MHz) and $^{13}C\{^{1}H\}$ NMR (100 MHz) spectra of **1t** (Acetone-d_6)

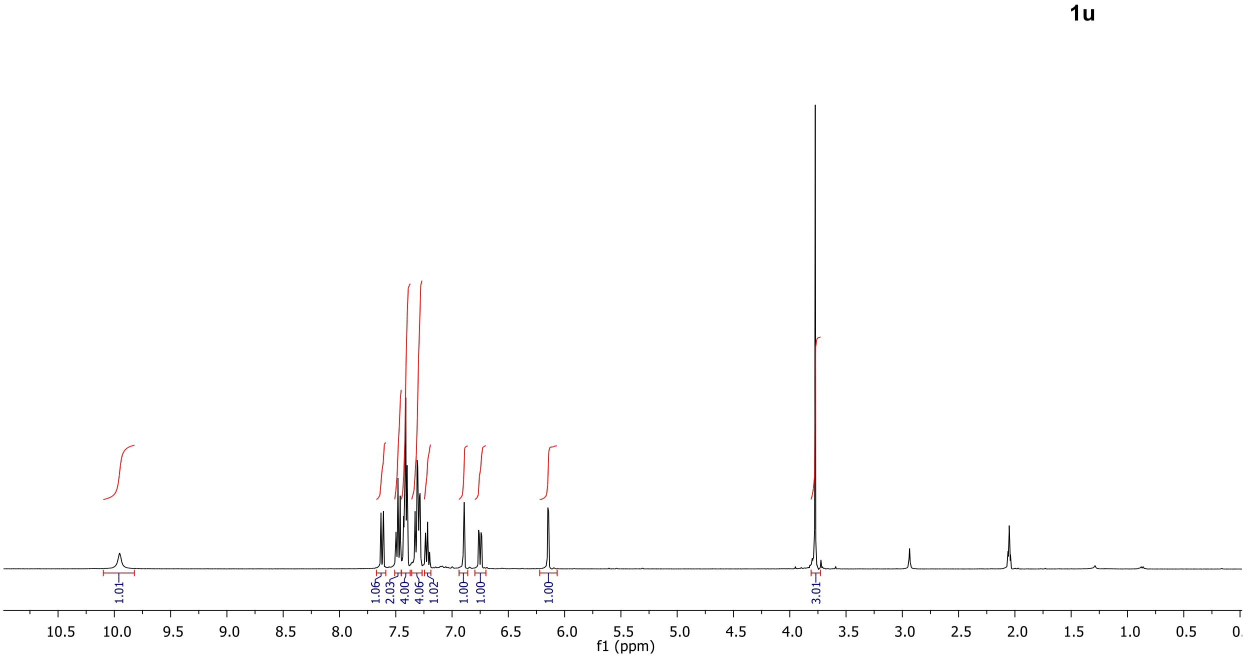

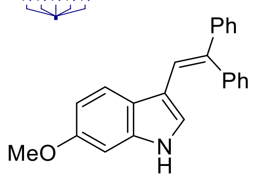

1u

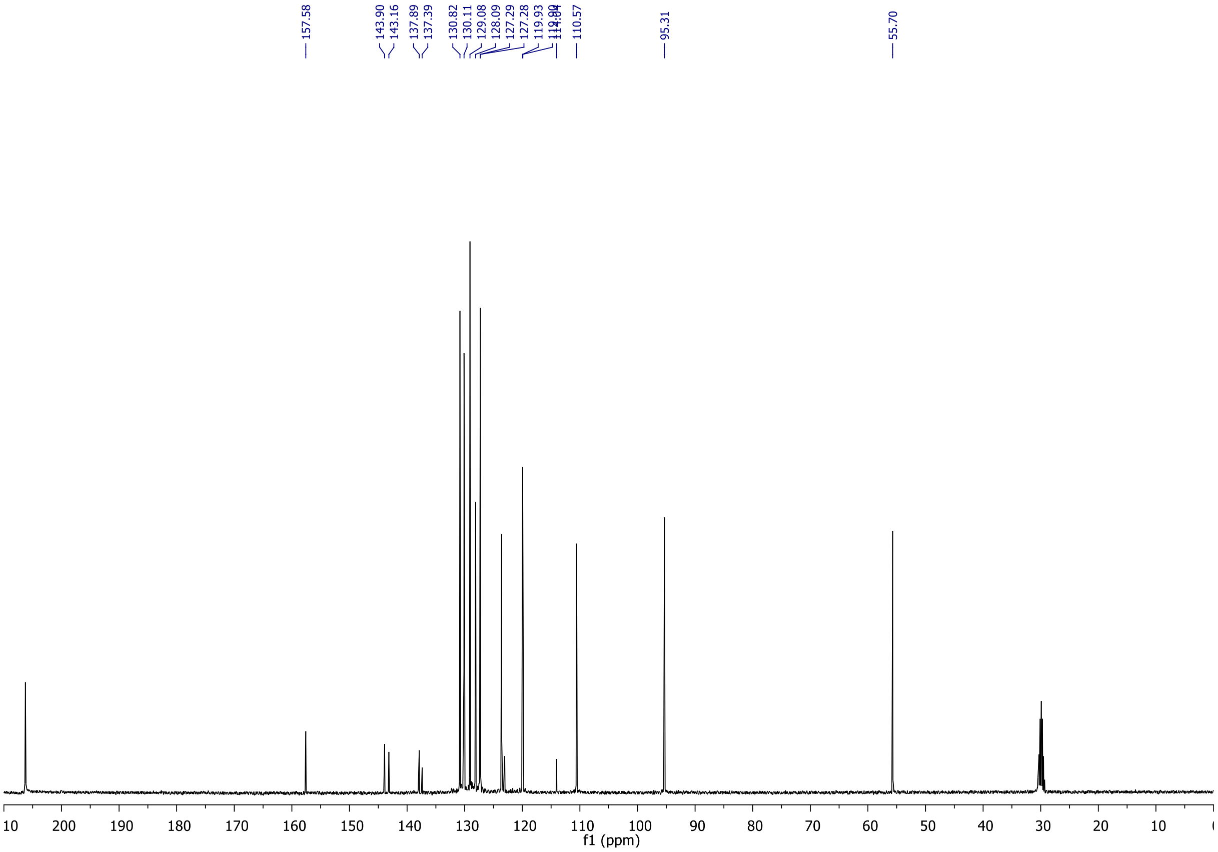

^{1}H NMR (400 MHz) and ^{13}C{^{1}H} NMR (100 MHz) spectra of **1u** (Acetone-d_6)

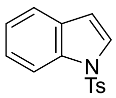

S43

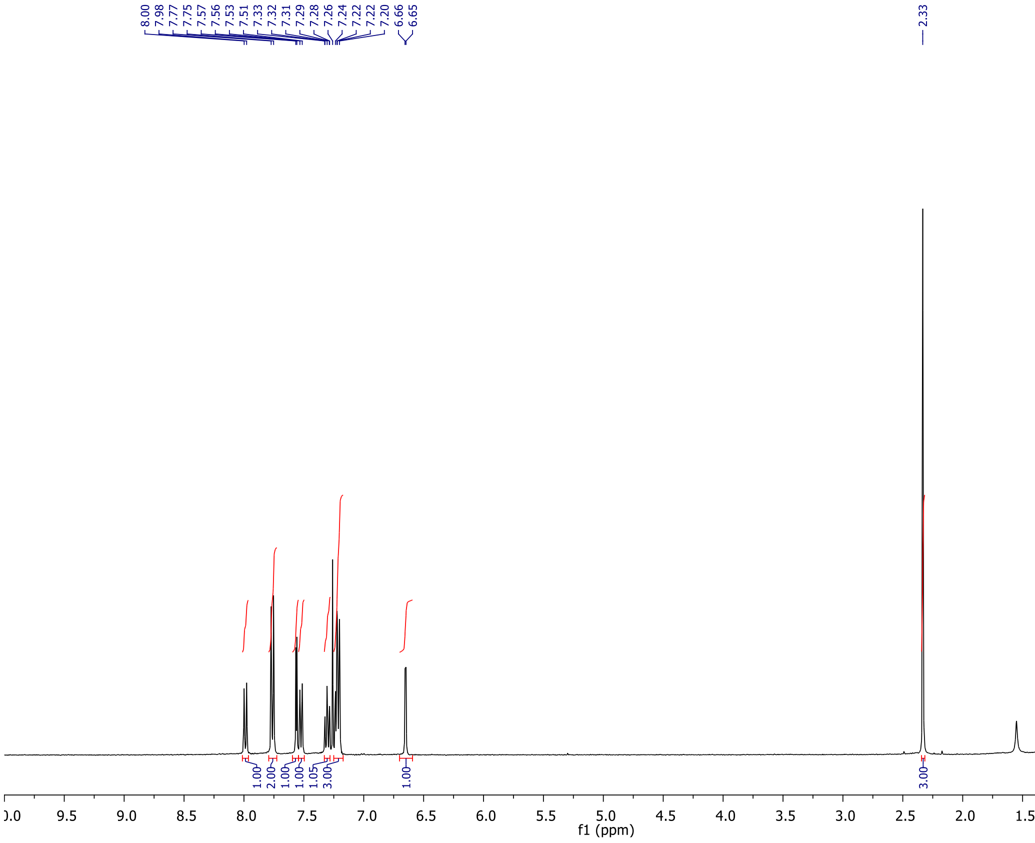

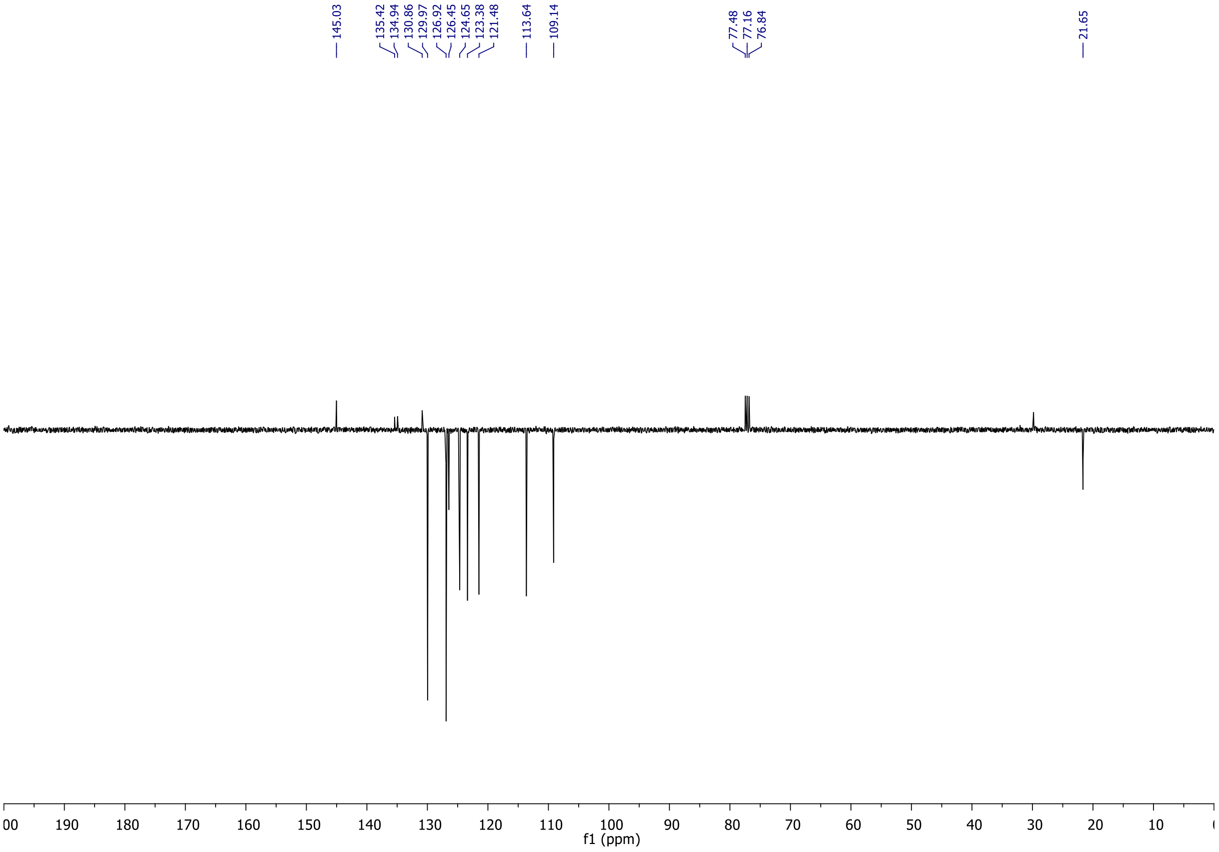

^{1}H NMR (400 MHz) and ^{13}C{^{1}H} (APT) NMR (100 MHz) spectra of **S43** ($CDCl_3$)

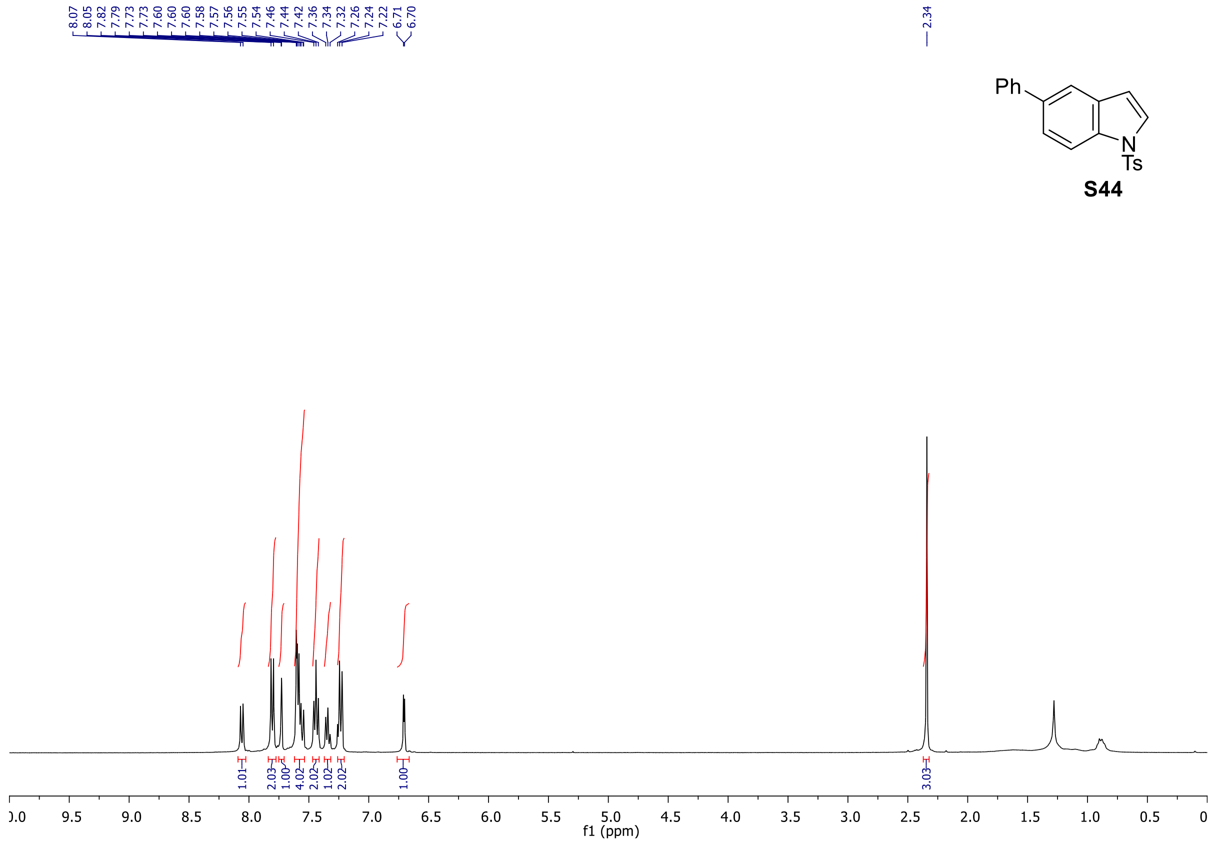

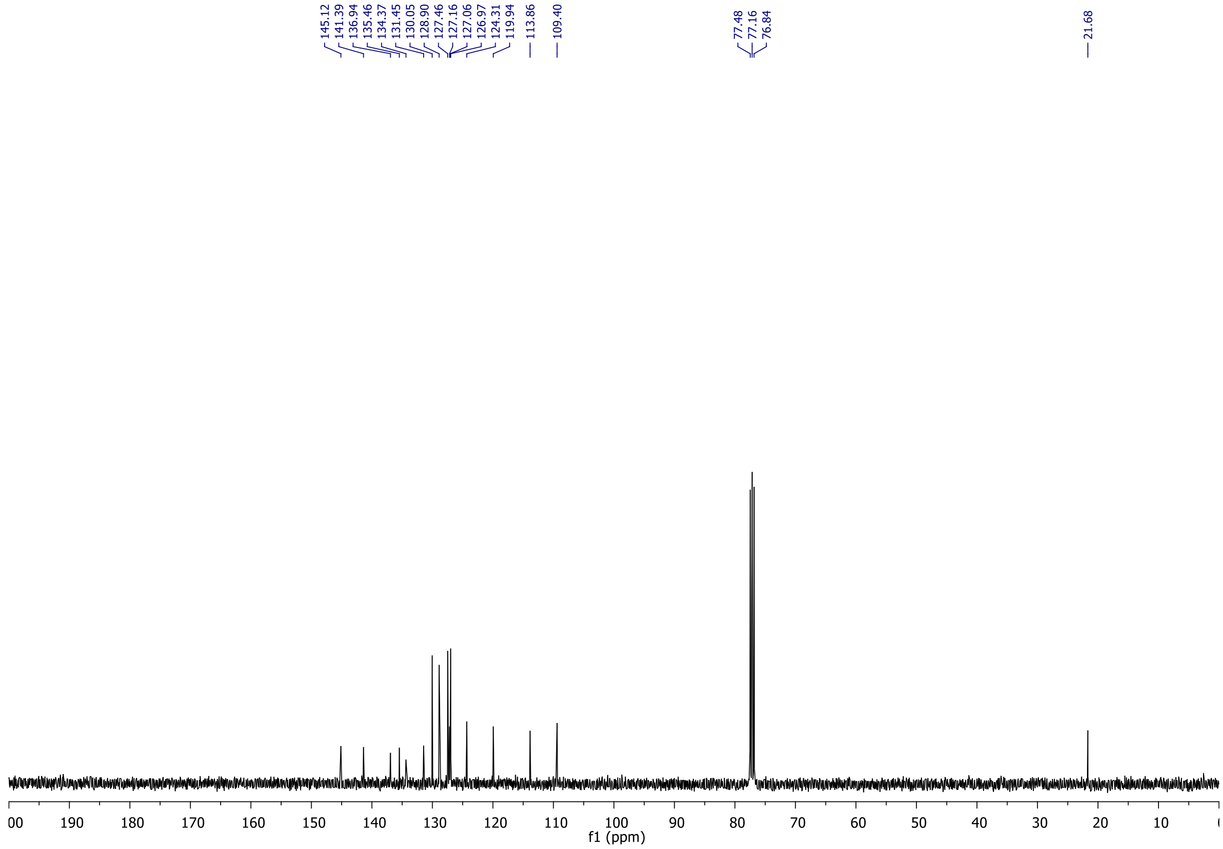

^{1}H NMR (400 MHz) and ^{13}C{^{1}H} NMR (100 MHz) spectra of **S44** ($CDCl_3$)

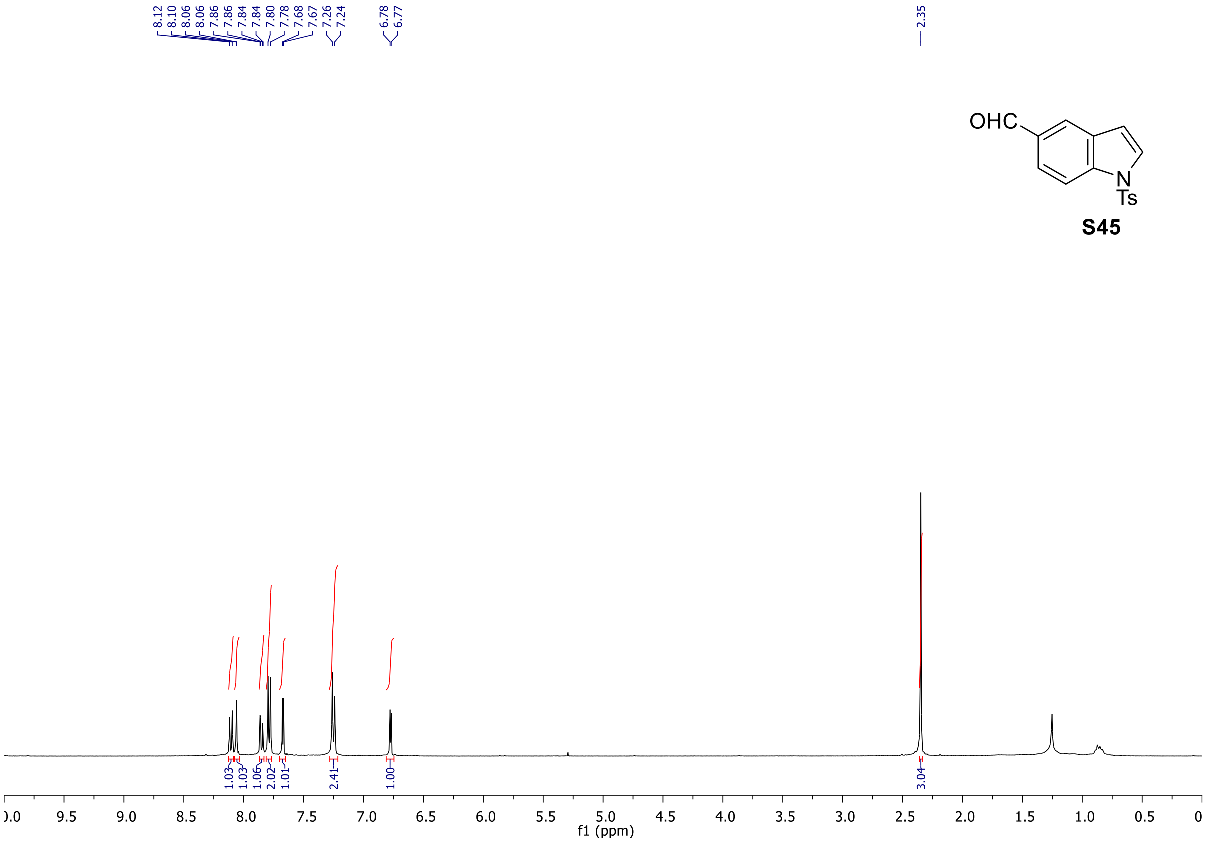

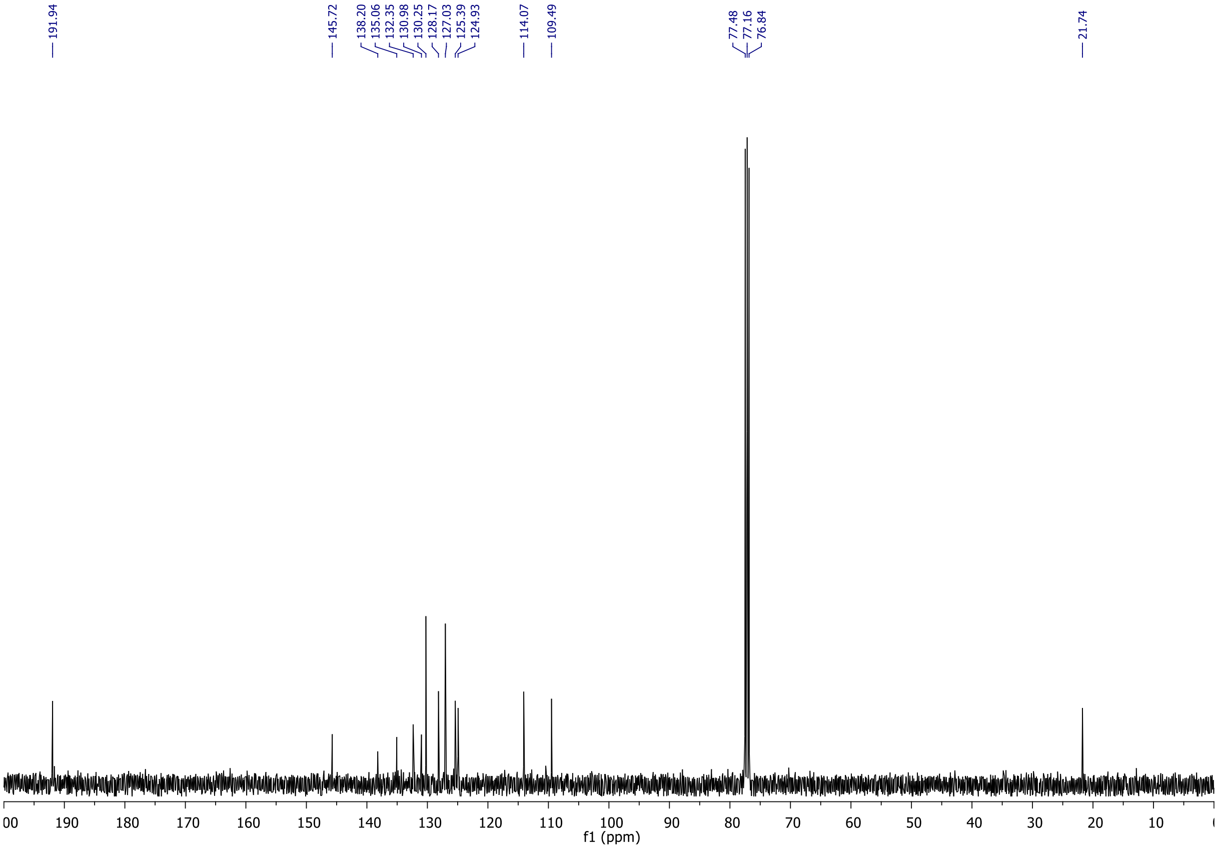

^{1}H NMR (400 MHz) and ^{13}C{^{1}H} NMR (100 MHz) spectra of **S45** ($CDCl_3$)

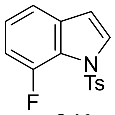

S46

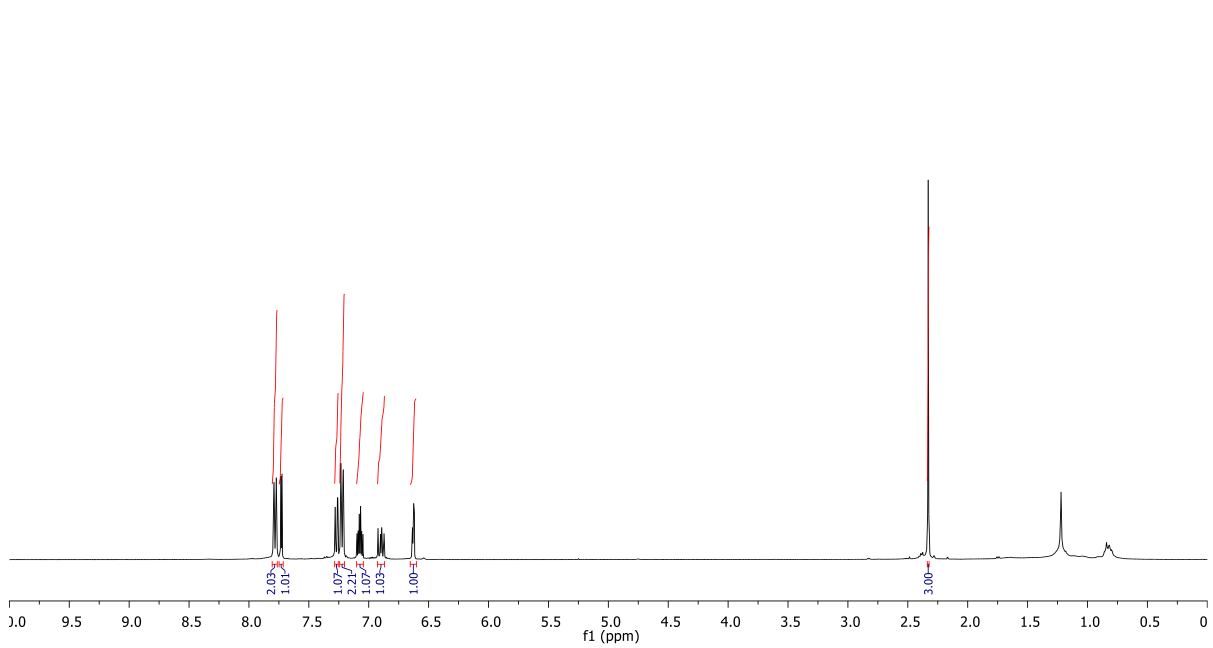

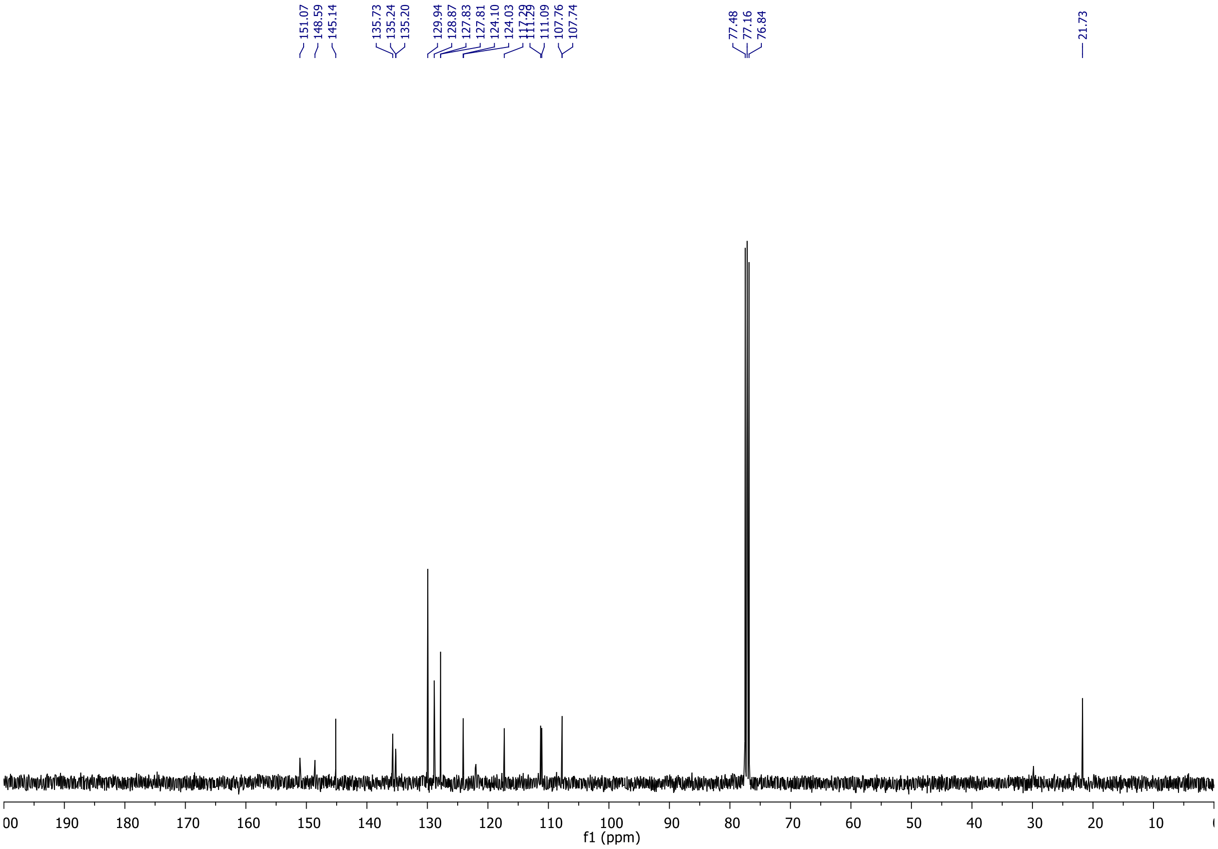

^{1}H NMR (400 MHz) and $^{13}C\{^{1}H\}$ NMR (100 MHz) spectra of **S46** ($CDCl_3$)

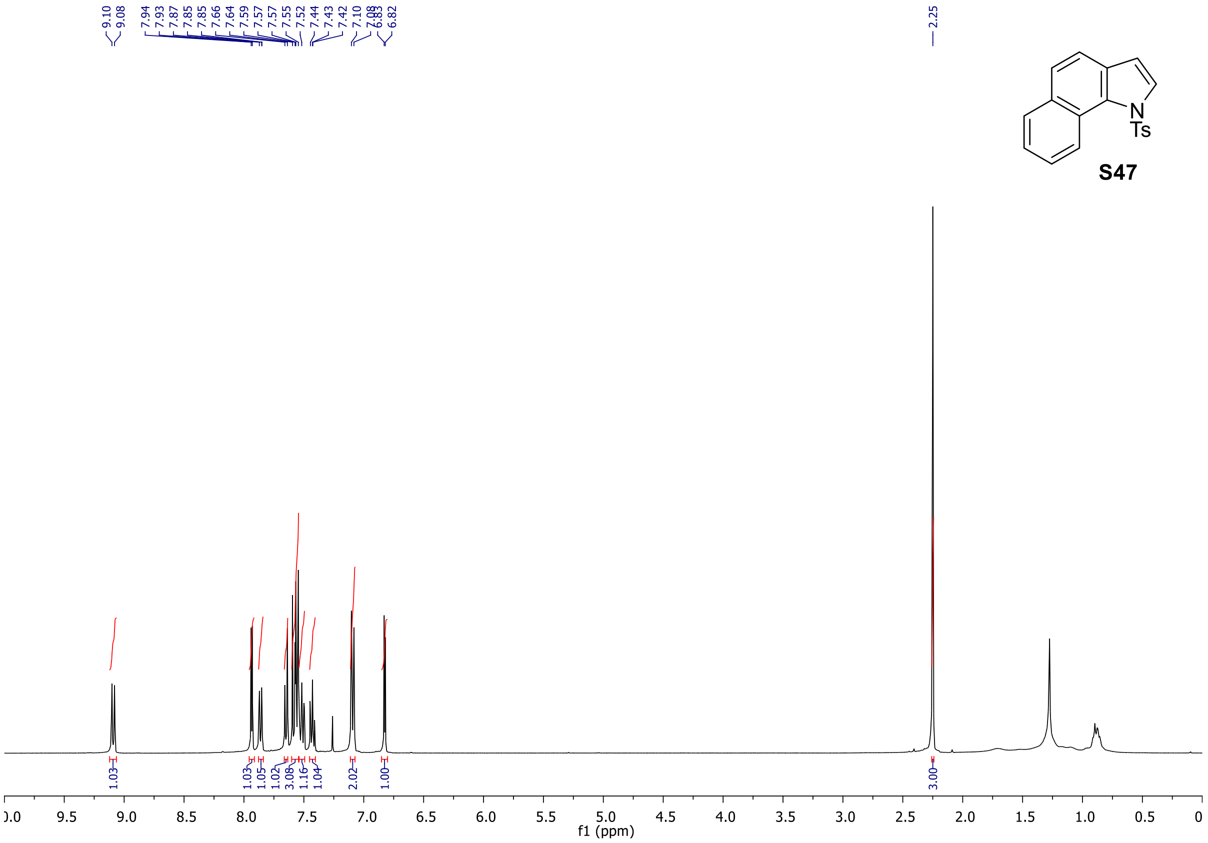

S47

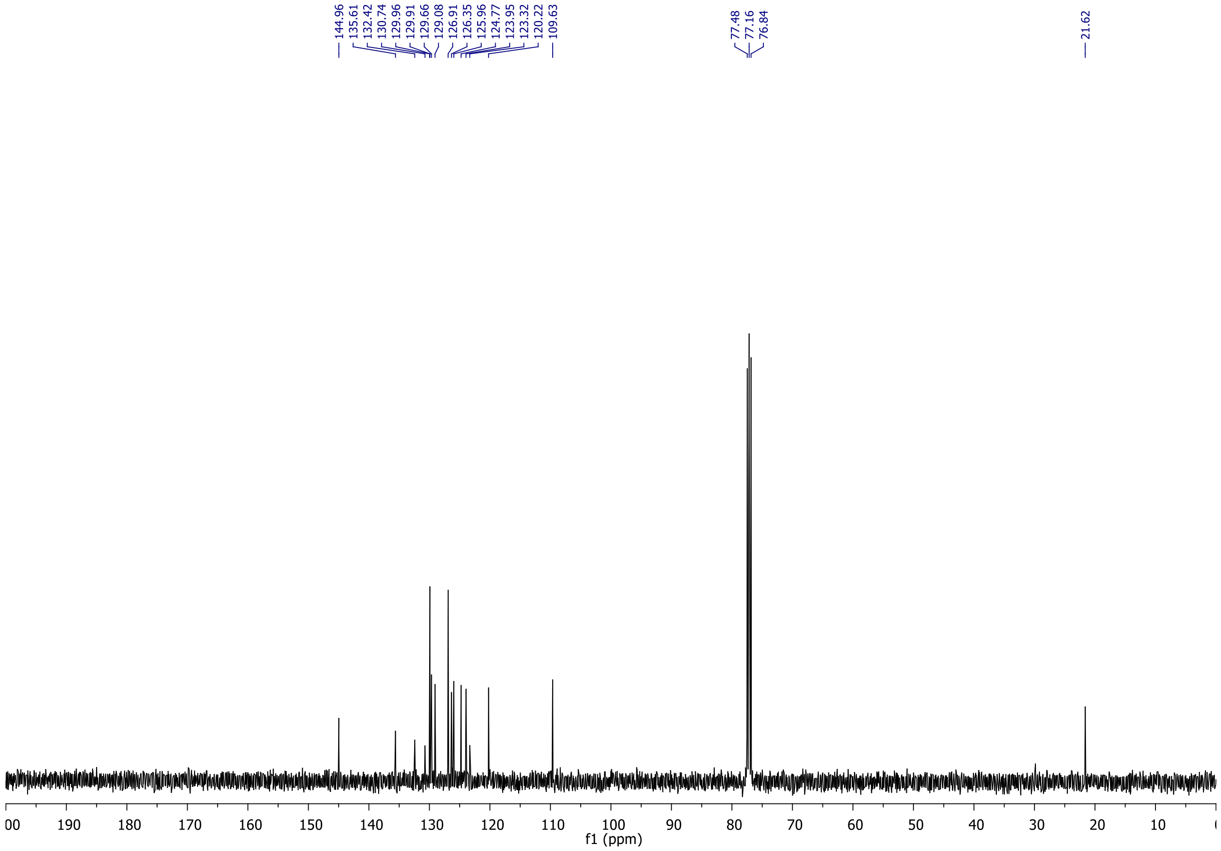

^{1}H NMR (400 MHz) and ^{13}C{^{1}H} NMR (100 MHz) spectra of **S47** ($CDCl_3$)

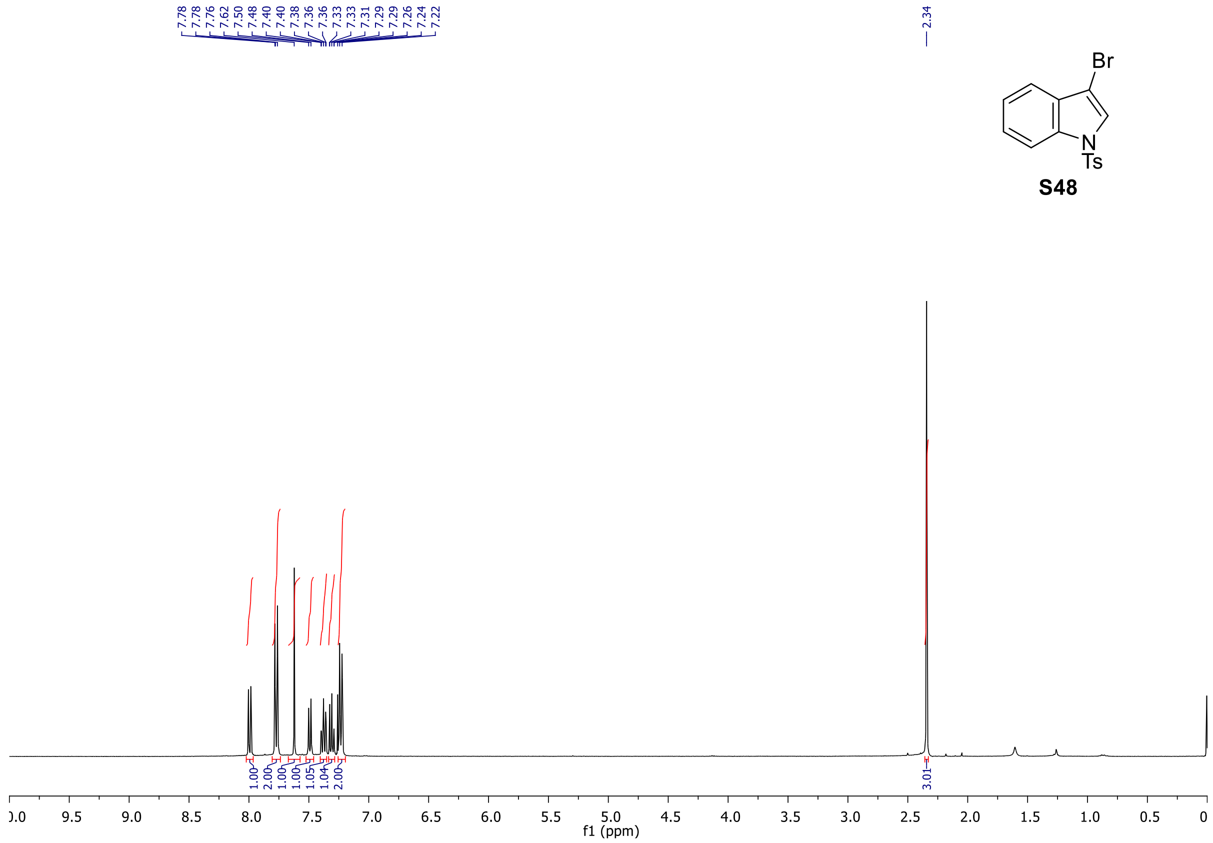

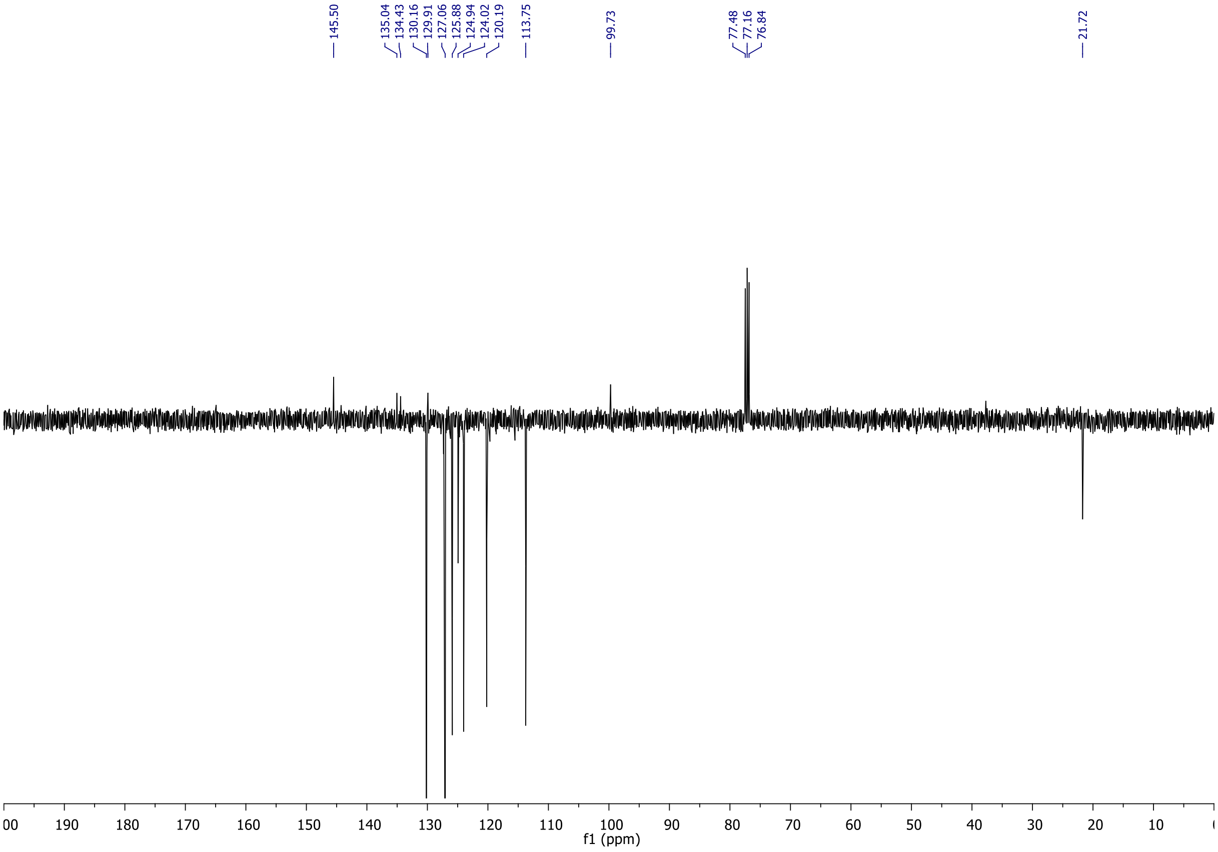

^{1}H NMR (400 MHz) and ^{13}C{^{1}H} (APT) NMR (100 MHz) spectra of **S48** ($CDCl_3$)

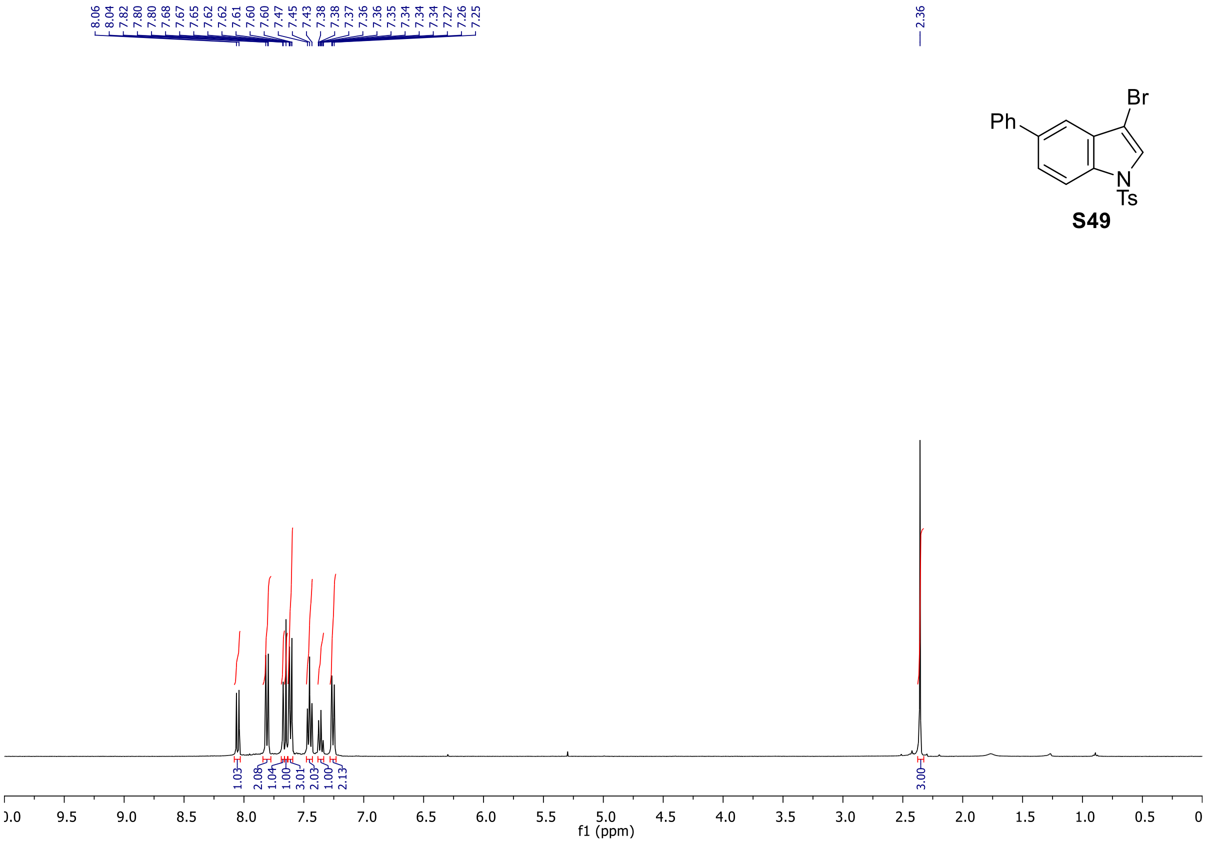

S49

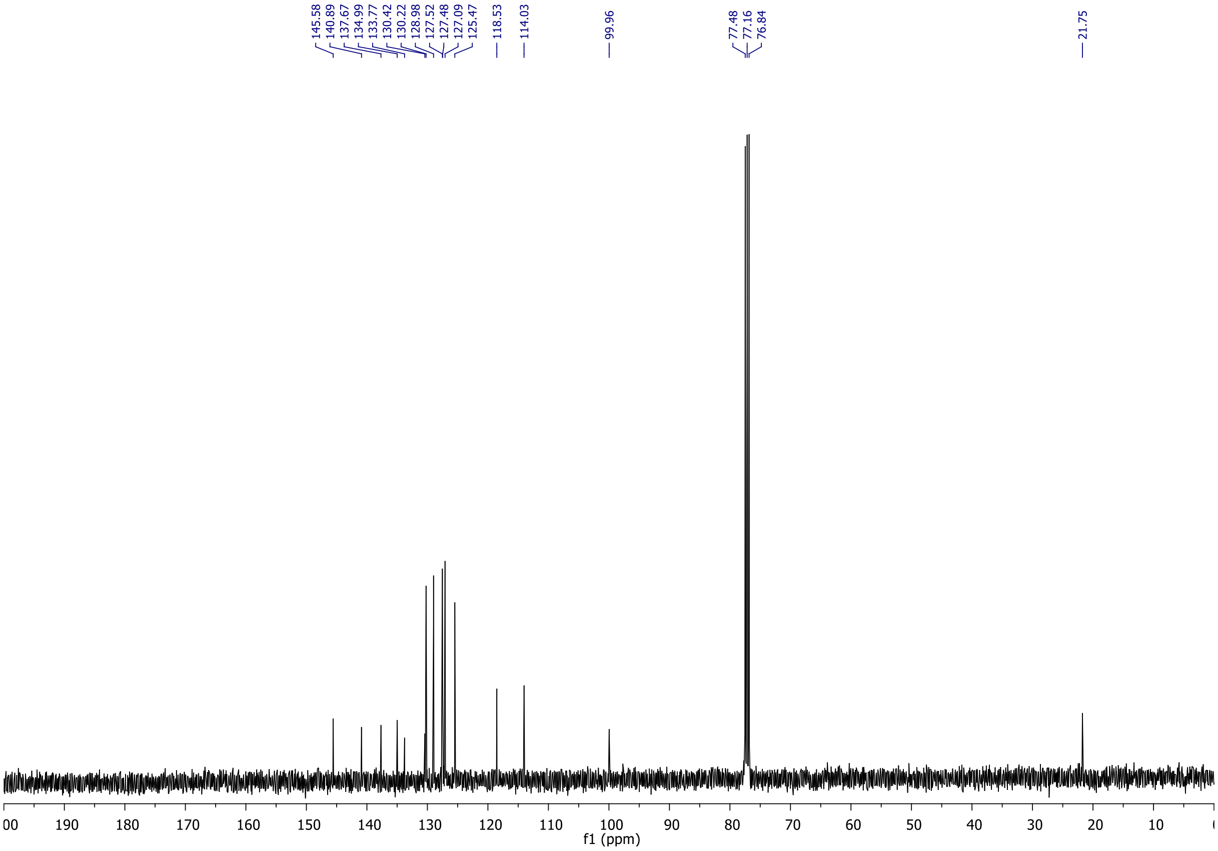

^{1}H NMR (400 MHz) and ^{13}C{^{1}H} NMR (100 MHz) spectra of **S49** ($CDCl_3$)

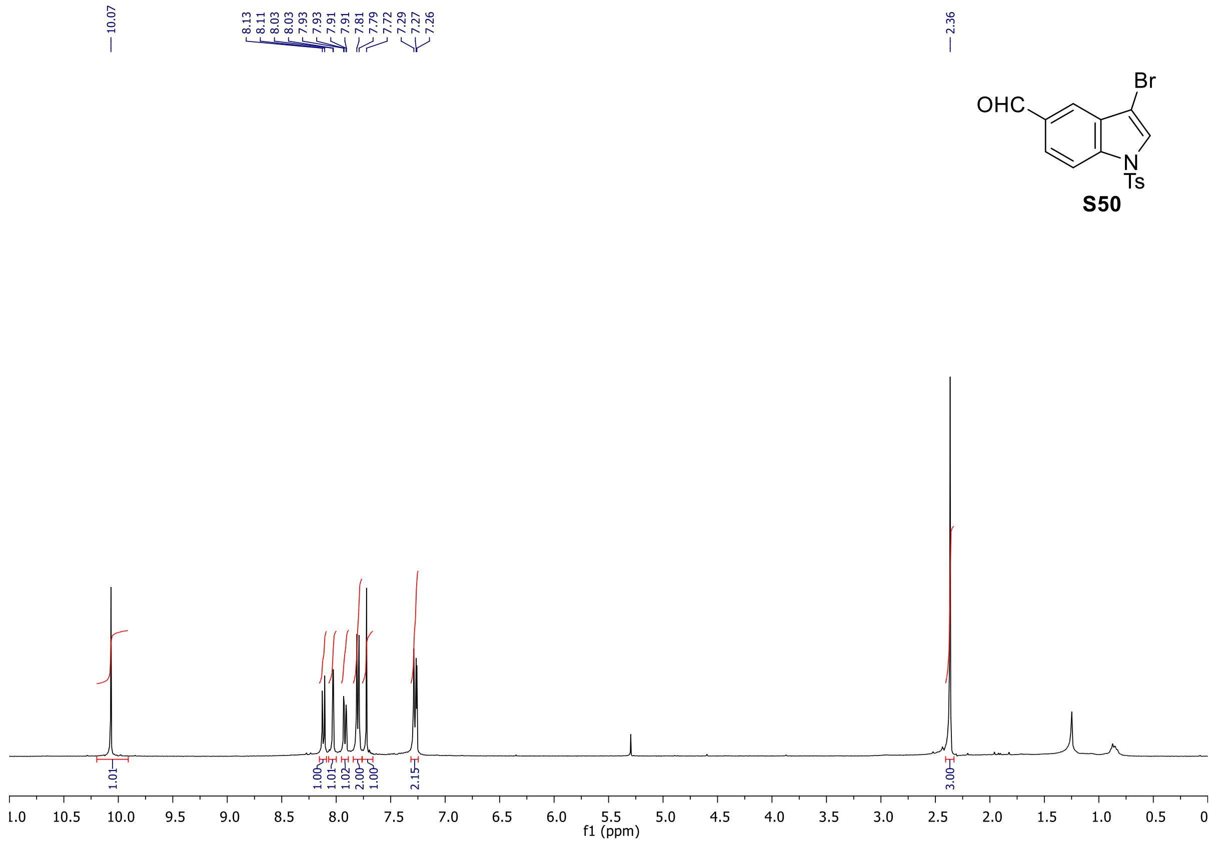

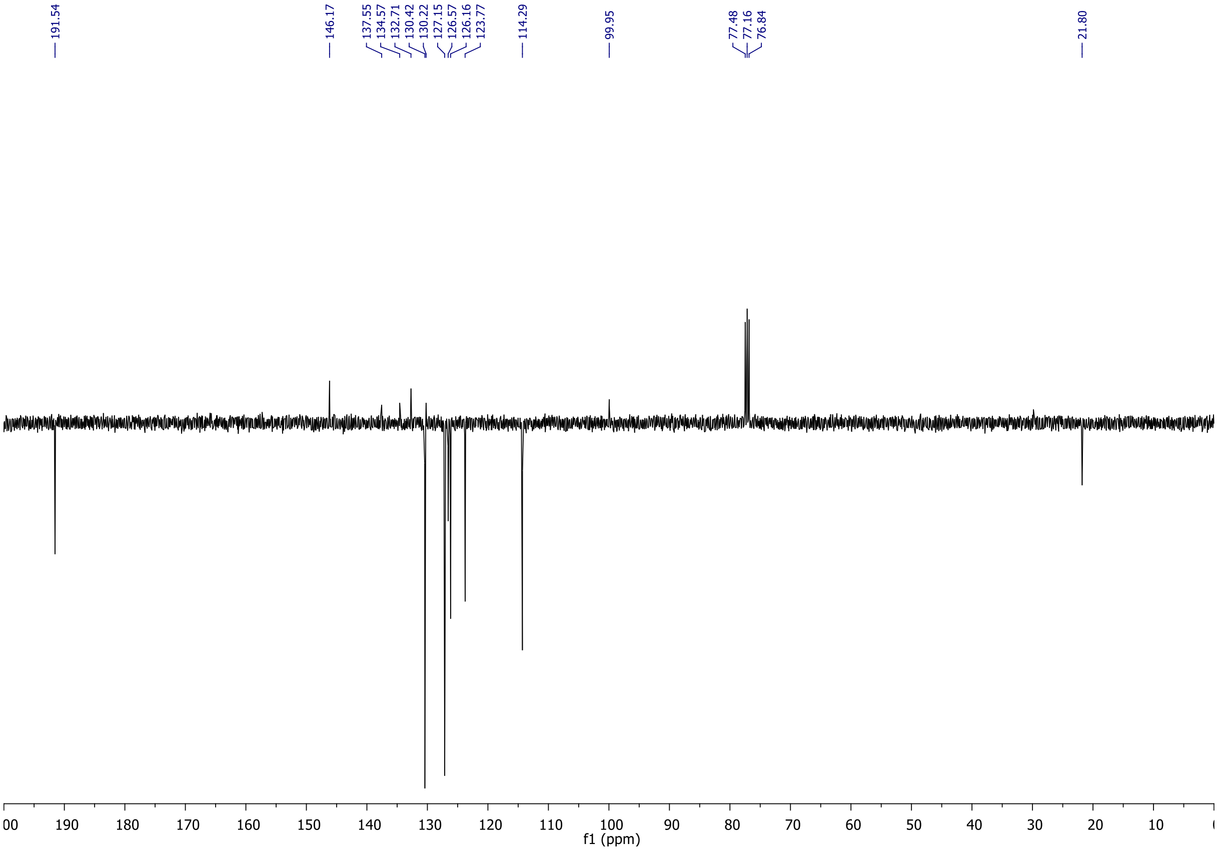

^{1}H NMR (400 MHz) and ^{13}C{^{1}H} (APT) NMR (100 MHz) spectra of **S50** ($CDCl_3$)

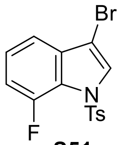

S51

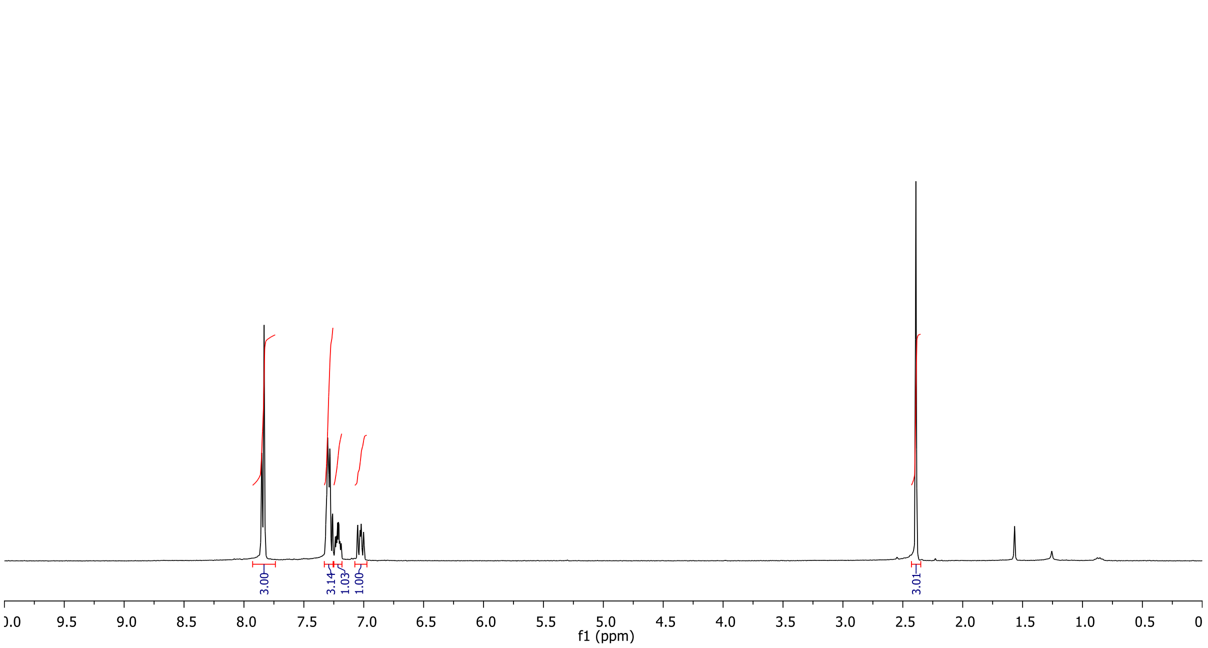

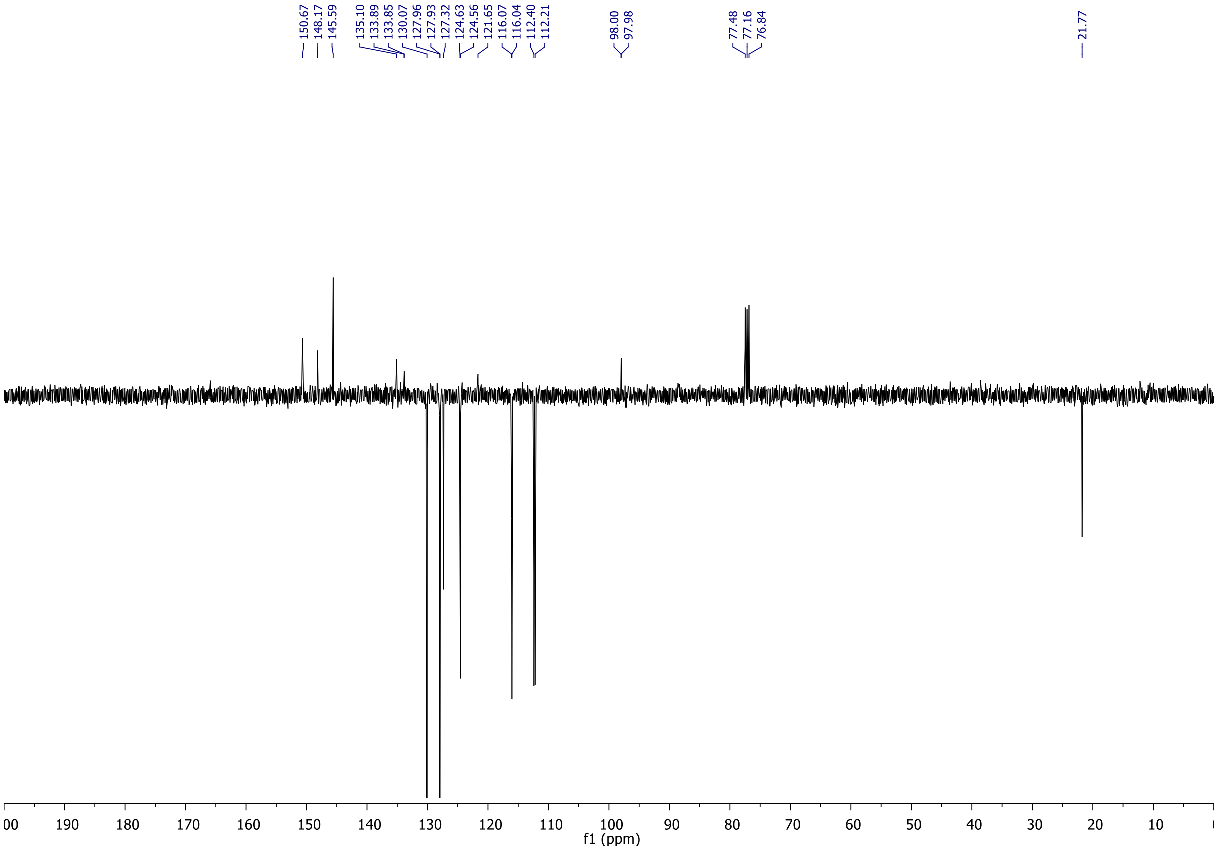

^{1}H NMR (400 MHz) and ^{13}C{^{1}H} (APT) NMR (100 MHz) spectra of **S51** ($CDCl_3$)

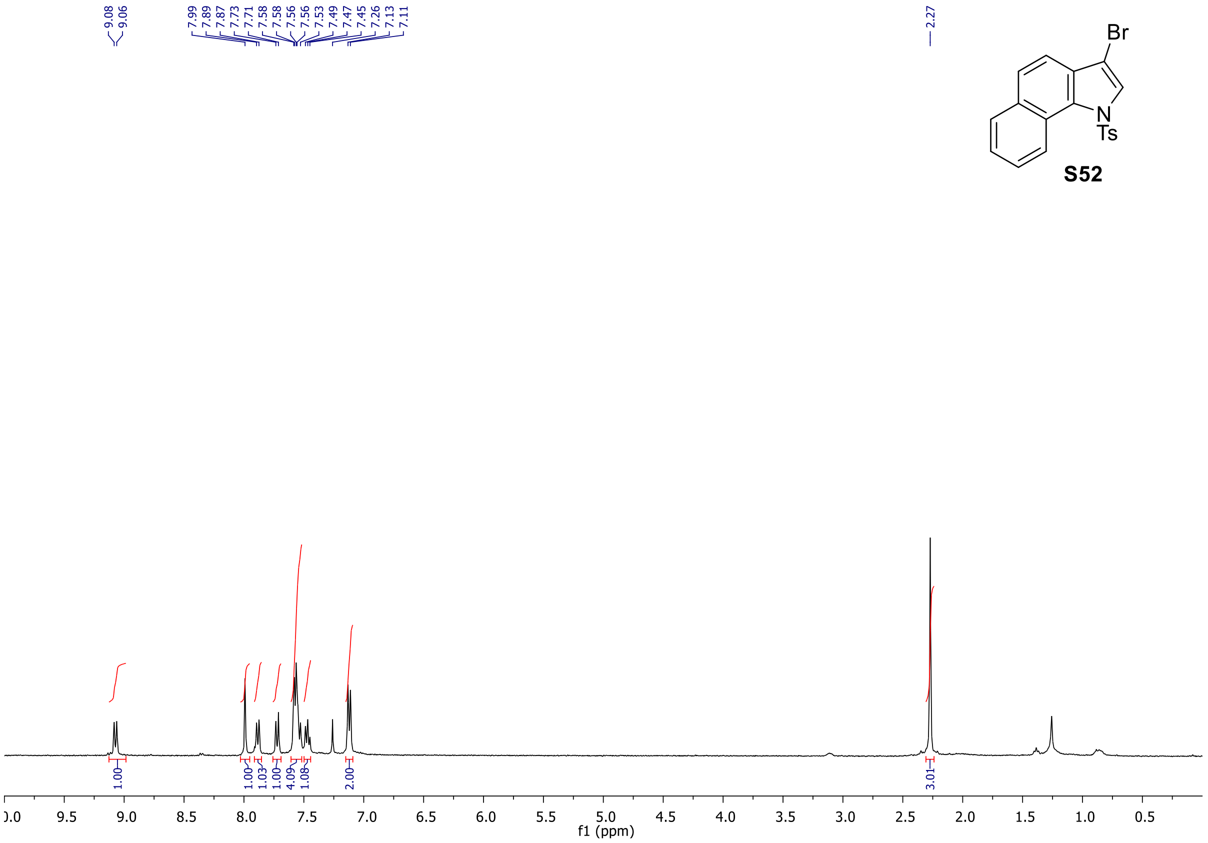

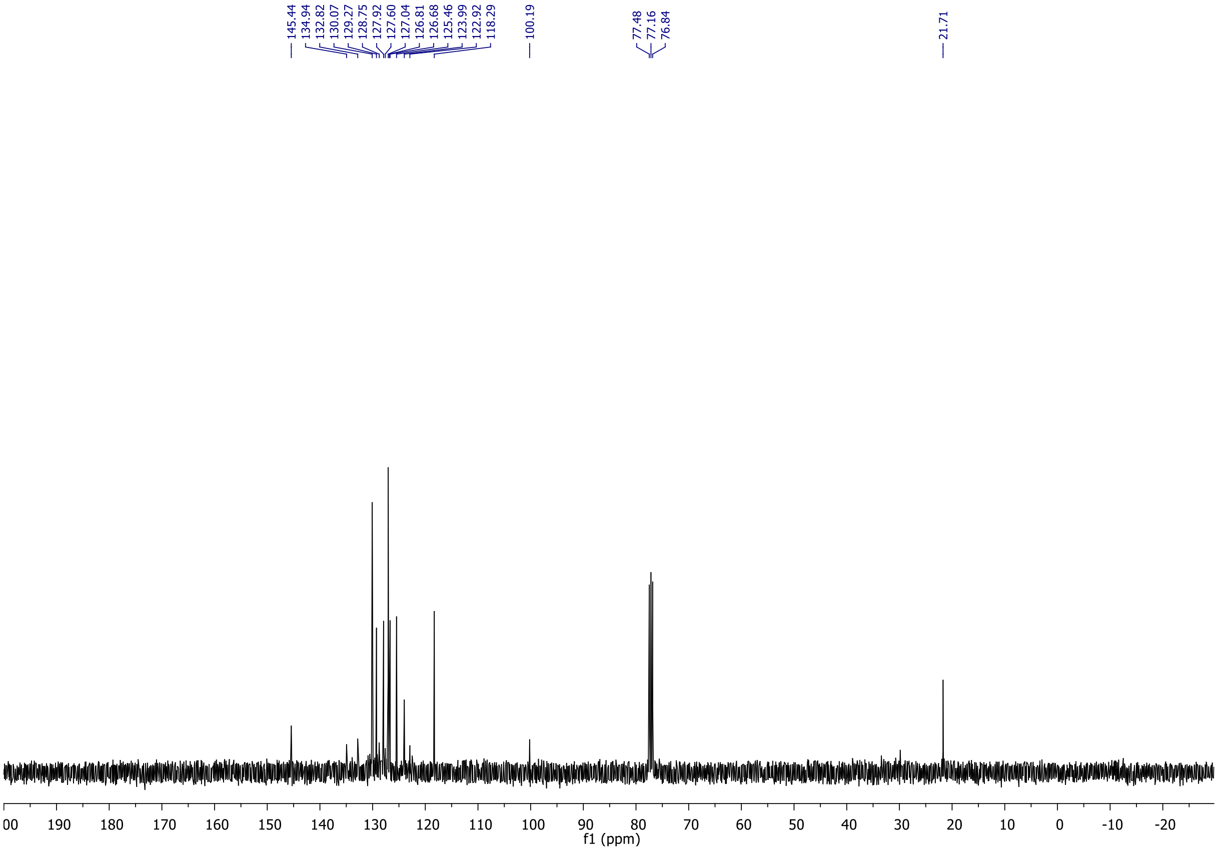

^{1}H NMR (400 MHz) and $^{13}C\{^{1}H\}$ NMR (100 MHz) spectra of **S52** ($CDCl_3$)

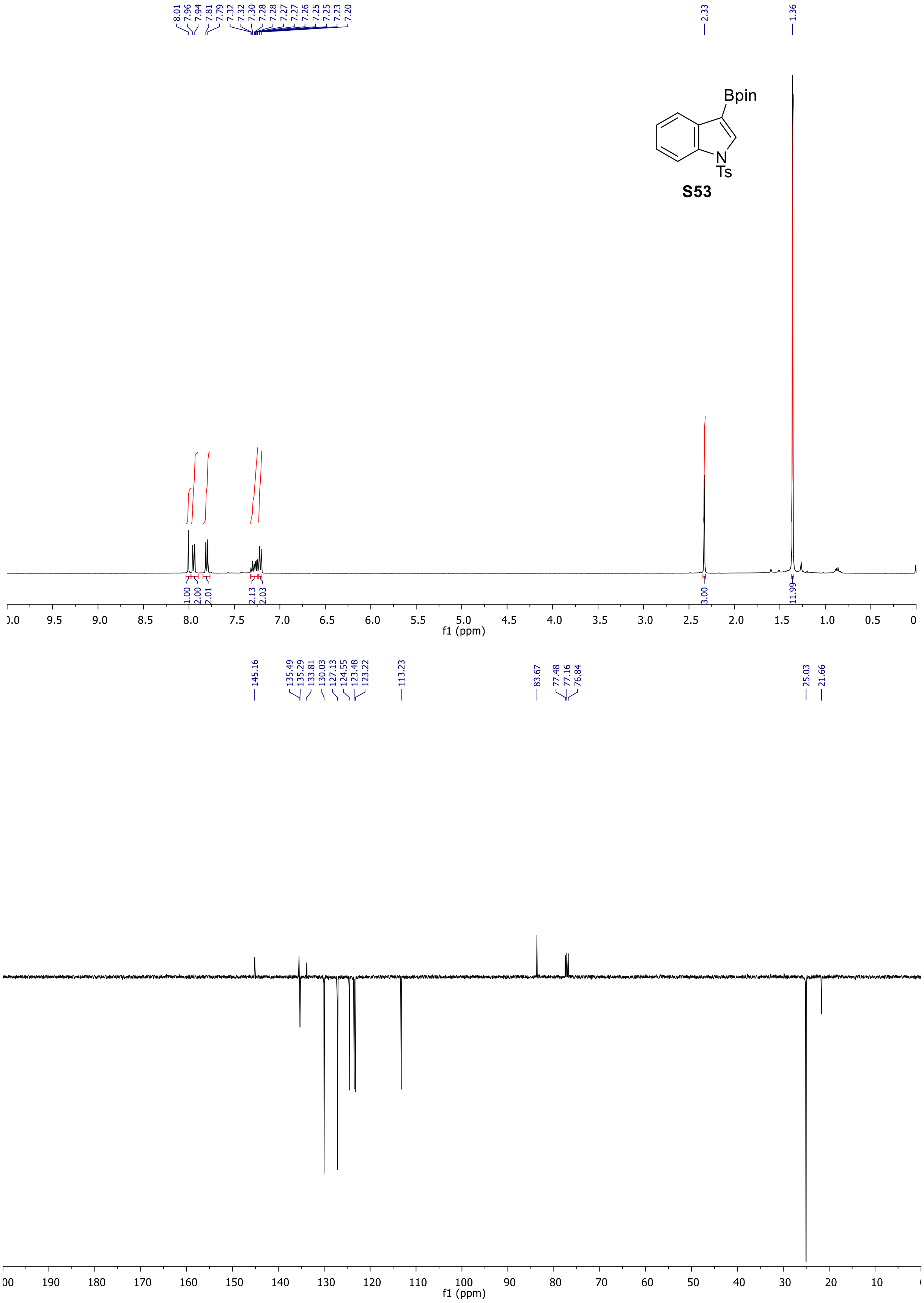

^{1}H NMR (400 MHz) and $^{13}C\{^{1}H\}$ (APT) NMR (100 MHz) spectra of **S53** ($CDCl_3$)

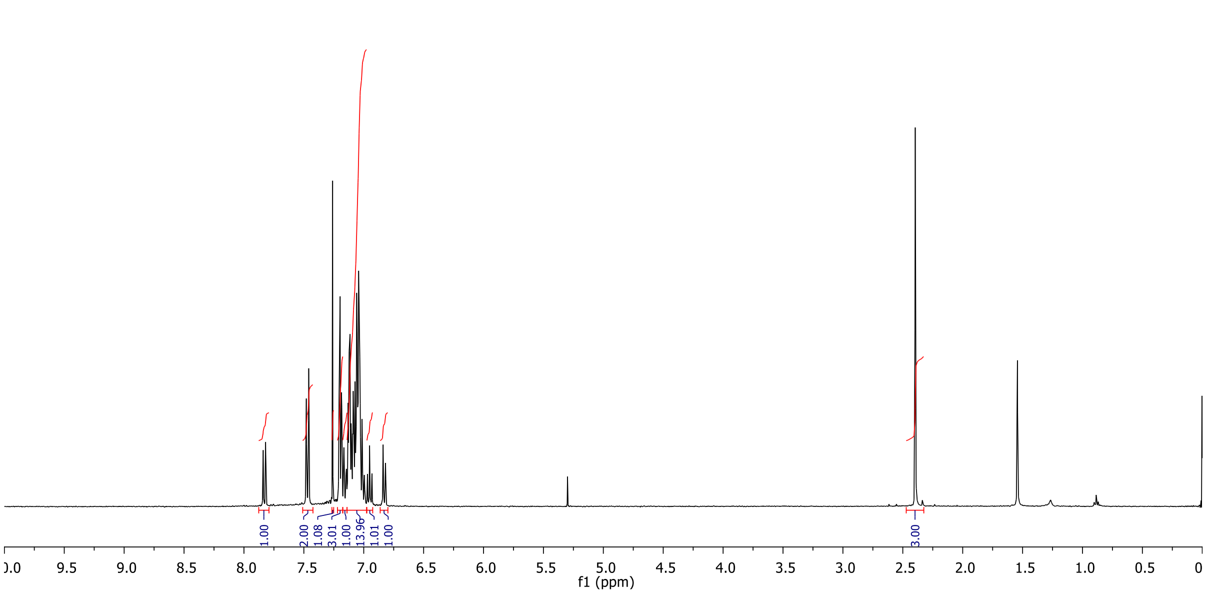

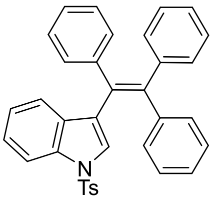

S57

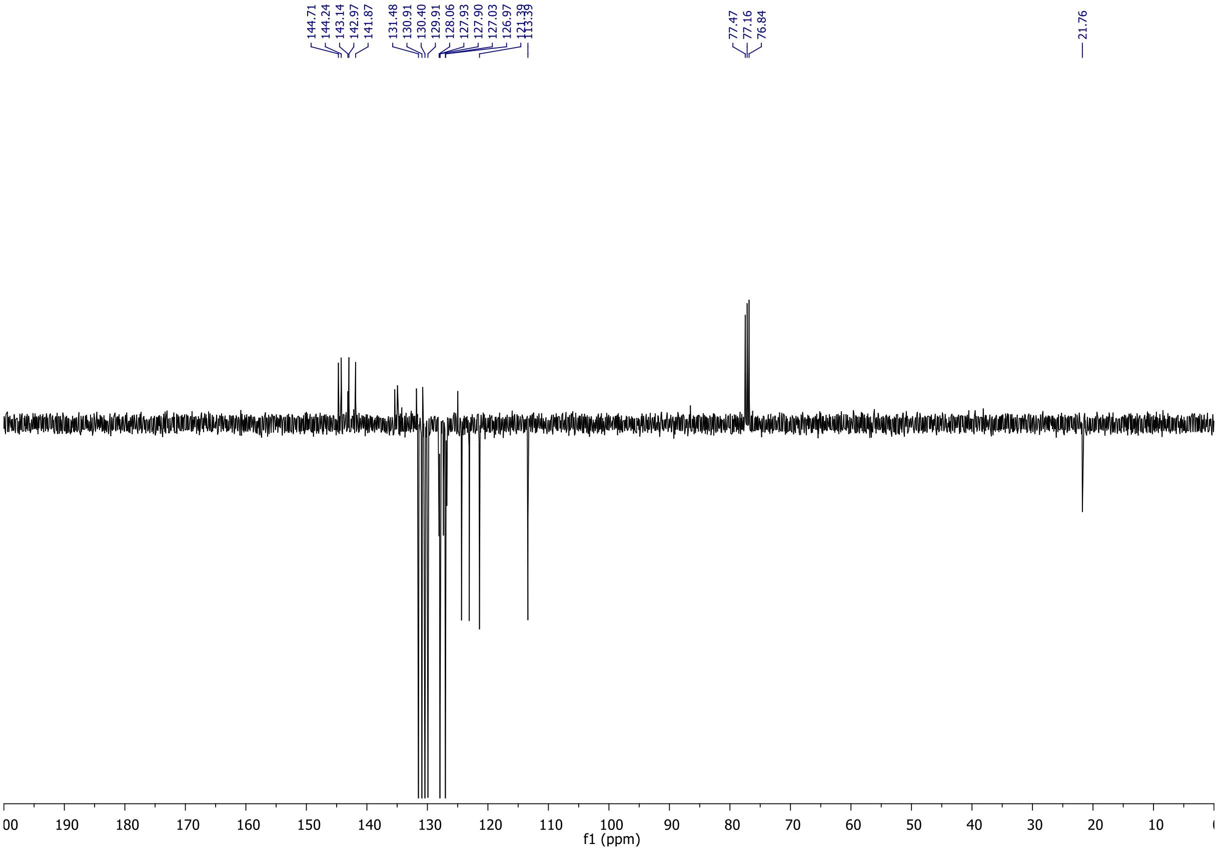

^{1}H NMR (400 MHz) and ^{13}C{^{1}H} (APT) NMR (100 MHz) spectra of **S57** ($CDCl_3$)

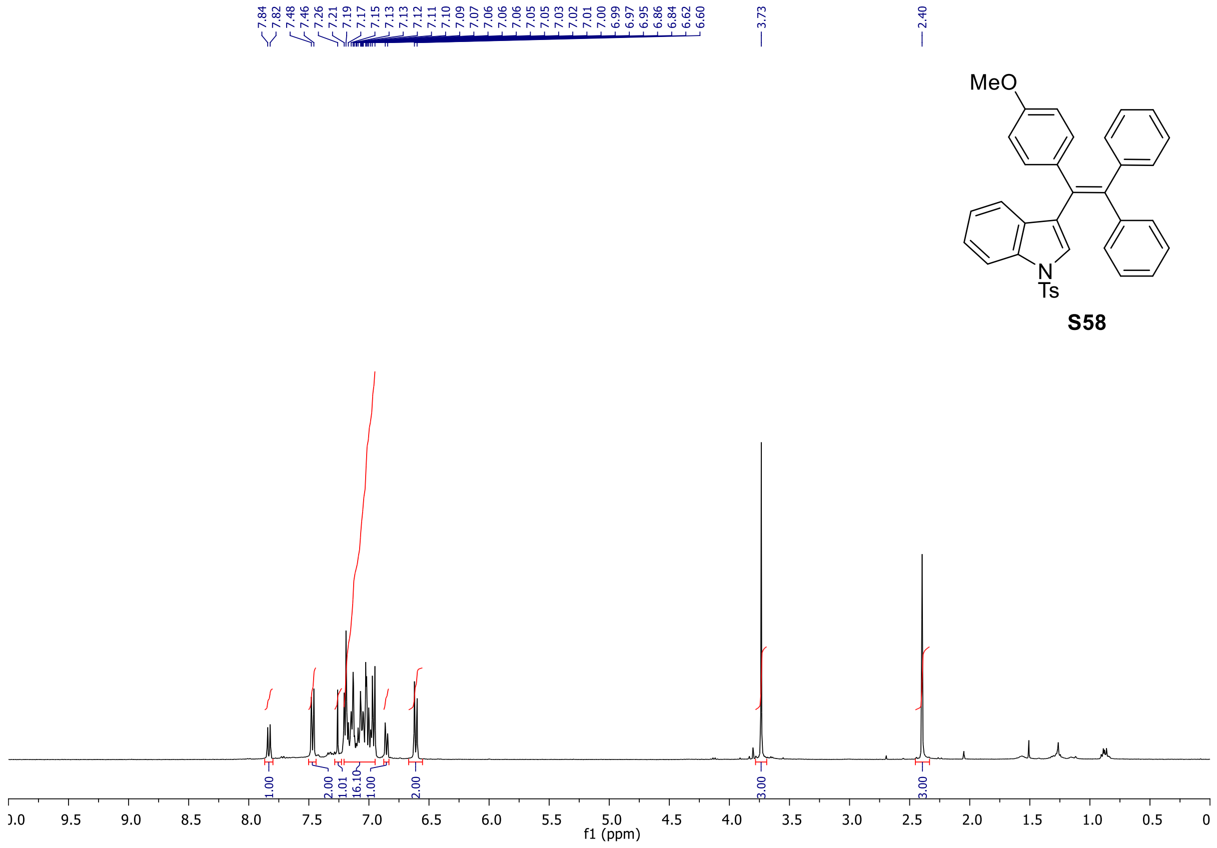

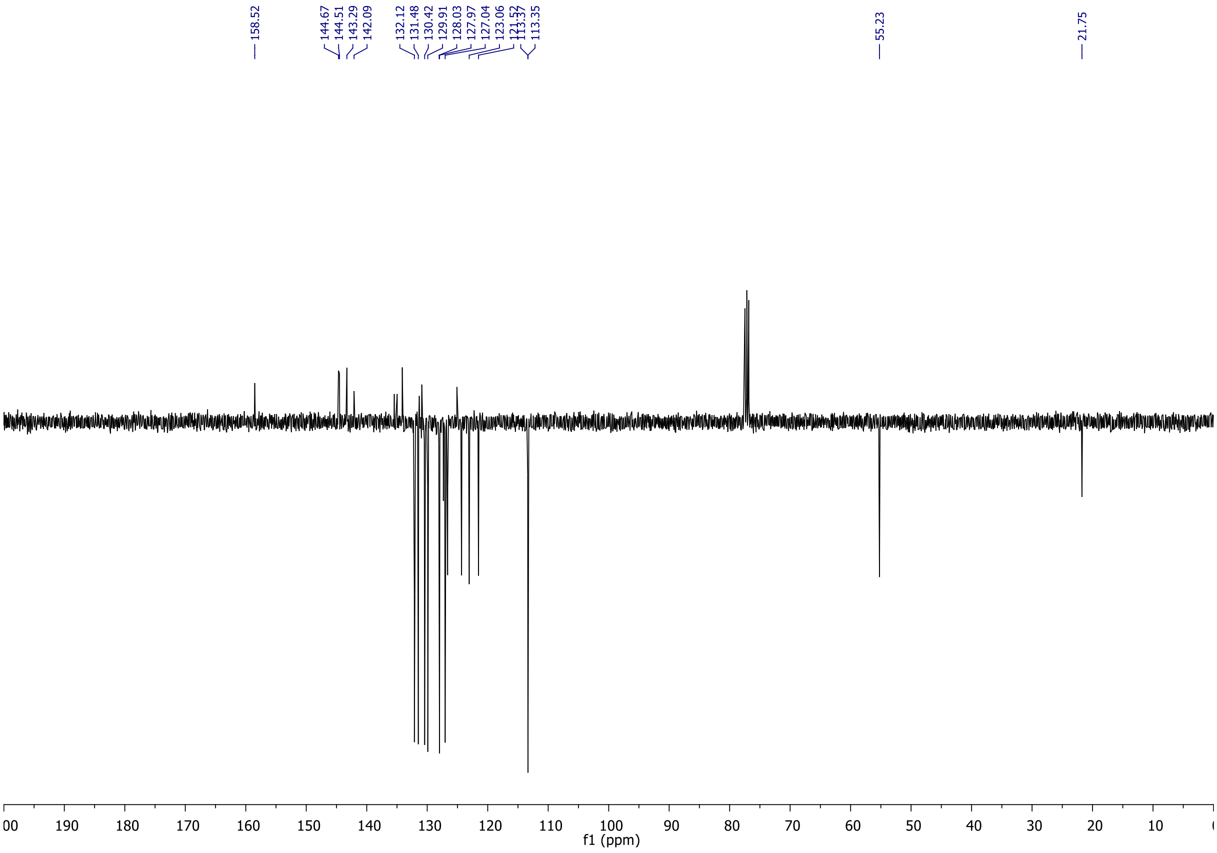

^{1}H NMR (400 MHz) and ^{13}C{^{1}H} (APT) NMR (100 MHz) spectra of **S58** ($CDCl_3$)

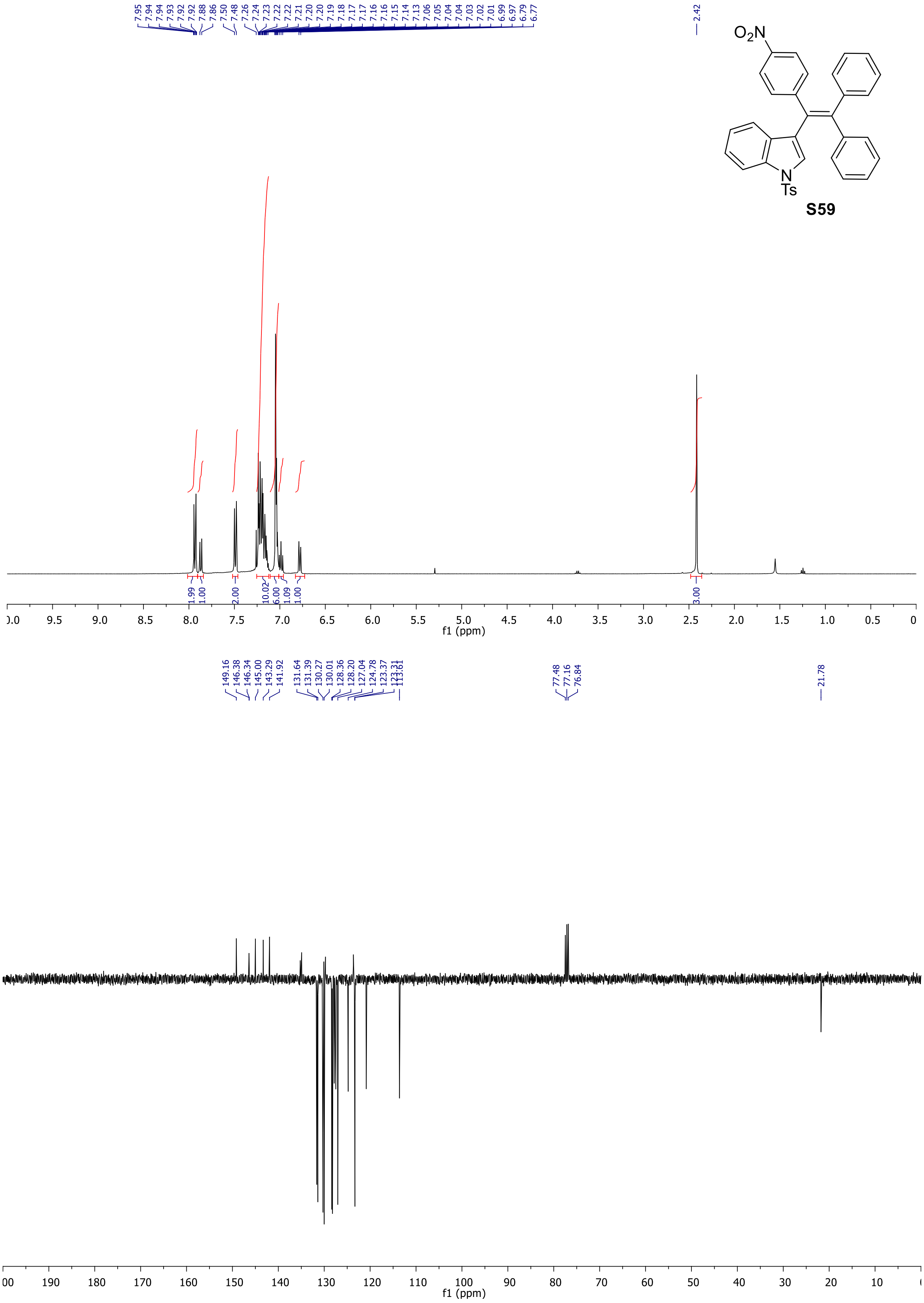

^{1}H NMR (400 MHz) and ^{13}C{^{1}H} (APT) NMR (100 MHz) spectra of **S59** ($CDCl_3$)

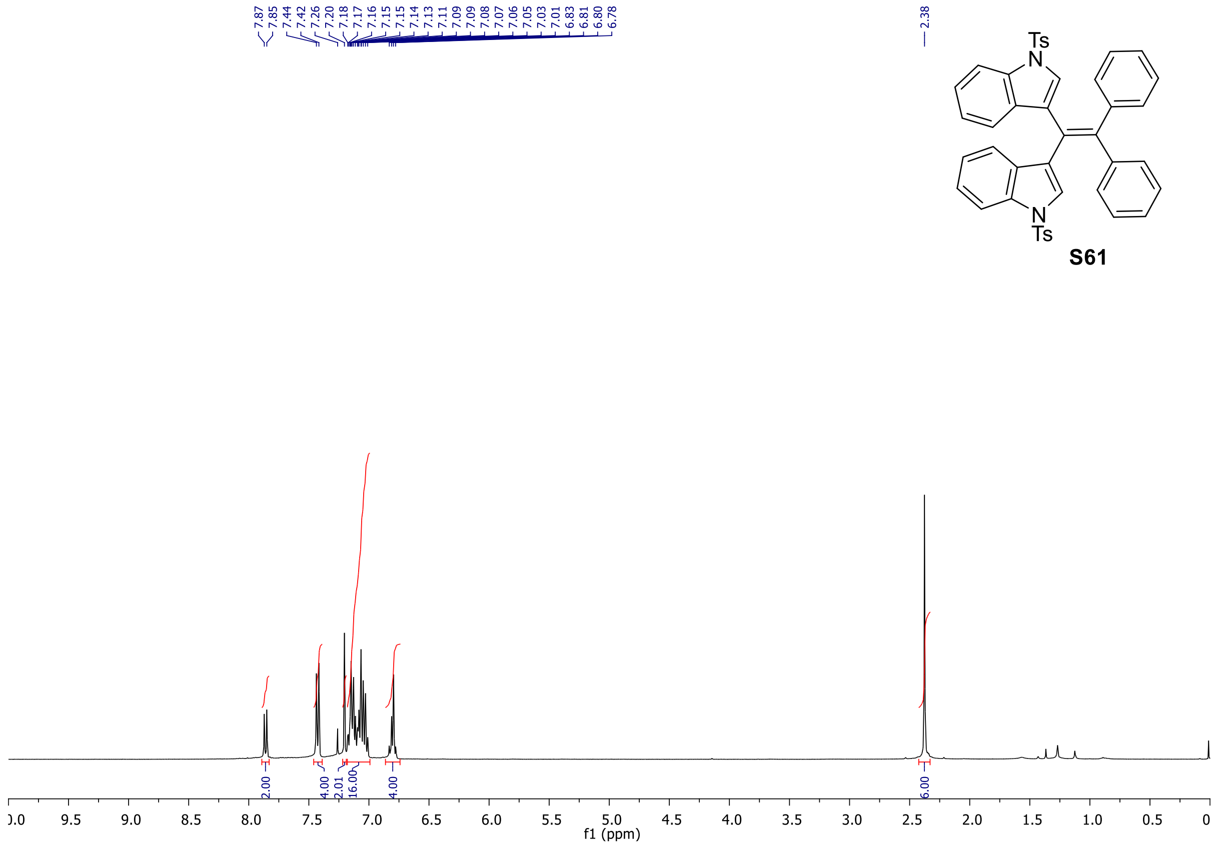

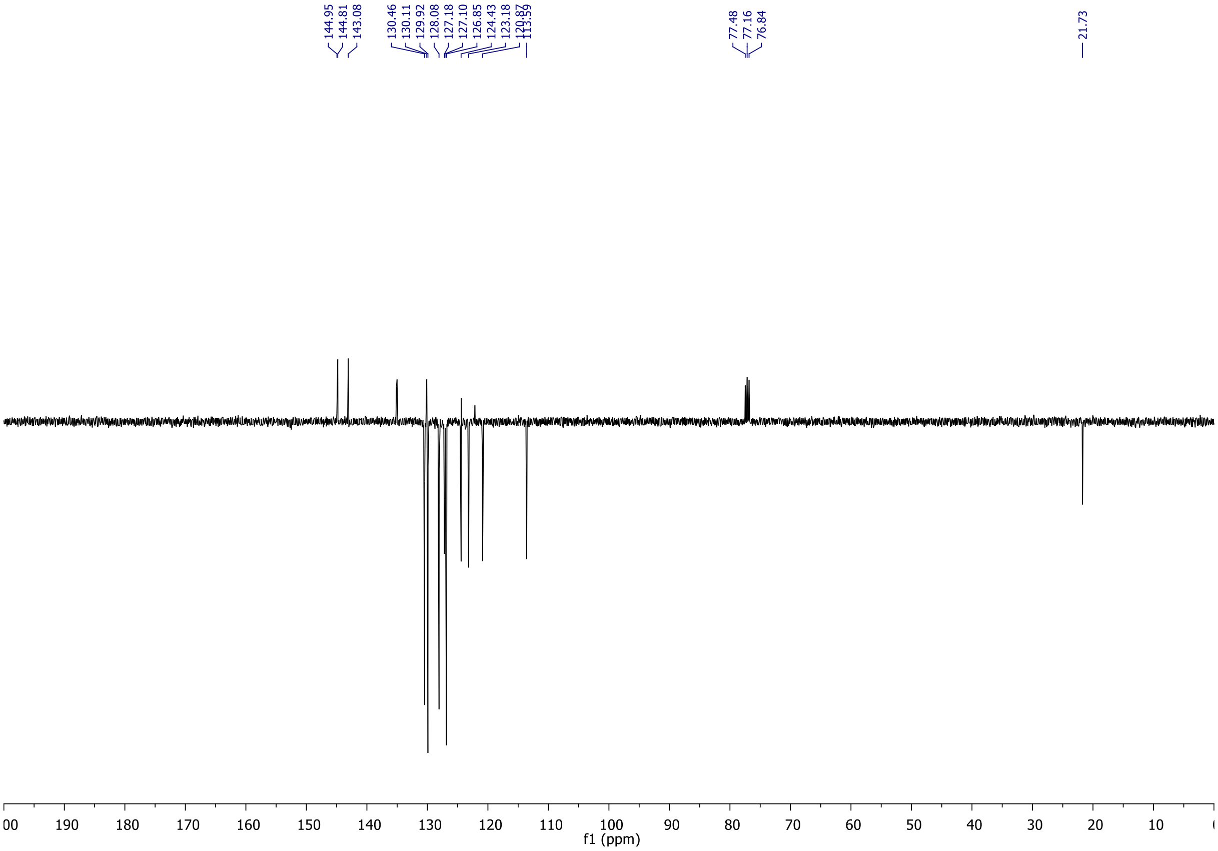

^{1}H NMR (400 MHz) and ^{13}C{^{1}H} (APT) NMR (100 MHz) spectra of **S61** ($CDCl_3$)

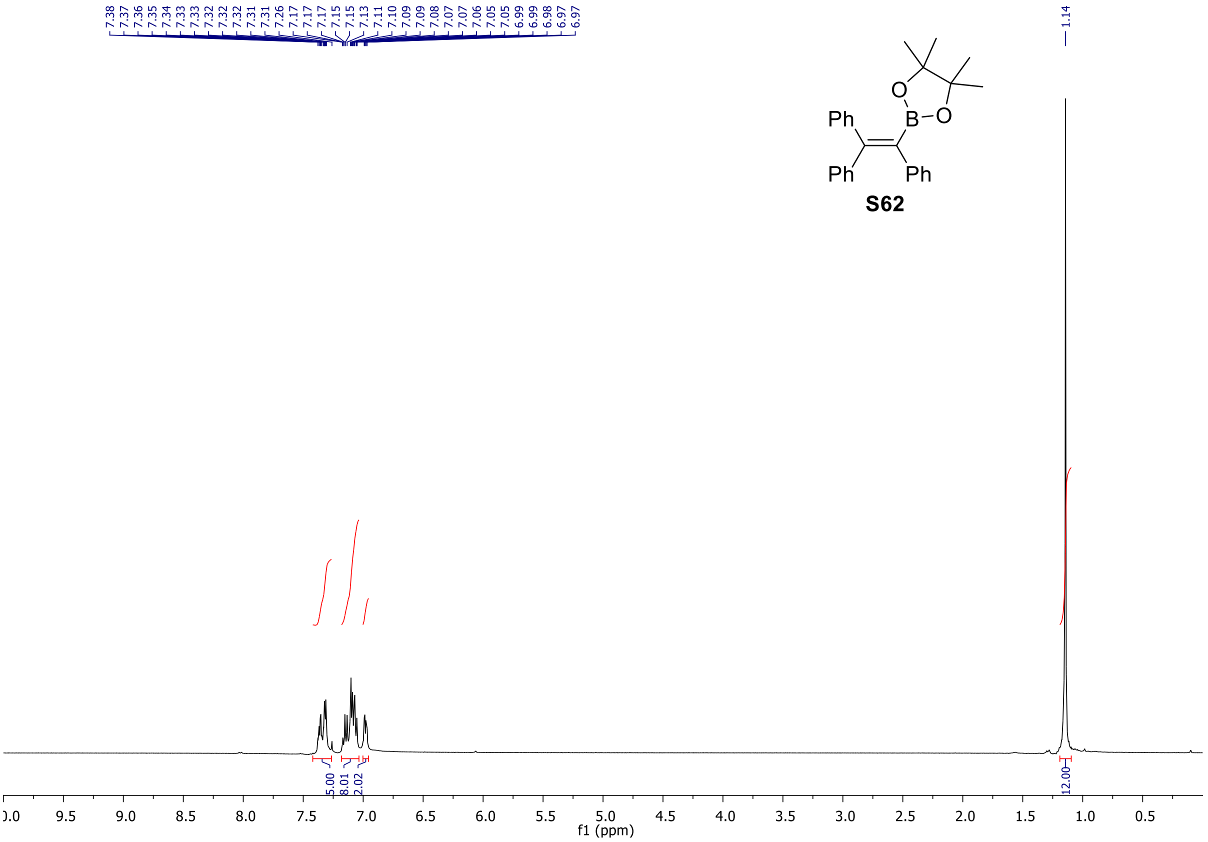

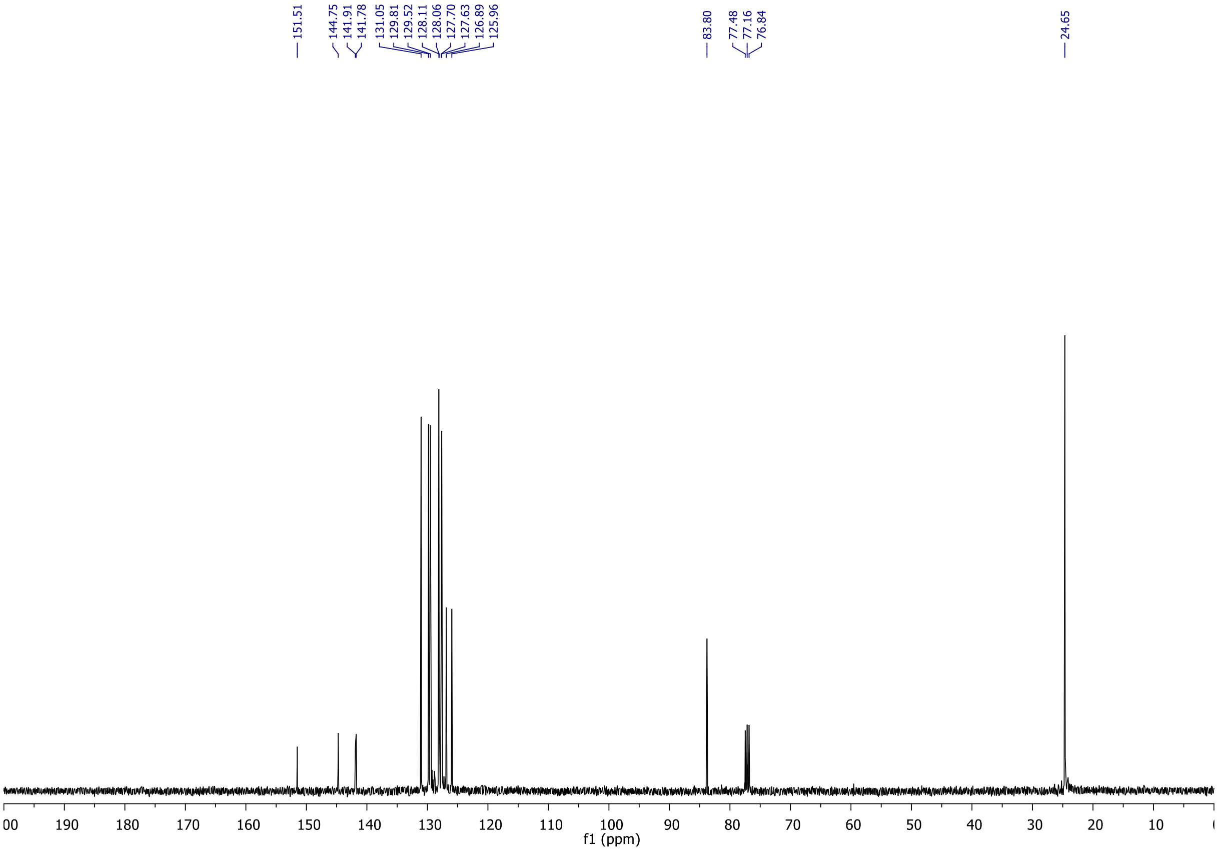

^{1}H NMR (400 MHz) and ^{13}C{^{1}H} NMR (100 MHz) spectra of **S62** ($CDCl_3$)

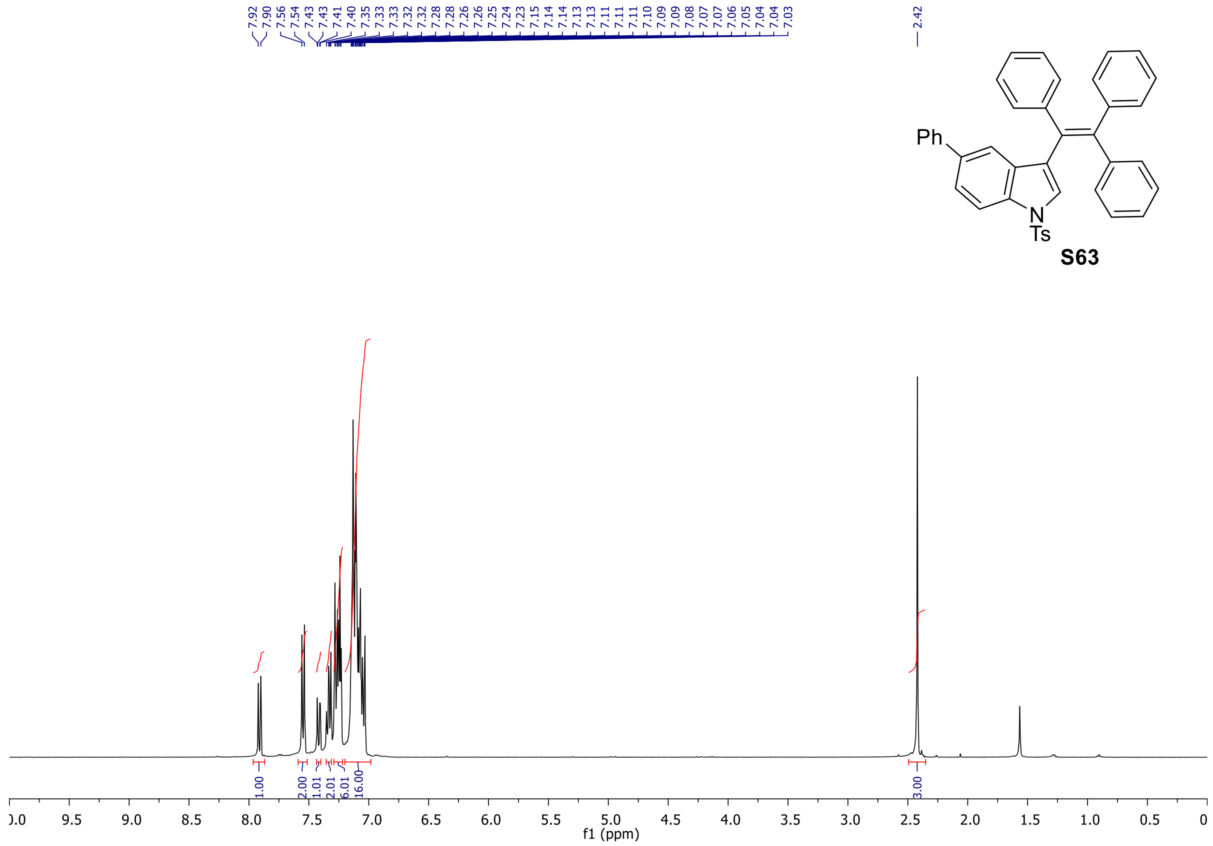

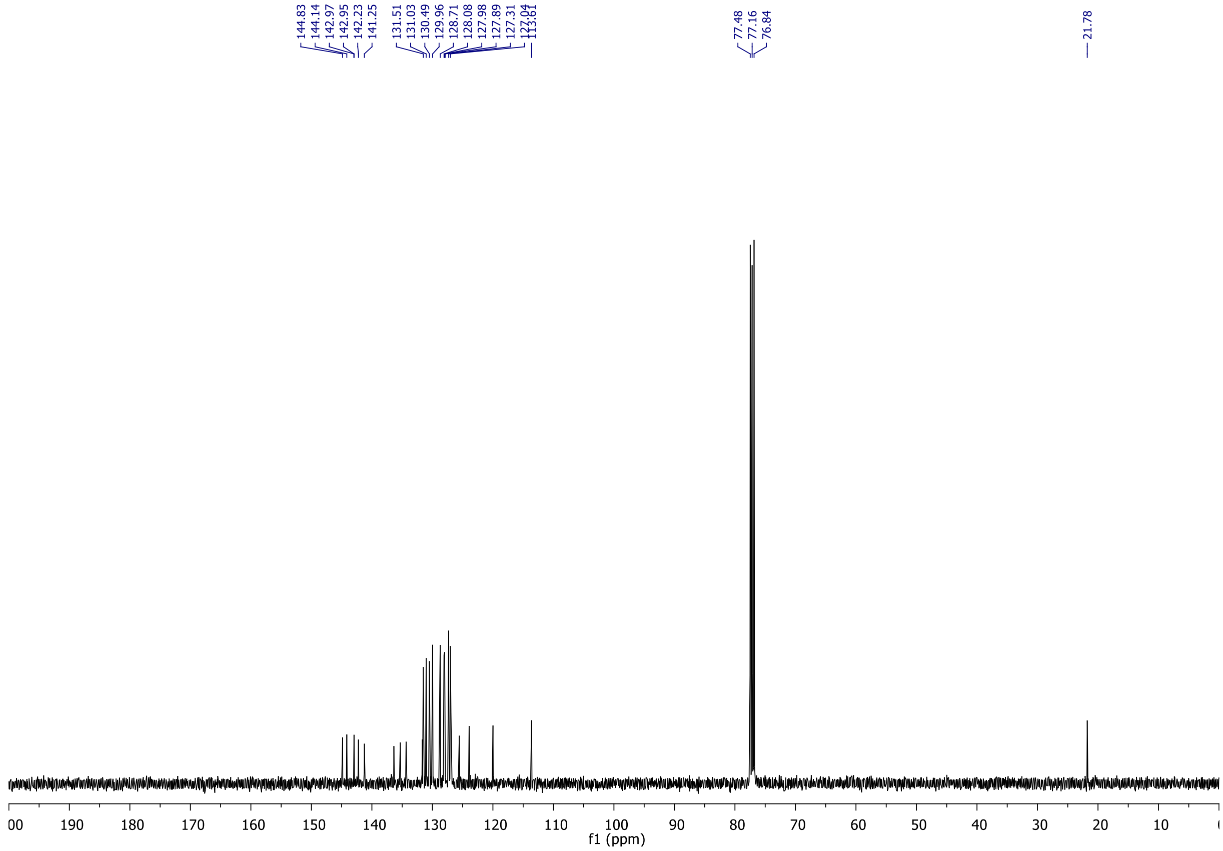

^{1}H NMR (400 MHz) and ^{13}C{^{1}H} NMR (100 MHz) spectra of **S63** ($CDCl_3$)

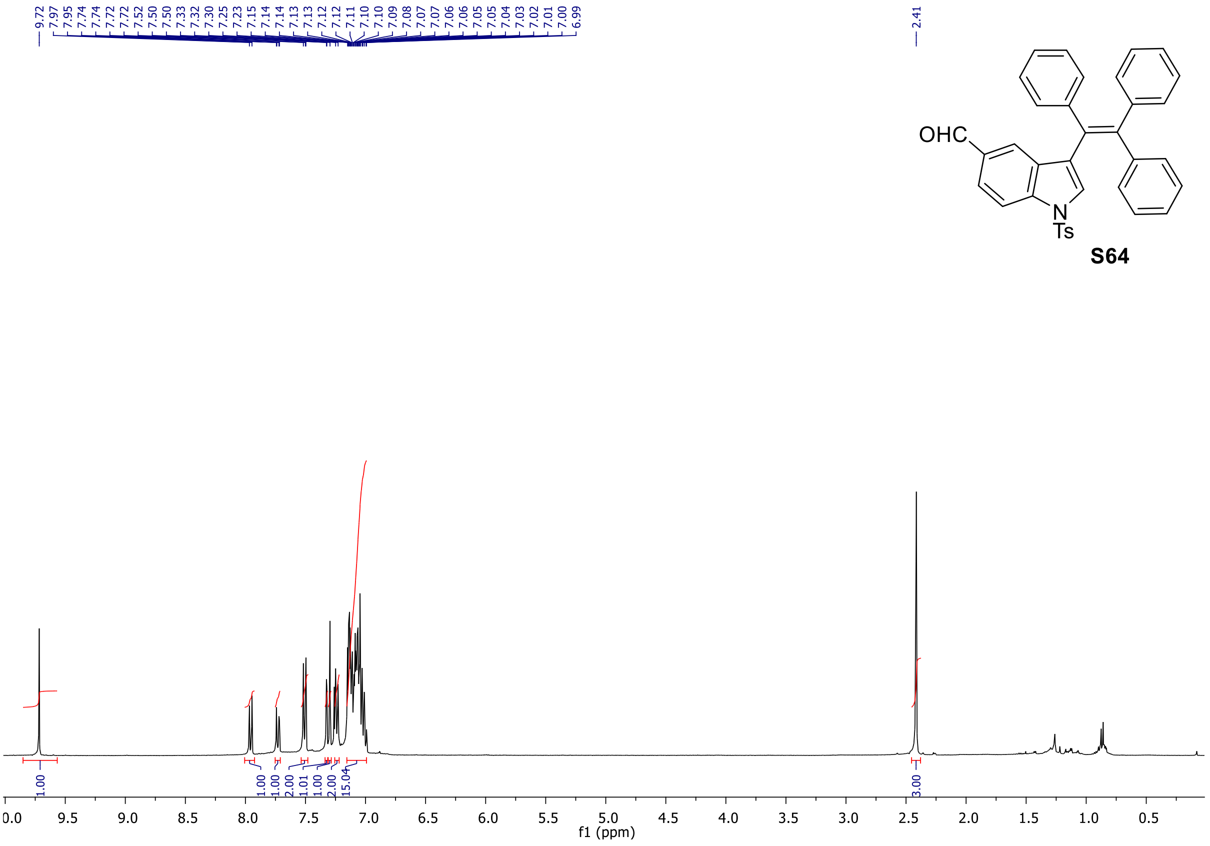

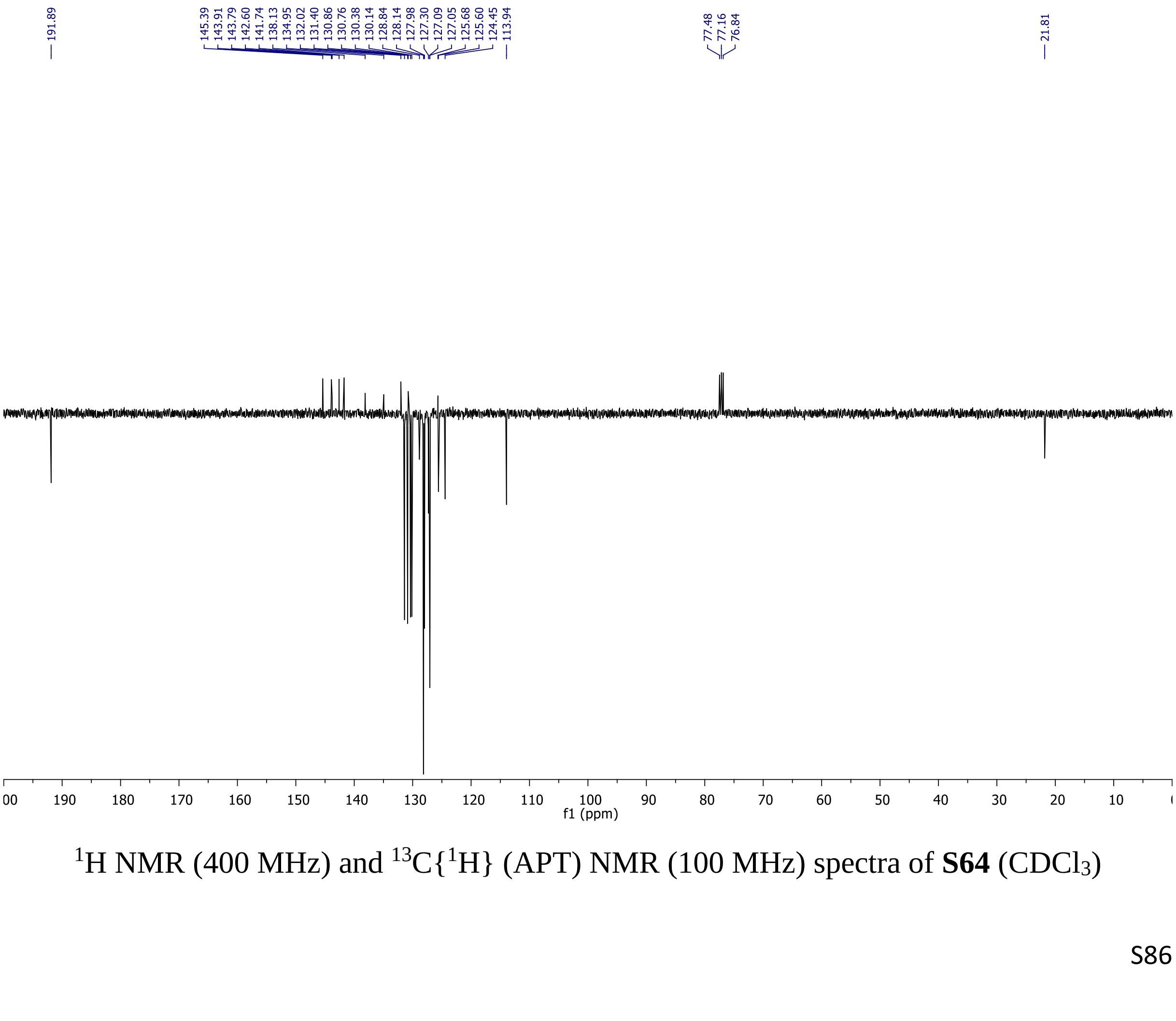

^{1}H NMR (400 MHz) and $^{13}C\{^{1}H\}$ (APT) NMR (100 MHz) spectra of **S64** ($CDCl_3$)

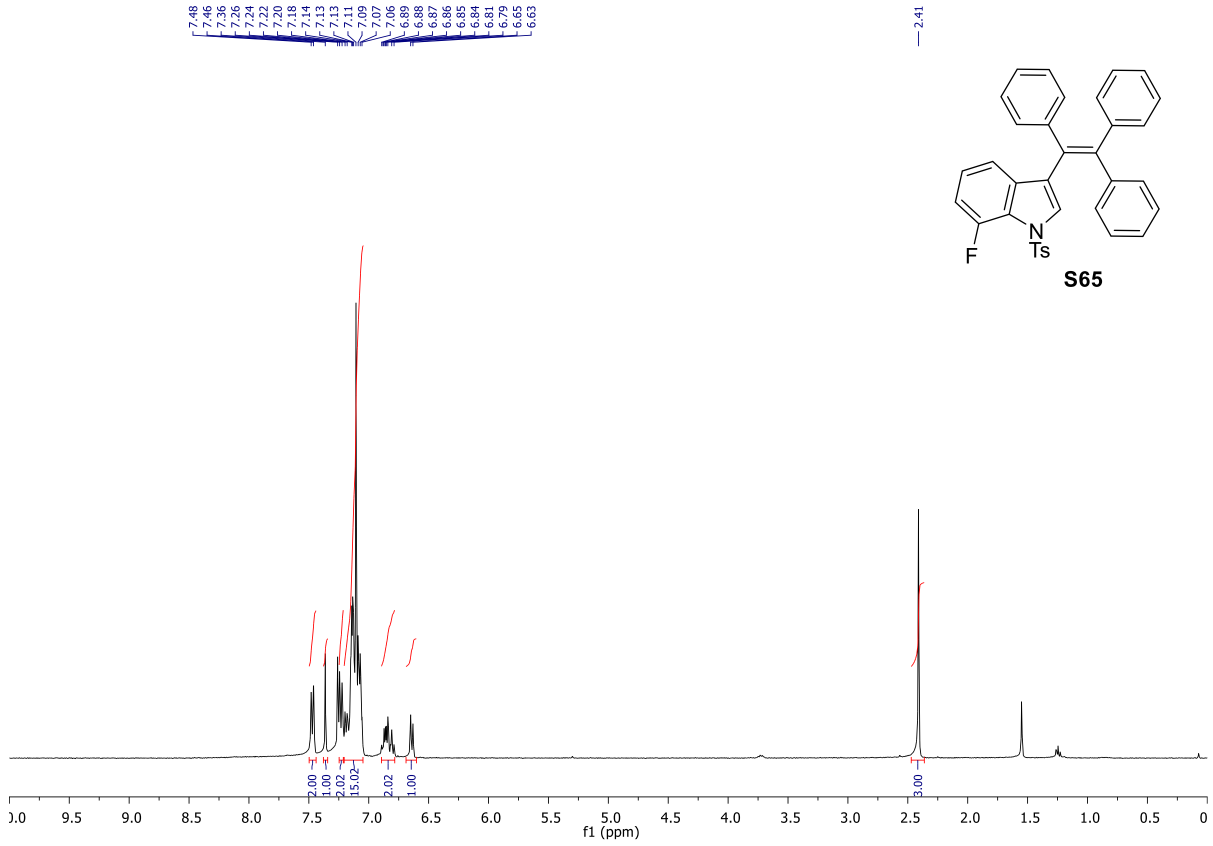

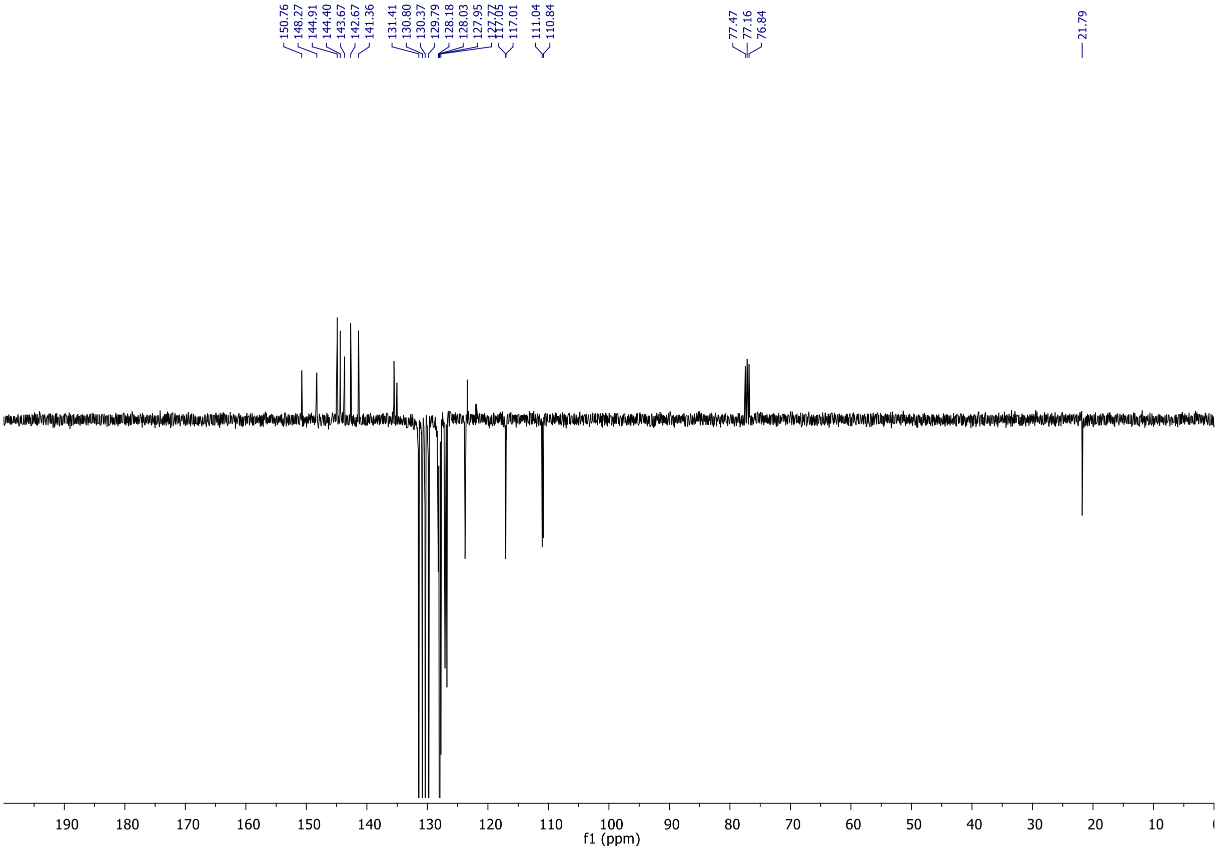

^{1}H NMR (400 MHz) and $^{13}C\{^{1}H\}$ (APT) NMR (100 MHz) spectra of **S65** ($CDCl_3$)

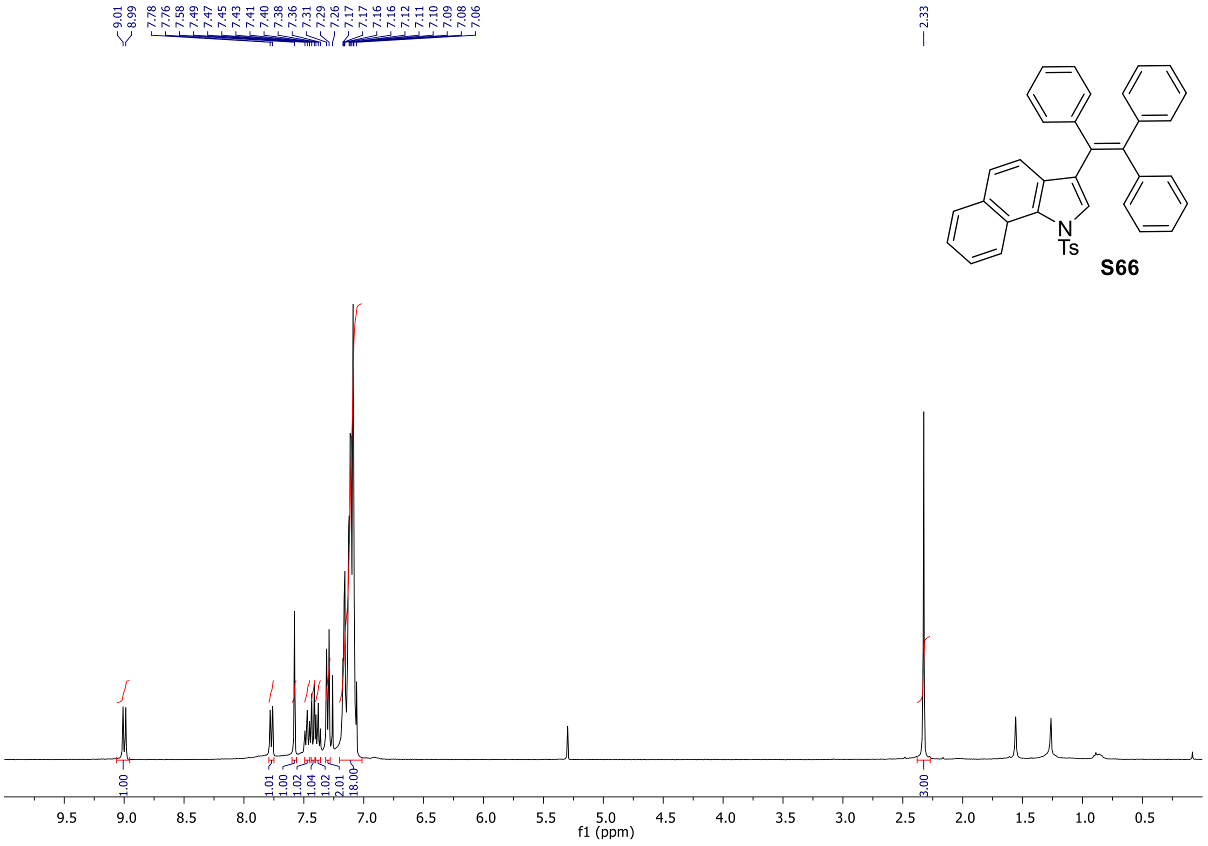

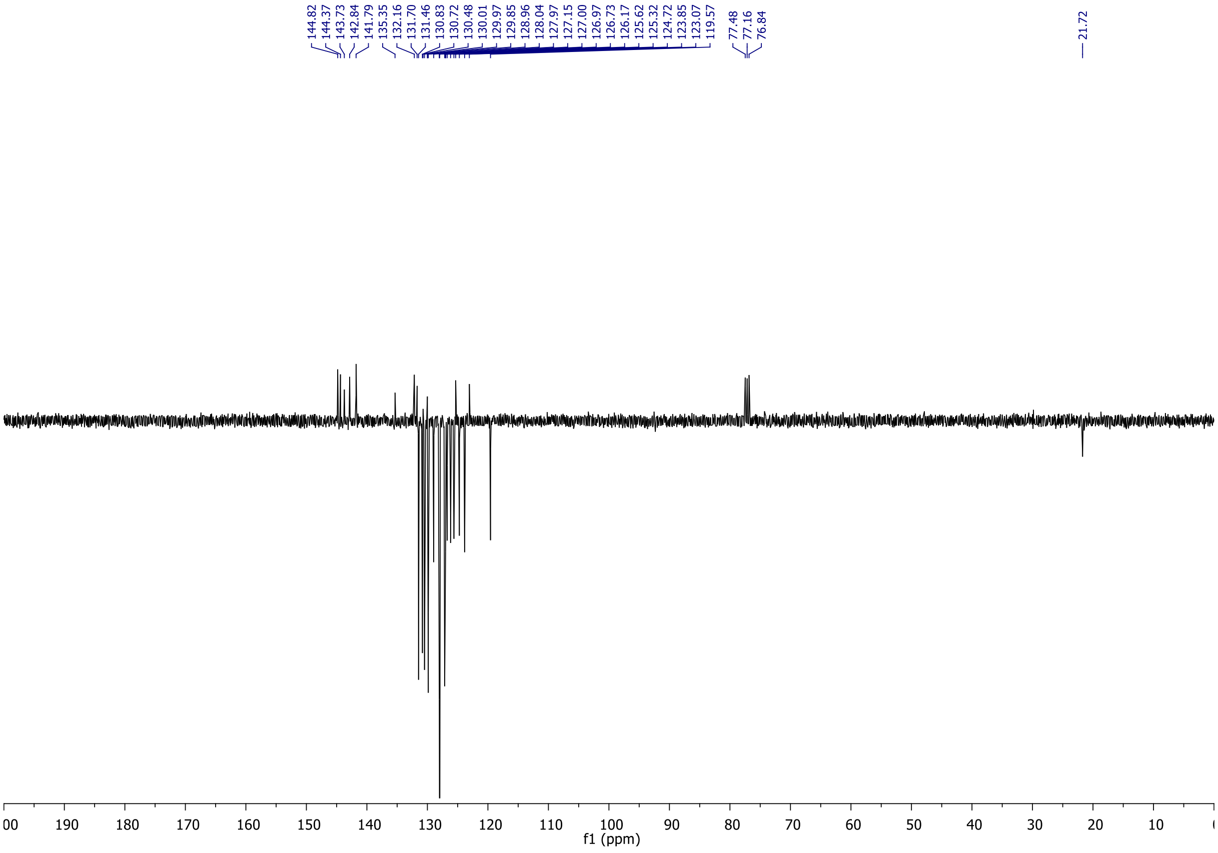

^{1}H NMR (400 MHz) and ^{13}C{^{1}H} (APT) NMR (100 MHz) spectra of **S66** ($CDCl_3$)

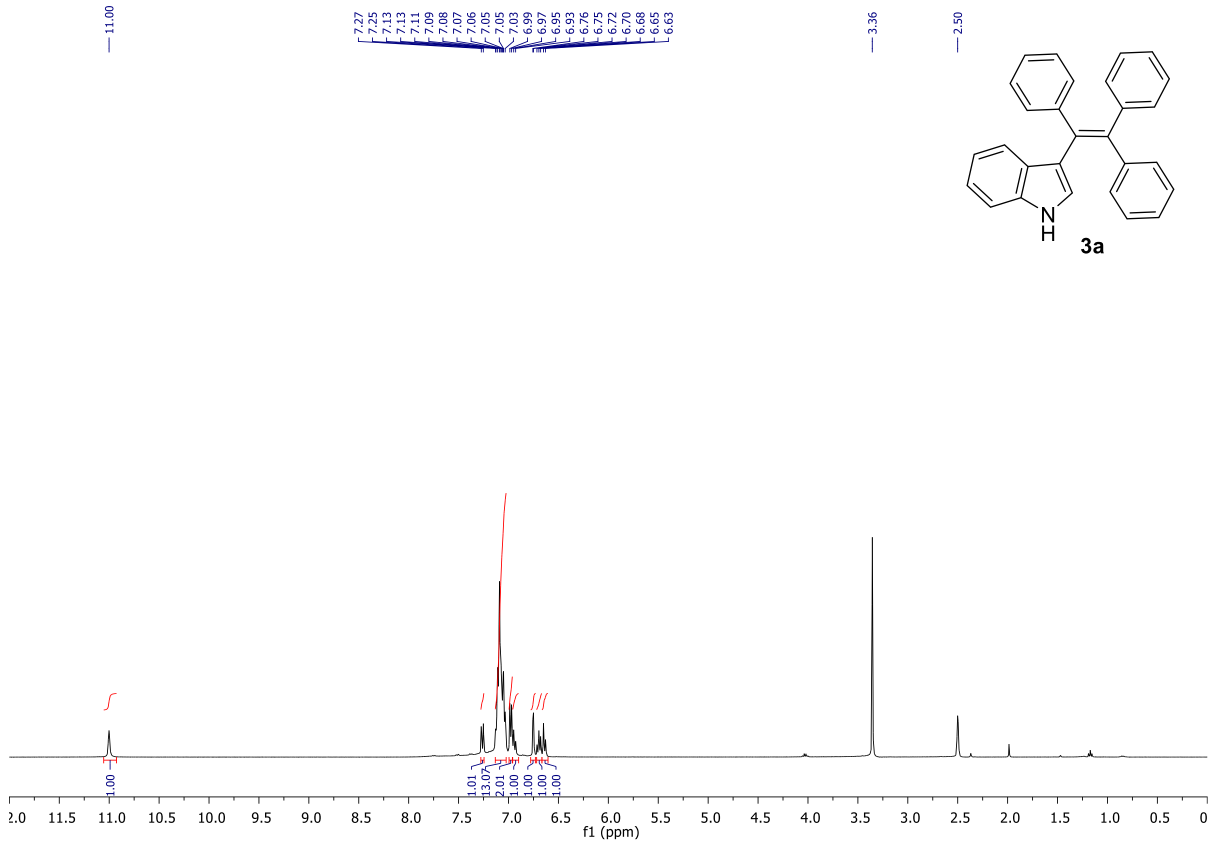

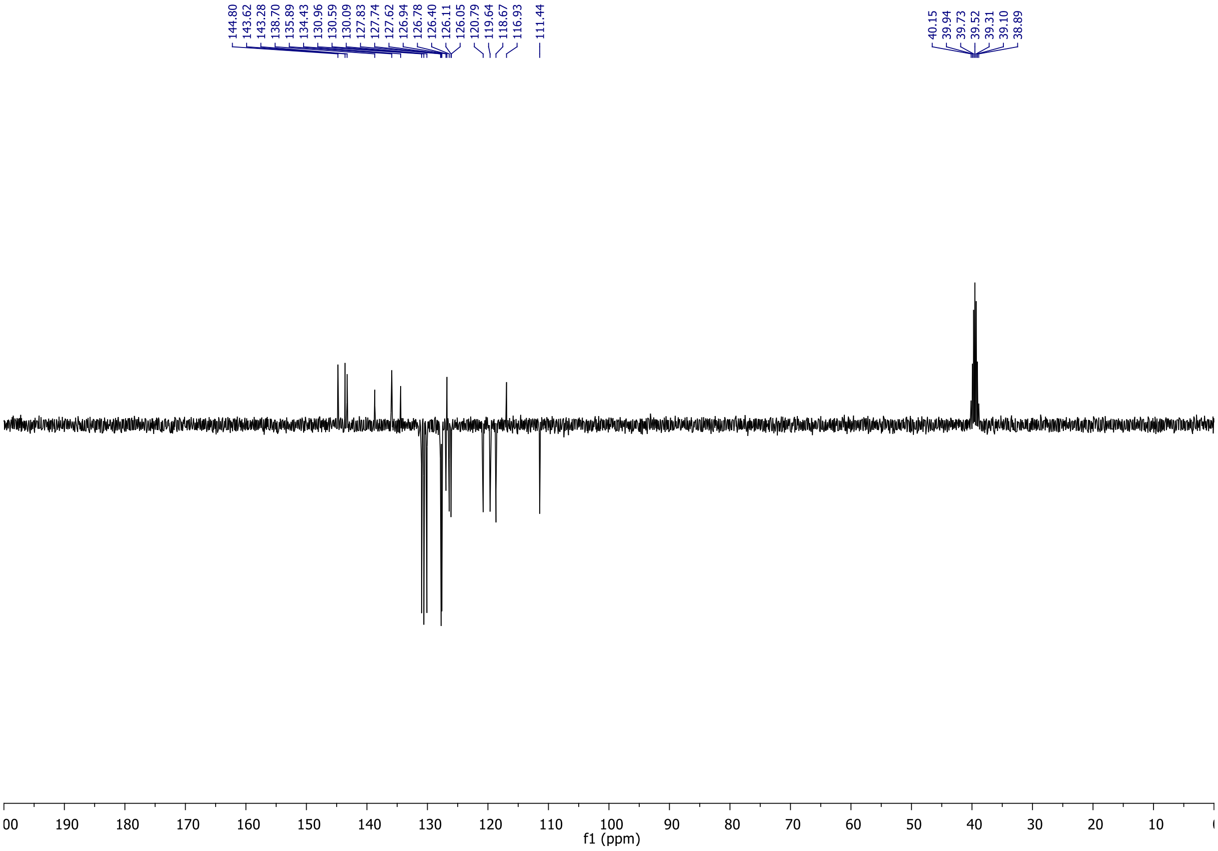

^{1}H NMR (400 MHz) and ^{13}C{^{1}H} (APT) NMR (100 MHz) spectra of **3a** (DMSO-d_6)

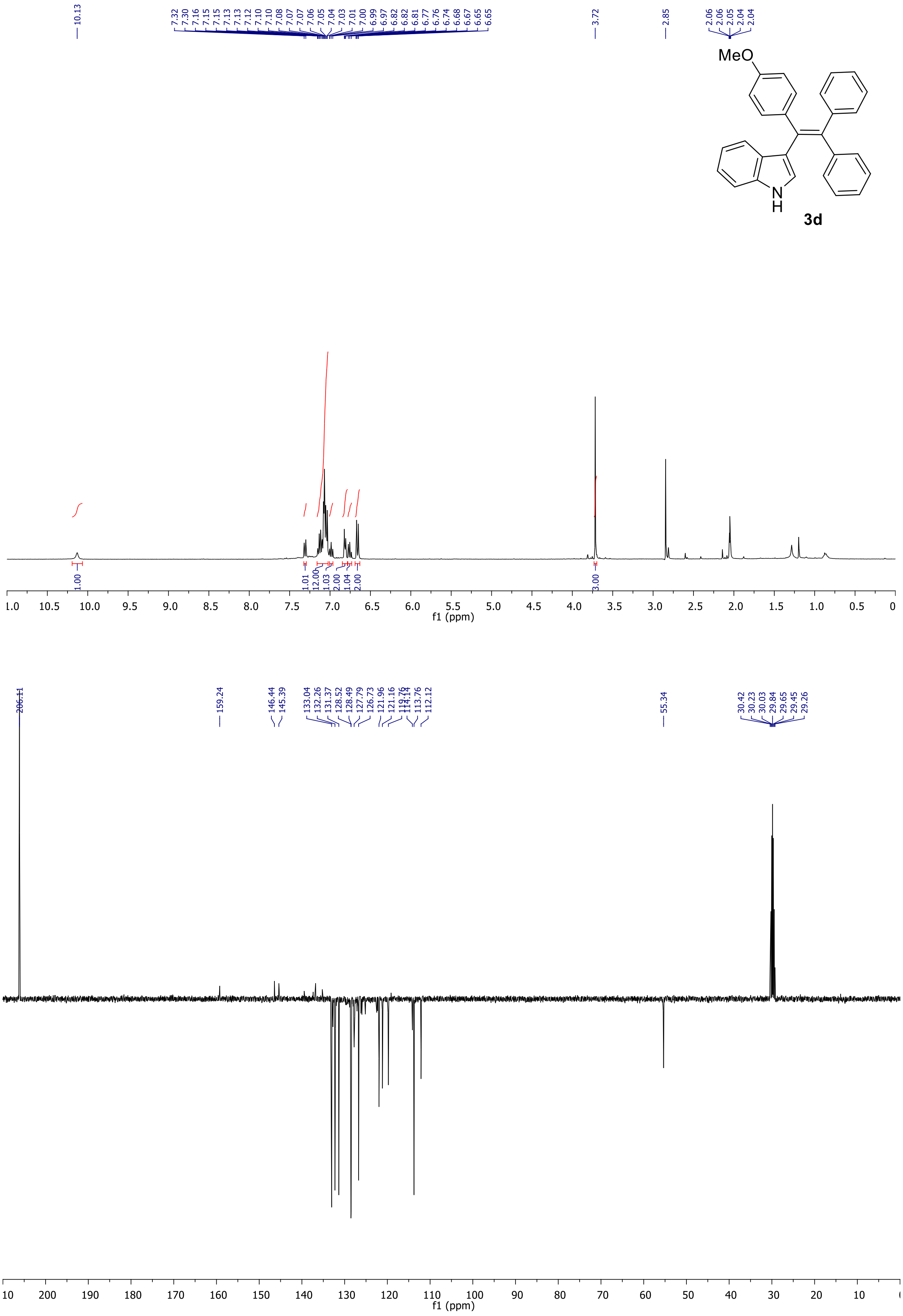

^{1}H NMR (400 MHz) and ^{13}C{^{1}H} (APT) NMR (100 MHz) spectra of **3d** (Acetone-d_6)

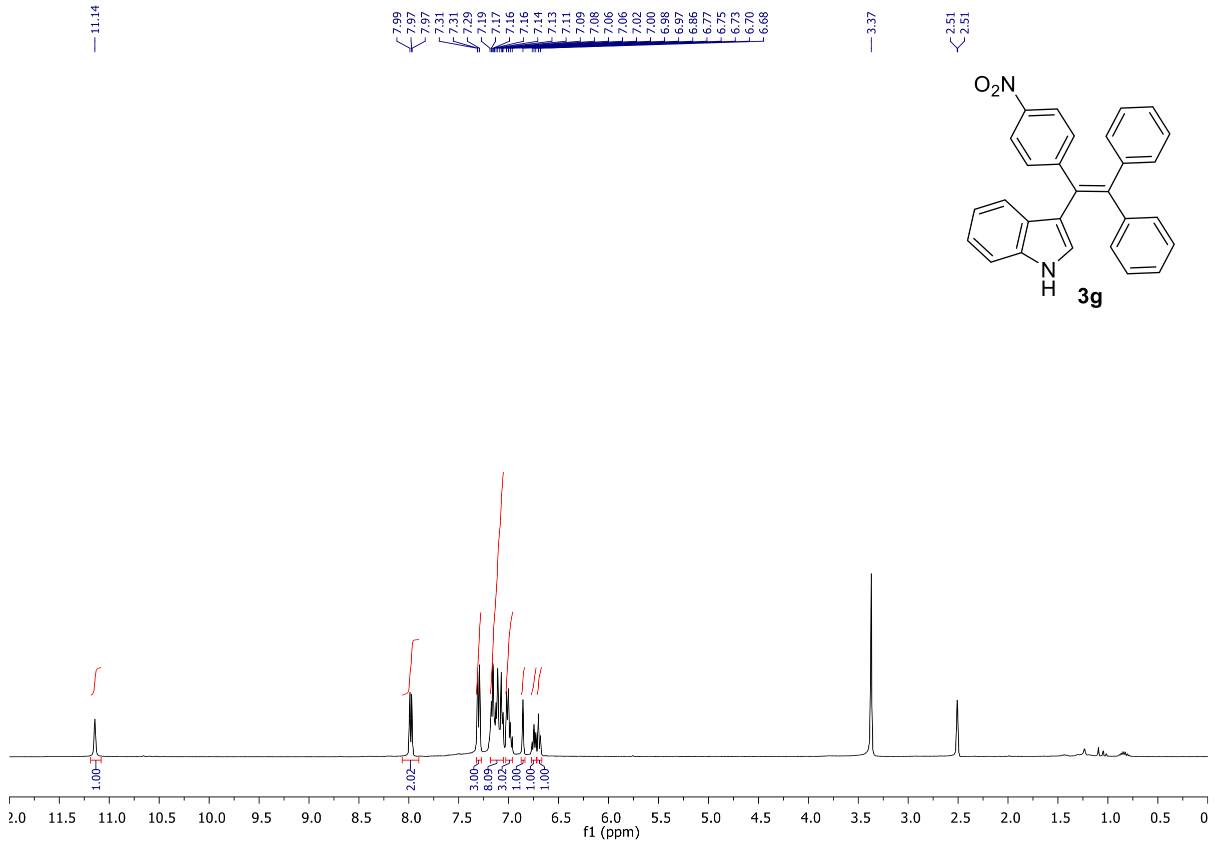

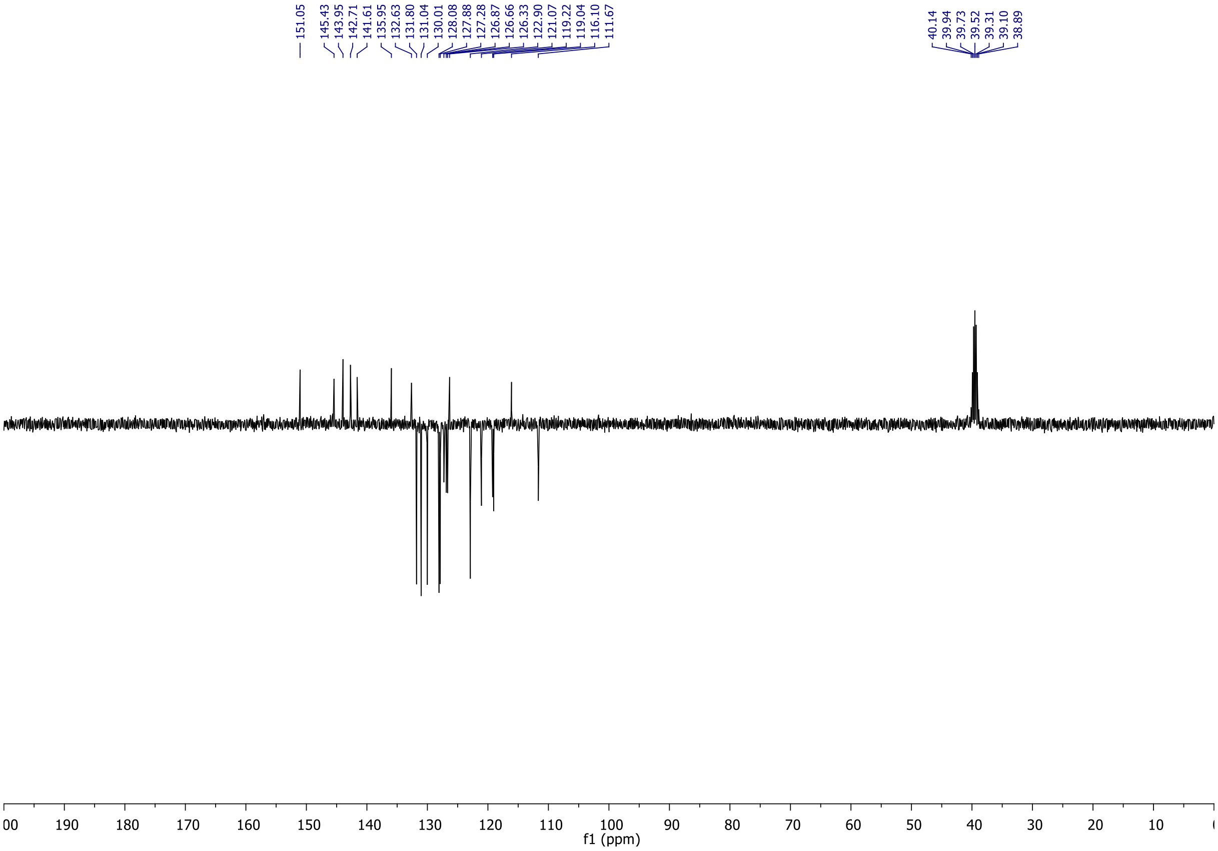

^{1}H NMR (400 MHz) and ^{13}C{^{1}H} (APT) NMR (100 MHz) spectra of **3g** (DMSO-d_6)

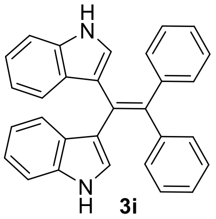

3i

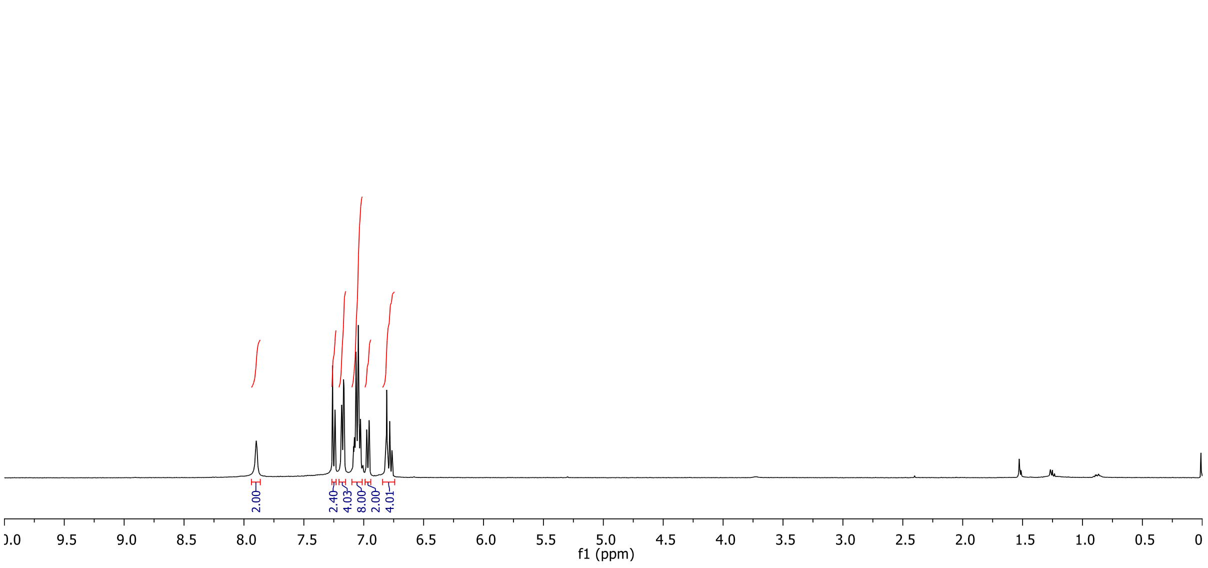

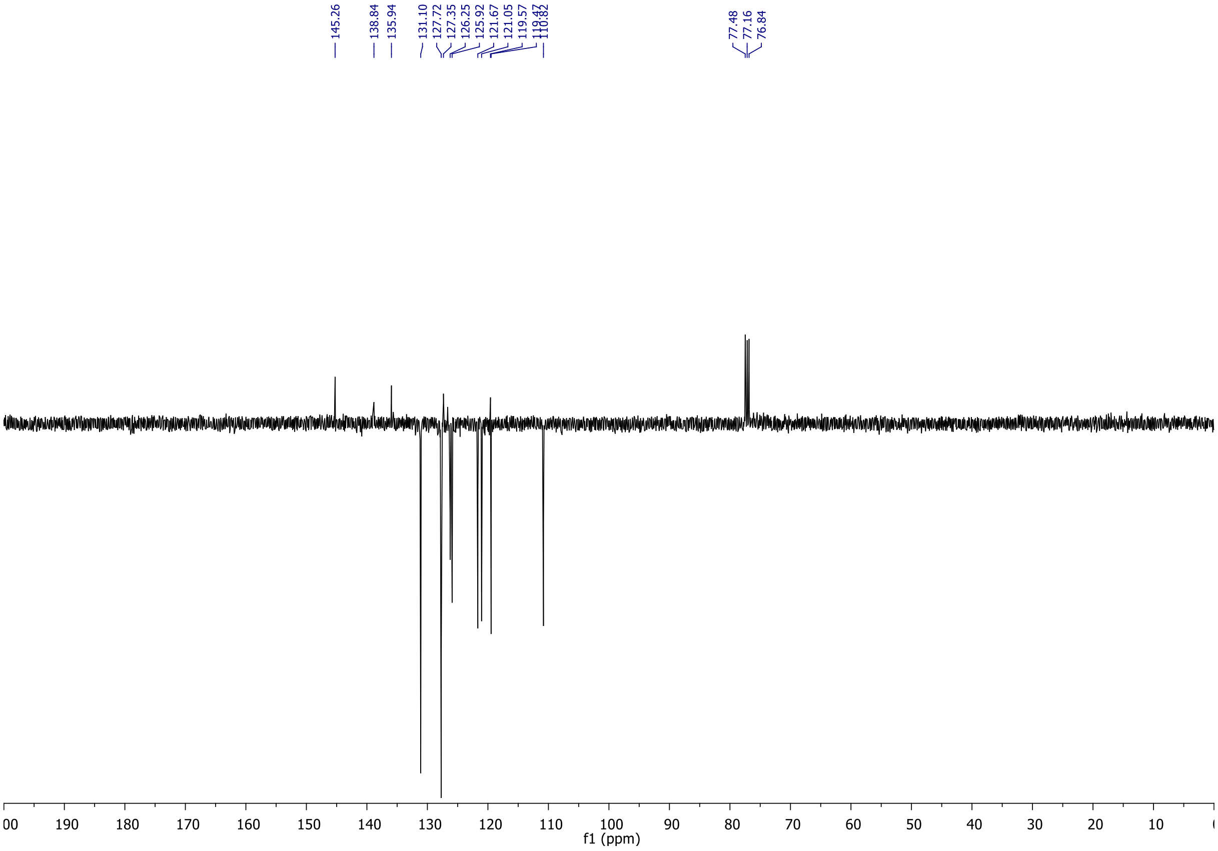

^{1}H NMR (400 MHz) and ^{13}C{^{1}H} (APT) NMR (100 MHz) spectra of **3i** ($CDCl_3$)

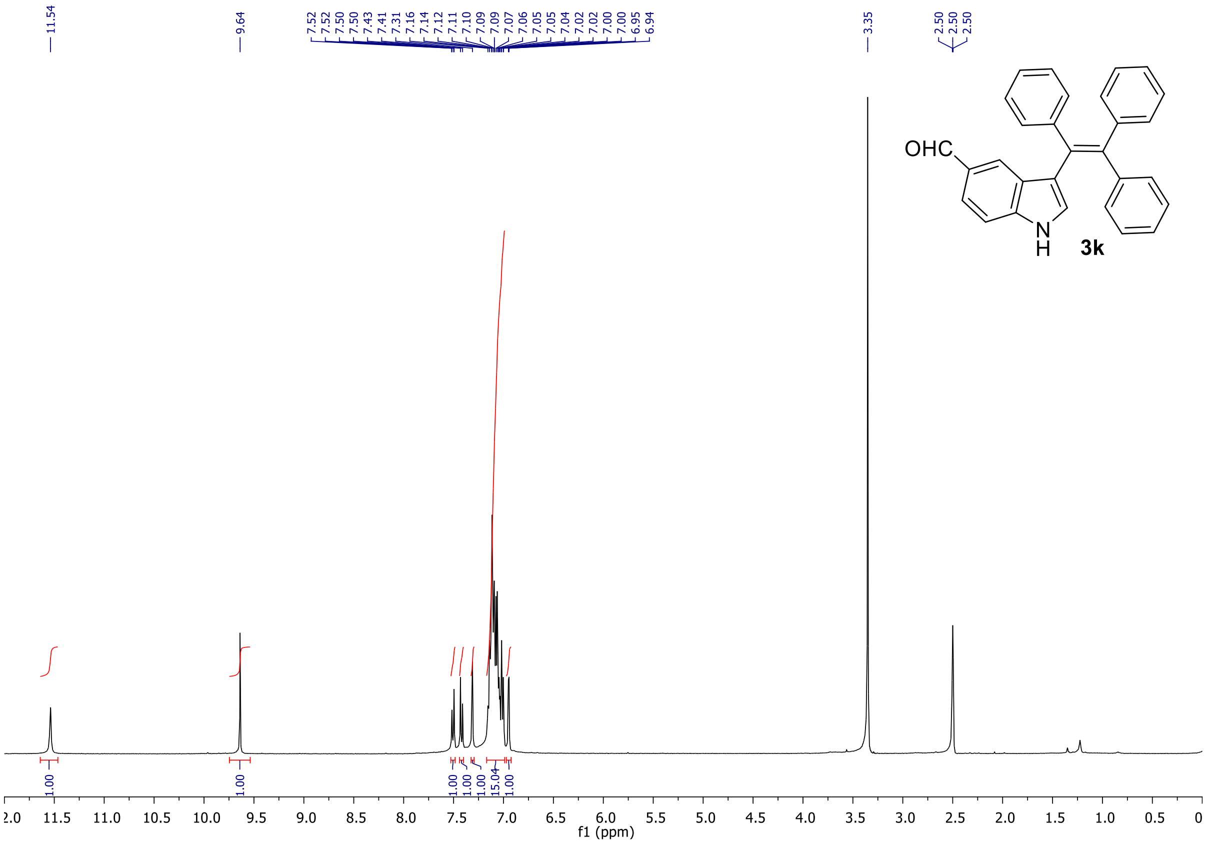

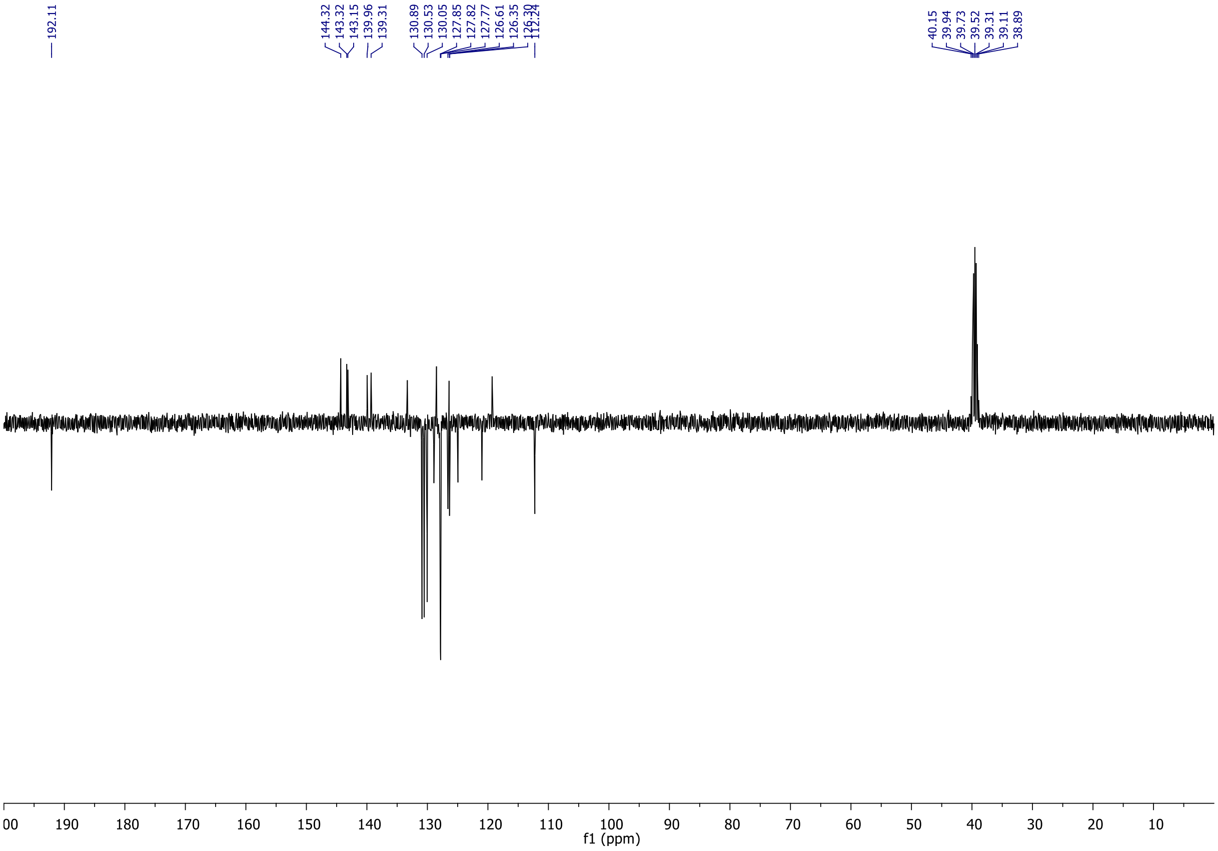

^{1}H NMR (400 MHz) and ^{13}C{^{1}H} (APT) NMR (100 MHz) spectra of **3k** (DMSO-d_6)

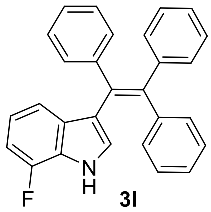

3l

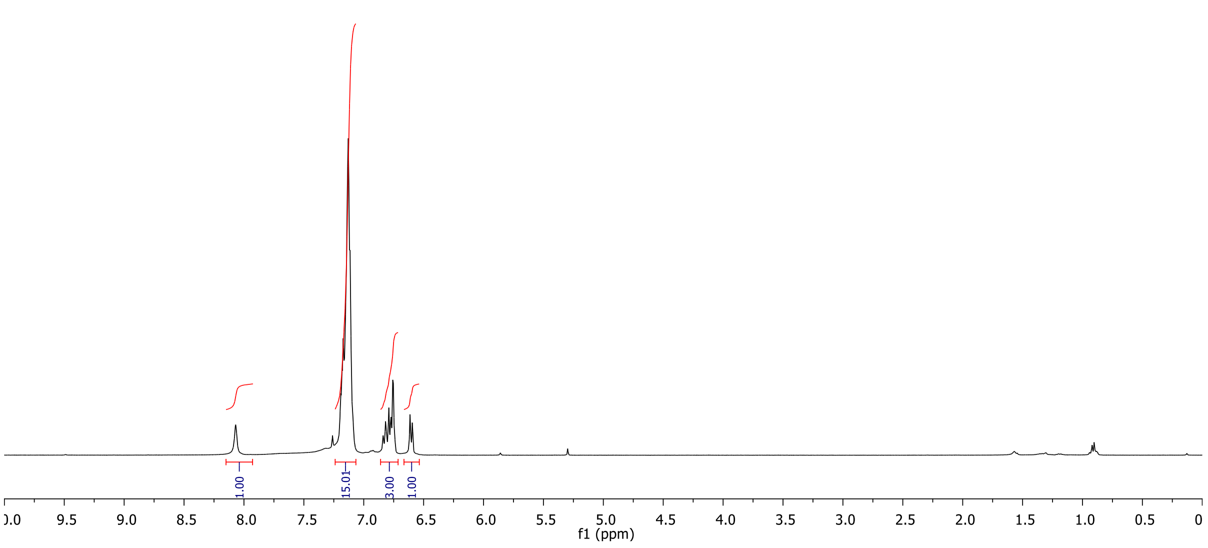

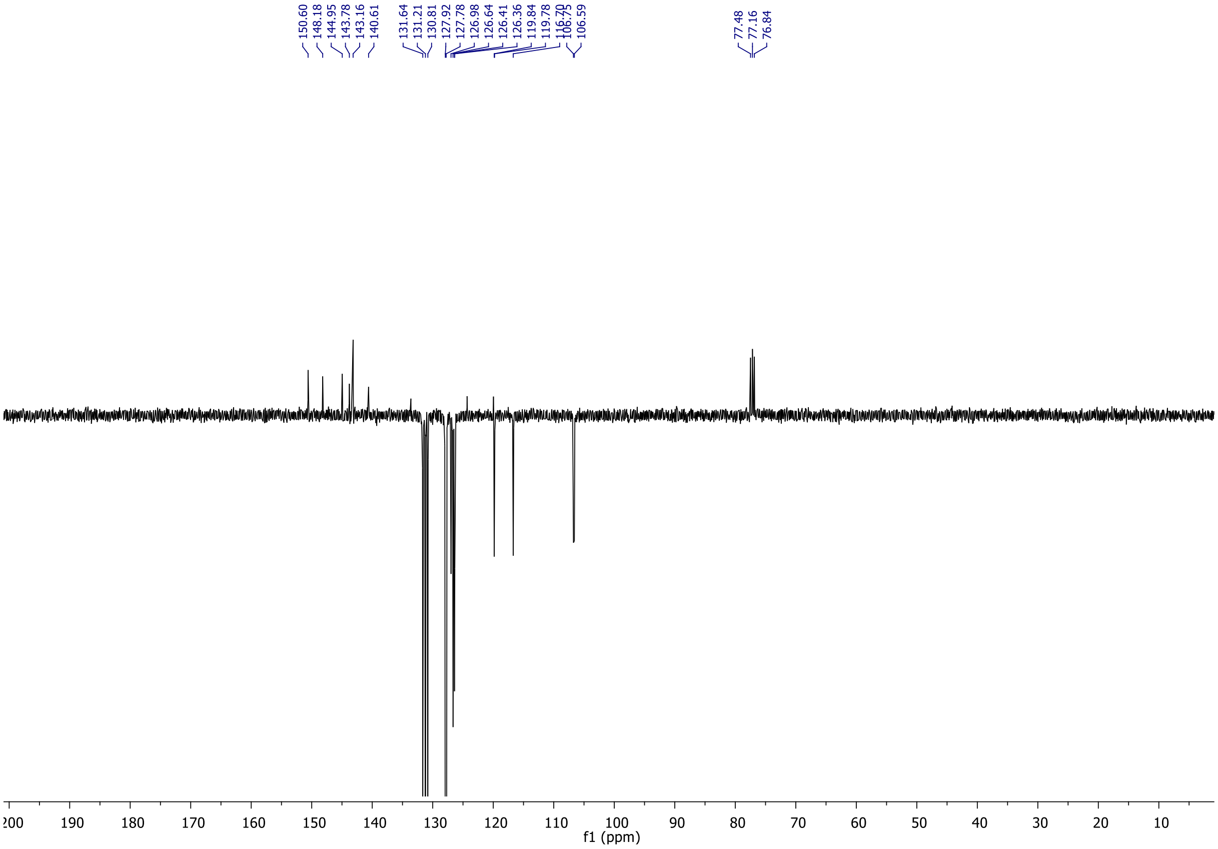

^{1}H NMR (400 MHz) and ^{13}C{^{1}H} (APT) NMR (100 MHz) spectra of **3l** ($CDCl_3$)

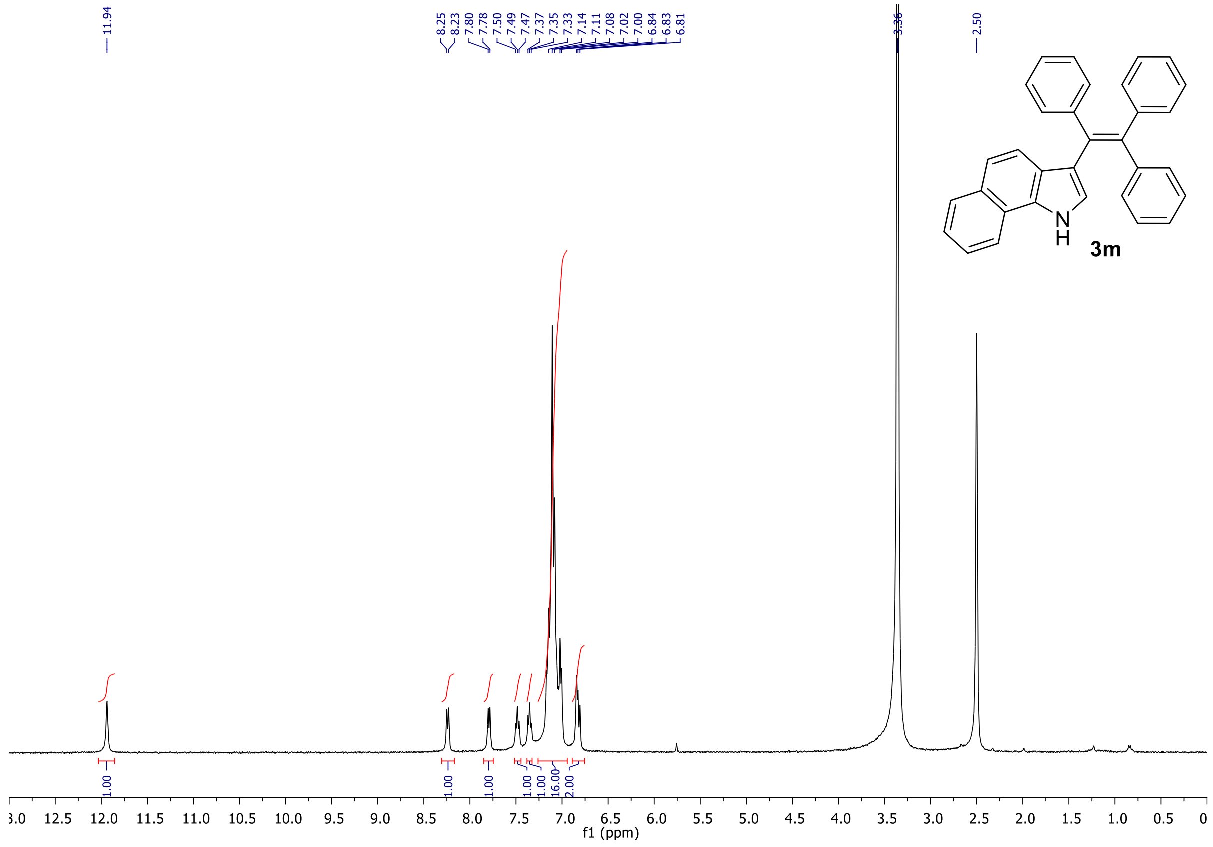

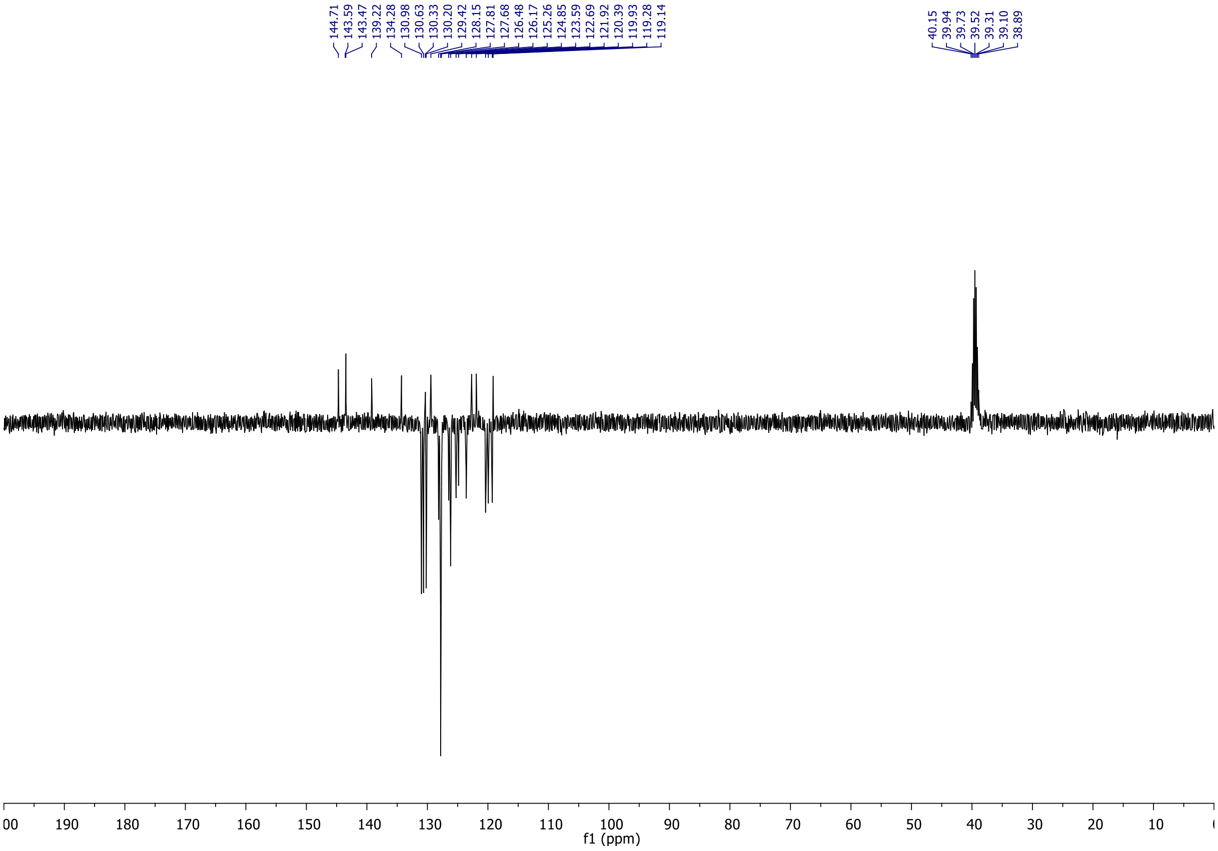

^{1}H NMR (400 MHz) and ^{13}C{^{1}H} (APT) NMR (100 MHz) spectra of **3m** (DMSO-d_6)

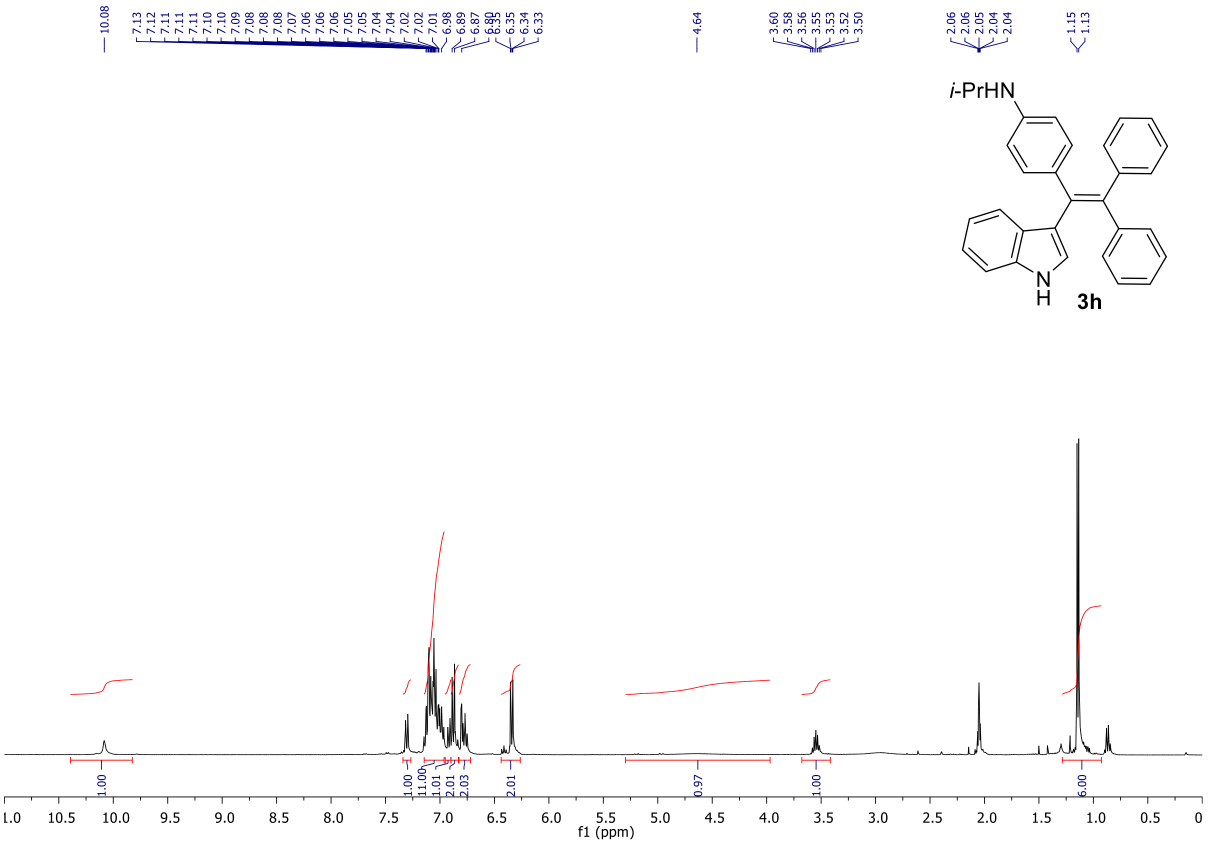

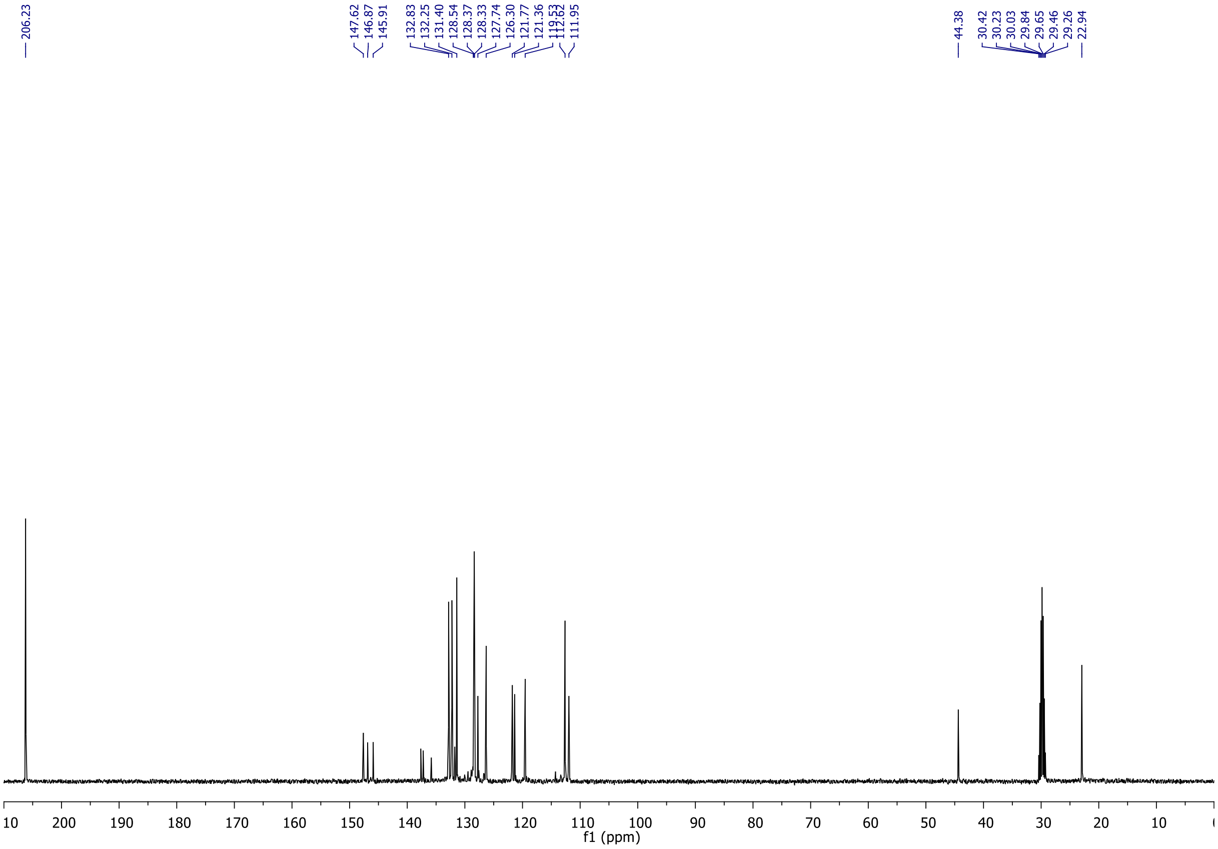

^{1}H NMR (400 MHz) and ^{13}C{^{1}H} NMR (100 MHz) spectra of **3h** (Acetone-d_6)

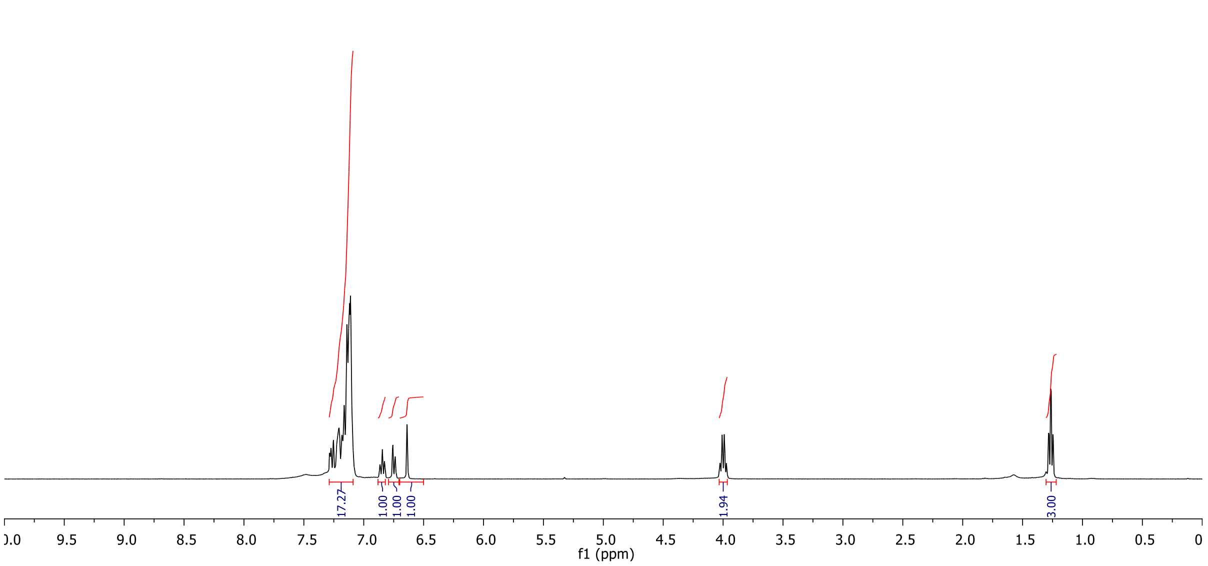

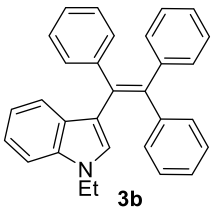

3b

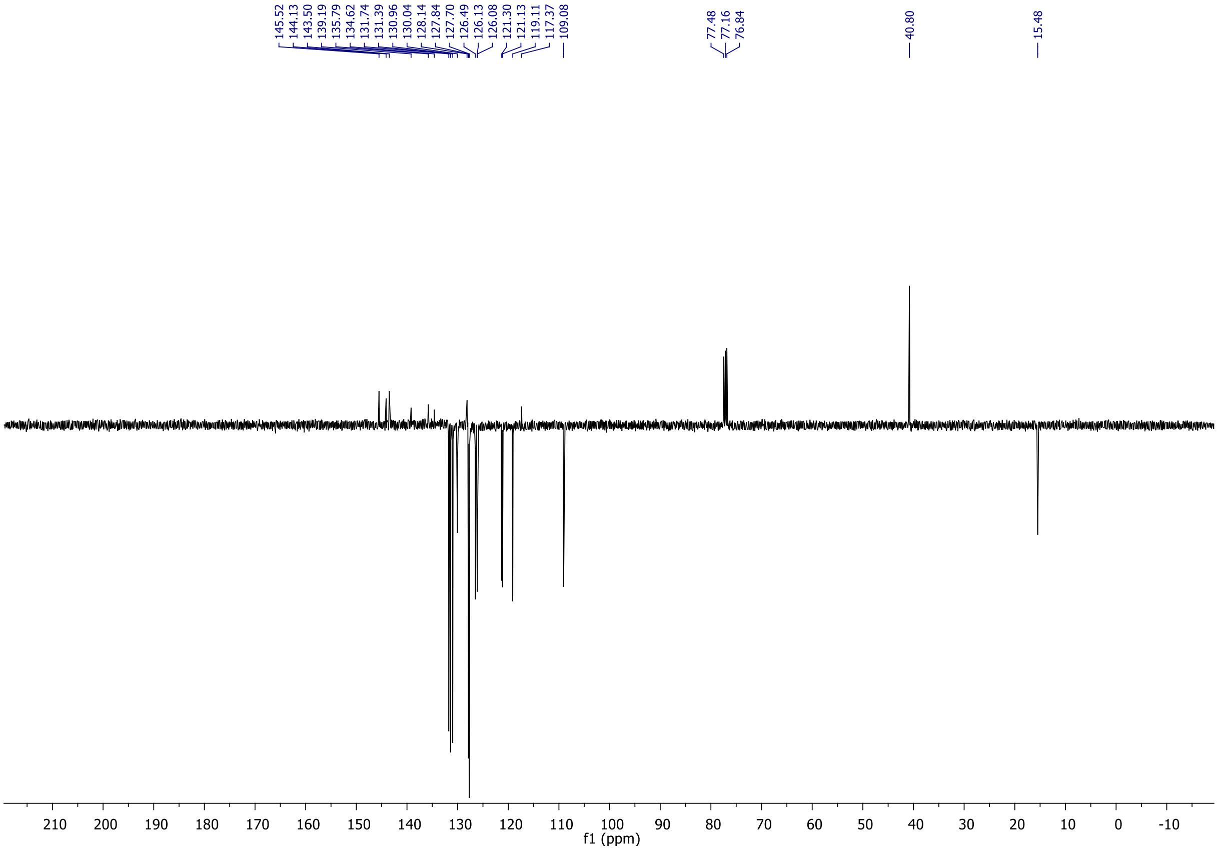

^{1}H NMR (400 MHz) and ^{13}C{^{1}H} (APT) NMR (100 MHz) spectra of **3b** ($CDCl_3$)

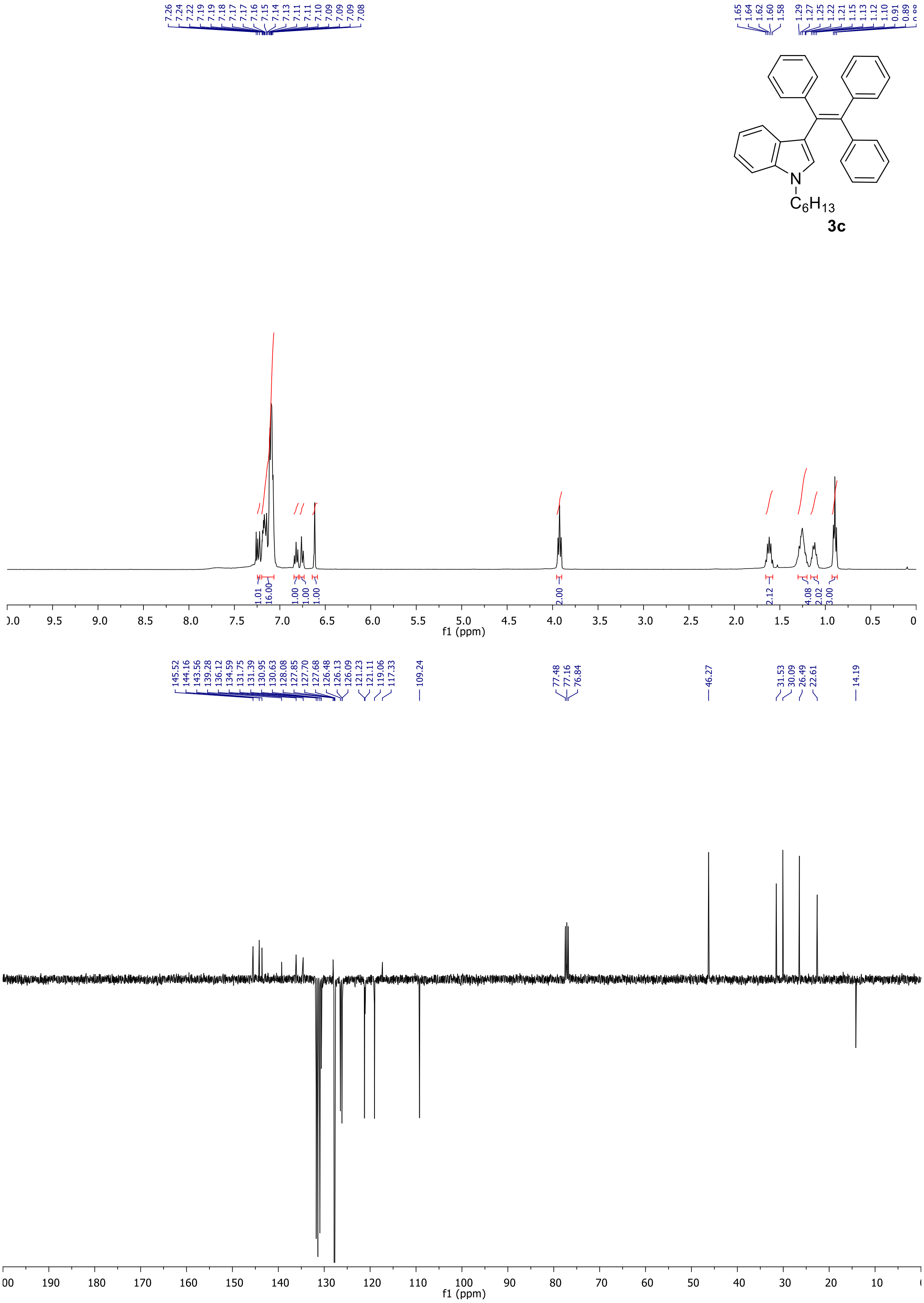

^{1}H NMR (400 MHz) and $^{13}C\{^{1}H\}$ (APT) NMR (100 MHz) spectra of **3c** ($CDCl_3$)

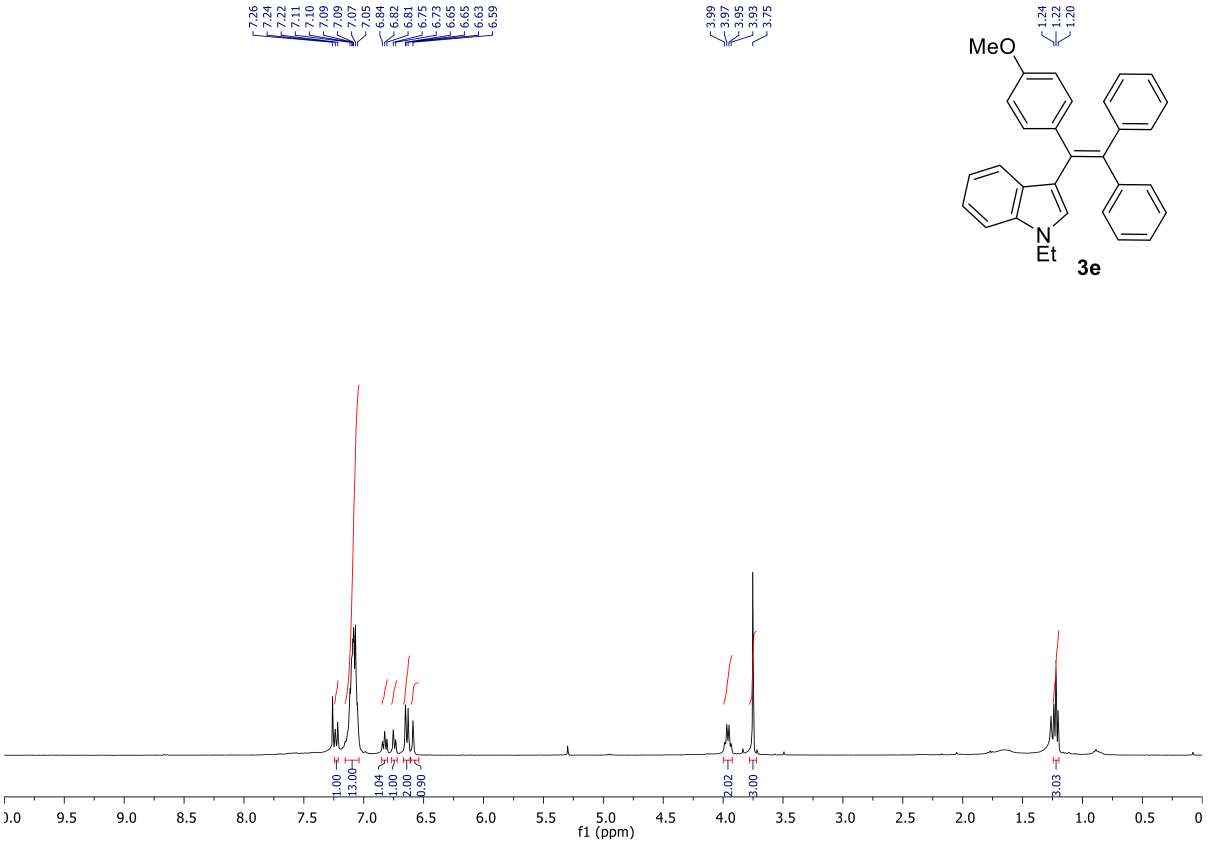

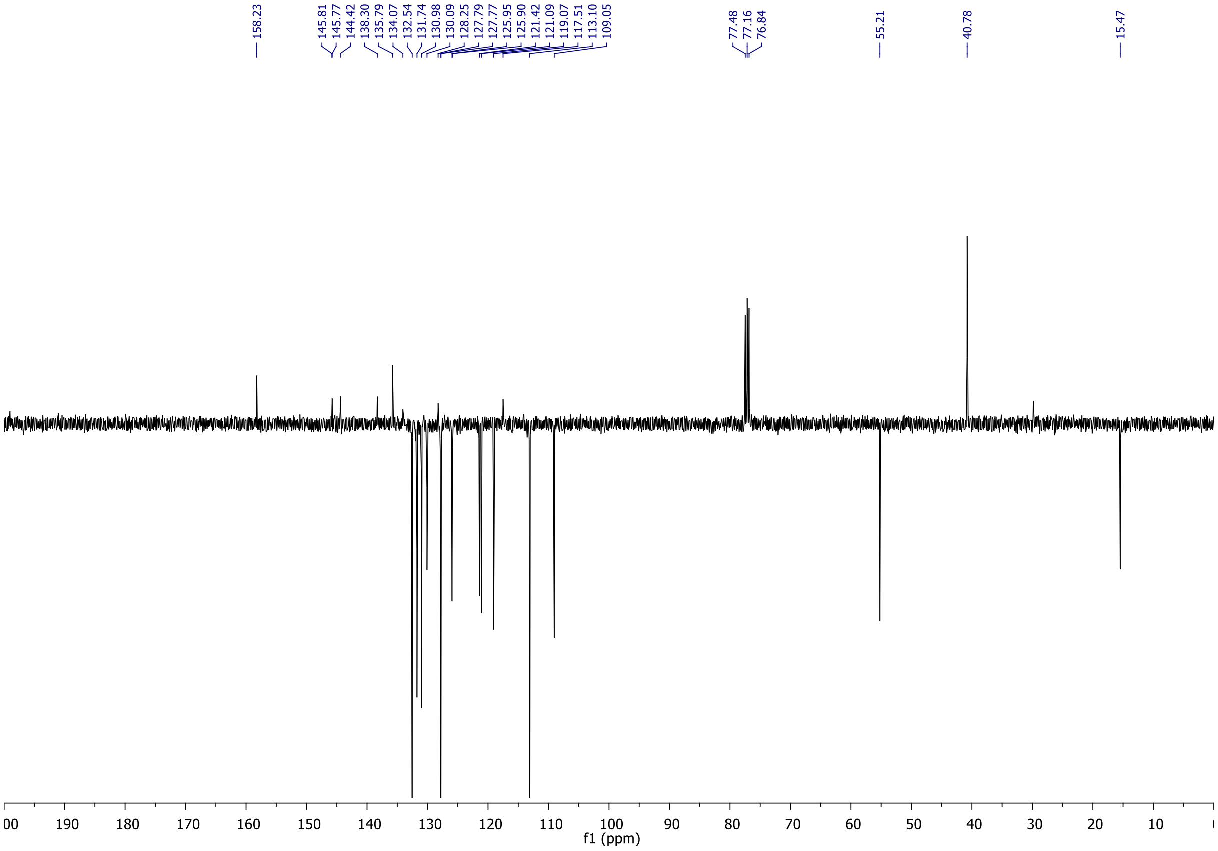

^{1}H NMR (400 MHz) and ^{13}C{^{1}H} (APT) NMR (100 MHz) spectra of **3e** ($CDCl_3$)

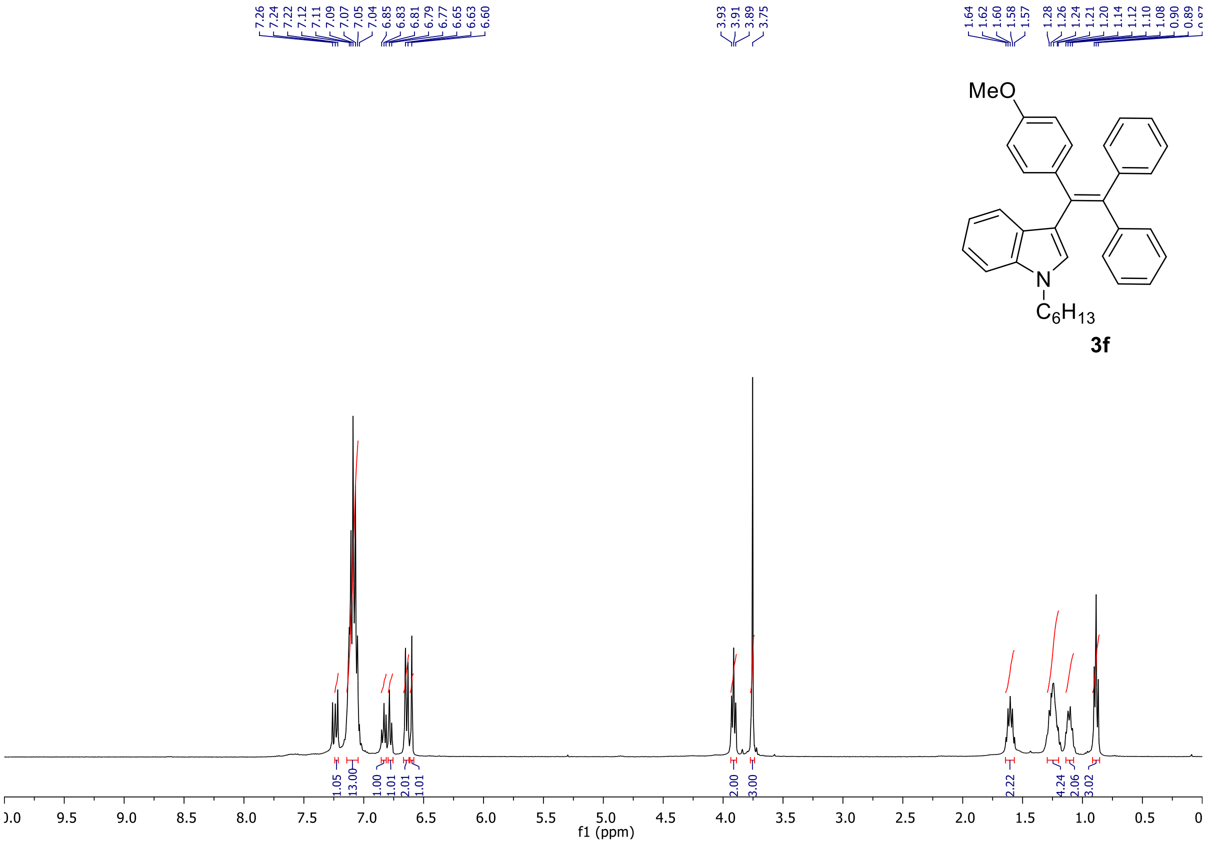

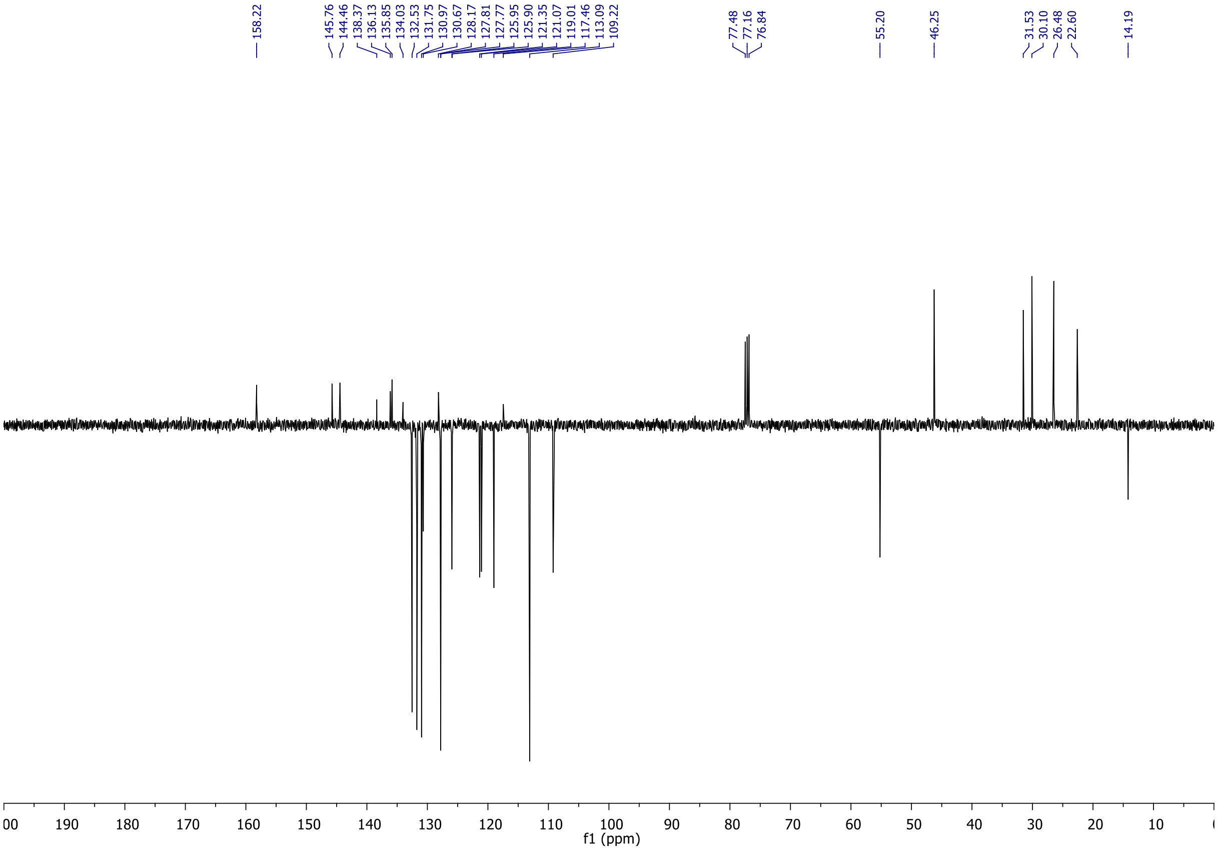

^{1}H NMR (400 MHz) and ^{13}C{^{1}H} (APT) NMR (100 MHz) spectra of **3f** ($CDCl_3$)

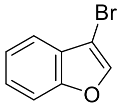

S68

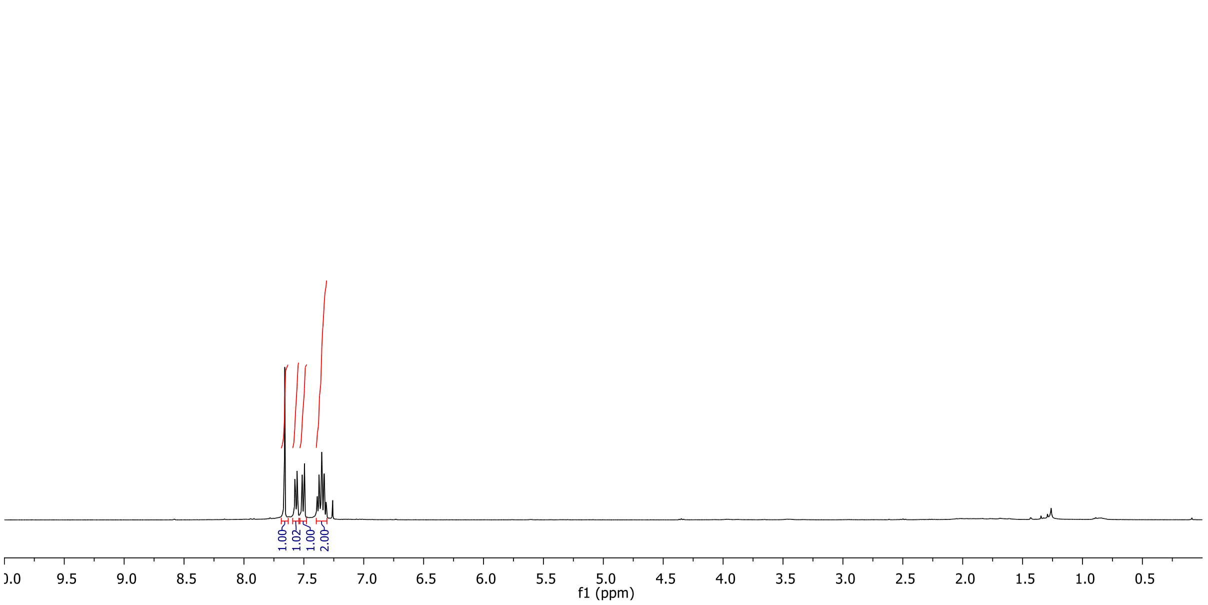

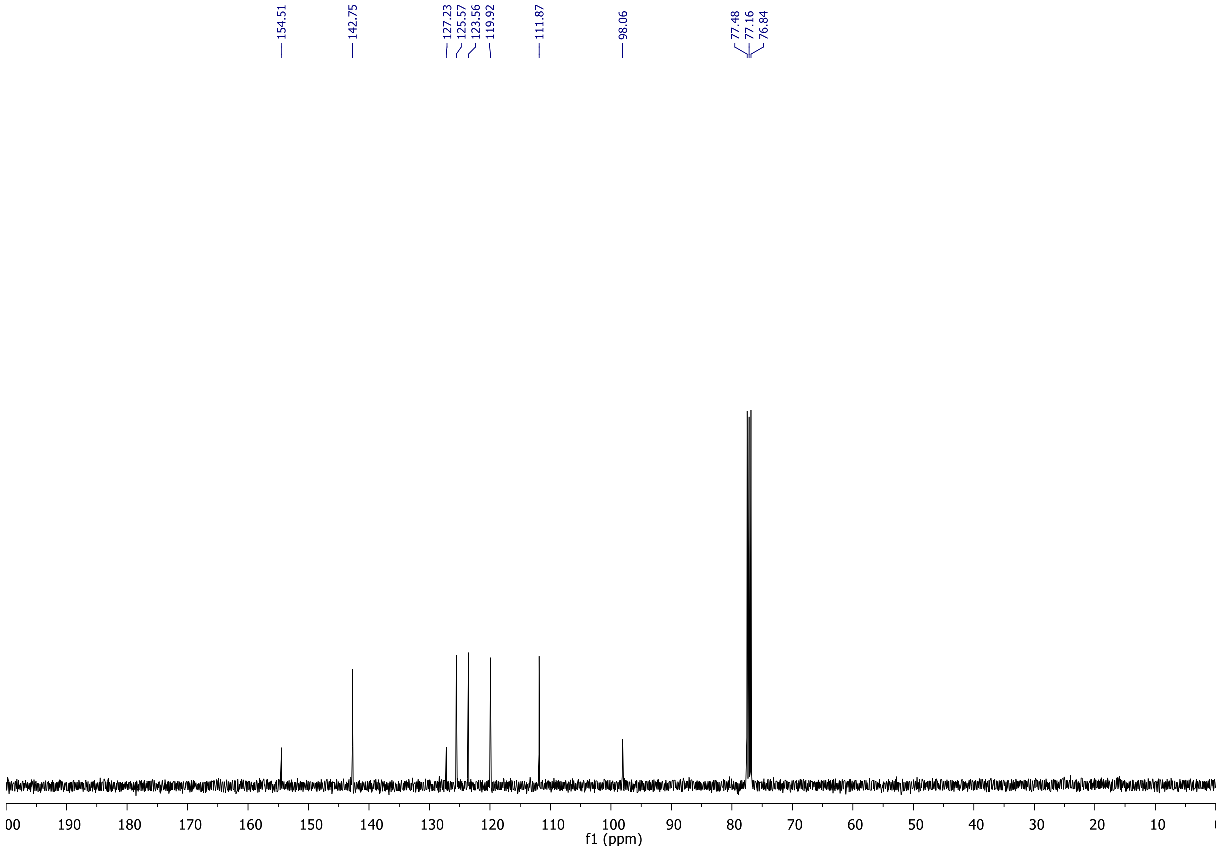

^1H NMR (400 MHz) and $^{13}C\{^1H\}$ NMR (100 MHz) spectra of **S68** ($CDCl_3$)

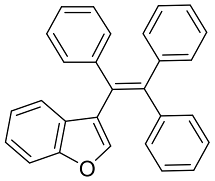

3n

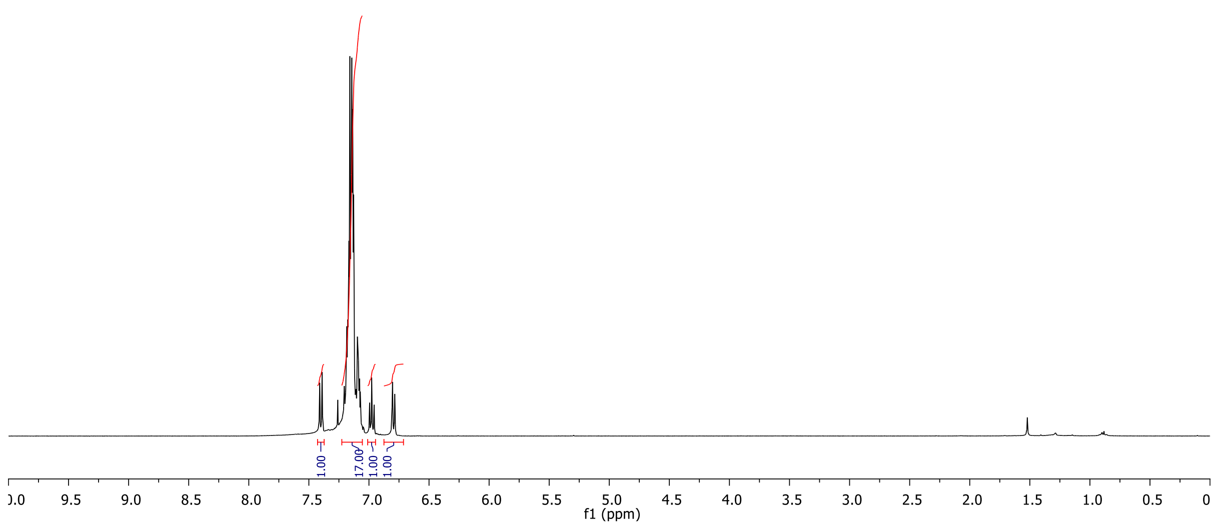

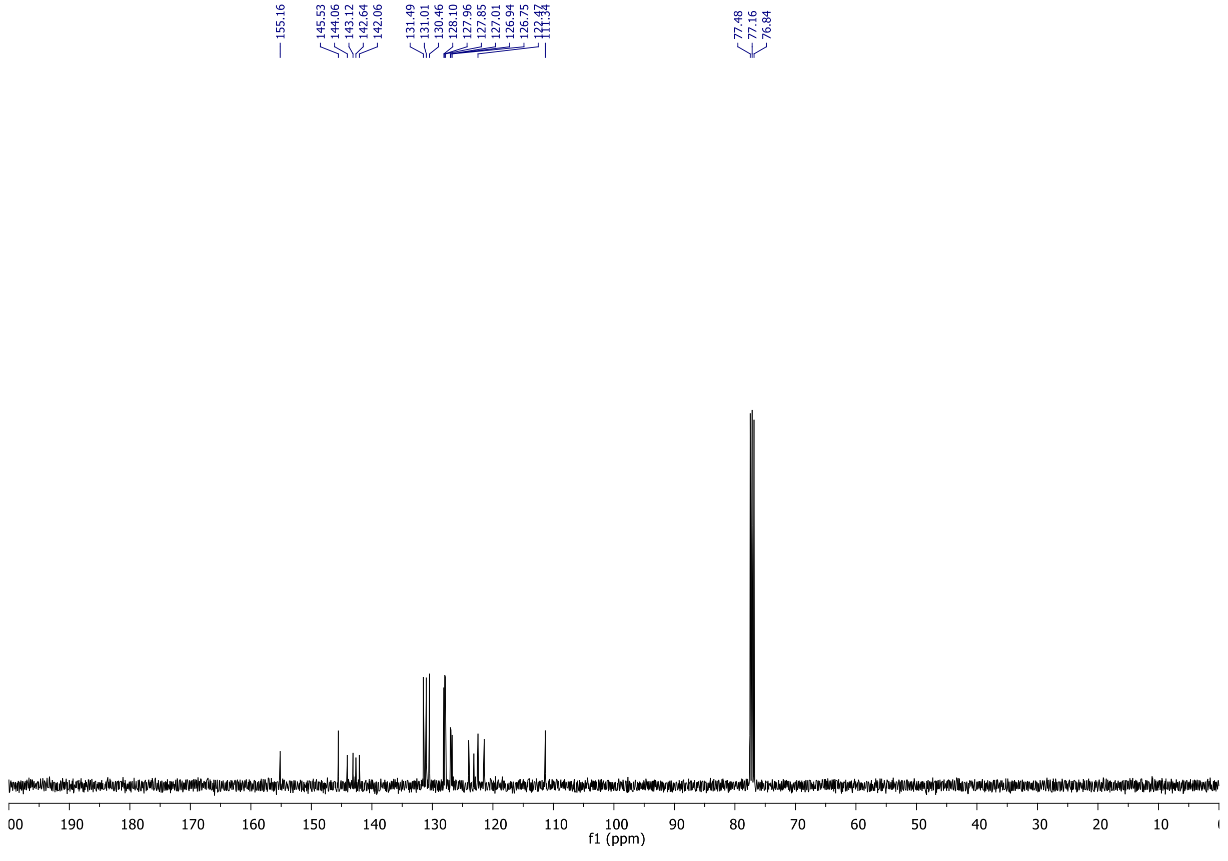

^{1}H NMR (400 MHz) and ^{13}C{^{1}H} NMR (100 MHz) spectra of **3n** ($CDCl_3$)

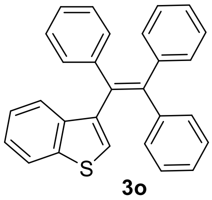

3o

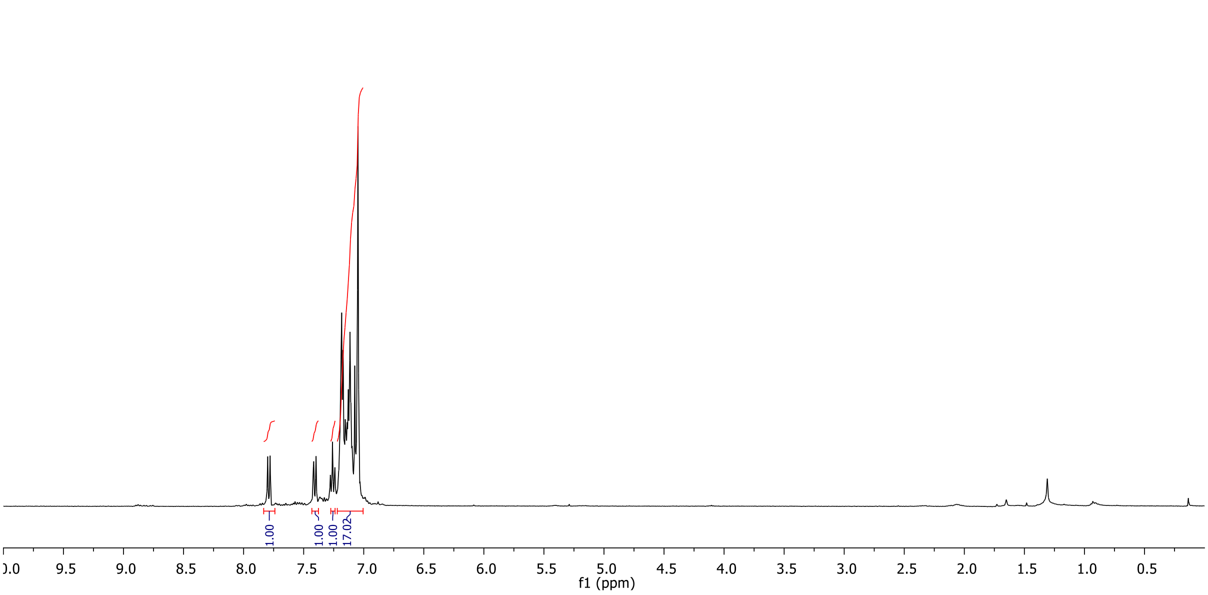

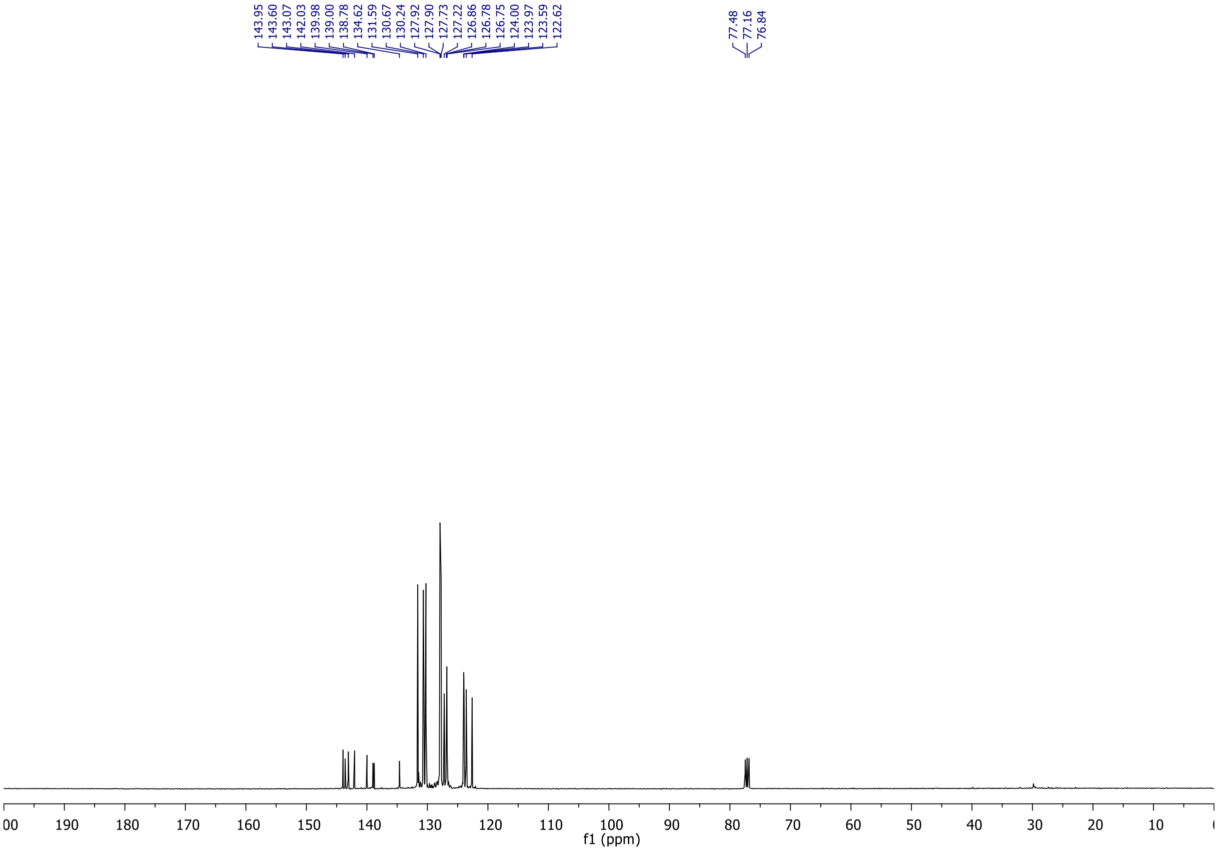

^{1}H NMR (400 MHz) and ^{13}C{^{1}H} NMR (100 MHz) spectra of **3o** ($CDCl_3$)

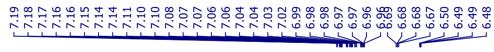

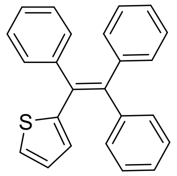

3p

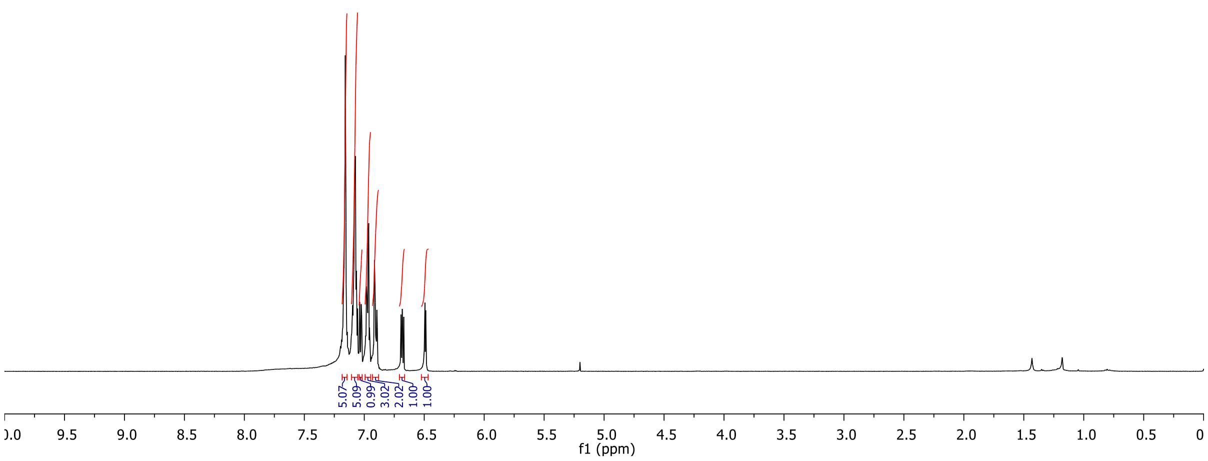

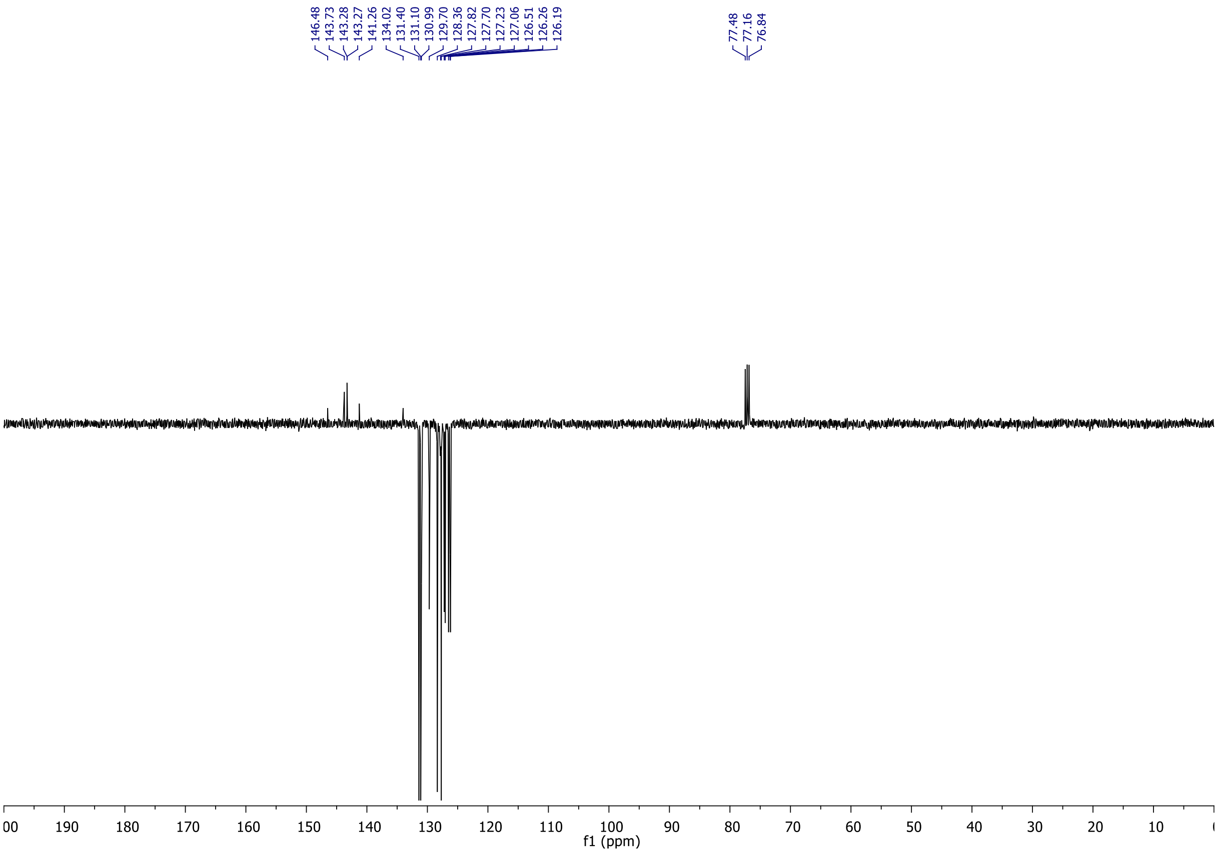

^{1}H NMR (400 MHz) and ^{13}C{^{1}H} NMR (100 MHz) spectra of **3p** ($CDCl_3$)

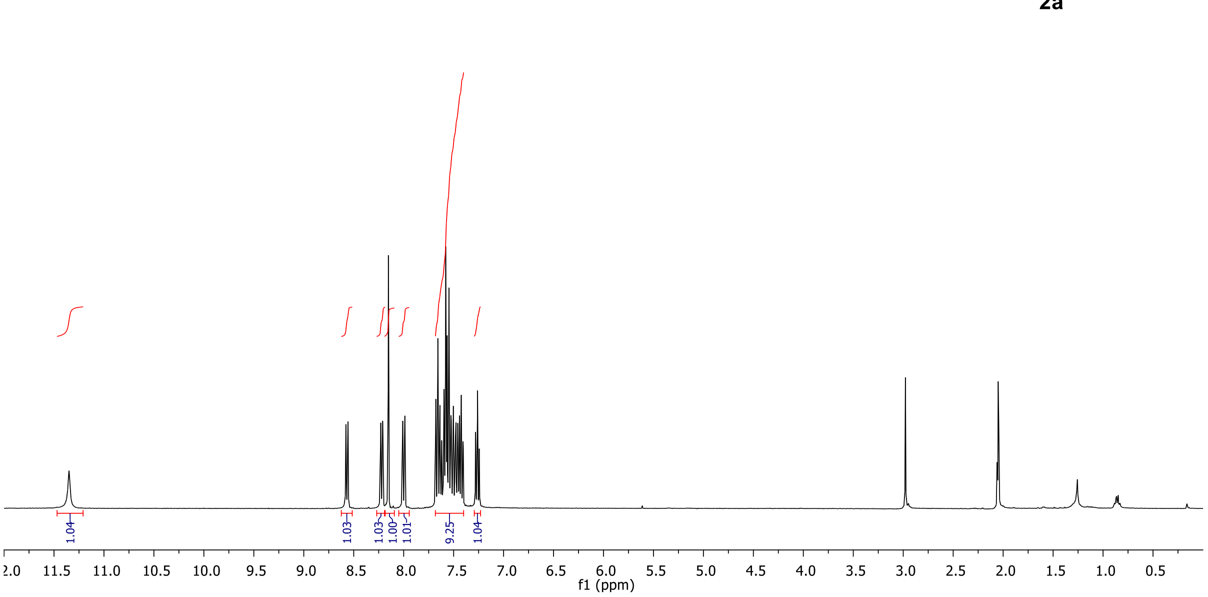

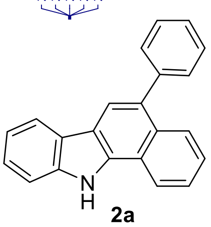

2a

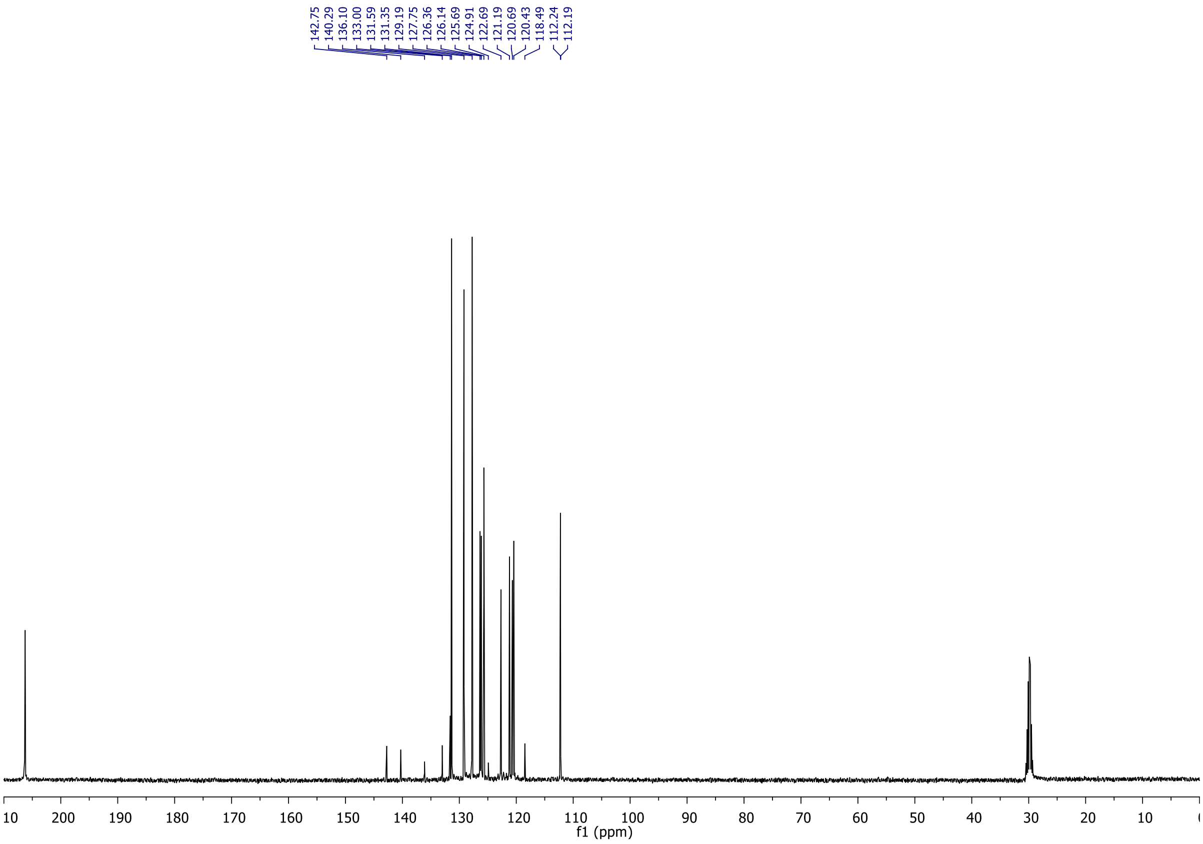

^{1}H NMR (400 MHz) and ^{13}C{^{1}H} NMR (100 MHz) spectra of **2a** (Acetone-d_6)

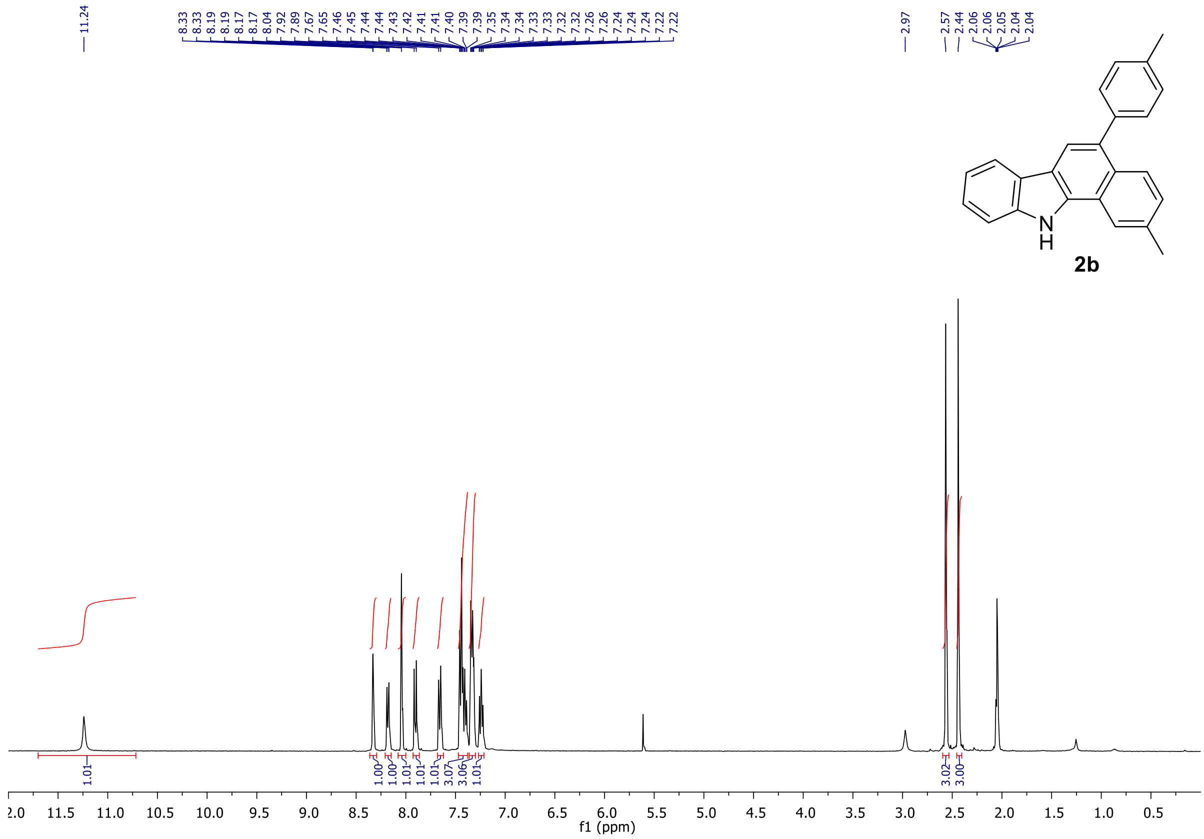

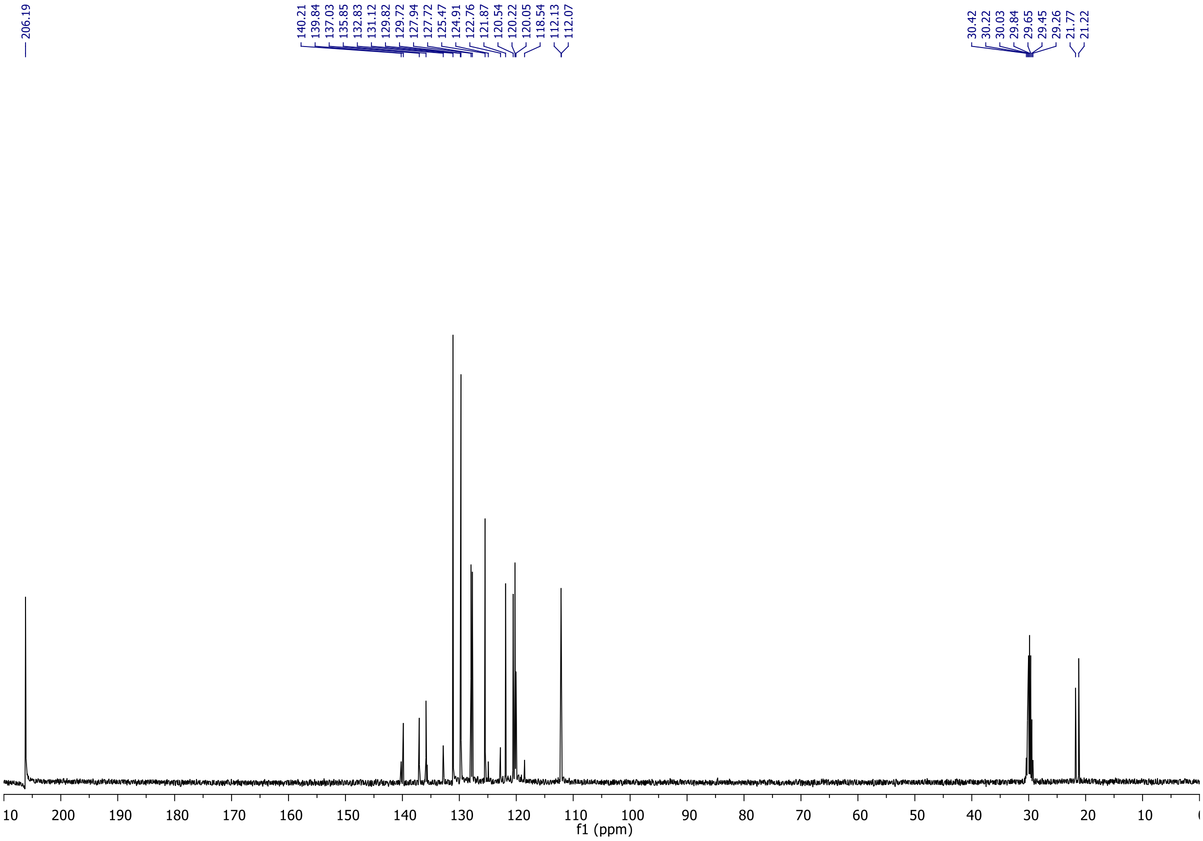

^{1}H NMR (400 MHz) and ^{13}C{^{1}H} NMR (100 MHz) spectra of **2b** (Acetone-d_6)

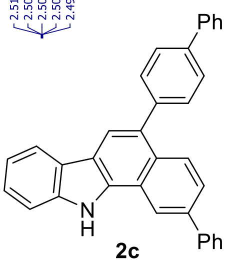

2c

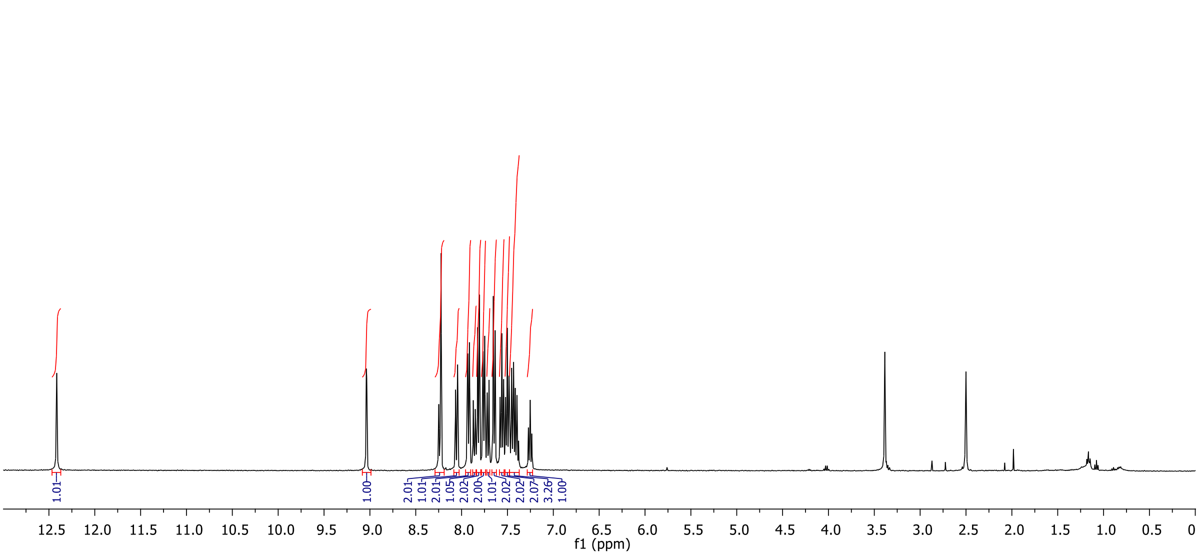

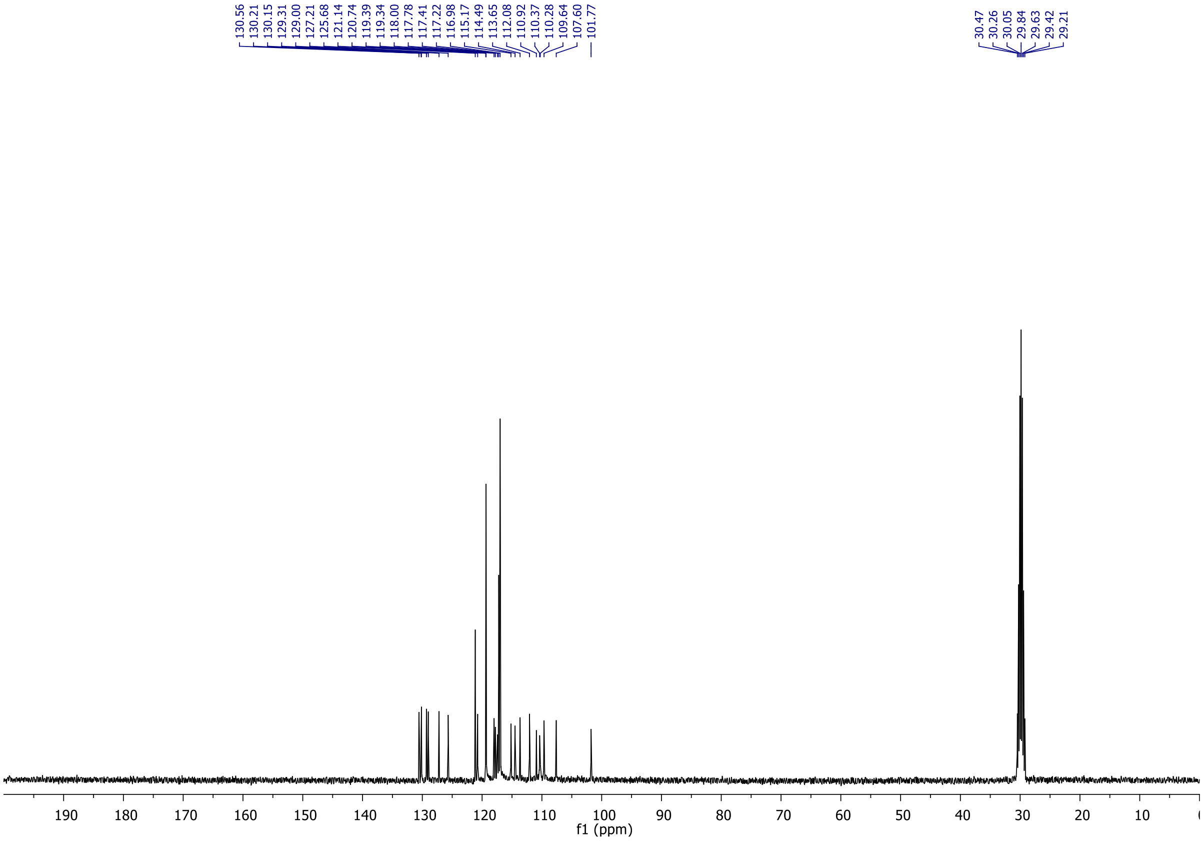

^{1}H NMR (400 MHz) and $^{13}C\{^{1}H\}$ NMR (100 MHz) spectra of **2c** (DMSO-d_6)

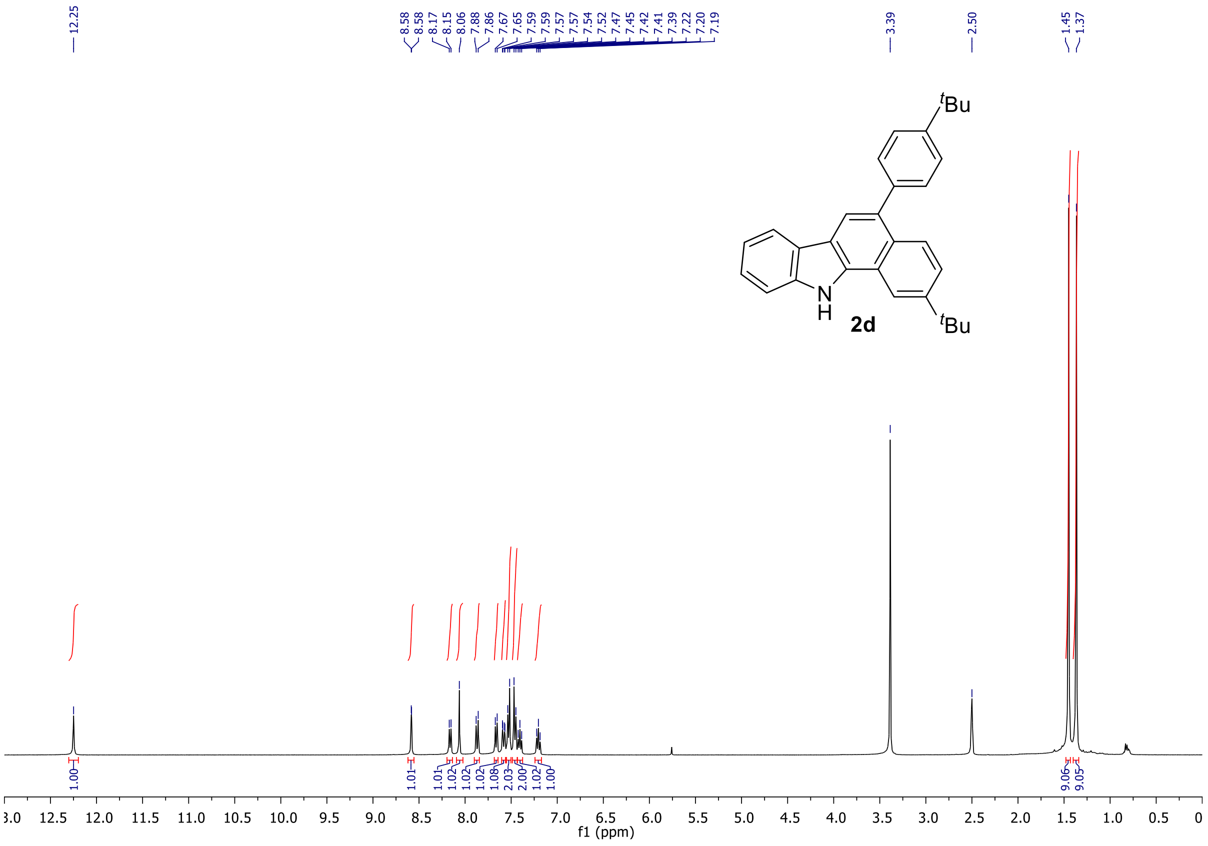

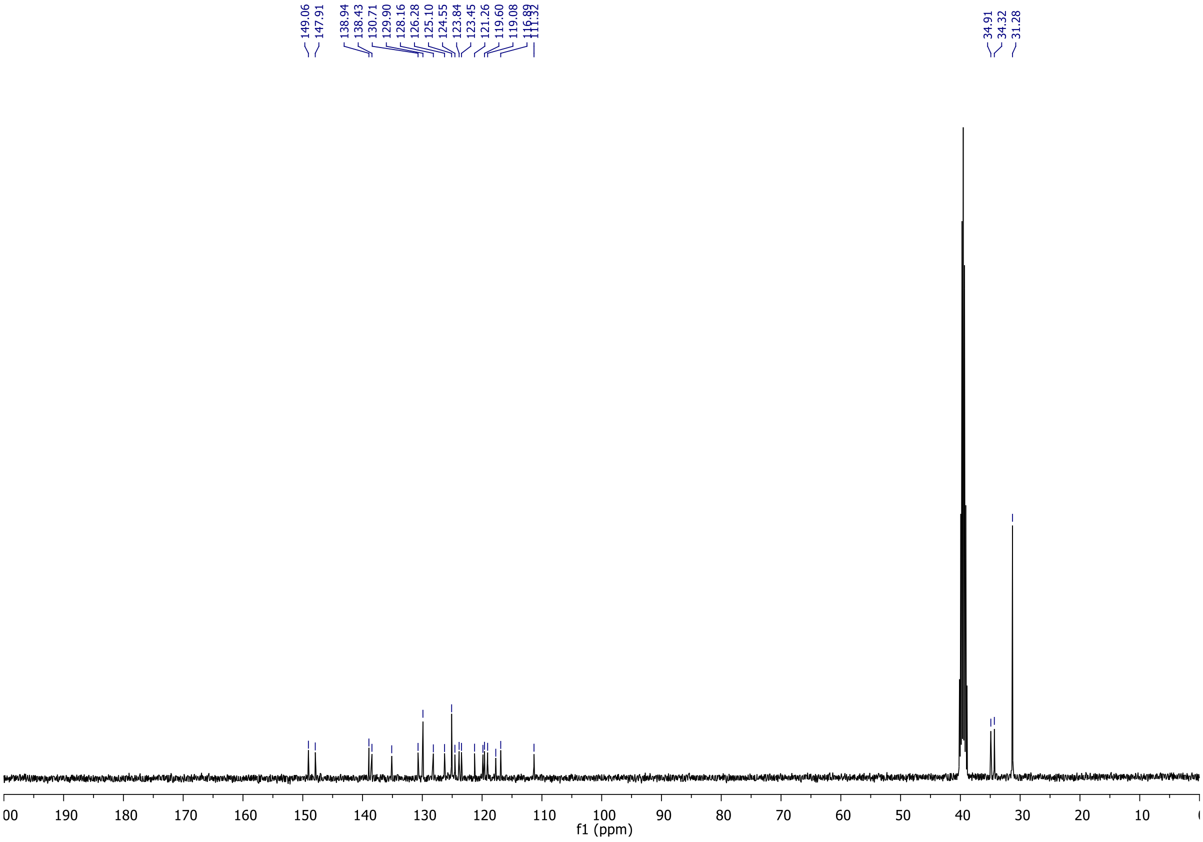

^{1}H NMR (400 MHz) and ^{13}C{^{1}H} NMR (100 MHz) spectra of **2d** (DMSO-d_6)

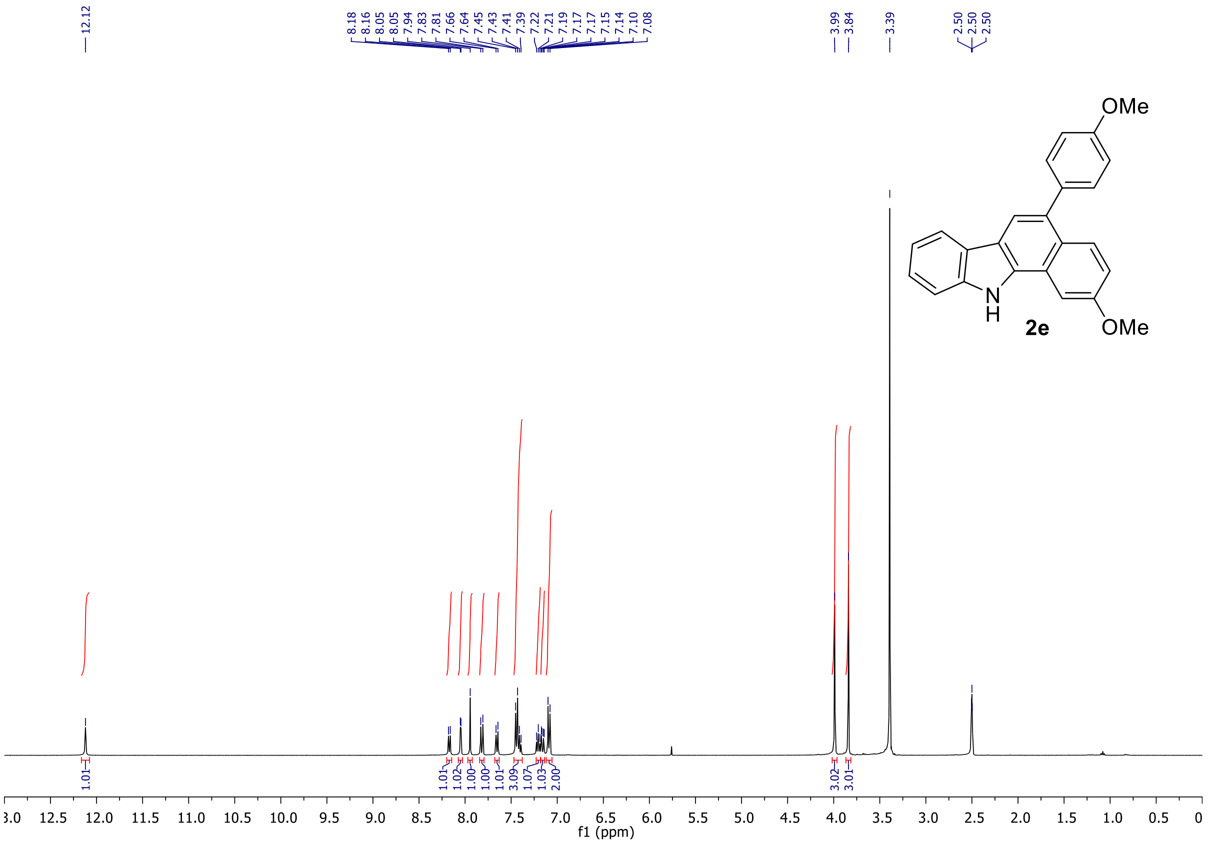

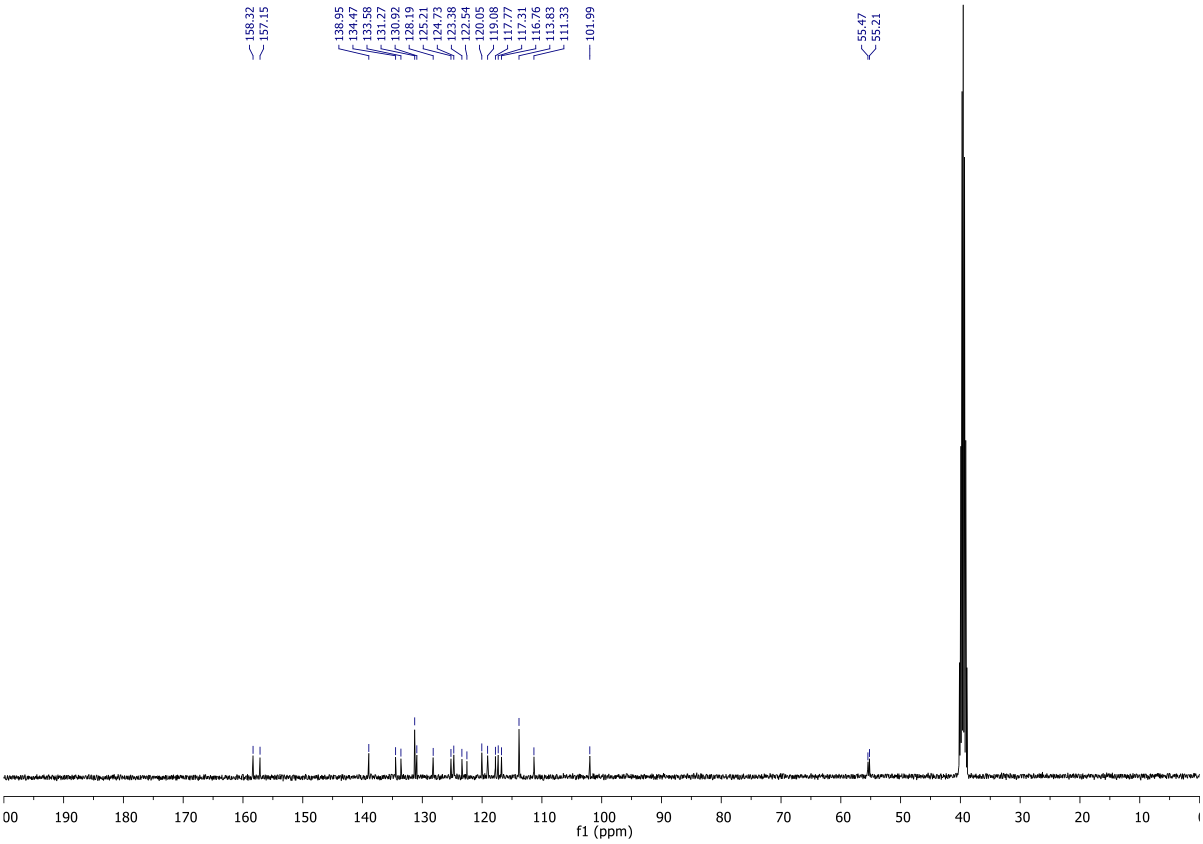

^{1}H NMR (400 MHz) and ^{13}C{^{1}H} NMR (100 MHz) spectra of **2e** (DMSO-d_6)

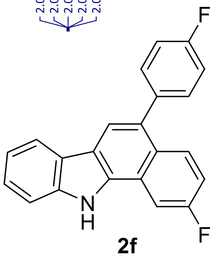

2f

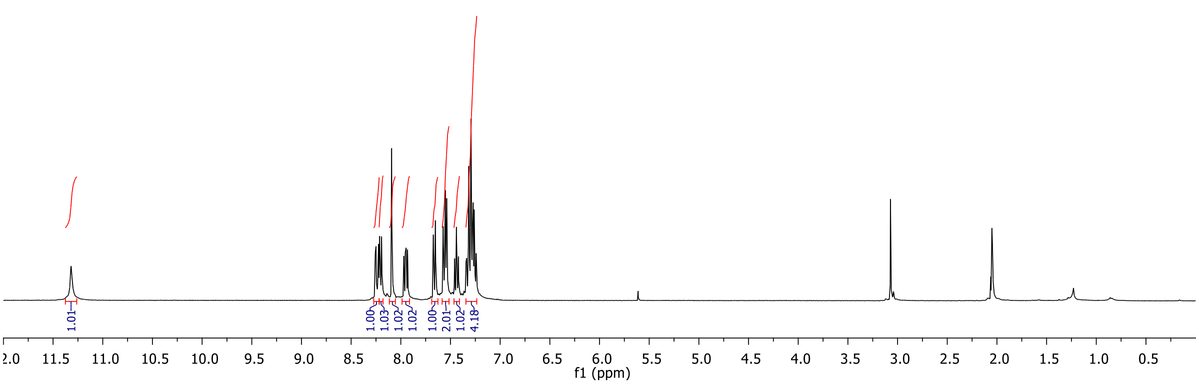

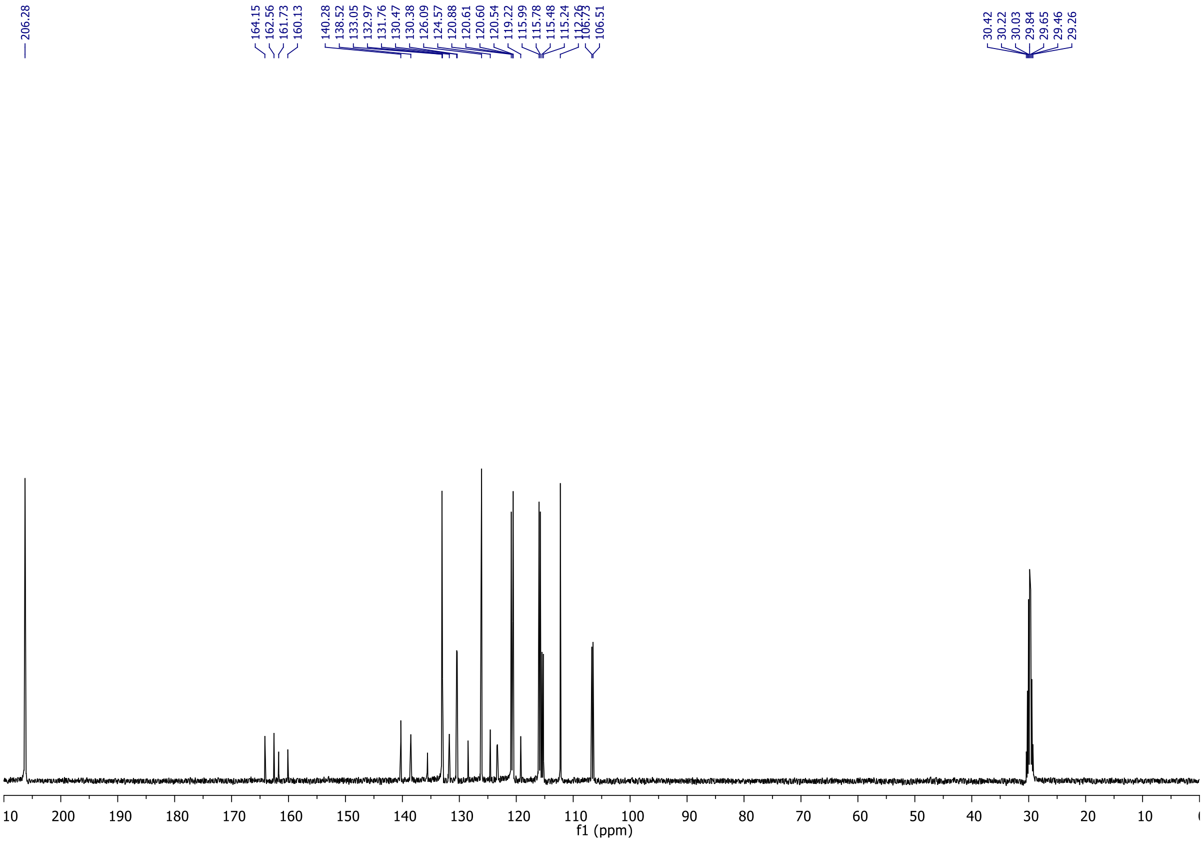

^{1}H NMR (400 MHz) and ^{13}C{^{1}H} NMR (100 MHz) spectra of **2f** (Acetone-d_6)

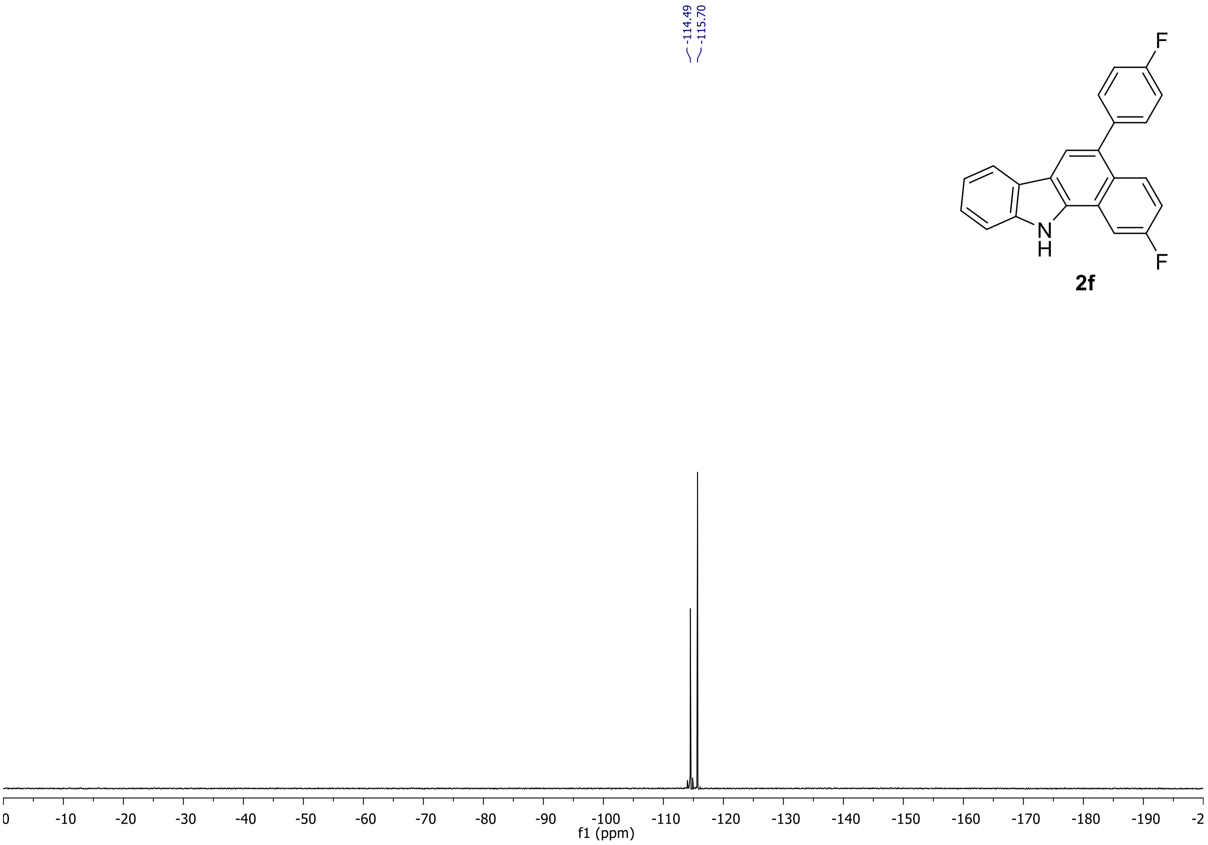

^{19}F NMR (376 MHz) spectra of **2f** ($CDCl_3$)

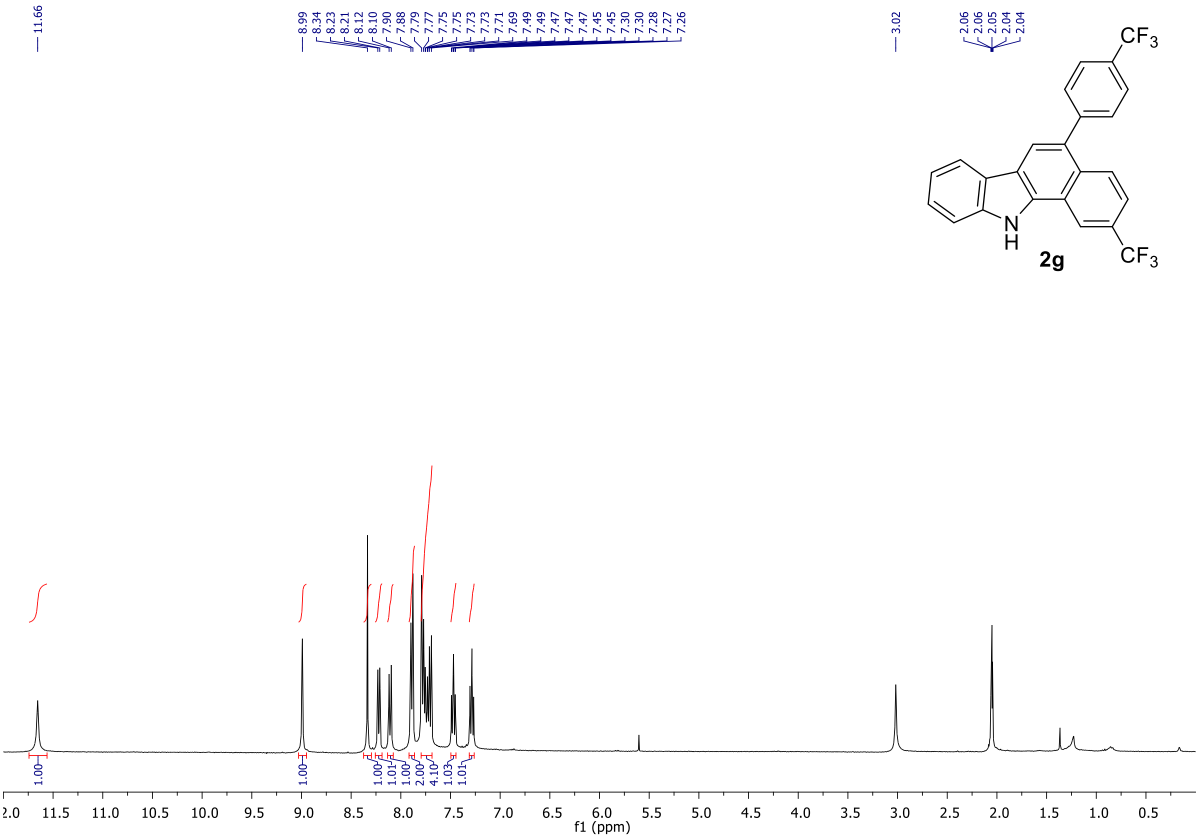

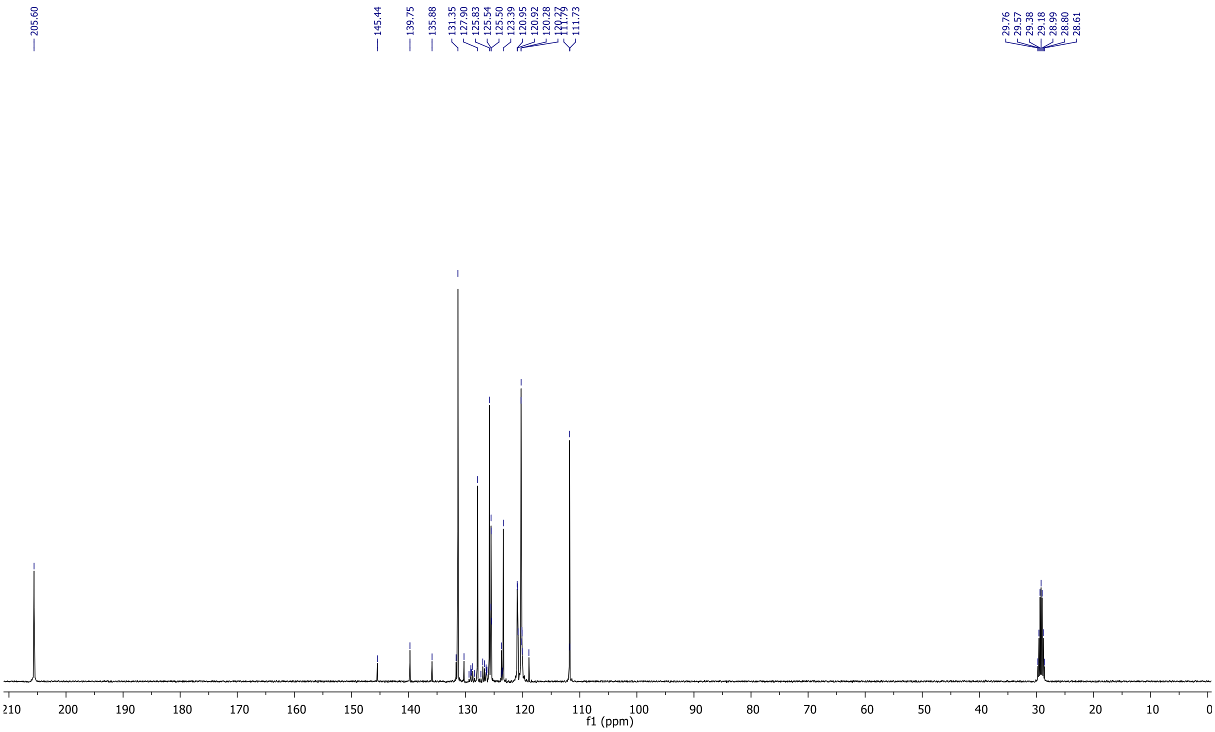

^{1}H NMR (400 MHz) and $^{13}C\{^{1}H\}$ NMR (100 MHz) spectra of **2g** (Acetone-d_6)

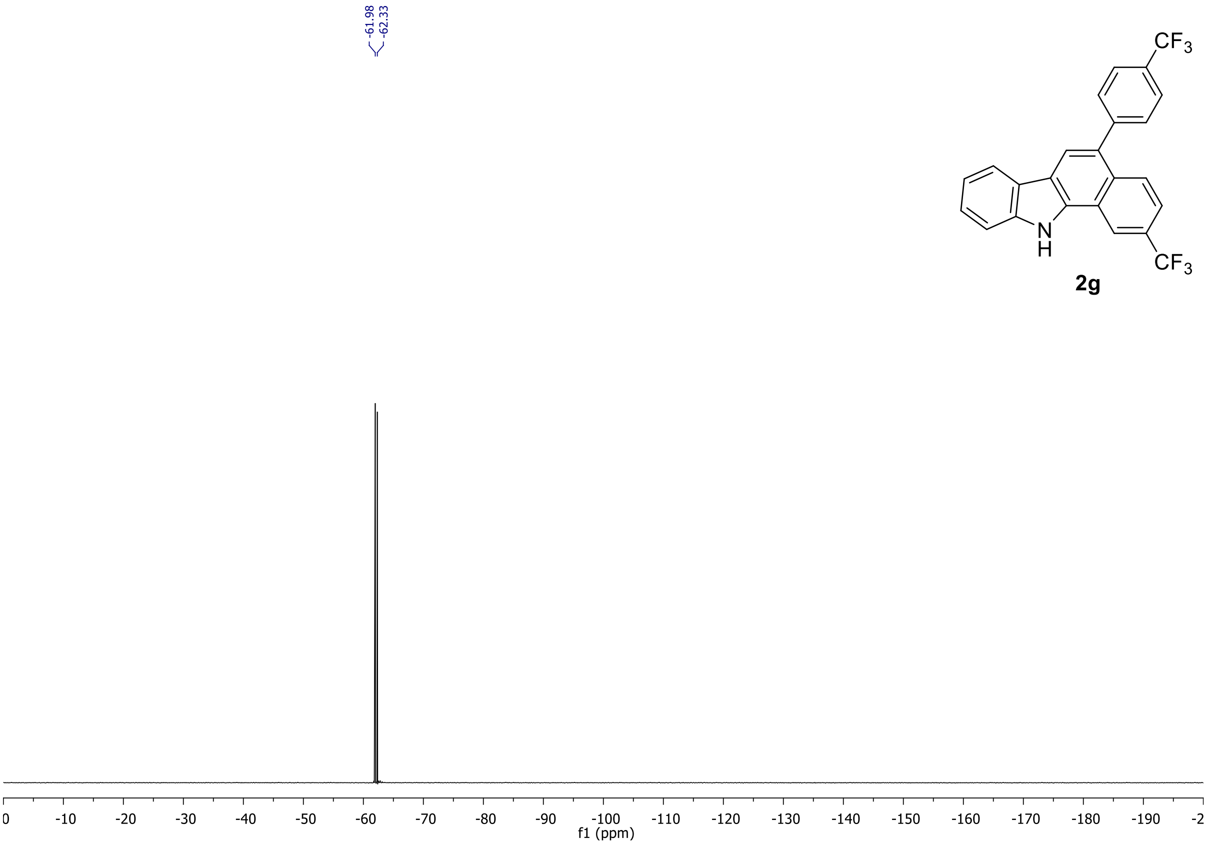

^{19}F NMR (376 MHz) spectra of **2g** ($CDCl_3$)

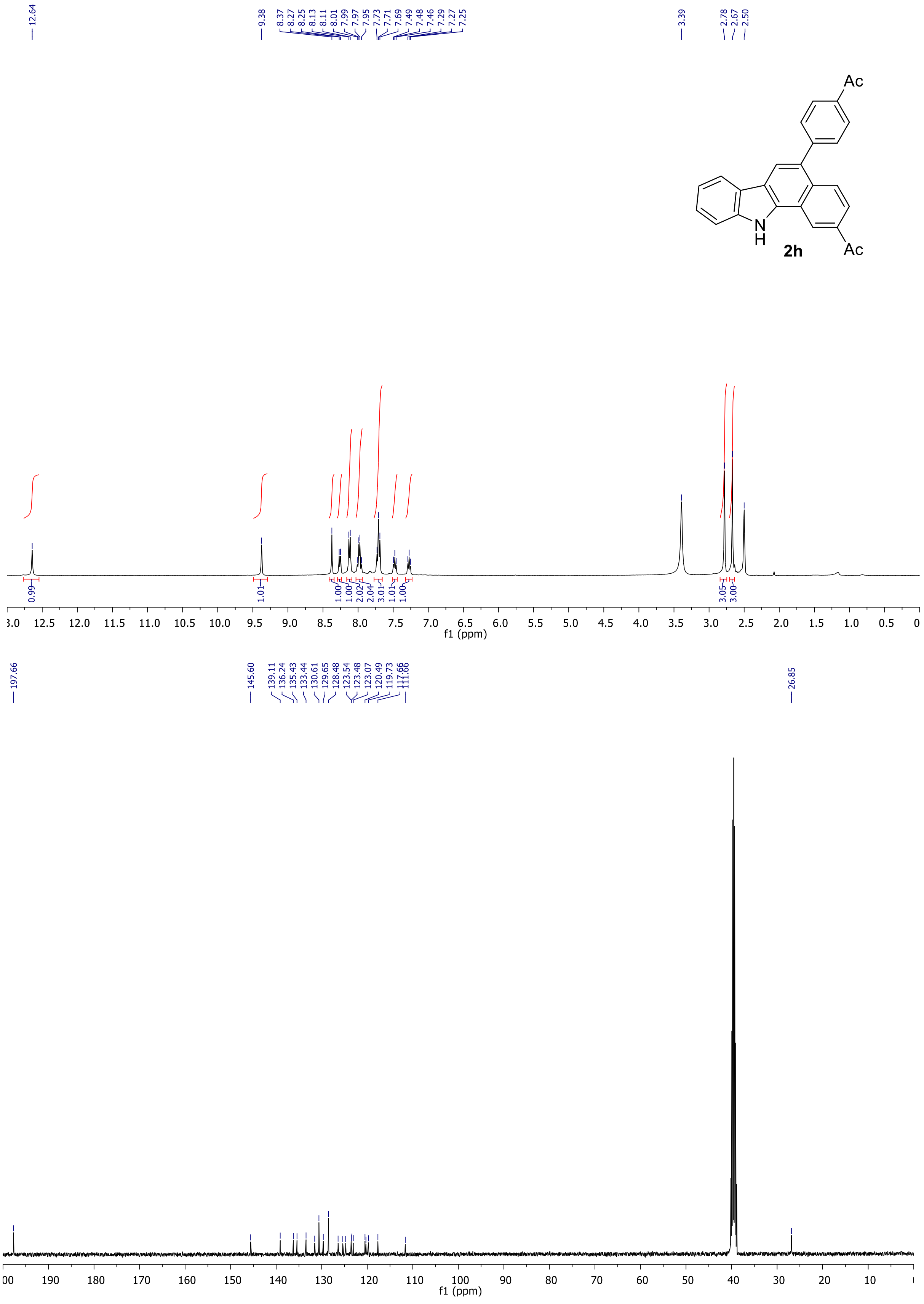

^{1}H NMR (400 MHz) and ^{13}C{^{1}H} NMR (100 MHz) spectra of **2h** (DMSO-d_6)

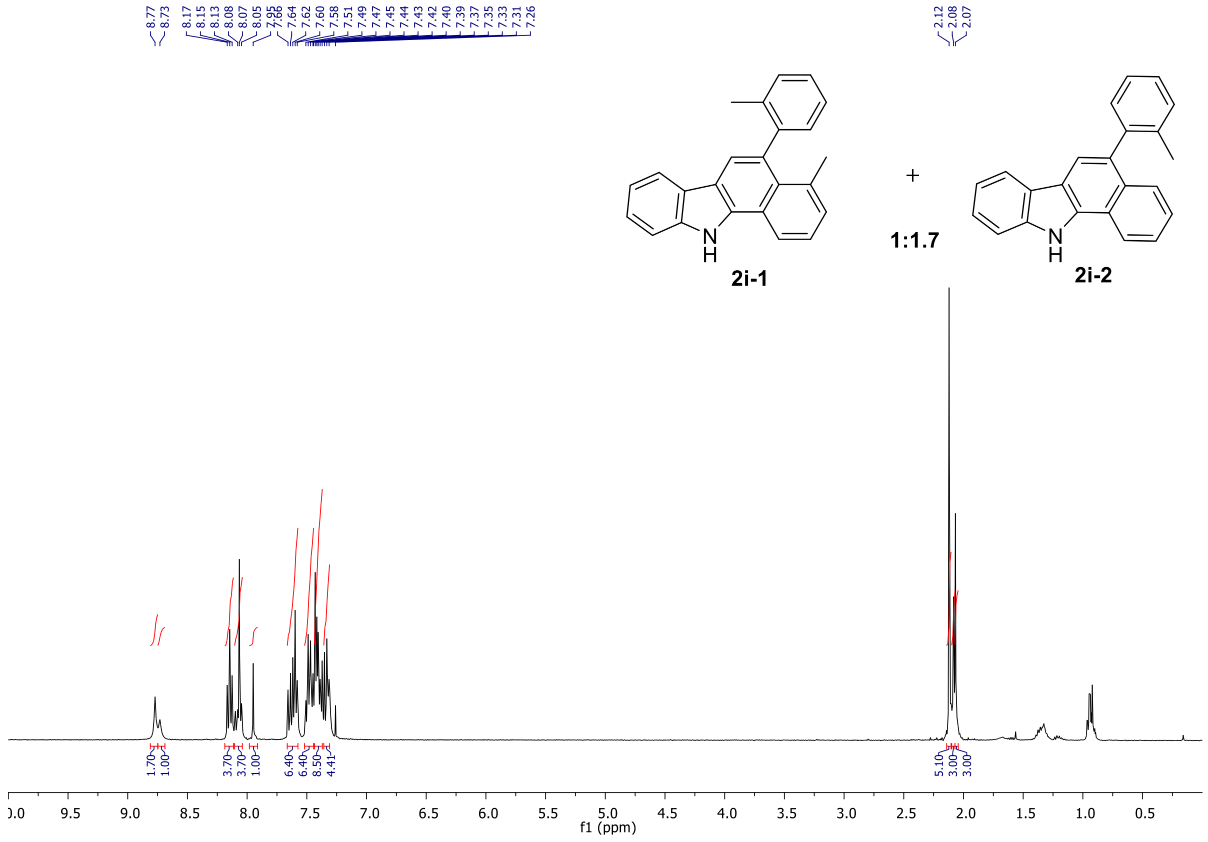

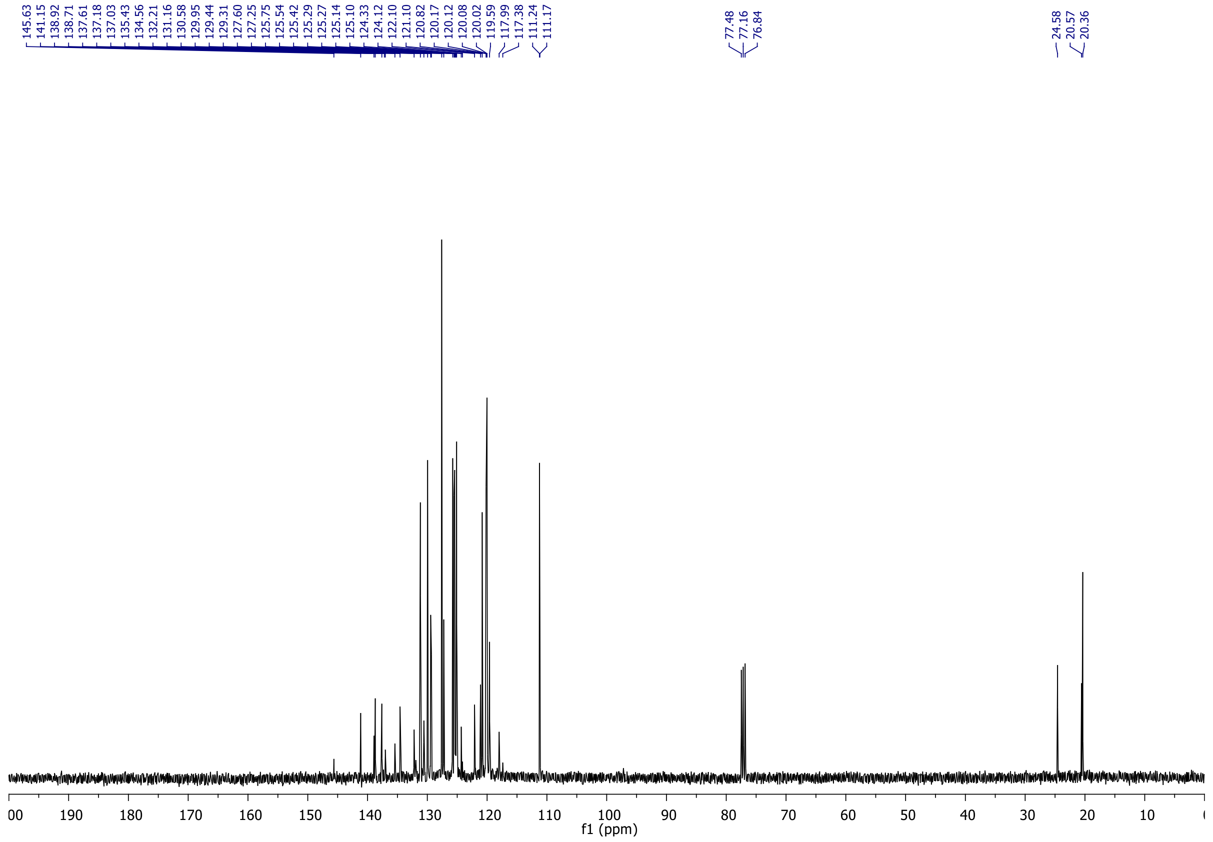

^{1}H NMR (400 MHz) and ^{13}C{^{1}H} NMR (100 MHz) spectra of **2i-1+2i-2** ($CDCl_3$)

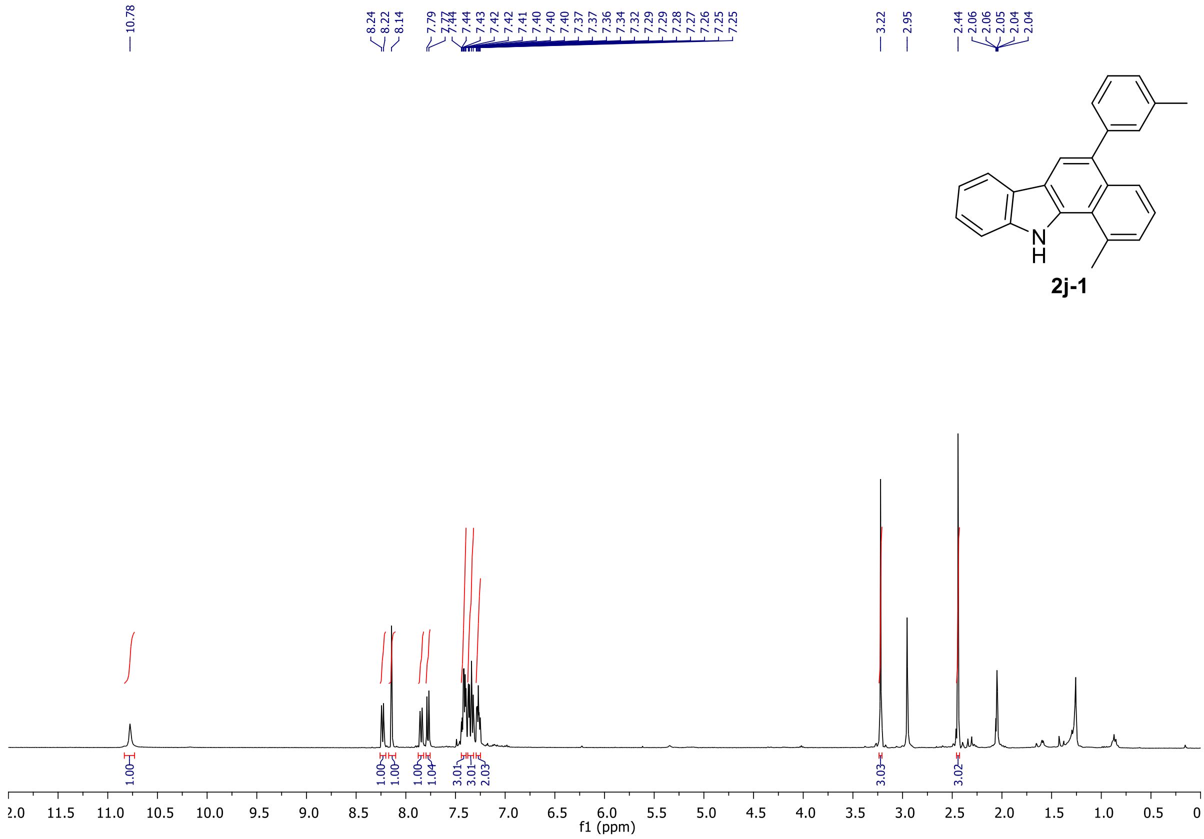

2j-1

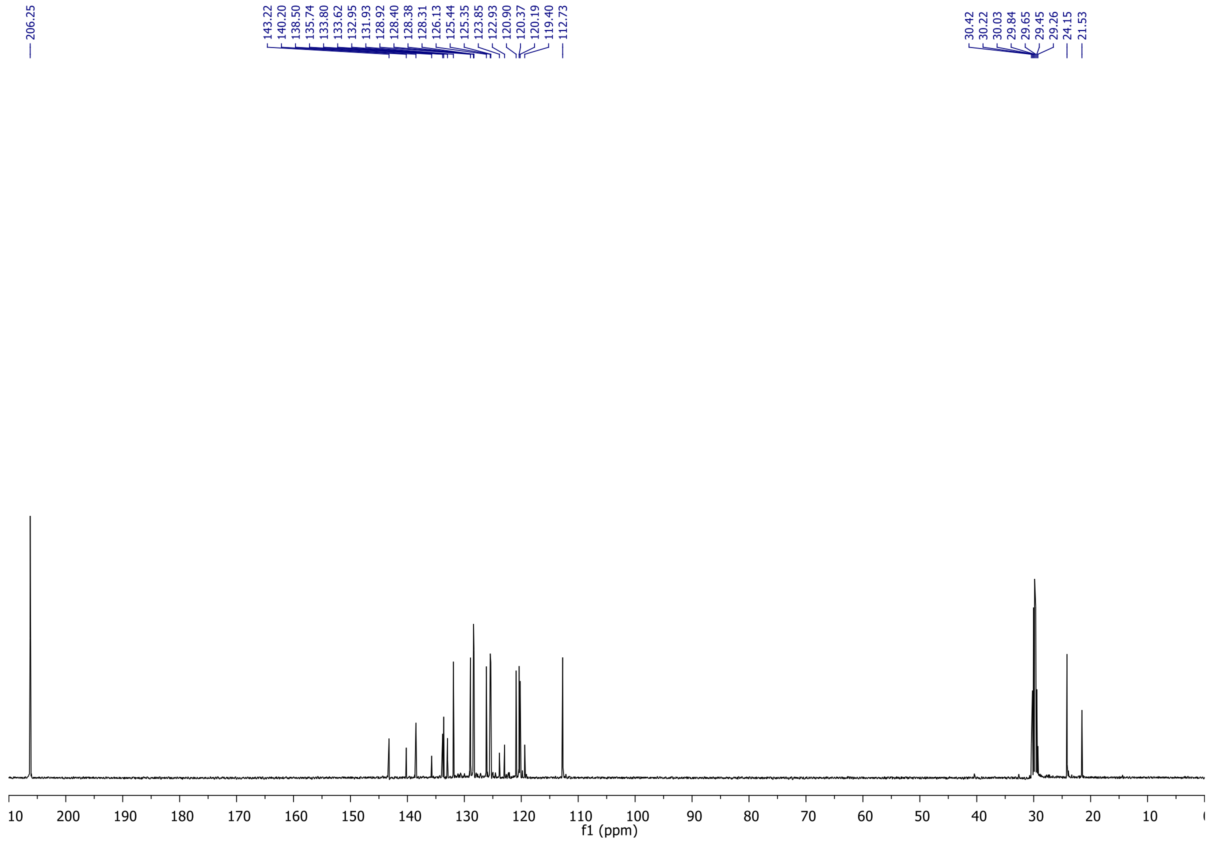

^{1}H NMR (400 MHz) and ^{13}C{^{1}H} NMR (100 MHz) spectra of **2j-1** (Acetone-d_6)

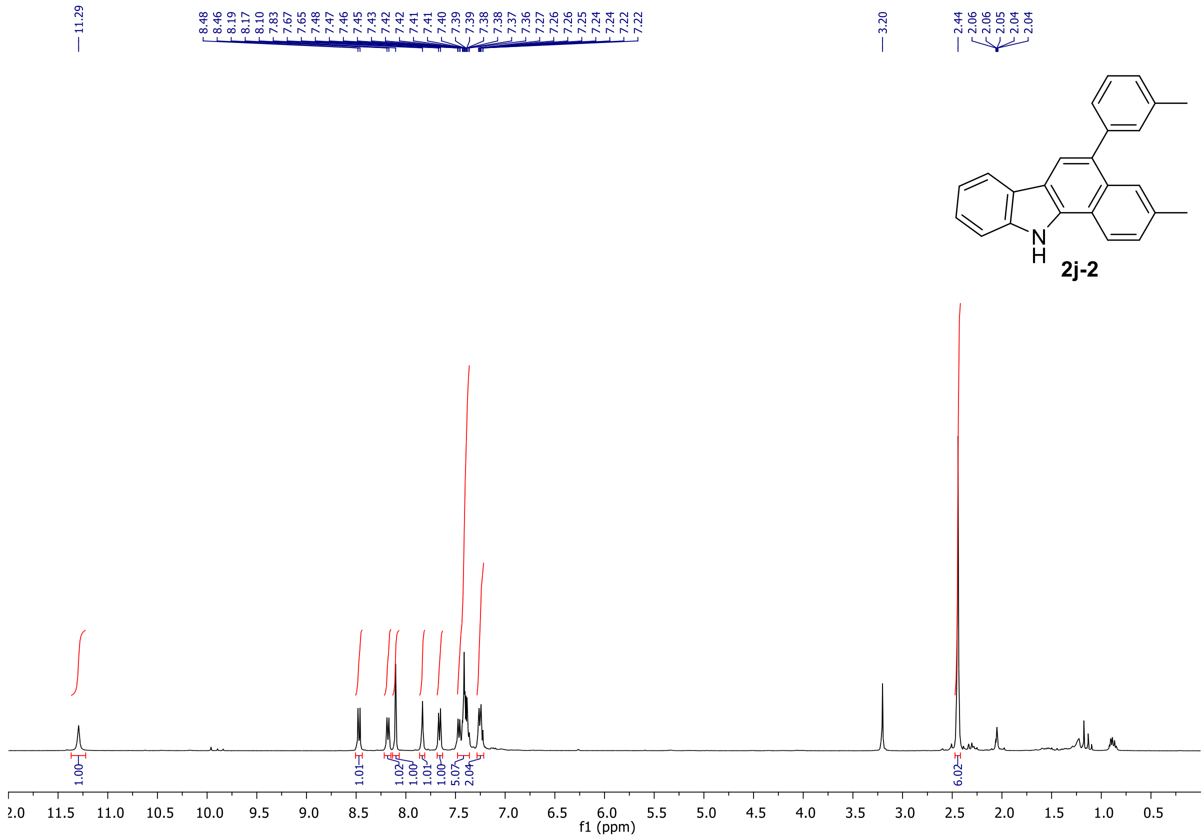

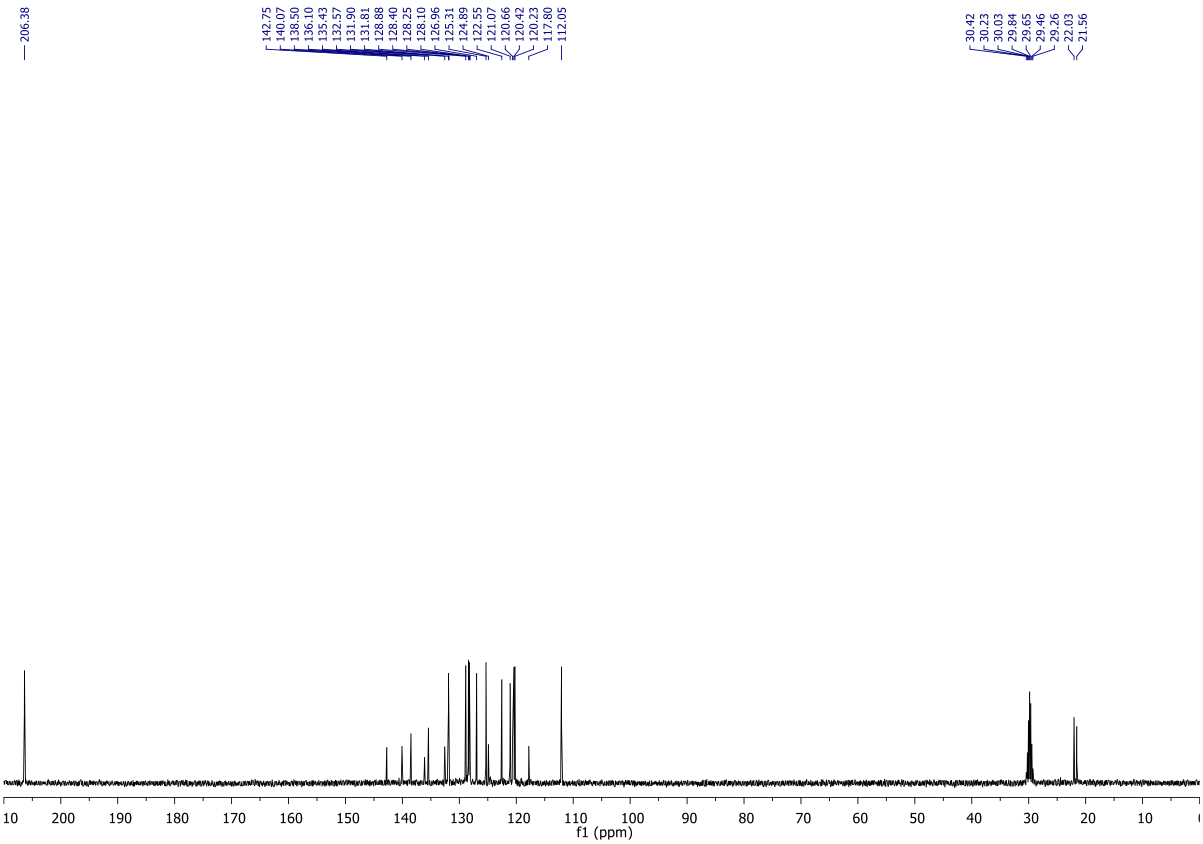

^{1}H NMR (400 MHz) and ^{13}C{^{1}H} NMR (100 MHz) spectra of **2j-2** (Acetone-d_6)

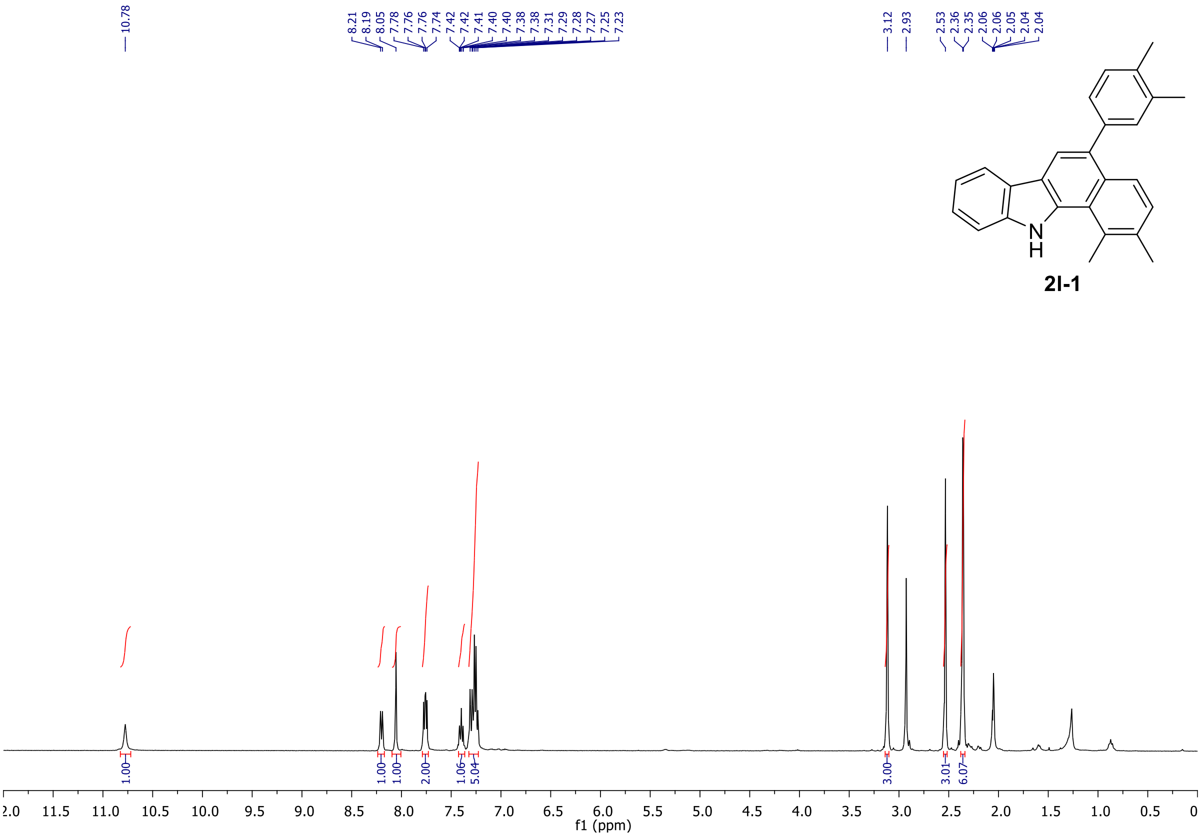

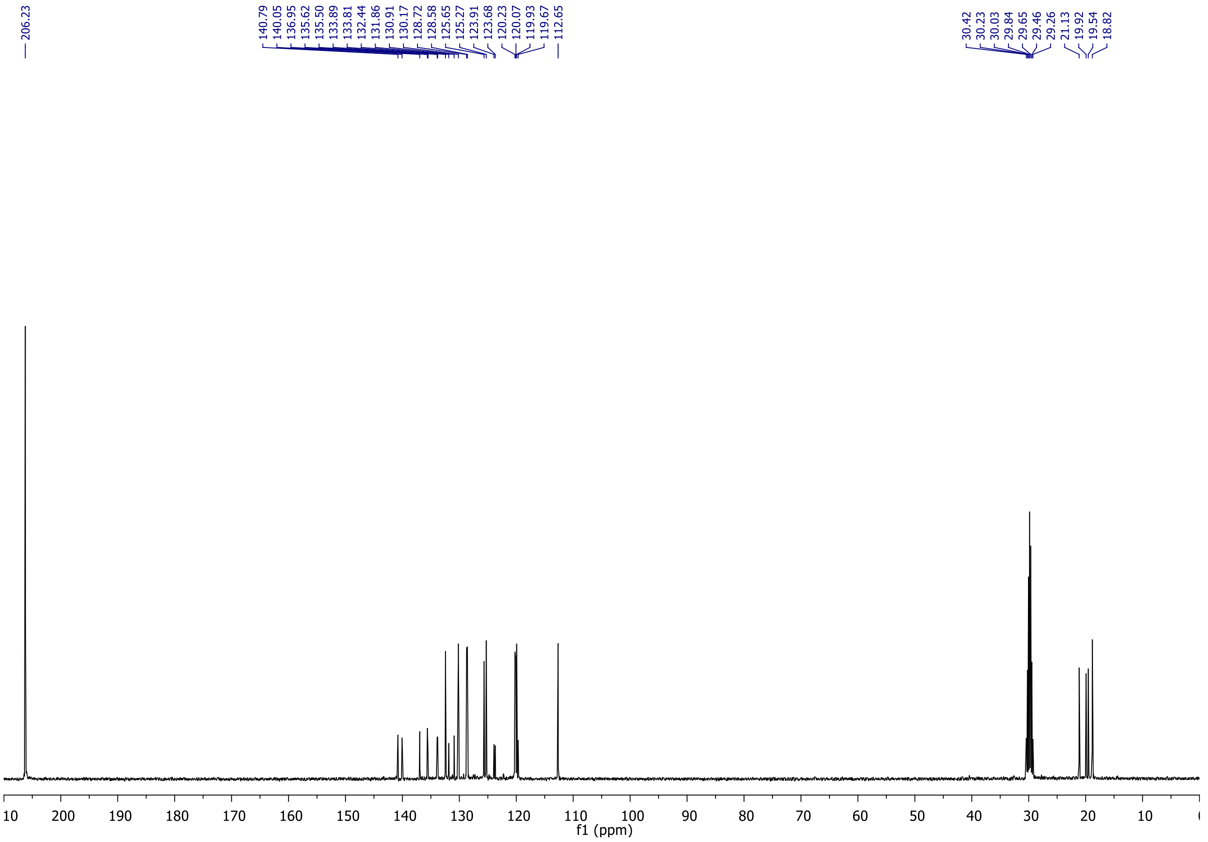

^{1}H NMR (400 MHz) and ^{13}C{^{1}H} NMR (100 MHz) spectra of **2l-1** (Acetone-d_6)

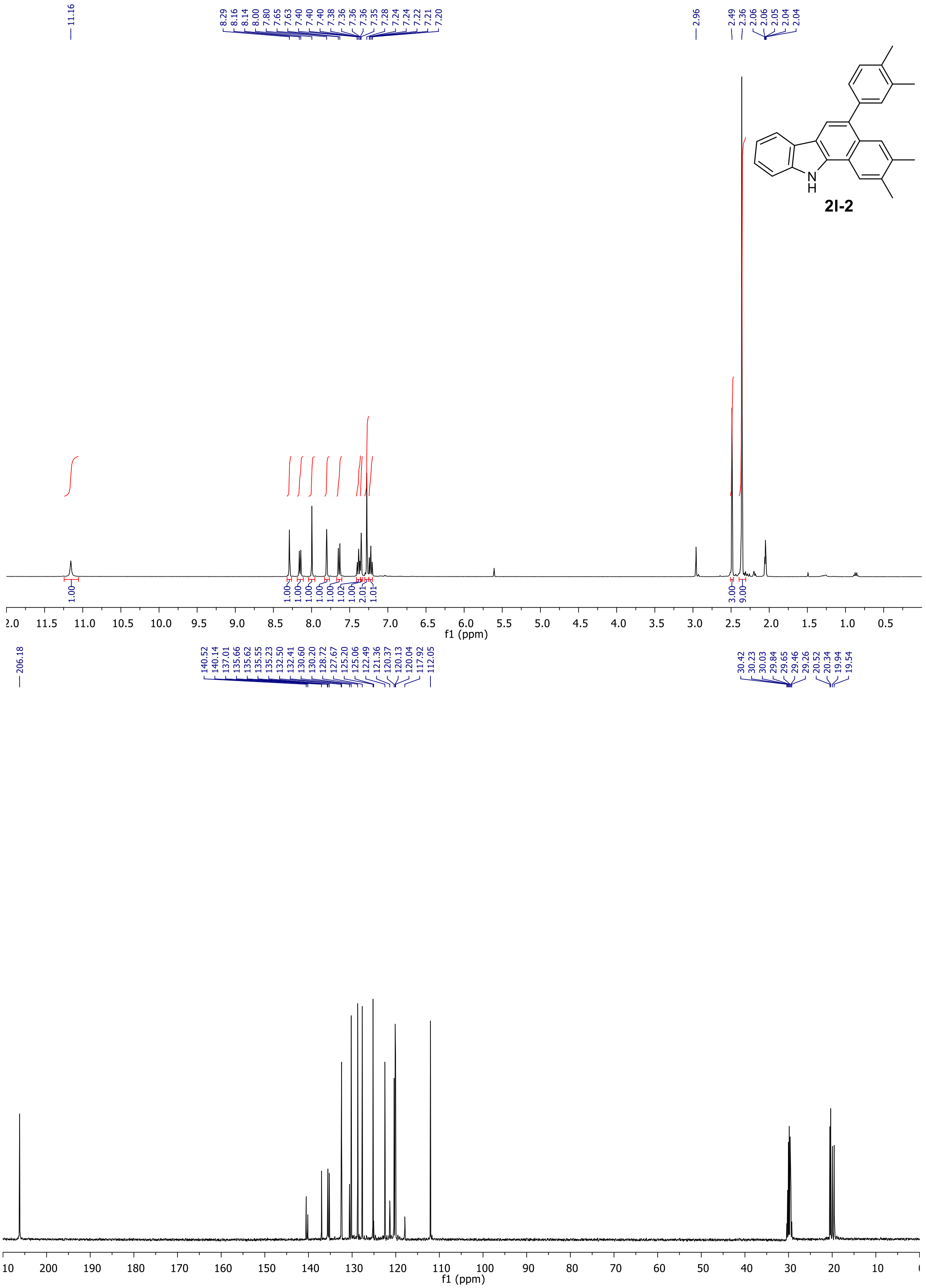

^{1}H NMR (400 MHz) and $^{13}C\{^{1}H\}$ NMR (100 MHz) spectra of **2l-2** (Acetone-d_6)

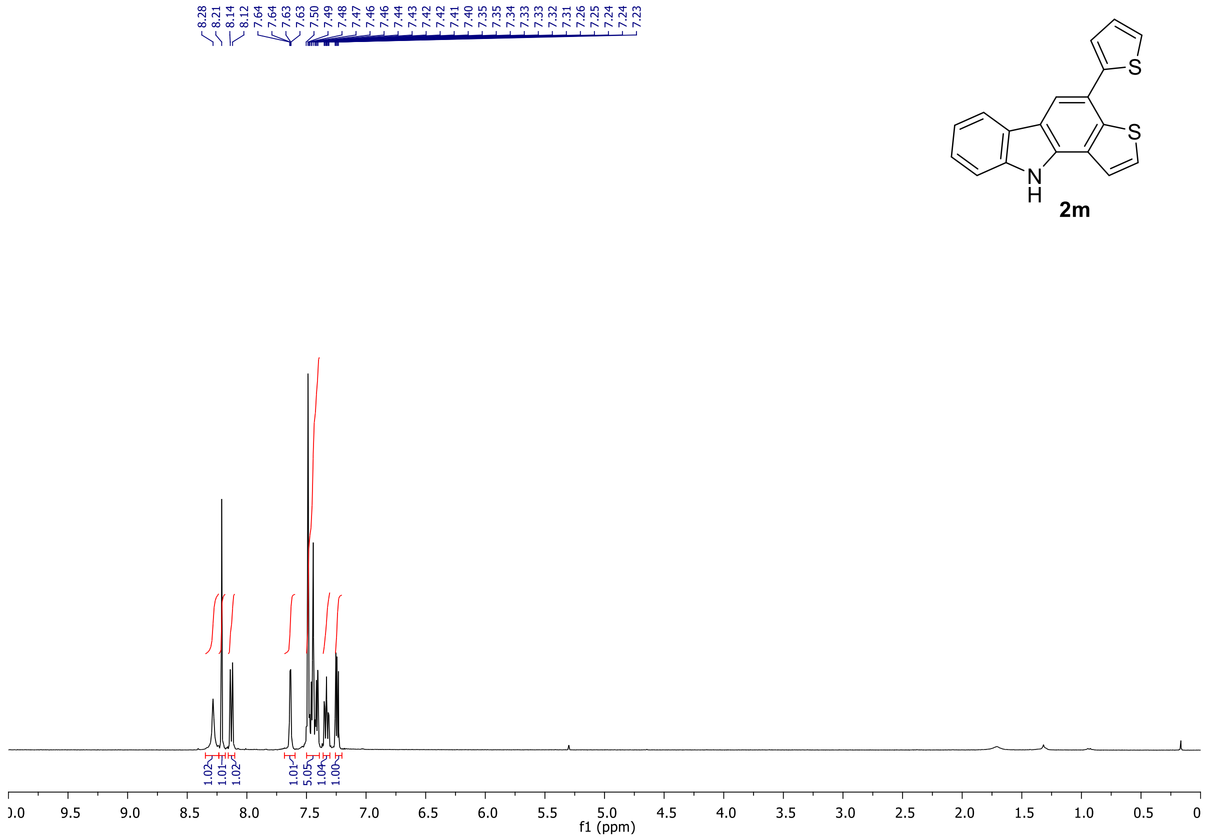

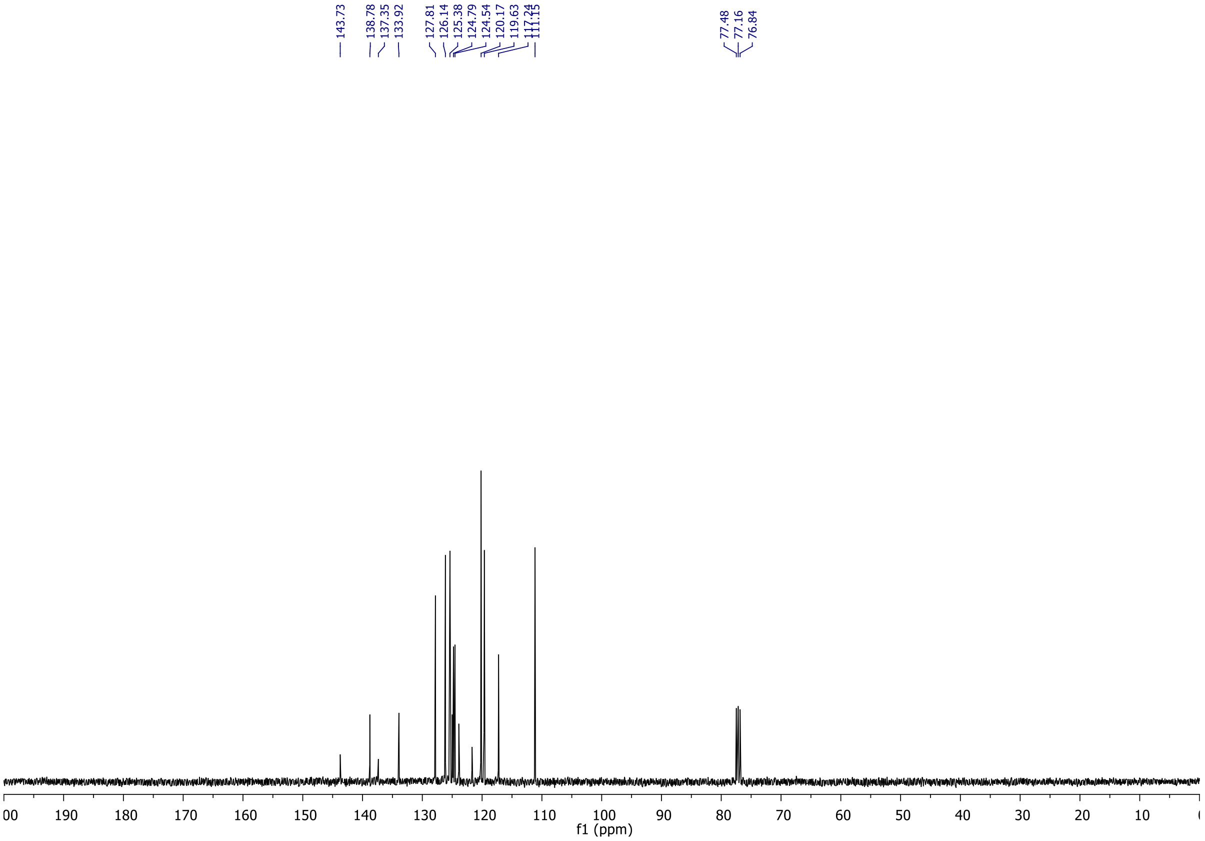

^{1}H NMR (400 MHz) and ^{13}C{^{1}H} NMR (100 MHz) spectra of **2m** ($CDCl_3$)

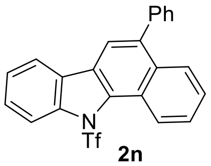

2n

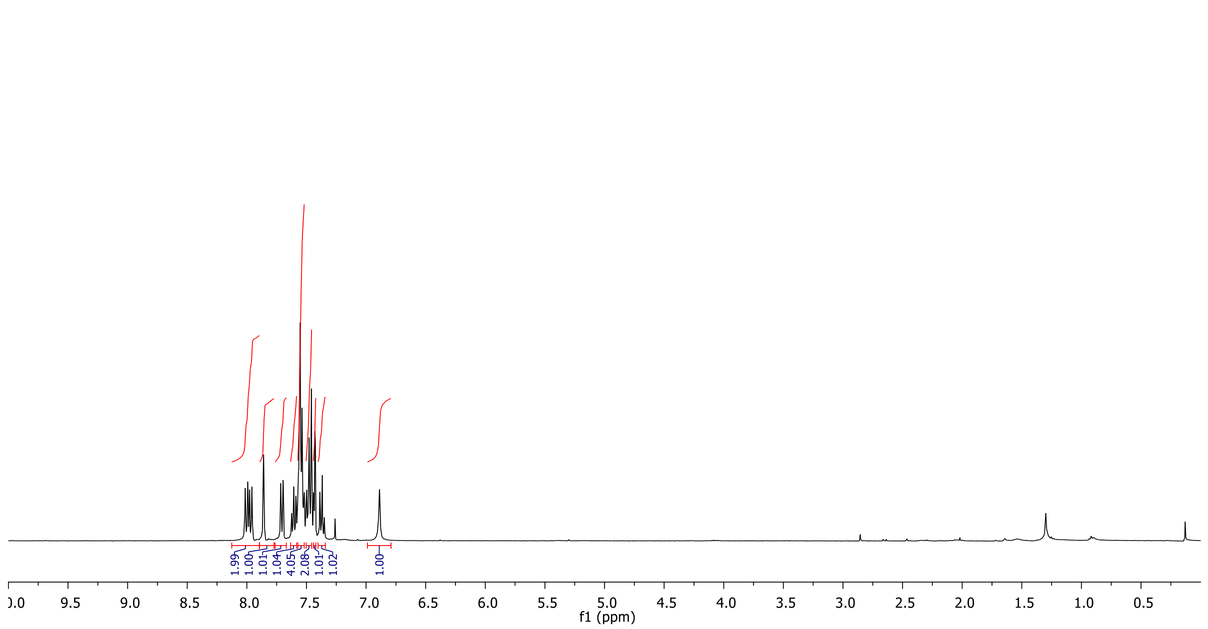

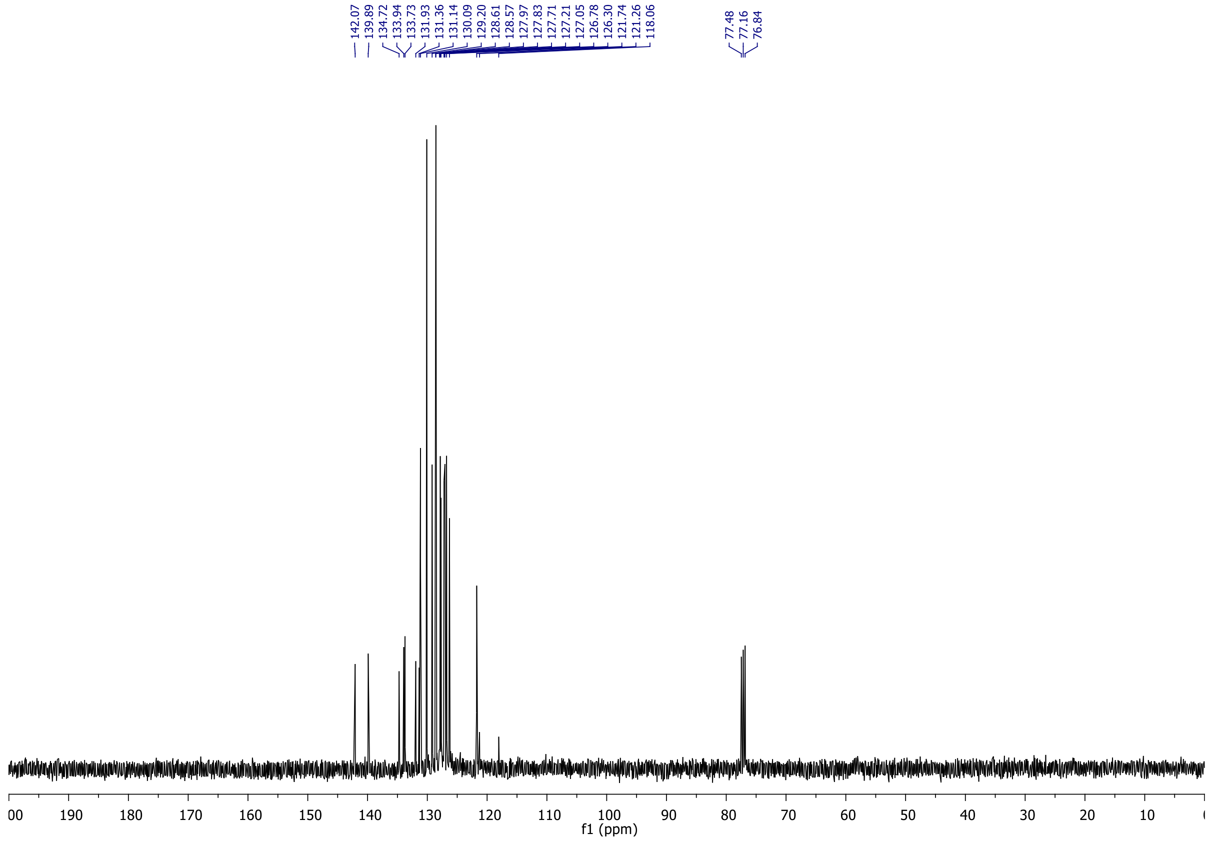

^{1}H NMR (400 MHz) and ^{13}C{^{1}H} NMR (100 MHz) spectra of **2n** ($CDCl_3$)

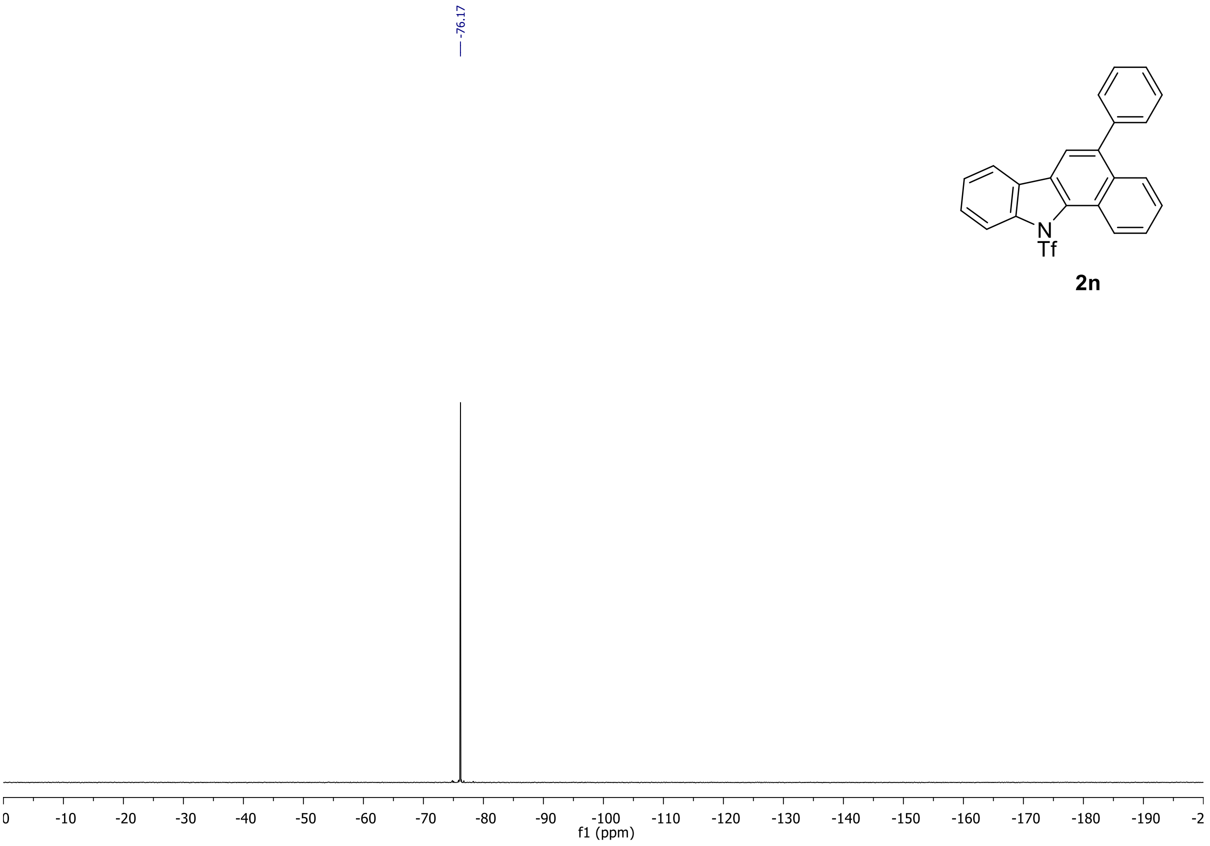

^{19}F NMR (376 MHz) spectra of **2n** ($CDCl_3$)

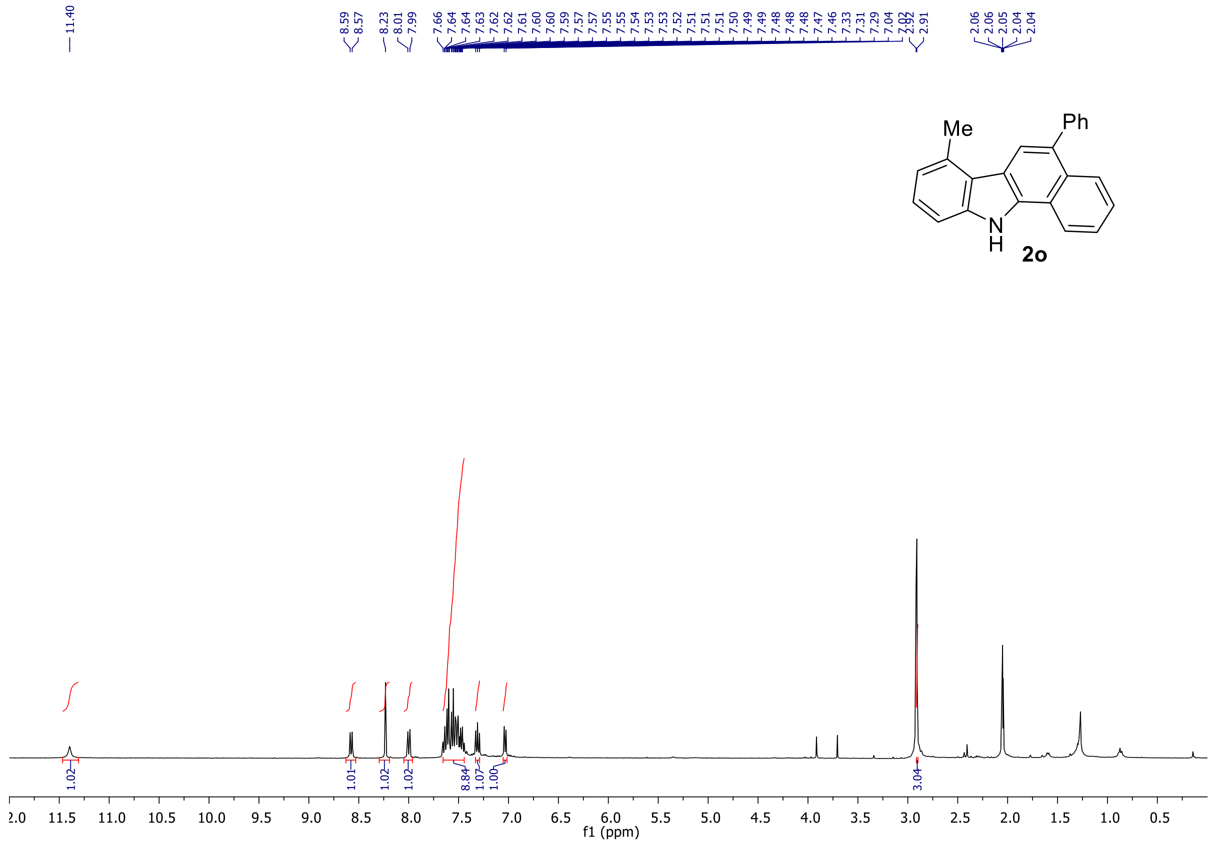

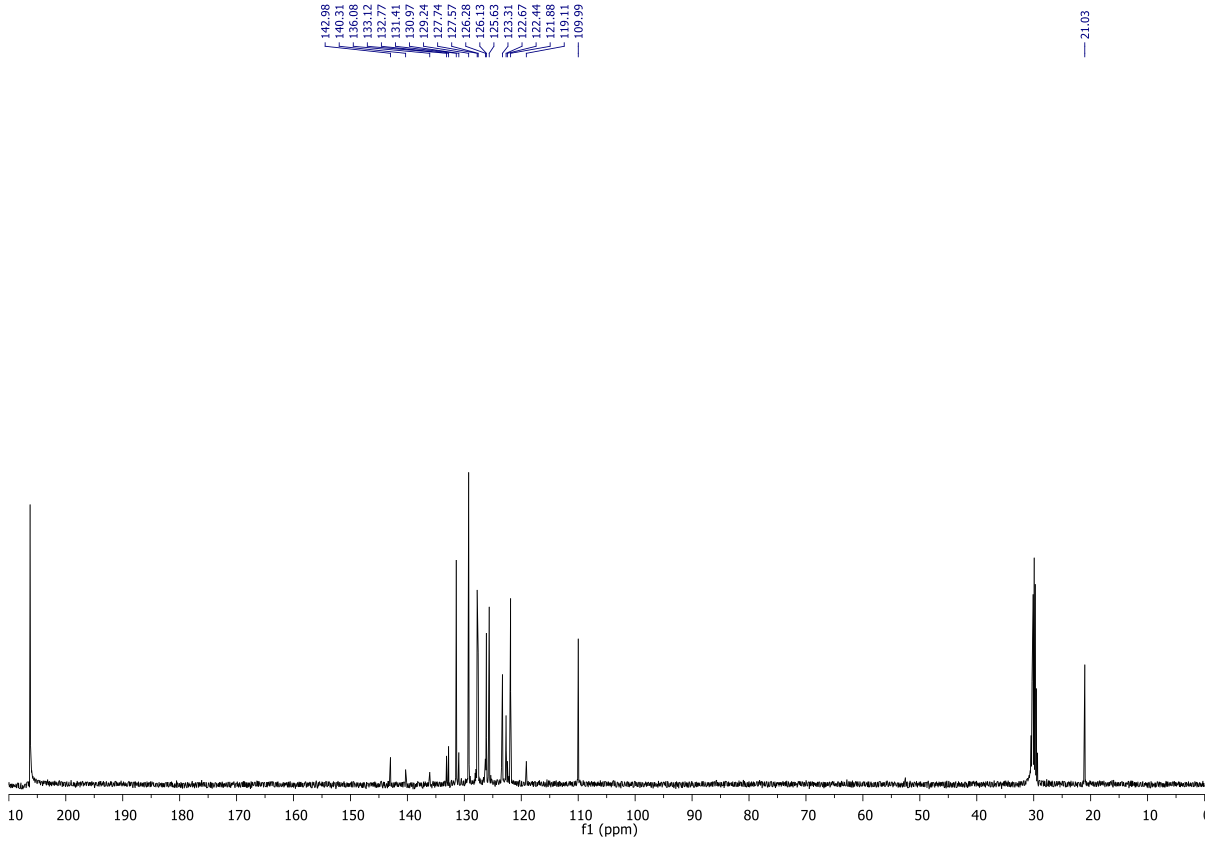

^{1}H NMR (400 MHz) and $^{13}C\{^{1}H\}$ NMR (100 MHz) spectra of **2o** (Acetone-d_6)

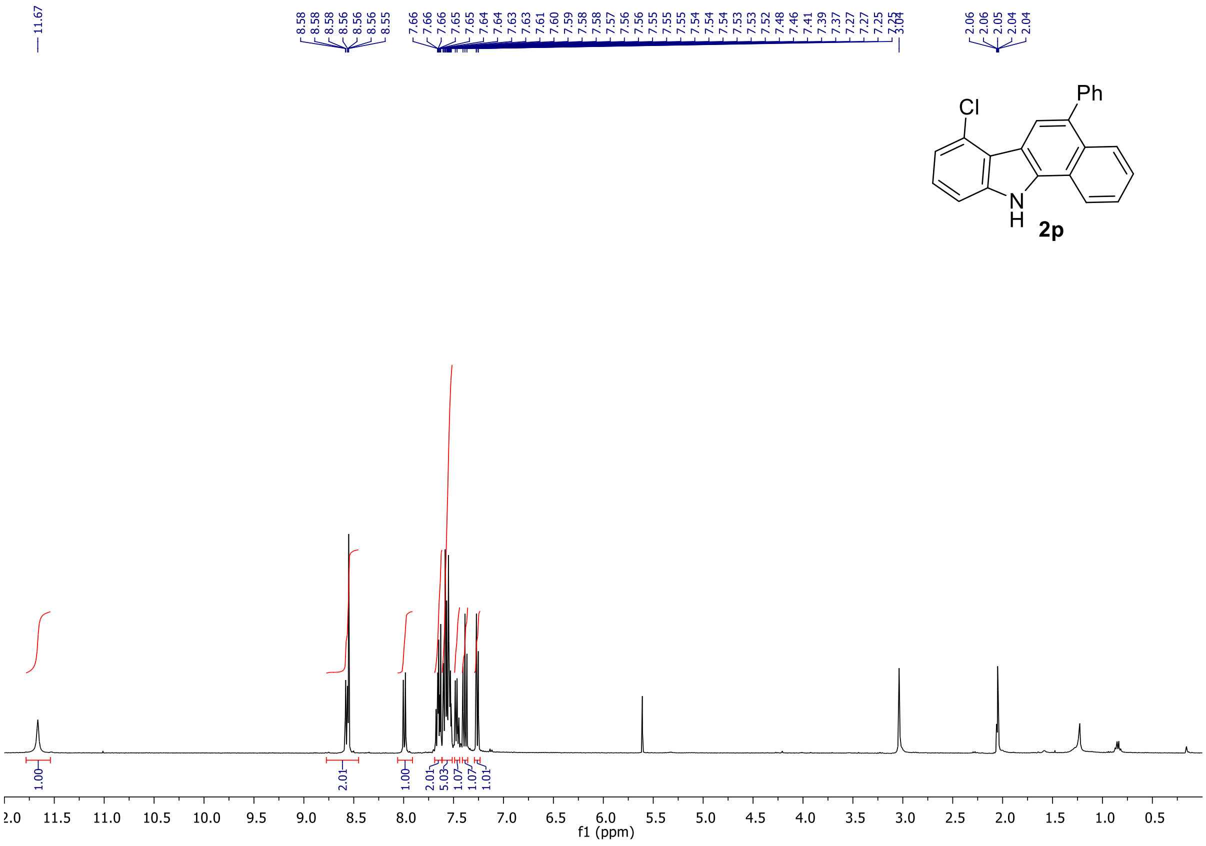

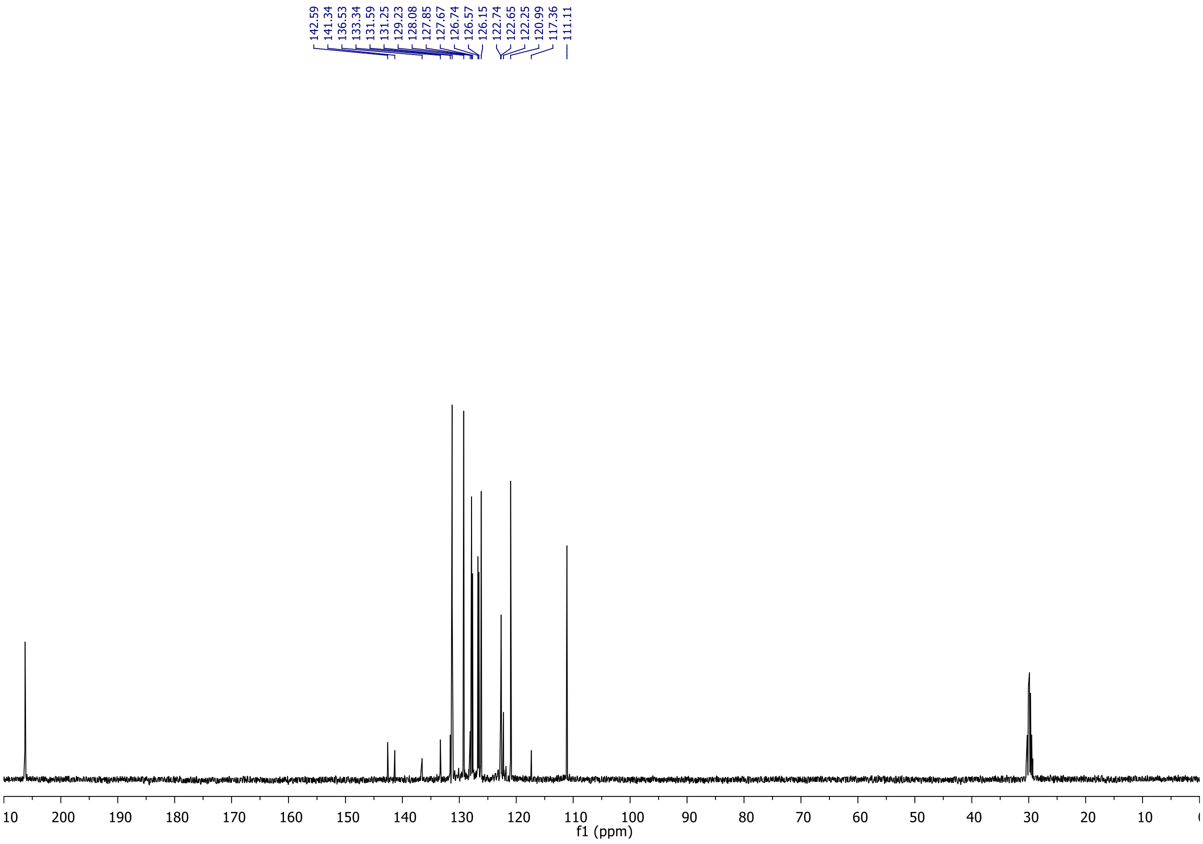

^{1}H NMR (400 MHz) and ^{13}C{^{1}H} NMR (100 MHz) spectra of **2p** (Acetone-d_6)

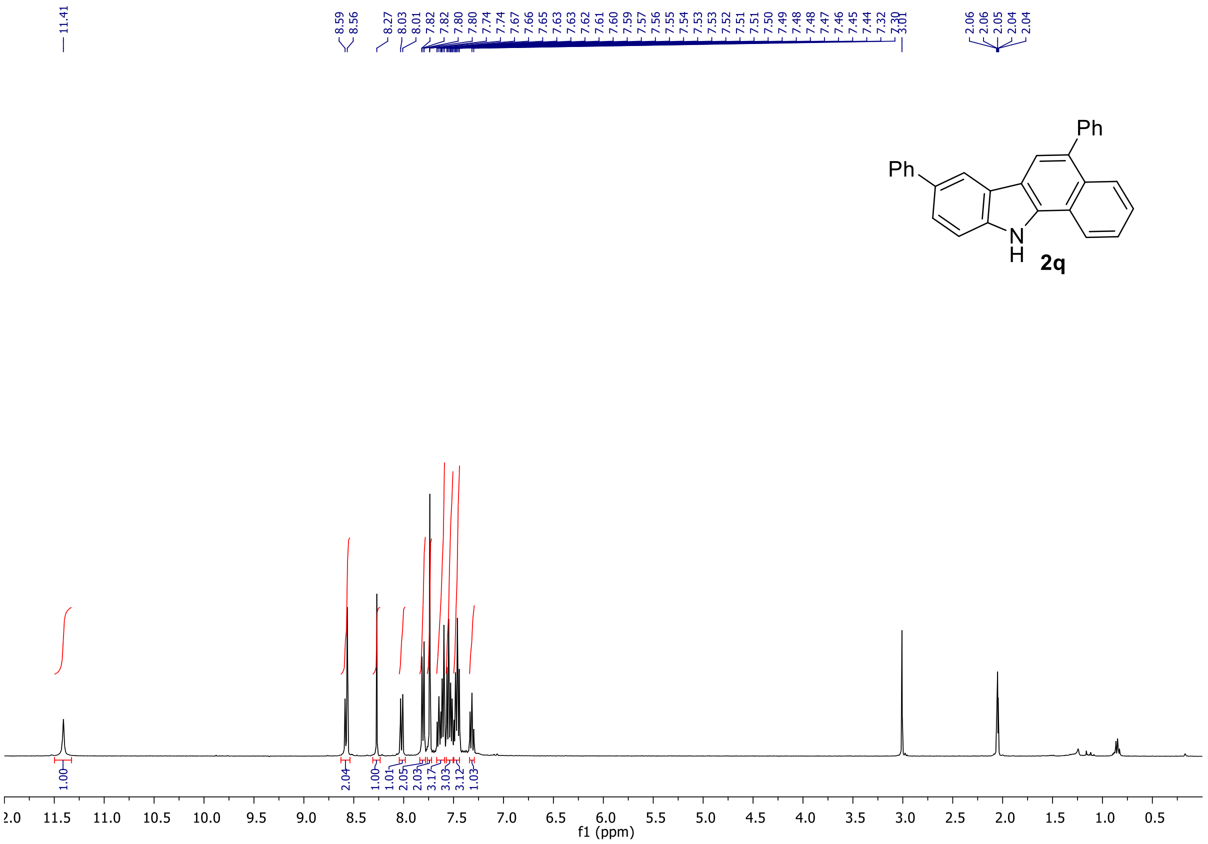

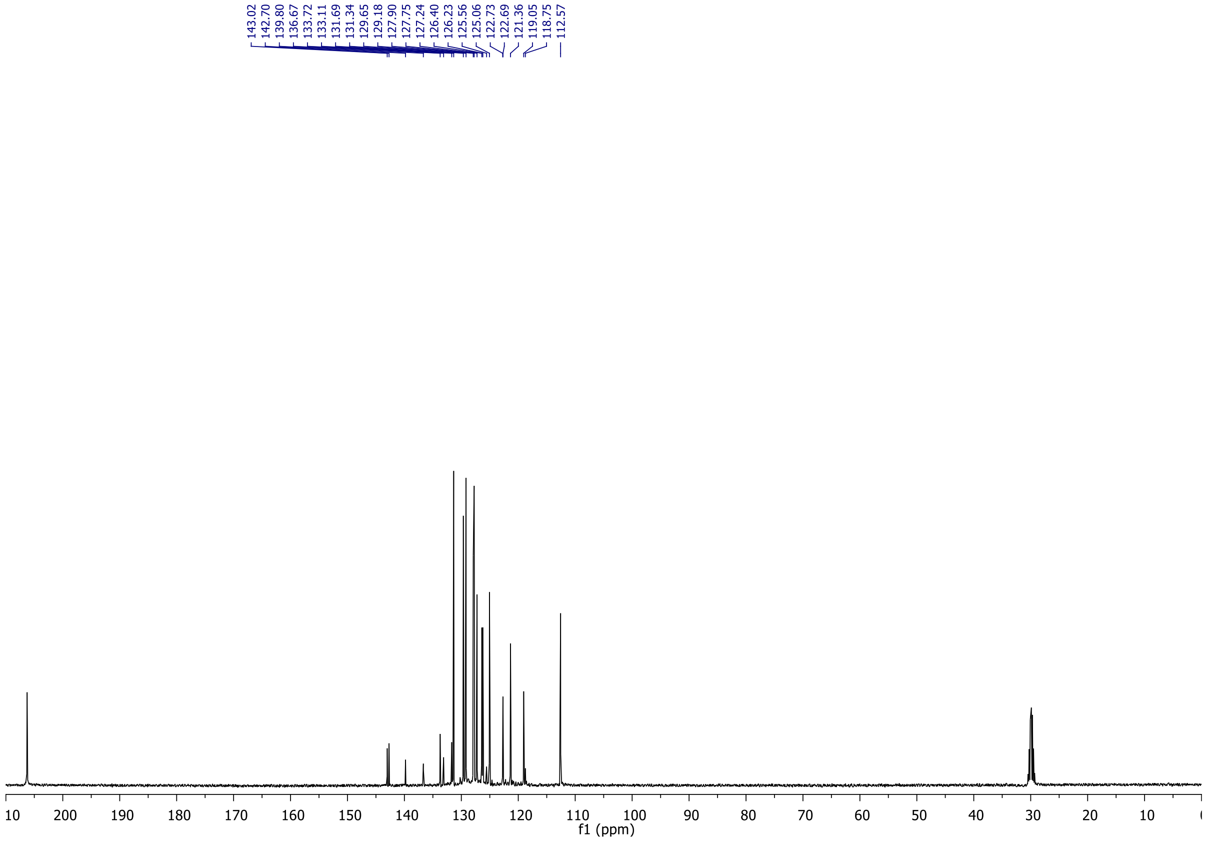

^{1}H NMR (400 MHz) and $^{13}C\{^{1}H\}$ NMR (100 MHz) spectra of **2q** (Acetone-d_6)

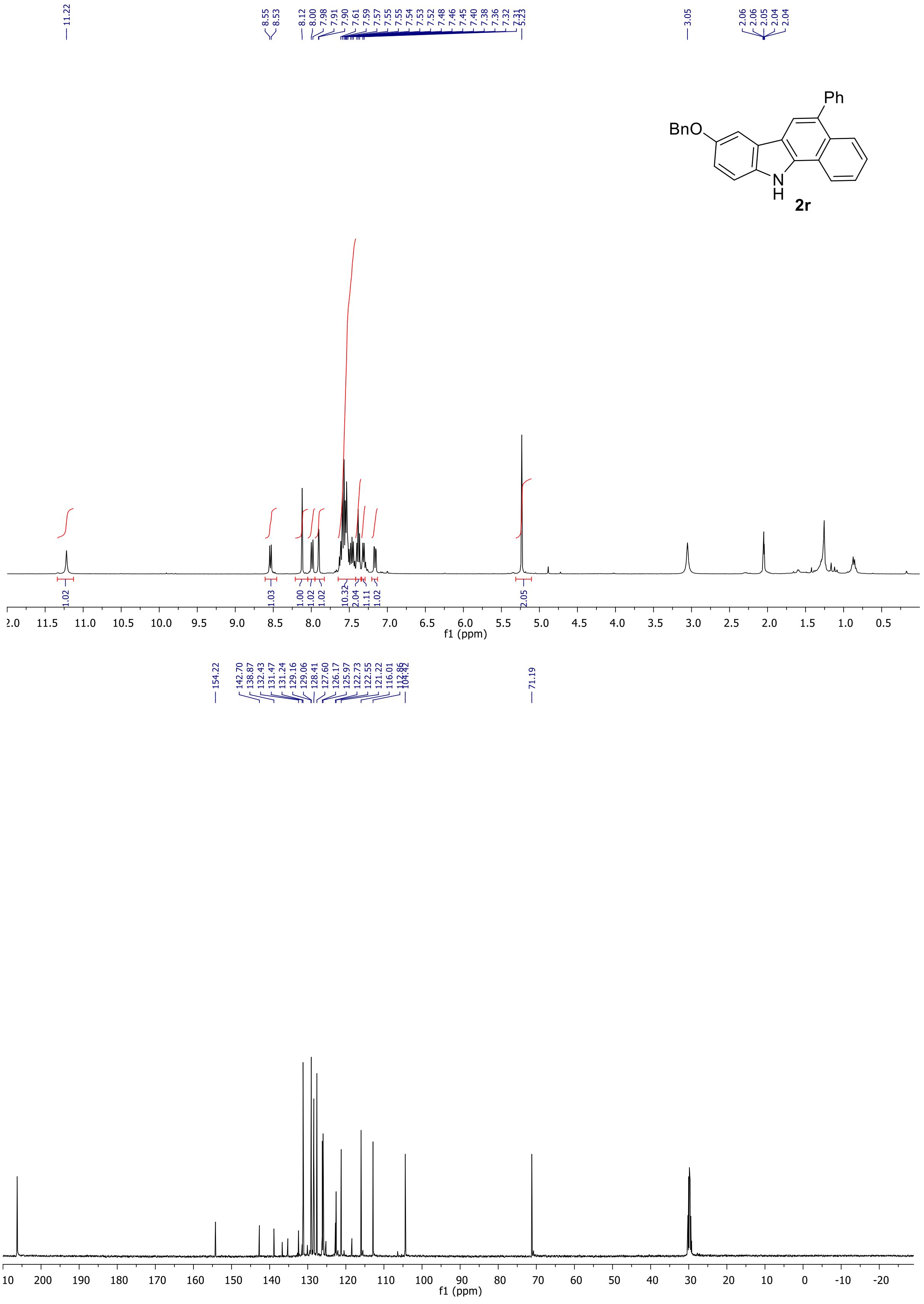

^{1}H NMR (400 MHz) and ^{13}C{^{1}H} NMR (100 MHz) spectra of **2r** (Acetone-d_6)

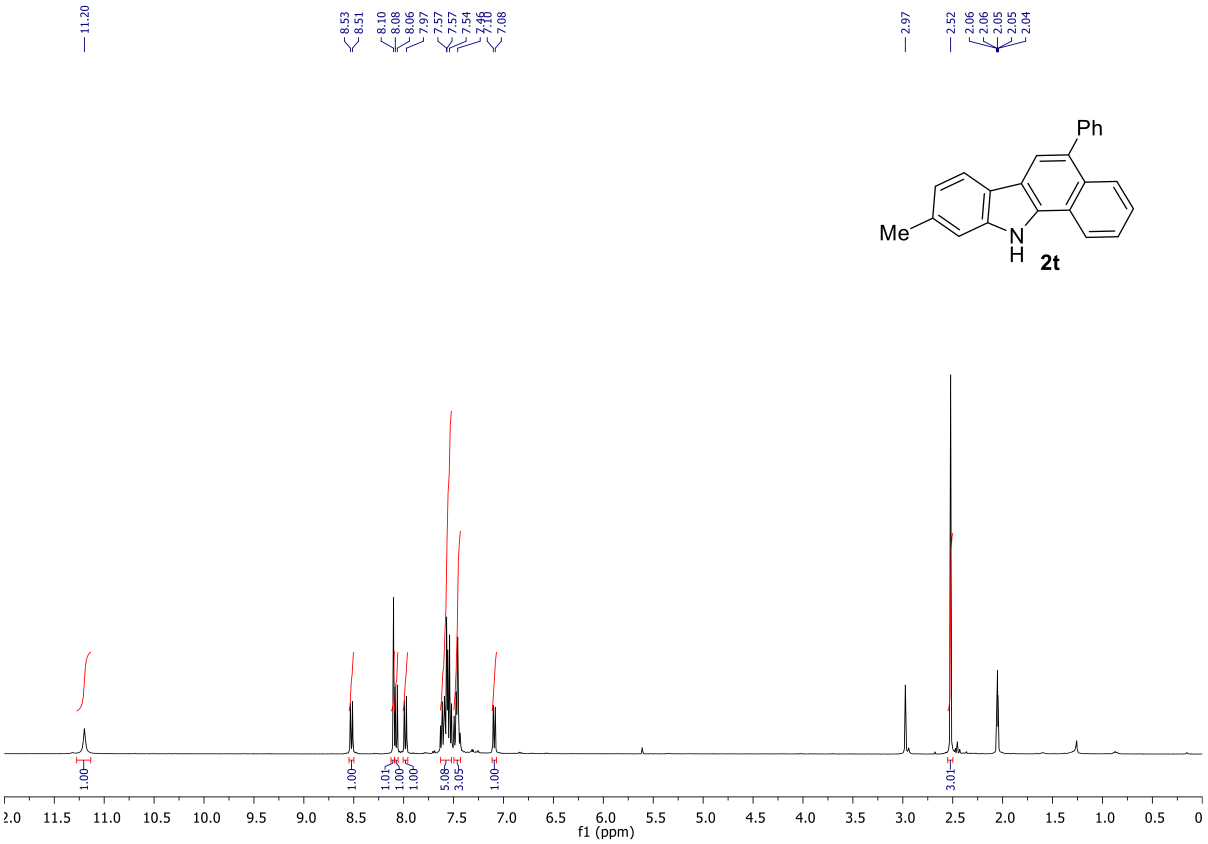

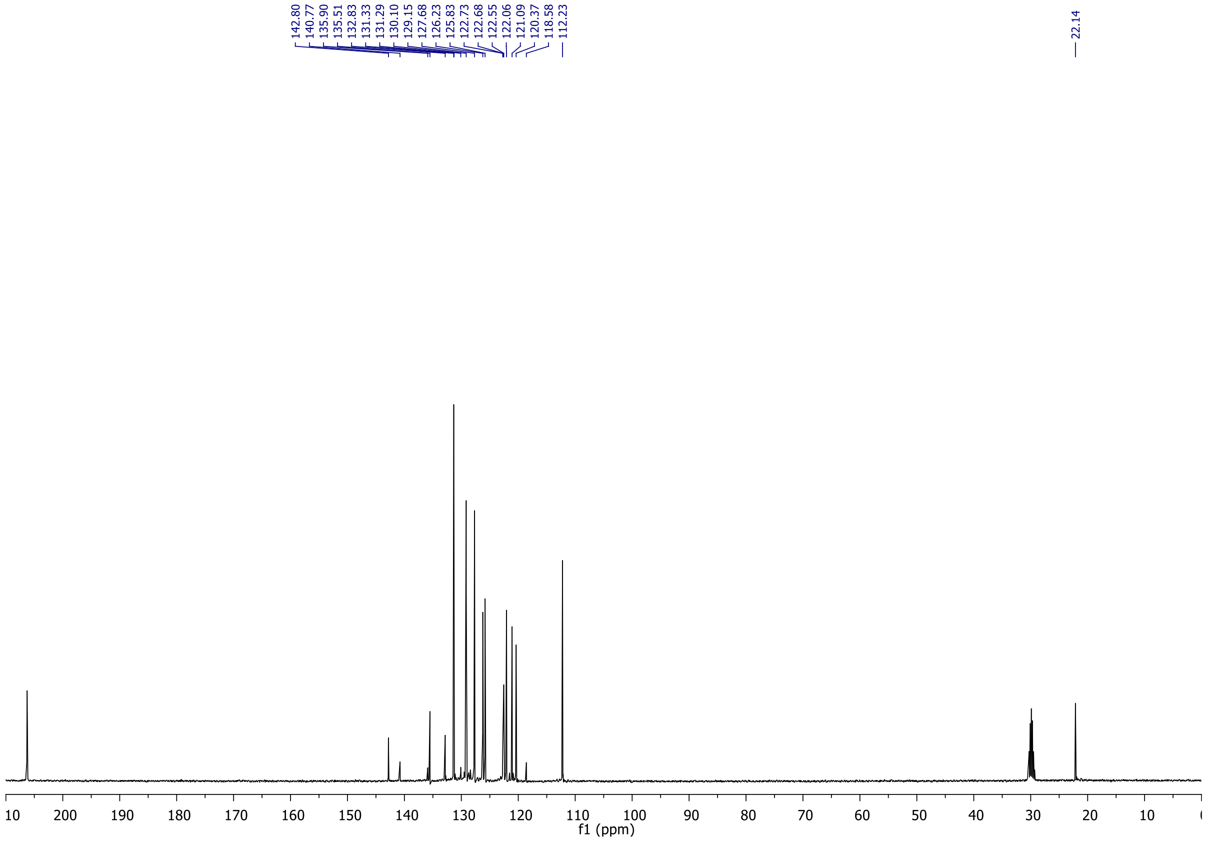

^{1}H NMR (400 MHz) and ^{13}C{^{1}H} NMR (100 MHz) spectra of **2t** (Acetone-d_6)

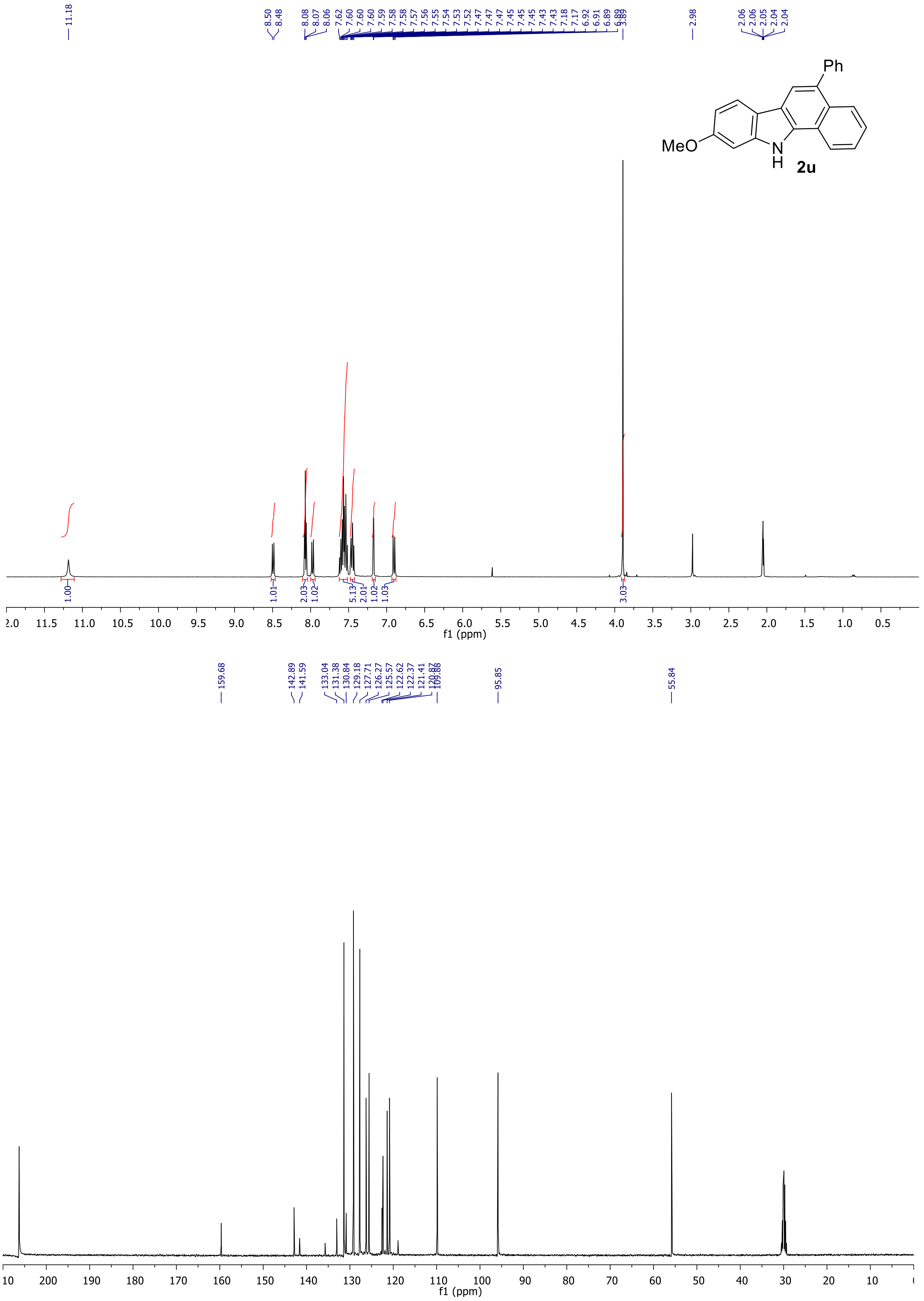

^{1}H NMR (400 MHz) and ^{13}C{^{1}H} NMR (100 MHz) spectra of **2u** (Acetone-d_6)

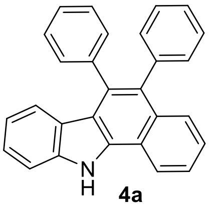

4a

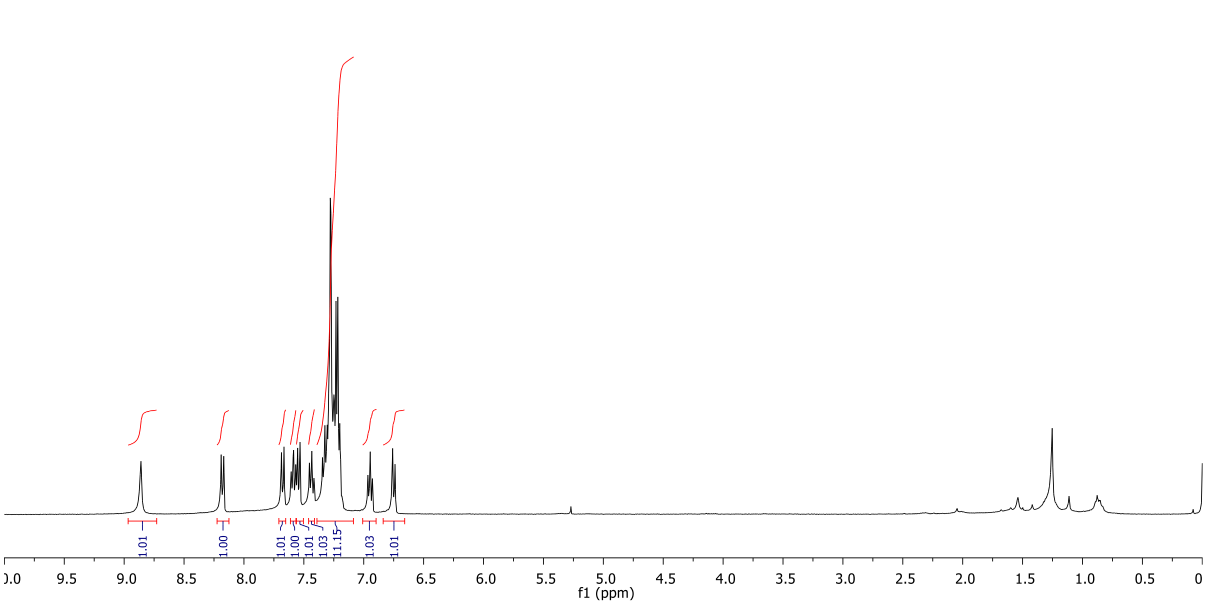

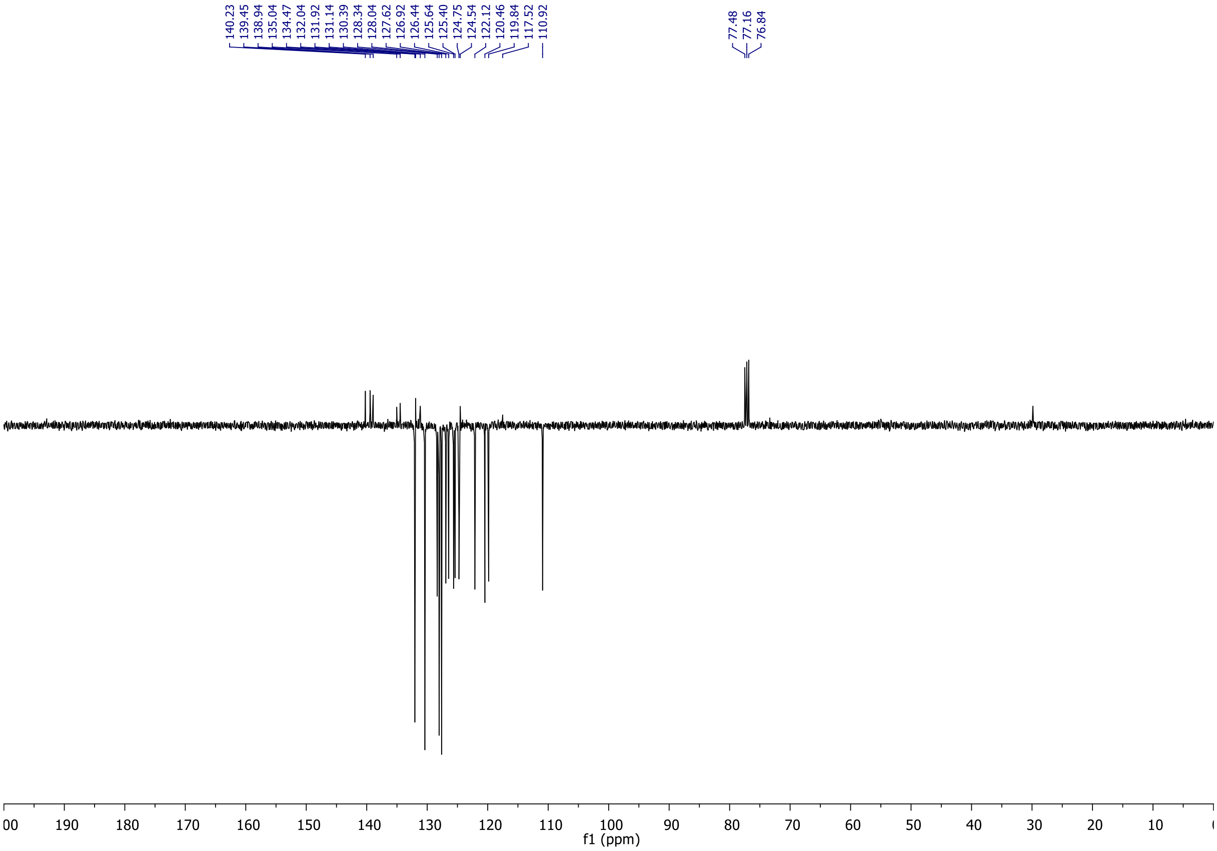

^{1}H NMR (400 MHz) and ^{13}C{^{1}H} (APT) NMR (100 MHz) spectra of **4a** ($CDCl_3$)

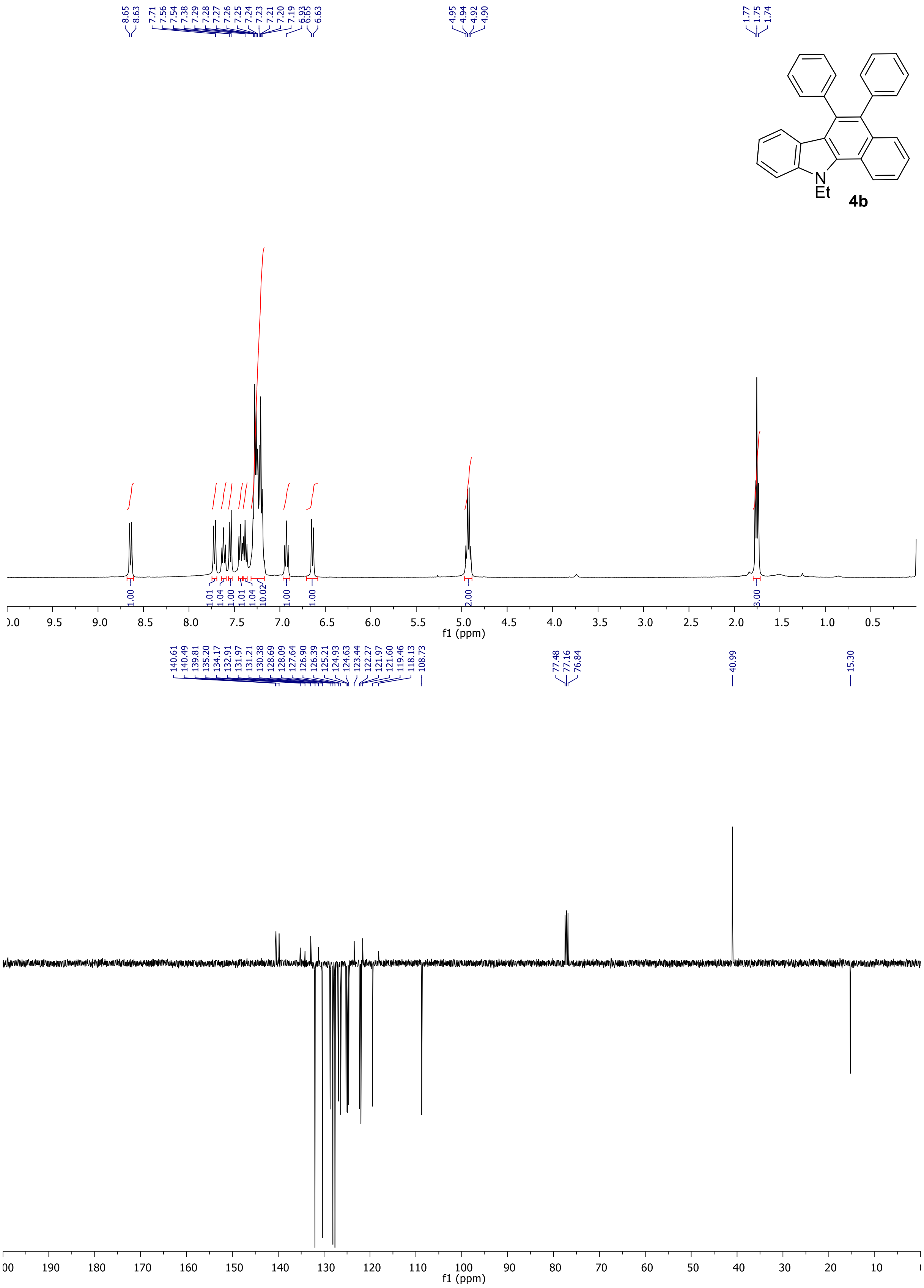

^{1}H NMR (400 MHz) and $^{13}C\{^{1}H\}$ (APT) NMR (100 MHz) spectra of **4b** ($CDCl_3$)

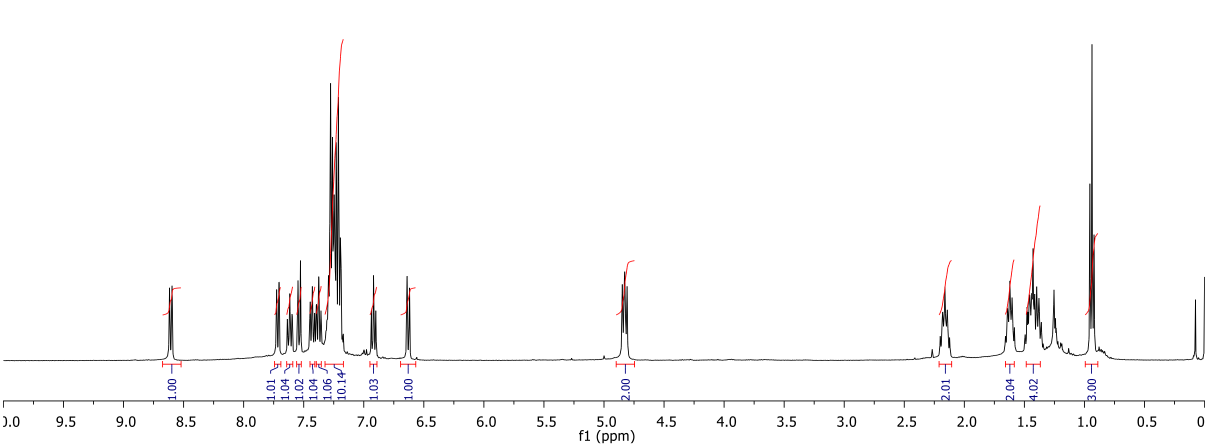

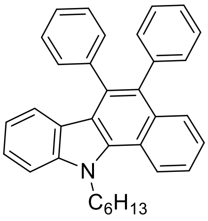

4c

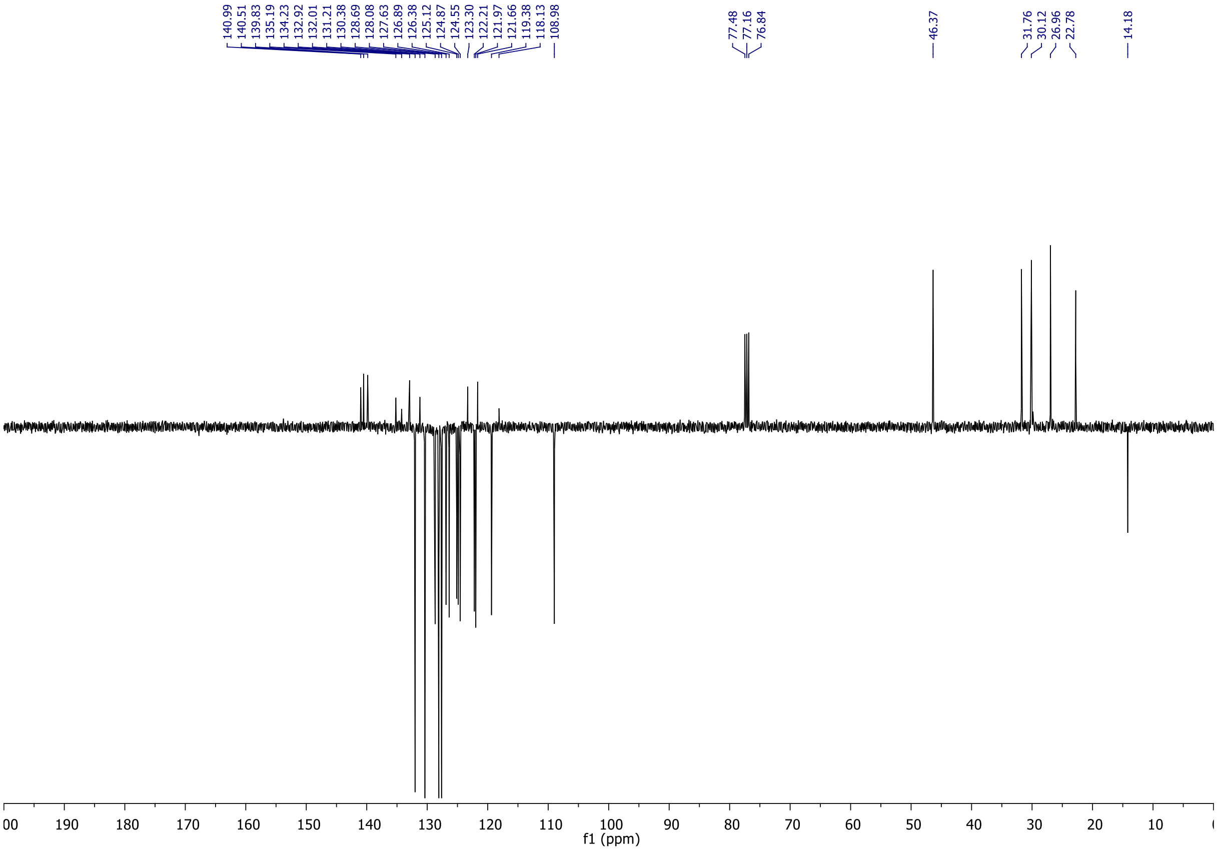

^{1}H NMR (400 MHz) and ^{13}C{^{1}H} (APT) NMR (100 MHz) spectra of **4c** ($CDCl_3$)

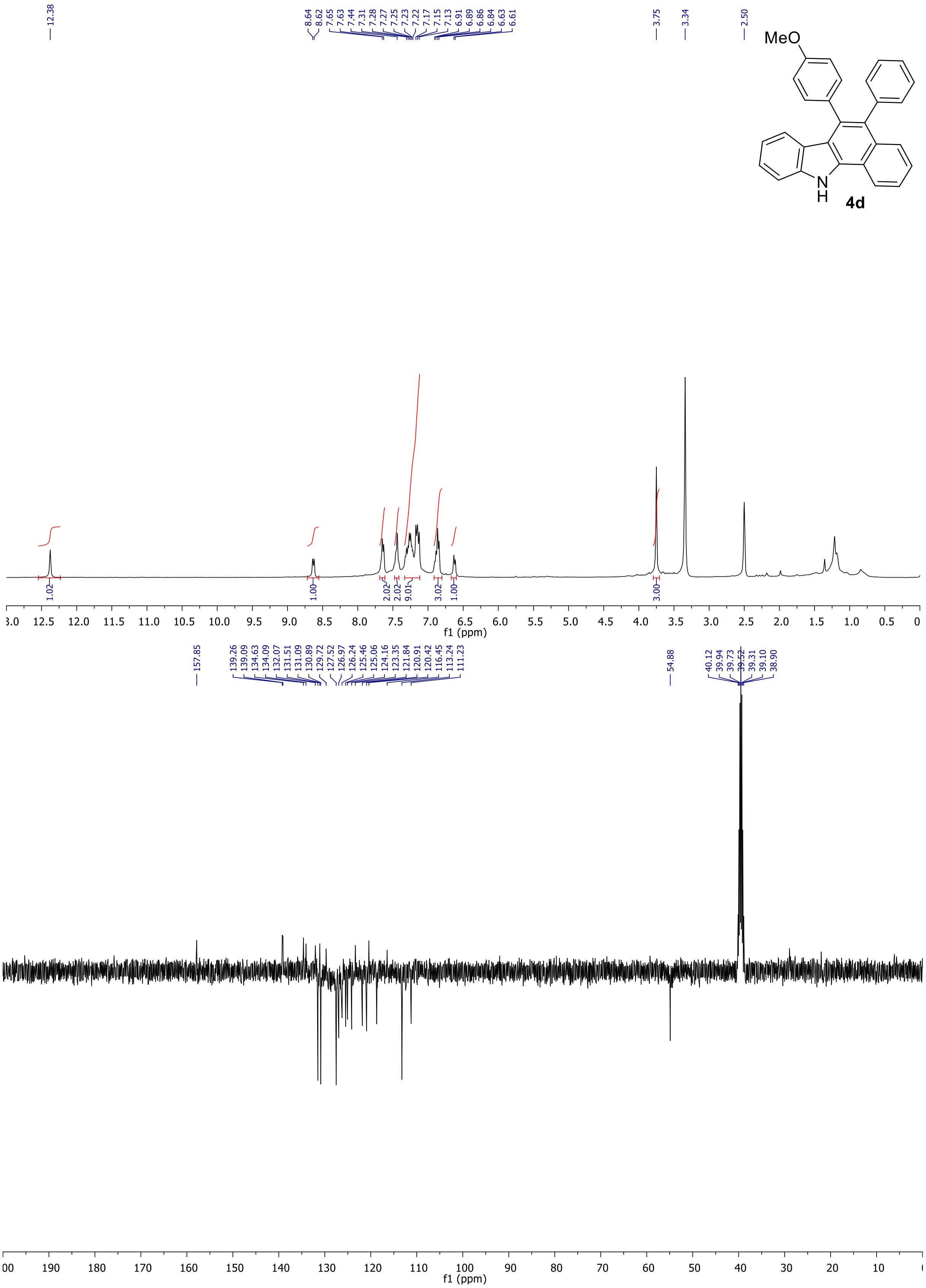

^{1}H NMR (400 MHz) and ^{13}C{^{1}H} NMR (APT) (100 MHz) spectra of **4d** (DMSO-d_6)

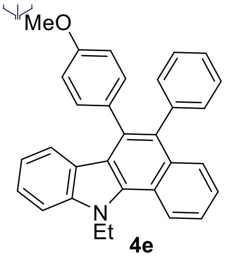

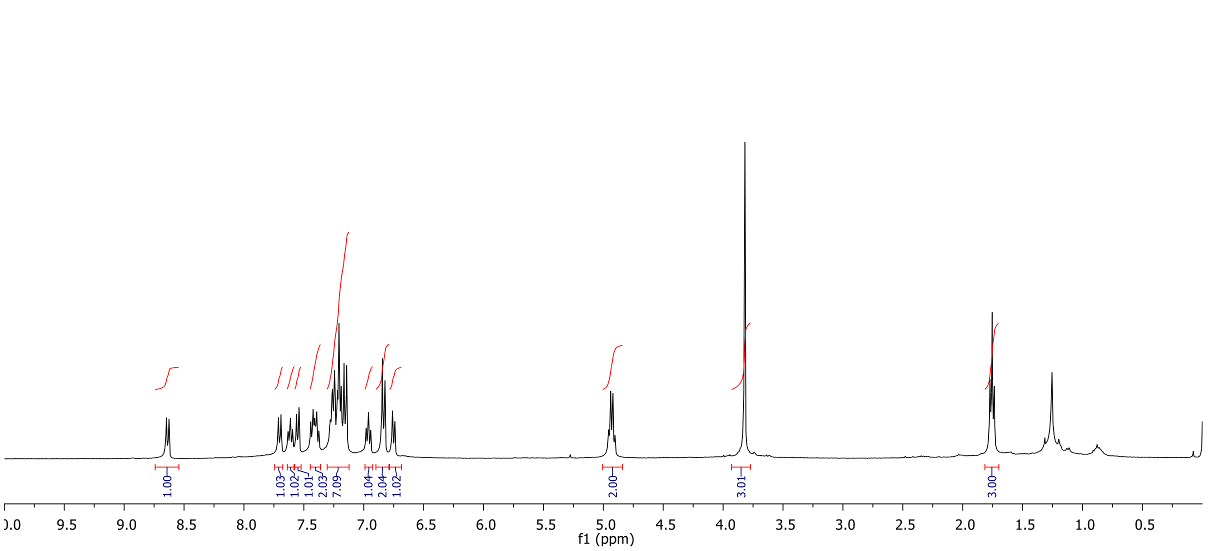

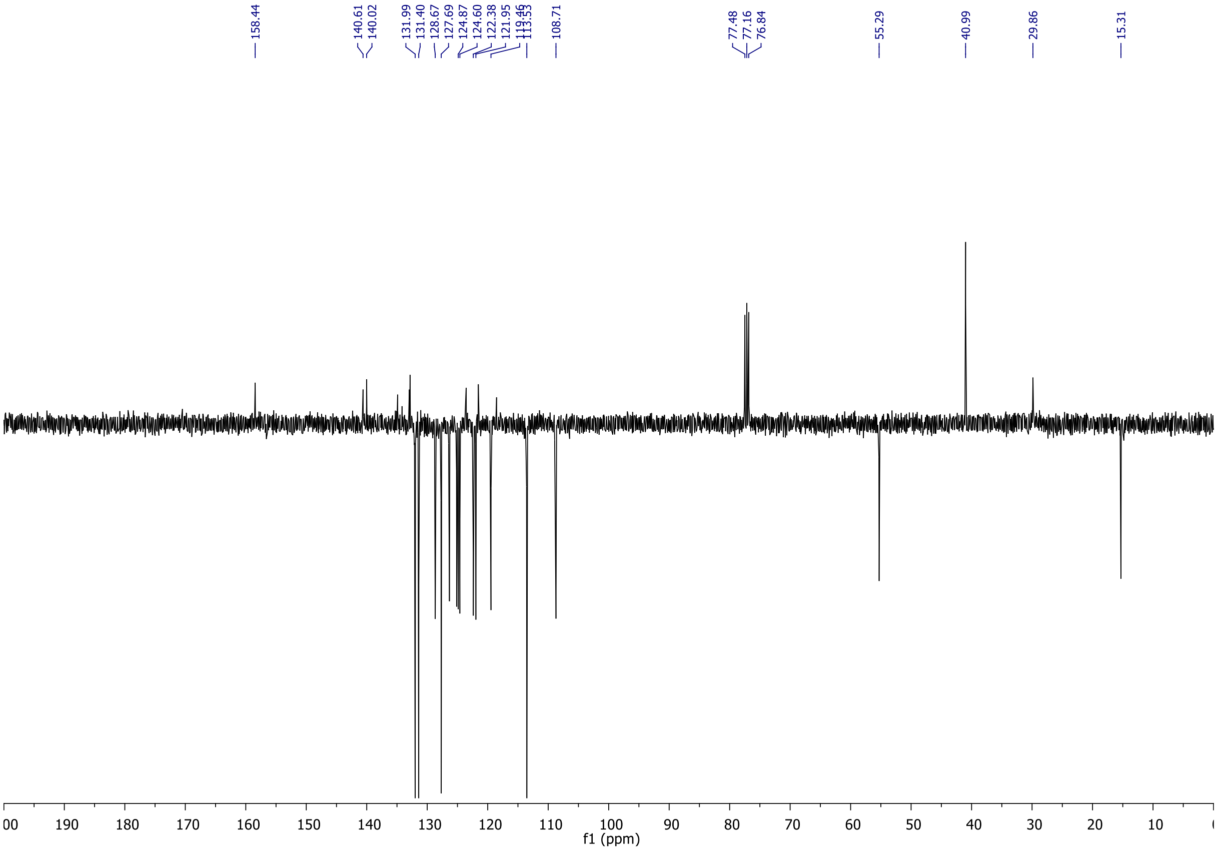

^{1}H NMR (400 MHz) and ^{13}C{^{1}H} (APT) NMR (100 MHz) spectra of **4e** ($CDCl_3$)

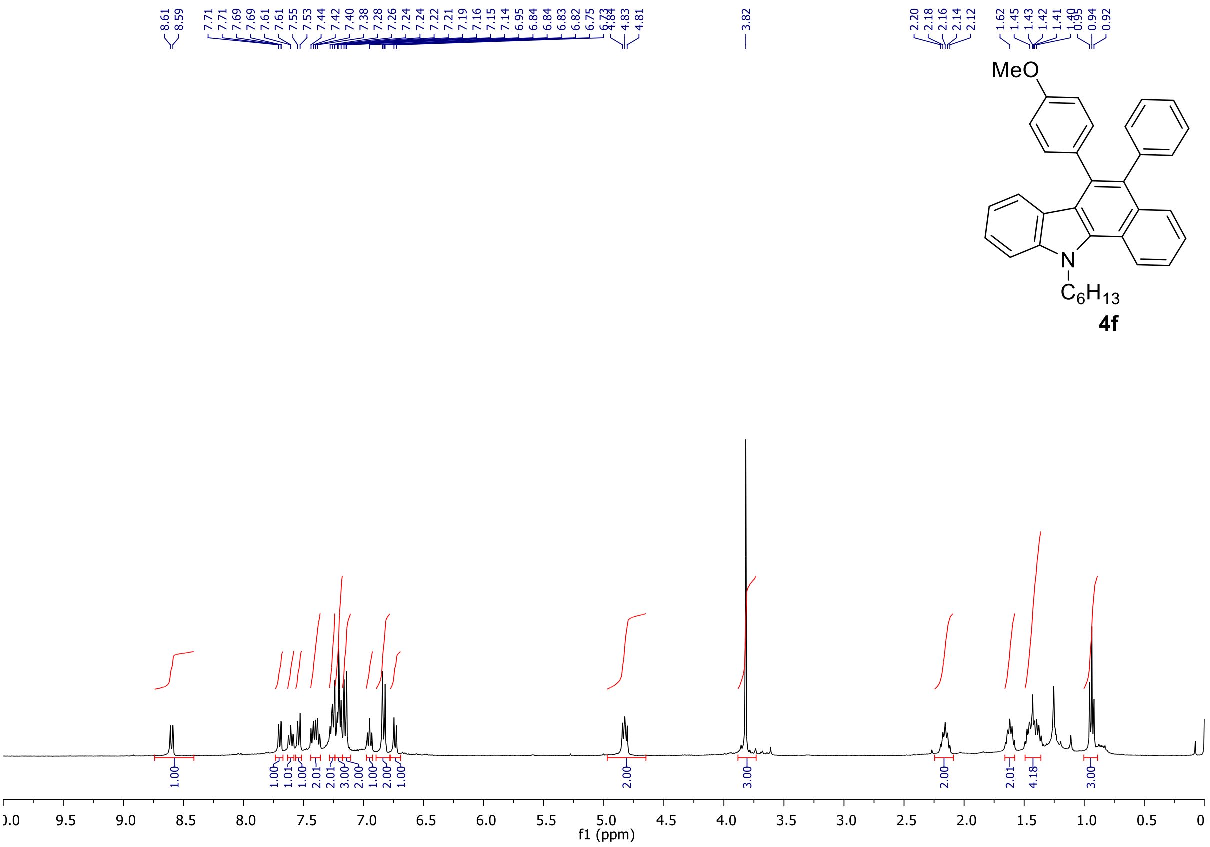

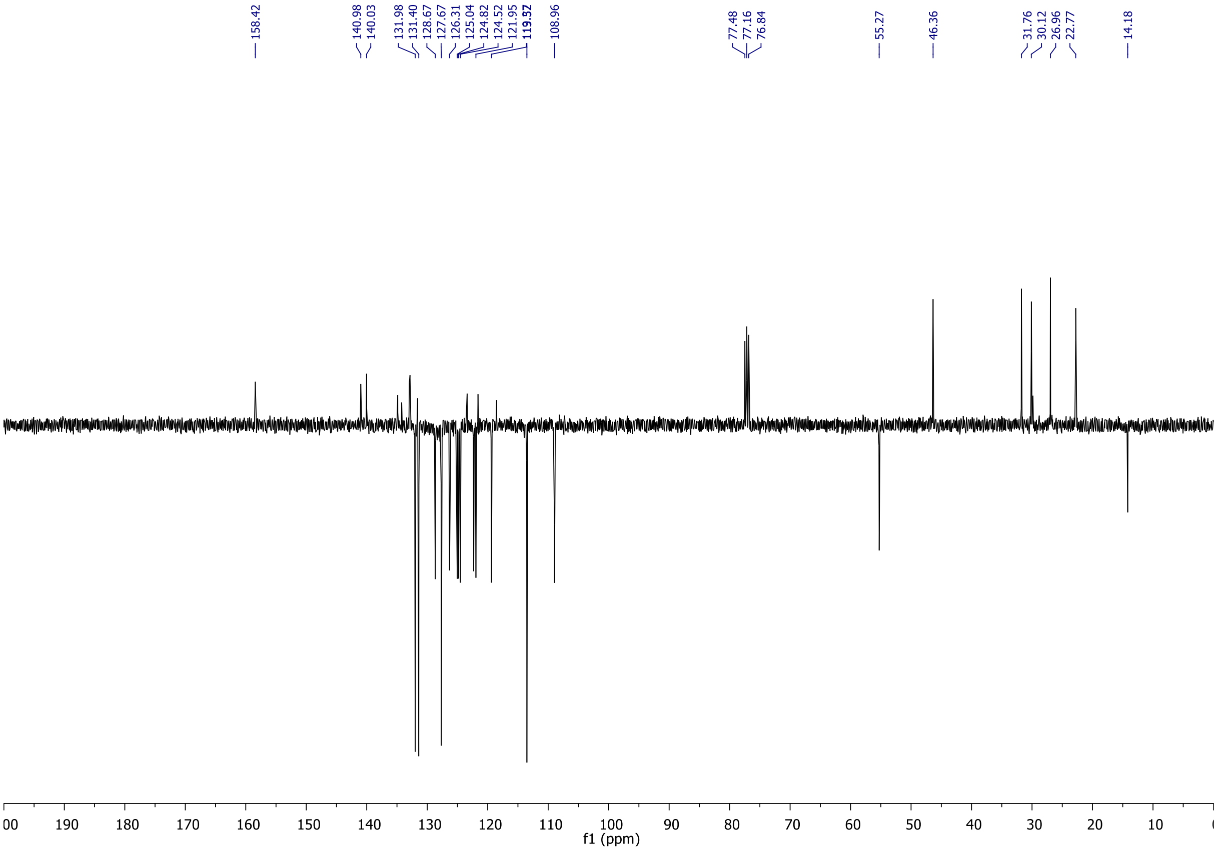

^{1}H NMR (400 MHz) and $^{13}C\{^{1}H\}$ (APT) NMR (100 MHz) spectra of **4f** ($CDCl_3$)

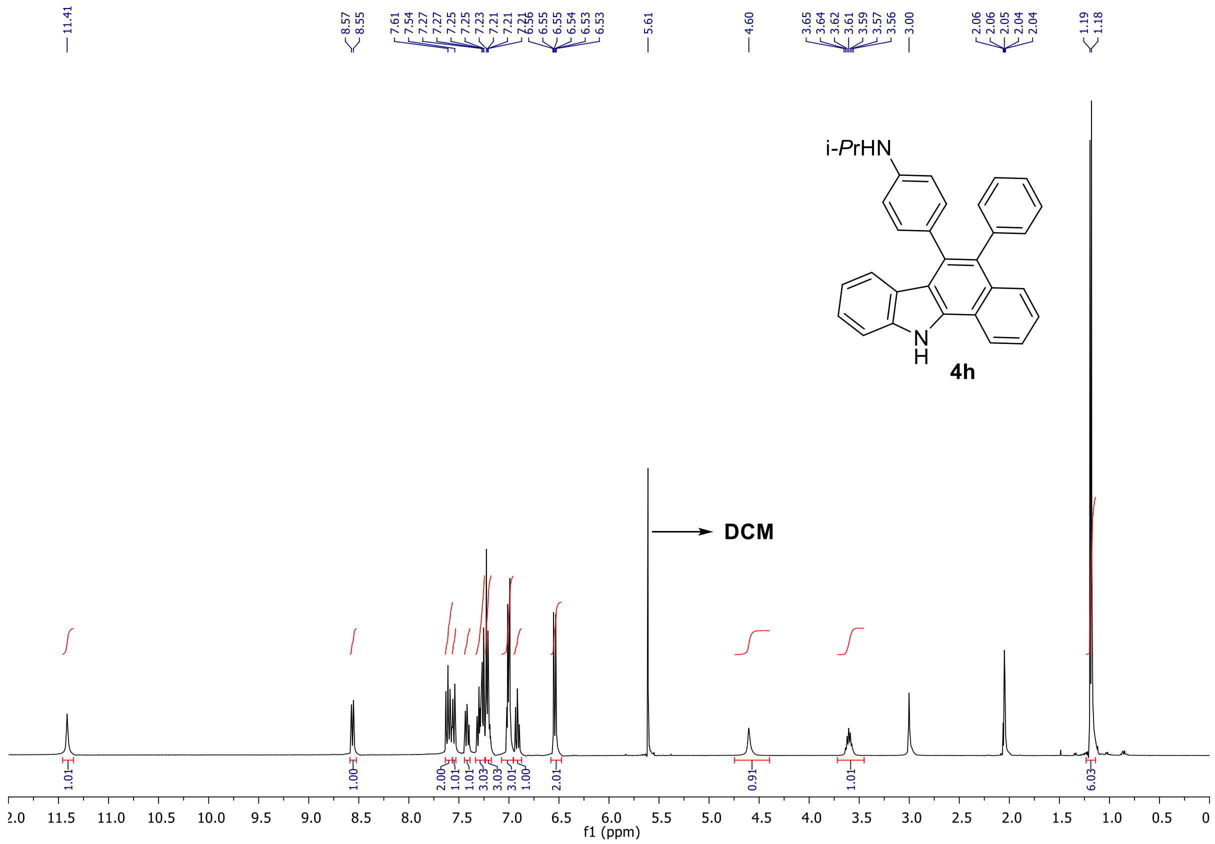

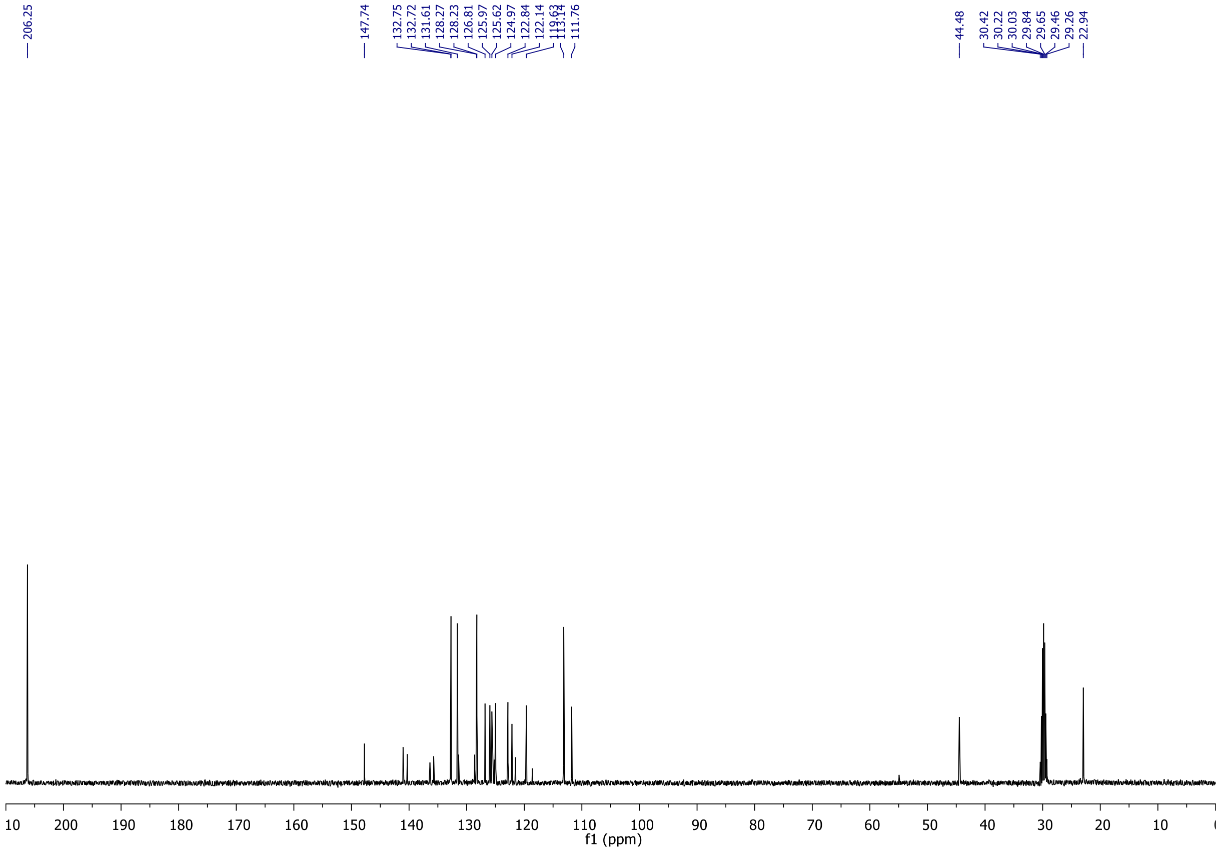

^{1}H NMR (400 MHz) and ^{13}C{^{1}H} NMR (100 MHz) spectra of **4h** (Acetone-d_6)

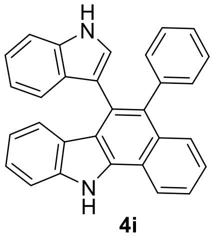

4i

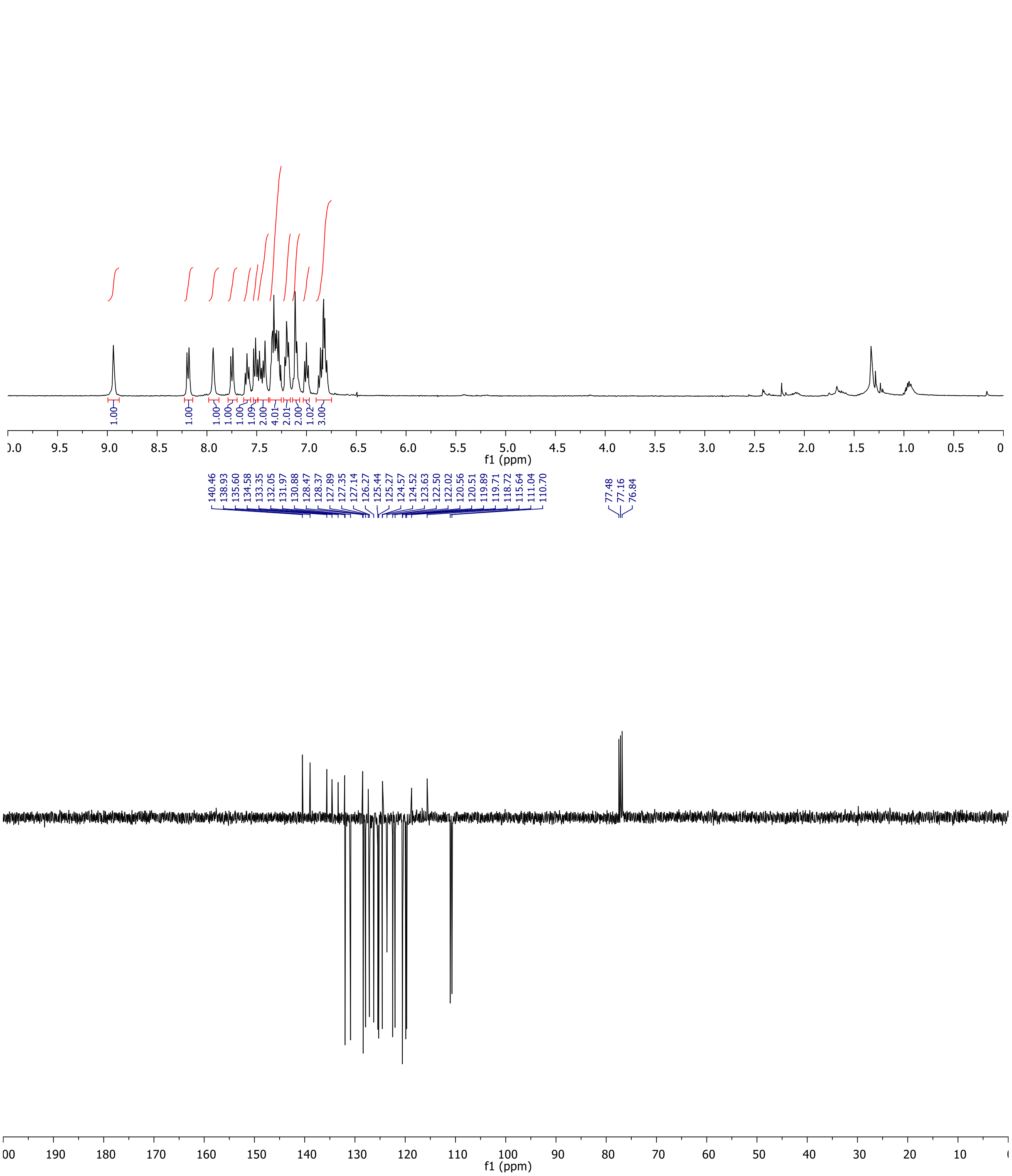

^{1}H NMR (400 MHz) and ^{13}C{^{1}H} (APT) NMR (100 MHz) spectra of **4i** ($CDCl_3$)

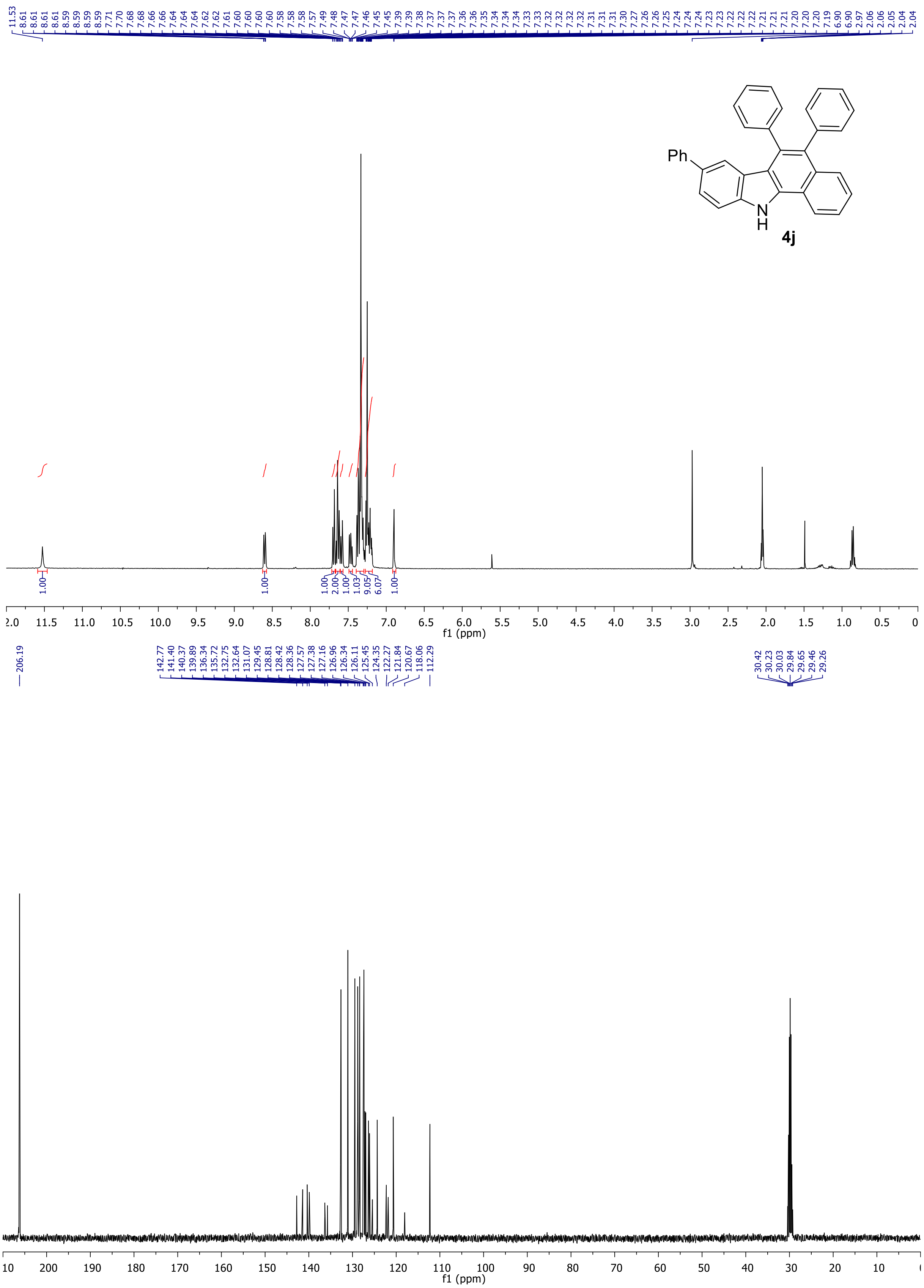

^{1}H NMR (400 MHz) and $^{13}C\{^{1}H\}$ NMR (100 MHz) spectra of **4j** (Acetone-d_6)

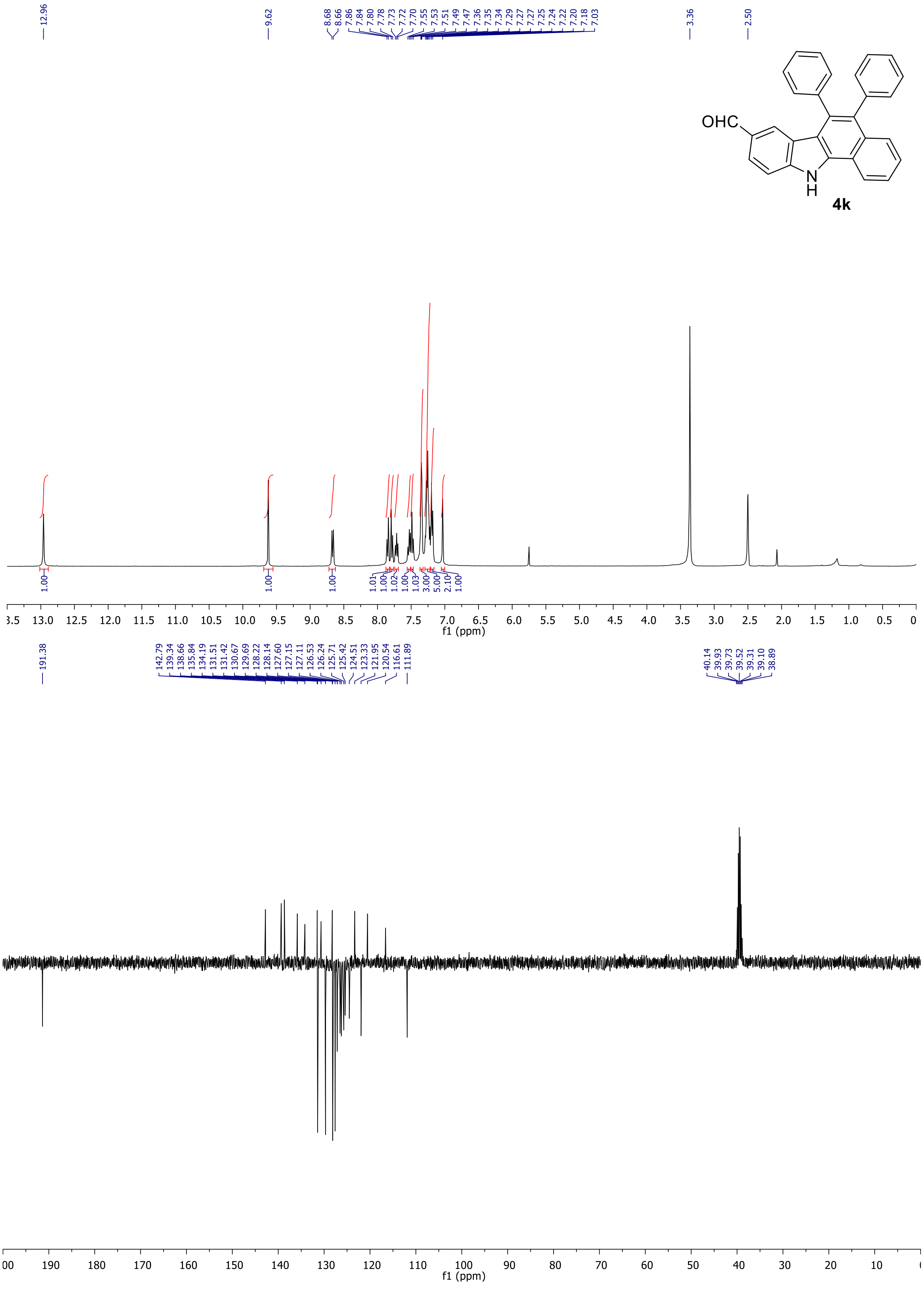

^{1}H NMR (400 MHz) and ^{13}C{^{1}H} (APT) NMR (100 MHz) spectra of **4k** (DMSO-d_6)

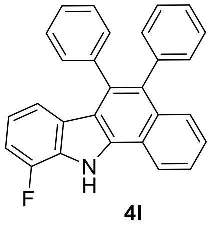

4l

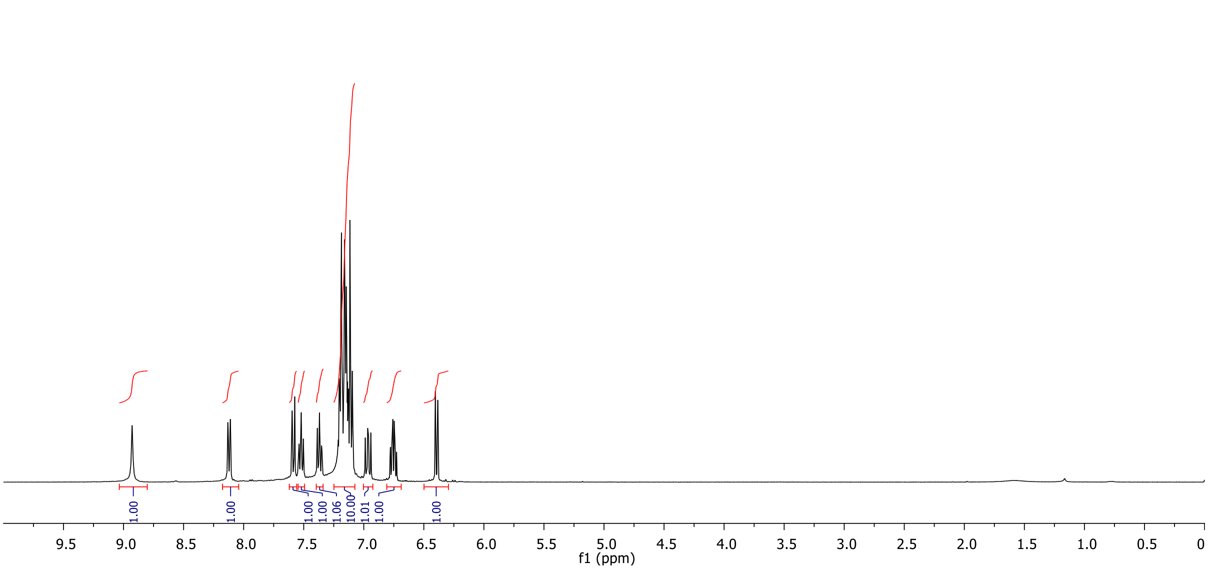

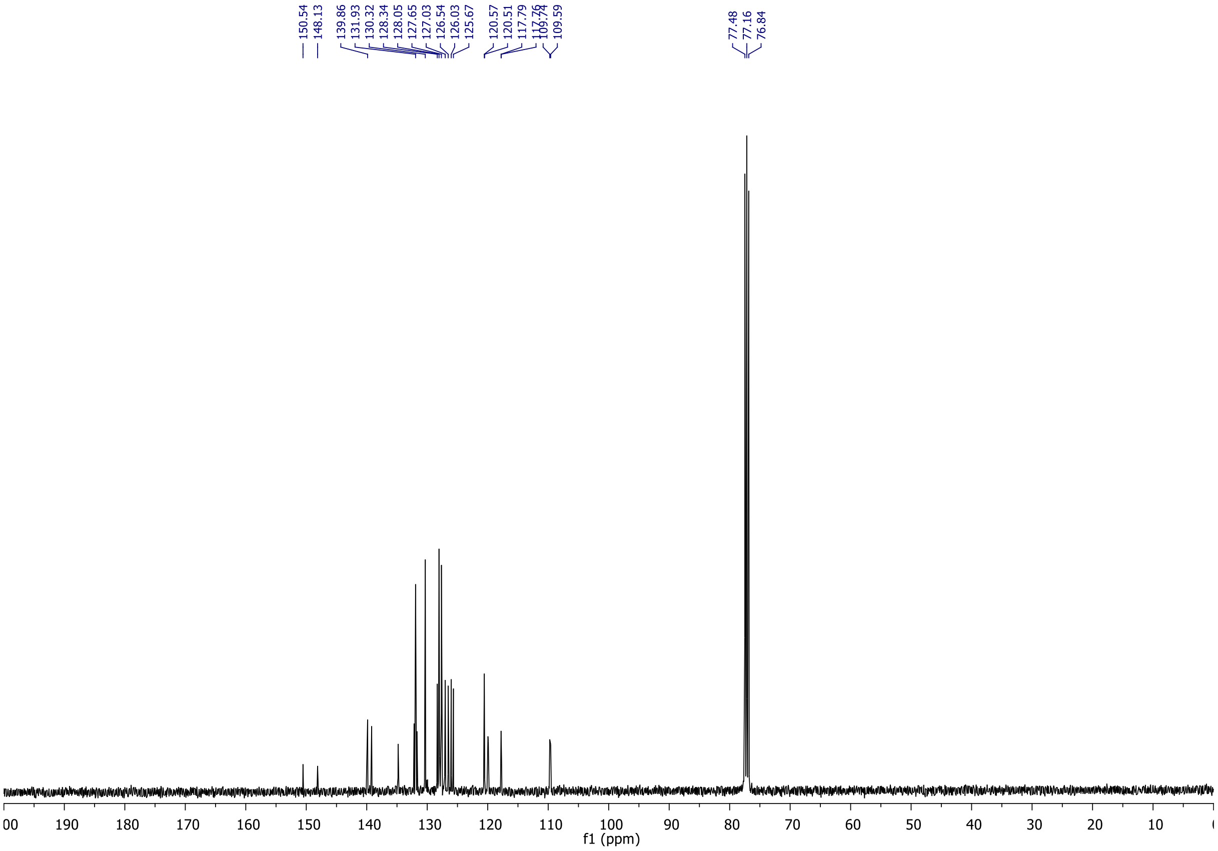

^{1}H NMR (400 MHz) and ^{13}C{^{1}H} NMR (100 MHz) spectra of **4l** ($CDCl_3$)

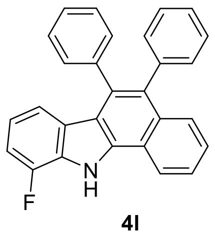

4l

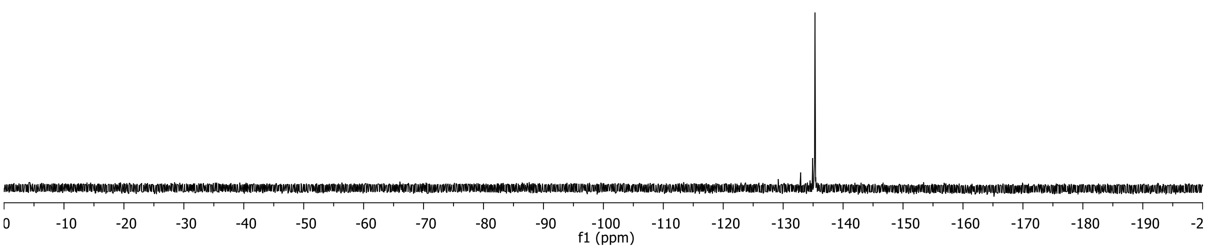

^{19}F NMR (376 MHz) spectra of **4l** ($CDCl_3$)

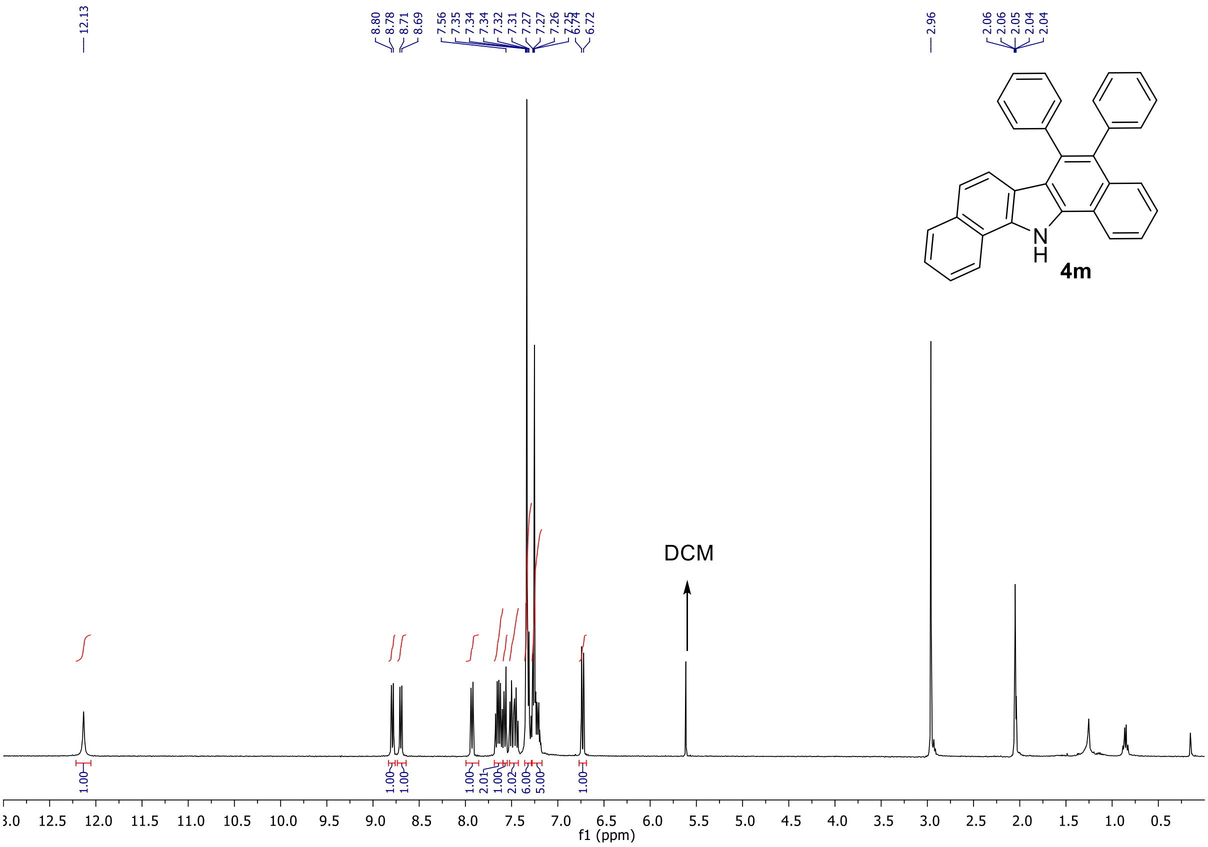

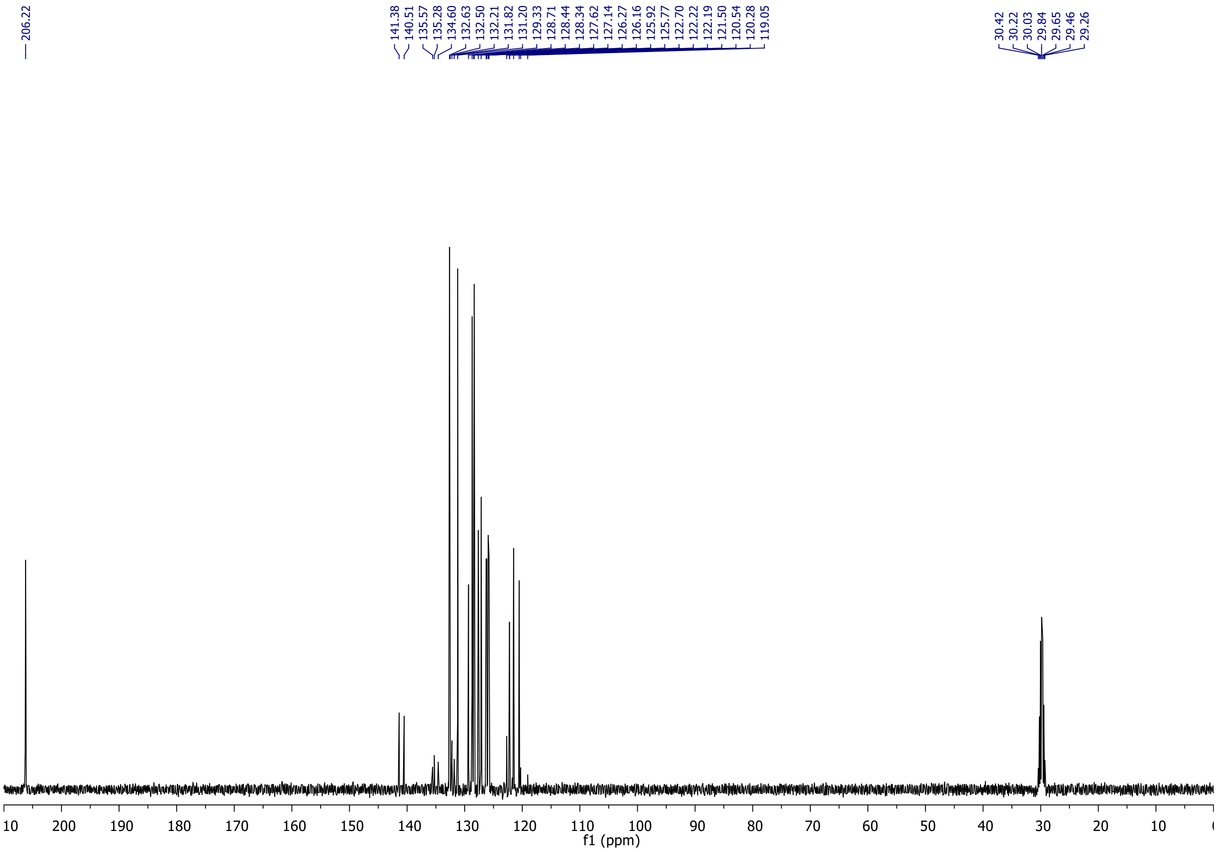

^{1}H NMR (400 MHz) and $^{13}C\{^{1}H\}$ NMR (100 MHz) spectra of **4m** (Acetone-d_6)

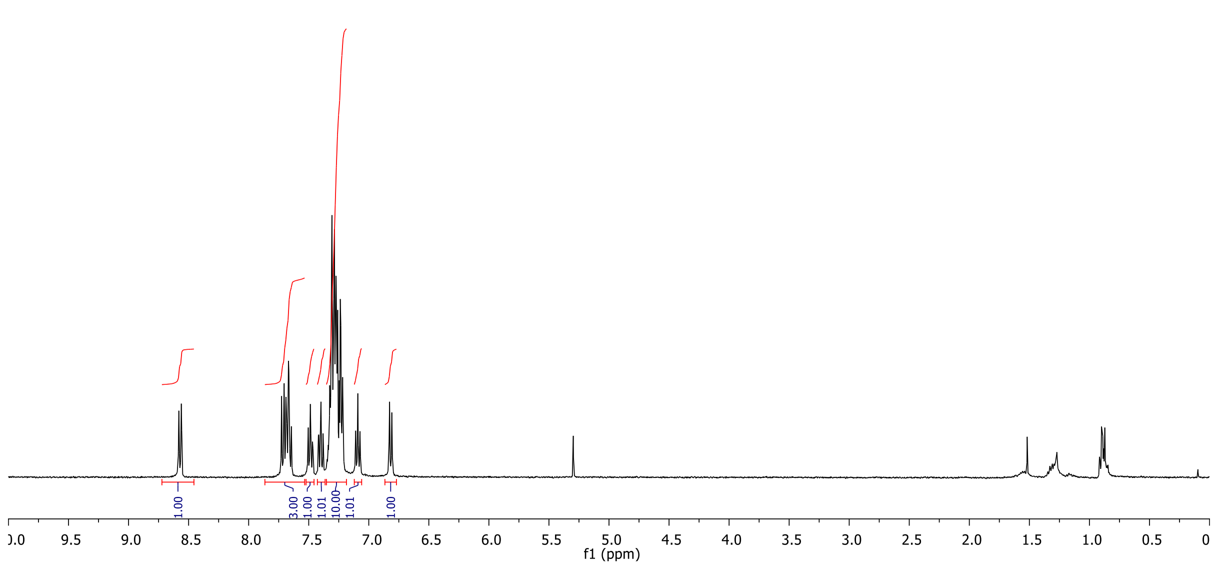

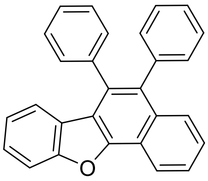

4n

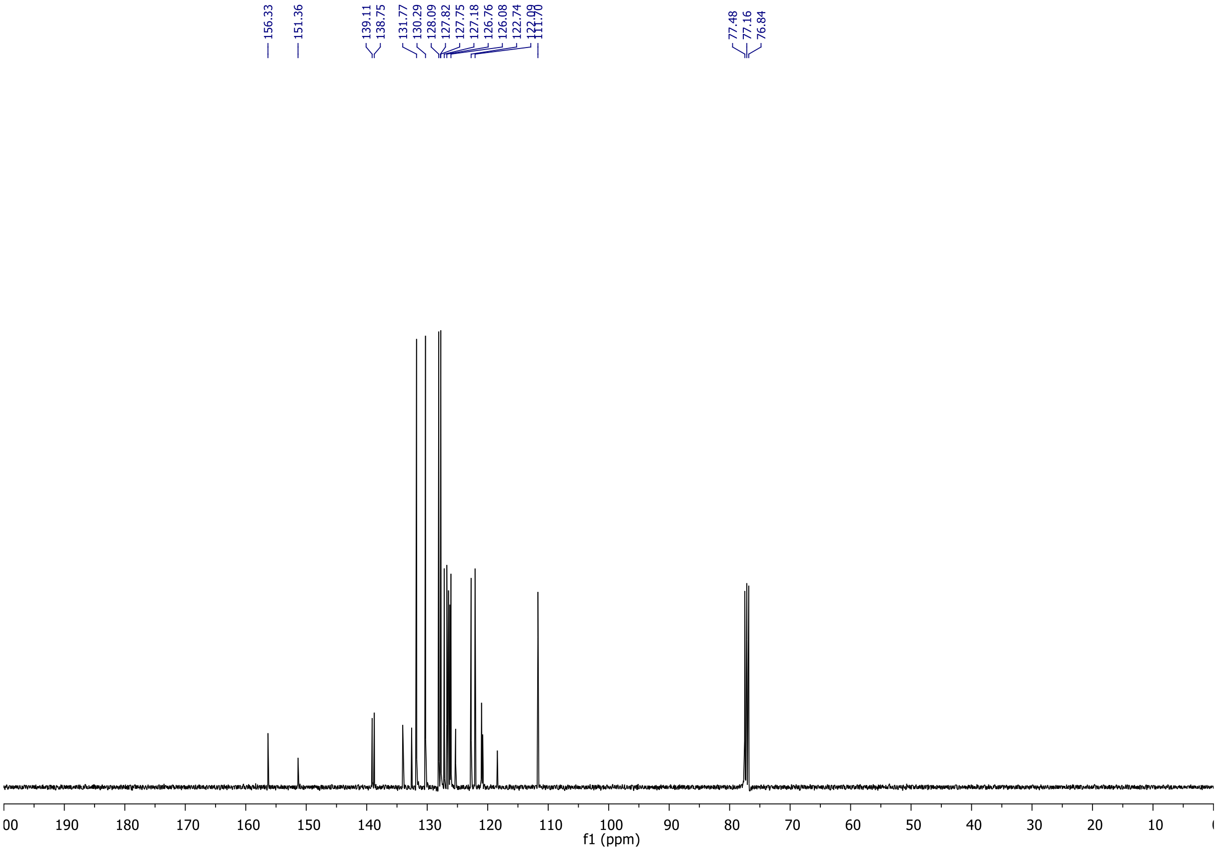

^{1}H NMR (400 MHz) and ^{13}C{^{1}H} NMR (100 MHz) spectra of **4n** ($CDCl_3$)

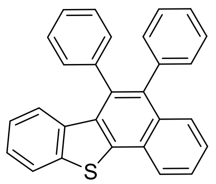

4o

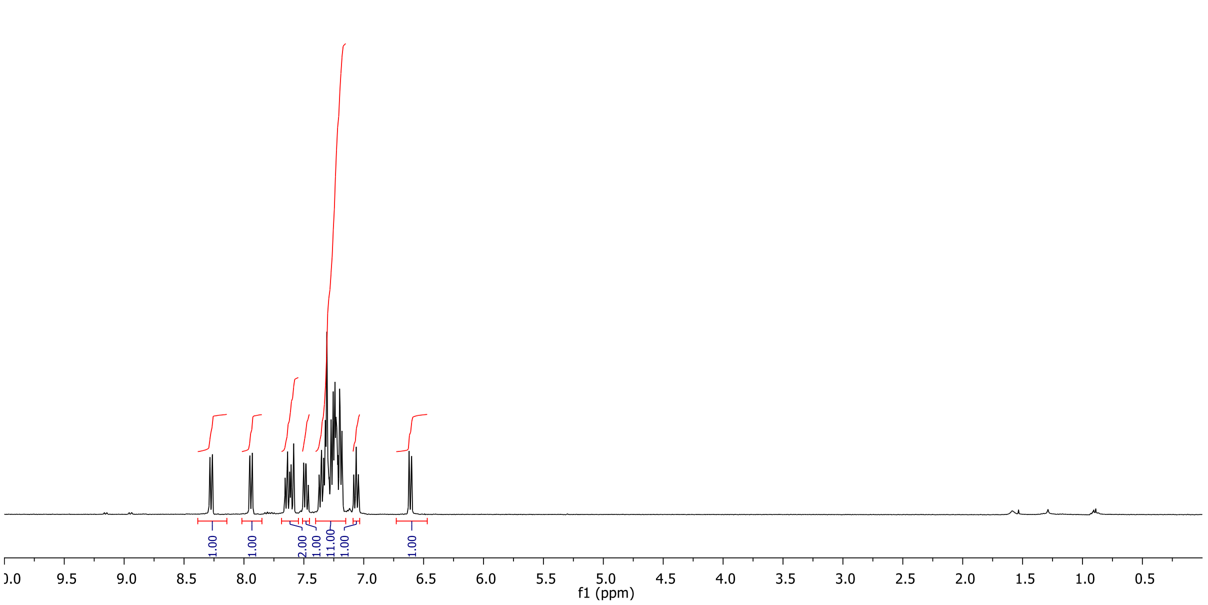

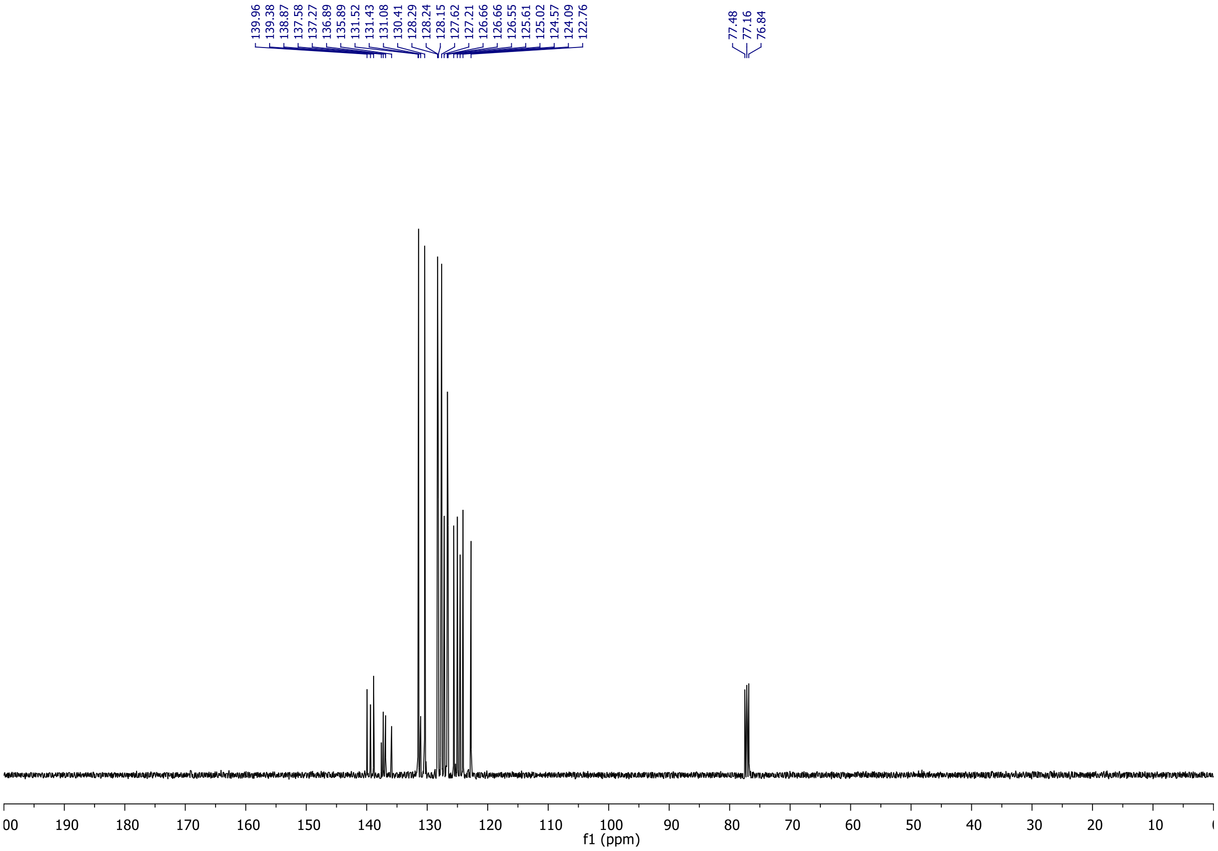

^{1}H NMR (400 MHz) and ^{13}C{^{1}H} NMR (100 MHz) spectra of **4o** ($CDCl_3$)

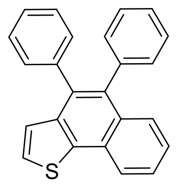

4p

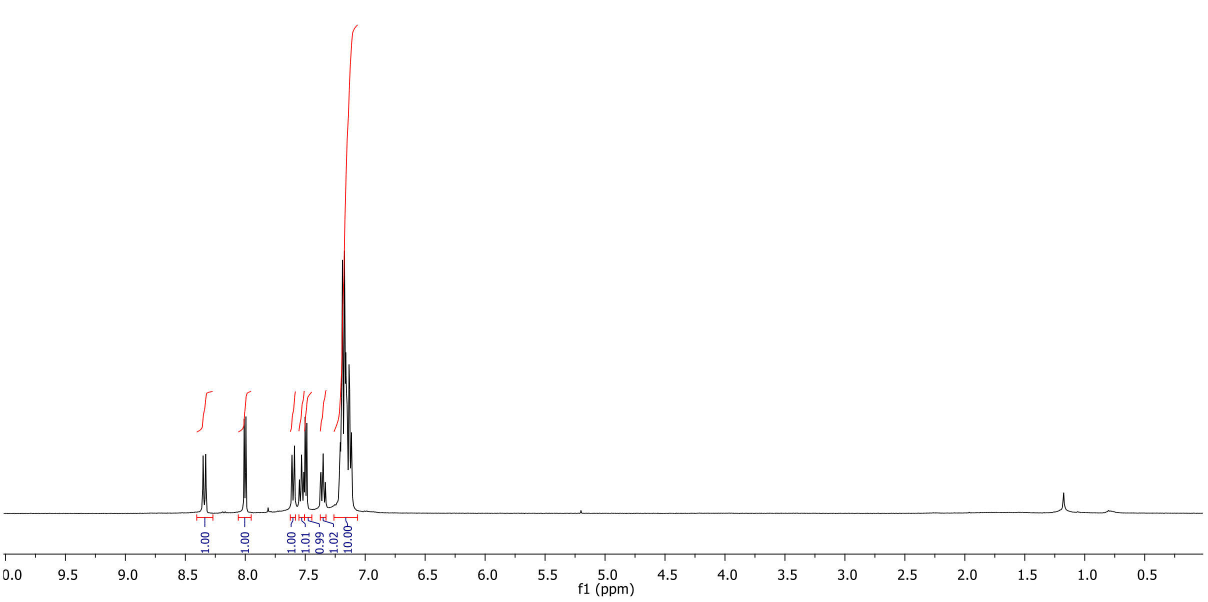

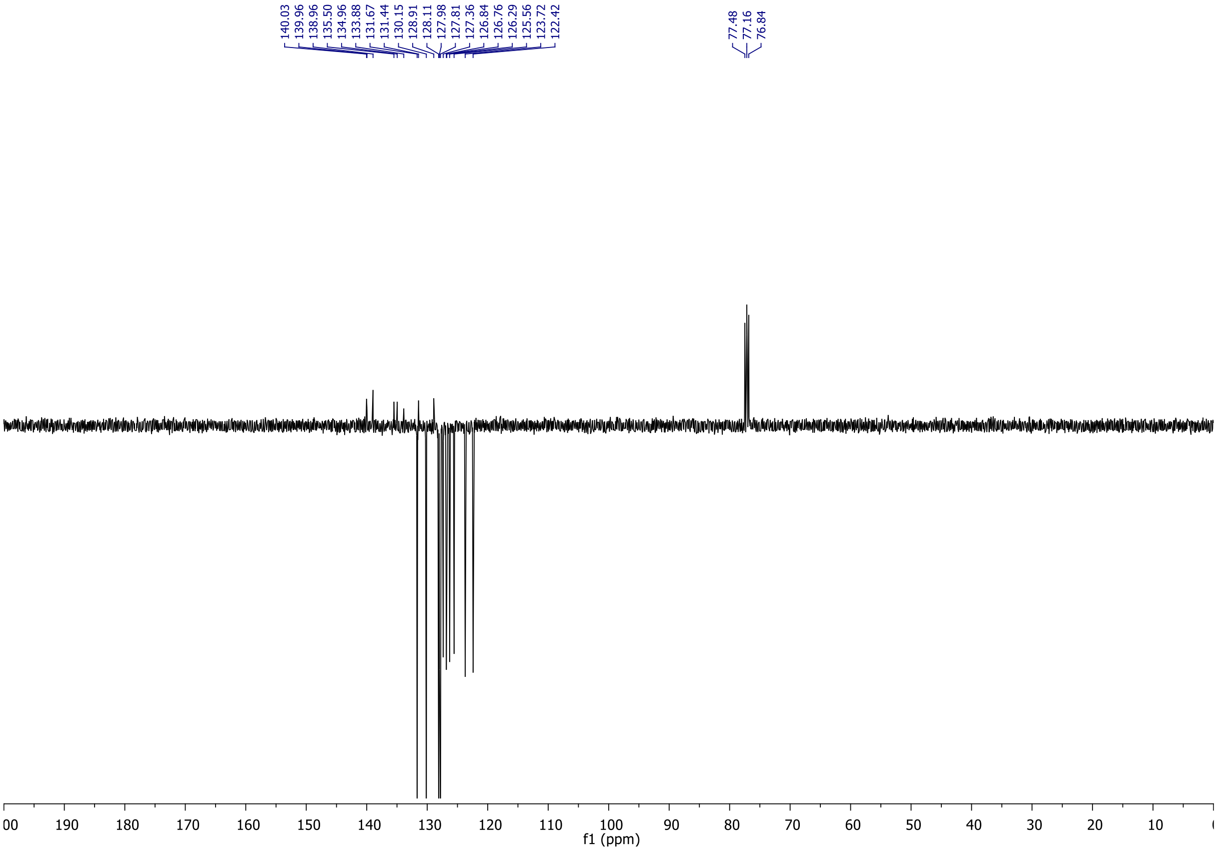

^{1}H NMR (400 MHz) and ^{13}C{^{1}H} (APT) NMR (100 MHz) spectra of **4p** ($CDCl_3$)

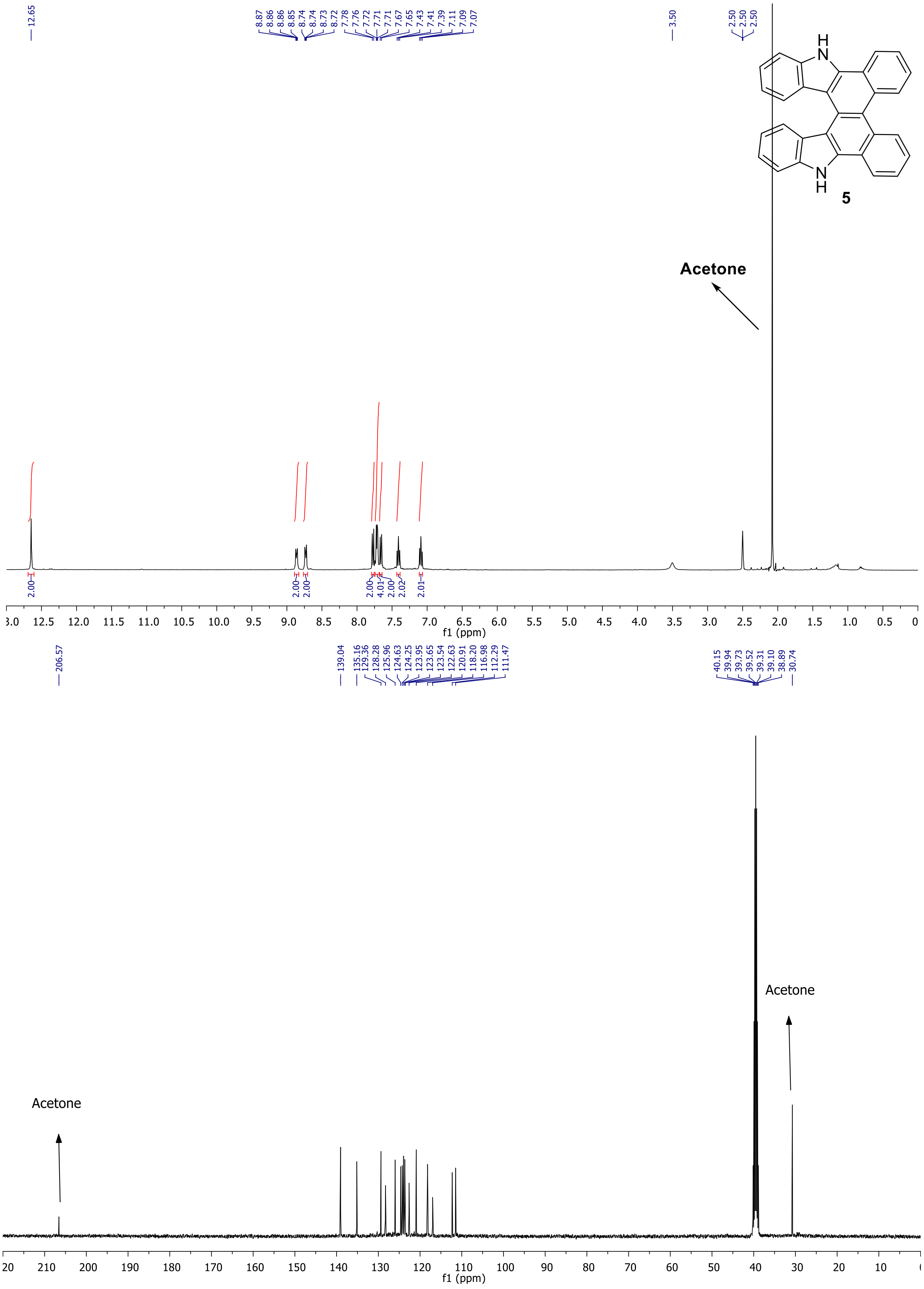

^{1}H NMR (400 MHz) and ^{13}C{^{1}H} NMR (100 MHz) spectra of **5** (DMSO-d_6)

5. Mass Spectrum

HRMS spectrum of S10

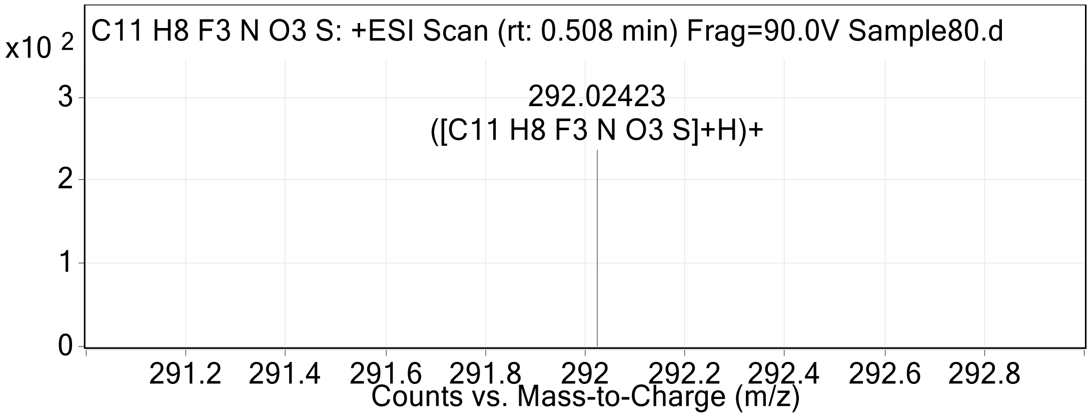

HRMS spectrum of S11

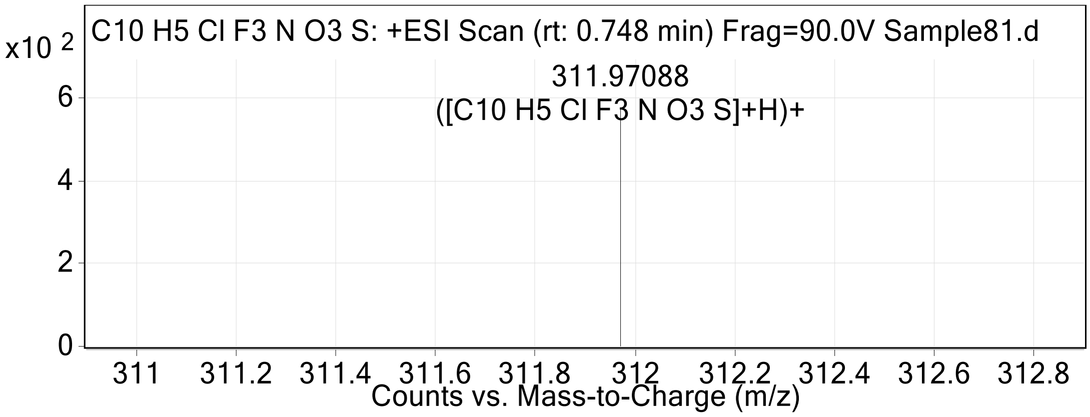

HRMS spectrum of S12

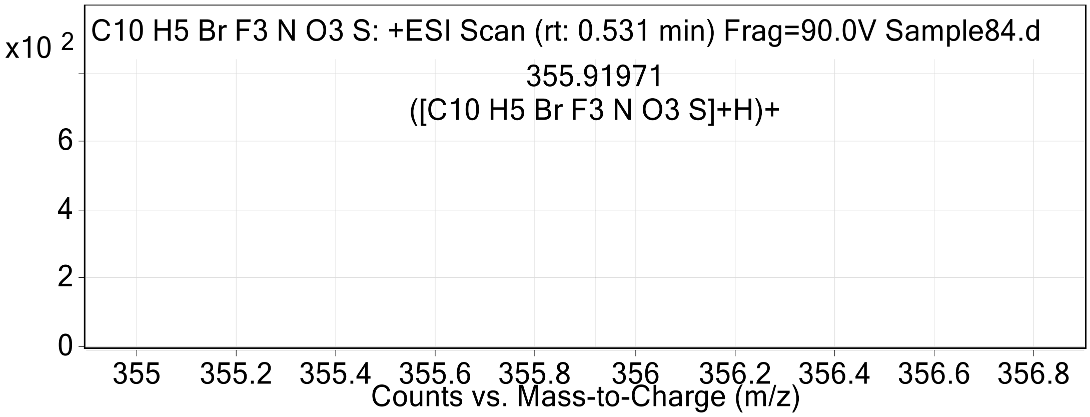

HRMS spectrum of S13

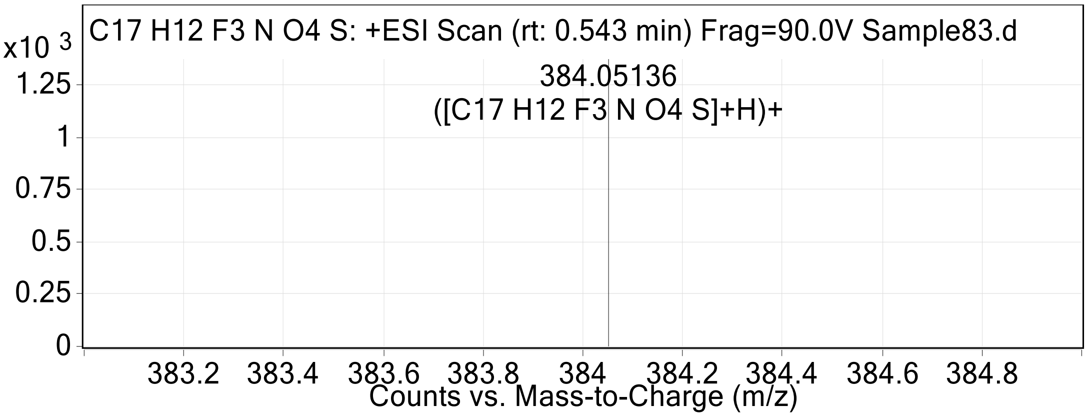

HRMS spectrum of S14

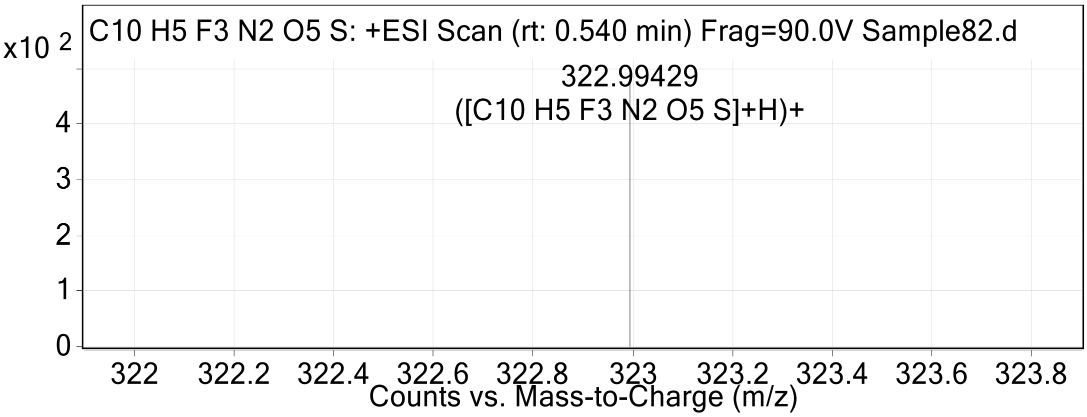

HRMS spectrum of S15

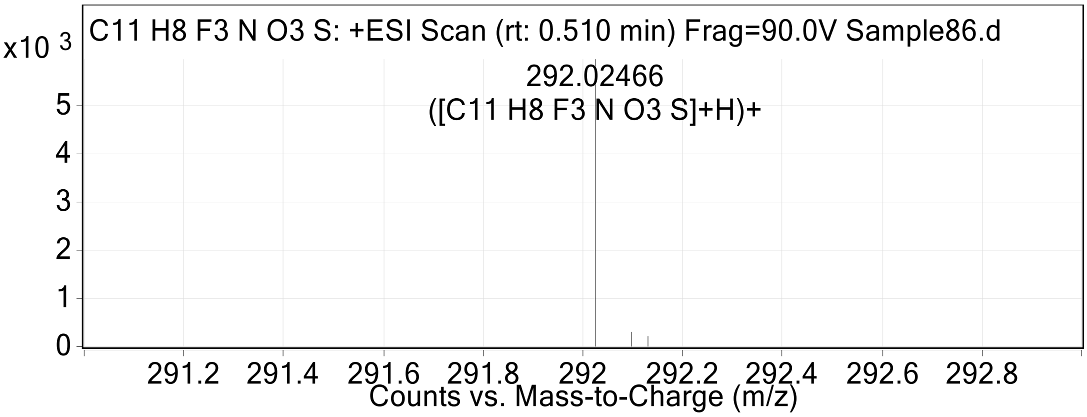

HRMS spectrum of S16

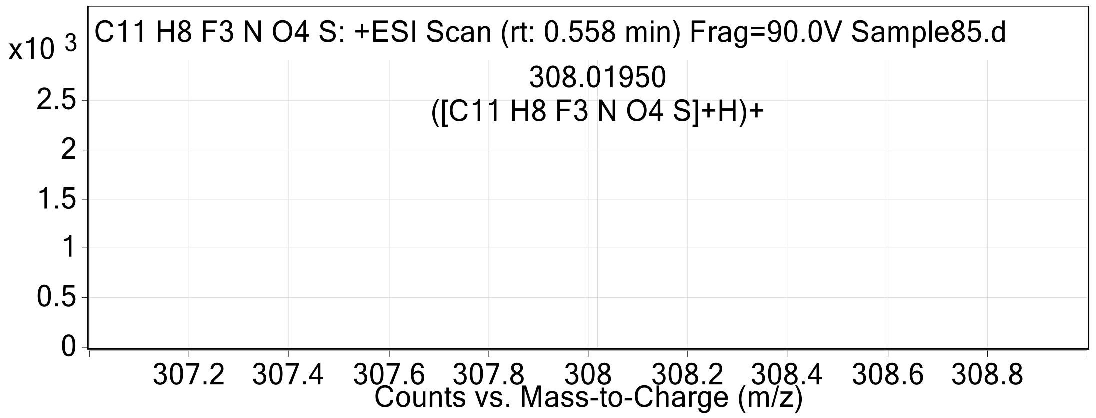

HRMS spectrum of S17

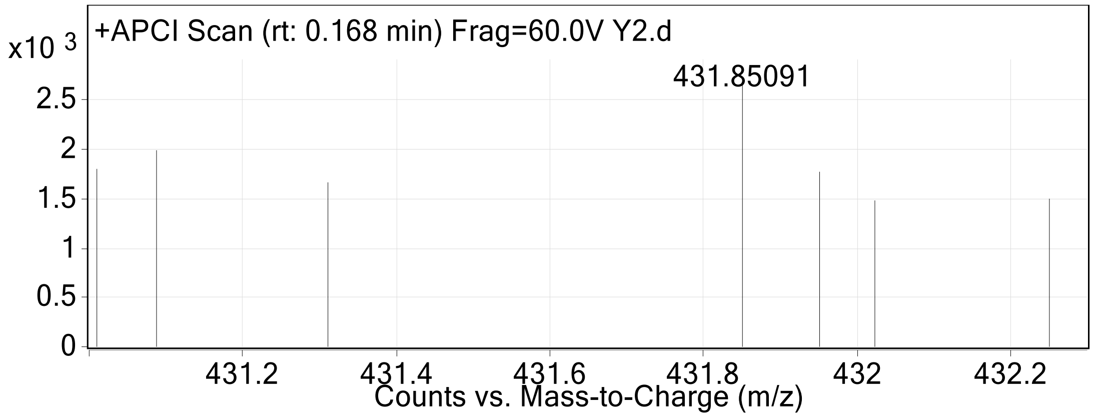

HRMS spectrum of S18

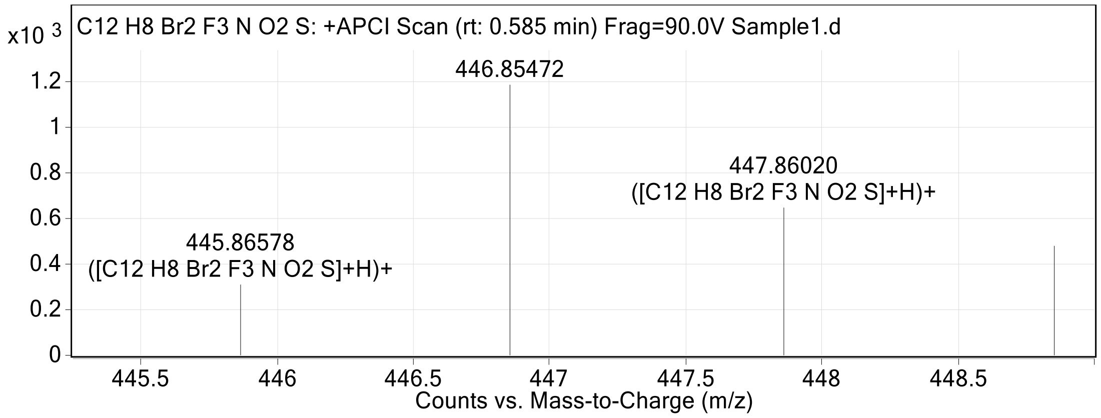

HRMS spectrum of S19

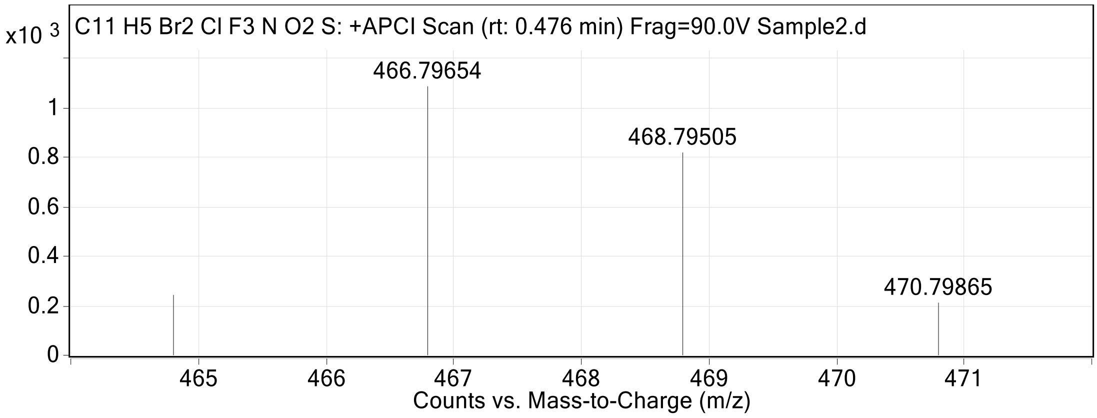

HRMS spectrum of S20

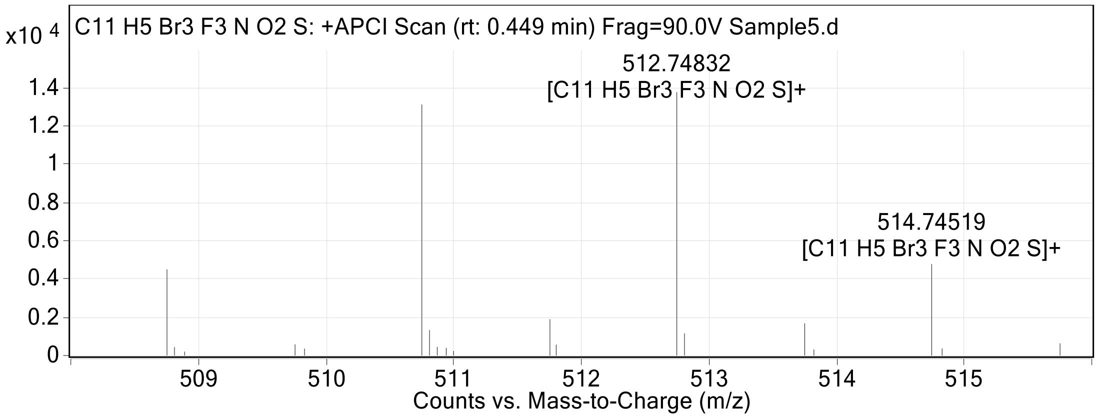

HRMS spectrum of S21

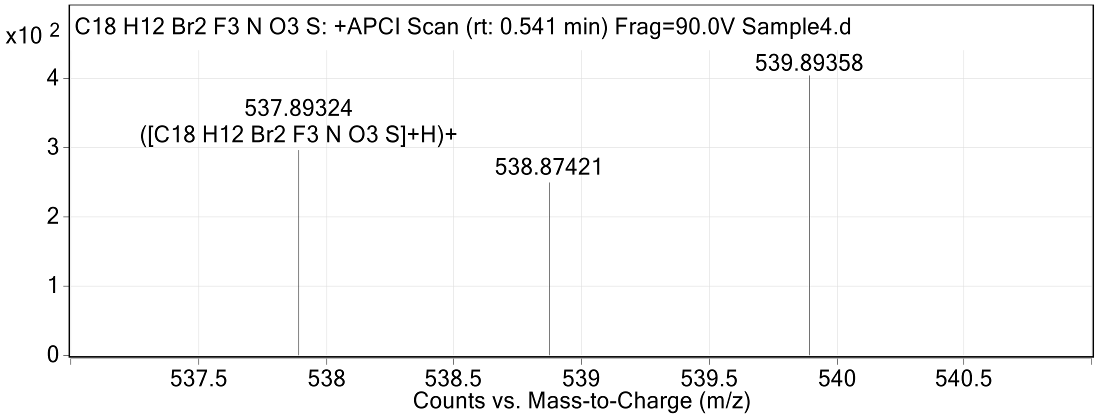

HRMS spectrum of S22

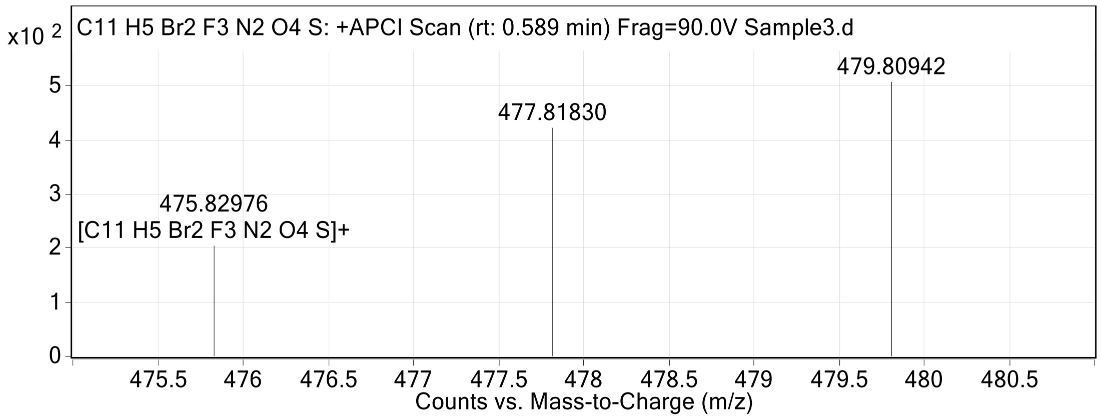

HRMS spectrum of S23

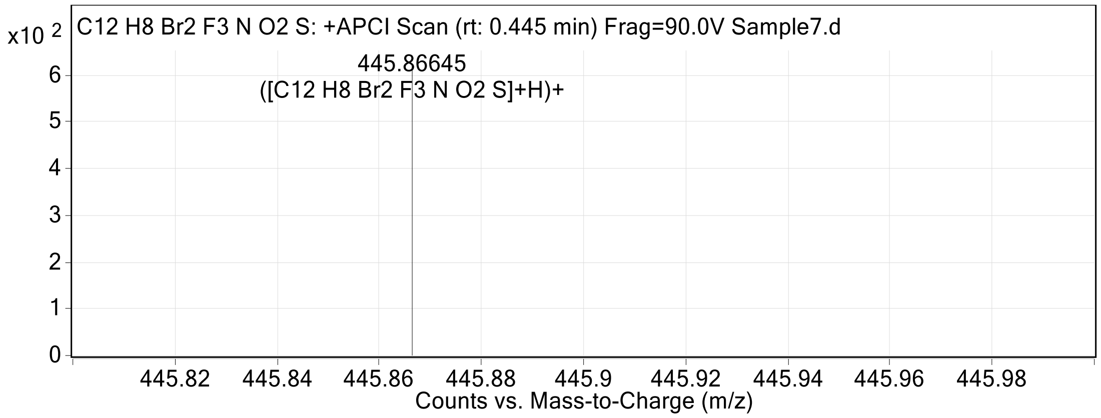

HRMS spectrum of S24

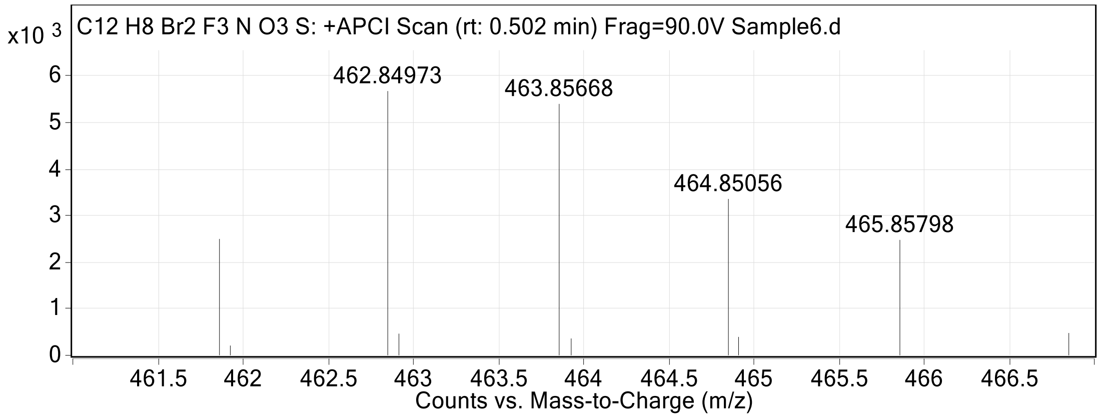

HRMS spectrum of 1a

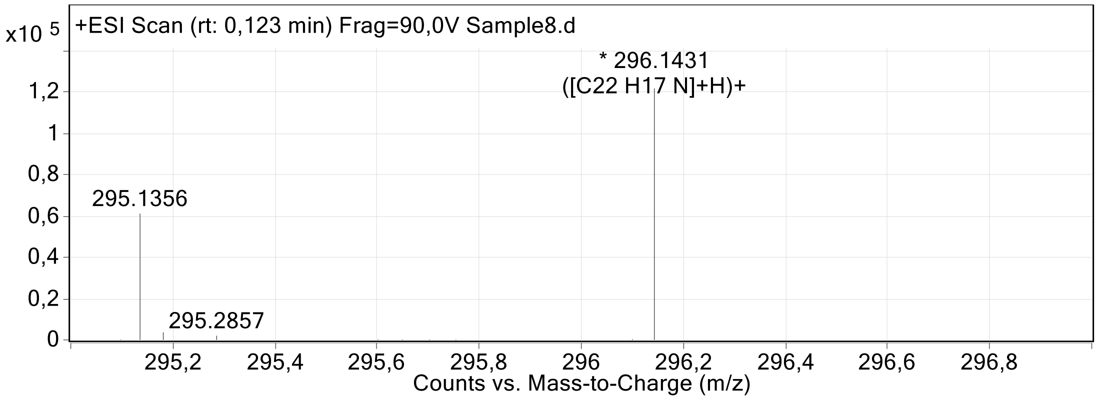

HRMS spectrum of 1b

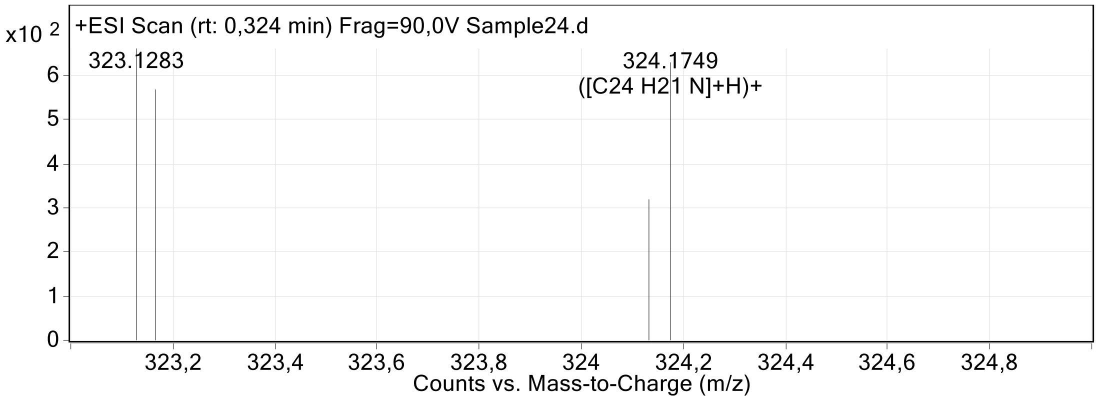

HRMS spectrum of 1c

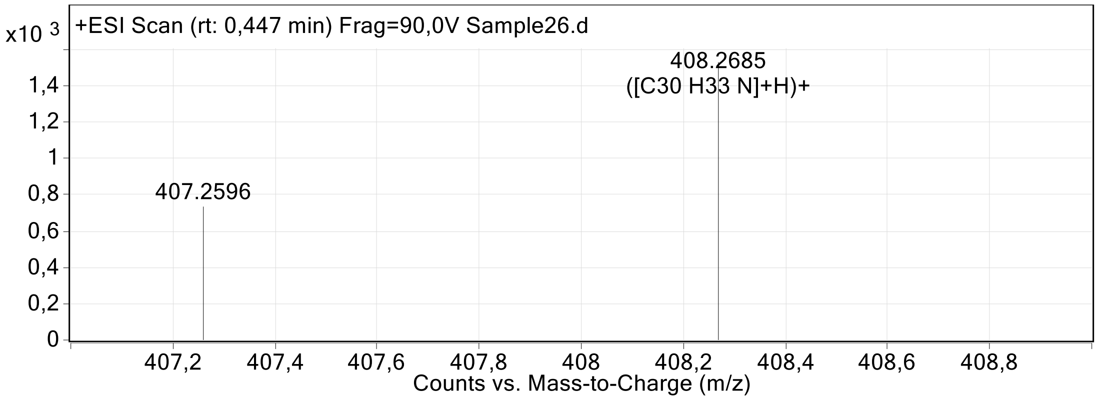

HRMS spectrum of 1d

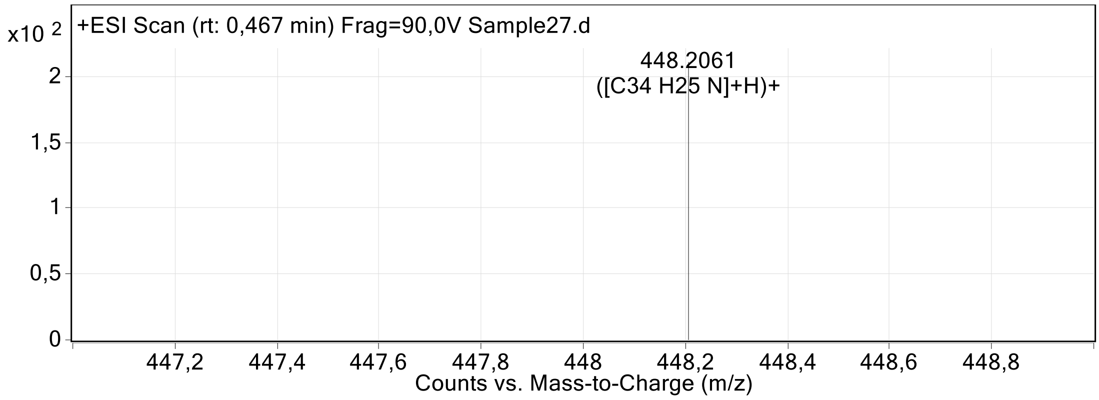

HRMS spectrum of 1e

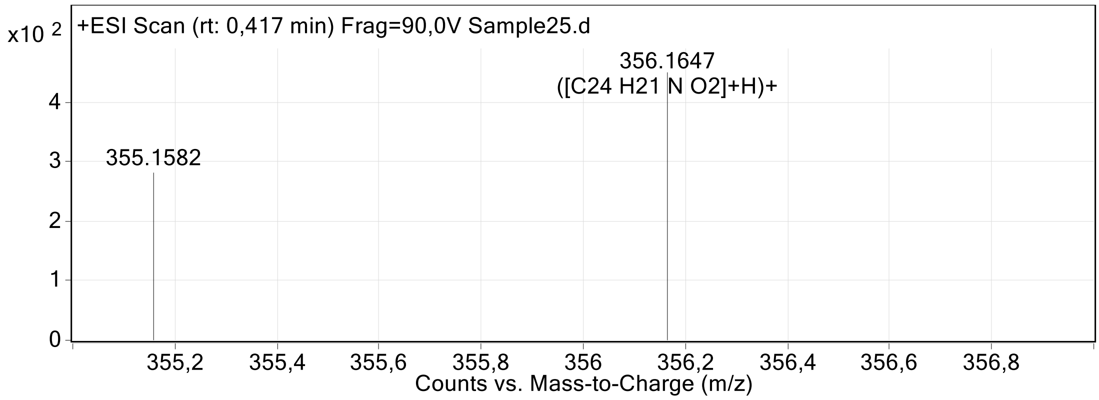

HRMS spectrum of 1f

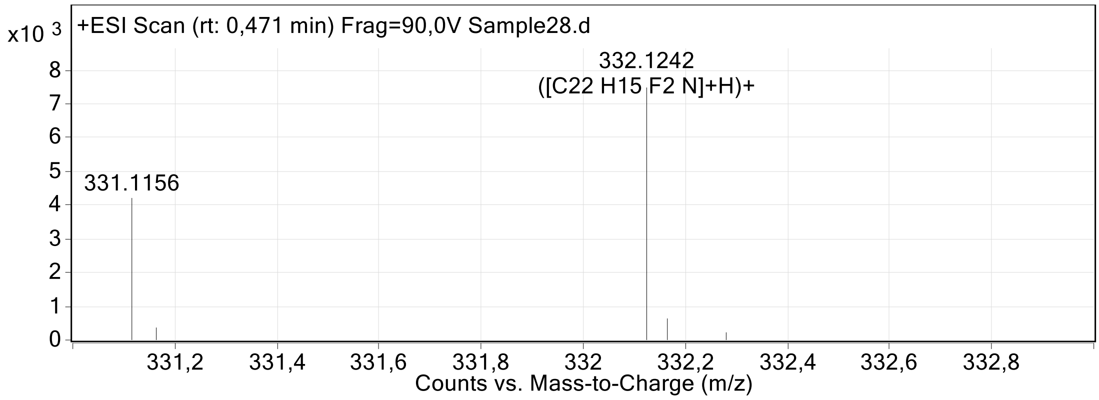

HRMS spectrum of 1g

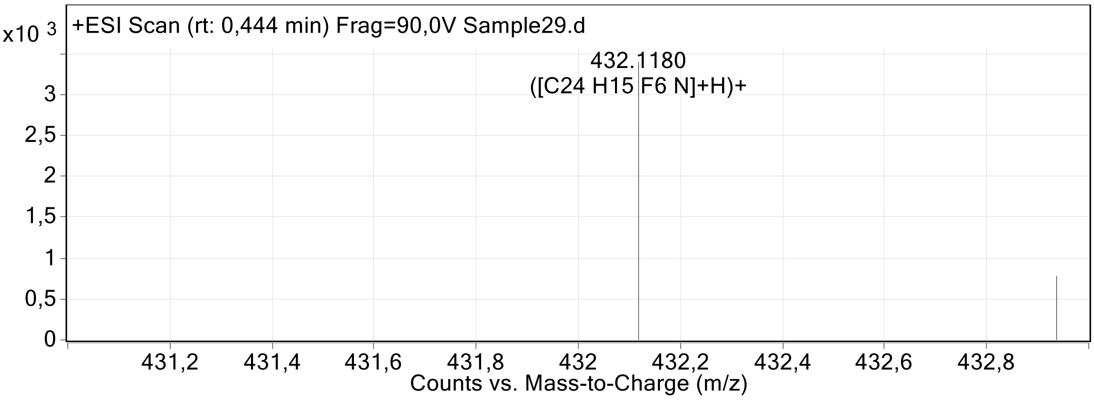

HRMS spectrum of 1h

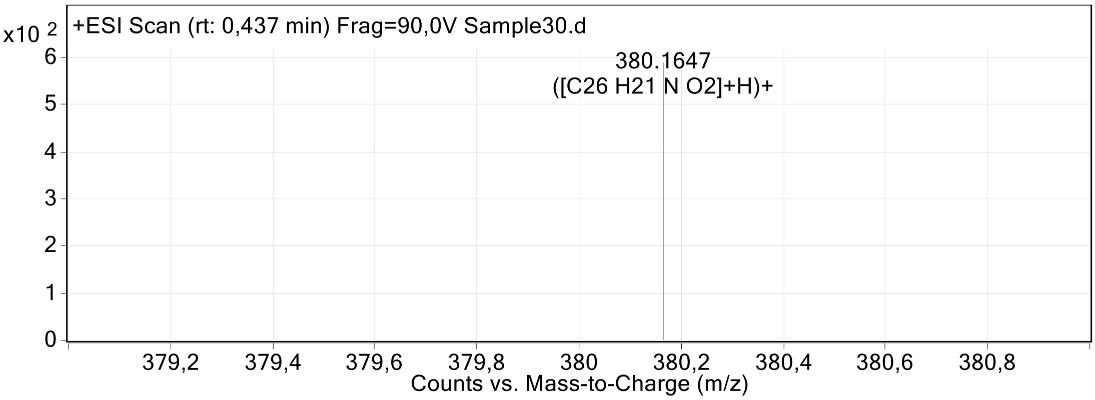

HRMS spectrum of 1i

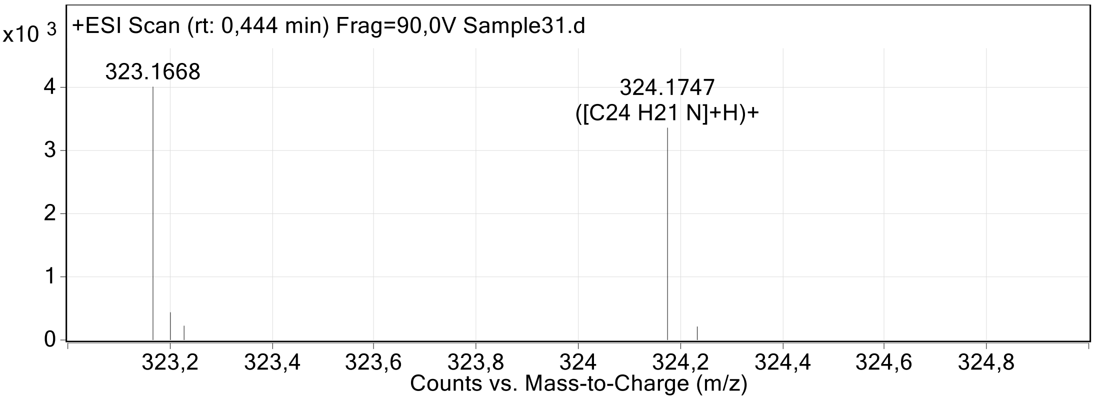

HRMS spectrum of 1j

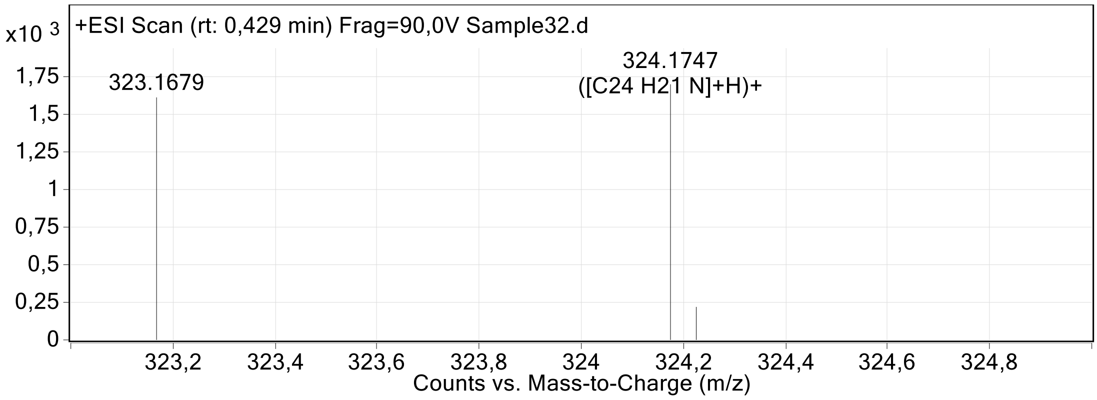

HRMS spectrum of 1k

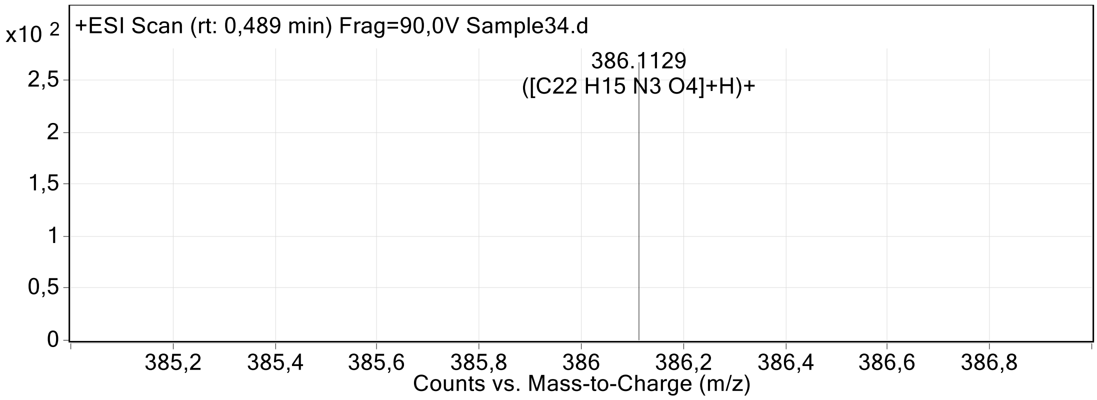

HRMS spectrum of 1l

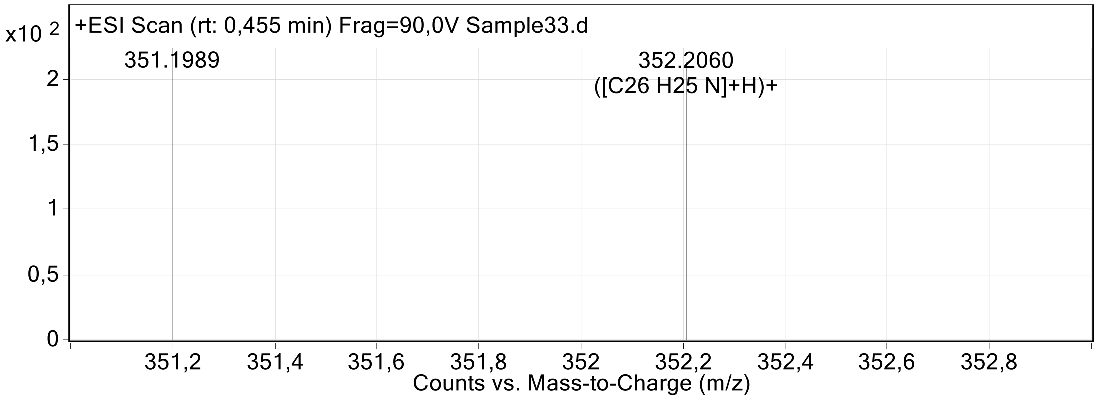

HRMS spectrum of 1m

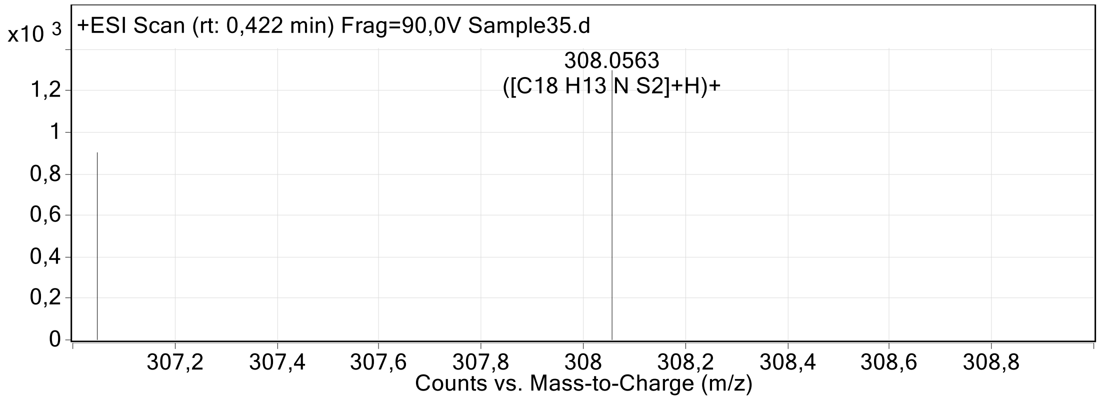

HRMS spectrum of 1n

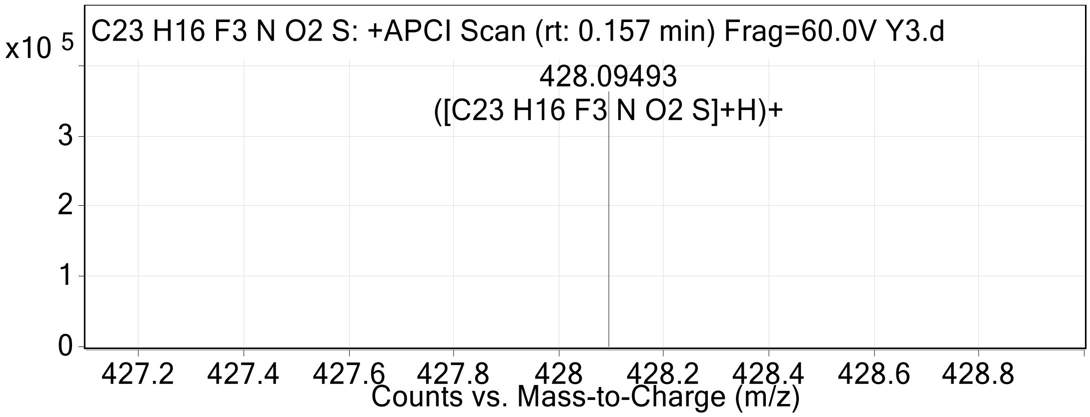

HRMS spectrum of 1o

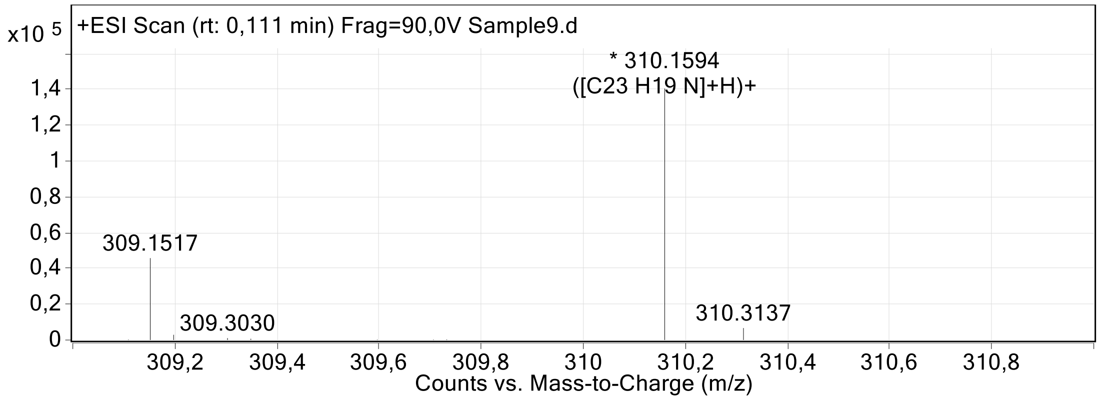

HRMS spectrum of 1p

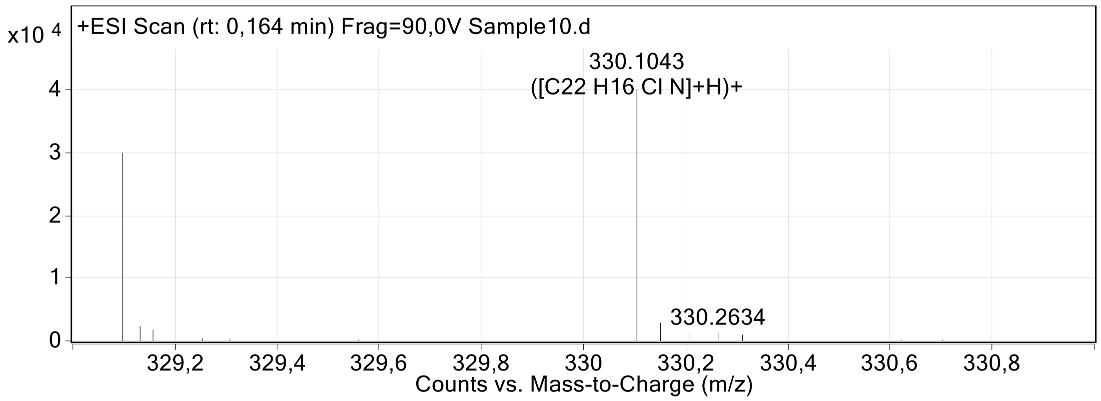

HRMS spectrum of 1q

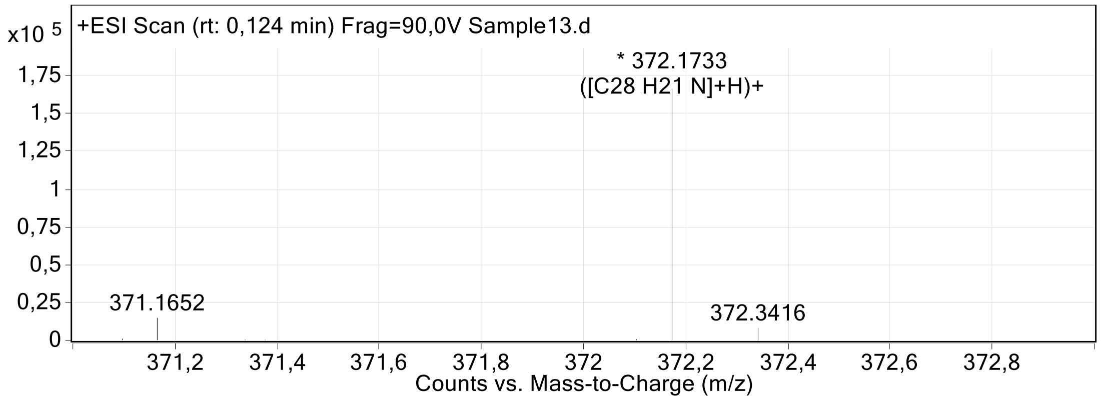

HRMS spectrum of 1r

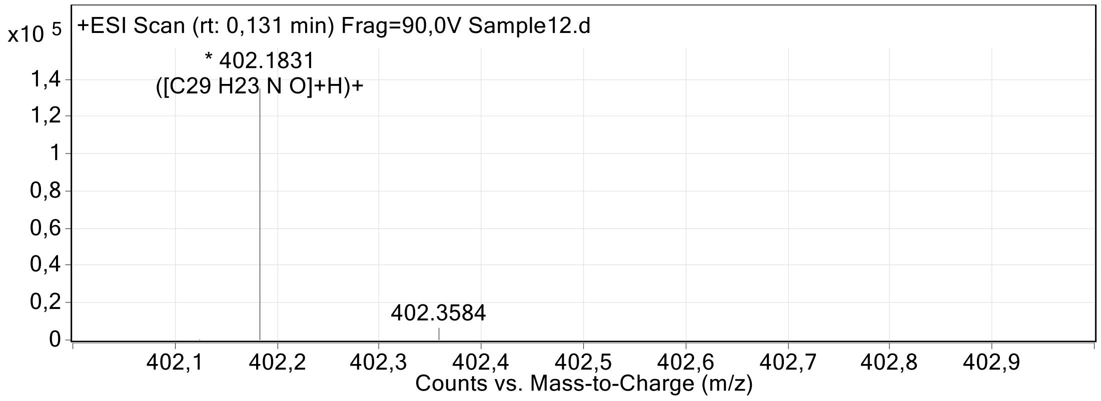

HRMS spectrum of 1s

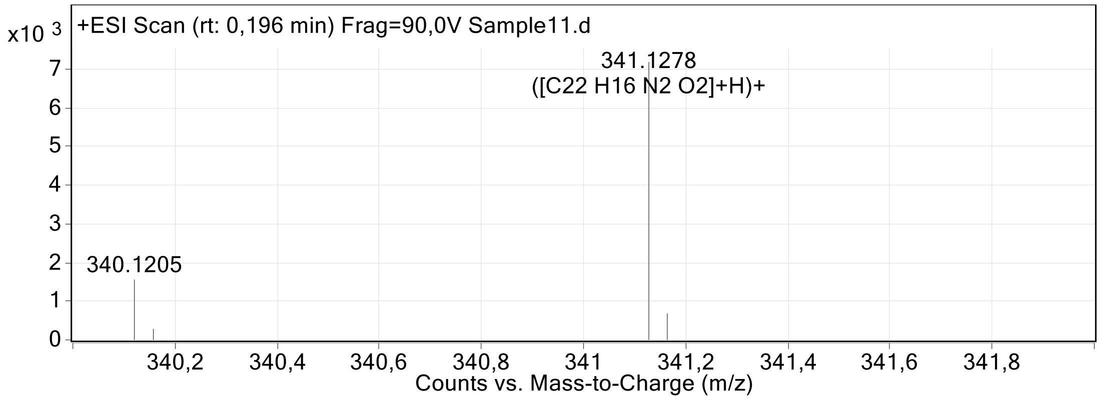

HRMS spectrum of 1t

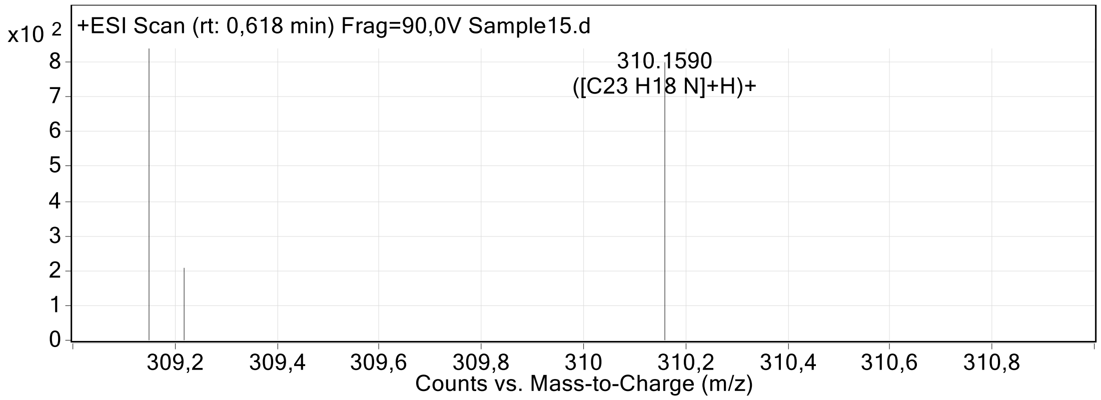

HRMS spectrum of 1u

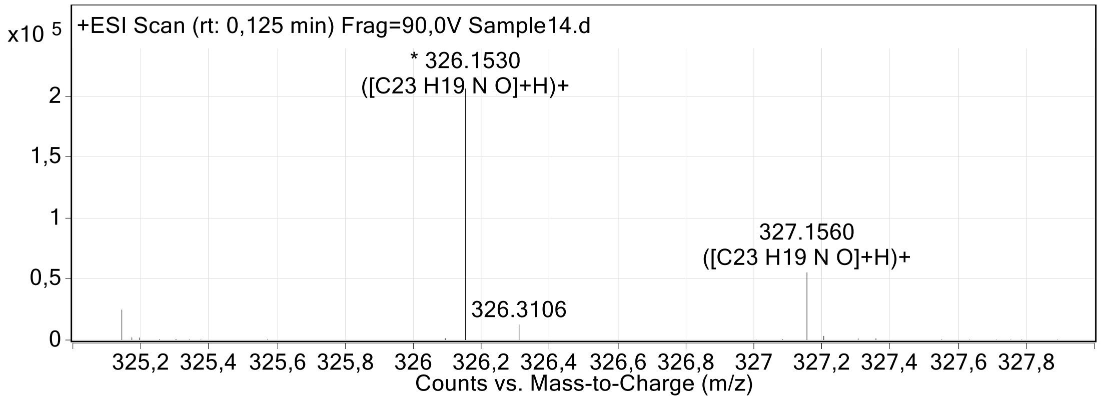

HRMS spectrum of S49

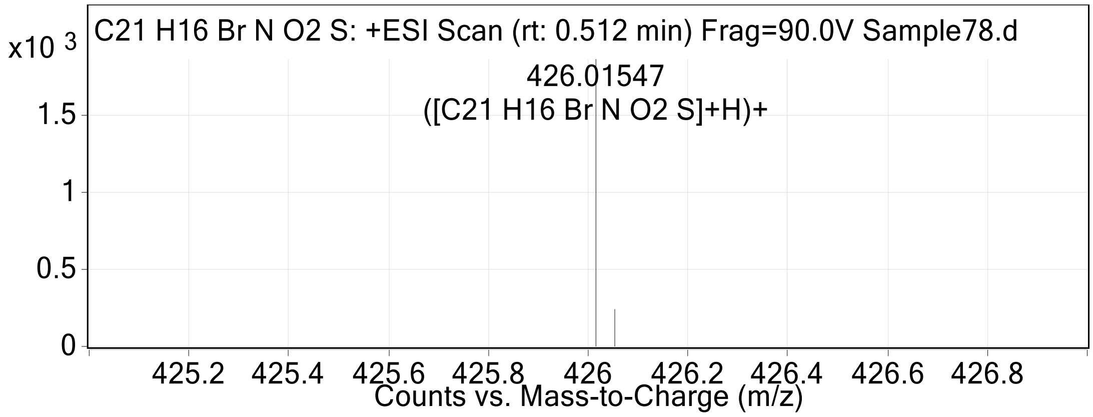

HRMS spectrum of S50

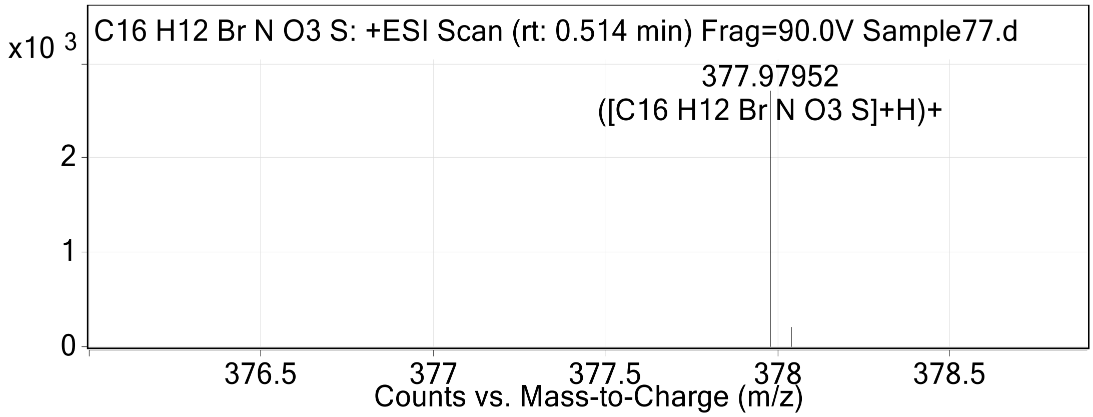

HRMS spectrum of S51

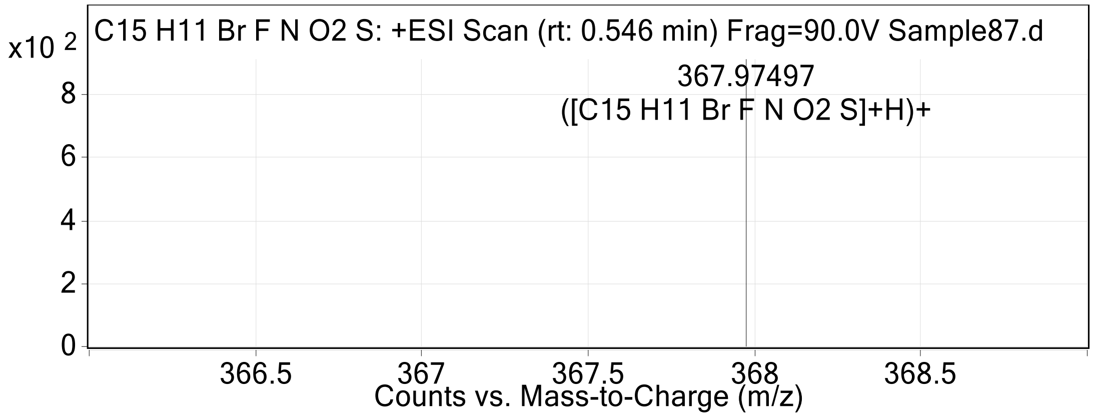

HRMS spectrum of S52

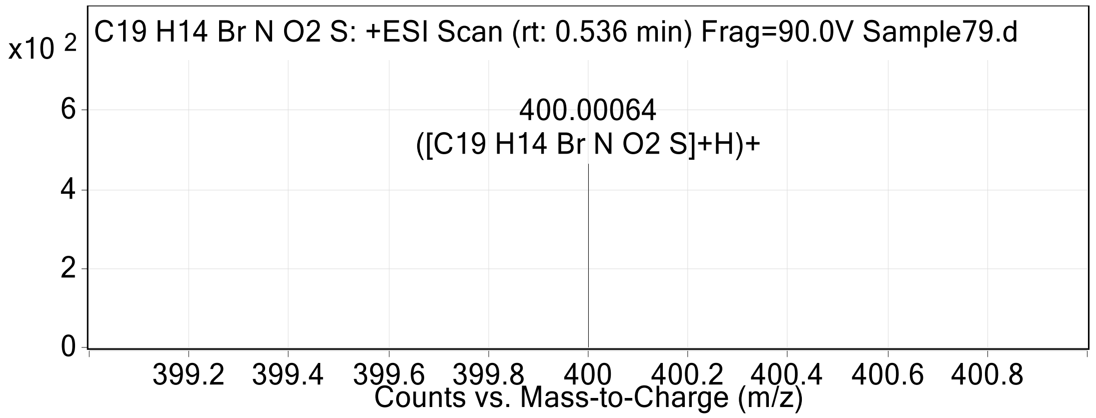

HRMS spectrum of S53

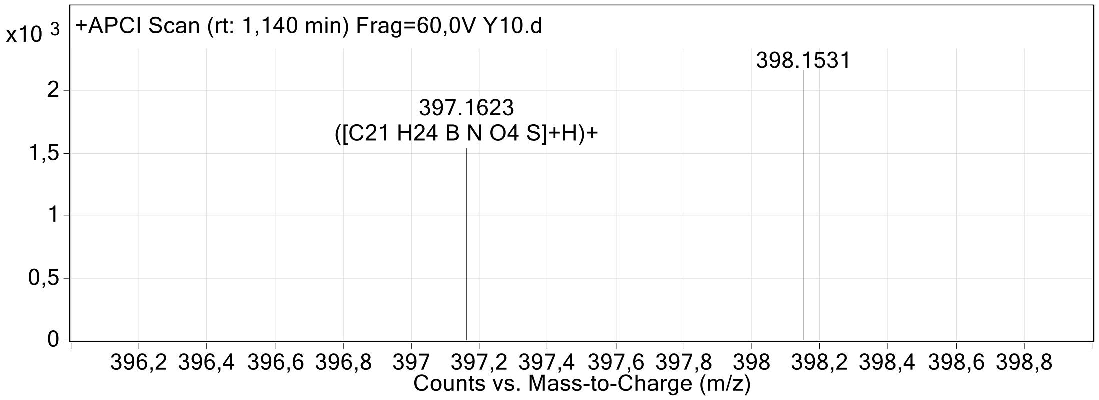

HRMS spectrum of S57

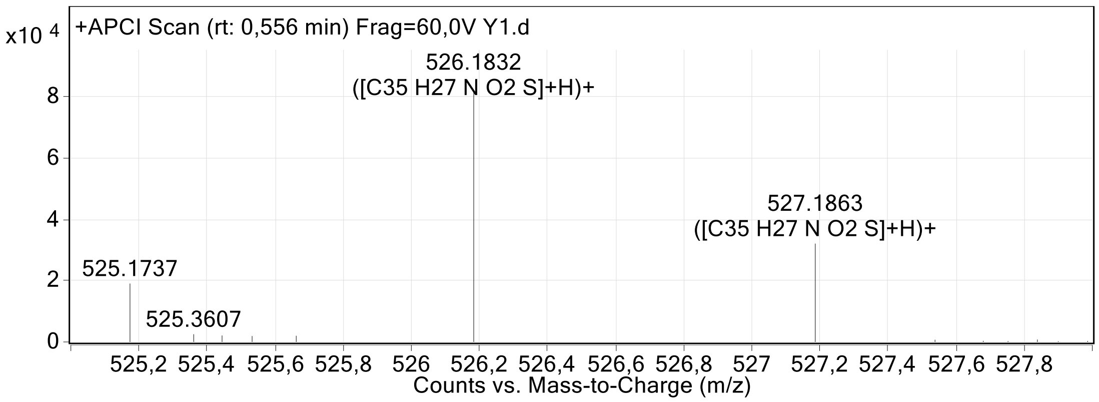

HRMS spectrum of S58

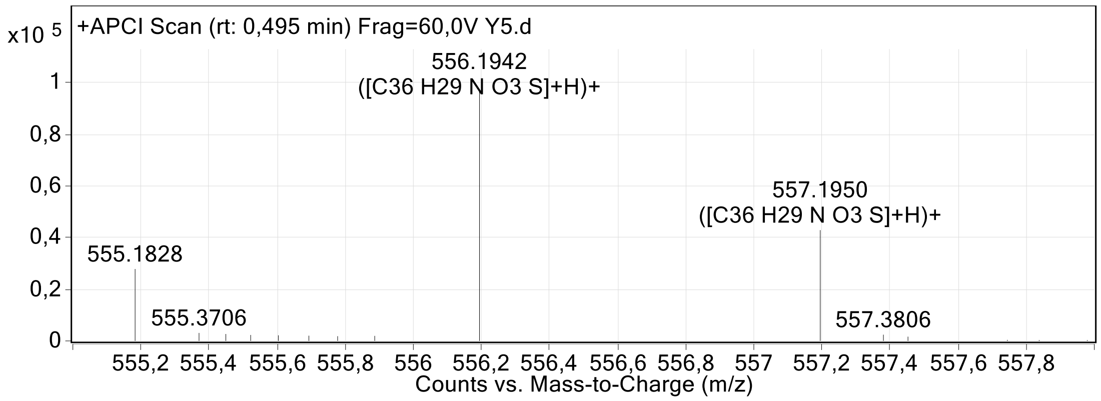

HRMS spectrum of S659

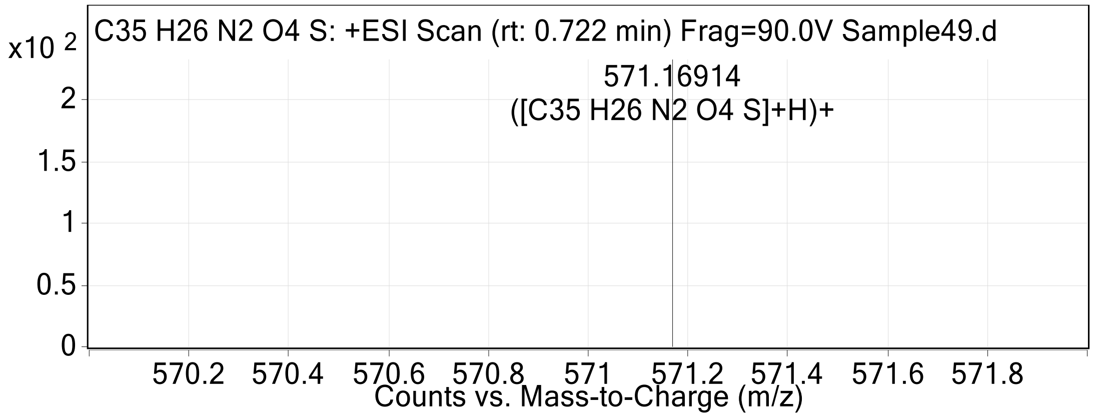

HRMS spectrum of S61

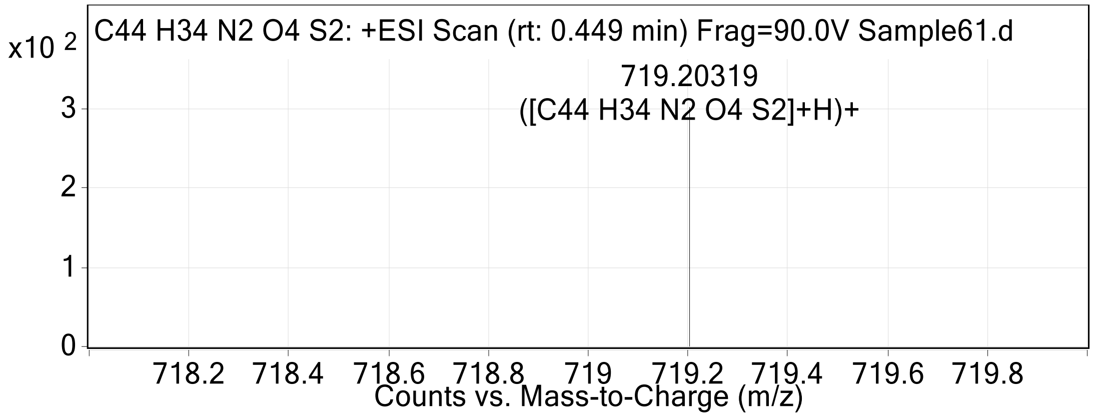

HRMS spectrum of S63

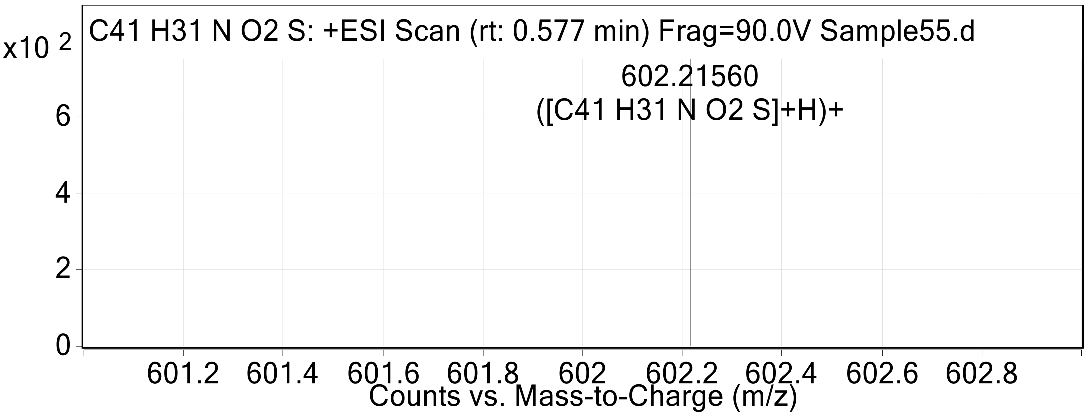

HRMS spectrum of S64

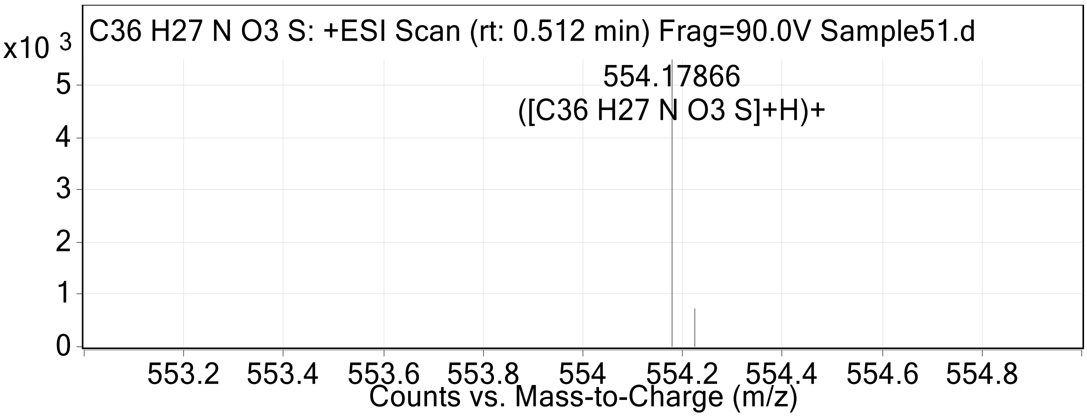

HRMS spectrum of S65

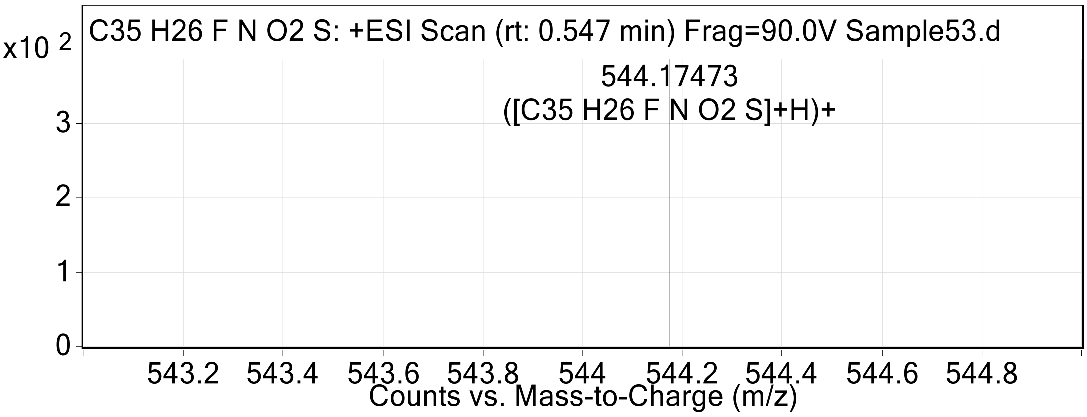

HRMS spectrum of S66

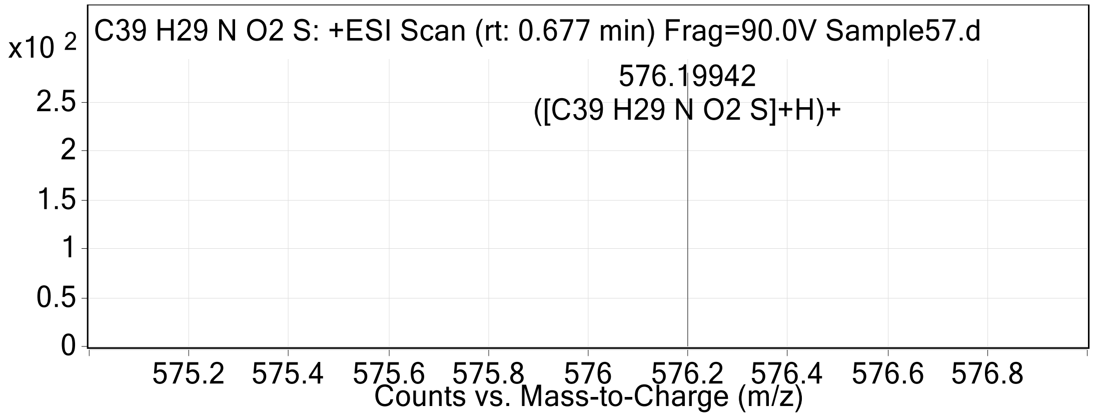

HRMS spectrum of 3a

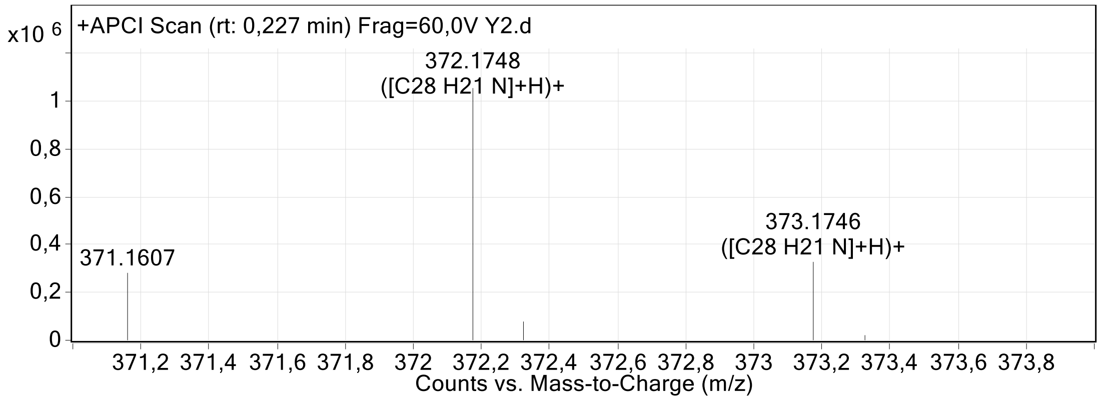

HRMS spectrum of 3d

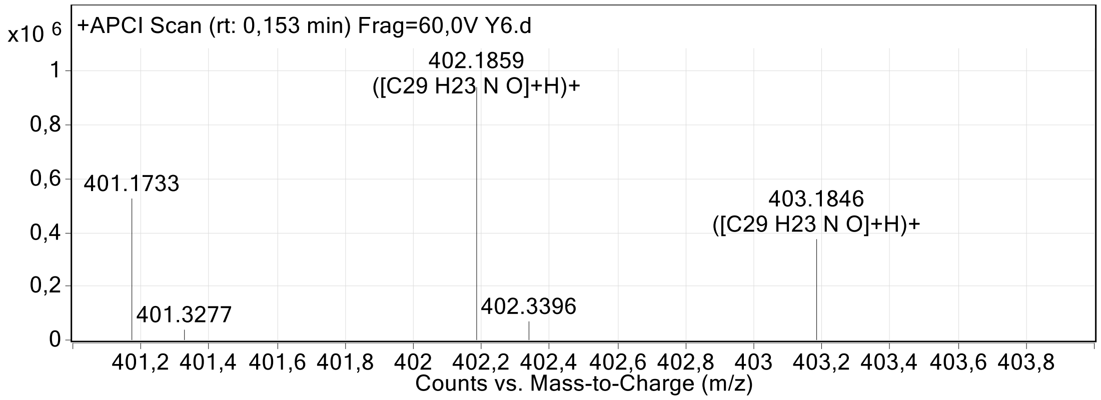

HRMS spectrum of 3g

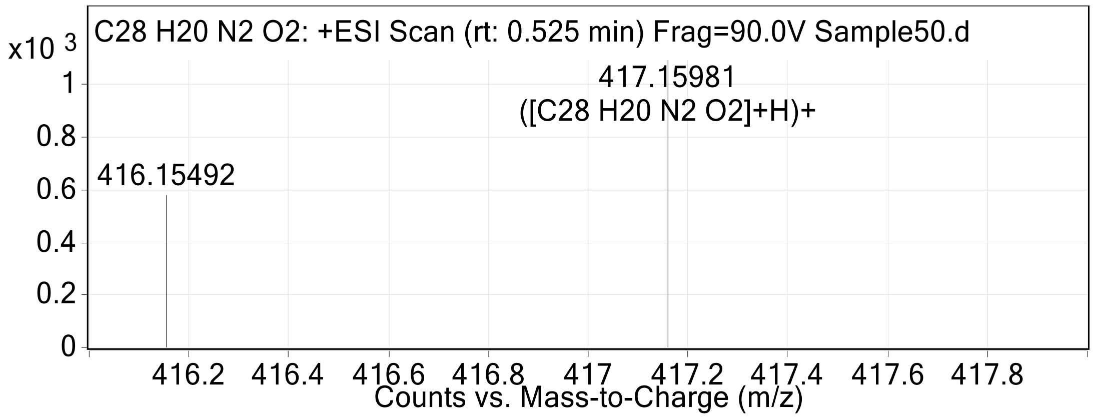

HRMS spectrum of 3i

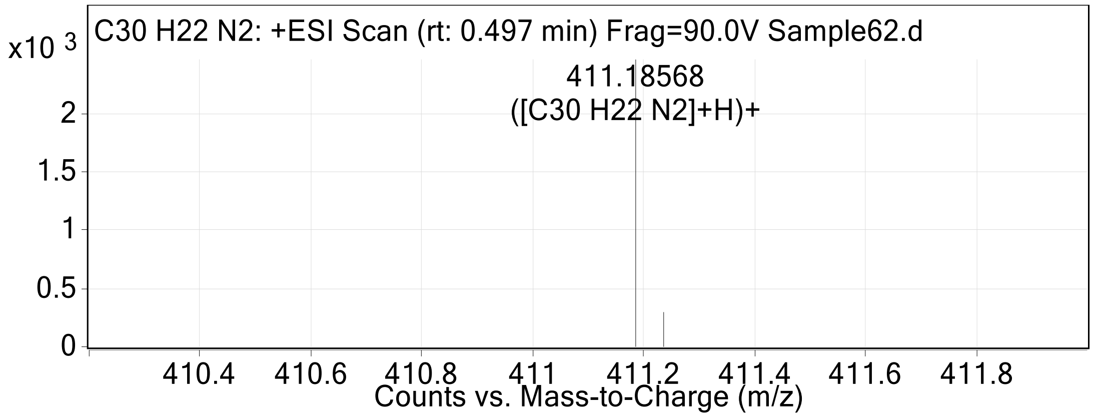

HRMS spectrum of 3k

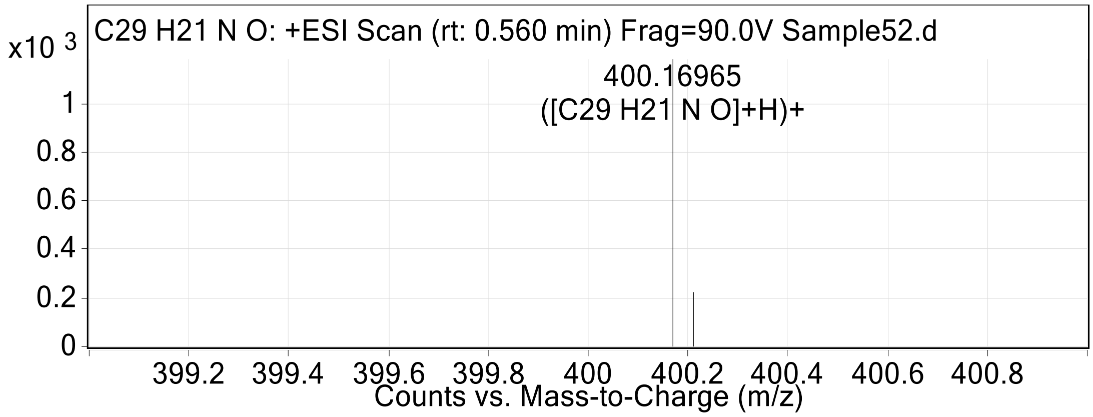

HRMS spectrum of 3l

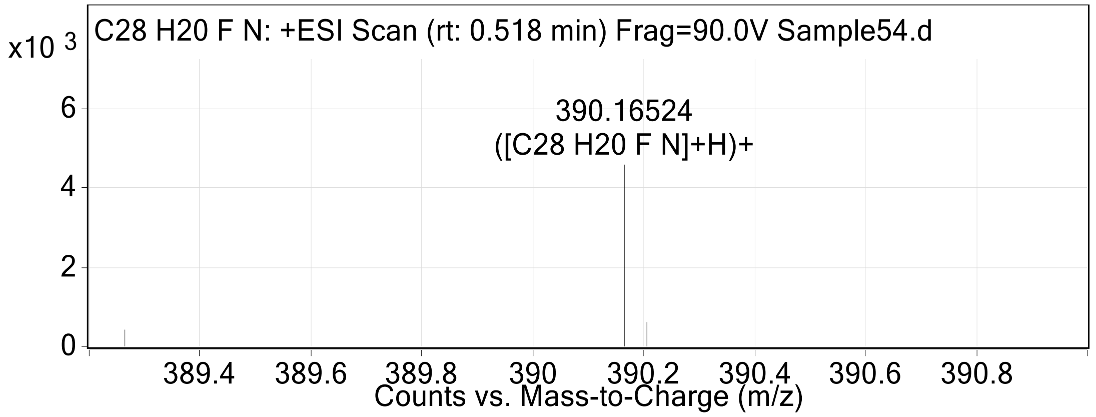

HRMS spectrum of 3m

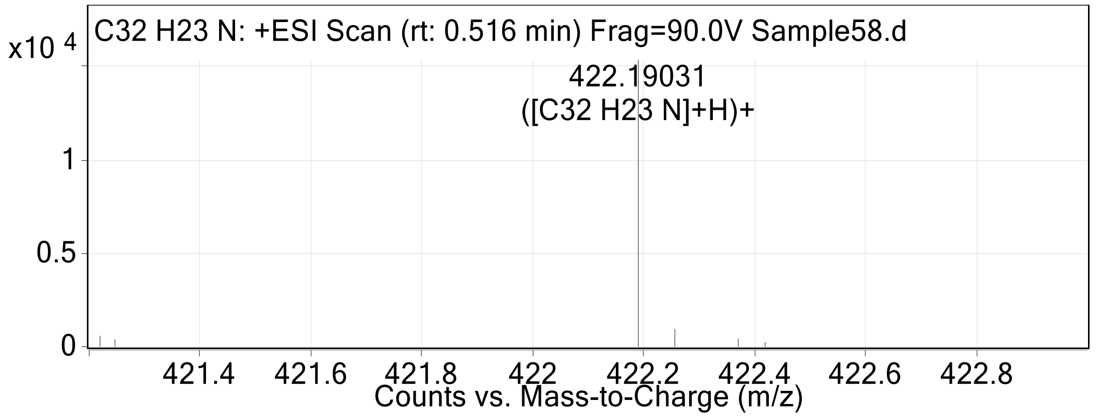

HRMS spectrum of 3h

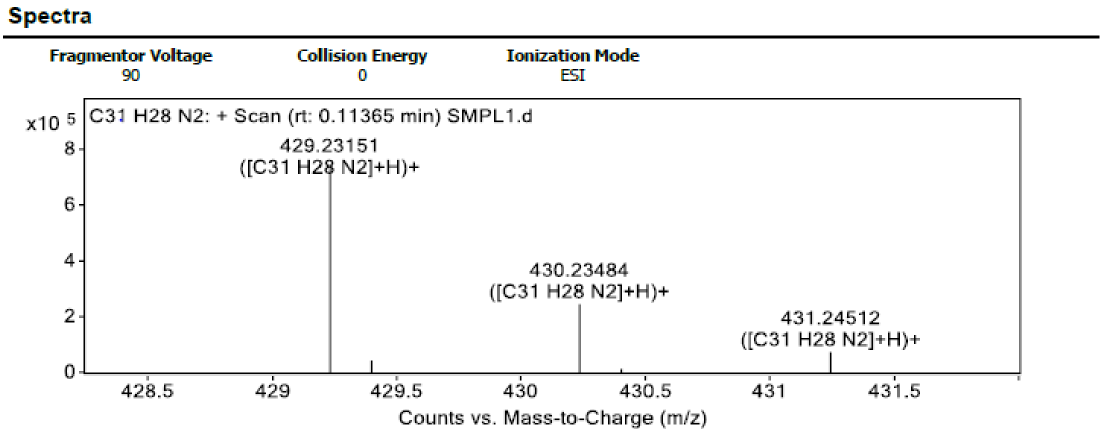

HRMS spectrum of 3b

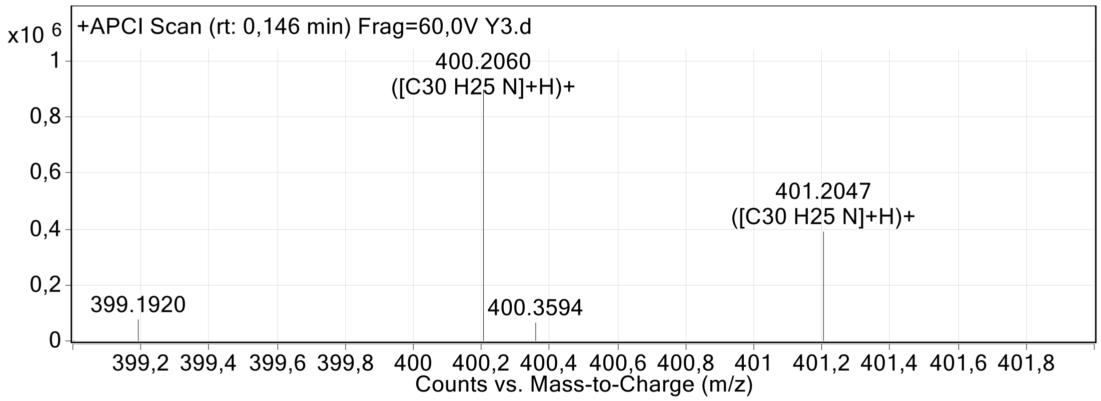

HRMS spectrum of 3c

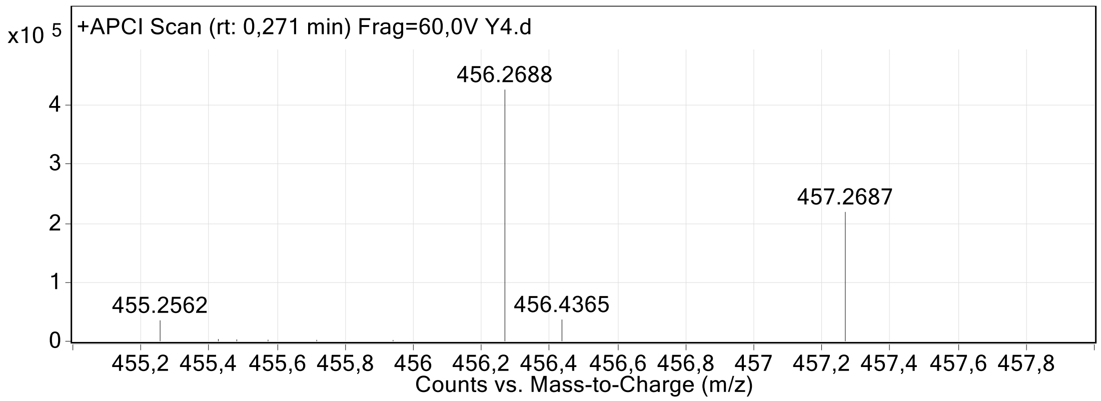

HRMS spectrum of 3e

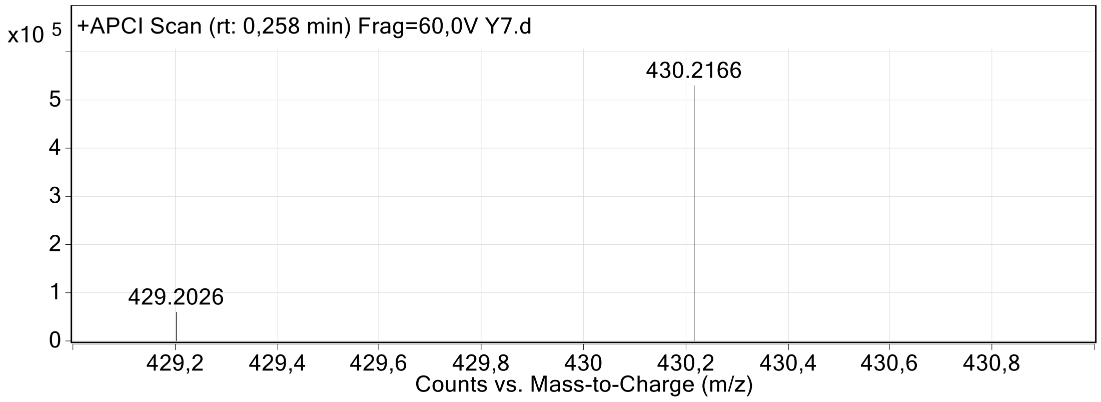

HRMS spectrum of 3f

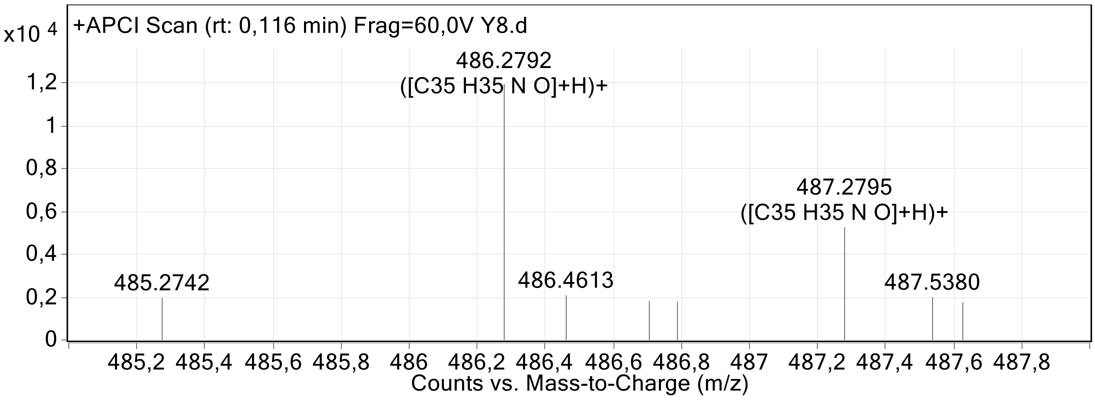

HRMS spectrum of 3n

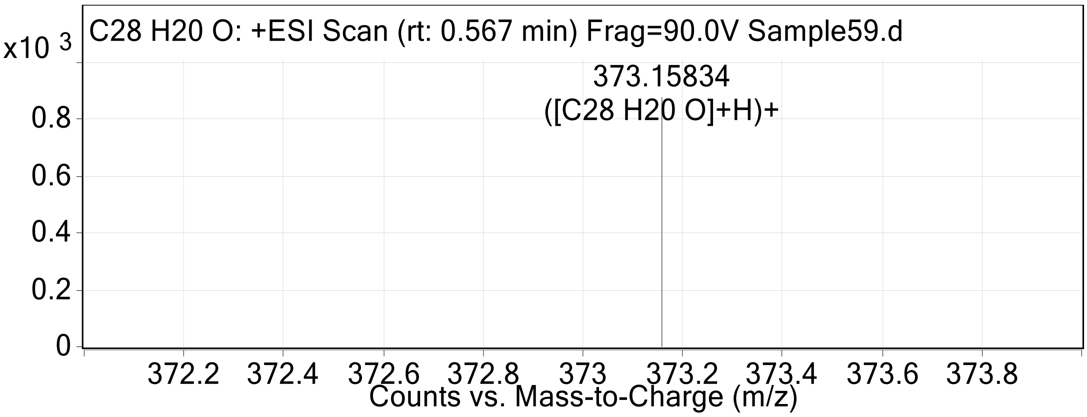

HRMS spectrum of 3p

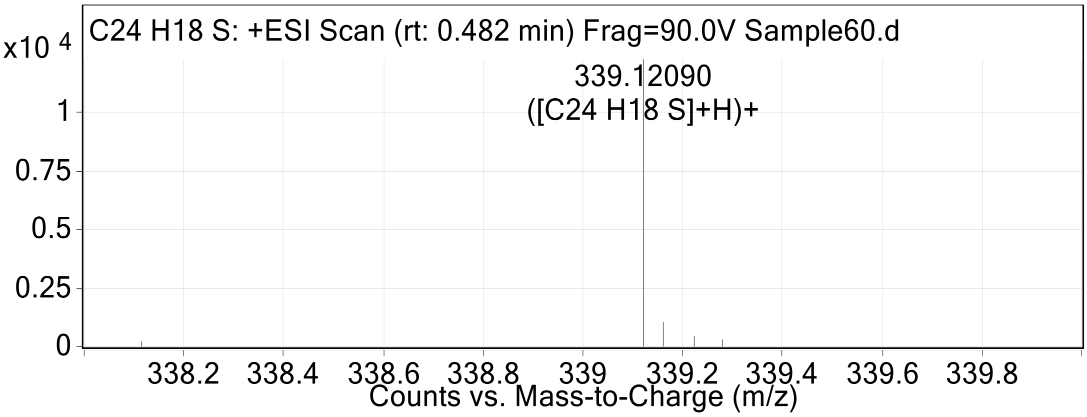

HRMS spectrum of 2a

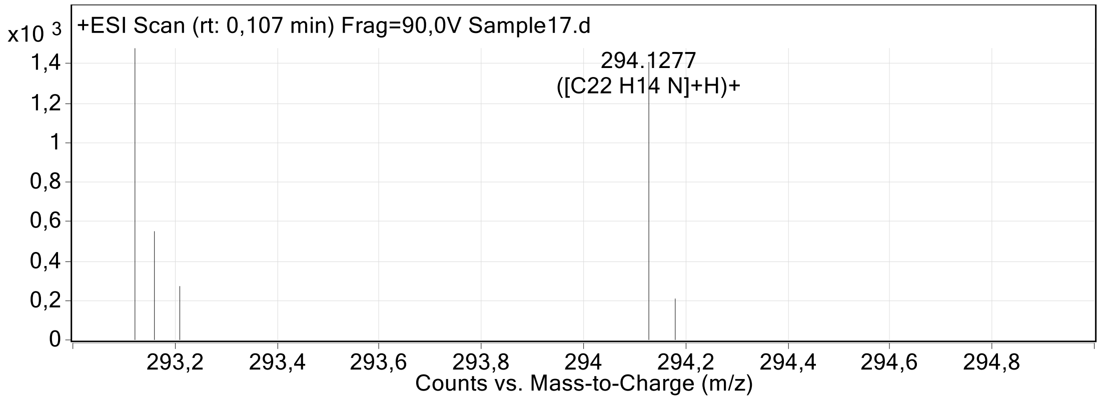

HRMS spectrum of 2b

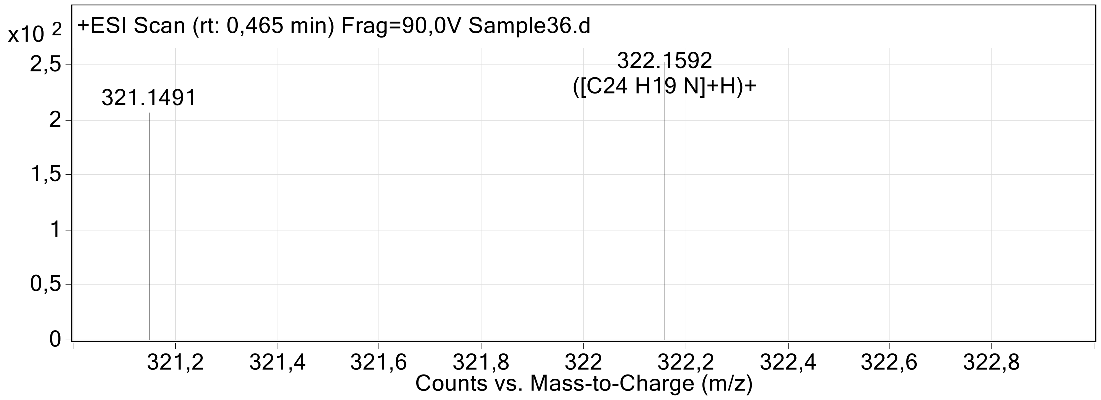

HRMS spectrum of 2c

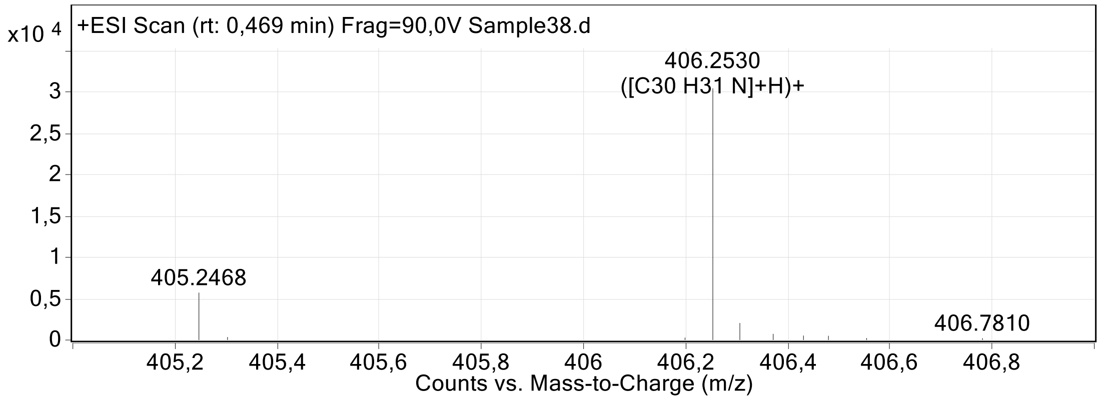

HRMS spectrum of 2d

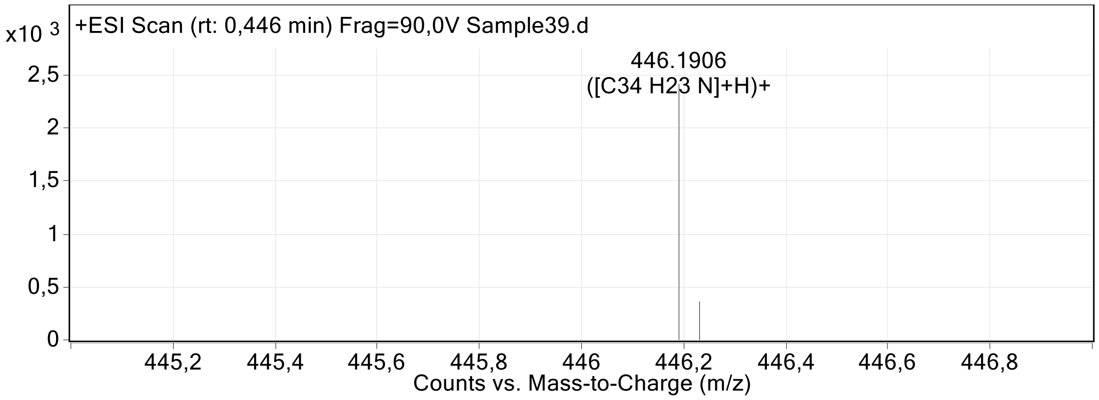

HRMS spectrum of 2e

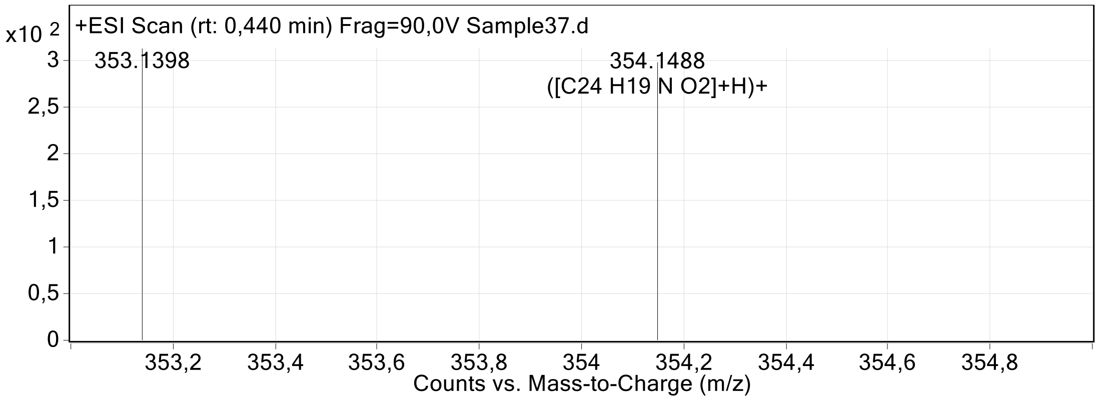

HRMS spectrum of 2f

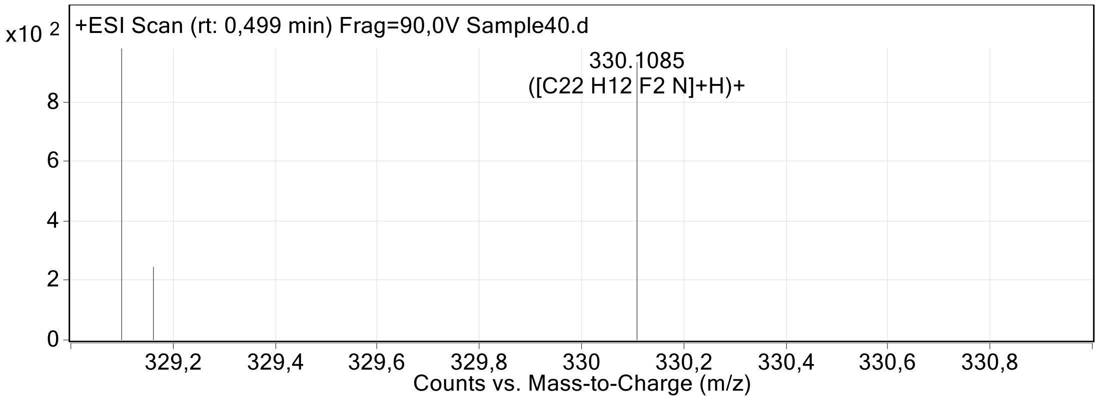

HRMS spectrum of 2g

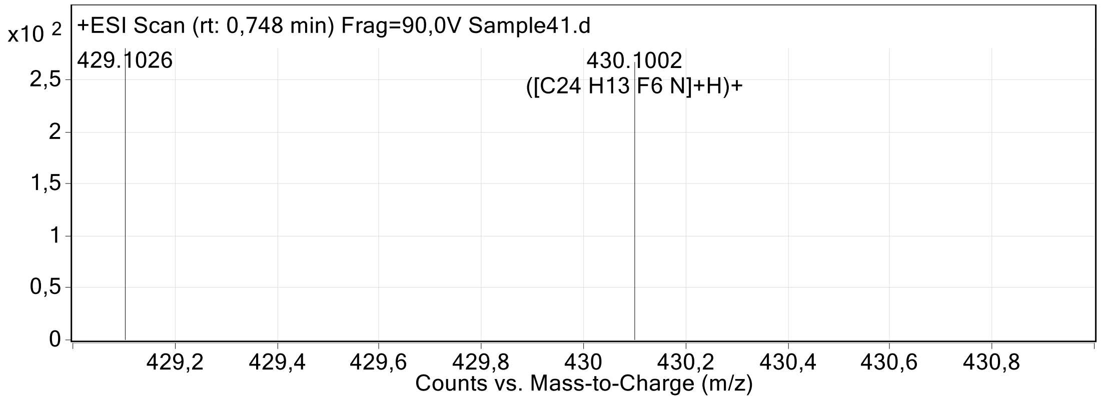

HRMS spectrum of 2h

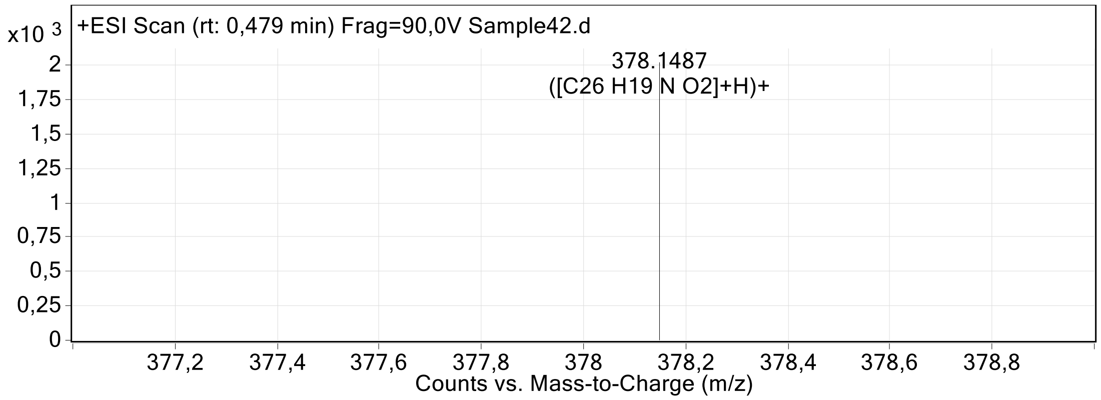

HRMS spectrum of 2i-1+2i-2

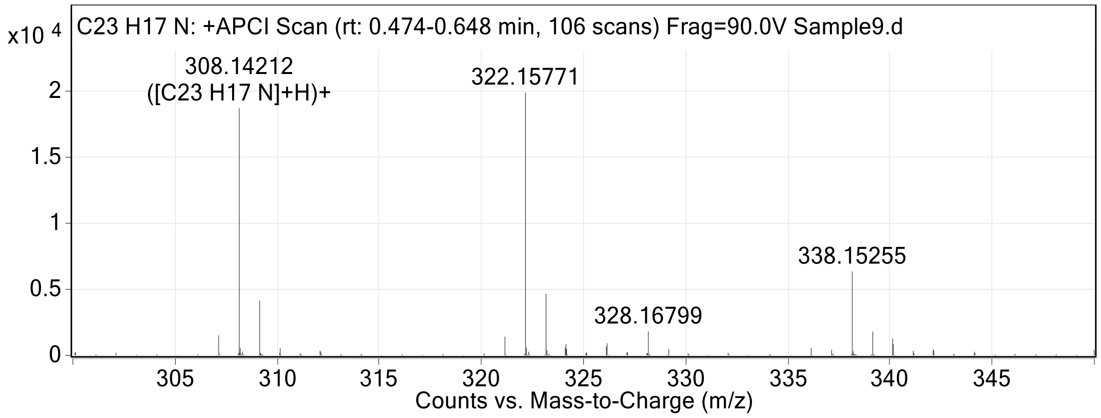

HRMS spectrum of 2j-1

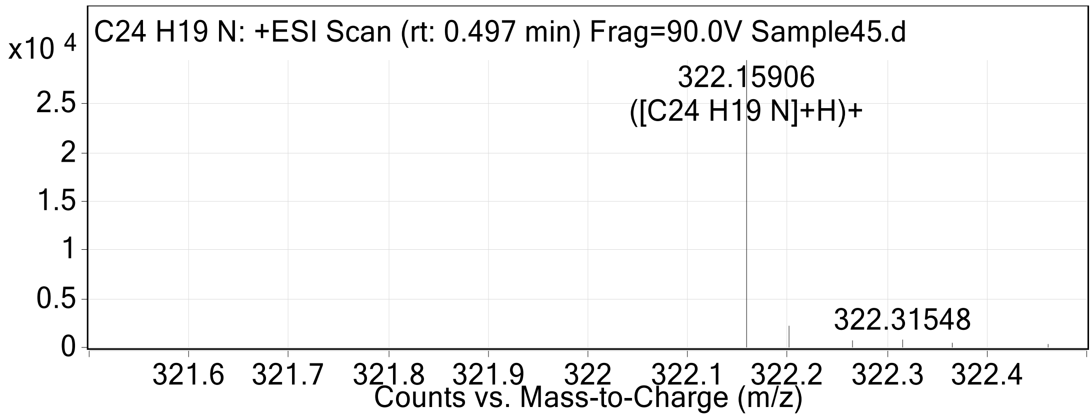

HRMS spectrum of 2j-2

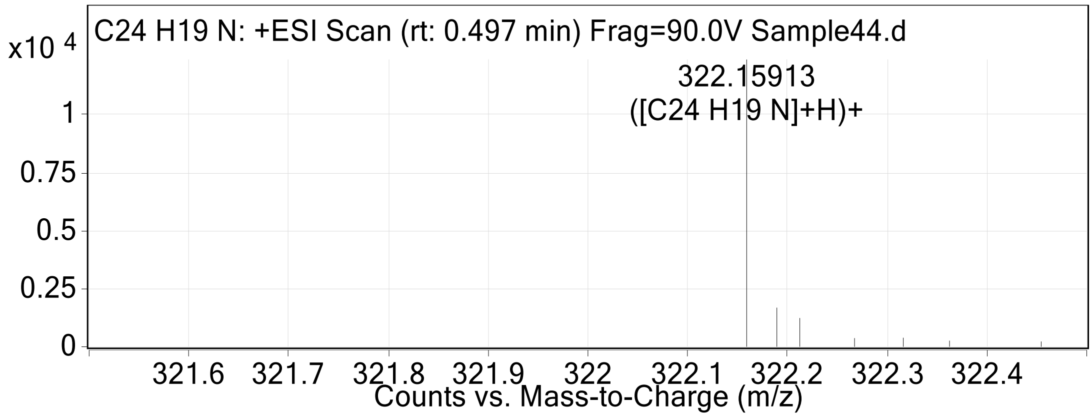

HRMS spectrum of 2l-1

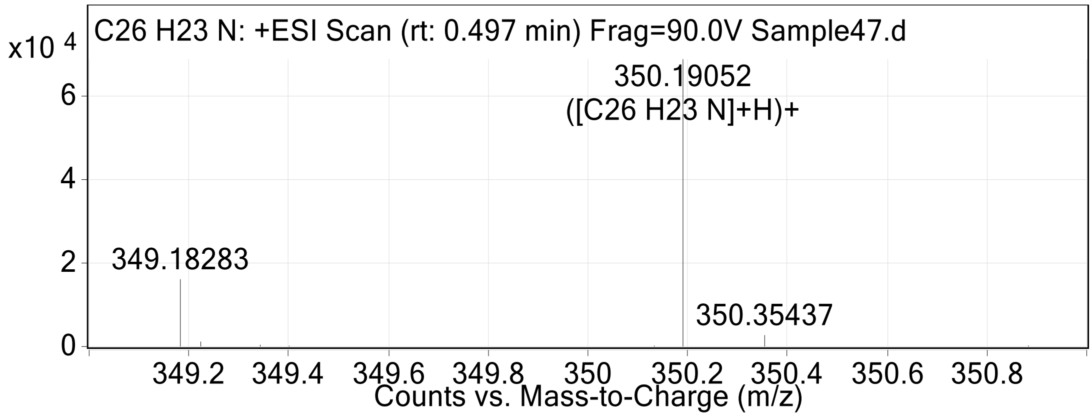

HRMS spectrum of 2l-2

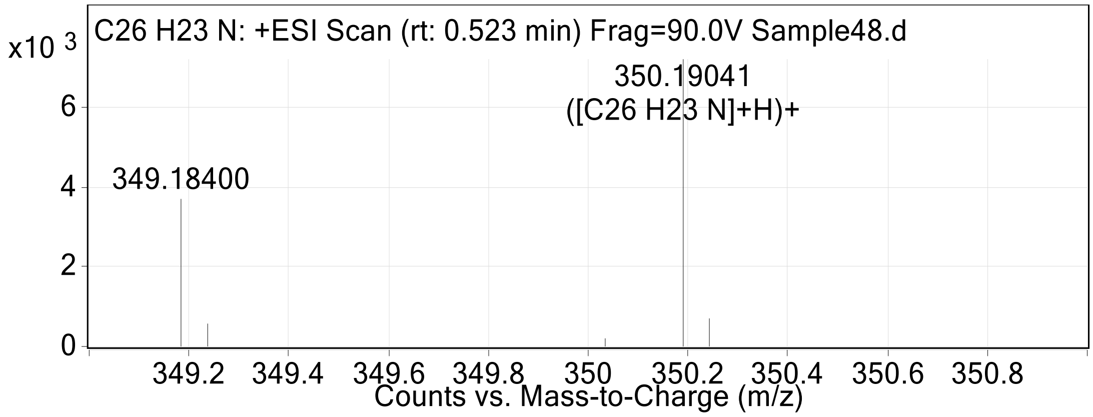

HRMS spectrum of 2m

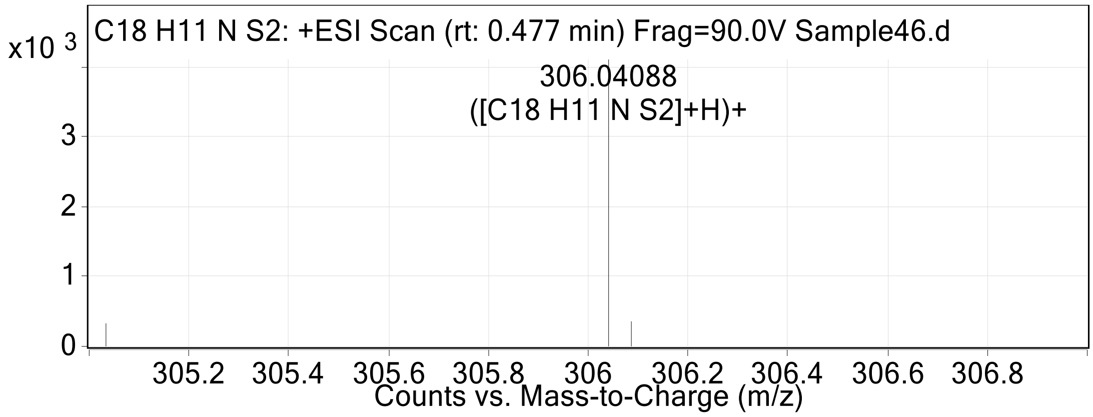

HRMS spectrum of 2n

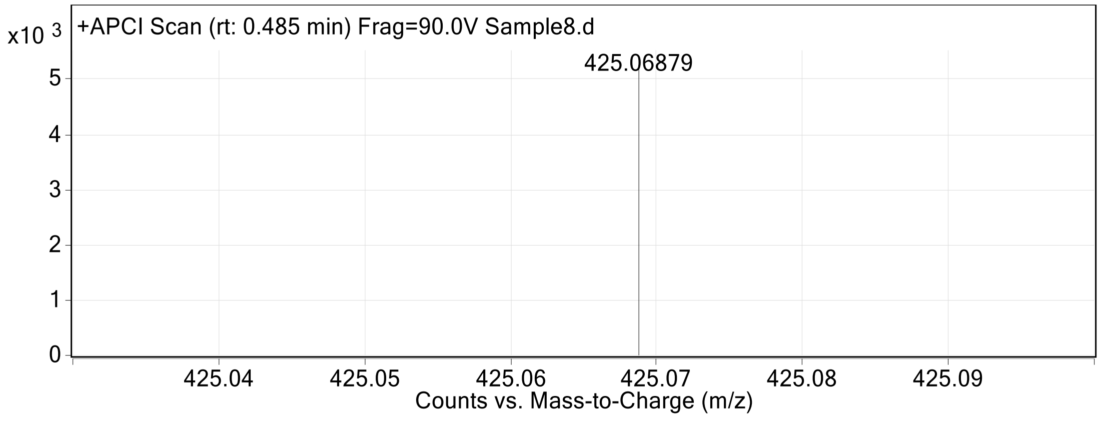

HRMS spectrum of 2o

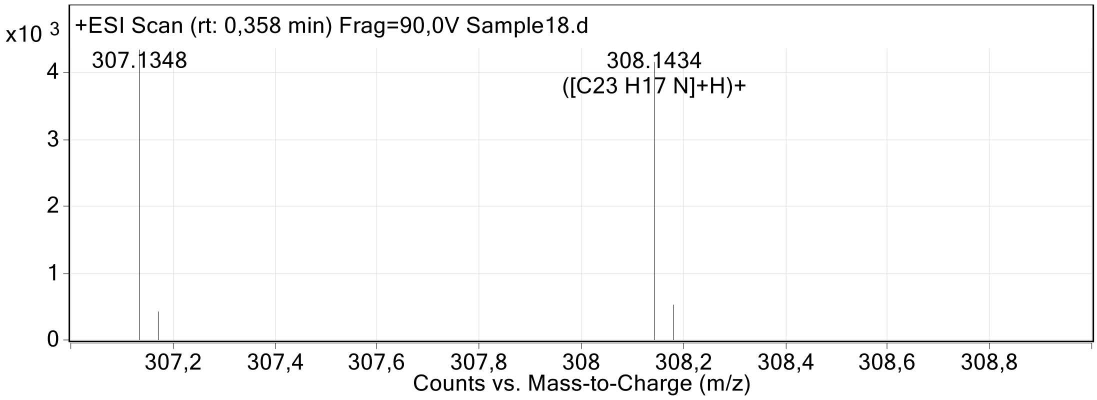

HRMS spectrum of 2p

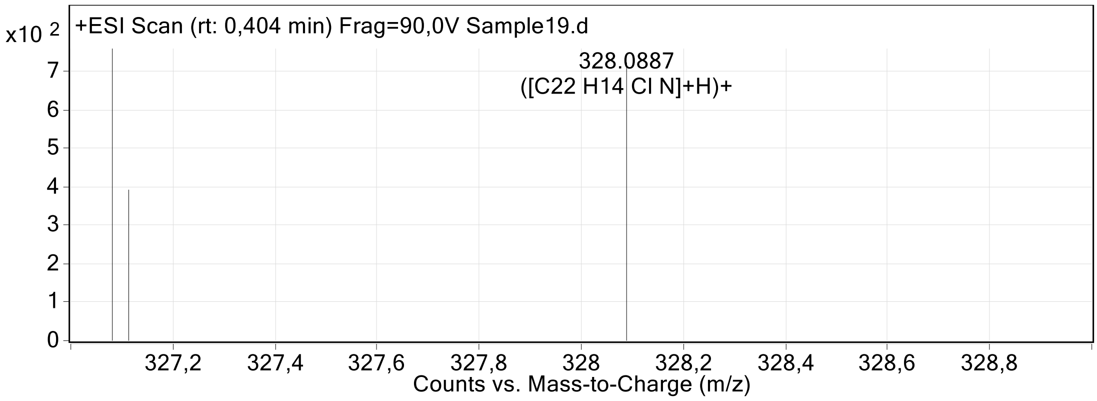

HRMS spectrum of 2q

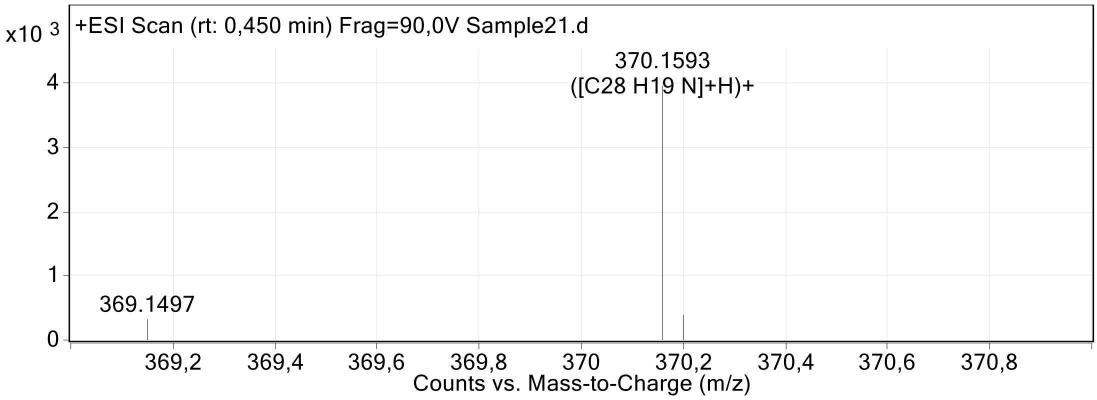

HRMS spectrum of 2r

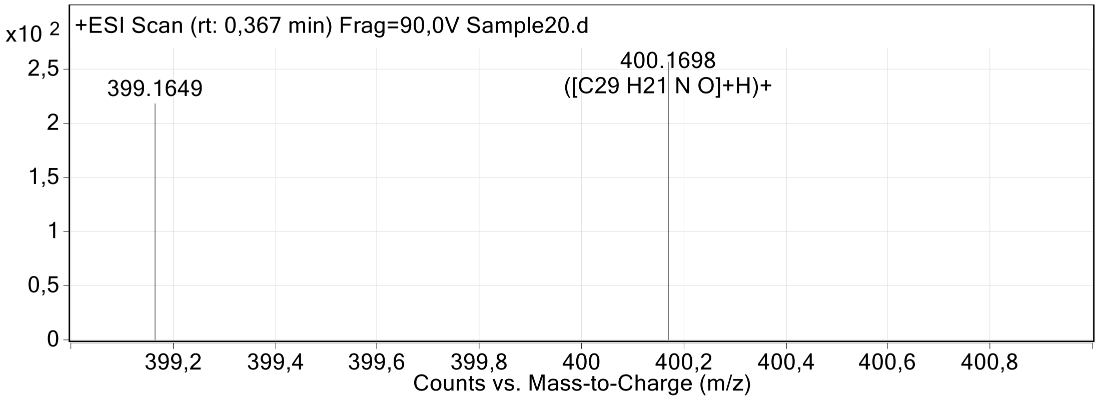

HRMS spectrum of 2t

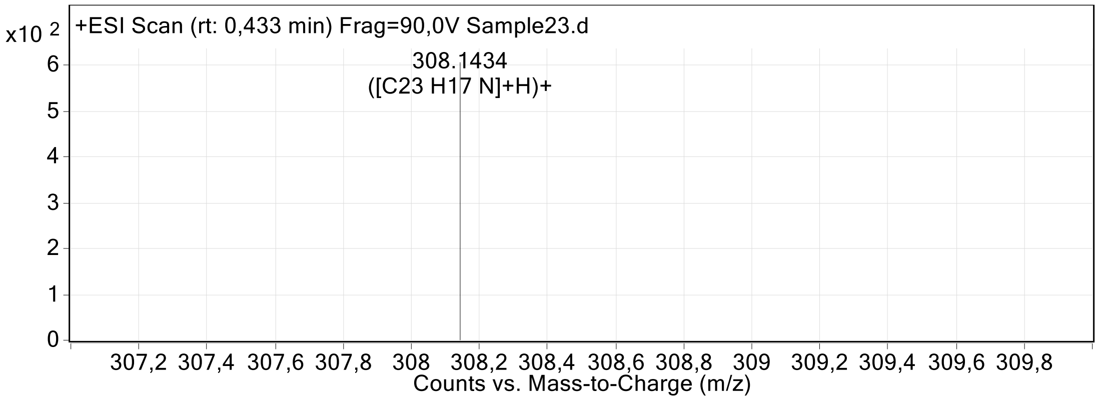

HRMS spectrum of 2u

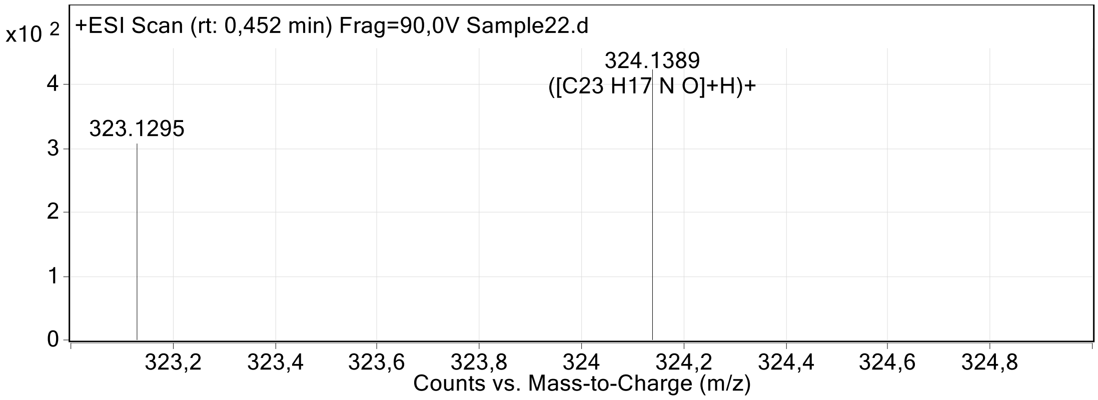

HRMS spectrum of 4a

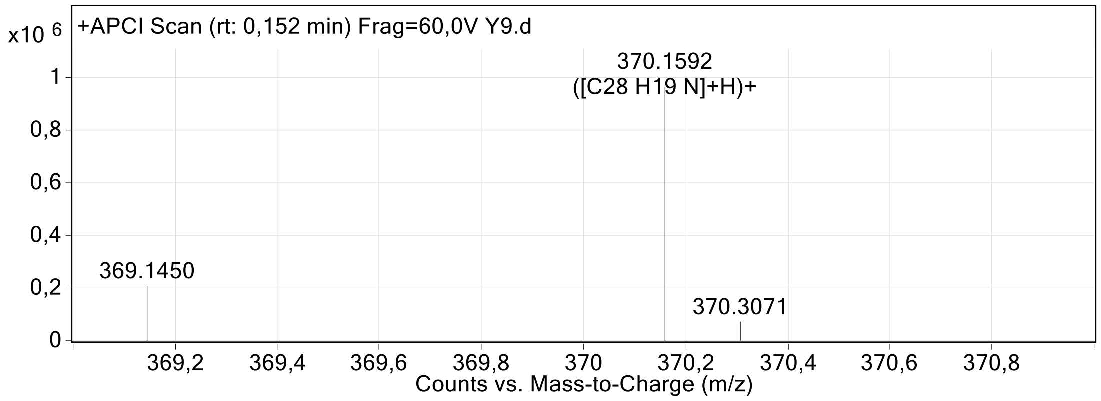

HRMS spectrum of 4b

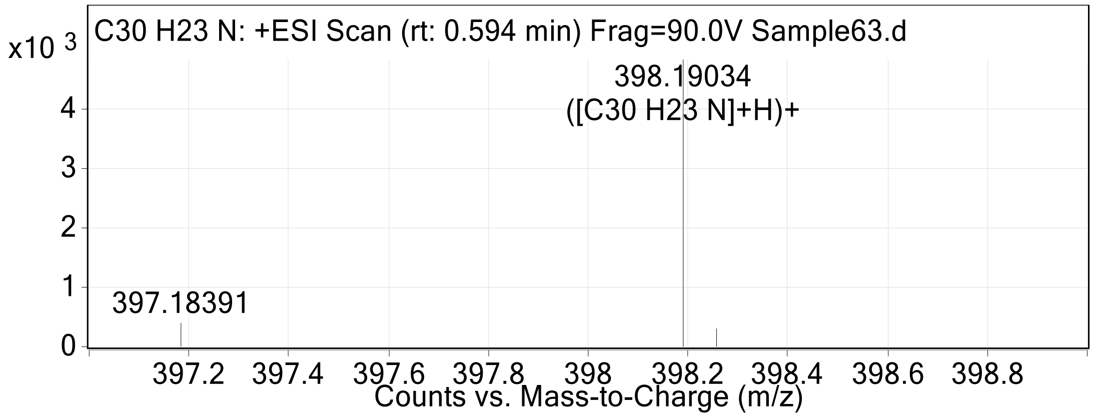

HRMS spectrum of 4c

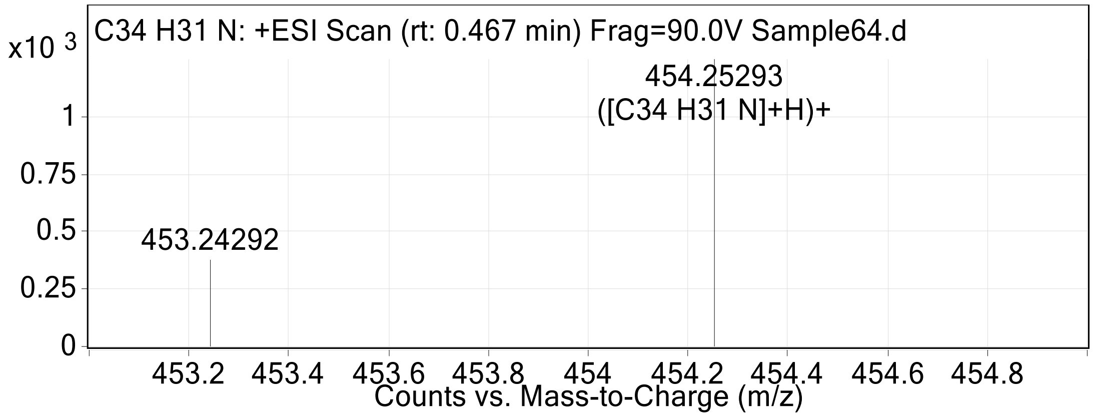

HRMS spectrum of 4d

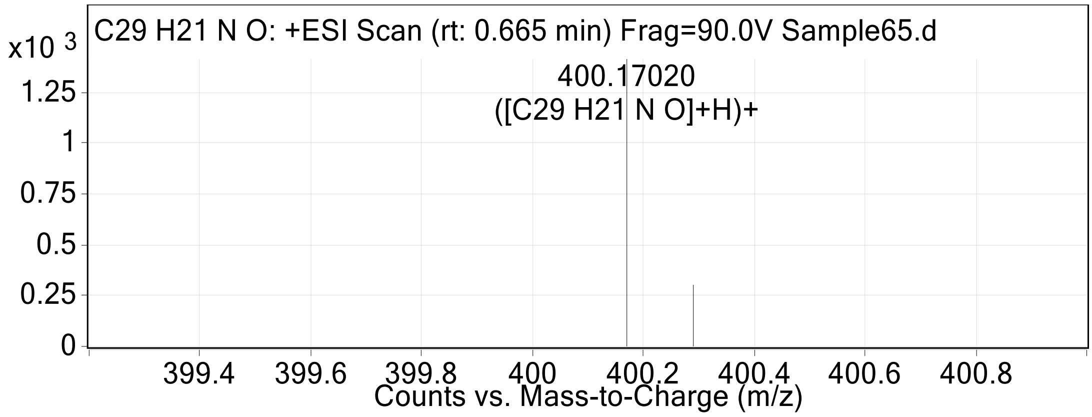

HRMS spectrum of 4e

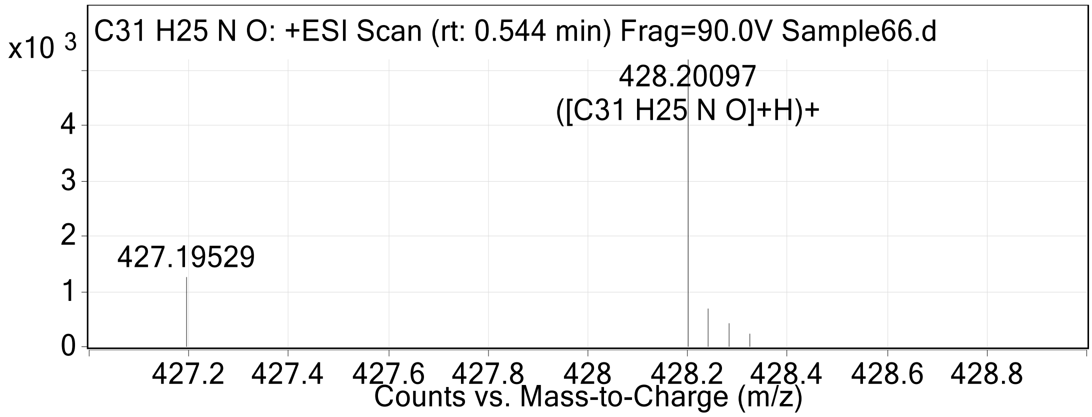

HRMS spectrum of 4f

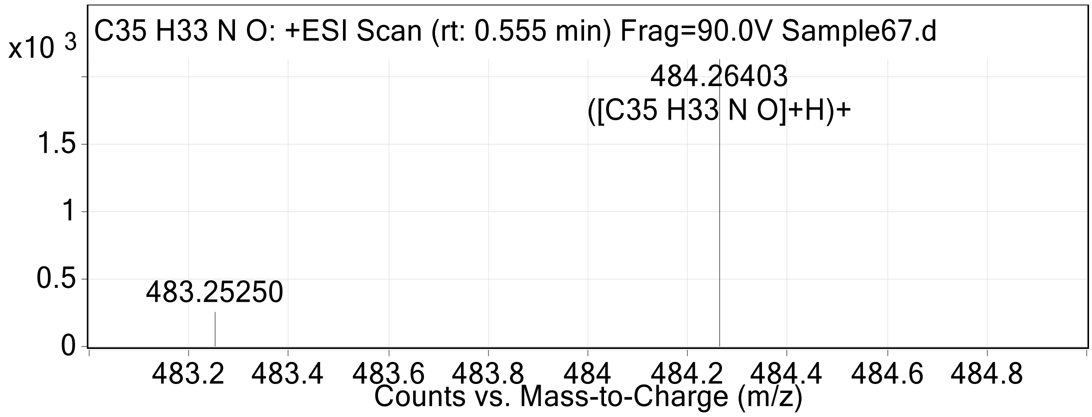

HRMS spectrum of 4h

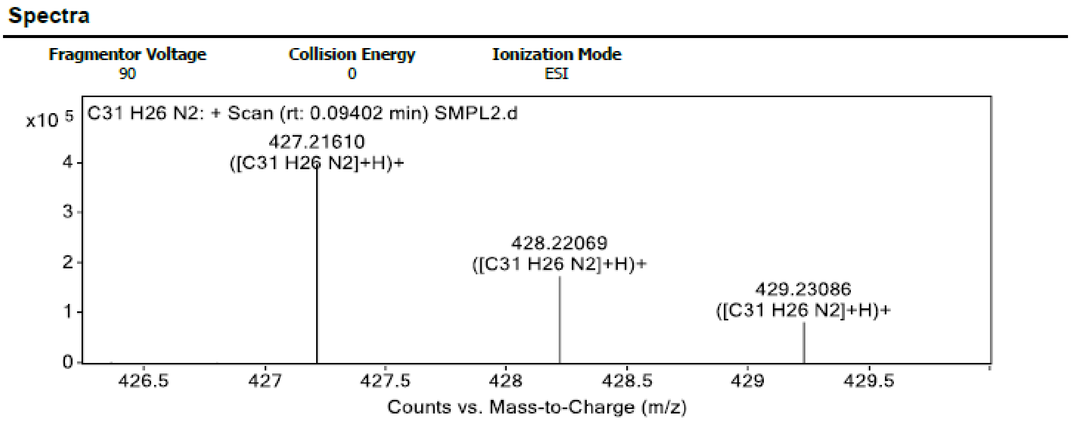

HRMS spectrum of 4i

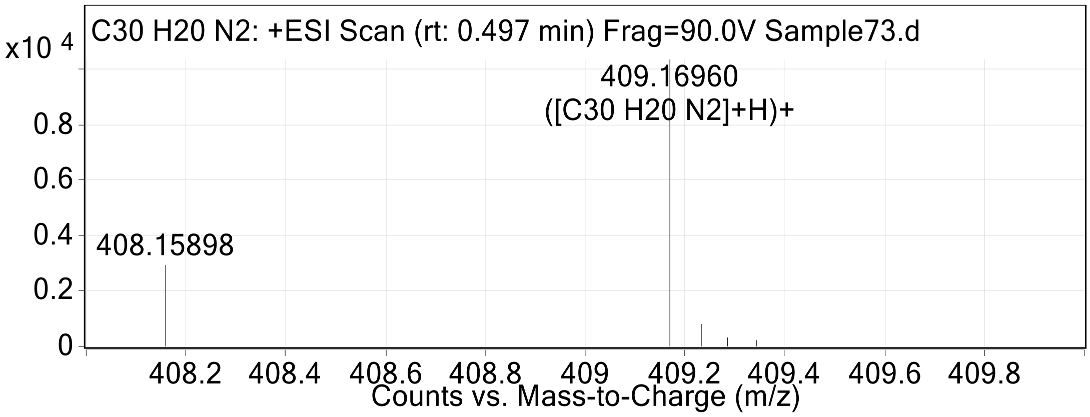

HRMS spectrum of 4j

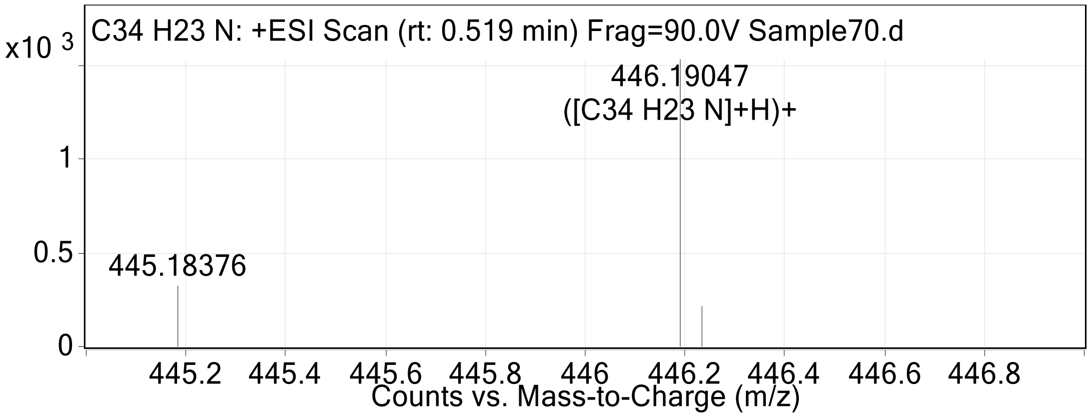

HRMS spectrum of 4k

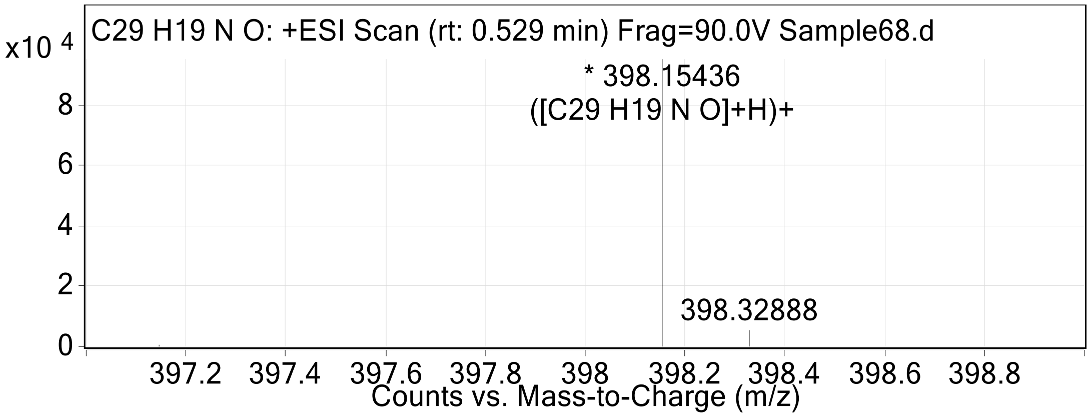

HRMS spectrum of 4l

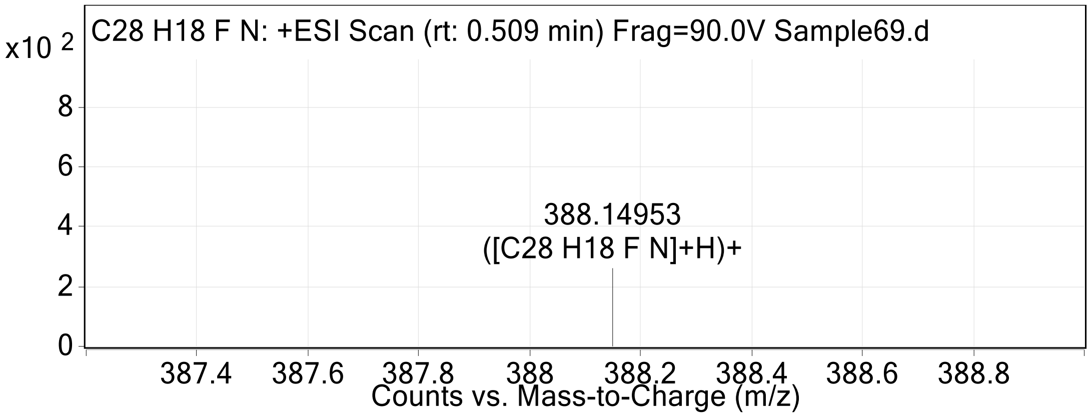

HRMS spectrum of 4m

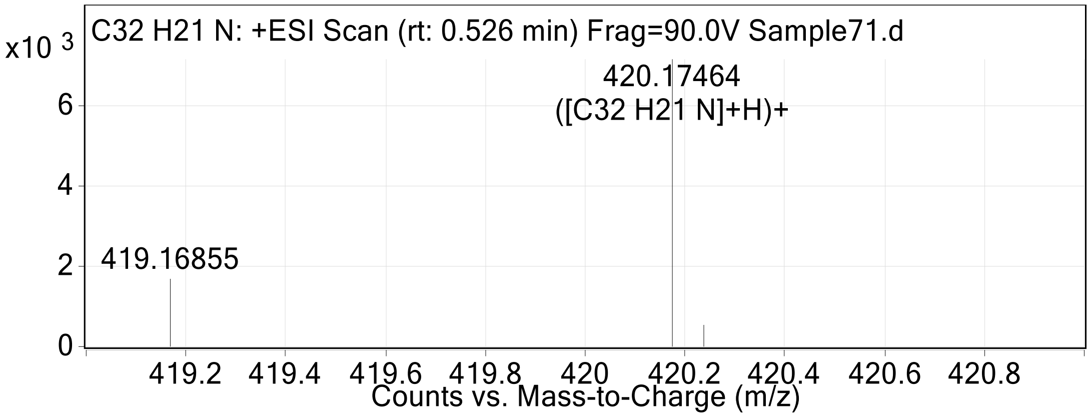

HRMS spectrum of 4n

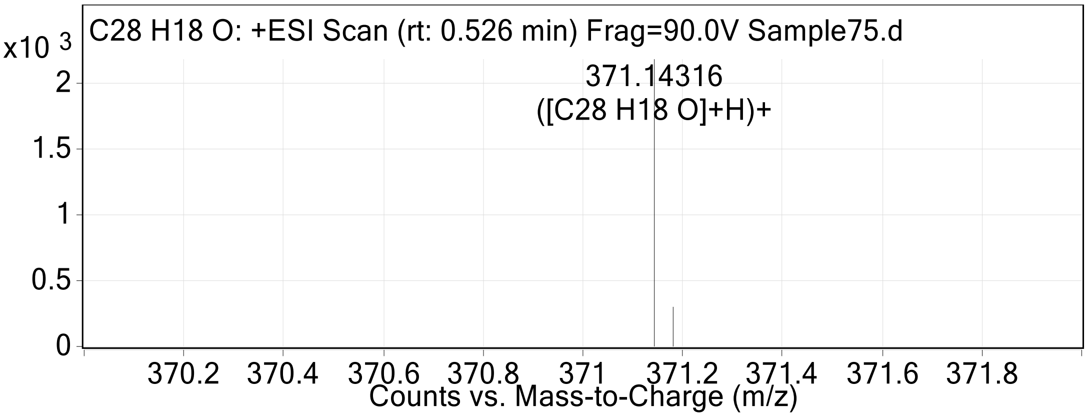

HRMS spectrum of 4o

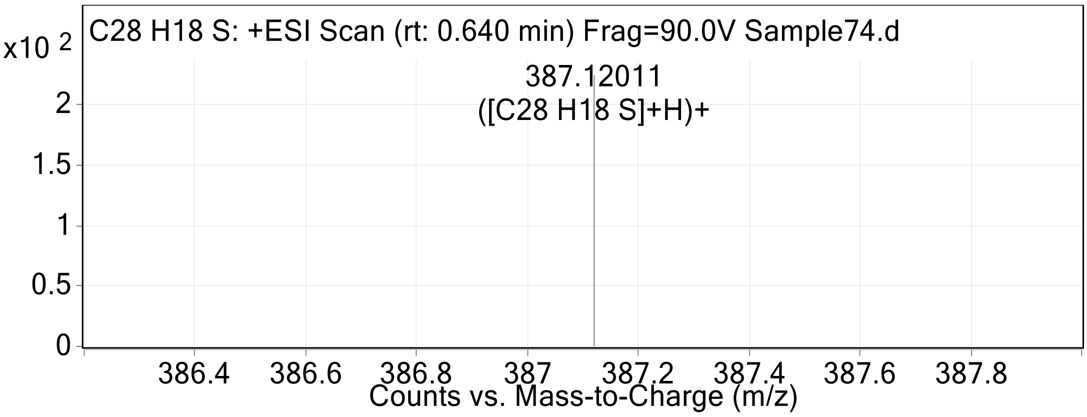

HRMS spectrum of 4p

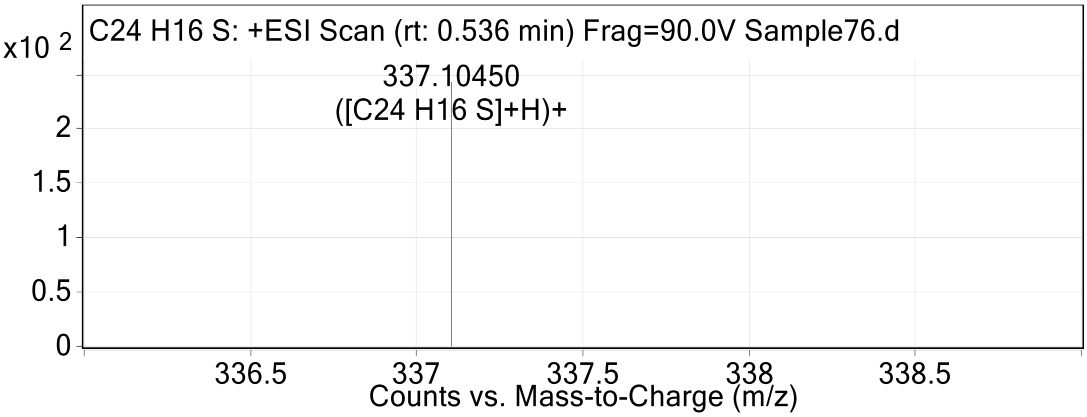

HRMS spectrum of 5

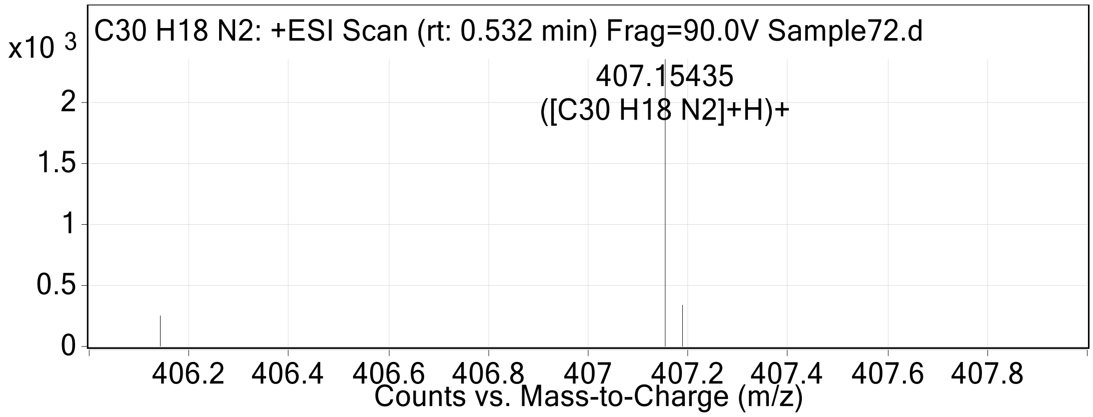

Supplement: Supplementary file 1 — jo4c02103_si_001.pdf [file jo4c02103_si_001.pdf]
